# Reactions of thermally generated benzynes with six-membered *N*-heteroaromatics: pathway and product diversity†
†Electronic supplementary information (ESI) available. See DOI: 10.1039/c9sc03479j


**DOI:** 10.1039/c9sc03479j

**Published:** 2019-08-14

**Authors:** Sahil Arora, Juntian Zhang, Vedamayee Pogula, Thomas R. Hoye

**Affiliations:** a Department of Chemistry , University of Minnesota , 207 Pleasant St., SE , Minneapolis , MN 55455 , USA . Email: hoye@umn.edu

## Abstract

We report here various pathways by which six-membered *N*-heteroaromatic compounds react with benzynes that are generated by the HDDA reaction. The initially formed 1,3-zwitterionic species (a) can collapse intramolecularly to give novel 1 : 1 adducts of the heterocycle and benzyne; (b) can react with an externally added, electrophilic third-component to give functionalized heterocyclic products; or (c) can react with an external protic nucleophile to produce, following collapse of the ion pair resulting from protonation of the zwitterion, a variety of three-component assemblies. Mechanisms for formation of some of the 1 : 1 adducts are supported by DFT methods. The scope of the protic nucleophilic coupling was also expanded to a two-pot operation by using triflic acid as a protic “non-nucleophile”, followed by the addition of a suitably reactive nucleophile.

## Abstract 2

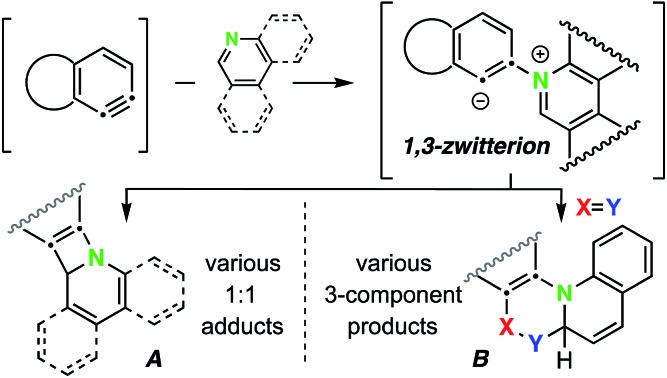
HDDA-benzynes + N-heteroaromatics of the pyridine family in the absence (*cf.***A**) or presence (*cf.***B**) of a third component.

## Introduction

The thermal cycloisomerization of tethered triynes (**I**)‡
‡Throughout the manuscript we have used Roman numerals to label generic or non-isolated structures and intermediates and Arabic numbering for structures of isolated compounds. leads to formation of benzynes (**II**) under neutral conditions in a hexadehydro-Diels–Alder (HDDA) reaction.^1^ These short-lived, reactive intermediates then participate in myriad intermolecular trapping processes, including three-component reactions (TCRs).^2^ In earlier work, we have described a variety of modes of reactivity initiated by the reaction of these electrophilic benzynes with cyclic sulfides,^3^ tertiary amines,^4^ aliphatic cyclic amines,^5^ oxygen-containing heterocycles,^6^ and alkaloidal natural products.^7^

In a number of instances, HDDA-generated benzynes have allowed the discovery of new modes of trapping reactions^6^,^8^ that are distinct from that seen for classically generated arynes (*i.e.*, those formed by way of various *ortho*-elimination reactions of precursor arenes).^9^ This complementarity is one of the major, distinguishing features between classical and HDDA aryne chemistries. These differences in reactivity can be attributed to the fact that the trapping reactions of HDDA-generated benzynes are studied in a “thermal-only” and, thus, pristine environment, devoid of the reagents and byproducts necessary for nearly all classical benzyne-generating protocols.

**Fig. 1:**
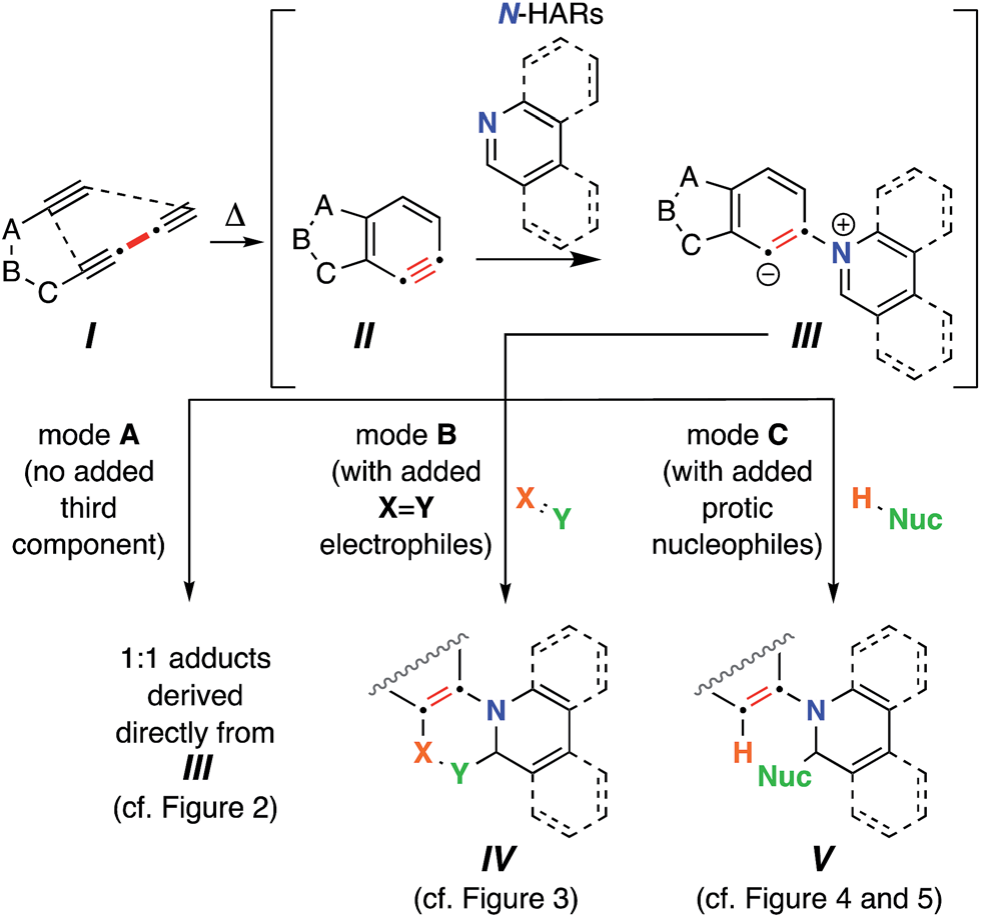
Trapping of HDDA-generated benzynes **II** with *N*-hetaryls (*N*-HARs) of the pyridine family (pyridine, quinoline, isoquinoline, and phenanthridine, *etc.*) *via* diverse reaction pathways (Modes A–C).

Accordingly, HDDA-benzynes provide an opportunity to reveal new modes of reactivity for reagents already known to engage classical arynes. Although there are various examples in which six-membered *N*-heterocyclic arenes (prototypically, pyridine, quinoline, and isoquinoline) have engaged classically generated arynes, nearly all have been performed in the presence of an external, third component to give tractable outcomes.^10^ In those studies, aliphatic nitriles, terminal alkynes, and various carbonyl compounds have been used as third components. This reaction is presumed to be initiated by attack of the nucleophilic nitrogen atom in the heterocycle onto the electrophilic aryne, producing a 1,3-zwitterion (*cf.***III**, Fig. 1 ‡). However, to our knowledge, formation of 1 : 1 adducts between benzynes and *N*-heterocyclic arene compounds (“*N*-hetaryls”, *N*-HARs) has never been definitively demonstrated. Fields and Meyerson (1966) speculated on the intermediacy of [2 + 2]-addition products from arynes and pyridine on the basis of gas chromatographic and mass spectrometric analyses of the complex product mixtures formed when, for example, phthalic anhydride, a benzyne precursor, was pyrolyzed in the presence of pyridine, but discrete products were not isolated.^11^

**Chart 1:**
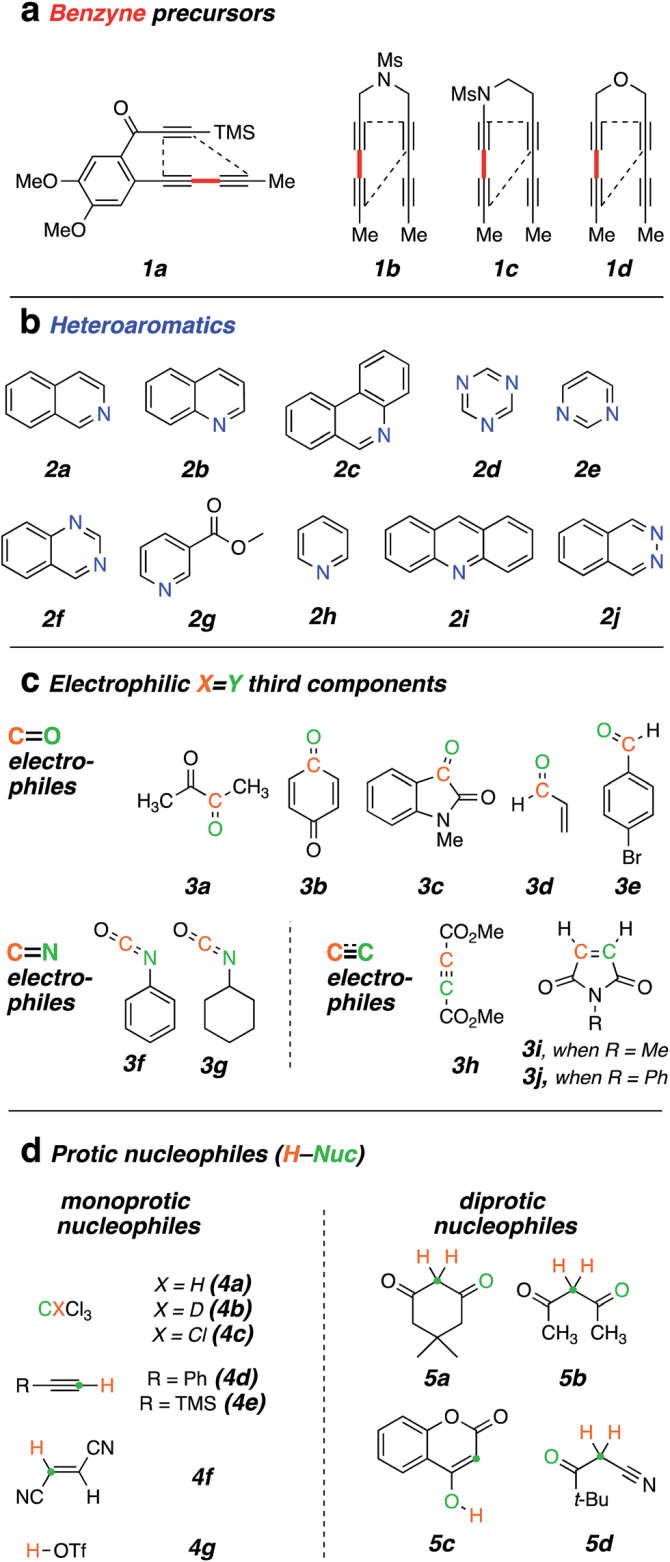
List of all reactants used in this study: (a) benzyne polyyne precursors; (b) *N*-heteroaromatics (*N*-HARs); (c) activated electrophiles; and (d) protic-nucleophiles.

Two factors prompted the investigations reported here: (a) the possibility of identifying reactions leading to 1 : 1 adducts of *N*-HARs and benzynes; and (b) the opportunity to uncover new types of three-component reactions of benzynes with *N*-HARs. We herein report that upon formation of **III**, a variety of reaction pathways can ensue. The reactions that we have observed for zwitterions such as **III** can be categorized into three modes. Mode A leads to products that are 1 : 1 adducts between **II** and the *N*-HAR. In mode B, *in situ* bimolecular reaction of zwitterion **III** with an electrophile of type 
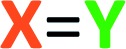
 as a third component forms a new 6-membered ring in products **IV**. In mode C, protonation of **III** by a Brønsted acid [*i.e.*, an *in situ* protic nucleophile (H-Nuc), the third component] and collapse of the nascent Nuc^–^/iminium^+^ ion pair produces adducts **V**. The full roster of reactants used in the studies we report here are compiled in Chart 1.

## Results

### Mode A (formation of a 1 : 1 adduct between an aryne and a *N*-HAR)

**Fig. 2:**
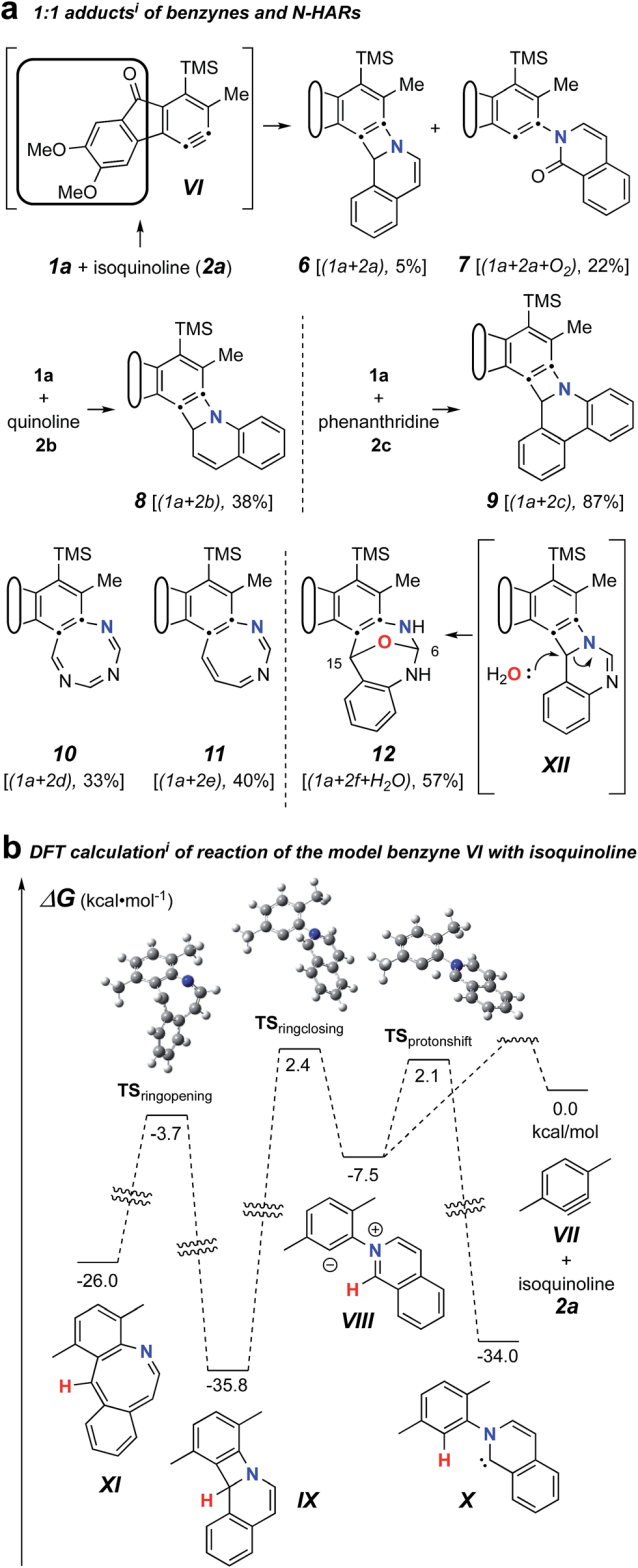
(a) Trapping of the benzyne **VI** (from **1a**, Chart 1) with *N*-hetaryls of the pyridine family (*i.e.*, pyridine, quinoline, isoquinoline, phenanthridine, pyrimidine, 1,3,5-triazine, and quinazoline) follow different reaction pathways. (b) DFT^i^ for **VII** + **2a** showing competitive formation of **IX** and **VIII** and a high barrier for ring opening of **IX** to the azocine **X**. ^*i*^[SMD(benzene)//M06-**2X**/6-311+G(d,p)].

We first examined reactions of the triyne substrate **1a** with several *N*-HARs in the absence of any added, potential third component. For example, when a benzene solution of **1a** was heated to 85 °C in the presence of isoquinoline (**2a**), the four-membered benzoazetidine **6**§
§The structures of isolated products bear a unique Arabic number. That number is followed (in brackets) by a listing of the reactants that have been incorporated into the compound (*cf.*Chart 1): polyyne **1** is the benzyne precursor, **2** the *N*-HAR, **3** an electrophilic third component, **4** a monoprotic-nucleophile, and/or **5** a diprotic nucleophile. and 2-isoquinolone **7**§ were formed (Fig. 2a) by *in situ* trapping of **VI** by **2a**. Probably because of the oxidative lability of product **6**, it was found to be unstable under ambient conditions and needed to be characterized immediately after isolation.

The pathways leading to each of these products were then studied using DFT calculations of the reaction between a simplified aryne [3,6-dimethylbenzyne (**VII**)] and isoquinoline (**2a**) (Fig. 2b). The formation of **6** can be explained by a net [2 + 2]-addition of **2a** to the benzyne, a process involving simple cyclization of the initial zwitterion (*cf.***VIII** to **IX**). Formation of **7**, on the other hand, can be rationalized by an intramolecular proton-transfer within the initially formed 1,3-zwitterion **VIII** and subsequent oxidation of the resulting carbene **X** by oxygen.^12^ This type of reactivity was earlier reported by Biju and coworkers.10*c* It is notable that the energies of the DFT transition structures for these two competing pathways (*i.e.*, TS_ringclosing_*vs.* TS_protonshift_) are quite similar. The computations also suggest that electrocyclic ring-opening of the benzoazetidine **IX** to the 8-membered benzoazocine derivative **XI** is both endergonic as well as a high-barrier process, the latter reflecting, at least in part, the loss of benzenoid aromaticity that is fully revealed in structure **XI**.

Trapping of the benzyne **VI** with quinoline (**2b**) led to the isolation of only the four-membered species **8**. This compound also was seen to degrade upon storage for extended periods of time. An analogous set of computations using quinoline **2b** showed a lower barrier (by 2.3 kcal mol^–1^) favouring formation of azetidine relative to carbene (see Fig. S1†). Accordingly, a 2-quinolone product was not observed to accompany **8**. When phenanthridine (**2c**) was used as the *N*-HAR, the yield of the analogous trapping product **9** was significantly higher (87%) than that of **6** or **8**.

Reactions with monocyclic *N*-HARs containing two or more nitrogen atoms proved interesting. 1,3,5-Triazine (**2d**) gave rise to the 1,3,5-triazocine derivative **10**;^13^ we can locate only a handful of examples of this (fully unsaturated) heterocycle, and they all bear an amino substituent on the carbon atom between two nitrogen atoms (*i.e.*, guanidines).^14^ Compound **10** presumably arises from ring opening of an initially formed, four-membered, [2 + 2]-adduct analogous to **IX** (*cf.***i** 15). In this case, that strain-relieving event is not accompanied by loss of aromaticity, as would be the case for the opening of any of **6**, **8**, or **9**. DFT calculations suggest that the electrocyclic ring-opening of **i** 15 [see ESI Fig. S2†] has both an accessible activation barrier (Δ*G*^‡^ = 20.7 kcal mol^–1^) as well as a favourable exergonicity (Δ*G*° = –17.1 kcal mol^–1^), in contrast to the conversion of **IX** to **XI** (Δ*G*° = +9.8 kcal mol^–1^).

Pyrimidine (**2e**) reacted by a similar pathway to produce **11**, preferring to close to C4 rather than C2 to give **ii**.^15^ The closure of **ii** at C4 maintains some of the N1–C2–N3 growing amidine character and stabilization throughout the reaction coordinate, which would be lost if cyclization were to take place at C2. Again, the diazocine **11** represents a relatively rare class of heterocycle, examples appearing in only four reports.14c,^16^

Finally, quinazoline (**2f**) gave rise to the unusual adduct **12**, incorporating a molecule of adventitious water. We suggest that in this instance, ring-opening of the initial benzoazetidine **XII** is assisted by water, the C–N bond being weakened by virtue of the amidine character of **XII** that is absent in the benzoazetidines from quinoline (**2b**) or isoquinoline (**2a**, *cf.***IX**). The structure assignment of **12** was secured by the clear HMBCs observed for both H6 (*δ* 6.18 ppm) to C15 (*δ* 66.8) and H15 (*δ* 6.67) to C6 (*δ* 82.1) as well as the chemical shifts of those four nuclei.

### Three-component reactions

Modes B and C offer the potential to produce structurally complex, polycyclic structures in a single, one-pot, thermal operation. As mentioned, the former (mode B) is precedented^10^ for the case of certain carbonyl compounds but not for other types of electrophilic species. The latter (mode C) has been reported only for classical benzyne trapping with *N*-HARs in the presence of various terminal alkynes, carbonyl compounds and aliphatic nitriles, as the protic nucleophiles.10*b*

### Mode B (TCR with *N*-HAR + X=Y electrophile)

A successful mode B TCR (*cf.*Fig. 1) would likely need to meet the following criteria: (i) neither the *N*–HAR nor the 
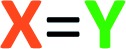
 electrophile should react with the HDDA polyyne precursor faster than its rate of cyclization to the benzyne intermediate (the rate-limiting event); (ii) the *N*–HAR should engage the benzyne faster than it reacts with (at least irreversibly) the electrophile (
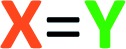
); (iii) the electrophile should be sufficiently reactive to trap the 1,3-zwitterionic species (*cf.***III**) to give products like **IV** before the former undergoes a ring-expansion process such as those discussed above in the mode A results.

We screened different electrophiles that might meet these requirements. Reactions were performed in benzene (or in a cosolvent of benzene and acetonitrile, depending on the solubility of the reactants) using an initial concentration of aryne precursor **1** of 0.02 M. Given the ready availability of all of the trapping components explored, we opted to use a stoichiometric ratio of the three reactants of, typically, 1 : 3 : 5 (benzyne precursor **1**: *N*-HAR **2**: electrophile **3**); this choice was not further optimized.

**Fig. 3:**
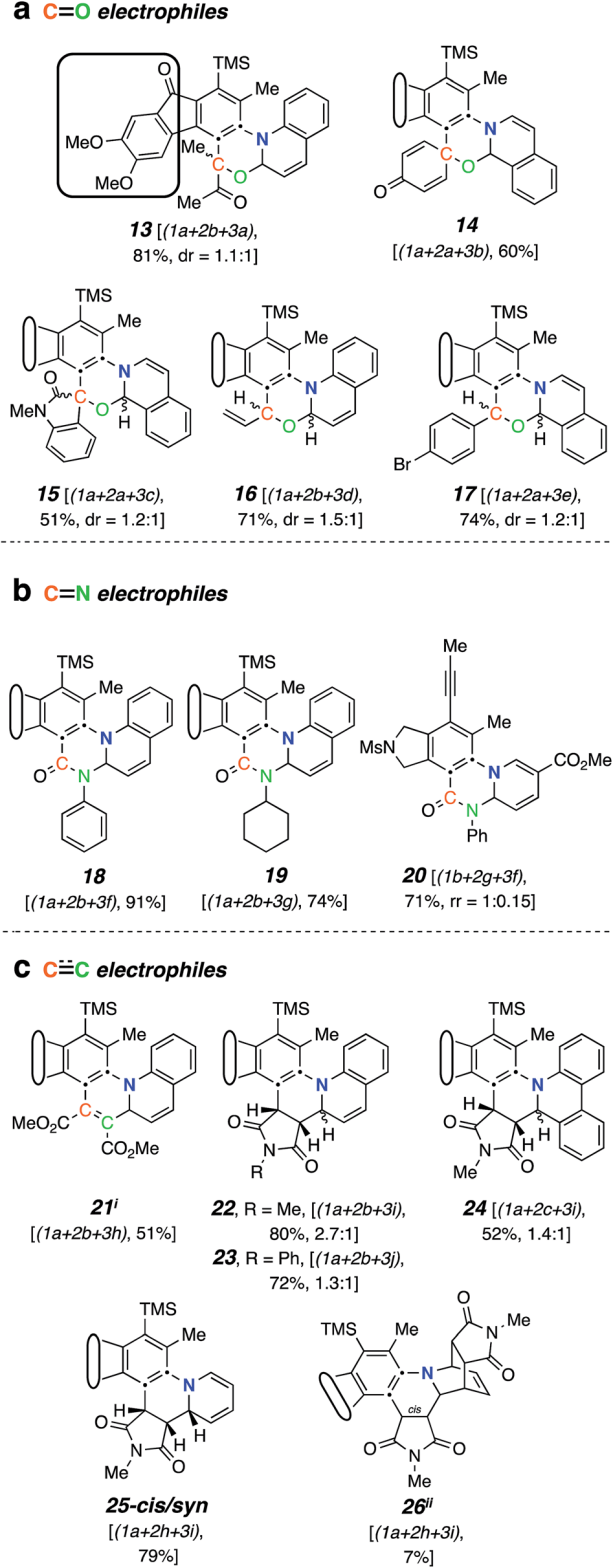
Products from mode **B** three-component reactions. Trapping of zwitterions (*cf.***III**) by (a) carbonyl electrophiles, (b) isocyanates, and (c) electron-deficient alkenes or alkynes. ^i^A byproduct comprising two molecules of dimethyl acetylenedicarboxylate and one of quinoline was isolated (in *ca.* equal mass to that of **21**, see ESI†). ^ii^Relative configuration not assigned.

Aliphatic aldehydes and ketones (*e.g.*, butyraldehyde and methyl vinyl ketone) did not perform well as a TCR third component. These coupling partners may not be sufficiently electrophilic. Upon switching to biacetyl (**3a**), we observed efficient formation of **13** (Fig. 3a) as a mixture of diastereomers in 81% yield. In this reaction triyne **1a**, quinoline (**2b**), and **3a** were heated at 85 °C for 16 h. Other reactive carbonyl compounds could also serve as a third component, as demonstrated by products **14**, **15**, **16**, and **17**, which arise from incorporation of *p*-benzoquinone (**3b**), *N*-methylisatin (**3c**), acrolein (**3d**), or *p*-bromobenzaldehyde (**3e**),^17^ respectively. These results also show that both isoquinoline (**2a**) and quinoline (**2b**) can participate in these TCRs. We did not extensively investigate all combinations of the three reactive components. In some instances, both **2a** and **2b** reacted in qualitatively similar fashion to provide the analogous type of adduct; we believe that the reaction pathways we have identified would be generally applicable to many sets of three reaction partners.

After exploring carbonyls as third components, we proceeded to identify other effective classes of 
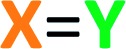
 electrophiles. Phenyl and cyclohexyl isocyanate (
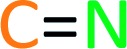
 electrophiles **3f** and **3g** respectively, Chart 1c) participated in the coupling process to furnish the quinazolinone derivatives **18**, **19** and **20** (Fig. 3b). The last involved the incorporation of a pyridine derivative, specifically methyl nicotinate (**2g**). When pyridine (**2h**) itself was used along with phenyl isocyanate (**3f**), the ^1^H NMR spectrum of the crude reaction product was fairly clean, suggesting an efficient initial transformation. However, the product proved difficult to isolate, perhaps a reflection of the lability of the 1,2-dihydropyridine that was initially formed. In contrast, the vinylogous carbamate character in **20** rendered that adduct readily tractable.

The final class of mode B TCRs involved trapping the initial 1,3-zwitterion with various electron-deficient alkenes or alkynes (Fig. 3c). Classic Diels–Alder dienophiles meet that definition. Dimethyl acetylenedicarboxylate (**3h**) as well as the *N*-substituted maleimides **3i** and **3j** all performed well, giving rise to adducts containing the new six-membered tetrahydropyridine ring present in each of **21–25** (Fig. 3c). In the case of formation of **24** (52%) from phenanthridine (**2c**) and *N*-methylmaleimide (**3i**), the 1 : 1 adduct **9** (*cf.*Fig. 2a) was formed competitively (34%). This suggests a shorter lifetime for the initially formed 1,3-zwitterion. This is expected because its cyclization to the azetidine **9** is not accompanied by as large of a loss in aromatic resonance stabilization as is the case for, say, quinoline or isoquinoline. Finally, when pyridine was the *N*-HAR component, **25** was produced efficiently (accompanied by a small amount of the *cis-anti* diastereomer, see ESI†). This product was also accompanied by one diastereomer of the Diels–Alder adduct **26**, again reflecting a lability of the 1,2-dihydropyridine moiety.

### Mode C (TCR with a monoprotic nucleophile)

**Fig. 4:**
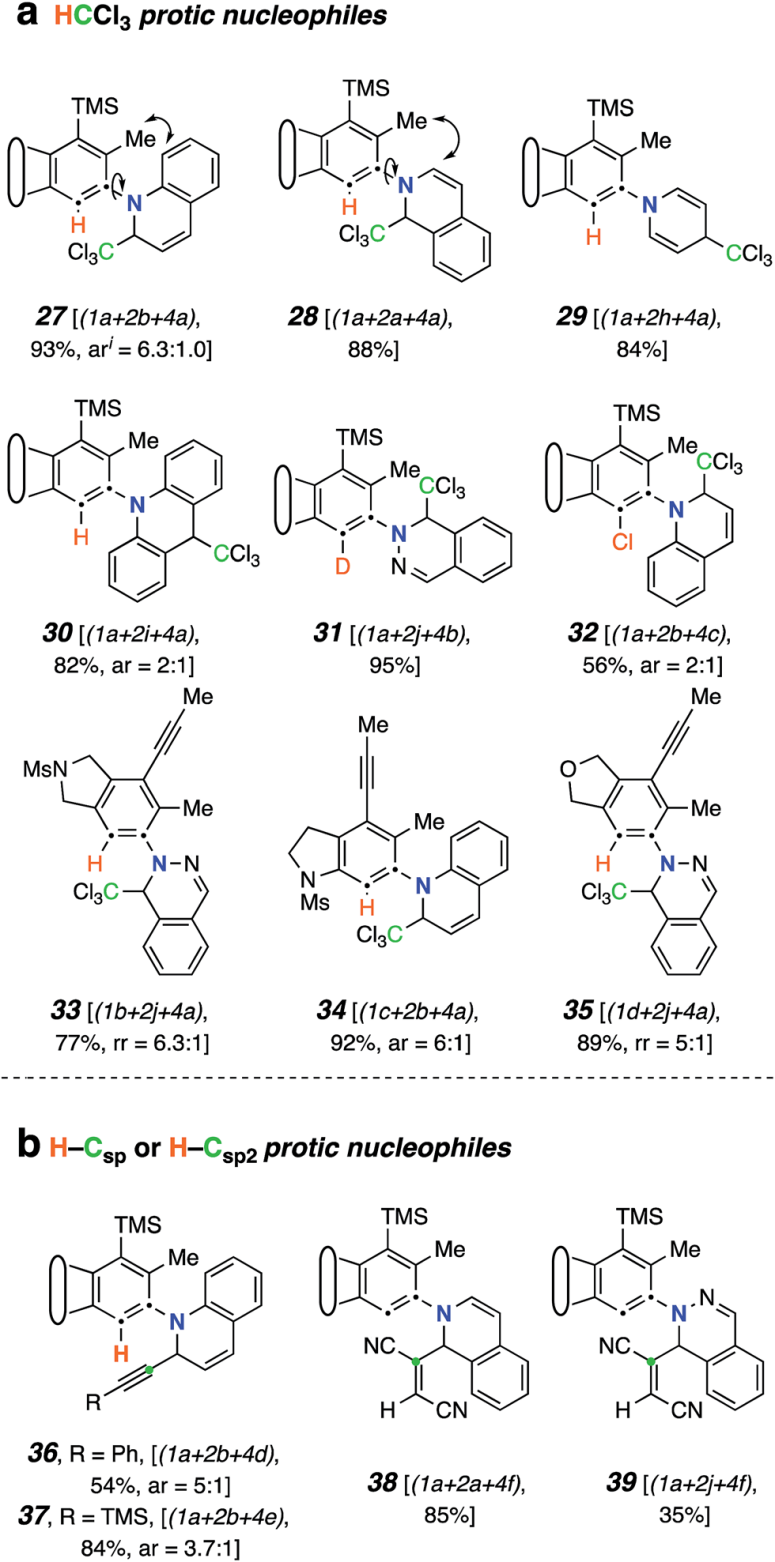
Products from mode C three-component reactions using monoprotic nucleophiles. Trapping of zwitterions (*cf.***III**) by (a) CCl_3_-containing electrophiles and (b) carbon-acids (terminal alkynes or fumaronitrile). **I** ar = atropisomeric ratio (observed by ^1^H NMR spectroscopy, see ESI†).

We next studied the outcome of heterocyclic trapping of the benzynes in the presence of various protic nucleophiles (mode C, Fig. 1). We began by heating the triyne substrate **1a** with quinoline (**2b**) in chloroform (**4a**, 0.02 M). The zwitterionic intermediate abstracted a proton from CHCl_3_, and the resulting ion pair collapsed to give the trichloromethylated adduct **27** (Fig. 4a). This molecule, as well as the other quinoline-derived biaryls, gave evidence of two atropisomers in its ^1^H and ^13^C NMR spectra. The analogous reaction with isoquinoline cleanly produced **28**; the NMR spectra for this isomeric compound showed a single set, albeit of broadened, resonances, reflecting the expected lower barrier for biaryl rotation compared to that in **27**.

Because of the relatively high efficiency of the trapping by CHCl_3_, we examined a wider array of *N*-HARs as well as other benzyne precursors for this class of TCR (Fig. 4a). In the case where pyridine (**2h**) was used as the *N*-HAR, the 4-substituted product **29** was formed. Use of acridine (**2i**) also led to the formation of its *para*-functionalized product, **30**, because the adduct arising from trapping by Cl_3_C^–^ at an *ortho*-carbon would significantly interrupt aromaticity. The remote nature of the Cl_3_C-bearing carbon in **30** results in a only subtly different pair of atropisomers, a fact revealed in the NMR data for this adduct (see ESI†). The fact that chloroform was the source of the proton in these reactions was validated when CDCl_3_ was used as the third component; **31** was formed in 95% yield. This result also demonstrated that phthalazine (**2j**) was a participant in this TCR. We then examined several other aryne precursors for this transformation. Each of **33**, **34**, and **35** was produced in a very good yield, starting with **1b**, **1c**, and **1d**, respectively. Finally, when carbon tetrachloride (**4c**) was used as solvent instead of CHCl_3_, chlorination of the 1,3-zwitterion from quinoline led to the formation of **32**.^18^

TCRs using carbon acids were also shown to be effective. Specifically, we found that terminal alkynes (**4d** and **4e**) were shown to have a sufficiently acidic proton to engage in a TCR to provide the alkynyl derivatives **36** and **37** (Fig. 4b). Surprisingly, fumaronitrile (**4f**), which we initially expected to capture the zwitterion in a net [4 + 2] manner (*i.e.*, *via* mode B), had a different outcome. Protonation and capture by the conjugate base of **4f** gave product **38** or **39** when isoquinoline (**2a**) or phthalazine (**2j**) was used as the *N*-HAR.

### Mode C (TCR with a diprotic nucleophile)

**Fig. 5:**
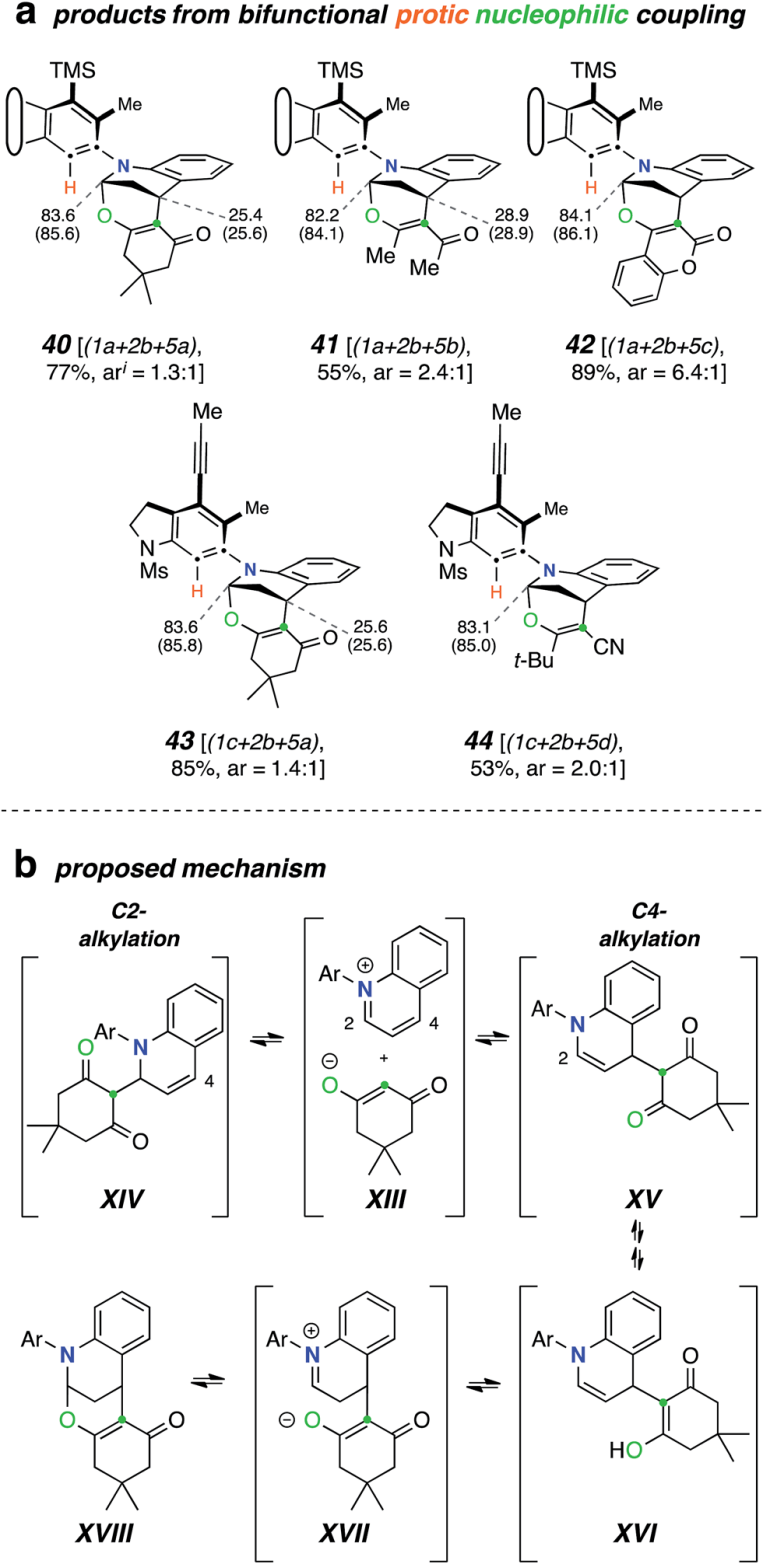
Products from mode C three-component reactions using diprotic nucleophiles. (a) Product structures [ratios of major and minor (separable) atropisomers]. (b) Mechanistic rationale consistent with the formation of the hemiaminal-containing skeletons in products **40–44**.

We then studied the behavior of several β-dicarbonyl protic nucleophiles (**5a–d**, Chart 1d) and encountered a new type of net bicyclization reaction (Fig. 5a). For example, heating the substrate **1a** with quinoline (**2b**) and dimedone (**5a**) resulted in the formation of the bridged benzoxazine **40** (77%) by sequential engagement of benzyne **VI** by **2b** and, then, **5a**. Thus, the third component here was serving as a “doubly protic” nucleophile. In this case the more crowded atropisomers interconverted sufficiently slowly that they now could be chromatographically separated. As the additional examples in Fig. 5a demonstrate, acetylacetone (**5b**), 4-hydroxycoumarin (**5c**), and α-cyanopinacolone (**5d**) all participated in this type of transformation. The structures of these novel products (**40–44**) were established on the basis of (i) the contiguous four-spin vicinal coupling array among the bridging methylene and bridgehead methine protons and, especially, (ii) the ^13^C NMR chemical shifts of the bridgehead aminal carbon (82–86 ppm). These spectroscopic signatures were consistently seen in all five of these adducts. HSQC and HMBC experiments were also consistent with these assignments.

A mechanistic rationale for this interesting transformation is suggested in Fig. 5b. Nucleophilic addition of quinoline (**2b**) to the HDDA-benzyne and proton transfer from, for example, dimedone (**5a**) produces the enolate-quinolinium ion pair **XIII**. This can undergo collapse by attack at either C2 (to **XIV**) or C4 (to **XV**). Since C2-attack is the main event in the earlier TCRs, we suggest that **XIII–XV** are sufficiently close in energy to be in dynamic equilibrium with one another. Keto–enol tautomerization, while possible for either **XIV** or **XV**, can proceed further from the latter because of its embedded enamine moiety. Specifically, internal protonation within enol **XVI** gives zwitterion **XVII**, the collapse of which would generate **XVIII** (*cf.***40**/**43**).

**Fig. 6:**
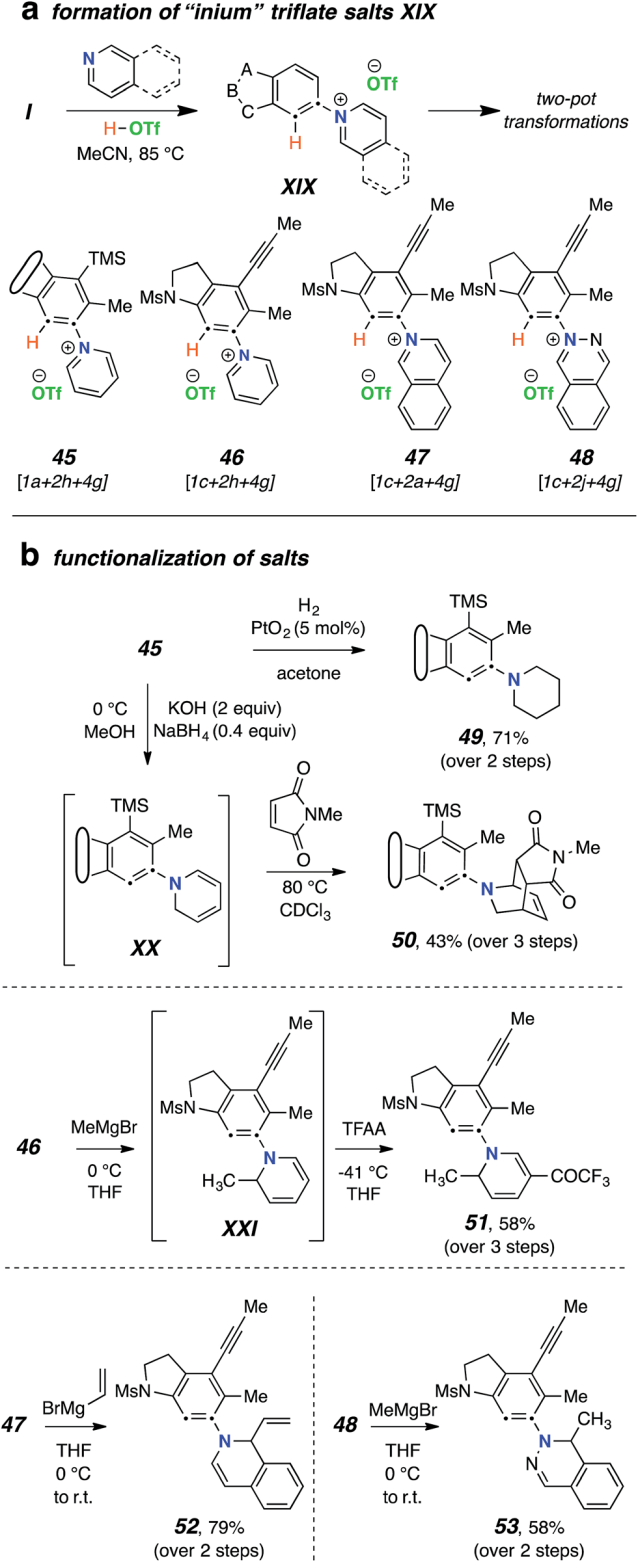
Use of the “non-nucleophile” triflic acid (**4g**) allows for the incorporation of a broader range of nucleophiles. (a) TCRs were performed using 3 equiv of the *N*-HAR and 2 equiv of triflic acid in acetonitrile (MeCN), which led to the formation of salts **45–48**. (b) Examples demonstrating the subsequent diversification of some of these salts to give products **49–53**.

Finally, we devised a two-pot variant that allows for the use of a wider variety of nucleophiles in these types of reactions. Namely, generation of the benzyne from the polyyne precursor in the presence of the *N*-HAR and “non-nucleophilic” triflic acid (**4g**), typically in a molar ratio of 1 : 3 : 2 to ensure the presence of the free-base form of the *N*-HAR, gave rise to isolable salts **XIX** (Fig. 6a). Specifically, the *N*-arylated “inium” triflate salts **45–48** were efficiently generated. These reactions were seen to be most efficient using acetonitrile as the reaction solvent. Crude samples of the salts could be isolated by solvent removal and were characterized by ^1^H NMR and HRMS analyses. These samples still retained the excess *N*-HAR H^+^TfO^–^ salts, but that did not seriously hamper their subsequent reactions with nucleophiles. These inium ions could then be redissolved (or suspended) in various solvents and further transformed to products that were either complementary to or not compatible with the one-pot TCRs. For example (Fig. 6b), the *N*-aryl pyridinium triflate **45** cleanly underwent hydrogenation in the presence of Adams catalyst, producing the piperidine derivative **49** in 71% yield at room temperature. In another reduction **46** was converted to the 1,2-dihydropyridine **XX** using sodium borohydride. As was the case with 1,2-dihydropyridines mentioned earlier, **XX** was also found to be unstable upon attempted silica gel chromatographic purification. However, the crude product could be further treated with *N*–methyl maleimide to furnish the Diels–Alder adduct **50** in 43% overall yield (from **1a**). The triflate salts were also found to react readily with (excess) Grignard reagents. For example, the **1c**-derived pyridinium salt **46** was reacted with MeMgBr in THF to produce **XXI**. This dihydropyridine was transformed directly into **51** using trifluoroacetic anhydride (58% overall yield from **1c**). Grignard reagents were used to convert other heterocyclic derived salts, namely **47** and **48**, into their corresponding alkylated products **52** and **53** in a clean manner.

## Conclusion

We have demonstrated that various modes (A–C) of reaction can be realized by reacting six-membered *N*-heteroaromatic compounds with benzynes generated by thermal HDDA cycloisomerization of triyne (or tetrayne) precursors. All reactions can be rationalized as passing through an initially formed 1,3-zwitterion arising from attack on the benzyne by the nitrogen atom of the heterocycle. In mode A and in unprecedented fashion, 1 : 1 adducts emanating from this zwitterion are produced. For the most part, these products belong to either of the benzoazetidine or benzoazocine family.

In mode B, the first of two types of three-component reaction, an intermediate electrophile is used to capture the 1,3-zwitterion. Mode B electrophiles can be suitably reactive carbonyl compounds, isocyanates, or electron-poor alkenes or alkynes. In mode C, the third component is a protic acid that quenches the anionic centre in the zwitterion to give an ion pair that collapses to product. Diprotic nucleophiles (*e.g.*, β-dicarbonyl compounds) can be used to give novel bridged polycyclic products. Finally, the triflic acid produces isolable *N*-arylinium triflates that subsequently can be subjected to nucleophilic addition to further widen the strategic scope of the process.

## Conflicts of interest

There are no conflicts to declare.

## Supplementary Material

Supplementary information
