# Supplementary material for: Reactions of thermally generated benzynes with six-membered N-heteroaromatics: pathway and product diversity
Source: Chem Sci. 2019 Aug 14;10(39):9069–76. doi: 10.1039/c9sc03479j (PMC6889833; doi:10.1039/c9sc03479j)
Supplement: Supplementary file 1 [file SC-010-C9SC03479J-s001.pdf]

Electronic Supplementary Material (ESI) for Chemical Science.
This journal is © The Royal Society of Chemistry 2019

Electronic Supplementary Information for

Reactions of thermally generated benzynes with six-membered *N*–heteroaromatics: Pathway and product diversity

Sahil Arora, Juntian Zhang, Vedamayee Pogula, and Thomas R. Hoye*

Department of Chemistry, University of Minnesota, 207 Pleasant St. SE, Minneapolis, MN 55455

E-mail: hoye@umn.edu

Table of Contents

I. General Experimental Protocols S3

II. Preparation procedures and characterization data for all compounds

(a) Poly-yne substrates and their precursors S5–S10

(b) Products obtained from mode **a**: 1:1 adducts of *N*–heterocycles and arynes S11–S17

(c) Products obtained from mode **b**: electrophilic three-component reactions S18–S39

(d) Products obtained from mode **c**: nucleophilic three-component reactions S40–S65

(e) Products obtained from triflate salt formations and their functionalizations S66–S76

(f) Product obtained from carbene trapping with *p*-bromobenzaldehyde (endnote #17) S77

III. Discussion of Computational Results S78–S109

IV. References for the Supplementary Information S110

V. Copies of NMR spectra S110

S-1 .... S111-S112

1b S113-S114

S-3 .... S115-S116

S-4 .... S117-S118

S-5 .... S119-S120

1a S121-S122

6 .... S123-S126

7 .... S127-S136
8 .... S137-S142
9 .... S143-S148
10 .... S149-S157
11 .... S158-S167
12 .... S168-S174
13-A S175-S176
13-B S177-S178
14 .... S179-S180
15-A S181-S182
15-B S183-S184
16-A S185-S186
16-B S187-S188
17-A S189-S192
17-B S193-S196
18 .... S197-S198
19 .... S199-S200
20-A and 20-B S201-S202
21 .... S202-S203
22-cis/anti S203-S204
22-cis/syn S205-S206
23-cis/anti S207-S210
23-cis/syn S211-S212
24-cis/anti S213-S215
24-cis/syn S216-S218
25-cis/anti S219-S220
25-cis/syn S221-S222
26 .... S223-S224
27 .... S225-S226
28 .... S227-S228
29 .... S229-S230

S-7 .... S231
30 .... S232-S233
31 .... S234-S235
32 .... S236-S237
33 .... S238-S239
34-A S240-S242
34-B S243-S245
35-A S246-S248
35-B S249-S251
36 .... S252-S253
37 .... S254-S255
38 .... S256-S257
39 .... S258-S259
40-A S260-S263
40-B S264-S267
41-A S268-S271
41-B S272-S275
42-A S276-S279
42-B S280-S281
43-A S282-S286
43-B S287-S291
44-A and 44-B S292-S296
45 and S-8 .... S297-S298
49 .... S299-S300
50 .... S301-S305
46 and S-8 .... S306-S307
51 .... S308-S309
47 .... S310
52 .... S311-S312
48 .... S313
53 .... S314-S315
v S316-S317

I. General Experimental Protocols

^{13}C and ^{1}H NMR spectra were taken on an HD-500 or AV-500 (500 MHz) spectrometer. ^{1}H chemical shifts solutions are referenced to TMS (δ 0.00 ppm) in $CDCl_3$ and to the residual CHD_5 (δ 7.15 ppm) in benzene-d_6. Where encountered, a non-first order multiplet in a ^{1}H NMR spectrum is denoted as 'nfom'. Resonances are reported in the following format: chemical shift (ppm) [multiplicity, coupling constant(s) (in Hz), integral (to the nearest integer), and assignment of the location within the structure]. This is indicated by, e.g., R^1C*Ha*Hb for diastereotopic geminal protons; arbitrarily, the more downfield resonance is labeled as H_a. Coupling constants have been analyzed using methods we have described elsewhere.[1,2] The ^{13}C NMR shifts are taken from the "1D" spectra.

Infrared spectra were recorded for samples as thin films deposited on a diamond window in the attenuated total reflectance (ATR) mode using a Bruker Alpha II Spectrometer. Absorption bands are given in cm^{-1}.

Some of the high-resolution **mass spectrometry** (HRMS) measurements were made on a Bruker BioTOF II (ESI-TOF) instrument in the electrospray ionization mode (ESI); poly(ethylene glycol) (PEG) or poly(propylene glycol) (PPG) was used as the standard/calibrant. Samples were infused as methanol solutions. HRMS data were collected as approximately ten separate data sets and then averaged to obtain the reported "found" value. The majority of the HRMS data were collected on a Thermo Orbitrap Velos instrument (having a mass accuracy of ≤3 ppm) in the ESI or atmospheric pressure chemical ionization (APCI) mode. An external calibrant (Pierce™ LTQ) was used; samples were introduced by way of a LC/MS run.

Medium pressure liquid **chromatography** (MPLC) was performed on columns of silica gel (25-200 psi, 20-40 μm, 60 Å pore size, Teledyne RediSep Rf Gold® normal-phase silica) that had been hand packed. The device consisted of a Waters HPLC pump (M6000), a Gilson (112 UV) detector, and a Waters (R401) differential refractive index detector. Agela silica gel (230-400 mesh) was used to prepare flash chromatography columns. Silica gel thin layer chromatography (TLC) was performed on glass- or plastic-backed plates that were visualized UV illumination and/or by a solution of potassium permanganate or ceric ammonium molybdate (CAM) and heating.

Some compounds were purified by HPLC to achieve mg quantities of samples of high purity, from which the full characterization data set. A 1 cm diameter x 25 cm long column of silica gel (Alltech, Econosil, 10 μm) was used.

Reactions performed under anhydrous conditions were carried out in oven-dried glassware under an atmosphere of nitrogen. Anhydrous THF was dried by passage through a column of activated alumina before use. Reaction temperature refers to the temperature of the external heating or cooling bath unless otherwise noted. HDDA reactions, including ones performed at temperatures higher than the boiling point of the reaction solvent, were done in a screw-capped vial or culture tube that was capped with an inert, Teflon®-lined closure.

II. Preparation procedures and characterization data for all compounds

(a) Poly-yne substrates and their precursors

N,N-Di(prop-2-yn-1-yl)methanesulfonamide (S-1)

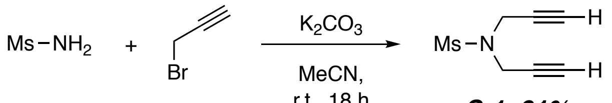

Methanesulfonamide (10.0 g, 105.1 mmol) and potassium carbonate (32.0 g, 231.2 mmol, 2.2 equiv) were placed in a 1 L round-bottom flask equipped with a stir bar. Acetonitrile (263 mL, 0.4 M) was added and the contents were protected from light by wrapping the flask in aluminum foil. Propargyl bromide (80 % w/w in toluene, 24.9 mL, 131.4 mmol, 2.2 equiv) was added, and the resulting suspension was allowed to stir overnight. The mixture was filtered through Celite®, and the filtrate was concentrated under reduced pressure. The crude product was purified by flash chromatography (2:1 hexane: EtOAc) to give **S-1** (16.4 g, 90.9 mmol, 91%) as a pale yellow solid.

^{1}H NMR (500 MHz, $CDCl_3$): δ 4.20 (d, 2.5 Hz, 4H NCH_2), 2.99 (s, 3H, NSO_2CH_3), and 2.40 (t, 2.5 Hz, 2H, C≡C*H*).

^{13}C NMR (125 MHz, $CDCl_3$): 76.6, 74.6, 38.6, and 36.5.

IR (neat): 3284, 2120, 1434, 1343, 1327, 1152, 1081, 951, 891, and 784 cm^{-1}.

HRMS (ESI-TOF): Calcd for $C_7H_9NNaO_2S^+$ [M+Na^+] requires 194.0246; found 194.0249.

mp: 54.5–56.5 °C.

N,N-Di(hexa-2,4-diyn-1-yl) methanesulfonamide (1b)

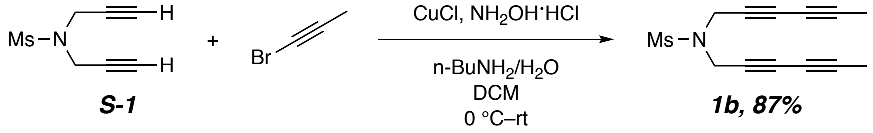

Copper (I) chloride (199 mg, 2.01 mmol) and hydroxylamine hydrochloride (698 mg, 10 mmol) were added to a 250 mL 3-neck round-bottom flask equipped with a magnetic stir bar and two addition funnels. The reaction vessel was placed under a nitrogen atmosphere and 40/60 (v/v) H_2O/*n*-$BuNH_2$ was added, and the mixture was cooled to 0 °C. *N,N*-Dipropargyl methanesulfonamide (**S-1**, 3.44 g, 20.1 mmol) in DCM (100 mL) was placed into one addition funnel and bromopropyne in hexane (29.9%, 48.9 mL, 80.4 mmol) into the other. Approximately 10% of the volume of the solution of diyne (~10 mL) was added dropwise, at which time bromopropyne addition was begun. The two solutions were then simultaneously added at approximately the same rate until addition of both reactants was complete. The mixture was allowed to warm to room temperature. After 2 hours, the reaction was judged to be complete by TLC. Note: allowing this coupling reaction to proceed too long makes it susceptible to the possibility of a subsequent (and undesired) pentadehydro-Diels-Alder reaction.[3] The mixture was quenched by the addition of saturated aqueous NH_4Cl (100 mL) and extracted with DCM (100 mL). The combined organic layers were washed with brine (50 mL, 1x), dried with $MgSO_4$, and concentrated to give crude **(1b)** as a pale yellow solid. The crude product was purified by column chromatography (3:1 Hex:EtOAc to 2:1 Hex:EtOAc) to yield **1b** as a white crystalline solid (4.1 g, 16.6 mmol, 87%). The data for this compound matched those previously described.[4]

^{1}H-NMR (500 MHz, $CDCl_3$): 4.21 [s, 4H, MsN(CH_2)$_2$], 2.97 (s, 3H, NSO_2CH_3), and 1.94 (s, 6H, C≡CH_3).

^{13}C-NMR (125 MHz, $CDCl_3$): 77.2, 71.5, 67.7, 63.5, 38.7, 37.5, and 4.3.

IR (neat): 2260, 1430, 1345, 1329, 1154, 1073, 965, 948, 893, and 781 cm^{-1}.

HRMS (ESI-TOF): Calcd for $C_{13}H_{13}NNaO_2S^+$ [M+Na$^+$] requires 270.0559; found 270.0553.

mp: 99-101 °C (lit.[4] mp = 99-101 °C)

1-(2-Ethynyl-4,5-dimethoxyphenyl)-3-(trimethylsilyl)prop-2-yn-1-ol (S-3)

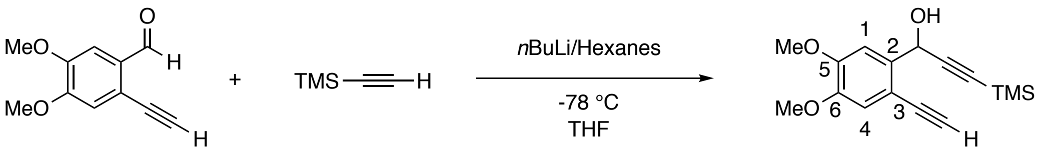

S-2 ***S-3, 82%***

TMS-acetylene (4.49 mL, 31.6 mmol, 1.25 equiv) was added to a 250 mL round-bottom flask, dissolved in THF (70 mL), and cooled to 0 °C. To this solution *n*-BuLi (2.5 M in hexanes, 12.1 mL, 1.2 equiv) was added dropwise and the solution was stirred at 0 °C for 40 min, at which time the solution was cooled to -78 °C. Aldehyde **S-2**[5] (4.8 g, 25.4 mmol) was added to a 250 mL round bottom flask equipped with an internal thermometer, dissolved in THF (50 mL), placed under N_2, and cooled to -78 °C (internal temperature). To this solution, the -78 °C solution of the lithium acetylide was added via cannula dropwise so that the internal temperature of the receiving solution did not rise above -70 °C. After the addition was complete (~30 min), the solution was stirred for an additional 1 h at which time TLC (3:1 Hex/EtOAc) of an aliquot indicated complete consumption of the starting aldehyde. The reaction mixture was quenched by the addition of a mixture of HOAc (2.5 mL) and THF (2.5 mL) at 0 ºC. The mixture was then warmed to room temperature and concentrated. The resulting residue was diluted with Et_2O (100 mL) and washed successively with sat. NH_4Cl (50 mL), $NaHCO_3$ (70 mL), water (100 mL), and brine (50 mL). The organic layer was dried ($MgSO_4$), filtered, and concentrated to give a sticky crude product. This crude product was purified by column chromatography (3:1 Hex:EtOAc) to give the desired alcohol **S-3** (5.9 g, 82.4 %) as a thick tan oil that turned into an amorphous solid, after being kept in the freezer (-10 °C).

^{1}H NMR (500 MHz, $CDCl_3$): δ 7.27 (s, 1H, Ar*H*1), 6.97 (s, 1H, Ar*H*4), 5.85 (s, 1H, ArC*H*OH), 3.93 (s, 3H, C*H*$_3$OC6), 3.88 (s, 3H, C*H*$_3$OC5), 3.29 (s, 1H, C≡C*H*), and 0.20 [s, 9H, Si(C*H*$_3$)$_3$].

^{13}C NMR (125 MHz, $CDCl_3$): δ 150.0, 148.6, 136.4, 115.0, 112.6, 110.0, 104.6, 91.6, 81.2, 81.1, 63.1, 56.1, 55.9, and -0.1.

IR (neat): 3281, 3001, 2959, 2907, 2850, 2172, 2102, 1604, 1510, 1251, 1210, 1095, 1036, and 846 cm^{-1}.

HRMS (ESI-TOF): Calcd for $C_{16}H_{20}NaO_3Si^+$ [M+Na$^+$] requires 311.1074; found 311.1072.

1-(4,5-Dimethoxy-2-(penta-1,3-diyn-1-yl)phenyl)-3-(trimethylsilyl)prop-2-yn-1-ol (S-4) and 1-(4,5-dimethoxy-2-(penta-1,3-diyn-1-yl)phenyl)hexa-2,4-diyn-1-ol (S-5)

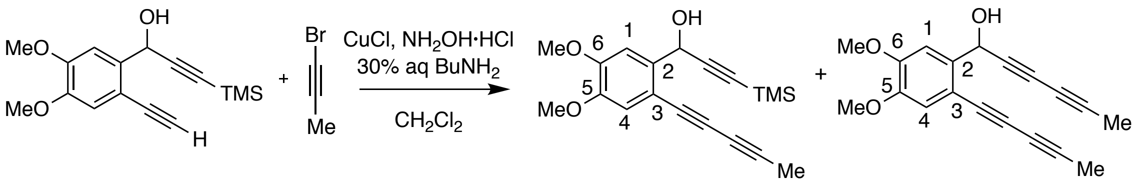

S-4, 62% ***S-5, 11%***

Copper (I) chloride (410 mg, 4.12 mmol) and hydroxylamine hydrochloride (714 mg, 21.6 mmol) were added to a 250 mL 3-neck round-bottom flask equipped with a magnetic stir bar and two addition funnels. The reaction vessel was placed under a nitrogen atmosphere, 70/30 (v/v) H_2O/*n*-$BuNH_2$ was added, and the mixture was cooled to 0 °C. 1-(2-Ethynyl-4,5-dimethoxyphenyl)-3-(trimethylsilyl)prop-2-yn-1-ol (5.82 g, 20.6 mmol) in DCM (60 mL) was placed into one addition funnel and bromopropyne in hexane (24.9%, 13.8 g, 28.8 mmol) into the other. Approximately 10% of the volume of the solution of diyne (~6 mL) was added dropwise, at which time bromopropyne addition was begun. The two solutions were then simultaneously added at approximately the same rate until addition of both reactants was complete. The mixture was allowed to warm to room temperature. After 1 hour, the reaction was judged to be complete by TLC. The mixture was quenched by the addition of saturated aqueous NH_4Cl (50 mL) and extracted with DCM (50 mL). The combined organic layers were washed with brine (50 mL, 1x), dried with $MgSO_4$, and concentrated to give crude product as a pale yellow solid. The crude product was purified by column chromatography (3:1 Hex:EtOAc) to yield, in order of elution, **S-4** as an orange-yellow oil (4.2 g, 0.013 mmol, 62%), which solidified upon storage at -10 °C to give an amorphous yellow powder and **S-5** as a pale yellow amorphous solid (642 mg, 11%).

Data for S-4 (Faster eluting product):

^{1}H NMR (500 MHz, $CDCl_3$): δ 7.15 (s, 1H, Ar*H1*), 6.94 (s, 1H, Ar*H4*), 5.87 (d, *J* = 3.3 Hz, 1H, ArC*H*OH), 3.92 (s, 3H, C*H*$_3$OC6), 3.87 (s, 3H, C*H*$_3$OC5), 2.41 (s, 1H, O*H*), 2.03 (s, 3H, *H*$_3$C–C≡), and 0.20 [s, 9H, Si(C*H*$_3$)$_3$].

^{13}C NMR (125 MHz, $CDCl_3$)**:** δ 150.2, 148.7, 137.3, 115.4, 112.7, 110.1, 104.6, 91.8, 81.6, 78.3, 71.5, 64.4, 63.3, 56.2, 56.0, 4.8, and -0.1.

IR (thin film): 3490, 2959, 2912, 2856, 2836, 2171, 1602, 1509, 1463, 1444, 1405, 1345, 1246, 1207, 1152, 1073, 1035, 1002, 966, 840, 758, 700, 641, 596, and 479 cm^{-1}.

HRMS (ESI-TOF): Calculated for $C_{19}H_{21}O_2Si^+$ [M+H$^+$–H_2O] 309.1305, found 309.1302. (most intense ion); Calculated for $C_{19}H_{23}O_3Si^+$ [M+H$^+$] 327.1411, found 327.1374. (minor ion)

Data for S-5 (Slower eluting product):

^{1}H NMR (500 MHz, $CDCl_3$): δ 7.15 (s, 1H, Ar*H1*), 6.95 (s, 1H, Ar*H4*), 5.87 (d, *J* = 4.9 Hz, 1H, ArC*H*OH), 3.93 (s, 3H, CH_3OC6), 3.86 (s, 3H, CH_3OC5), 2.41 (br s, 1H O*H*), 2.04 (s, 3H, H_3C–C≡), and 1.95 (s, 3H, H_3C–C≡).

^{13}C NMR (125 MHz, $CDCl_3$)**:** δ 150.3, 148.7, 136.7, 115.2, 112.3, 109.6, 81.6, 78.22 (2x), 73.8, 71.5, 71.2, 64.2, 63.7, 63.0, 56.065, 56.060, 4.7, and 4.4.

IR (thin film): 3438, 3003, 2961, 2937, 2913, 2836, 2255, 1601, 1509, 1463, 1404, 1373, 1345, 1246, 1206, 1149, 1071, 992, 939, 862, 757, 735, 595, 535, 470, and 419 cm^{-1}.

HRMS (ESI-TOF): Calculated for $C_{19}H_{15}O_2^+$ [M+H^+–H_2O] 275.1067, found 275.1064 (most intense ion); Calculated for $C_{19}H_{17}O_3^+$ [M+H^+] 293.1172, found 293.1183 (minor ion).

1-(4,5-Dimethoxy-2-(penta-1,3-diyn-1-yl)phenyl)-3-(trimethylsilyl)prop-2-yn-1-one (1a)

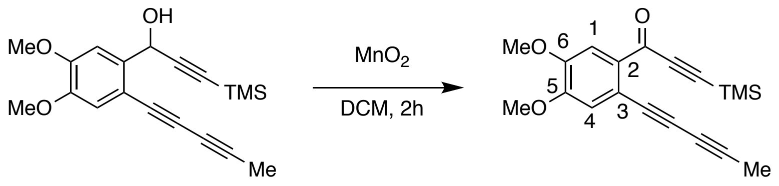

S-4 ***1a, 81%***

MnO_2 (4.98 g, 55.4 mmol) was added to a solution of triyne **S-4** (3.61 g, 11.1 mmol) in CH_2Cl_2 (24 mL), and the resulting suspension was stirred at room temperature overnight. The reaction mixture was filtered through Celite®, the filtrate was concentrated in vacuo, and the residue was purified by flash chromatography (hexanes:EtOAc = 9:1) to afford the ketotriyne **1a** as a yellow crystalline powder (2.91 g, 81%).

^{1}H NMR (500 MHz, $CDCl_3$): δ 7.59 (s, 1H, Ar*H1*, nOe CH_3OC6), 7.03 (s, 1H, Ar*H4*, nOe CH_3OC5), 3.94 (s, 3H, CH_3OC6), 3.93 (s, 3H, CH_3OC5), 2.04 (s, 3H, H_3C–C≡), and 0.31 [s, 9H, $Si(CH_3)_3$].

A difference nOe experiment showed enhancement of the C6-methoxy and C5-methoxy protons upon irradiation of ArH1 and ArH4, respectively, allowing the assignment of the proton chemical shifts given above for **1a.**

^{13}C NMR (125 MHz, $CDCl_3$): δ 175.1, 152.4, 149.1, 132.6, 117.1, 116.3, 113.4, 101.8, 101.3, 82.4, 80.0, 72.4, 65.0, 56.3, 56.0, 4.76, and -0.66.

IR (neat): 3007, 2961, 2913, 2849, 2238, 2154, 1686, 1659, 1642, 1587, 1556, 1463, 1441, 1397, 1353, 1255, 1202, 1170, 1096, 1029, 967, 846, and 760 cm^{-1}.

HRMS (ESI-TOF): Calculated for $C_{19}H_{20}NaO_3Si^+$ [M+Na$^+$] 347.1074, found 347.1080.

mp: 130-132 °C

TLC: R_f 0.4 (9:1 Hex/EtOAc).

(b) Products obtained from mode a: 1:1 adducts of *N*–heterocycles and arynes

12,13-Dimethoxy-8-methyl-9-(trimethylsilyl)fluoreno[4',3':3,4]azeto[2,1-*a*]isoquinolin-10(14d*H*)-one (6) and

2-(6,7-Dimethoxy-2-methyl-9-oxo-1-(trimethylsilyl)-9*H*-fluoren-3-yl)isoquinolin-1(2*H*)-one (7)

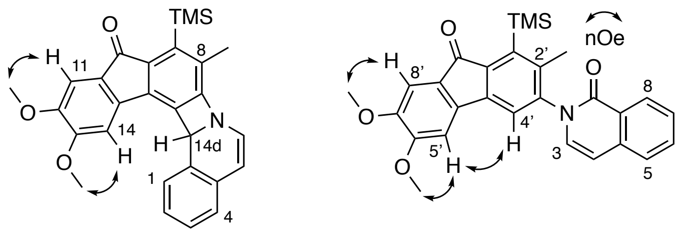

6 (5%) ***7*** (22%)

A solution of ketone **1a** (50.0 mg, 0.154 mmol) and isoquinoline (**2a**, 36.2 μL, 0.308 mmol) in benzene (10 mL) was heated in an 85 °C bath in a screw-capped culture tube. After 16 h the reaction mixture was concentrated and the residue was purified by MPLC (3:1 hexanes:EtOAc) to give the azetidine **6** (3.6 mg, 5%) and isoquinolone **7** (15.5 mg, 22%), each as an orange, foamy solid.

Compound 6 (azetidine)

^{1}H NMR (500 MHz, $CDCl_3$): δ 7.33–7.31 (nfom, 1H, Ar*H1*), 7.19 (s, 1H, Ar*H11*), 7.17–7.14 (m, 2H, Ar*H2* and Ar*H3*), 7.14 (s, 1H, Ar*H14*), 6.99–6.96 (nfom, 1H, Ar*H4*), 6.68 (s, 1H, *H14d*), 6.59 (d, *J* = 7.2 Hz, 1H, Ar*H6*), 5.73(d, *J* = 7.2 Hz, 1H, Ar*H5*), 4.06 (s, 3H, C13OCH_3), 3.93 (s, 3H, C12OCH_3), 2.27 (s, 3H, ArCH_3), and 0.40 [s, 9H, Si(CH_3)$_3$].

^{13}C NMR (125 MHz, $CDCl_3$): δ 192.7, 161.3, 153.4, 149.9, 145.1, 135.7, 135.5, 131.8, 131.6, 129.4, 128.6, 128.3, 127.6, 126.4, 126.0, 125.6, 125.1, 122.3, 110.5, 106.9, 104.6, 69.2, 56.4, 56.2, 17.5, and 2.7. (Some ^{13}C chemical shift values were obtained from the HSQC and HMBC data)

IR (neat): 2942, 2902, 2844, 1701, 1644, 1592, 1494, 1456, 1417, 1366, 1313, 1270, 1243, 1214, 1122, 1083, 1059, 1018, and 909 cm^{-1}.

HRMS (ESI-TOF): Calculated for $C_{28}H_{28}NO_3Si^+$ [M+H$^+$] 454.1833, found 454.1832.

TLC: R_f 0.2 (3:1 hexanes:EtOAc).

Compound 7 (isoquinolone)

^{1}H NMR (500 MHz, $CDCl_3$): δ 8.49 (dddd, *J* = 8.1, 1.2, 0.5, 0.5 Hz, 1H, Ar*H8*), 7.72 (ddd, *J* = 8.0, 7.1, 1.4 Hz, 1H, Ar*H7*), 7.60 (br d, *J* = 8.0 Hz, 1H, Ar*H5*), 7.56 (ddd, *J* = 8.2, 7.1, 1.2 Hz, 1H, Ar*H6*), 7.29 (d, *J* = 0.5 Hz, 1H, *H4'*), 7.16 (s, 1H, Ar*H8'*), 7.02 (d, *J* = 7.4 Hz, 1H, Ar*H3*), 6.90 (s,

1H, Ar*H5'*), 6.63 (dd, *J* = 7.5, 0.5 Hz, 1H, Ar*H4*), 3.94 (s, 3H, C6'OCH_3), 3.92 (s, 3H, C7'OCH_3), 2.20 (s, 3H, ArCH_3), and 0.45 [s, 9H, Si(CH_3)$_3$].

^{13}C NMR (125 MHz, $CDCl_3$): δ 193.8, 161.8, 154.6, 149.8, 144.1, 143.8, 143.4, 141.0, 140.8, 138.2, 137.2, 132.8, 131.6, 128.3, 127.4, 126.7, 126.5, 126.1, 119.7, 106.9, 106.7, 102.9, 56.3, 56.2, 19.2, and 2.8.

IR (neat): 3000, 2990, 2933, 2900, 2836, 1704, 1652, 1625, 1591, 1557, 1494, 1455, 1417, 1381, 1359, 1316, 1266, 1244, 1212, 1152, 1117, 1093, 1056, 1017, 1005, and 983 cm^{-1}.

HRMS (ESI-TOF): Calculated for $C_{28}H_{28}NO_4Si^+$ [M+H$^+$] 470.1782, found 470.1773.

TLC: R_f 0.1 (3:1 hexanes:EtOAc).

10,11-Dimethoxy-6-methyl-7-(trimethylsilyl)fluoreno[4',3':3,4]azeto[1,2-*a*]quinolin-8(12d*H*)-one (4e)

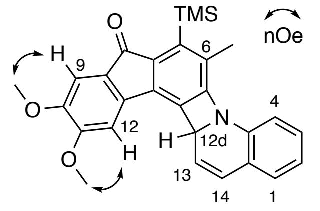

***8** (38%)*

A solution of ketone (**1a**, 50.0 mg, 0.154 mmol) and quinoline (**2b**, 36.2 μL, 0.308 mmol) in benzene (10 mL) was heated in an 85 °C bath in a screw-capped culture tube. After 16 h the reaction mixture was concentrated and the residue was purified by MPLC (3:1 hexanes:EtOAc) to give ketone **8** (27.2 mg, 38%) as an orange, foamy solid.

^{1}H NMR (500 MHz, $CDCl_3$): δ 7.27 (br d, *J* = 7.9 Hz, 1H, Ar*H4*), 7.18 (ddd, *J* = 7.9, 6.9, 2.2 Hz, 1H, Ar*H3*), 7.16 (s, 1H, Ar*H9*), 7.01 (ddd, *J* = 7.5, 7.5, 1.2 Hz, 1H, Ar*H2*), 6.99 (br dd, *J* = 7.5, 2.2 Hz, 1H, Ar*H1*), 6.71 (s, 1H, Ar*H12*), 6.34 (dd, *J* = 10.0, 2.2 Hz, 1H, *H13* or *H14*, 6.17 (dd, *J* = 2.3, 2.3 Hz, 1H, *H12d*), 6.07 (dd, *J* = 10.0, 2.2 Hz, 1H, *H13* or *H14*), 4.00 (s, 3H, C11OCH_3), 3.92 (s, 3H, C10OCH_3), 2.36 (s, 3H, ArCH_3), and 0.39 [s, 9H, Si(CH_3)$_3$].

^{13}C NMR (125 MHz, $CDCl_3$): δ 192.7 (C8), 162.4 (C5a), 153.7, 149.8, 145.8, 139.4, 135.7, 134.4, 133.2, 132.7, 129.0 (C3), 128.8, 128.0 (C1), 127.1, 126.1 (C13 or C14), 124.8 (C2), 123.7, 123.4 (C13 or C14), 123.3 (C4), 106.9, 104.2, 68.9 (C12d), 56.4, 56.2, 17.8, and 2.8.

IR (neat): 2945, 2904, 2841, 1703, 1638, 1590, 1496, 1456, 1419, 1363, 1323, 1273, 1241, 1214, 1119, 1080, 1059, 1018, and 989 cm^{-1}.

HRMS (ESI-TOF): Calculated for $C_{28}H_{28}NO_3Si^+$ [M+H$^+$] 454.1833, found 454.1830.

TLC: R_f 0.4 (2:1 hexanes:EtOAc).

10,11-Dimethoxy-6-methyl-7-(trimethylsilyl)fluoreno[4',3':3,4]azeto[1,2-f]phenanthridin-8(12dH)-one (9)

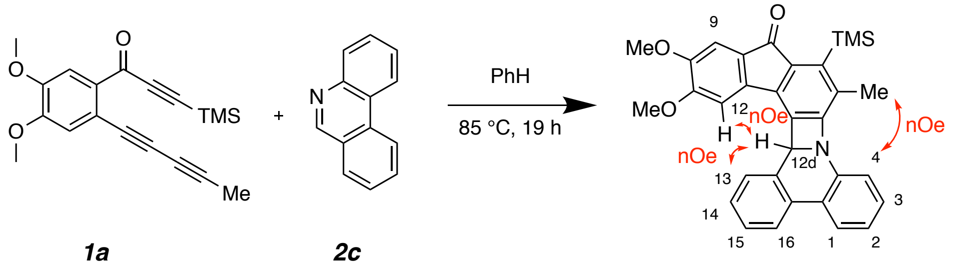

Triynone **1a** (25 mg, 0.077 mmol) and phenanthridine (**2c**, 41 mg, 0.231 mmol, 3 equiv) were added to a culture tube, dissolved in benzene (6 mL), and sealed with a Teflon-lined screw cap. The solution was heated overnight (18-19 h) in an oil bath at 85 °C, cooled, and passed through a plug of silica (EtOAc elution). The residue was purified by MPLC (1:1 Hex:EtOAc) to give **9** (34 mg, 87%) as a yellow oil.

Data for 9:

^{1}H NMR (400 MHz, $CDCl_3$): δ 7.82 (dd, J = 8.0, 1.5 Hz, 1H, Ar*H16*), 7.81 (dd, J = 8.0, 1.3 Hz, 1H, Ar*H1*), 7.53 (ddd, J = 7.6, 1.3, 1.3 Hz, 1H, Ar*H13*), 7.42 (dd, J = 7.9, 1.3 Hz, 1H, Ar*H4*), 7.34 (dddd, J = 8.0, 8.0, 1.5, 0.9 Hz, 1H, Ar*H15*), 7.30 (ddd, J = 7.9, 7.3, 1.5 Hz, 1H, Ar*H14*), 7.28 (dd, J = 7.5, 7.5, 1.3 Hz, 1H, Ar*H3*), 7.20 (s, 1H, Ar*H9*), 7.19 (s, 1H, Ar*H12*), 7.16 (ddd, J = 7.9, 7.3, 1.3 Hz, 1H, Ar*H2*), 6.50 (dd, J = 1.1, 0.8 Hz, 1H, Ar*H12d*), 4.07 (s, 3H, C11OCH_3), 3.94 (s, 3H, C10OCH_3), 2.36 (s, 3H, ArCH_3), and 0.38 [s, 9H, Si$(CH_3)_3$].

^{13}C NMR (126 MHz, $CDCl_3$): 192.4 (C8), 160.4 (C5a), 153.7 (C11), 150.0 (C10), 146.3 (C7), 138.7 (C4a), 135.5, 135.2 (C8a or C12a), 133.2 (C12e), 132.9, 131.0 (C16a), 129.7 (C8a or C12a), 129.6 (C12c), 129.1 (C14), 128.54 (C3), 128.47 (C15), 127.5 (C16b), 126.2 (C13), 124.6 (C2), 124.5 (C1), 124.3 (C16), 123.9 (C4), 121.2 (C6), 107.1 (C9), 104.7 (C12), 68.8 (C12d), 56.7 (O*Me*), 56.4 (O*Me*), 17.8 (Ar*Me*), and 3.0 (*TMS*). The assignments of carbon resonances were deduced using HSQC, HMBC, and differential nOe interactions (red arrows).

HRMS (APCI-Orbitrap): Calculated for $C_{32}H_{30}NO_3Si^+$ [M+H$^+$]: 504.1989, found 504.1935.

IR ($CDCl_3$): 3071, 3007, 2940, 2900, 2837, 2253, 1698, 1650, 1593, 1493, 1440, 1365, 1311, 1245, 1103, 1079, 1019, 906, 842, 726, 647, 602, 541, 450, and 420 cm^{-1}.

(1*Z*,3*Z*,5*Z*)-11,12-Dimethoxy-7-methyl-8-(trimethylsilyl)-9*H*-fluoreno[3,4-*f*][1,3,5]triazocin-9-one (4d)

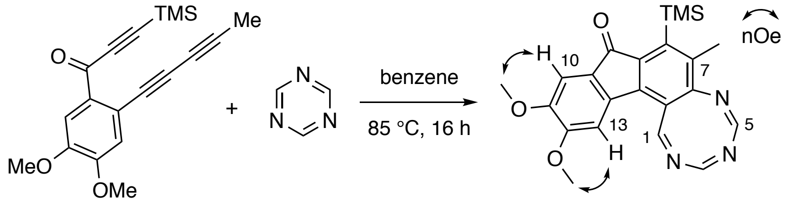

1a ***2d*** ***10** (33%)*

A solution of ketone **1a** (50.0 mg, 0.154 mmol) and 1,3,5-triazine (**2d**, 25.0 mg, 0.308 mmol) in benzene (10 mL) was heated in an 85 °C bath in a screw-capped culture tube. After 16 h the reaction mixture was concentrated and the residue was purified by MPLC (2:1 hexanes:EtOAc) to give ketone **10** (20.8 mg, 33%) as an orange solid.

^{1}H NMR (500 MHz, $CDCl_3$): δ 8.62 (d, J = 0.8 Hz, 1H, Ar*H1*), 8.04 (s, 1H, Ar*H5*), 7.97 (d, J = 0.8 Hz, 1H, Ar*H3*), 7.18 (s, 1H, Ar*H10*), 6.80 (s, 1H, Ar*H13*), 3.99 (s, 3H, C12OCH_3), 3.93 (s, 3H, C11OCH_3), 2.29 (s, 3H, ArCH_3), and 0.42 [s, 9H, Si(CH_3)$_3$].

^{13}C NMR (125 MHz, $CDCl_3$): δ 193.1 (C9), 163.8 (C1), 159.7 (C3), 156.0 (C5), 154.0, 150.0, 148.7, 145.4, 139.9, 137.2, 137.1, 136.6, 127.5, 121.8, 107.1 (C10), 105.8 (C13), 56.5, 56.2, 19.8 (ArMe), and 2.6 (TMS). (indicated carbon assignments from HSQC and/or HMBC)

IR (neat): 2999, 2942, 2897, 2836, 1702, 1633, 1588, 1565, 1531, 1492, 1457, 1420, 1361, 1312, 1294, 1240, 1212, 1149, 1081, 1048, 1017, and 949 cm^{-1}.

HRMS (ESI-TOF): Calculated for $C_{22}H_{24}N_3O_3Si^+$ [M+H$^+$] 406.1581, found 406.1579.

TLC: R_f 0.1 (2:1 hexanes:EtOAc).

(1*Z*,3*Z*,5*Z*)-11,12-Dimethoxy-7-methyl-8-(trimethylsilyl)-9*H*-fluoreno[3,4-*d*][1,3]diazocin-9-one (4c)

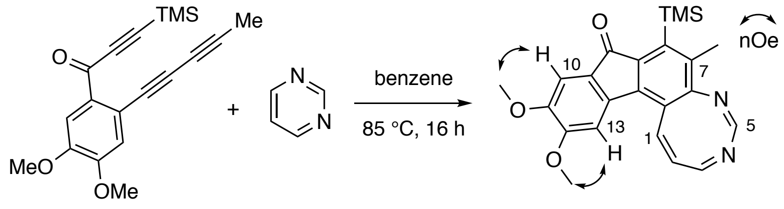

1a *2e* *11 (40%)*

A solution of ketone **1a** (30.0 mg, 0.0924 mmol) and pyrimidine (**2e**, 14.8 μL, 0.185 mmol) in benzene (8 mL) was heated in an 85 °C bath in a screw-capped culture tube. After 16 h the reaction mixture was concentrated and the residue was purified by MPLC (1:1 hexanes:EtOAc) to give ketone **11** (14.8 mg, 40%) as an orange solid.

^{1}H NMR (500 MHz, $CDCl_3$): δ 8.10 (dd, J = 0.9, 0.9 Hz, 1H, Ar*H5*), 7.86 (dd, J = 1.1, 1.1 Hz, 1H, Ar*H3*), 7.16 (s, 1H, Ar*H10*), 6.99 (d, J = 11.7 Hz, 1H, Ar*H1*), 6.95 (s, 1H, Ar*H13*), 6.50 (ddd, J = 11.7, 1.2, 0.9 Hz, 1H, Ar*H2*), 3.98 (s, 3H, C12OCH_3), 3.91 (s, 3H, C11OCH_3), 2.33 (s, 3H, ArCH_3), and 0.42 [s, 9H, Si(CH_3)$_3$].

^{13}C NMR (125 MHz, $CDCl_3$): δ 193.8, 164.0, 157.3, 153.5, 149.7, 149.4, 142.5, 140.6, 138.1, 136.9, 136.2, 134.6, 130.7, 127.6, 123.0, 106.9, 106.7, 56.3, 56.2, 20.2, and 2.7.

IR (neat): 2998, 2941, 2899, 2835, 1702, 1637, 1590, 1560, 1525, 1492, 1465, 1416, 1367, 1312, 1246, 1213, 1182, 1149, 1081, 1039, 1019, and 968 cm^{-1}.

HRMS (ESI-TOF): Calculated for $C_{23}H_{25}N_2O_3Si^+$ [M+H$^+$] 405.1629, found 405.1619.

TLC: R_f 0.1 (1:1 hexanes:EtOAc).

12,13-Dimethoxy-8-methyl-9-(trimethylsilyl)-5,6,7,15-tetrahydro-10*H*-6,15-epoxybenzo[*d*]fluoreno[4,3-*g*][1,3]diazocin-10-one (4h)

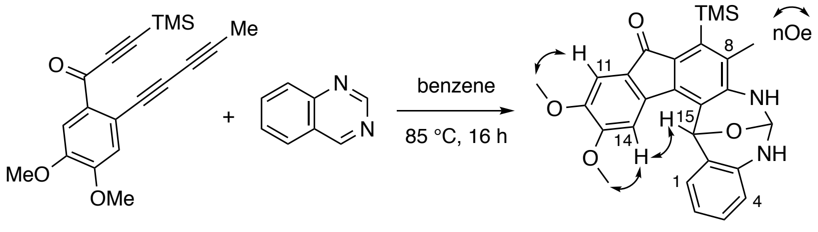

1a ***2f*** ***12*** *(57%)*

A solution of ketone **1a** (30.0 mg, 0.0924 mmol) and quinazoline (**2f**, 24.0 mg, 0.185 mmol) in benzene (8 mL) was heated in an 85 °C bath in a screw-capped culture tube. After 16 h the reaction mixture was concentrated and the residue was purified by MPLC (2:1 hexanes:EtOAc) to give ketone **12** (24.7 mg, 57%) as an orange, foamy solid.

^{1}H NMR (500 MHz, $CDCl_3$): δ 7.45 (s, 1H, Ar*H14*), 7.32 (dd, *J* = 7.8, 1.0 Hz, 1H, Ar*H1*), 7.19 (s, 1H, Ar*H11*), 7.14 (ddd, *J* = 7.8, 7.8, 1.4 Hz, 1H, Ar*H3*), 6.82 (ddd, *J* = 7.6, 7.6, 1.1 Hz, 1H, Ar*H2*), 6.80 (dd, *J* = 7.8, 1.0 Hz, 1H, Ar*H4*), 6.67 (s, 1H, Ar*H15*), 6.18 (d, *J* = 2.7 Hz, 1H, *H6*), 5.06 (d, *J* = 2.7 Hz, 1H, N*H*), 4.84 (br s, 1H, N*H*), 4.03 (s, 3H, C13OCH_3), 3.94 (s, 3H, C12OCH_3), 2.16 (s, 3H, ArCH_3), and 0.38 [s, 9H, Si(CH_3)$_3$].

^{13}C NMR (125 MHz, $CDCl_3$): δ 192.3 156.0, 152.9, 149.3, 142.0, 137.6, 137.0, 130.3, 129.4, 128.4, 125.2, 124.3, 124.1, 121.0, 119.0, 118.2, 106.9, 106.0, 102.9, 82.1 (C6), 66.8 (C15), 56.5, 56.2, 17.4 (ArMe), and 2.9 (TMS). (indicated carbon assignments from HSQC and/or HMBC)

IR (neat): 3433, 3336, 2993, 2944, 2905, 2835, 1681, 1607, 1579, 1547, 1496, 1461, 1419, 1381, 1362, 1341, 1296, 1247, 1209, 1179, 1095, 1049, 1036, 1018, and 989 cm^{-1}.

HRMS (ESI-TOF): Calculated for $C_{27}H_{29}N_2O_4Si^+$ [M+H$^+$] 473.1891, found 473.1882.

TLC: R_f 0.1 (2:1 hexanes:EtOAc).

(c) Products obtained from mode b: electrophilic three-component reactions

15-Acetyl-12,13-dimethoxy-8,15-dimethyl-9-(trimethylsilyl)-16aH-fluoreno[3',4':4,5][1,3]oxazino[3,2-a]quinolin-10(15H)-one (13-A and 13-B)

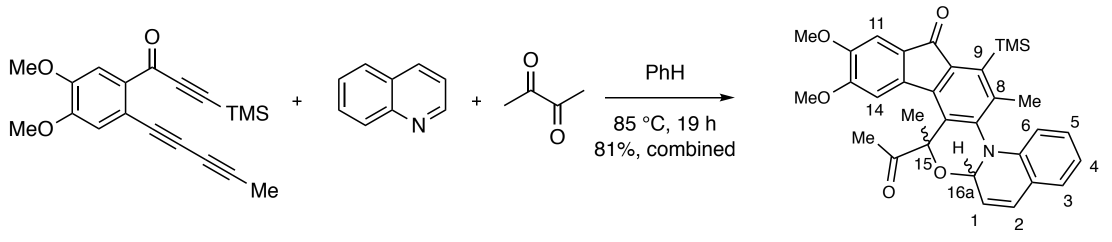

1a ***2b*** ***3a***

***13-A** (38%) and **13-B** (43%): separable pair of diastereomers, **13-A** is faster eluting*

Triynone **1a** (20 mg, 0.062 mmol), quinoline (**2b**, 15 μL, 0.124 mmol, 2 equiv) and diacetyl (**3a**, 27 mg, 0.310 mmol, 5 equiv) were combined in a culture tube, dissolved in benzene (4 mL, 0.02 M), and sealed with a Teflon-lined cap. The solution was heated overnight (18-19 h) in an oil bath at 85 °C, cooled, and passed through a plug of silica (EtOAc). The residue was purified by MPLC (1:1 Hex:EtOAc) to give, in order of elution, **13-A** (0.024 mmol, 38%) as a yellow oil and **13-B** (0.028 mmol, 43%) also as a yellow oil, which solidified upon storage at -10 °C to give as pale yellow flakes. This product returned to an oily state upon being allowed to warm to ambient temperature.

Data for 13-A (Faster eluting isomer)

^{1}H NMR (500 MHz, $CDCl_3$): δ 7.28 (dd, *J* = 8.5, 1.7 Hz, 1H, Ar*H*3), 7.19 (1H, ddd, *J* = 7.4, 7.4, 1.5 Hz, 1H, Ar*H5*), 7.17 (s, 1H, Ar*H11*), 7.10 (s, 1H, Ar*H14*), 6.96 (br d, *J* = 9.8 Hz, 1H, C=C*H2*), 6.93 (ddd, *J* = 7.4, 7.4, 1.0 Hz, 1H, Ar*H4*) , 6.49 (br d, *J* = 8.5 Hz, 1H, Ar*H*6), 6.03 (dd, *J* =9.2, 5.2 Hz, 1H, *H1*C=C), 5.51 (br d, *J* = 5.1 Hz, 1H, C*H*16a), 3.99 (s, 3H, OCH_3), 3.92 (s, 3H, OCH_3), 2.27 (s, 3H, $ArCH_3$ or $O{=}CCH_3$), 1.89 (s, 3H, $ArCH_3$ or $O{=}CCH_3$ or $C15CH_3$), 1.80 (s, 3H, $ArCH_3$ or $O{=}CCH_3$ or $C15CH_3$), and 0.48 [s, 9H, $Si(CH_3)_3$].

^{13}C NMR (125 MHz, $CDCl_3$): δ 203.5, 193.5, 153.9, 149.1, 142.1, 142.0, 140.9, 140.5, 139.7, 138.4, 137.2, 130.3, 129.7, 129.3, 128.4, 127.2, 121.7, 120.3, 117.8, 114.8, 109.7, 106.4, 82.8, 76.1, 56.7, 56.1, 25.9, 21.4, 20.0, and 3.1.

HRMS (APCI-Orbitrap): Calculated for $C_{32}H_{34}NO_5Si^+$ [M+H$^+$]: 540.2201, found 540.2204.

IR (thin film): 3004, 2924, 2852, 1718, 1704, 1644, 1601, 1588, 1526, 1499, 1488, 1456, 1419, 1350, 1311, 1291, 1243, 1221, 1118, 1092, 1074, 1044, 1024, 994, 970, 938, 909, 893, 846, 805, 772, 733, 681, 640, 608, 586, 546, 516, 495, 472, and 411 cm^{-1}.

Data for 13-B (Slower eluting isomer)

^{1}H NMR (500 MHz, $CDCl_3$): δ 7.28 (dd, *J* = 7.7, 1.7 Hz, 1H, Ar*H*3), 7.20 (1H, ddd, *J* = 7.4, 7.4, 1.5 Hz, 1H, Ar*H5*), 7.19 (s, 1H, Ar*H11*), 6.95 (br d, *J* = 8.7 Hz, 1H, C=C*H2*), 6.94 (ddd, *J* = 7.4, 7.4, 1.0 Hz, 1H, Ar*H4*), 6.65 (s, 1H, Ar*H14*), 6.47 (br d, *J* = 8.2 Hz, 1H, Ar*H*6), 6.00 (dd, *J* =9.5, 5.3 Hz, 1H, *H1*C=C), 5.50 (d, *J* = 5.2 Hz, 1H, C*H*16a), 3.919 (s, 3H, OCH_3), 3.921 (s, 3H, OCH_3), 2.37 (s, 3H, $ArCH_3$ or $O{=}CCH_3$ or $C15CH_3$), 2.22 (s, 3H, $ArCH_3$ or $O{=}CCH_3$ or $C15CH_3$), 1.70 (s, 3H, $ArCH_3$ or $O{=}CCH_3$ or $C15CH_3$) and 0.47 [s, 9H, $Si(CH_3)_3$].

^{13}C NMR (125 MHz, $CDCl_3$): δ 201.1, 193.4, 153.5, 149.2, 142.7, 141.9, 140.7, 140.3, 139.6, 138.6, 137.0, 130.2, 130.0, 129.3, 128.2, 127.5, 121.6, 120.3, 117.9, 114.5, 108.6, 106.7, 81.2, 78.3, 56.5, 56.1, 25.8, 23.6, 19.6, and 3.0.

IR (thin film): 3055, 2979, 2927, 2853, 1705, 1642, 1601, 1588, 1531, 1488, 1456, 1419, 1354, 1312, 1293, 1262, 1245, 1219, 1204, 1167, 1101, 1075, 1044, 1023, 994, 968, 941, 878, 843, 804, 772, 733, 701, 678, 629, 607, 575, 547, 514, 486, 453, and 414 cm^{-1}.

HRMS (APCI-Orbitrap): Calculated for $C_{32}H_{34}NO_5Si^+$ [$M+H^+$]: 540.2201, found 540.2201.

12',13'-Dimethoxy-8'-methyl-9'-(trimethylsilyl)-10'H,16a'H-spiro[cyclohexane-1,15'-fluoreno[3',4':4,5][1,3]oxazino[2,3-a]isoquinoline]-2,5-diene-4,10'-dione (14)

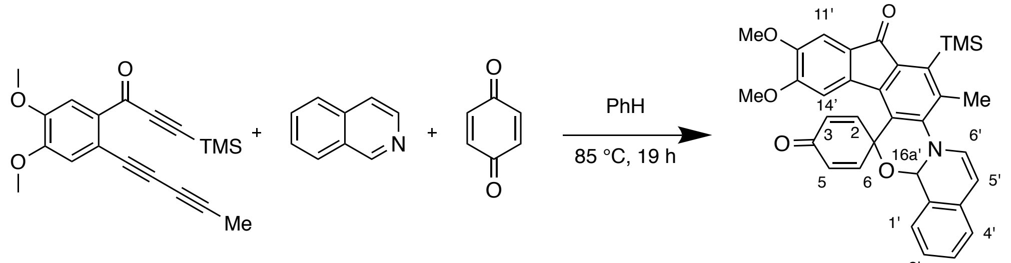

1a *2a* *3b* ***14* (60%)**

Triynone **1a** (25 mg, 0.077 mmol, 1 equiv), isoquinoline (**2a**, 30 μL, 0.231 mmol, 3 equiv), and benzoquinone (**3b**, 42 mg, 0.385 mmol, 5 equiv) were combined in a culture tube, dissolved in benzene (10 mL), and sealed with a Teflon-lined screw-cap. The tube was heated overnight (18-19 h) in an oil bath at 85 °C and cooled. The contents were passed through a plug of silica (1:1, Hex:EtOAc eluant). The eluate was concentrated and the residue was purified by MPLC (2:1 Hex:EtOAc) to give **14** (26 mg, 0.0463 mmol, 60%) as a yellow oil, which changed into a flaky, amorphous solid after being subjected to high vacuum.

Data for 101afb (contains a trace (<1%) of benzoquinone as an impurity):

^{1}H NMR (400 MHz, $CDCl_3$): δ 7.62 (dd, *J* = 10.0, 2.7 Hz, 1H, *H2* or *H6*), 7.37 (nfom, 1H, Ar*H1'*), 7.26 (m, 2H, Ar*H2'* and Ar*H3'*), 7.19 (d, *J* = 7.4 Hz, 1H, Ar*H4'*), 7,19 (s, 1H, Ar*H14'* or Ar*H11'*), 7.15 (s, 1H, Ar*H11'* or Ar*H14'*), 6.94 (dd, J = 9.8, 2.8 Hz, 1H, *H2* or *H6*), 6.51 (dd, *J* = 10.1, 1.8 Hz, 1H, *H3* or *H5*), 6.44 (dd, *J* = 9.8, 1.8 Hz, 1H, *H3* or *H5*), 6.37 (dd, *J* = 7.7, 1.5 Hz, Ar*H6'*), 6.10 (d, *J* = 1.5 Hz, 1H, *H16a'*), 5.93 (d, *J* = 7.6 Hz, 1H, *H5'*), 3.89 (s, 3H, OCH_3), 3.66 (s, 3H, OCH_3), 2.45 (s, 3H, $ArCH_3$), and 0.46 [s, 9H, $Si(CH_3)_3$].

^{13}C NMR (125 MHz, $CDCl_3$): δ 193.3, 185.4, 153.7, 149.4, 148.8, 147.6, 145.2, 145.1, 140.6, 139.1, 137.4, 136.4, 132.2, 131.2, 131.0, 130.2, 129.8, 128.7, 127.6, 126.5, 126.0, 125.2, 124.8, 110.5, 106.6, 102.7, 80.8, 71.7 (quaternary aliphatic carbon), 56.8 (OCH_3), 56.3 (OCH_3), 21.7 ($ArCH_3$), and 3.0 (TMS).

IR ($CDCl_3$): 3073, 3006, 2939, 2900, 2837, 2253, 1702, 1665, 1631, 1605, 1587, 1520, 1492, 1458, 1423, 1366, 1351, 1310, 1288, 1255, 1235, 1209, 1166, 1120, 1096, 1048, 1024, 975, 909, 884, 871, 849, 802, 772, 728, 679, 642, 603, 552, 482, and 408 cm^{-1}.

HRMS (APCI-Orbitrap): Calculated for $C_{34}H_{32}NO_5Si^+$ [M+H$^+$]: 562.2044, found 562.2037.

(±)-(15*R*,16a*R*)-12,13-Dimethoxy-1',8-dimethyl-9-(trimethylsilyl)-10H,16aH-spiro[fluoreno[3',4':4,5][1,3]oxazino[2,3-a]isoquinoline-15,3'-indoline]-2',10-dione (15-A)
and
(±)-(15*R*,16a*S*)-12,13-Dimethoxy-1',8-dimethyl-9-(trimethylsilyl)-10H,16aH-spiro[fluoreno[3',4':4,5][1,3]oxazino[2,3-a]isoquinoline-15,3'-indoline]-2',10-dione (15-B):

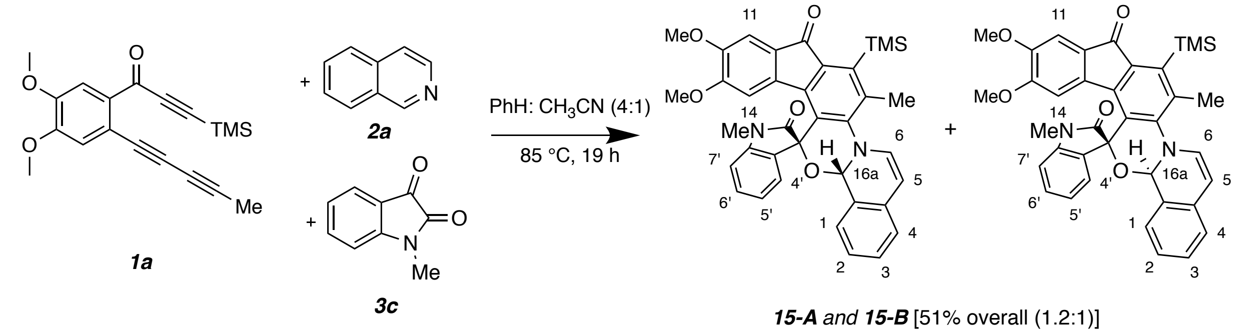

15-A** and **15-B [51% overall (1.2:1)]

Triynone **1a** (25 mg, 0.077 mmol, 1 equiv), isoquinoline (**2a**, 30 μL, 0.231 mmol, 3 equiv), and *N*–methylisatin (**3c**, 62 mg, 0.385 mmol, 5 equiv) were combined in a culture tube, dissolved in a mixture of benzene and acetonitrile (4:1, overall 10 mL), and sealed with a Teflon-lined screw-cap. The tube was heated overnight (18-19 h) in an oil bath at 85 °C and cooled. The contents were passed through a plug of silica (EtOAc elution). The eluate was concentrated and the residue was purified by MPLC (1:1 Hex:EtOAc) to give **15** (24 mg, 0.039 mmol, 51%), a yellow oil, as a coeluting mixture of diastereomers (1.2:1 ratio). A small portion of this mixture was resolved by normal phase HPLC (2:1, Hex:EtOAc) to give, **15-A** and **15-B**, each as a yellow oil. The assignment of relative configuration of each was not undertaken.

Data for the faster eluting, major diastereomer, 15-A:

^{1}H NMR (500 MHz, $CDCl_3$): δ 7.31 (ddd, *J* = 7.5, 7.5, 1.5, Hz, 1H, Ar*H3* or Ar*H6'*), 7.29$^+$ (ddd, *J* = 7.8, 7.8, 1.3 Hz, 1H, Ar*H3* or Ar*H6'*), 7.29$^-$ (dd, *J* = 7.8, 1.2 Hz, 1H, Ar*H1* or Ar*H4'*), 7.20 (ddd, *J* = 7.5, 7.5, 1.4 Hz, 1H, Ar*H2* or Ar*H5'*), 7.13 (dd, *J* = 7.7, 1.2 Hz, 1H, Ar*H4* or Ar*H7'*), 7.11 (dd, *J* = 7.4, 1.4 Hz, 1H, Ar*H1* or Ar*H4'*), 7.08 (s, 1H, Ar*H11*), 6.94 (ddd, *J* = 7.6, 7.6, 1.0 Hz, 1H, Ar*H2* or Ar*H5'*), 6.85 (ddd, *J* = 7.7, 0.8, 0.8 Hz, 1H, Ar*H7'* or Ar*H4*), 6.78 (d, *J* = 1.5 Hz, H*16a*), 6.44 (dd, *J* = 7.6, 1.4 Hz, 1H, Ar*H6*), 6.13 (s, 1H, Ar*H14*), 5.91 (d, *J* = 7.7 Hz, 1H, Ar*H5*), 3.83 (s, 3H, OCH_3), 3.54 (s, 3H, OCH_3), 3.36 (s, 3H, NCH_3), 2.46 (s, 3H, $ArCH_3$) and 0.47 [s, 9H, $Si(CH_3)_3$].

^{13}C NMR (125 MHz, $CDCl_3$): δ 193.4, 174.1, 153.5, 149.5, 147.6, 145.0, 144.8, 141.2, 138.6, 137.8, 137.1, 136.9, 132.5, 131.6, 130.8, 129.9, 129.5, 129.0, 127.9, 126.3, 125.1, 124.5, 124.1, 121.1, 109.2, 107.7, 106.7, 102.7, 80.5, 79.3, 56.8, 56.2, 27.0, 21.6, and 2.9.

IR ($CDCl_3$): 3060, 2937, 2899, 2838, 2252, 1718, 1704, 1632, 1612, 1588, 1525, 1491, 1460, 1433, 1365, 1312, 1290, 1244, 1210, 1167, 1131, 1091, 1054, 1030, 1013, 969, 942, 910, 891, 849, 811, 792, 772, 729, 690, 648, 629, 599, 553, 539, 488, 470, and 410 cm^{-1}.

HRMS (APCI-Orbitrap): Calculated for $C_{37}H_{35}N_2O_5Si^+$ [M+H$^+$]: 615.2310, found 615.2308.

Data for the slower eluting, minor diastereomer, 15-B:

^{1}H NMR (500 MHz, $CDCl_3$): δ 7.45 (ddd, *J* = 7.8, 7.8, 1.2 Hz, 1H, Ar*H3* or Ar*H6'*), 7.41 (dd, *J* = 7.3, 1.3 Hz, 1H, Ar*H1* or Ar*H4'*), 7.30 (ddd, *J* = 7.6, 7.6, 1.3 Hz, Ar*H3* or Ar*H6'*), 7.15 (dd, *J* = 7.8, 1.3 Hz, 1H, Ar*H1* or Ar*H4'*), 7.11 (ddd, *J* = 7.5, 7.5, 1.0 Hz, 1H, Ar*H2* or Ar*H5'*), 7.08 (ddd, *J* = 7.3, 7.3, 1.3 Hz, 1H, Ar*H2* or Ar*H5'*), 7.08 (s, 1H, Ar*H11*), 6.95 (d, *J* = 7.8 Hz, 1H, Ar*H7'* or Ar*H4*), 6.78 (dd, *J* = 7.7 Hz, 1.2 Hz, 1H, Ar*H7'* or Ar*H4*), 6.47 (dd, *J* = 7.6, 1.5 Hz, 1H, Ar*H6*), 6.14 (d, *J* = 1.6 Hz, H*16a*), 5.96 (s, 1H, Ar*H14*), 5.92 (d, *J* = 7.7 Hz, 1H, Ar*H5*), 3.83 (s, 3H, OCH_3), 3.42 (s, 3H, OCH_3), 3.26 (s, 3H, NCH_3) 2.49 (s, 3H, ArCH_3) and 0.46 [s, 9H, $Si(CH_3)_3$].

^{13}C NMR (125 MHz, $CDCl_3$): δ 193.4, 172.9, 153.3, 149.2, 147.1, 144.7, 143.8, 140.9, 138.1, 136.9, 136.1, 133.0, 131.8, 131.6, 130.5, 129.8, 128.5, 127.8, 125.9, 124.7, 124.5, 123.7, 123.2, 119.9, 109.4, 107.3, 106.5, 102.5, 81.2, 80.0, 56.6, 56.0, 27.0, 21.7, and 2.9.

IR ($CDCl_3$): 3057, 3001, 2938, 2902, 2837, 2253, 1737, 1705, 1633, 1609, 1559, 1525, 1492, 1460, 1427, 1365, 1313, 1288, 1247, 1211, 1120, 1095, 1053, 1030, 1012, 966, 944, 913, 881, 847, 797, 772, 754, 730, 693, 648, 629, 598, 540, and 489 cm^{-1}.

HRMS (APCI-Orbitrap): Calculated for $C_{37}H_{35}N_2O_5Si^+$ [M+H$^+$]: 615.2310, found 615.2302.

(±)-(15*R*,16a*R*)-12,13-Dimethoxy-8-methyl-9-(trimethylsilyl)-15-vinyl-16aH-fluoreno[3',4':4,5][1,3]oxazino [3,2-a]quinolin-10(15H)-one (16-A)

and

(±)-(15*R*,16a*S*)-12,13-Dimethoxy-8-methyl-9-(trimethylsilyl)-15-vinyl-16aH-fluoreno[3',4':4,5][1,3]oxazino [3,2-a]quinolin-10(15H)-one (16-B)

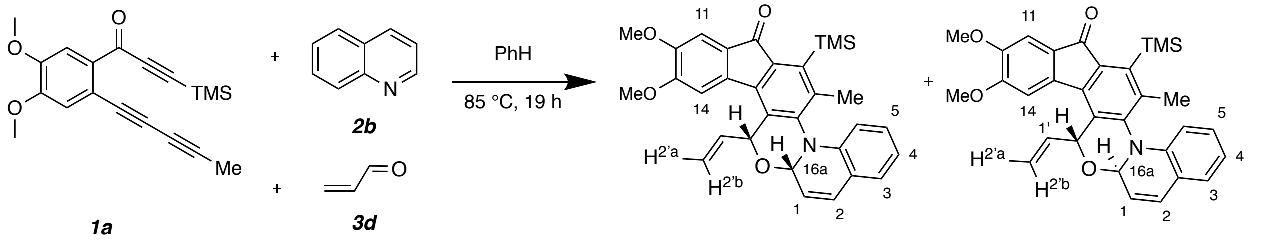

***16-A** and **16-B** (71%*, ratio from NMR*: 1.5:1)*

Triynone **1a** (25 mg, 0.077 mmol, 1 equiv), quinoline (**2b**, 30 μL, 0.231 mmol, 3 equiv), and acrolein (**3d**, 26 μL, 0.385 mmol, 5 equiv) were combined in a culture tube, dissolved in benzene (10 mL), and sealed with a Teflon-lined screw-cap. The tube was heated overnight (18-19 h) in an oil bath at 85 °C and cooled. The contents were passed through a plug of silica (1:1, Hex:EtOAc). The residue was purified by MPLC (2:1 Hex:EtOAc) to **16** (28 mg, 0.0263 mmol, 71%), a yellow oil, as a coeluting mixture of diastereomers (1.5:1 ratio). A small portion of this sample was further resolved by normal phase HPLC (2:1, Hex:EtOAc) to give, as partially overlapping peaks, **16-A** and **16-B**, each as a yellow oil. The assignment of relative configuration of each was not undertaken.

Data for the faster eluting, minor diastereomer, 16-A:

^{1}**H NMR** (500 MHz, $CDCl_3$): δ 7.22 (dd, *J* = 7.5, 1.7 Hz, 1H, Ar*H3*), 7.18 (s, 1H, Ar*H11*), 7.15 (ddd, *J* = 8.8, 7.6, 1.8 Hz, 1H, Ar*H5*), 6.91 (d, *J* = 10.0 Hz, 1H, *H2*), 6.90 (ddd, *J* = 7.4, 7.4, 1.0 Hz, 1H, Ar*H4*), 6.78 (s, 1H, Ar*H14*), 6.40 (ddd, *J* = 8.3, 1.0, 1.0 Hz, 1H, *H6*), 6.18 (ddd, *J* = 17.4, 10.6, 3.7 Hz, *H1'*), 6.06 (dd, *J* = 9.5, 5.3 Hz, 1H, *H1*), 5.72 (ddd, *J* = 3.6, 1.7, 1.7 Hz, 1H, *H15*), 5.47 (d, *J* = 5.0 Hz, 1H, *H16a*), 5.41 (ddd, *J* = 10.5, 1.4, 1.4 Hz, *H2'a*), 5.30 (ddd, *J* = 17.2, 1.4, 1.4 Hz, *H2'b*), 3.92 (s, 3H, OCH_3), 3.90 (s, 3H, OCH_3), 2.23 (s, 3H, ArCH_3), and 0.48 [s, 9H, $Si(CH_3)_3$].

^{13}**C NMR** (125 MHz, $CDCl_3$): 194.0, 153.6, 149.3, 142.9, 141.0, 140.3, 139.8, 139.0, 137.8, 137.7, 137.0, 130.0, 129.1, 128.2, 127.7, 127.3, 122.2, 120.4, 119.2, 118.7, 115.7, 108.7, 106.8, 75.9, 73.3, 56.4, 56.3, 20.0, and 3.2.

IR (thin film): 3003, 2925, 2902, 2852, 2253, 1703, 1645, 1591, 1569, 1536, 1488, 1455, 1421, 1365, 1313, 1288, 1243, 1207, 1114, 1081, 1019, 992, 935, 911, 840, 804, 772, 729, 646, 635, 602, 567, 541, 468, and 410 cm^{-1}.

HRMS (APCI-Orbitrap): Calculated for $C_{31}H_{32}NO_4Si^+$ [$M+H^+$]: 510.2095, found 510.2095.

Data for the slower eluting, major diastereomer, 16-B (contains ~20% of the minor diastereomer, **16-A**):

^{1}H NMR (500 MHz, $CDCl_3$): δ 7.25 (dd, *J* = 7.5, 1.5 Hz, 1H, Ar*H3*), 7.20 (s, 1H, Ar*H11*), 7.18 (ddd, *J* = 8.1, 8.1, 1.6 Hz, Ar*H5*), 7.00 (s, 1H, Ar*H14*), 6.92 (br d, *J* = 9.5 Hz, 1H, *H2*) 6.91 (ddd, *J* = 7.5, 7.5, 1.2 Hz, 1H, Ar*H4*), 6.48 (ddd, *J* = 8.3, 1.1, 1.1 Hz, 1H, Ar*H6*), 6.06 (dd, *J* = 9.5, 5.1 Hz, 1H, *H1*), 6.06 (dd, *J* = 5.5, 1.3 Hz, 1H, *H16a*), 5.85 (ddd, *J* = 17.0, 10.3, 5.6 Hz, *H1'*), 5.52 (d, *J* = 5.0 Hz, 1H, *H15*), 5.15 (ddd, *J* = 17.0, 1.3, 1.3 Hz, *H2'b*), 5.02 (ddd, *J* = 10.2, 1.3, 1.3 Hz, *H2'a*), 3.94 (s, 3H, OCH_3), 3.93 (s, 3H, OCH_3), 2.18 (s, 3H, $ArCH_3$) and 0.46 [s, 9H, $Si(CH_3)_3$].

^{13}C NMR (125 MHz, $CDCl_3$): 193.8, 153.7, 149.4, 143.6, 142.0, 140.0, 139.9, 139.0, 138.7, 137.6, 137.0, 136.3, 129.8, 129.2, 128.2, 127.7, 121.5, 120.0, 118.4, 117.2, 114.8, 107.8, 107.1, 79.6, 74.2, 56.4, 56.3, 19.6, and 2.8.

IR ($CDCl_3$): 3039, 3004, 2956, 2929, 2902, 2853, 2838, 2253, 1703, 1642, 1591, 1570, 1541, 1489, 1456, 1421, 1357, 1313, 1290, 1244, 1206, 1114, 1081, 1015, 907, 840, 803, 772, 727, 647, 606, 543, 459, and 409 cm^{-1}.

HRMS (APCI-Orbitrap): Calculated for $C_{31}H_{32}NO_4Si^+$ [$M+H^+$]: 510.2095, found 510.2075.

(±)-(15*R,16*R**)- and (±)-(15*R**,16*S**)-15-(4-Bromophenyl)-12,13-dimethoxy-8-methyl-9-(trimethylsilyl)-16a*H*-fluoreno[3',4':4,5][1,3]oxazino[2,3-*a*]isoquinolin-10(15*H*)-one (17-A and 17-B)**

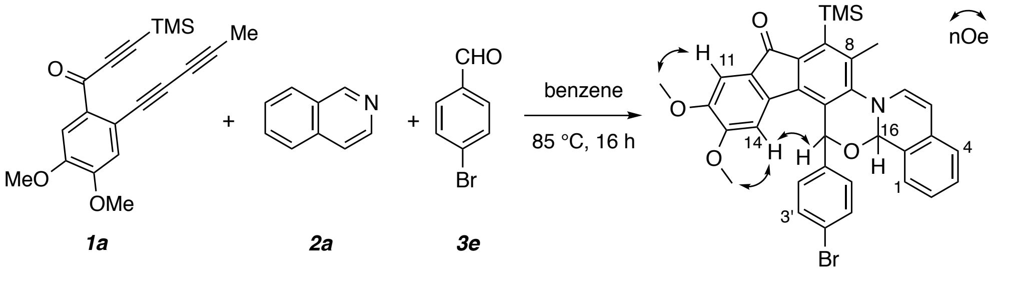

***17-A** and **17-B** (74%, ratio: 1.2:1)*

A solution of ketone **1a** (30.0 mg, 0.0924 mmol) isoquinoline (**2a**, 21.7 μL, 0.185 mmol), and *p*-bromobenzaldehyde (**3e**, 85.5 mg, 0.462 mmol) in benzene (5 mL) was heated in an 85 °C bath in a screw-capped culture tube. After 16 h the reaction mixture was concentrated and the residue was purified by MPLC (6:1 hexanes:EtOAc) to give the diastereomeric ketones **17-A** (23.9 mg, 41%) and **17-B** (19.4 mg, 33%), each as an orange, foamy, amorphous solid.

17-A (faster eluting, major isomer)

^{1}H NMR (500 MHz, $CDCl_3$): δ 7.53 (br s, 2H, Ar*H3'a* and Ar*H3'b*), 7.32 (ddd, *J* = 7.6, 7.6, 1.3 Hz, 1H, Ar*H3*), 7.32 (br s, 1H, Ar*H2'a*), 7.19–7.10 (m, 3H, Ar*H2*, Ar*H4,* Ar*H2'b*), 7.12 (s, 1H, Ar*H11*), 6.73 (d, *J* = 7.6 Hz, 1H, Ar*H1*), 6.34 (dd, *J* = 7.7, 1.5 Hz, 1H, Ar*H6*), 6.17 (s, 1H, Ar*H14*), 6.14 (s, 1H, *H15*), 5.87 (d, *J* = 7.7 Hz, 1H, Ar*H5*), 5.74 (d, *J* = 1.4 Hz, 1H, *H16*), 3.86 (s, 3H, C12OCH_3), 3.45 (s, 3H, C13OCH_3), 2.48 (s, 3H, ArCH_3), and 0.49 [s, 9H, Si(CH_3)$_3$]. (rotation about the hindered *p*-BrC_6H_4–C bond was slow enough at ambient temperature to broaden the four ArH resonances on the *p*-BrC_6H_4 ring.)

^{13}C NMR (125 MHz, $CDCl_3$): δ 193.6, 153.3, 149.0, 145.7, 143.4, 140.1, 139.7, 136.7, 136.3, 134.9, 133.1, 131.1, 130.7, 129.7, 128.3, 127.6, 126.1, 125.6, 125.1, 124.3, 122.92, 122.89, 108.3, 106.5, 102.1, 78.3, 75.2, 56.1, 56.0, 21.2, and 3.0.

IR (neat): 2999, 2932, 2903, 2848, 1702, 1633, 1591, 1569, 1535, 1493, 1457, 1431, 1400, 1367, 1314, 1284, 1244, 1211, 1120, 1098, 1057, 1031, 1009, and 958 cm^{-1}.

HRMS (ESI-TOF): Calculated for $C_{35}H_{33}BrNO_4Si^+$ [M+H$^+$] 638.1357, found 638.1343.

TLC: R_f 0.2 (6:1 hexanes:EtOAc).

17-B (slower eluting, minor isomer)

^{1}H NMR (500 MHz, $CDCl_3$): δ 7.37 (superposition of two doublets: d, *J* = 8.5 Hz, 2H, H3' and d, *J* ca. 8 Hz, 1H, H4), 7.34 (ddd, *J* = 7.4, 7.4, 1.3 Hz, 1H, *H2* or *H3*), 7.24 (ddd, *J* = 7.4, 7.4, 1.2 Hz, 1H, *H2* or *H3*), 7.15 (superposition of two doublets: d, *J* = 8.4 Hz, 2H, H2' and d, *J* ca. 8 Hz, 1H, H1), 7.12 (s, 1H, Ar*H11*), 6.64 (s, 1H, *H15*), 6.58 (s, 1H, Ar*H14*), 6.46 (dd, *J* = 7.5, 1.3 Hz, 1H, Ar*H6*), 6.06 (d, *J* = 1.1 Hz, 1H, *H16*), 5.86 (d, *J* = 7.6 Hz, 1H, Ar*H5*), 3.86 (s, 3H, C13OCH_3), 3.73 (s, 3H, C12OCH_3), 2.42 (s, 3H, ArCH_3), and 0.48 [s, 9H, Si(CH_3)$_3$].

^{13}C NMR (125 MHz, $CDCl_3$): δ 193.5, 153.4, 149.2, 146.2, 143.4, 140.0, 139.3, 137.7, 136.9, 136.1, 132.0, 131.7, 131.3, 129.6, 129.5, 128.3, 127.4, 126.6, 126.0, 125.7, 124.6, 122.6, 108.1, 106.7, 102.0, 83.0, 77.0, 56.3, 56.1, 20.2, and 2.6.

IR (neat): 3006, 2940, 2894, 1702, 1630, 1590, 1566, 1538, 1492, 1458, 1433, 1404, 1360, 1312, 1285, 1241, 1210, 1119, 1098, 1059, 1031, 1009, and 986 cm^{-1}.

HRMS (ESI-TOF): Calculated for $C_{35}H_{33}{}^{79}BrNO_4Si^+$ [M+H$^+$] 638.1357, found 638.1336.

TLC: R_f 0.1 (6:1 hexanes:EtOAc).

12,13-Dimethoxy-8-methyl-16-phenyl-9-(trimethylsilyl)-16,16a-dihydroindeno[1,2-*f*]quinolino[1,2-*a*]quinazoline-10,15-dione (18)

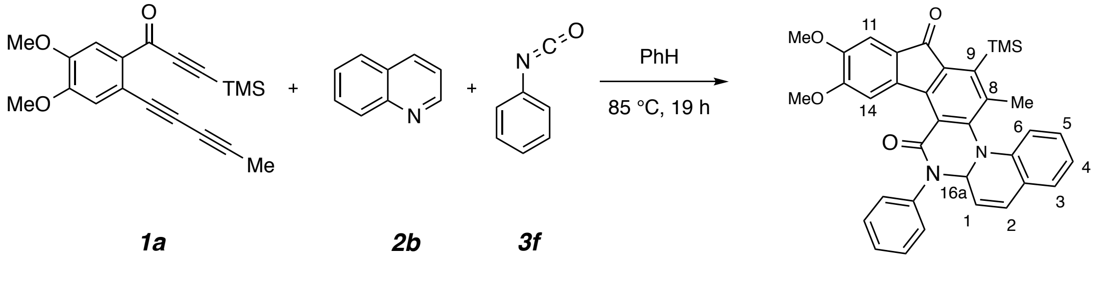

1a *2b* *3f*

18 (91%)

Triynone **1a** (20 mg, 0.062 mmol), quinoline(**2b**, 15 μl, 0.124 mmol, 2 equiv), and phenyl isocyanate (**3f**, 45.9 μL, 0.310 mmol, 5 equiv) were combined in a culture tube, dissolved in benzene (4 mL, 0.02 M), and sealed with a Teflon-lined cap. The vial was heated overnight (18-19 h) in an oil bath at 85 °C and cooled, and the contents were passed through a plug of silica (EtOAc elution). The residue was purified by MPLC (3:1 Hex:EtOAc +1% NEt_3) to give **18** (91%) as a yellow crystalline film.

Data for 18:

^{1}H NMR (500 MHz, $CDCl_3$): δ 8.10 (s, 1H, Ar*H14*), 7.32 (br m, 3H, Ph*Hs*), 7.24 (1H, ddd, *J* = 7.4, 7.4, 1.5 Hz, 1H, Ar*H5*), 7.16 (s, 1H, Ar*H11*), 7.14 (dd, *J* = 7.5, 1.5 Hz, 1H, Ar*H*3), 6.97 (ddd, *J* = 7.4, 7.4, 1.0 Hz, 1H, Ar*H4*), 6.89 (vbr s, 2H, Ph*Hs*), 6.64 (d, *J* = 9.8 Hz, 1H, C=C*H2*), 6.53 (d, *J* = 8.2 Hz, 1H, Ar*H*6), 6.01 (d, *J* = 5.9 Hz, 1H, C*H*16a), 5.53 (dd, *J* =9.6, 5.8 Hz, 1H, *H1*C=C), 3.93 (s, 3H, OCH_3), 3.89 (s, 3H, OCH_3), 2.25 (s, 3H, ArCH_3) and 0.48 [s, 9H, $Si(CH_3)_3$].

^{13}C NMR (125 MHz, $CDCl_3$): δ 193.6, 162.4, 154.0, 149.9, 146.9, 144.3, 143.8, 140.2, 138.1, 137.9, 137.2, 137.1, 129.61, 129.57, 129.3, 129.0, 128.0, 127.7, 127.5, 123.5, 122.2, 121.2, 117.2, 115.9, 110.5, 105.9, 67.8, 56.5, 56.1, 20.2, and 2.7.

IR (thin film): 2955, 2922, 2853, 1703, 1656, 1593, 1562, 1533, 1488, 1455, 1420, 1397, 1354, 1314, 1292, 1260, 1239, 1220, 1183, 1118, 1074, 1022, 1003, 955, 929, 847, 820, 804, 789, 763, 735, 696, 654, 636, 607, 596, 569, 550, 514, 463, and 433 cm^{-1}.

HRMS (APCI-Orbitrap): Calculated for $C_{35}H_{33}N_2O_4Si^+$ [M+H$^+$]: 573.2204, found 573.2201.

mp: >230 °C.

16-Cyclohexyl-12,13-dimethoxy-8-methyl-9-(trimethylsilyl)-16,16a-dihydroindeno[1,2-f]quinolino[1,2-a]quinazoline-10,15-dione (19)

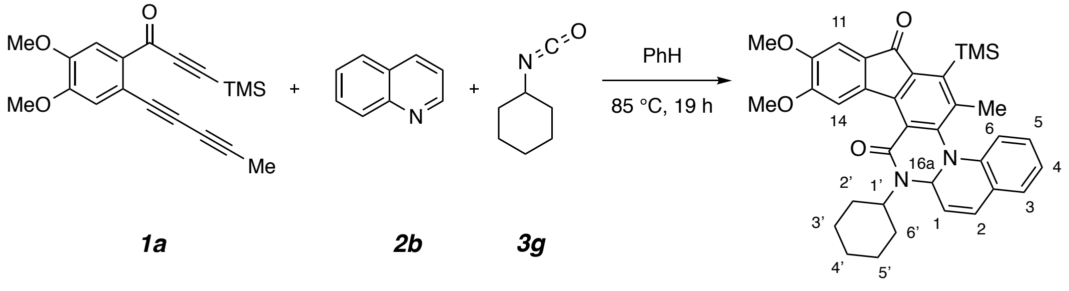

Triynone **1a** (25 mg, 0.077 mmol), quinoline (**2b**, 28 μL, 0.231 mmol, 3 equiv), and cyclohexyl isocyanate (**3g**, 49 μL, 0.385 mmol, 5 equiv) were combined in a culture tube, dissolved in benzene (10 mL), and sealed with a Teflon-lined screw-cap. The tube was heated overnight (18-19 h) in an oil bath at 85 °C and cooled. The contents were passed through a plug of silica (1:1, Hex:EtOAc). The residue was purified by MPLC (3:1 Hex:EtOAc) to give **19** (74%) as a yellow oil.

Data for 19:

^{1}H NMR (500 MHz, $CDCl_3$): δ 8.05 (s, 1H, Ar*H14*), 7.24 (dd, *J* = 7.5, 1.3 Hz, 1H, Ar*H3*), 7.19 (ddd, *J* = 8.2, 7.5, 1.5 Hz, 1H, Ar*H5*), 7.14 (s, 1H, Ar*H11*), 6.98 (br d, *J* = 9.6 Hz, 1H, *H2*), 6.94 (ddd, *J* = 8.4, 7.4, 1.0 Hz, 1H, Ar*H4*), 6.45 (d, *J* = 8.0 Hz, 1H, Ar*H6*), 5.96 (dd, *J* = 9.5, 6.1, 1H, *H1*C=C), 5.66 (d, *J* = 6.1 Hz, 1H, C*H*16a), 4.00 (s, 3H, OCH_3), 3.93 (s, 3H, OCH_3'), 3.44 (br m, 1H, *H1'*), 2.58 (br m, 1H), 2.04 (dddd, *J* = 12.6, 12.6, 12.6, 3.9 Hz, 1H), 2.16 (s, 3H, ArCH_3), 1.81 (br d, *J* = 12.7 Hz, 1H), 1.67 (br d, *J* = 12.0 Hz, 1H), 1.61 (br d, *J* = 12.9 Hz, 1H), 1.56 (br d, *J* = 12.1 Hz, 1H), 1.21 (ddddd, *J* = 13.0, 13.0, 13.0, 3.2, 3.2 Hz, 1H), 1.16 (ddddd, *J* = 12.8, 12.8, 12.8, 3.3, 3.3 Hz, 1H), and 1.06 (ddddd, *J* = 12.9, 12.9, 12.9, 3.5, 3.5 Hz, 1H), and 0.45 [s, 9H, $Si(CH_3)_3$].

^{13}C NMR (125 MHz, $CDCl_3$): δ 193.8, 162.7, 153.9, 149.8, 146.1, 144.1, 143.1, 139.9, 138.1, 137.5, 137.3, 130.2, 129.7, 127.6, 124.6, 122.3, 121.0, 116.9, 116.3, 110.2, 106.0, 67.4, 56.4, 56.2, 55.7, 30.0, 29.3, 26.9, 26.4, 25.3, 20.2, and 2.8.

IR (thin film): 2930, 2853, 2253, 1703, 1638, 1599, 1567, 1536, 1488, 1455, 1359, 1315, 1289, 1246, 1219, 1114, 1085, 1053, 1022, 1003, 903, 881, 844, 788, 767, 728, 698, 646, 635, 607, 595, 570, 551, 519, 467, and 418 cm^{-1}.

HRMS: (APCI-Orbitrap): Calculated for $C_{35}H_{39}N_2O_4Si^+$ [M+H$^+$]: 579.2674, found 579.2651.

Methyl-11-methyl-2-(methylsulfonyl)-4-oxo-5-phenyl-12-(prop-1-yn-1-yl)-1,2,3,4,5,5a-hexahydropyrido[1,2-a]pyrrolo[3,4-f]quinazoline-8-carboxylate (20-A) and Methyl-5-methyl-2-(methylsulfonyl)-6-oxo-7-phenyl-4-(prop-1-yn-1-yl)-1,2,3,6,7,7a-hexahydropyrido[1,2-a]pyrrolo[3,4-h]quinazoline-10-carboxylate (20-B):

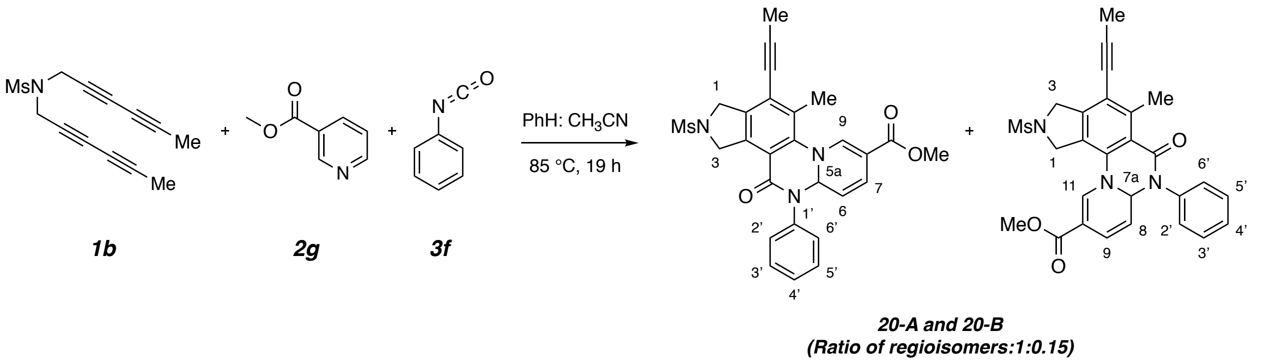

Tetrayne **1b** (60 mg, 0.242 mmol), methyl nicotinate (**2g**, 100 mg, 0.727 mmol, 3 equiv), and phenyl isocyanate (**3f**, 132 μL, 1.21 mmol, 5 equiv) were combined in a culture tube, dissolved in a mixture of benzene and acetonitrile (14 mL, 3:2 ratio), and sealed with a Teflon-lined cap. The solution was heated overnight (18-19 h) in an oil bath at 85 °C, cooled, and passed through a plug of silica (pure EtOAc). The residue was purified by gradient flash chromatography–first by 2:1, Hex:EtOAc and then pure EtOAc to obtain a crude mixture of isomers (ratio: 1:0.15). This product was repurified by MPLC (1:1, EtOAc:Hex+ 1% NEt_3) to give **20** as a pale brown powder, which was obtained as a mixture of coeluting isomers **20-A** and **20-B** (86 mg, 71% yield, ratio: 1:0.15).

Data for the major isomer:

^{1}H NMR (500 MHz, $CDCl_3$): δ 7.44–7.36 (m, 4H, Ph*Hm*, Ph*Hp* and *H9*), 7.08 (br d, *J* = 6.5 Hz, 2H, Ph*Ho*), 6.71 (dd, *J* = 10.0, 1.0 Hz, 1H, *H7*), 6.23 (d, *J* = 5.0 Hz, 1H, *H5a*), 5.22 (br d, *J* = 16.1 Hz, 1H, MsNC3H_aC3H_b), 5.02 (dd, *J* = 10.1, 5.0 Hz, 1H, *H6*) 4.99 (dd, *J* = 16.5, 2.7 Hz, 1H, MsNC3H_aC3H_b), 4.80 (br d, *J* = 15.1 Hz, 1H, MsN*C1*H_aC1H_b), 4.70 (br d, *J* = 15.0 Hz, 1H, MsNC1H_aC1H_b), 3.77 (s, 3H, CO_2*Me*), 2.88 (s, 3H, CH_3SO_2N), 2.51 (s, 3H, NArCH_3), and 2.19 (s, 3H, C≡CCH_3).

^{13}C NMR (125 MHz, $CDCl_3$): δ 165.9, 161.7, 141.6, 140.8, 138.8, 138.2, 136.3, 135.7, 134.7, 131.4, 129.4, 128.5, 124.7, 120.2, 110.0, 104.3, 99.4, 75.3, 68.1, 55.6, 53.6, 51.6, 35.3, 16.2, and 5.0.

HRMS (APCI-Orbitrap): Calculated for $C_{23}H_{26}N_3O_5S^+$ [M+H$^+$]: 504.1588, found 504.1587.

IR ($CDCl_3$): 3066, 2952, 2920, 2852, 2253, 2234, 1726, 1698, 1642, 1593, 1531, 1494, 1436, 1406, 1336, 1262, 1191, 1153, 1114, 1075, 1025, 961, 907, 824, 725, 692. 647, 613, 565, and 518 cm^{-1}.

^{1}H NMR identifiable, unique resonances for the minor isomer (500 MHz, $CDCl_3$): 6.64 (s, 1H, *H9*) 6.60 (d, *J* = 7.2 Hz, 1H, *H5a* or *H7*), 5.57 (dd, *J* = 6.7, 6.7 Hz, 1H, *H6*), 5.26 (br d, 1H, MsNC3H_aC3H_b), 4.99 (br d, 1H, MsNC3H_aC3H_b), 3.48 (s, 3H, CO_2Me), and 2.45 (s, 3H, NArCH_3). (the integral values for resonances corresponding to H1, H3, PhH_m, PhH_p, CH_3SO_2, and $CH_3C{\equiv}C$ suggest that these are overlapping from both major and minor isomers.)

Dimethyl-12,13-Dimethoxy-8-methyl-10-oxo-9-(trimethylsilyl)-10,16a dihydroindeno[1,2*f*]quinolino [1,2-*a*]quinoline-15,16-dicarboxylate (21)

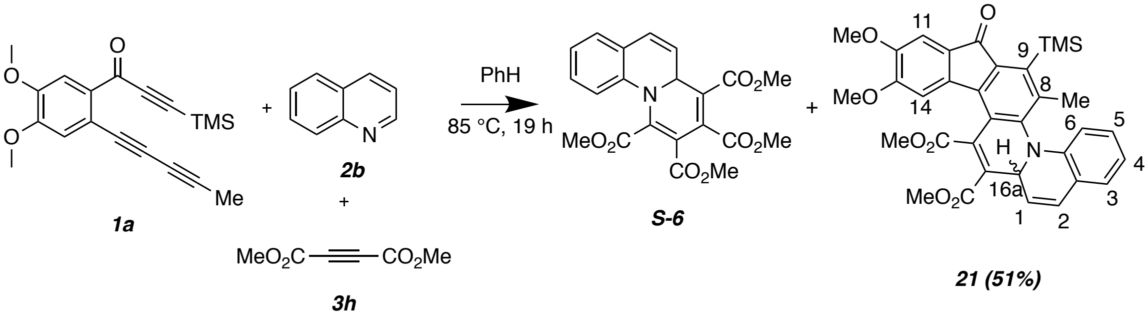

Triynone **1a** (20 mg, 0.062 mmol), quinoline (**2e**, 22 μL, 0.186 mmol, 3 equiv) and dimethyl acetylenedicarboxylate (**3h**, 76 μL, 0.62 mmol, 10 equiv) were combined in a culture tube, dissolved in benzene (4 mL, 0.02 M), and sealed with a Teflon-lined cap. The solution was heated overnight (18-19 h) in an oil bath at 85 °C, cooled, and passed through a plug of silica (EtOAc). The residue was purified by MPLC (3:2 Hex:EtOAc) to give, in order of elution, the known 2:1 adduct of quinoline and **3h** [**S-6** (25 mg, 0.060 mmol) as a pale yellow solid] followed by the three-component coupling product **21** (19 mg, 51%) as a yellow oil. The latter was further purified by HPLC (3:2 Hex:EtOAc) to give a more pure sample of **21** that was used for collection of spectral data.

Data for S-6: Characterization data were in accordance with the reported literature.[6]

Data for 21:

^{1}H NMR (500 MHz, $CDCl_3$): δ 7.16 (s, 1H, Ar*H11*), 7.07 (1H, ddd, *J* = 7.8, 7.8, 1.6 Hz, 1H, Ar*H5*), 7.03 (dd, *J* = 7.4, 1.4 Hz, 1H, Ar*H*3), 6.82 (ddd, *J* = 7.4, 7.4, 0.9 Hz, 1H, Ar*H4*), 6.72 (s, 1H, Ar*H14*), 6.67 (ddd, *J* = 9.7, 0.8, 0.8 Hz, 1H, C=C*H2*), 6.31 (d, *J* = 8.2 Hz, 1H, Ar*H*6), 5.97 (dd, *J* = 9.8, 6.1 Hz, 1H, *H1*C=C), 4.71 (dd, *J* = 6.1, 0.7 Hz, 1H, C*H*16a), 3.92 (s, 3H, OCH_3), 3.90 (s, 3H, OCH_3), 3.74 (s, 3H, CO_2CH_3), 3.56 (s, 3H, CO_2CH_3), 2.27 (s, 3H, $ArCH_3$), and 0.48 [s, 9H, $Si(CH_3)_3$].

^{13}C NMR (125 MHz, $CDCl_3$): δ 193.6, 166.4, 165.9, 154.2, 149.5, 144.5, 142.6, 141.3, 140.3, 138.6, 138.5, 138.2, 138.1, 129.2, 128.8, 127.6, 127.1, 126.3, 122.3, 121.6, 120.8, 119.5, 115.7, 106.7, 105.9, 56.6, 56.3, 54.9, 52.7, 52.5, 21.0, and 2.8.

IR (thin film): 2956, 2921, 2852, 1736, 1728, 1711, 1659, 1598, 1531, 1486, 1458, 1365, 1302, 1259, 1218, 1136, 1092, 1021, 849, 801, 721, 637, and 608 cm^{-1}.

HRMS (APCI-Orbitrap): Calculated for $C_{34}H_{32}NO_7Si^+$ [M–H$^-$]: 594.1943, found 594.1940.

(±)-(14d*R*,17a*S*,17b*R*)- and (±)-(14d*R*,17a*S*,17b*S*)-12,13-Dimethoxy-8,16-dimethyl-9-(trimethylsilyl)-17a,17b-dihydro-10H-indeno[1,2-f]pyrrolo[3,4-c]quinolino[1,2-a]quinoline-10,15,17(14dH,16H)-trione (22-cis/anti) and (22-cis/syn), respectively.

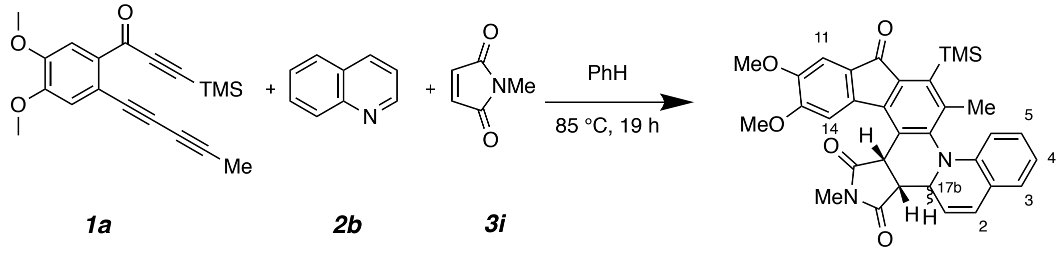

1a ***2b*** ***3i***

22-cis/anti H_{17b} "down" (58%)
22-cis/syn H_{17b} "up"(22%)
80% (combined)

Triynone **1a** (30 mg, 0.092 mmol), quinoline (**2b**, 22 μL, 0.185 mmol, 2 equiv) and *N*-methylmaleimide (**3i**, 51 mg, 0.460 mmol, 5 equiv) were combined in a culture tube, dissolved in benzene (6 mL, 0.02 M), and sealed with a Teflon-lined screw-cap. The solution was heated overnight (18-19 h) in an oil bath at 85 °C, cooled, and passed through a plug of silica (EtOAc). The filtrate was concentrated and the residue was purified by MPLC (1:1 Hex:EtOAc + 1% NEt_3) to give, in order of elution, **22-cis/anti** (30 mg, 58% yield) as a yellow oil and **22-cis/syn** as a yellow amorphous solid (11 mg, 22% yield).

Data for 22-cis/anti:

^{1}H NMR (500 MHz, $CDCl_3$): δ 7.20 (s, 1H, Ar*H11*), 7.09 (d, *J* = 7.4 Hz, 1H, Ar*H*3), 7.00 (1H, dd, *J* = 7.9, 7.9, 1H, Ar*H5*), 6.82 (s, 1H, Ar*H14*), 6.82 (ddd, *J* = 7.5, 7.5, 1.0 Hz, 1H, Ar*H4*), 6.71 (d, *J* = 9.9 Hz, 1H, C=C2*H*), 6.04 (d, *J* = 8.4 Hz, 1H, Ar*H*6), 6.01 (dd, *J* = 9.6, 5.8 Hz, 1H, *H*C1=C2), 4.52 (d, *J* = 7.0 Hz, 1H, C*H14d*), 3.97 (dd, *J* =10.4, 6.0 Hz, 1H, *H17b*), 3.93 (s, 3H, OCH_3), 3.86 (s, 3H, OCH_3), 3.52 (dd, *J* =10.3, 7.0 Hz, 1H, *H17a*), 3.05 (s, 3H, NCH_3), 2.22 (s, 3H, ArCH_3), and 0.44 [s, 9H, Si$(CH_3)_3$].

^{13}C NMR (125 MHz, $CDCl_3$): δ 193.9, 174.9, 174.3, 153.6, 149.7, 143.7, 143.5, 142.0, 140.6, 139.9, 139.6, 138.8, 129.4, 127.84, 127.78, 127.77, 123.5, 121.9, 120.7, 120.1, 115.3, 107.9, 106.5, 56.5, 56.3, 56.2, 43.0, 42.9, 25.4, 19.6, and 3.2.

HRMS (APCI-Orbitrap): Calculated for $C_{33}H_{33}N_2O_5Si^+$ [M+H$^+$]: 565.2153, found 565.2151.

IR (thin film): 3056, 2943, 2898, 2836, 2253, 1776, 1699, 1591, 1567, 1534, 1485, 1454, 1431, 1366, 1313, 1274, 1241, 1204, 1113, 1089, 1067, 1029, 993, 963, 928, 911, 838, 810, 797, 772, 728, 701, 680, 647, 622, 605, 573, 555, 515, 445, and 411 cm^{-1}.

Data for 22-cis/syn:

^{1}H NMR (500 MHz, $CDCl_3$): δ 7.22 (s, 1H, Ar*H11*), 7.15 (s, 1H, Ar*H14*), 7.01 (d, *J* = 7.6 Hz, 1H, Ar*H*3), 6.96 (1H, ddd, *J* = 7.7, 7.7, 1.4 Hz, 1H, Ar*H5*), 6.71 (d, *J* = 10.1 Hz, 1H, C=C2*H*), 6.70 (ddd, *J* = 7.5, 7.5, 1.2 Hz, 1H, Ar*H4*), 6.11 (d, *J* = 8.2 Hz, 1H, Ar*H*6), 6.02 (dd, *J* = 9.9, 5.6 Hz, 1H, *H*C1=C2), 4.69 (d, *J* = 8.6 Hz, 1H, C*H14d*), 4.44 (dd, *J* =5.8, 5.8 Hz, 1H, *H17b*), 3.95 (s, 3H, OCH_3), 3.94 (s, 3H, OCH_3), 3.35 (dd, *J* = 8.6, 5.8 Hz, 1H, *H17a*), 2.47 (s, 3H, NCH_3), 2.13 (s, 3H, ArCH_3), and 0.43 [s, 9H, $Si(CH_3)_3$].

^{13}C NMR (125 MHz, $CDCl_3$): δ 193.5, 175.4, 175.1, 153.9, 149.8, 145.5, 143.0, 142.8, 140.0, 139.6, 139.5, 138.5, 129.5, 128.2, 127.9, 127.5, 124.8, 122.4, 121.0, 119.9, 112.4, 107.2, 106.6, 56.9, 56.7, 56.3, 50.9, 41.9, 25.1, 19.3, and 2.9.

IR (thin film): 3053, 2944, 2899, 2837, 1774, 1695, 1573, 1545, 1486, 1455, 1433, 1380, 1359, 1301, 1242, 1219, 1160, 1106, 1047, 1022, 954, 908, 845, 799, 769, 730, 700, 678, 647, 606, 573, 542, 502, 443, and 409 cm^{-1}.

HRMS (APCI-Orbitrap): Calculated for $C_{33}H_{33}N_2O_5Si^+$ [M+H$^+$]: 565.2153, found 565.2137

(±)-(14d*R*,17a*S*,17b*R*)- and (±)-(14d*R*,17a*S*,17b*S*)-12,13-Dimethoxy-8-methyl-16-phenyl-9-(trimethylsilyl)-17a,17b-dihydro-10*H*-indeno[1,2-*f*]pyrrolo[3,4-*c*]quinolino[1,2-*a*]quinoline-10,15,17(14d*H*,16*H*)-trione: (23-cis/anti) and (23-cis/syn), respectively.

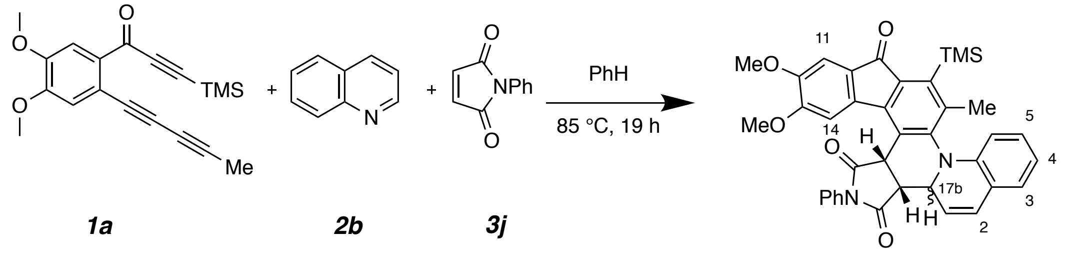

23-cis/anti H_{17b} "down" (32%)
23-cis/syn H_{17b} "up"(40%)
72% (combined)

Triynone **1** (30 mg, 0.092 mmol), quinoline (**2b**, 22 μL, 0.185 mmol, 2 equiv) and *N*-phenylmaleimide (**3j**, 79.7 mg, 0.460 mmol, 5 equiv) were combined in a culture tube, dissolved in benzene (6 mL, 0.02 M), and sealed with a Teflon-lined cap. The solution was heated overnight (18-19 h) in an oil bath at 85 °C, cooled, and passed through a plug of silica (EtOAc). The eluate was concentrated and the residue was purified by MPLC (2:1 Hex:EtOAc) to give, in order of elution **23-cis/anti** (32%) as a yellow oil and **23-cis/syn** (40%), which solidified upon storage at -10 °C to a pale yellow amorphous powder.

Data for faster eluting, minor isomer (23-cis/anti):

^{1}H NMR (500 MHz, $CDCl_3$): δ 7.45 (t, *J* = 7.8 Hz, 2H, ArH_m), 7.39 (t, *J* = 7.4 Hz, 1H, ArH_p), 7.29 (d, *J* = 7.8 Hz, 2H, ArH_o), 7.19 (s, 1H, Ar*H11*), 7.11 (d, *J* = 7.3 Hz, 1H, Ar*H3*), 7.03 (dd, *J* = 7.4, 7.4 Hz, 1H, Ar*H5*), 6.88 (s, 1H, Ar*H14*), 6.85 (dd, J = 7.4, 7.4 Hz, 1H, Ar*H4*), 6.72 (d, *J* = 9.8, 1H, C=C2*H*), 6.10 (d, *J* = 8.1 Hz, 1H, Ar*H6*), 6.04 (dd, *J* = 9.6, 5.9 Hz, 1H, *H*C1=C2), 4.70 (d, *J* = 7.0 Hz, 1H, *H14d*), 4.17 (dd, *J* = 10.2, 5.9 Hz, *H17b*), 3.92 (s, 3H, OCH_3), 3.87 (s, 3H, OCH_3), 3.62 (dd, *J* = 10.3, 7.0 Hz, 1H, *H17a*), 2.25 (s, 3H, $ArCH_3$), and 0.45 [s, 9H, $Si(CH_3)_3$].

^{13}C NMR (125 MHz, $CDCl_3$): δ 193.9, 173.6, 173.3, 153.6, 149.7, 143.9, 143.4, 142.1, 140.5, 139.9, 139.5, 138.8, 131.6, 129.41, 129.40, 129.0, 128.0, 127.84, 127.83, 126.1, 123.5, 121.7, 120.8, 119.9, 115.4, 107.7, 106.5, 56.4, 56.4, 56.3, 43.1, 43.0, 19.7, and 3.2.

HRMS (APCI-Orbitrap): Calculated for $C_{38}H_{35}N_2O_5Si^+$ [M+H$^+$]: 627.2310, found 627.2304.

IR (thin film): 3066, 2945, 2899, 2836, 1707, 1596, 1536, 1497, 1455, 1376, 1316, 1244, 1207, 1115, 1066, 1023, 852, 808, 772, 743, 690, and 609 cm^{-1}.

Data for slower eluting, major isomer (23-cis/syn):

^{1}H NMR (500 MHz, $CDCl_3$): 7.25–7.23 (m, 3H, Ph*H*), 7.22 (s, 1H, Ar*H14* or Ar*H11*), 7.21 (s, 1H, Ar*H14* or Ar*H11*), 7.02 (dd, *J* = 7.5, 1.5 Hz, 1H, Ar*H3*), 7.00 (ddd, *J* = 7.6, 7.6, 1.6 Hz, 1H, Ar*H5*), 6.76 (dd, *J* = 7.6, 7.6, 1.0 Hz, 1H, Ar*H4*), 6.69 (ddd, *J* = 9.9, 1.1, 1.1 Hz, 1H, C=C2*H*), 6.48–6.49 (m, 2H, PhH), 6.21 (d, *J* = 8.0 Hz, 1H, Ar*H6*), 6.04 (dd, *J* = 9.8, 5.5 Hz, 1H, *H*C1=C2), 4.89 (d, *J* = 9.0 Hz, 1H, Ar*H14d*), 4.61 (ddd, *J* = 5.7, 5.7, 1.1 Hz, *H17b*), 3.98 (s, 3H, OCH_3), 3.93 (s, 3H, OCH_3), 3.54 (dd, *J* = 8.8, 5.8 Hz, 1H, *H17a*), 2.16 (s, 3H, ArCH_3), and 0.43 [s, 9H, $Si(CH_3)_3$].

^{13}C NMR (125 MHz, $CDCl_3$): 193.4, 174.2, 174.1, 153.9, 149.9, 145.4, 143.3, 143.0, 140.0, 139.6, 139.4, 138.4, 131.6, 129.8, 128.9, 128.7, 128.3, 127.9, 127.7, 126.3, 124.6, 122.2, 121.0, 119.9, 112.8, 107.2, 106.8, 57.2, 56.8, 56.3, 51.6, 41.9, 19.6, and 2.9.

HRMS (APCI-Orbitrap): Calculated for $C_{38}H_{35}N_2O_5Si^+$ [M+H$^+$]: 627.2310, found 627.2304.

IR (thin film): 3055, 2945, 2899, 2837, 1704, 1594, 1487, 1455, 1380, 1301, 1243, 1204, 1108, 1019, 923, 843, 799, 766, 732, 694, 650, 606, 542, 523, and 462 cm^{-1}.

10,11-Dimethoxy-6-methyl-7-(trimethylsilyl)fluoreno[4',3':3,4]azeto[1,2-f]phenanthridin-8(12dH)-one (9),

(±)- (16dS,19aR,19bR)-14,15-dimethoxy-10,18-dimethyl-11-(trimethylsilyl)-19a,19b-dihydro-12H-indeno[1',2':5,6]pyrrolo[3',4':3,4]quinolino[1,2-f]phenanthridine-12,17,19(16dH,18H)-trione (24-cis/anti), and

(±)-(16dS,19aR,19bS)-14,15-Dimethoxy-10,18-dimethyl-11-(trimethylsilyl)-19a,19b-dihydro-12H-indeno[1',2':5,6]pyrrolo[3',4':3,4]quinolino[1,2-f]phenanthridine-12,17,19(16dH,18H)-trione (24-cis/syn):

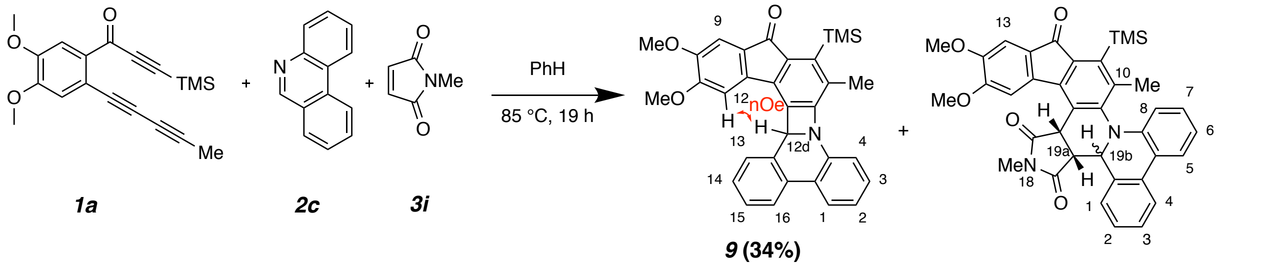

24-cis/anti H_{19b} "down" (30%)
24-cis/syn H_{19b} "up"(22%)

Triynone **1a** (25 mg, 0.077 mmol), phenanthridine (**2c**, 41 mg, 0.231 mmol, 3 equiv), and N-methyl maleimide (**3i**, 70 mg, 0.615 mmol, 5 equiv) were combined in a culture tube, dissolved in benzene (10 mL), and sealed with a Teflon-lined cap. The solution was heated overnight (18-19 h) in an oil bath at 85 °C, cooled, and passed through a plug of silica (1:1, Hex:EtOAc). The residue was purified by MPLC (1:1, Hex:EtOAc) to give, in order of elution, **1a** (13 mg, 0.026 mmol, 34%; characterized on page S14, above) as an orange oil, **24-cis/anti** (14 mg, 0.023 mmol, 30%) as a yellow amorphous solid, and **24-cis/syn** (10 mg, 0.016 mmol, 22%), also as an orange oil.

Data for the faster eluting, three-component isomer 24-cis/anti

^{1}H NMR (500 MHz, CDCl$_3$): δ 7.86 (dd, *J* = 8.0, 1.2 Hz, 1H, Ar*H4*), 7.82 (dd, *J* = 7.8, 1.6 Hz, 1H, Ar*H5*), 7.50 (ddd, *J* = 7.6, 7.6 , 1.4 Hz, 1H, Ar*H3*), 7.33 (ddd, *J* = 7.6, 7.6, 1.3 Hz, 1H, Ar*H2*), 7.22 (s, 1H, Ar*H13*), 7.10 (ddd, *J* = 8.2, 7.3, 1.5 Hz, 1H, Ar*H7*), 7.00 (ddd, *J* = 7.6, 7.6, 1.2 Hz, 1H, Ar*H6*), 6.87 (dd, *J* = 7.6, 1.3 Hz, 1H, Ar*H1),* 6.22 (s, 1H, Ar*H16*), 6.15 (dd, *J* = 8.2, 1.2 Hz, 1H, Ar*H8*), 4.53 (d, *J* = 7.0 Hz, 1H, *H19b*), 4.32 (d, *J* = 10.4 Hz, 1H, *H16d*), 3.94 (s, 3H, C14OC*H$_3$*), 3.85 (s, 3H, C15OC*H$_3$*), 3.47 (dd, *J* = 10.4, 6.7 Hz, 1H, *H19a*), 3.11 (s, 3H, N*Me*), 2.23 (s, 3H, ArCH$_3$), and 0.38 [s, 9H, Si(CH$_3$)$_3$].

^{13}C NMR (126 MHz, CDCl$_3$): δ 193.9, 174.7, 174.4, 153.6, 149.7, 144.4, 143.8, 142.1, 141.3, 140.7, 139.5, 139.0, 130.9, 130.5, 129.5, 129.4, 128.3, 127.8, 127.2, 124.6, 123.5, 123.1, 121.5, 120.2, 116.7, 107.8, 106.6, 61.2, 56.4, 56.3, 43.6, 42.5, 25.4, 19.2, and 3.2.

HRMS (APCI-Orbitrap): Calculated for $C_{37}H_{35}N_2O_5Si^+$ [M+H$^+$]: 615.2310, found 615.2305.

IR (CDCl$_3$): 3069, 3000, 2941, 2899, 2837, 2251, 1777, 1699, 1591, 1568, 1536, 1494, 1457, 1436, 1353, 1314, 1271, 1242, 1208, 1117, 1088, 1067, 1026, 991, 967, 940, 910, 849, 799, 749, 728, 669, 647, 608, 586, 506, and 455 cm^{-1}.

Data for slower eluting, three-component isomer 24-cis/syn:

^{1}H NMR (500 MHz, CDCl$_3$): δ 7.88 (dd, *J* = 8.0, 1.0 Hz, 1H, Ar*H4*), 7.77 (dd, *J* = 7.8, 1.6 Hz, 1H, Ar*H5*), 7.50 (ddd, *J* = 7.7, 7.7, 1.4 Hz, 1H, Ar*H3*), 7.40 (ddd, *J* = 7.5, 7.5, 1.3 Hz, 1H, Ar*H2*), 7.26 (dd, *J* = 7.6, 1.3 Hz, 1H, Ar*H1),* 7.24 (s, 1H, Ar*H13* or Ar*H16*), 7.18 (s, 1H, Ar*H13* or Ar*H16*), 7.06 (ddd, *J* = 8.7, 7.5, 1.6 Hz, 1H, Ar*H7*), 6.88 (ddd, *J* = 7.6, 1.2 Hz, 1H, Ar*H6*), 6.25 (dd, *J* = 8.1, 1.1 Hz, 1H, Ar*H8*), 4.86 (d, *J* = 6.0 Hz, 1H, *H19b*), 4.75 (d, *J* = 8.9 Hz, 1H, *H16d*), 3.950 (s, 3H, OC*H$_3$*), 3.947 (s, 3H, OC*H$_3$*), 3.51 (dd, *J* = 8.8, 6.0 Hz, 1H, *H19a*), 2.35 (s, 3H, N*Me*), 2.15 (s, 3H, ArCH$_3$), and 0.43 [s, 9H, Si(CH$_3$)$_3$].

^{13}C NMR (126 MHz, CDCl$_3$): δ 193.6, 175.3, 174.9, 154.0, 149.8, 145.8, 143.2, 142.8, 140.6, 140.5, 140.0, 138.5, 132.0, 130.6, 129.5, 129.0, 128.2, 127.9, 126.5, 124.6, 124.3, 123.0, 122.9, 120.7, 113.7, 107.1, 106.8, 59.6, 56.7, 56.3, 49.9, 41.7, 24.9, 18.7, and 3.0.

HRMS (APCI-Orbitrap): Calculated for $C_{37}H_{35}N_2O_5Si^+$ [M+H$^+$]: 615.2310, found 615.2276.

IR (CDCl$_3$): 3069, 2999, 2945, 2904, 2838, 2253, 1775, 1698, 1602, 1546, 1495, 1439, 1382, 1361, 1302, 1285, 1244, 1215, 1113, 1079, 1022, 992, 911, 848, 797, 781, 752, 732, 678, 647, 602, and 526 cm^{-1}.

(±)-(12d*S*,15a*R*,15b*S*)-10,11-Dimethoxy-6,14-dimethyl-7-(trimethylsilyl)-15a,15b-dihydro-8H-indeno[1,2-f]pyrido[1,2-a]pyrrolo[3,4-c]quinoline-8,13,15(12dH,14H)-trione (25-cis/anti)

(±)-(12d*S*,15a*R*,15b*S*)-10,11-Dimethoxy-6,14-dimethyl-7-(trimethylsilyl)-15a,15b-dihydro-8H-indeno[1,2-f]pyrido[1,2-a]pyrrolo[3,4-c]quinoline-8,13,15(12dH,14H)-trione (25-cis/syn)

and

10,11-Dimethoxy-2,6,14-trimethyl-7-(trimethylsilyl)-3a,12d,15a,15b,16,16a-hexahydro-4,16-ethenoindeno[1,2-f]pyrrolo[3,4-c]pyrrolo[3',4':4,5]pyrido[1,2-a]quinoline-1,3,8,13,15(2H,4H,14H)-pentaone (26)

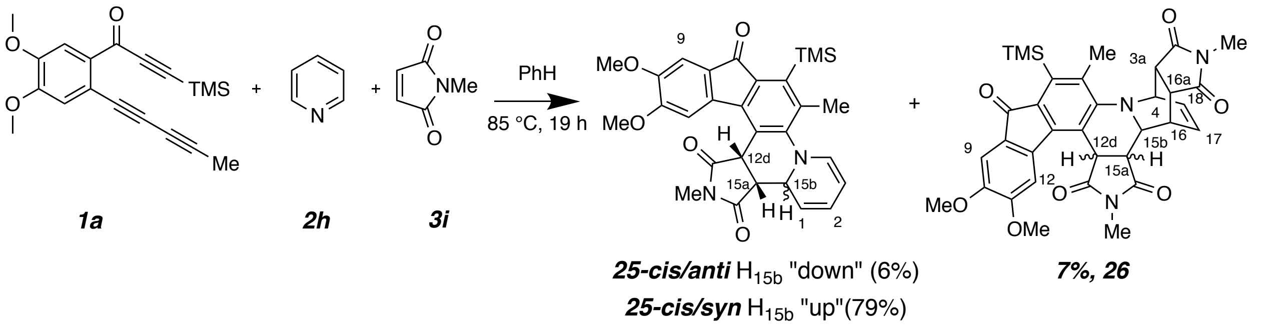

Triynone **1a** (50 mg, 0.123 mmol, 1equiv), pyridine (**2h**, 30 μL, 0.369 mmol, 3 equiv), and N-methyl maleimide (**3i**, 68 mg, 0.615 mmol, 5 equiv) were combined in a culture tube, dissolved in a mixture of benzene (10 mL), and sealed with a Teflon-lined cap. The solution was heated overnight (18-19 h) in an oil bath at 85 °C, cooled, and passed through a plug of silica (EtOAc). The eluate was concentrated and the residue was purified by MPLC (1:1, Hex:EtOAc) to give, in order of elution, **25-cis/anti** (4 mg, 0.008 mmol, 6%) as a yellow oil, **25-cis/syn** (50 mg, 0.115 mmol, 79%) as a dark red oil, and the 2:1 adduct **26** (5 mg, 0.065 mmol, 7%) as a yellow oil. Both of the primary products **25-cis/anti** and **25-cis/syn** showed signs of decomposition upon storage and handling, and therefore were characterized soon after their synthesis.

Data for the faster eluting, minor, primary isomer, 25-cis/anti:

^{1}H NMR (500 MHz, CDCl$_3$): 7.17 (s, 1H, Ar*H9*), 6.82 (s, 1H, Ar*H12*), 6.16 (dd, *J* = 9.5, 5.6 Hz, 1H, *H2*), 5.82 (d, *J* = 7.4 Hz, 1H, *H4*), 5.50 (dd, *J* = 9.6, 5.7 Hz, 1H, *H1*), 5.14 (dd, *J* = 7.3, 5.6 Hz, 1H, *H3*), 4.48 (d, *J* = 6.6 Hz, 1H, *H12d*), 4.03 (dd, *J* = 9.7, 5.6 Hz, *H15b*), 3.92 (s, 3H, OC*H*$_3$), 3.87 (s, 3H, OC*H*$_3$), 3.69 (dd, *J* = 9.9, 6.9 Hz, 1H, *H15a*), 3.05 (s, 3H, NC*H*$_3$), 2.38 (s, 3H, ArCH$_3$), and 0.43 [s, 9H, Si(CH$_3$)$_3$].

^{13}C NMR (126 MHz, CDCl$_3$): δ 193.7, 174.7, 174.3, 153.5, 149.7, 146.4, 143.4, 143.1, 138.9, 137.7, 135.4, 135.3, 128.2, 124.8, 118.0, 114.2, 108.0, 106.5, 100.7, 56.5, 56.3, 56.1, 43.0, 41.5, 25.3, 21.0, and 3.2.

HRMS (APCI-Orbitrap): Calculated for $C_{29}H_{31}N_2O_5Si^+$ [M+H$^+$]: 515.1997, found 515.1984.

IR ($CDCl_3$): 2947, 1776, 1701, 1642, 1590, 1568, 1535, 1494, 1462, 1433, 1369, 1314, 1275, 1242, 1210, 1106, 1023, 995, 919, 846, 796, 768, 731, 698, 628, 602, 562, 536, 505, and 464 cm^{-1}.

Data for the faster eluting, major, primary isomer, 25-cis/syn:

^{1}H NMR (500 MHz, $CDCl_3$): 7.20 (s, 1H, Ar*H9*), 7.17 (s, 1H, Ar*H12*), 6.19 (dd, *J* = 9.9, 5.8 Hz, 1H, *H2*), 5.89 (d, *J* = 7.4 Hz, 1H, *H4*), 5.54 (dd, *J* = 9.9, 5.2 Hz, 1H, *H1*), 4.90 (dd, *J* = 7.1, 5.7 Hz, 1H, *H3*), 4.70 (d, *J* = 8.9 Hz, 1H, *H12d*), 4.56 (dd, *J* = 5.4, 5.4 Hz, *H15b*), 3.96 (s, 3H, OCH_3), 3.93 (s, 3H, OCH_3), 3.29 (dd, *J* = 8.8, 5.4 Hz, 1H, *H15a*), 3.03 (s, 3H, NCH_3), 2.31 (s, 3H, $ArCH_3$), and 0.41 [s, 9H, $Si(CH_3)_3$].

^{13}C NMR (126 MHz, $CDCl_3$): 193.3, 175.3, 175.2, 153.8, 149.8, 147.6, 143.9, 142.2, 138.7, 138.4, 136.3, 132.5, 128.4, 124.9, 122.4, 113.9, 107.0, 106.9, 98.1, 56.7, 56.30, 56.28, 51.4, 41.6, 26.0, 20.4, and 2.7.

HRMS (APCI-Orbitrap): Calculated for $C_{29}H_{31}N_2O_5Si^+$ [M+H$^+$]: 515.1997, found 515.1990.

IR (thin film)**:** 2945, 1774, 1695, 1641, 1566, 1543, 1494, 1461, 1433, 1366, 1285, 1246, 1209, 1104, 1046, 1022, 954, 930, 843, 797, 776, 731, 700, 602, 553, and 508 cm^{-1}.

Data for 26 (post-HDDA adduct):

^{1}H NMR (500 MHz, $CDCl_3$): 7.18 (s, 1H, Ar*H9*), 6.86 (s, 1H, Ar*H12*), 6.40 (ddd, *J* = 7.8, 6.0, 1.5 Hz, 1H, *H17* or *H18*), 5.98 (ddd, *J* = 7.7, 6.1, 1.4 Hz, 1H, *H17* or *H18*), 4.53 (d, *J* = 9.3 Hz, 1H, *H12d*), 4.31 (ddd, *J* = 5.8, 4.1, 1.5 Hz, 1H, *H4*), 4.06 (dddd, 1H, *J* = 6.3, 3.2, 1.9, 1.9 Hz, 1H, *H16*), 3.99 (dd, *J* = 9.0, 1.9 Hz, 1H, *15b*), 3.91 (s, 3H, OCH_3), 3.89 (s, 3H, OCH_3), 3.82 (dd, *J* = 7.9, 4.0 Hz, 1H, *H3a*), 3.55 (dd, *J* = 9.1, 9.1 Hz, 1H, *H15a*), 3.11 (dd, *J* = 7.9, 2.9 Hz, 1H, *H16a*), 3.01 (s, 3H, NCH_3), 2.90 (s, 3H, NCH_3), 2.40 (s, 3H, $ArCH_3$), and 0.43 [s, 9H, $Si(CH_3)_3$].

^{13}C NMR (126 MHz, $CDCl_3$): δ 193.1, 177.5, 177.2, 176.0, 175.3, 153.4, 149.7, 148.7, 145.5, 142.7, 138.0, 135.5, 134.8, 133.5, 130.6, 129.0, 118.6, 107.0, 107.0, 56.7, 56.6, 56.3, 52.8, 47.7, 47.5, 41.4, 40.4, 36.3, 25.8, 25.0, 23.7, and 2.6.

HRMS (APCI-Orbitrap): Calculated for $C_{34}H_{36}N_3O_7Si^+$ [M+H$^+$]: 626.2317, found 626.2315.

IR (thin film): 3057, 2944, 2931, 2855, 1776, 1692, 1570, 1541, 1493, 1433, 1378, 1353, 1311, 1290, 1247, 1210, 1176, 1131, 1104, 1032, 968, 915, 842, 778, 763, 730, 700, 677, 646, 614, 595, 514, and 445 cm^{-1}.

(d) Products obtained from mode c: nucleophilic three-component reactions

6,7-Dimethoxy-2-methyl-3-(2-(trichloromethyl)quinolin-1(2H)-yl)-1-(trimethylsilyl)-9H-fluoren-9-one (27)

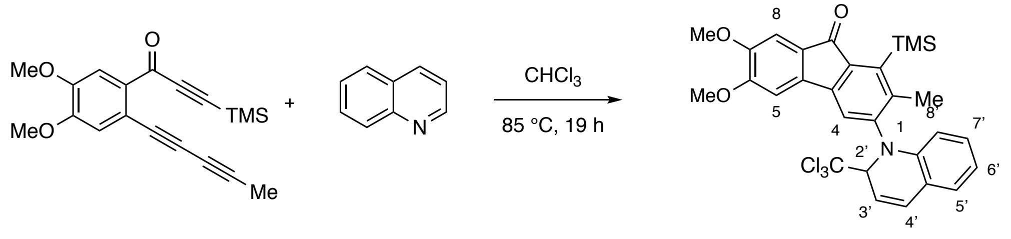

1a ***2b*** ***27*** **(93%)**

Triynone **1a** (25 mg, 0.077 mmol) and quinoline (**2b**, 30 μL, 0.231 mmol, 3 equiv) were combined in a culture tube, dissolved in $CHCl_3$ (6 mL), and sealed with a Teflon-lined cap. The solution was heated overnight (18-19 h) in an oil bath at 85 °C, cooled, and passed through a plug of silica (1:1, Hex:EtOAc). The residue was purified by MPLC (3:1 Hex:EtOAc) to give **27** (41 mg, 93%), as a mixture of atropisomers (1: 0.16 ratio) and yellow oil.

Data for 27 [a mixture of atropisomers in a ratio of 1:0.16]:

^{1}H NMR for the major atropisomer (500 MHz, $CDCl_3$): δ 7.79 (s, 1H, Ar*H4*), 7.17 (s, 1H, Ar*H8*), 7.14 (dd, *J* = 7.5, 1.5 Hz, 1H, Ar*H5'*), 7.06 (ddd, *J* = 9.0, 7.2, 1.7 Hz, 1H, Ar*H7'*), 7.01 (s, 1H, Ar*H5*), 6.96 (d, *J* = 9.7 Hz, C*H4'*), 6.80 (ddd, *J* = 7.4, 7.4, 1.0 Hz, 1H, Ar*H6'*), 6.74 (d, *J* = 8.3 Hz, Ar*H8'*), 6.15 (dd, *J* = 9.8, 5.8 Hz, 1H, C*H3'*), 4.89 (d, *J* = 5.8 Hz, 1H, C*H2'*), 4.05 (s, 3H, C*H3*OC7), 3.93 (s, 3H, C*H3*OC6), 2.93 (s, 3H, ArC*H3*), and 0.41 [s, 9H, Si(C*H3*)3].

^{1}H NMR identifiable resonances for the minor atropisomer (500 MHz, $CDCl_3$): 6.37 (d, *J* = 8.4 Hz, 1H, Ar*H8'*), 6.18 (dd, *J* = 9.7, 5.4 Hz, 1H, C*H3'*), 5.42 (d, *J* = 5.5 Hz, 1H, C*H2'*), 3.96 (s, 3H, C*H3*OC7), 3.91 (s, 3H, C*H3*OC6), 2.58 (s, 3H, ArC*H3*), and 0.48 [s, 9H, Si(C*H3*)3].

^{13}C NMR for the major atropisomer (126 MHz, $CDCl_3$): δ 193.9, 154.9, 149.9, 149.2, 145.1, 142.5, 141.8, 141.7, 139.9, 138.7, 130.7, 129.3, 128.0, 127.2, 126.9, 121.7, 119.5, 116.3, 115.0, 107.0, 105.5 (CCl_3), 103.1, 74.9, 56.7, 56.4, 19.4, and 2.9.

IR (thin film): 3054, 3002, 2941, 2837, 1703, 1645, 1588, 1487, 1455, 1367, 1315, 1291, 1243, 1213, 1173, 1151, 1106, 1070, 1016, 991, 921, 845, 830, 799, 771, 736, 717, 701, 678, 639, 605, 528, 504, and 426 cm^{-1}.

HRMS (APCI-Orbitrap): Calculated for $C_{28}H_{28}NO_3Si^+$ [M–CCl_3^-]$^+$: 454.1833, found 454.1817.

Reverse phase liquid chromatography: Only one peak corresponding to **27** was observed, consistent with the assumption that the atropisomers are interconverting sufficiently rapidly to coeluted.

2-Methyl-3-(1-(trichloromethyl)isoquinolin-2(1H)-yl)-1-(trimethylsilyl)-9H-fluoren-9-one (28)

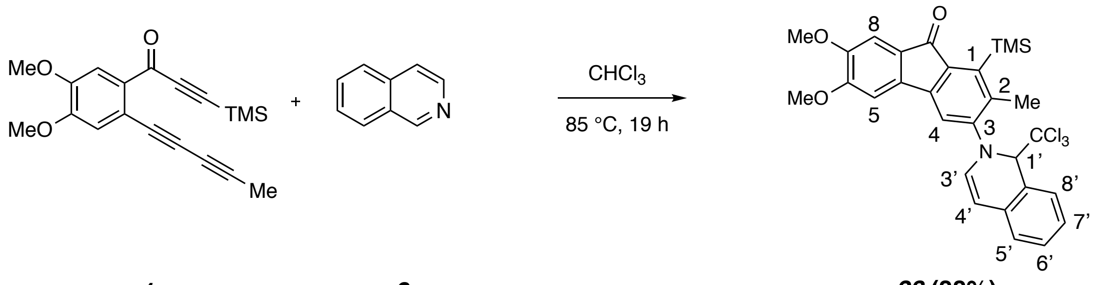

1a ***2a*** ***28* (88%)**

Triynone **1a** (30 mg, 0.093 mmol) and isoquinoline (**2a**, 32.7 μL, 0.277 mmol, 3 equiv) were added to a culture tube, dissolved in $CHCl_3$ (6 mL), and sealed with a Teflon-lined cap. The solution was heated overnight (18-19 h) in an oil bath at 85 °C, cooled, and passed through a plug of silica (EtOAc elution). The residue was purified by MPLC (3:1 Hex:EtOAc) to give **28** (46.6 mg, 88%) as a yellow oil.

^{1}H NMR (500 MHz, C_6D_6, **343K**): δ 7.40 (br s, 1H, Ar*H4*), 7.34 (dd, *J* = 7.5, 0.5 Hz, 1H, Ar*H8'*), 7.18 (ddd, *J* = 7.5, 7.5, 1.5 Hz, 1H, Ar*H6'*), 7.13 (s, 1H, Ar*H8*), 7.08 (ddd, *J* = 7.5, 7.5, 1.5 Hz, 1H, Ar*H7'*), 7.03 (dd, *J* = 8.0, 1.5 Hz, 1H, Ar*H5'*), 6.66 (s, 1H, Ar*H5*), 6.40 (dd, *J* = 7.5, 1.0 Hz, 1H, C*H3'*), 5.72 (dd, *J* = 7.5, 0.5 Hz, 1H, C*H4'*), 5.63 (br s, 1H, *CH1'*), 3.35 (s, 3H, C6OCH_3), 3.32 (s, 3H, C5OCH_3), 2.31 (s, 3H, ArCH_3), and 0.58 [s, 9H, Si(CH_3)$_3$].

A differential nOe experiment showed enhancement of the *H*5, *H*1', and *H3'* upon irradiation of Ar*H*4, and C6-methoxy and *H*4 upon irradiation of *H5* for allowing the regioisomeric assignment of the shown **28**.

^{13}C NMR (126 MHz, $CDCl_3$): δ 193.6, 154.6, 152.4, 149.9, 145.0, 143.8, 138.27 (br), 138.20, 138.18, 134.1, 133.9, 131.2, 129.6, 127.1, 125.4, 124.2, 121.4, ca. 119 (v br), 106.9, 105.3 (x), 103.3, 103.0, 76.9, 56.6, 56.3, 21.1, and 2.9.

IR (neat): 2950, 1711, 1625, 1420, 1391, 1298, 1274, 1247, 1006, 968, 945, 934, 856, 846, 822, and 768 cm^{-1}.

HRMS (ESI-TOF): Calculated for $C_{28}H_{28}NO_3Si^+$ [M–CCl_3^-]$^+$ 454.1833, found 454.1849.

TLC: R_f 0.3 (3:1 Hex/EtOAc).

6,7-Dimethoxy-2-methyl-3-(4-(trichloromethyl)pyridin-1(4H)-yl)-1-(trimethylsilyl)-9H-fluoren-9-one (29):

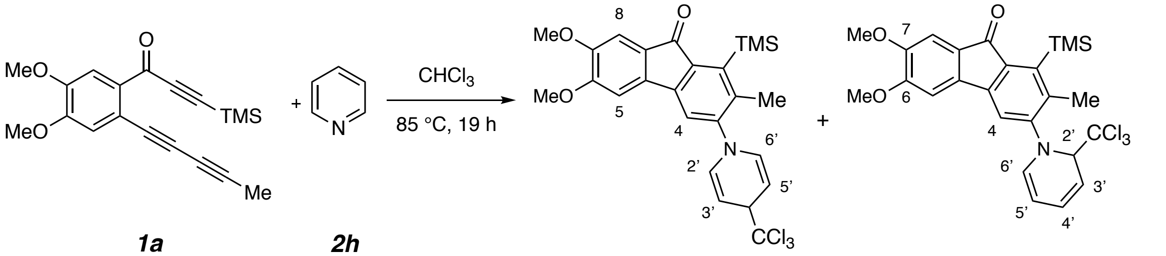

1a ***2h*** ***29*** *(84% yield)* ***S7***

Triynone **1a** (40 mg, 0.123 mmol) and pyridine (**2h**, 30 μL, 0.370 mmol, 3 equiv) were combined in a culture tube, dissolved in $CHCl_3$ (10 mL), and sealed with a Teflon-lined cap. The solution was heated overnight (18-19 h) in an oil bath at 85 °C, cooled, and passed through a plug of silica (1:1, Hex:EtOAc). The residue was purified by MPLC (6:1 Hex:EtOAc) to give a 10:1 mixture of isomers (60 mg, 94% combined yield) as a yellow oil. A small portion of the mixture was separately repurified by normal phase HPLC (6:1, Hex:EtOAc) to give, in order of elution, **29** (major isomer) as an orange oil, and the minor isomer **S7**, also as a transparent oil. The latter was contaminated with ca. 15% of **29**. The broad resonances observed in the ^{1}H NMR spectrum were an indicator of rotamer issues; not surprisingly, the ^{13}C data were of marginal quality and gave very limited useful information.

Data for the major regioisomer, 29: ^{1}H NMR (500 MHz, $CDCl_3$): δ 7.14 (s, 1H, Ar*H8*), 7.09 (s, 1H, Ar*H5* or Ar*H4*), 6.93 (s, 1H, Ar*H5* or Ar*H4*), 6.41 (d, *J* = 7.9 Hz, 2H, NC*H*CH), 5.05 (dd, *J* = 8.1, 4.5 Hz, 2H, NCHC*H*), 4.17 (t, *J* = 4.3 Hz, 1H, Cl_3CC*H*), 4.01 (s, 3H, CH_3O), 3.91 (s, 3H, CH_3O'), 2.32 (s, 3H, ArC*H3*), and 0.43 [s, 9H, Si(CH_3)$_3$].

^{13}C NMR for the major regioisomer, 29 (100 MHz, $CDCl_3$): δ 193.7, 154.7, 150.0, 147.6, 144.9, 143.8, 139.0, 138.6, 138.1, 133.4 (br), 127.1, 116.8 (br), 107.0, 102.9, 97.4 (br), 56.6, 56.4, 55.4 (br), 20.4, and 2.7. (one resonance was not observed)

IR (thin film): 3111, 3065, 3000, 2943, 2900, 2837, 1706, 1673, 1589, 1495, 1456, 1444, 1383, 1359, 1317, 1245, 1213, 1153, 1102, 1046, 1015, 996, 911, 843, 769, 728, 699, 601, 536, 514, 483, and 415 cm^{-1}.

HRMS (APCI-Orbitrap): Calculated for $C_{25}H_{27}Cl_3NO_3Si^+$ $[M+H^+]^+$: 522.0820, found 522.0781, Calculated for $C_{24}H_{26}NO_3Si^+$ $[M–CCl_3^-]^+$: 404.1676, found 404.1660 (minor ion).

Data for the minor isomer S-7 [contains coeluting major regioisomer 29 (15%)]:

^{1}H NMR for the minor regioisomer, 4aak-C2 (500 MHz, $CDCl_3$): δ ca. 7.4–7.0 (v br, ca. 1H, Ar*H4*), 7.13 (s, 1H, Ar*H8*), 6.96 (s, 1H, Ar*H5*), 6.49 (dd, *J* = 9.4, 6.0 Hz, 1H, *H4'*), 6.38 (br s, 1H, *H6'*), 5.73 (br s, 1H, *H3'*), 5.22 (ddd, J = 7.2, 6.0, 1.2 Hz, 1H, *H5'*), 5.5 and 4.9 (v br s, not yet coalesced, 1H, *H2'*), 4.03 (s, 3H, CH_3O), 3.91 (s, 3H, CH_3O), 2.44 (br s, 3H, ArC*H3*), and 0.44 [s, 9H, Si(CH_3)$_3$].

HRMS (APCI-Orbitrap): Calculated for $C_{24}H_{26}NO_3Si^+$ [M–CCl_3^-]$^+$: 404.1676, found 404.1675

IR (CH_2Cl_2): 3061, 2931, 2853, 1705, 1588, 1495, 1463, 1382, 1358, 1316, 1246, 1214, 1153, 1102, 1017, 845, 769, 734, 700, 603, and 415 cm^{-1}.

6,7-Dimethoxy-2-methyl-3-(9-(trichloromethyl)acridin-10(9*H*)-yl)-1-(trimethylsilyl)-9*H*-fluoren-9-one (30):

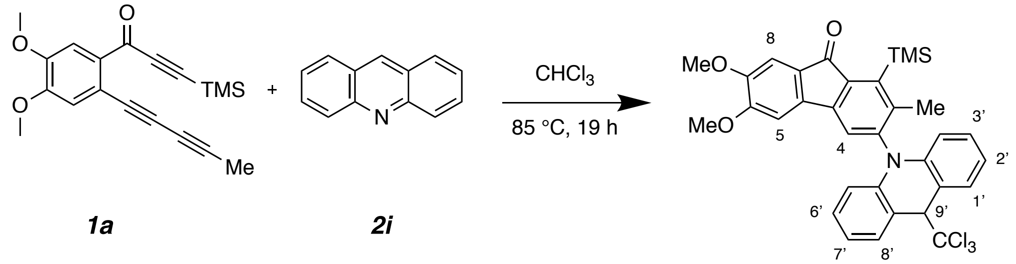

1a ***2i*** ***30*** **(82%, ratio: 1:0.5)**

Triynone **1a** (15 mg, 0.046 mmol) and acridine (**2i**, 25 mg, 0.092 mmol, 2 equiv) were dissolved in benzene (4 mL, 0.01M), paced into a threaded vial, and sealed with a Teflon-lined screw-cap. The solution was heated overnight (18-19 h) in an oil bath at 85 °C, cooled, and passed through a plug of silica (2:1, Hex:EtOAc). The residue was purified by MPLC (3:1, Hex:EtOAc) to give **30** (23 mg) as a yellow oil.

Data for 30 [a mixture of atropisomers in a ratio of 1 :0.38]:

^{1}H NMR for the major atropisomer (500 MHz, CDCl$_3$): 7.65 (dd, *J* = 7.7, 1.0 Hz, 2H, Ar*H1'* and Ar*H8'*), 7.26 (ddd, *J* = 8.7, 7.2, 1.7 Hz, 2H, Ar*H3'* and Ar*H6'*), 7.181 (s, 1H, Ar*H5* or Ar*H8*), 7.177 (s, 1H, Ar*H5* or Ar*H8*), 7.06 (ddd, *J* = 7.6, 7.6, 1.2 Hz, 2H, Ar*H2'* and Ar*H7'*), 6.80 (s, 1H, Ar*H4*), 6.46 (dd, *J* = 8.4, 1.0 Hz, 2H, Ar*H4'* and Ar*H5'*), 5.06 (s, 1H, *H9'*), 3.93 (s, 3H, OCH_3), 3.93 (s, 3H, OCH_3), 1.87 (s, 3H, ArCH$_3$), and 0.480 [s, 9H, Si(CH$_3$)$_3$].

^{1}H NMR identifiable resonances for the minor atropisomer (500 MHz, CDCl$_3$): 7.70 (dd, *J* = 7.8, 1.3 Hz, 2H, Ar*H1'* and Ar*H8'*), 7.25 (ddd, *J* = 8, 7.4, 1.5 Hz, 2H, Ar*H3'* and Ar*H6'*), 7.18 (s, 1H, Ar*H5* or Ar*H8*), 7.08 (ddd, *J* = 7.3, 7.3, 1.2 Hz, 2H, Ar*H2'* and Ar*H7'*), 6.91 (s, 1H, Ar*H5* or Ar*H8*), 6.76 (s, 1H, Ar*H4*), 6.49 (dd, *J* = 8.4, 1.0 Hz, 2H, Ar*H4'* and Ar*H5'*), 5.14 (s, 1H, *H9'*), 3.92 (s, 3H, OCH_3), 3.86 (s, 3H, OCH_3), 2.33 (s, 3H, ArCH$_3$), and 0.484 [s, 9H, Si(CH$_3$)$_3$].

^{13}C NMR for the major atropisomer (126 MHz, CDCl$_3$): δ 194.0, 154.9, 150.1, 145.3, 144.72, 144.2, 142.4, 142.2, 141.0, 138.30, 133.3, 129.6, 126.7, 122.0, 120.5, 115.8, 113.9, 107.01, 105.8 (CCl$_3$), 103.0, 62.18, 56.6, 56.37, 19.18, and 3.0.

^{13}C NMR for the minor atropisomer (126 MHz, CDCl$_3$): δ 193.9, 154.8, 150.0, 145.1, 144.8, 144.70, 144.0, 141.3, 140.0, 138.31, 133.2, 129.4, 126.8, 122.4, 120.6, 115.7, 114.6, 106.97, 103.1, 62.17, 56.43, 56.39, 19.19, and 2.9. (CCl$_3$ resonance not observed)

HRMS (APCI-Orbitrap): Calculated for $C_{32}H_{30}NO_3Si^+$ [M–CCl$_3^-$]$^+$: 504.1989, found 504.1993.

IR (thin film): 3072, 3002, 2928, 2903, 2839, 1708, 1590, 1494, 1476, 1459, 1374, 1359, 1341, 1311, 1264, 1243, 1216, 1169, 1149, 1130, 1079, 1051, 1016, 990, 929, 910, 843, 783, 751, 731, 672, 635, 617, 603, 547, 509, 464, and 422 cm^{-1}.

6,7-Dimethoxy-2-methyl-3-(1-(trichloromethyl)phthalazin-2(1*H*)-yl)-1-(trimethylsilyl)-9*H*-fluoren-9-one-4-*d* (31):

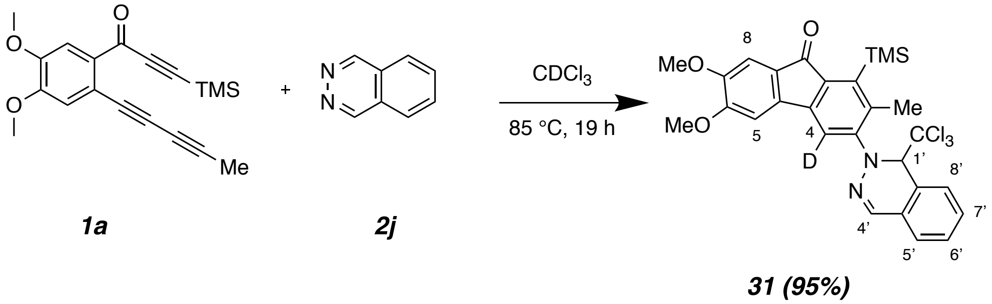

1a ***2j*** ***31 (95%)***

Triynone **1a** (20 mg, 0.062 mmol) and phthalazine (**2j**, 16 mg, 0.124 mmol, 2 equiv) were dissolved in benzene (4 mL, 0.02 M), and sealed with a Teflon-lined screw-cap. The solution was heated overnight (18-19 h) in an oil bath at 85 °C, cooled, and passed through a plug of silica (2:1, Hex:EtOAc). The residue was purified by MPLC (2:1, Hex:EtOAc) to give **31** (0.059 mmol, 95%) as a yellow oil.

Data for 31:

^{1}H NMR (500 MHz, CDCl$_3$): 7.69 (s, 1H, Ar*H4'*), 7.64 (nfom, 1H, Ar*H5'* or Ar*H8'*), 7.60 (nfom, 2H, Ar*H6'* and Ar*H7'*), 7.42 (nfom, 1H, Ar*H5'* or Ar*H8'*), 7.14 (s, 1H, Ar*H8*), 7.01 (s, 1H, Ar*H5*), 5.76 (s, 1H, Ar*H1'*), 4.02 (s, 3H, OCH_3), 3.91 (s, 3H, OCH_3), 2.35 (s, 3H, ArCH$_3$), and 0.44 [s, 9H, Si(CH$_3$)$_3$].

^{13}C NMR (125 MHz, CDCl$_3$): 193.8, 154.5, 152.4, 149.8, 144.7, 143.4, 138.5, 138.0, 137.6, 135.5, 130.6, 130.33, 130.30, 127.3, 126.4, 124.9, 123.1, 106.8, 103.6, 103.3, 72.9 (C1'), 56.6, 56.3, 21.3, and 2.9. (one carbon resonance was not identified).

HRMS (ESI-TOF): Calculated for $C_{28}H_{27}DCl_3N_2O_3Si^+$ [M+H$^+$]$^+$: 574.0992, found 574.0984.

IR (thin film): 3066, 2999, 2936, 2901, 2852, 2837, 1701, 1598, 1578, 1492, 1453, 1406, 1365, 1315, 1266, 1243, 1214, 1128, 1095, 1048, 1014, 963, 922, 847, 829, 780, 754, 734, 701, 674, 647, 635, 612, 600, 584, 536, 470, and 422 cm^{-1}.

4-Chloro-6,7-dimethoxy-2-methyl-3-(2-(trichloromethyl)quinolin-1(2H)-yl)-1-(trimethylsilyl)-9H-fluoren-9-one (32):

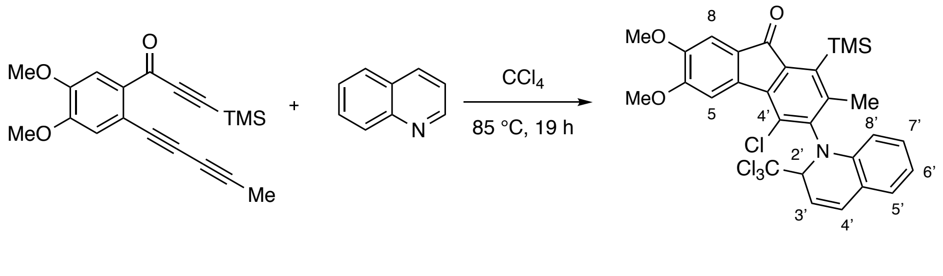

1a ***2b*** ***32* (56%)**
Ratio of atropisomers: 1:0.5

Triynone **1a** (20 mg, 0.062 mmol) and quinoline (**2b**, 14.7 μL, 0.124 mmol, 2 equiv) were combined in a culture tube, dissolved in CCl_4 (2 mL), and sealed with a Teflon-lined screw cap. The solution was heated overnight (18-19 h) in an oil bath at 85 °C, cooled, and passed through a plug of silica (1:1, Hex:EtOAc). The residue was purified by MPLC (3:1 Hex:EtOAc) to give **32** (21 mg, 0.035 mmol, 56%) as an orange oil. A small portion of this product was separately repurified by HPLC (1:1, Hex:EtOAc) to give sample of higher purity for characterization purposes. The ^{1}H NMR spectrum indicated that this compound was a 2.3:1 ratio of diastereomeric atropisomers.

Data for 32:

^{1}H NMR for the major atropisomer (500 MHz, $CDCl_3$): δ 7.72 (s, 1H, Ar*H5*), 7.68 (dd, *J* = 7.8, 1.4 Hz, 1H, ArH5'), 7.20 (s, 1H, Ar*H8*), 7.20 (1H, ddd, *J* = 8, 7.3, 1.8 Hz, 1H, Ar*H7'*), 7.05 (ddd, *J* =7.8, 7.8, 1.3 Hz, 1H, Ar*H6'*), 6.39 (d, *J* = 8.0 Hz, Ar*H8'*), 6.39 (d, *J* = 8.0 Hz, Ar*H4'*), 5.24 (dd, *J* = 8.0, 5.2 Hz, 1H, *H3'*), 4.68 (d, *J* = 5.1 Hz, 1H, *H2'*), 3.98 (s, 3H, OCH_3), 3.94 (s, 3H, OCH_3'), 2.04 (s, 3H, ArCH_3), and 0.43 [s, 9H, Si$(CH_3)_3$].

^{1}H NMR identifiable resonances for the minor atropisomer (500 MHz, $CDCl_3$): δ 7.87 (s, 1H, Ar*H5*), 7.21 (s, 1H, Ar*H8*), 7.14 (dd, *J* = 7.5, 1.5 Hz, 1H, ArH5'), 7.09 (ddd, *J* = 8.8, 7.3, 1.9 Hz, 1H, Ar*H7'*), 6.93 (d, *J* = 9.9 Hz, Ar*H4'*), 6.83 (dd, *J* = 7.4, 7.4, 0.9 Hz, Ar*H6'*), 6.54 (dd, *J* = 8.4, 0.7 Hz, 1H, *H8'*), 6.13 (dd, *J* = 9.8, 5.4 Hz, 1H, *H3'*), 4.98 (dd, *J* = 5.5, 0.9 Hz, 1H, *H2'*), 4.04 (s, 3H, OCH_3), 3.95 (s, 3H, OCH_3'), 2.22 (s, 3H, ArCH_3), and 0.42 [s, 9H, Si$(CH_3)_3$].

^{13}C NMR for the major atropisomer (125 MHz, $CDCl_3$): δ 192.9, 154.5, 150.0, 145.0, 142.6, 142.1, 140.1, 140.0, 137.9, 134.3, 133.0, 130.8, 129.2, 128.1, 127.0, 121.7, 119.8, 113.3, 107.2, 106.9, 106.0, 95.2, 58.4, 56.5, 56.3, 20.3, and 2.9.

HRMS (APCI-Orbitrap): Calculated for $C_{29}H_{28}Cl_4NO_3Si^+$ $[M+H^+]^+$: 606.0587, found 606.0561.

IR (thin film): 3057, 3003, 2928, 2901, 2837, 1707, 1655, 1588, 1489, 1458, 1394, 1359, 1337, 1312, 1246, 1214, 1119, 1106, 1074, 1019, 996, 949, 934, 886, 845, 770, 732, 628, 605, 575, 530 515, 493, 436, and 409 cm^{-1}.

1-(5-Methyl-1-(methylsulfonyl)-4-(prop-1-yn-1-yl)indolin-6-yl)-2-(trichloromethyl)-1,2-dihydroquinoline (33):

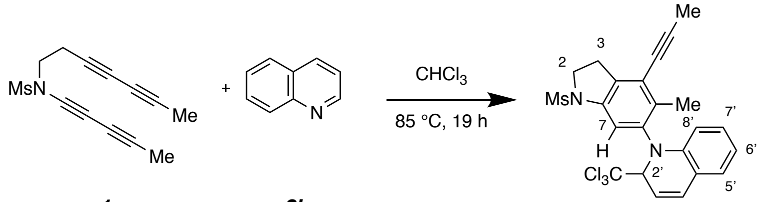

1c ***2b***

***33* (92%)**
Ratio of atropisomers: 1:0.33

Tetrayne **1c** (25 mg, 0.101 mmol, 1 equiv) and quinoline (**2b**, 42 μL, 0.303 mmol, 3 equiv) were combined in a culture tube, dissolved in chloroform (6 mL, 0.02M), and sealed with a Teflon-lined cap. The solution was heated overnight (18-19 h) in an oil bath at 85 °C, cooled, and passed through a plug of silica (1:1, Hex:EtOAc). The residue was purified by MPLC (2:1, Hex:EtOAc) to give a mixture of atropisomers **33** (46 mg, 92% overall, 1:0.33 ratio) as a flaky white amorphous solid.

Data for 33 [a mixture of atropisomers in a ratio of 1:0.33]:

^{1}H NMR for the major atropisomer (400 MHz, $CDCl_3$): δ 7.88 (s, 1H, Ar*H7*), 7.10 (dd, *J* = 7.4, 1.7 Hz, 1H, Ar*H5'*), 7.01 (ddd, *J* = 8.5, 7.4, 1.6 Hz, 1H, Ar*H7'*), 6.95 (ddd, *J* = 9.7, 1.0, 1.0 Hz, 1H, Ar*H4'*), 6.77 (ddd, *J* = 7.4, 7.4, 1.0 Hz, 1H, Ar*H6'*), 6.60 (d, *J* = 8.2 Hz, 1H, Ar*H8'*), 6.13 (dd, *J* = 9.7, 5.7 Hz, 1H, *H3'*), 4.87 (dd, *J* = 5.8, 0.9 Hz, 1H, *H2'*), 4.14–4.04 (overlapping m, 1H, $CH_3SO_2NCH_aH_bCH_2$), 4.03–3.91 (overlapping m, 1H, $CH_3SO_2NCH_aH_bCH_2$), 3.21 (t, *J* = 8.8 Hz, 2H, $NMsCH_2CH_2$), 2.89 (s, 3H, CH_3SO_2N), 2.10 (s, 3H, $NArCH_3$), and 2.05 (s, 3H, $C{\equiv}CCH_3$).

^{1}H NMR identifiable resonances for the minor atropisomer (400 MHz, $CDCl_3$): δ 6.89 (ddd, *J* = 9.9, 1.0, 1.0 Hz, 1H, Ar*H4'*), 6.27 (d, *J* = 8.3 Hz, 1H, Ar*H8'*), 6.10 (dd, *J* = 9.8, 5.4 Hz, 1H, *H3'*), 5.38 (dd, *J* = 5.4, 1.0 Hz, 1H, *H2'*), 4.14–4.04 (overlapping m, 1H, $CH_3SO_2NCH_aH_bCH_2$), 4.03–3.91 (overlapping m, 1H, $CH_3SO_2NCH_aH_bCH_2$), 3.22 (t, *J* = 8.4 Hz, 2H, $NMsCH_2CH_2$), 2.82 (s, 3H, CH_3SO_2N), 2.47 (s, 3H, $NArCH_3$), and 2.17 (s, 3H, $C{\equiv}CCH_3$).

^{13}C NMR for the major atropisomer (126 MHz, $CDCl_3$): δ 145.6, 142.9, 139.3, 134.0, 133.8, 130.6, 129.3, 127.6, 120.7, 119.4, 116.4, 116.0, 114.4, 112.8, 105.4 (CCl_3), 95.2, 75.8, 74.9, 50.6, 34.9, 28.4, 15.4, and 4.7.

HRMS (APCI-Orbitrap): Calculated for $C_{22}H_{21}N_2O_2S^+$ $[M{-}CCl_3^-]^+$: 377.1318, found 377.1318.

IR (thin film): 3055, 3024, 2916, 2850, 2232, 1737, 1643, 1593, 1486, 1453, 1346, 1287, 1254, 1154, 1111, 1066, 1000, 968, 893, 829, 801, 774, 751, 717, 663, 629, 607, 568, 545, 514, and 441 cm^{-1}.

Reverse phase liquid chromatography: Only one peak corresponding to **33** was observed, consistent with the assumption that the atropisomers are interconverting sufficiently rapidly to coeluted.

2-(6-Methyl-7-(prop-1-yn-1-yl)-1,3-dihydroisobenzofuran-4-yl)-1-(trichloromethyl)-1,2-dihydrophthalazine (34-A) and

2-(6-Methyl-7-(prop-1-yn-1-yl)-1,3-dihydroisobenzofuran-5-yl)-1-(trichloromethyl)-1,2-dihydrophthalazine (34-B)

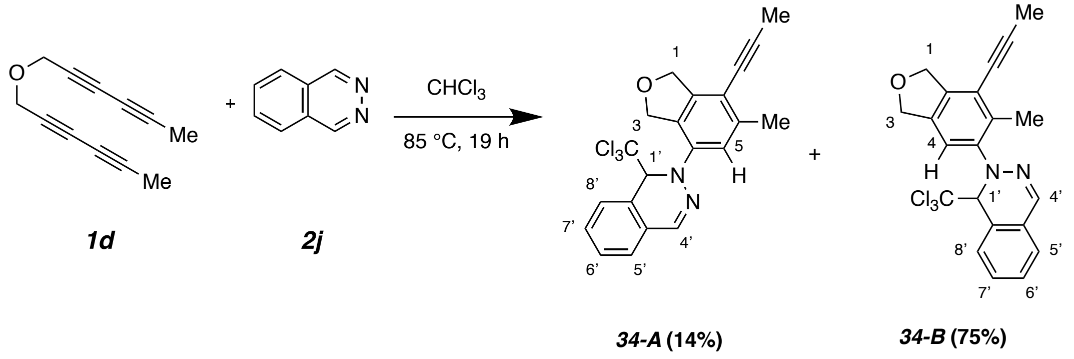

Tetrayne **1d** (20 mg, 0.118 mmol) and phthalazine (**2j**, 45.9 mg, 0.353 mmol, 3 equiv) were combined in a culture tube, dissolved in chloroform (4 mL, 0.03M), and sealed in a vial with a Teflon-lined screw-cap. The solution was heated overnight (18-19 h) in an oil bath at 85 °C, cooled, and passed through a plug of silica (2:1, Hex:EtOAc). The residue was purified by MPLC (6:1, Hex:EtOAc) to give, in order of elution, **34-A** (7 mg, 0.016 mmol, 14%) as a pale yellow oil, and (37 mg, 0.088 mmol, 75%), also as a yellow oil. The assignment of the structure of both isomers was based upon observed nOe interactions (difference nOe) between the aromatic proton with benzylic methyl or methylene protons, respectively.

Data for faster eluting, minor regioisomer, 34-A:

^{1}H NMR (500 MHz, CDCl$_3$): δ 7.64 (s, 1H, Ar*H4'*), 7.63 (nfom, 1H, Ar*H5'* or Ar*H8'*), 7.55 (m, 2H, Ar*H6'* and Ar*H7'*), 7.37 (nfom, 1H, Ar*H5'* or Ar*H8'*), 6.94 (s, 1H, Ar*H5*), 6.14 (s, 1H, *H1'*), 5.44 (br d, *J* = 12.9 Hz, 1H, O*C3H$_a$*H$_b$), 5.30 (br d, *J* = 12.9 Hz, 1H, OC3H$_a$*H$_b$*), 5.15 (br d, *J* = 13.6 Hz, 1H, C1*H$_a$*H$_b$), 5.11 (br d, *J* = 13.1 Hz, 1H, C1H$_a$*H$_b$*), 2.41 (s, 3H, NArC*H$_3$*), and 2.10 (s, 3H, C≡CC*H$_3$*).

^{13}C NMR (126 MHz, CDCl$_3$) δ 144.9, 142.1, 140.3, 137.9, 130.32, 130.28, 130.2, 128.0, 126.5, 124.7, 123.4, 116.4, 111.8, 103.3, 92.6, 75.9, 75.6, 74.1, 69.8, 20.5, and 4.7.

HRMS (APCI-Orbitrap): Calculated for $C_{21}H_{18}Cl_3N_2O^+$ [M+H$^+$]: 419.0479, found 419.0477.

IR (thin film): 3068, 3036, 2917, 2852, 2253, 1603, 1490, 1453, 1414, 1373, 1318, 1288, 1247, 1224, 1172, 1130, 1108, 1053, 929, 906, 853, 833, 809, 783, 759, 734, 649, 612, 582, 521, and 419 cm^{-1}.

Data for slower eluting, major regioisomer 34-B:

^{1}H NMR (500 MHz, $CDCl_3$): δ 7.60 (s, 1H, Ar*H4'*), 7.58–7.52 [m, 3H, 1H, Ar*H5'* (or Ar*H8'*), Ar*H6',* and Ar*H7'*), 7.37 [nfom, 1H, Ar*H8'* (or Ar*H5'*)], 7.26 (s, 1H, Ar*H4*), 5.80 (s, 1H, *H1'*), 5.12 (br s, 2H, C1H_2), 5.10 (br s, 2H, C3H_2), 2.44 (s, 3H, NArCH_3), and 2.11 (s, 3H, C≡CCH_3).

^{13}C NMR (126 MHz, $CDCl_3$): δ 148.0, 139.5, 137.3, 136.4, 133.8, 130.6, 130.1, 130.0, 126.5, 124.6, 122.6, 119.2, 118.8, 103.9, 94.2, 76.1, 74.2, 74.1, 73.3, 17.0, and 4.7.

IR (thin film): 2954, 2922, 2852, 2227, 1681, 1589, 1555, 1453, 1371, 1303, 1254, 1128, 1101, 1054, 961 923, 902, 864, 829, 781, 761, 735, 706, 650, 612, 583, 564, and 424 cm^{-1}.

HRMS (APCI-Orbitrap): Calculated for $C_{21}H_{18}Cl_3N_2O^+$ $[M+H^+]^+$: 419.0479, found 419.0481.

2-(6-Methyl-2-(methylsulfonyl)-7-(prop-1-yn-1-yl)isoindolin-4-yl)-1-(trichloromethyl)-1,2-dihydrophthalazine (35-A) and

2-(6-Methyl-2-(methylsulfonyl)-7-(prop-1-yn-1-yl)isoindolin-5-yl)-1-(trichloromethyl)-1,2-dihydrophthalazine (35-B):

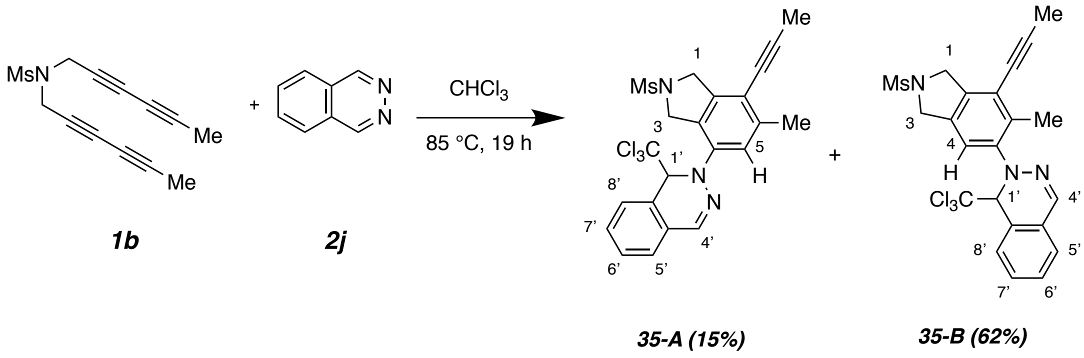

Tetrayne **1b** (40 mg, 0.162 mmol) and phthalazine (**2j**, 42 mg, 0.323 mmol, 2 equiv) were combined in a culture tube, dissolved in chloroform (6 mL, 0.02M), and sealed in a vial with a Teflon-lined screw-cap. The solution was heated overnight (18-19 h) in an oil bath at 85 °C, cooled, and passed through a plug of silica (2:1, Hex:EtOAc). The residue was purified by MPLC (2:1, Hex:EtOAc) to give, in order of elution, **35-A** (12 mg, 0.024 mmol, 15%) as a white crystalline solid, and **35-B** (50 mg, 0.101, 62%), as a transparent oil, which turned into a white crystalline solid after being subjected to high vacuum. The assignment of the structure of both isomers was based upon observed nOe interactions (difference nOe) between the aromatic proton with benzylic methyl or methylene protons, respectively.

Data for faster eluting, minor isomer: 35-A:

^{1}H NMR (500 MHz, CDCl$_3$): 7.63 (nfom, 1H, Ar*H5'* or Ar*H8'*), 7.63 (s, 1H, Ar*H4'*), 7.57 (m, 2H, Ar*H6'* and Ar*H7'*), 7.38 (nfom, 1H, Ar*H5'* or Ar*H8'*), 6.92 (s, 1H, Ar*H5*), 5.79 (s, 1H, *H1'*), 5.12 (br d, *J* = 14.8 Hz, 1H, MsN*C3H$_a$*C3H$_b$), 4.87 (dd, *J* = 14.8, 2.2 Hz, 1H, MsNC3H$_a$*C3H$_b$*), 4.76 (dd, *J* = 14.4, 2.9 Hz, 1H, MsN*C1H$_a$*C1H$_b$), 4.70 (br d, *J* = 14.4 Hz, 1H, MsNC1H$_a$C1*H$_b$*), 2.88 (s, 3H, C*H*$_3$SO$_2$N), 2.40 (s, 3H, NArC*H$_3$*), and 2.11 (s, 3H, C≡CC*H$_3$*).

^{13}C NMR (125 MHz, CDCl$_3$): 143.0, 141.8, 140.9, 137.8, 130.42, 130.39, 130.35, 126.4, 126.2, 124.9, 123.3, 117.6, 113.8, 103.4, 94.0, 75.4, 70.3, 55.6, 54.5, 34.4, 20.6, and 4.7.

HRMS (ESI-TOF): Calculated for $C_{21}H_{20}N_3O_2S^+$ $[M–CCl_3^-]^+$: 378.1271, found 378.1262.

IR (thin film): 3051, 2919, 2853, 2232, 1603, 1489, 1452, 1418, 1375, 1320, 1265, 1222, 1151, 1129, 1107, 1061, 959, 927, 912, 862, 832, 809, 783, 755, 731, 702, 649, 612, 581, 554, 518, 495, 449, and 418 cm^{-1}.

mp: 225–228 °C.

Data for slower eluting, major isomer, 35-B:

^{1}H NMR (500 MHz, $CDCl_3$): 7.61 (s, 1H, Ar*H4'*), 7.59 (nfom, 1H, Ar*H5'* or Ar*H8'*), 7.56 (m, 2H, Ar*H6'* and Ar*H7'*), 7.38 (nfom, 1H, Ar*H5'* or Ar*H8'*), 7.28 (s, 1H, Ar*H4*), 5.79 (s, 1H, *H1'*), 4.72–4.66 (m, 4H, MsNC3H_2 and MsNC1H_2), 2.87 (s, 3H, CH_3SO_2N), 2.45 (s, 3H, NArCH_3), and 2.13 (s, 3H, C≡CCH_3).

^{13}C NMR (125 MHz, $CDCl_3$): 148.6, 136.8, 136.4, 134.5, 134.1, 130.6, 130.2, 130.2, 126.4, 124.7, 122.6, 121.0, 120.1, 103.8, 95.5, 75.7, 73.1, 54.3, 54.3, 34.9, 17.2, and 4.7.

HRMS (ESI-TOF): Calculated for $C_{22}H_{21}Cl_3N_3O_2S^+$ $[M+H^+]^+$: 496.0415, found 496.0403.

IR (thin film): 3051, 2918, 2853, 2227, 1739, 1602, 1589, 1461, 1372, 1334, 1265, 1150, 1082, 1002, 959, 921, 865, 828, 781, 755, 731, 702, 652, 612, 598, 582, 556, 519, 506, 488, and 422 cm^{-1}.

mp: 171–174 °C.

6,7-Dimethoxy-2-methyl-3-(2-(phenylethynyl)quinolin-1(2*H*)-yl)-1-(trimethylsilyl)-9*H*-fluoren-9-one (36):

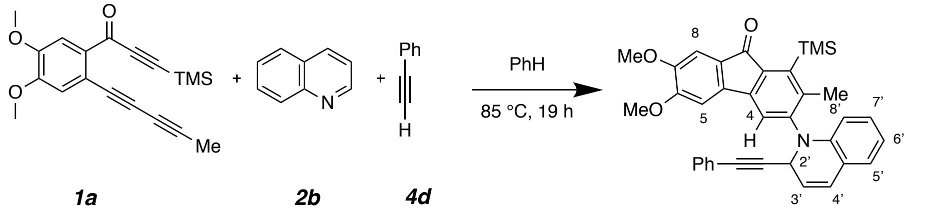

Triynone **1a** (30 mg, 0.092 mmol, 1 equiv), quinoline (**2b**, 22 μL, 0.184 mmol, 2 equiv), and ethynylbenzene (**4d**, 48 µL, 0.462 mmol, 5 equiv) were combined in a culture tube, dissolved in benzene (6 mL, 0.02M), and sealed with a Teflon-lined screw-cap. The solution was heated overnight (18-19 h) in an oil bath at 85 °C, cooled, and passed through a plug of silica (1:1, Hex:EtOAc). The residue was purified by MPLC (6:1, Hex:EtOAc) to give **36** (28 mg, 54% overall, 1:0.3 ratio) as a yellow oil, which turned into a flaky amorphous solid after being subjected to high vacuum.

Data for 36 [a mixture of atropisomers in a ratio of 1 :0.3]:

^{1}H NMR for the major atropisomer (500 MHz, $CDCl_3$): 7.89 (s, 1H, Ar*H4*), 7.37 (dd, *J* = 7.4, 1.5 Hz, 2H, PhH_o), 7.23 (m, 3H, PhH_m and PhH_p), 7.15 (s, 1H, Ar*H8*), 7.07 (dd, *J* = 7.4, 1.6 Hz, 1H, Ar*H5*'), 7.00 (ddd, *J* = 8.1, 7.4, 1.7 Hz, 1H, Ar*H7'*), 6.78 (s, 1H, Ar*H5*), 6.74 (ddd, *J* =7.3, 7.3, 0.9 Hz, 1H, Ar*H6'*), 6.58 (d, *J* = 9.6 Hz, 1H, *H4*'), 6.28 (d, *J* = 8.2 Hz, 1H, Ar*H8*'), 5.89 (dd, *J* = 9.5, 5.7 Hz, 1H, *H3'*), 5.21 (dd, *J* = 5.7, 0.8 Hz, 1H, *H2'*), 3.91 (s, 3H, OCH_3), 3.79 (s, 3H, OCH_3), 2.25 (s, 3H, ArCH_3), and 0.44 [s, 9H, Si$(CH_3)_3$].

^{1}H NMR identifiable resonances for the minor atropisomer (500 MHz, $CDCl_3$): 7.15 (s, 1H, Ar*H8*), 6.96 (1H, ddd, *J* = 8.3, 7.5, 1.5 Hz, 1H, Ar*H7'*), 6.89 (s, 1H, Ar*H5*), 6.68 (ddd, *J* =7.3, 7.3, 1.1 Hz, 1H, Ar*H6'*), 6.51 (dd, *J* = 9.9, 1.6 Hz, 1H, *H*4'), 6.01 (br d, *J* = 8.1 Hz, 1H, *H8*'), 5.67 (dd, *J* = 2.1, 3.3 Hz, 1H, *H2'*), 5.78 (dd, J = 9.7, 3.4, 1H, *H3'*), 3.96 (s, 3H, OCH_3), 3.91 (s, 3H, OCH_3), 2.47 (s, 3H, ArCH_3), and 0.42 [s, 9H, ArSi$(CH_3)_3$].

^{13}C NMR for the major atropisomer (126 MHz, $CDCl_3$): δ 194.1, 154.5, 149.7, 146.8, 144.3, 143.5, 142.3, 141.6, 139.2, 138.8, 131.8, 129.2, 128.6, 128.4, 127.8, 127.0, 126.5, 123.0, 121.8, 121.6, 120.7, 118.7, 113.8, 107.0, 102.6, 89.0, 85.1, 56.3, 56.2, 51.4, 19.2, and 2.9.

HRMS (ESI-TOF): Calculated for $C_{36}H_{32}NO_3Si^+$ $[M–H^-]^+$: 554.2146, found 554.2135 and calculated for $C_{28}H_{28}NO_3Si^+$ $[M–C≡CPh^-]^+$: 454.1833, found 454.1825.

IR (thin film): 3053, 3003, 2939, 2899, 2837, 1703, 1659, 1590, 1487, 1454, 1410, 1381, 1314, 1243, 1214, 1119, 1098, 1065, 1015, 990, 914, 843, 798, 733, 690, 633, 602, 525, 500, 451, and 416 cm^{-1}.

Reverse phase liquid chromatography: Only one peak corresponding to **36** was observed, consistent with the assumption that the atropisomers are interconverting sufficiently rapidly to coeluted.

6,7-Dimethoxy-2-methyl-1-(trimethylsilyl)-3-(2-((trimethylsilyl)ethynyl)quinolin-1(2*H*)-yl)-9*H*-fluoren-9-one (37):

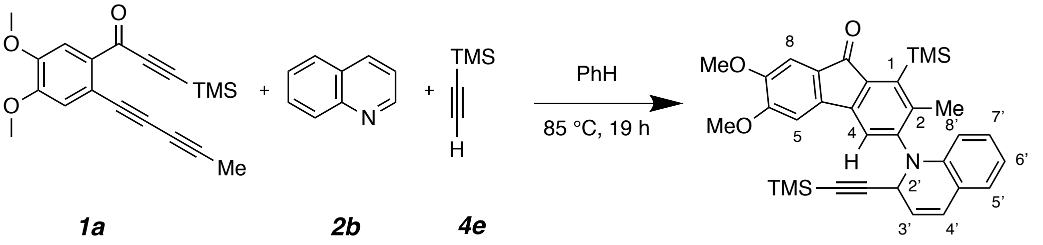

***37* (84%)**
Ratio of atropisomers: 1:0.2

Triynone **1a** (30 mg, 0.092 mmol), quinoline (**2b,** 22 μL, 0.184 mmol, 2 equiv), and ethynyltrimethylsilane (**4e**, 64 μL, 0.462 mmol, 5 equiv) were combined in a culture tube, dissolved in benzene (6 mL, 0.02M), and sealed with a Teflon-lined screw-cap. The solution was heated overnight (18-19 h) in an oil bath at 85 °C, cooled, and passed through a plug of silica (2:1, Hex:EtOAc eluant). The residue was purified by MPLC (3:1, Hex:EtOAc) to give **37** (43 mg, 84% overall, 1:0.2 ratio) as a yellow oil.

Data for 37 [a mixture of atropisomers in a ratio of 1 :0.2]:

^{1}H NMR for the major atropisomer (500 MHz, $CDCl_3$): 7.76 (s, 1H, Ar*H4*), 7.16 (s, 1H, Ar*H8*), 7.04 (dd, *J* = 7.3, 1.5 Hz, 1H, ArH5'), 6.99 (1H, ddd, *J* = 8.2, 7.4, 1.7 Hz, 1H, Ar*H7'*), 6.90 (s, 1H, Ar*H5*), 6.72 (ddd, *J* =7.5, 7.5, 1.2 Hz, 1H, Ar*H6'*), 6.52 (ddd, *J* = 9.6, 1.0, 1.0 Hz, 1H, *H*4'), 6.24 (ddd, *J* = 8.2, 0.9, 0.9 Hz, 1H, *H8*'), 5.79 (dd, *J* = 9.6, 5.6 Hz, 1H, *H3'*), 5.01 (dd, *J* = 5.6, 0.9 Hz, 1H, *H2'*), 3.957 (s, 3H, O*CH₃*), 3.925 (s, 3H, O*CH₃*), 2.18 (s, 3H, ArCH_3), 0.42 [s, 9H, $ArSi(CH_3)_3$], and 0.14 [s, 9H, C≡CH_3Si$(CH_3)_3$].

^{1}H NMR identifiable resonances for the minor atropisomer (500 MHz, $CDCl_3$): 7.15 (s, 1H, Ar*H8*), 6.94 (1H, ddd, *J* = 7.4, 7.4, 1.6 Hz, 1H, Ar*H7'*), 6.89 (s, 1H, Ar*H5*), 6.65 (ddd, *J* =7.4, 7.4, 0.9 Hz, 1H, Ar*H6'*), 6.45 (nfom, 1H, *H*4'), 6.01 (br d, *J* = 8.1 Hz, 1H, *H8*'), 5.67 (nfom, 1H, *H3'*), 5.66 (s, 1H, *H2'*), 3.961 (s, 3H, OCH_3), 3.916 (s, 3H, OCH_3), 2.41 (s, 3H, ArCH_3), 0.45 [s, 9H, $ArSi(CH_3)_3$], and 0.03 [s, 9H, C≡CH_3Si$(CH_3)_3$].

^{13}C NMR for the major atropisomer (101 MHz, $CDCl_3$): δ 194.1, 154.6, 149.7, 146.9, 144.3, 143.6, 142.2, 141.4, 139.2, 138.8, 129.1, 127.0, 127.7, 126.3, 121.5, 121.6, 120.8, 118.6, 113.7, 107.0, 105.1, 102.8, 89.4 56.4, 56.4, 51.5, 19.3, 2.9, and 0.2.

HRMS (APCI-Orbitrap): Calculated for $C_{33}H_{38}NO_3Si_2^+$ [M+H$^+$]: 552.2385, found 552.2373.

IR (thin film): 3001, 2955, 2927, 2900, 2853, 2161, 1704, 1589, 1487, 1455, 1410, 1381, 1315, 1244, 1214, 1153, 1099, 1066, 1016, 944, 916, 840, 800, 748, 701, 659, 634, 603, 539, 498, 460, and 418 cm^{-1}.

2-(2-(6,7-Dimethoxy-2-methyl-9-oxo-1-(trimethylsilyl)-9H-fluoren-3-yl)-1,2-dihydroisoquinolin-1-yl)fumaronitrile (38) :

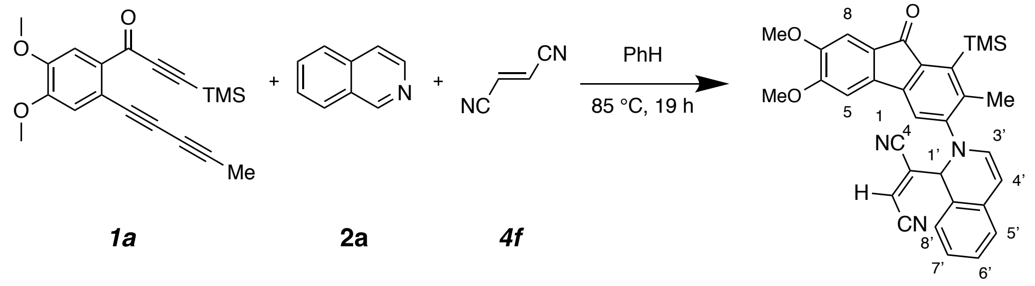

Triynone **1a** (20 mg, 0.062 mmol), isoquinoline (**2a**, 14.5 μL, 0.124 mmol, 2 equiv) and fumaronitrile (**4f**, 24.2 mg, 0.310 mmol, 5 equiv) were combined in a culture tube, dissolved in benzene (4 mL, 0.02 M), and sealed with a Teflon-lined cap. The solution was heated overnight (18-19 h) in an oil bath at 85 °C, cooled, and passed through a plug of silica (EtOAc elution). The eluant was concentrated and the residue purified by MPLC (3:1 Hex:EtOAc) to give **38** (0.060 mmol, 85%) as a brick red oil, which was an orange oil after passage through a normal phase high pressure liquid chromatography column (HPLC). The ^{1}H NMR spectra appeared essentially identical before and after the HPLC treatment.

Data for 38:

^{1}H NMR (500 MHz, $CDCl_3$): δ 7.32 (ddd, *J* = 7.6, 7.6, 1.2 Hz, 1H, Ar*H6'*), 7.31 (s, 1H, Ar*H5* or Ar*H4*), 7.19 (ddd, *J* = 7.6, 7.6, 1.3 Hz, 1H, Ar*H7'*), 7.15 (s, 1H, Ar*H*8), 7.13 (dd, *J* = 7.8, 1.1 Hz, 1H, Ar*H8'*), 7.09 (dd, *J* = 7.7. 1.0 Hz, 1H, Ar*H*5'), 6.99 (s, 1H, Ar*H5* or Ar*H4*), 6.45 (br d, 1H, *J* = 7.8 Hz, C=C*H3'*), 6.18 [br s, 1H, *H*C=C(CN)], 5.62 (d, *J* = 8.0 Hz, 1H, C=C*H4'*), 5.58 (br s, 1H, C*H1'*), 4.03 (s, 3H, OCH_3), 3.92 (s, 3H, OCH_3), 2.51 (s, 3H, ArCH_3) and 0.44 [s, 9H, $Si(CH_3)_3$].

^{13}C NMR (125 MHz, $CDCl_3$): δ 193.6, 154.8, 150.1, 147.6, 145.7, 144.0, 139.1, 138.7, 138.1, 134.2, 133.7, 132.1, 130.2, 127.0, 127.0, 126.8, 125.2, 123.6, 118.3, 115.9, 113.1, 109.0, 107.0, 103.1, 101.7, 63.0, 56.7, 56.4, 20.1, and 2.6.

IR ($CDCl_3$): 2956, 2923, 2853, 2255, 2208, 1704, 1589, 1495, 1463, 1377, 1315, 1244, 1216, 1091, 1045, 1015, 910, 845, 793, 767, 730, 647, 603, and 417 cm^{-1}.

HRMS (APCI-Orbitrap): Calculated for $C_{28}H_{28}NO_3Si^+$ [M-(CN)C=C(CN)(H)]: 454.1833, found 454.1831 (most intense ion); Calculated for $C_{32}H_{30}N_3O_3Si^+$ [M+H$^+$]: 532.2051, found 532.2009 (minor ion).

2-(2-(6,7-Dimethoxy-2-methyl-9-oxo-1-(trimethylsilyl)-9H-fluoren-3-yl)-1,2-dihydrophthalazin-1-yl)fumaronitrile (39):

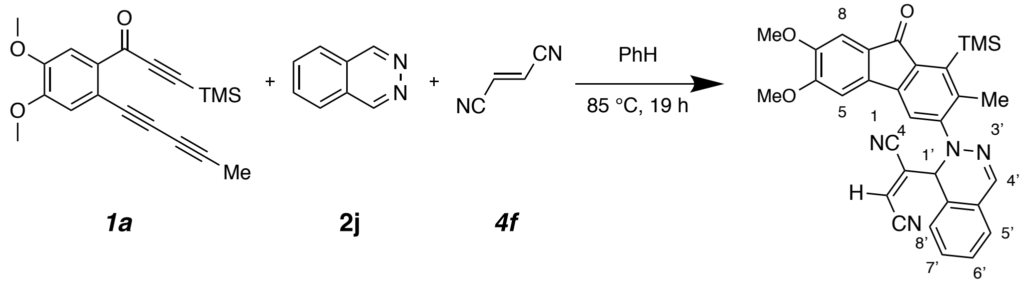

1a **2j** ***4f*** ***39*** **(35%)**

Triynone **1a** (25 mg, 0.077 mmol, 1 equiv), phthalazine (**2j**, 31 mg, 0.231 mmol, 3 equiv), and fumaronitrile (**4f**, 30 mg, 0.385 mmol, 5 equiv) were combined in a culture tube, dissolved in benzene (10 mL), and sealed with a Teflon-lined screw-cap. The tube was heated overnight (18-19 h) in an oil bath at 85 °C and cooled. The contents were passed through a plug of silica (EtOAc elution). The eluate was concentrated and the residue purified by MPLC (1:1 Hex:EtOAc) to give **39** (14 mg, 0.0263 mmol, 35%) as a yellow oil. The absence of a difference nOe between *H1'* and (NC)*H*C=CCN was used as the basis for assigning the *E*-alkene geometry.

Data for 39:

^{1}H NMR (400 MHz, $CDCl_3$): δ 7.61 (s, 1H, Ar*H4'*), 7.64 (s, 1H, Ar*H4 or ArH5*), 7.55 (m, 2H, Ar*H6* and Ar*H7'*), 7.44 (nfom, 1H, Ar*H5'* or Ar*H8'*), 7.25 (nfom, 1H, Ar*H5'* or Ar*H8'*), 7.15 (s, 1H, Ar*H8*), 7.05 (s, 1H, Ar*H4 or ArH5*), 6.06 [s, 1H, *H*C=C(CN)], 5.63 (s, 1H, C*H1'*), 4.04 (s, 3H, OCH_3), 3.92 (s, 3H, OCH_3), 2.54 (s, 3H, ArCH_3), and 0.45 [s, 9H, $Si(CH_3)_3$].

^{13}C NMR (125 MHz, $CDCl_3$): δ 193.8, 154.7, 149.9, 148.8, 145.2, 143.7, 139.2, 138.7, 138.5, 135.7, 133.2, 132.0, 130.9, 127.1, 126.8, 126.6, 126.3, 124.4, 117.7, 115.5, 112.8, 111.2, 106.8, 103.3, 59.4, 56.7, 56.4, 20.0, and 2.6.

IR ($CDCl_3$): 3038, 3007, 2942, 2900, 2839, 2254, 1702, 1588, 1494, 1463, 1455, 1381, 1360, 1315, 1244, 1215, 1150, 1089, 1018, 993, 908, 847, 759, 727, 647, 594, and 564 cm^{-1}.

HRMS (APCI-Orbitrap): Calculated for $C_{27}H_{27}N_2O_3Si^+$ [M-(CN)C=C(CN)(H)]: 455.1785, found 455.1785 (most intense ion); Calculated for $C_{31}H_{29}N_4O_3Si^+$ [M+H$^+$]: 533.2003, found 533.2004 (minor ion).

7-(6,7-Dimethoxy-2-methyl-9-oxo-1-(trimethylsilyl)-9H-fluoren-3-yl)-3,3-dimethyl-2,3,4,6,7,12-hexahydro-1H-6,12-methanodibenzo[d,g][1,3]oxazocin-1-one (40-A) and

7-(6,7-Dimethoxy-2-methyl-9-oxo-1-(trimethylsilyl)-9H-fluoren-3-yl)-3,3-dimethyl-2,3,4,6,7,12-hexahydro-1H-6,12-methanodibenzo[d,g][1,3]oxazocin-1-one (40-B)

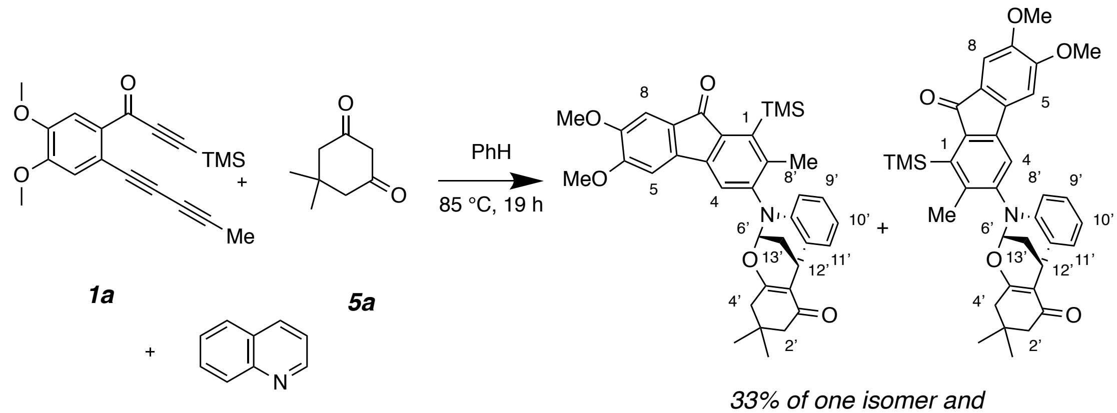

Triynone **1a** (30 mg, 0.092 mmol), quinoline (**2b,** 22 µL, 0.184 mmol, 2 equiv), and dimedone (**5a**, 64.7 mg, 0.462 mmol, 5 equiv) were combined in a culture tube, dissolved in a mixture of benzene and acetonitrile (8 mL, 0.02M, 3:1 ratio), and sealed in a vial with a Teflon-lined screw cap. The solution was heated overnight (18-19 h) in an oil bath at 85 °C, cooled, and passed through a plug of silica (1:1, Hex:EtOAc). The residue was purified by MPLC (2:1, Hex:EtOAc) to give, in order of elution, atropisomeric diastereomers, **40-A** (018 mg, 0.030 mmol, 33%) as yellow oil and **40-B** (24 mg, 0.040, 44%) also as a yellow oil, which solidified upon storage at -10 °C. This product returned to an oily state upon being allowed to warm to ambient temperature.

Data for faster eluting, minor isomer, 40-A:

^{1}H NMR (500 MHz, CDCl$_3$): 7.47 (dd, J = 7.6, 1.7 Hz, 1H, ArH*11'*), 7.15 (s, 1H, Ar*H8*), 6.98 (s, 1H, Ar*H4*), 6.96 (ddd, J = 8.0, 7.3, 1.6 Hz, 1H, Ar*H9'*), 6.78 (s, 1H, Ar*H5*), 6.76 (ddd, J = 7.4, 7.4, 1.2 Hz, 1H, Ar*H10'*), 6.28 (dd, J = 8.2, 1.1 Hz, 1H, Ar*H8'*), 5.55 (nfom, 1H, *H6'*), 4.23 (nfom, 1H, *H12'*), 3.93 (s, 3H, OCH_3), 3.92 (s, 3H, OCH_3), 2.33 (s, 3H, ArCH$_3$), 2.29 (br s, 2H, *H4'*), 2.25 (s, 2H, *H2'*), 2.19 (ddd, J = 13.1, 2.6, 2.6 Hz, 1H, *C13'*H_aH$_b$), 2.16 (ddd, J = 12.8, 3.1, 3.1 Hz, 1H, *C13'*H$_a$$H_b$), 1.08 (s, 3H, C3'*CH$_3$*), 1.04 (s, 3H, C3'*CH$_3$*), and 0.47 [s, 9H, Si(CH$_3$)$_3$].

^{13}C NMR (126 MHz, CDCl$_3$): δ 195.9(C1'), 194.0(C9), 168.0(C4a'), 154.7, 149.9, 146.5, 144.5, 143.8, 142.2, 140.7, 140.0, 138.5, 128.5, 127.6, 127.2, 126.9, 122.1(C4), 119.3, 115.5, 113.5, 107.0(C8), 102.9(C5), 83.6, 56.5, 56.4, 50.6, 42.3, 32.5, 29.5, 27.9, 26.0, 25.4, 19.2, and 2.9.

HRMS (ESI-TOF): Calculated for $C_{36}H_{40}NO_5Si^+$ [M+H$^+$]: 594.2670, found 594.2659.

IR (thin film): 2955, 2897, 2870, 2838, 1706, 1650, 1618, 1591, 1491, 1457, 1375, 1316, 1266, 1245, 1215, 1196, 1181, 1162, 1111, 1074, 1039, 1017, 992, 958, 910, 845, 790, 730, 699, 677, 650, 621, 601, 571, 508, 490, 453, 432, and 412 cm^{-1}.

Data for slower eluting, major isomer, 40-B:

^{1}H NMR (500 MHz, $CDCl_3$): 7.47 (dd, *J* = 7.6, 1.6 Hz, 1H, ArH*11'*), 7.20 (s, 1H, Ar*H4*), 7.17 (s, 1H, Ar*H8*), 6.95 (ddd, *J* = 8.2, 7.4, 1.7 Hz, 1H, Ar*H9'*), 6.93 (s, 1H, Ar*H5*), 6.76 (ddd, *J* = 7.5, 7.5, 1.2 Hz, 1H, Ar*H10*'), 6.19 (dd, *J* = 8.2, 1.2 Hz, 1H, Ar*H8*'), 5.94 (nfom, *H6'*), 4.23 (dd, *J* = 3.2, 3.2 Hz, 1H, *H12'*), 3.99 (s, 3H, OCH_3), 3.93 (s, 3H, OCH_3), 2.33 (s, 3H, ArCH_3), 2.17–2.29 (m, 4H, C*H2'* and C*H4'*), 2.16 (ddd, *J* =12.5, 3.2, 3.2 Hz, 1H, $C13'H_aH_b$), 2.12 (ddd, *J* = 12.8, 2.4, 2.4 Hz, 1H, $C13'H_aH_b$), 1.07 (s, 3H, C3'CH_3), 0.97 (s, 3H, C3'CH_3) and 0.44 [s, 9H, $Si(CH_3)_3$].

^{13}C NMR (126 MHz, $CDCl_3$): δ 196.2 (C1'), 193.9 (C9), 167.7 (C4a'), 154.7 (C67), 149.9 (C67), 146.9, 144.8, 144.1, 143.6, 139.9 (2x), 138.3, 128.6 (C11'), 127.3 (C8'), 126.9 (C9'), 126.4, 120.9 (C4), 119.2 (C10'), 114.8, 111.9 (C8'), 107.0 (C8), 102.8 (C5), 85.6, 56.5, 56.4, 50.7, 42.1, 32.3, 29.2, 27.9, 26.2, 25.6, 19.7, and 3.0.

HRMS (ESI-TOF): Calculated for $C_{36}H_{40}NO_5Si^+$ [M+H$^+$]: 594.2670, found 594.2658.

IR (thin film): 3036, 2956, 2898, 2871, 1706, 1651, 1618, 1592. 1491, 1459, 1381, 1369, 1317, 1269, 1246, 1215, 1180, 1151, 1111, 1075, 1040, 1018, 994, 959, 910, 861, 840, 792, 751, 731, 700, 677, 648, 622, 603, 573, 512, 490, 454, and 432 cm^{-1}.

3-(5- -4-methyl-2H-2,6-methanobenzo[d][1,3]oxazocin-1(6H)-yl)-6,7-dimethoxy-2-methyl-1-(trimethylsilyl)-9H-fluoren-9-one (41-A) and

3-(5-Acetyl-4-methyl-2H-2,6-methanobenzo[d][1,3]oxazocin-1(6H)-yl)-6,7-dimethoxy-2-methyl-1-(trimethylsilyl)-9H-fluoren-9-one (41-B):

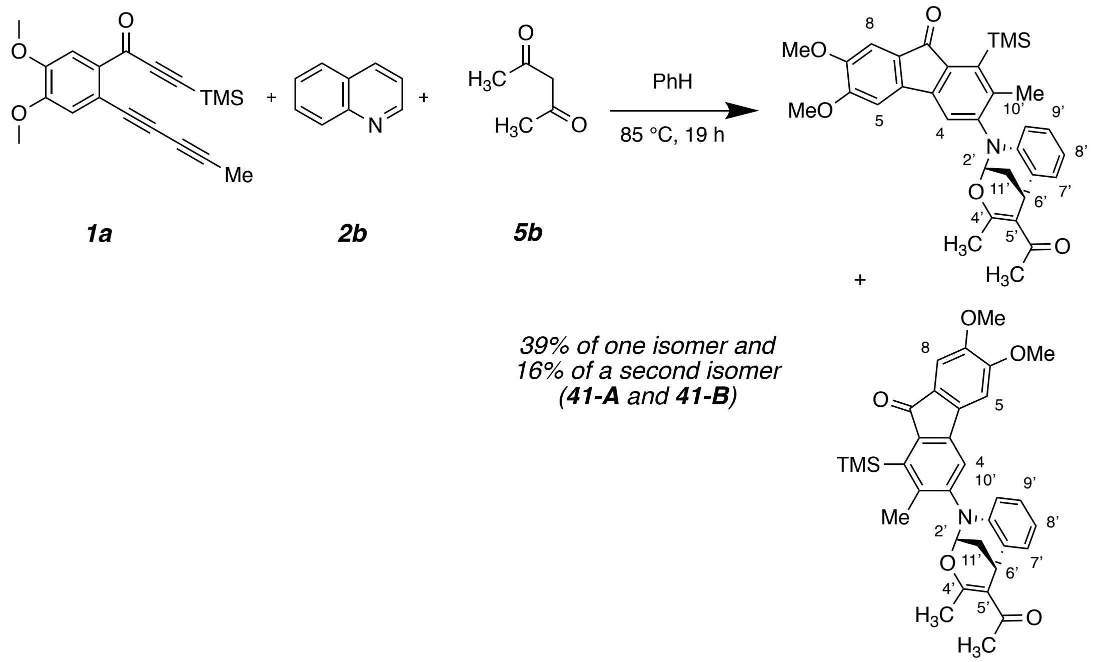

Triynone **1a** (30 mg, 0.092 mmol), quinoline (**2b**, 22 *μ*l, 0.184 mmol, 2 equiv), and acetylacetone (**5b**, 10 μL, 0.462 mmol, 5 equiv) were combined in a culture tube, dissolved in benzene (6 mL, 0.02M), and sealed in a vial with a Teflon-lined screw-cap. The solution was heated overnight (18-19 h) in an oil bath at 85 °C, cooled, and passed through a plug of silica (1:1, Hex:EtOAc). The residue was purified by MPLC (2:1, Hex:EtOAc) to give, in order of elution, impure **41-A** (20 mg, 0.029 mmol, 39% yield, corrected for the presence of residual quinoline from the mixture) as yellow oil and **41-B** (8 mg, 0.015 mmol, 16%) also as a yellow oil. A small portion of each product was later repurified by HPLC (2:1, Hex:EtOAc for **41-A** and 1:1, Hex:EtOAc for **41-B** to give samples of higher purity for characterization purposes.

Data for faster eluting, major isomer, 41-A:

^{1}H NMR (500 MHz, $CDCl_3$): 7.38 (dd, *J* = 7.5, 1.6 Hz, 1H, ArH*7'*), 7.15 (s, 1H, Ar*H8*), 7.07 (s, 1H, Ar*H4*), 6.97 (ddd, *J* = 8.1, 7.3, 1.7 Hz, 1H, Ar*H9'*), 6.84 (s, 1H, Ar*H5*), 6.75 (ddd, *J* = 7.4, 7.4, 1.2 Hz, 1H, Ar*H8*'), 6.29 (dd, *J* = 8.2, 1.2 Hz, 1H, Ar*H10*'), 5.45 (nfom, 1H, *H2'*), 4.28 (nfom, 1H, *H6'*), 3.95 (s, 3H, OCH_3), 3.91 (s, 3H, OCH_3), 2.45 (s, 3H, CH_3C=O), 2.30 [s, 3H, ArCH_3], 2.27 [s, 3H, C4'CH_3], 2.15 (dd, *J* = 3, 3 Hz, 2H, *C11'H*$_2$), and 0.47 [s, 9H, Si(CH_3)$_3$].

^{13}C NMR (126 MHz, CDCl$_3$): δ 197.1(MeC=O), 193.9(C9), 162.9(C4'), 154.6, 149.7, 146.4, 144.2, 143.8, 142.1, 140.8, 139.9, 138.5, 127.7, 127.4, 127.4, 126.7, 122.2(C4), 118.8, 117.7(C5'), 113.4, 106.8(C8), 102.9(C5), 82.2(C2'), 56.5, 56.2, 31.0, 28.9(C6'), 25.5, 21.2, 19.1, and 2.8.

HRMS (APCI-Orbitrap): Calculated for $C_{33}H_{36}NO_5Si^+$ [M+H$^+$]: 554.2357, found 554.2359.

IR (thin film): 3068, 2926, 2853, 1705, 1666, 1589, 1492, 1458, 1376, 1356, 1317, 1264, 1246, 1216, 1176, 1150, 1120, 1108, 1075, 1049, 1018, 976, 933, 896, 843, 810, 751, 732, 700, 655, 613, 517, and 436 cm^{-1}.

Data for slower eluting, minor isomer, 41-B: ^{1}H NMR (500 MHz, CDCl$_3$): 7.36 (dd, *J* = 7.5, 1.6 Hz, 1H, ArH*7'*), 7.19 (s, 1H, *H4*), 7.17 (s, 1H, Ar*H8*), 6.97 (ddd, *J* = 8.1, 7.3, 1.7 Hz, 1H, Ar*H9'*), 6.92 (s, 1H, *H5*), 6.75 (ddd, *J* = 7.4, 7.4, 1.2 Hz, 1H, Ar*H8*'), 6.22 (dd, *J* = 8.1, 1.3 Hz, 1H, Ar*H10*'), 5.86 (nfom, 1H, Σ*J* = 6.8 Hz, *H2'*), 4.27 (nfom, Σ*J* = 8.0 Hz, 1H, *H6'*), 3.98 (s, 3H, OCH_3), 3.93 (s, 3H, OCH_3), 2.39 (s, 3H, CH_3C=O), 2.21 [s, 3H, C4'CH_3], 2.15 (ddd, *J* = 12.7, 3.3, 3.3 Hz, 1H, *C11*'H_aH$_b$), 2.07 (ddd, *J* = 12.5, 2.6, 2.6 Hz, 1H, *C11'*H$_a$$H_b$) 2.01 [s, 3H, Ar$CH_3$], and 0.43 [s, 9H, Si(CH$_3$)$_3$].

^{13}C NMR (126 MHz, CDCl$_3$): δ 197.4 (MeC=O), 194.0(C9), 162.6(C4'), 154.7, 149.9, 146.8, 144.7, 144.2, 143.7, 140.3, 139.8, 138.4, 127.8, 127.4, 126.9, 126.7, 120.8 (C4), 118.9, 117.0, 112.1, 107.0 (C8), 102.8 (C5), 84.1 (C2'), 56.5, 56.4, 31.3, 28.9 (C6'), 25.9, 21.2, 19.4, and 2.9.

HRMS (APCI-Orbitrap): Calculated for $C_{33}H_{36}NO_5Si^+$ [M+H$^+$]: 554.2357, found 554.2355.

IR (thin film): 3065, 2926, 2853, 1704, 1666, 1589, 1491, 1458, 1379, 1355, 1316, 1264, 1244, 1215, 1177, 1149, 1120, 1107, 1075, 1048, 1008, 976, 932, 896, 839, 730, 700, 648, 620, 602, 575, 517, and 432 cm^{-1}.

9-(6,7-Dimethoxy-2-methyl-9-oxo-1-(trimethylsilyl)-9H-fluoren-3-yl)-9,14-dihydro-1H,8H-8,14-methanobenzo[d]chromeno[3,4-g][1,3]oxazocin-1-one (42-A) and

9-(6,7-Dimethoxy-2-methyl-9-oxo-1-(trimethylsilyl)-9H-fluoren-3-yl)-9,14-dihydro-1H,8H-8,14-methanobenzo[d]chromeno[3,4-g][1,3]oxazocin-1-one (42-B):

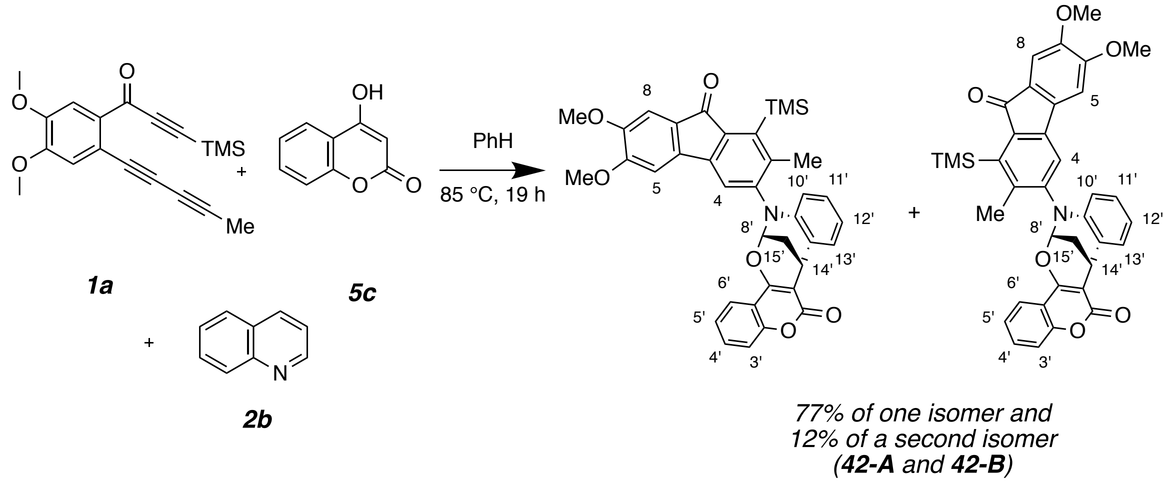

Triynone **1a** (30 mg, 0.092 mmol), quinoline (**2b**, 22 μL, 0.184 mmol, 2 equiv), and 4-hydroxycoumarin (**5c**, 75 mg, 0.462 mmol, 5 equiv) were combined in a culture tube, dissolved in a mixture of benzene and acetonitrile (6 mL, 0.02M, 6:1 ratio), and sealed with a Teflon-lined screw-cap. The solution was heated overnight (18-19 h) in an oil bath at 85 °C, cooled, and passed through a plug of silica (3:1, Hex:EtOAc). The residue was purified by MPLC (3:1, Hex:EtOAc) to give, in order of elution, **42-A** (43 mg, 0.070 mmol, 77%) as a pale yellow amorphous film and **42-B** (7 mg, 0.011 mmol, 12%) as a yellow amorphous powder. A small portion of each sample was separately repurified by HPLC (3:1, Hex:EtOAc) to give samples of somewhat higher purity for characterization purposes.

Data for faster eluting, major diastereomer: 42-A:

^{1}H NMR (500 MHz, $CDCl_3$): 7.73 (dd, *J* = 7.9, 1.6 Hz, 1H, ArH*6'*), 7.60 (dd, *J* = 7.5, 1.6 Hz, 1H, ArH*13*'), 7.49 (ddd, *J* = 8.4, 7.3, 1.7 Hz, 1H, Ar*H4'*), 7.32 (dd, *J* = 8.3, 1.2 Hz, 1H, Ar*H3*'), 7.19 (ddd, *J* = 8.1, 7.2, 1.1 Hz, 1H, Ar*H5'*), 7.13 (s, 1H, Ar*H8*), 6.99 (ddd, *J* = 8.1, 7.3, 1.7 Hz, 1H, Ar*H11'*), 6.87 (s, 1H, Ar*H4*), 6.82 (ddd, *J* = 7.4, 7.4, 1.1 Hz, 1H, Ar*H12'*), 6.45 (s, 1H, Ar*H5*), 6.28 (dd, *J* = 8.2, 1.2 Hz, 1H, Ar*H10*'), 5.82 (nfom, 1H, *H8'*), 4.40 (ddd, *J* = 3.0, 3.0, 1.5 Hz, 1H, *H14'*), 3.90 (s, 3H, OCH_3), 3.70 (s, 3H, OCH_3), 2.47 (ddd, *J* = 13.1, 3.2, 3.2 Hz, 1H, $C15'H_aH_b$), 2.45 (ddd, *J* = 13.0, 2.8, 2.2 Hz, 1H, $C15'H_aH_b$), 2.40 (s, 3H, $ArCH_3$), and 0.50 [s, 9H, $Si(CH_3)_3$].

^{13}C NMR (125 MHz, $CDCl_3$): δ 194.0, 161.9, 158.9, 154.7, 152.5, 149.9, 145.8, 144.2, 143.9, 142.7, 140.7, 140.4, 138.4, 131.8, 128.6, 127.9, 126.7, 126.2, 123.9, 122.8, 122.6, 119.7, 116.9, 115.9, 113.5, 106.9, 106.6, 102.7, 84.1, 56.36, 56.34, 27.6, 25.9, 18.9, and 3.0.

HRMS (APCI-Orbitrap): Calculated for $C_{37}H_{34}NO_6Si^+$ [M+H$^+$]: 616.2150, found 616.2154.

IR (thin film)**:** 3070, 2945, 2903, 2853, 1708, 1626, 1592, 1492, 1456, 1383, 1317, 1295, 1266, 1246, 1214, 1151, 1114, 1101, 1076, 1038, 1018, 965, 929, 912, 860, 751, 699, 673, 622, 604, 499, and 437 cm^{-1}.

Data for slower eluting, minor isomer, 42-B:

^{1}H NMR (500 MHz, $CDCl_3$): 7.71 (br d, *J* = 7.8 Hz, 1H, ArH*6'*), 7.60 (br d, *J* = 7.6 Hz, 1H, ArH*13*'), 7.50 (br t, *J* = 7.9 Hz, 1H, Ar*H4'*), 7.30 (br d, *J* = 8.0 Hz, 1H, Ar*H3*'), 7.26 (s, 1H, *H4* or *H5*), 7.25 (br t, *J* = 7.5 Hz, 1H, Ar*H5'*), 7.18 (s, 1H, Ar*H8*), 7.00 (ddd, *J* = 7.5, 7.5, 1.7 Hz, 1H, Ar*H11'*), 6.96 (s, 1H, *H4* or *H5*), 6.82 (br dd, *J* =7.4, 7.4 Hz, 1H, Ar*H12'*), 6.22 (br d, *J* = 8.1 Hz, 1H, Ar*H10'*), 6.21 (m, 1H, *H8'*), 4.39 (br s, 1H, *H14'*), 4.00 (s, 3H, OCH_3), 3.94 (s, 3H, OCH_3), 2.44 (br d, *J* = 12.8 Hz, 1H, $C15'H_aH_b$), 2.35 (br d, *J* = 12.8 Hz, 1H, $C15'H_aH_b$), 1.79 (s, 3H, $ArCH_3$), and 0.37 [s, 9H, $Si(CH_3)_3$].

^{13}C NMR (126 MHz, $CDCl_3$): δ 193.9, 162.1, 158.7, 154.8, 152.4, 150.0, 146.4, 144.8, 144.3, 144.1, 140.3, 140.0, 138.3, 131.9, 128.7, 128.0, 126.9, 125.0, 124.1, 122.8, 121.0, 119.6, 116.9, 115.6, 112.2, 107.1, 105.6, 102.9, 86.1, 56.5, 56.4, 27.7, 26.0, 19.5, and 2.8.

HRMS (ESI-TOF): Calculated for $C_{37}H_{33}NNaO_6Si^+$ [M+Na$^+$]: 638.1969, found 638.1957.

IR (thin film)**:** 3056, 2926, 2853, 1702, 1625, 1590, 1491, 1455, 1382, 1370, 1316, 1295, 1265, 1241, 1210, 1146, 1113, 1100, 1075, 1037, 1015, 964, 929, 912, 889, 845, 813, 732, 699, 671, 637, 622, 602, 574, 557, 543, 497, 479, 437, and 411 cm^{-1}.

3,3-Dimethyl-7-(5-methyl-1-(methylsulfonyl)-4-(prop-1-yn-1-yl)indolin-6-yl)-2,3,4,6,7,12-hexahydro-1H-6,12-methanodibenzo[d,g][1,3]oxazocin-1-one (43-A) and

3,3-Dimethyl-7-(5-methyl-1-(methylsulfonyl)-4-(prop-1-yn-1-yl)indolin-6-yl)-2,3,4,6,7,12-hexahydro-1H-6,12-methanodibenzo[d,g][1,3]oxazocin-1-one (43-B)

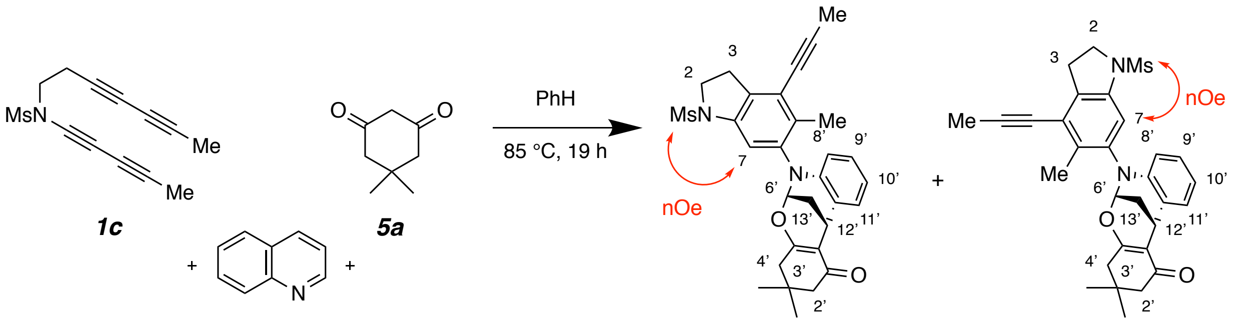

2b

*85% (overall), **43-A** and **43-B***
1:0.74 (ratio of isomers from NMR)

Tetrayne **1c** (50 mg, 0.202 mmol), quinoline (**2b**, 71.9 μL, 0.606 mmol, 3 equiv), and dimedone (**5a**, 142 mg, 1.01 mmol, 5 equiv) were combined in a culture tube, dissolved in a mixture of benzene and acetonitrile (14 mL, 3:2 ratio, 0.02M), and sealed in a vial with a Teflon-lined screw-cap. The solution was heated overnight (18–19 h) in an oil bath at 85 °C, cooled, and passed through a plug of silica (1:1, Hex:EtOAc). The residue was purified by MPLC (2:1, Hex:EtOAc) to give, as partially overlapping peaks, a mixture of **43-A** and **43-B** (88 mg, 85% yield). These were present in a 1:0.74 ratio (^{1}H NMR spectrum) in the crude product mixture. A small portion of the mixture was separately repurified by normal phase HPLC (2:1, Hex:EtOAc) to give, in order of elution, **43-A** (major isomer) as a transparent oil, and **43-B** (minor isomer) also as a transparent oil. Differential nOe was used to confirm that each isomer had the same constitution (i.e., that the compounds were atropisomers and not regioisomers). The assignment of relative configuration within each atropisomeric is ambiguous. These further-purified samples were used for characterization purposes.

Data for faster eluting isomer, major isomer, 43-A:

^{1}H NMR (500 MHz, CDCl$_3$): δ 7.41 (dd, *J* = 7.5, 1.5 Hz, 1H, Ar*H11'*), 7.03 (s, 1H, Ar*H7*), 6.87 (ddd, *J* = 8.0, 8.0, 1.5 Hz, 1H, Ar*H9'*), 6.70 (ddd, *J* = 7.5, 7.5, 1.0 Hz, 1H, Ar*H10'*), 6.03 (d, *J* = 8.1 Hz, Ar*H8'*), 5.40 (nfom, 1H, *H6'*), 4.19 (nfom, 1H, *H12'*), 4.03 (ddd, *J* = 10.3, 8.5, 8.5 Hz, 1H, $CH_3SO_2NCH_aH_bCH_2$), 3.99 (ddd, *J* = 10.2, 8.9, 8.9 Hz, 1H, $CH_3SO_2NCH_aH_bCH_2$), 3.23 (t, J = 8.7 Hz, 2H, $NMsCH_2CH_2$), 2.80 (s, 3H, CH_3SO_2N), 2.70 (d, *J* = 17.2 Hz, 1H, C2'H_aH$_b$ or C4'H_aH$_b$), 2.26–2.15 (overlapping m, 2H, C4'H_2), 2.25 (s, 3H, $NArCH_3$), 2.21 (d, *J* = 17.2 Hz, 1H, C2'H$_a$$H_b$ or C4'H$_a$$H_b$), 2.17 (ddd, *J* = 12.6, 2.6, 2.6 Hz, 1H, C13'H_aH$_b$), 2.16 (s, 3H, NArC≡CCH$_3$), 2.13 (ddd, *J* = 12.7, 3.1, 3.1 Hz, 1H, C13'H$_a$$H_b$), 1.05 [s, 3H, ($CH_3$)C3'], and 0.99 [s, 3H, (CH_3)C3'].

^{13}C NMR (125 MHz, $CDCl_3$): δ 196.3, 168.3, 141.8, 140.8, 140.8, 134.6, 133.8, 128.3, 127.1, 127.0, 123.1, 118.8, 115.8, 115.5, 112.4, 95.3 (alkyne), 83.6 (*C6'*), 75.9 (alkyne), 50.64 (*C2'* or *C4'*), 50.59 (NMsCH_2), 41.9 (*C2'* or *C4'*), 34.4 (Ms), 32.4 (C3'), 29.2 [(CH_3)C3'], 28.4 (NMsCH_2CH_2), 27.7 [(CH_3)C3'], 26.1 (C13'), 25.5 (*C12'*), 15.3 (NArCH_3), and 4.7 (NArC≡CCH_3). The indicated carbon peaks are assigned using HSQC data.

HRMS (APCI-Orbitrap): Calculated for $C_{30}H_{33}N_2O_4S^+$ [M+H$^+$]: 517.2156, found 517.2162.

IR (thin film): 3055, 2958, 2929, 2890, 2870, 2358, 2234, 1649, 1616, 1490, 1454, 1380, 1348, 1265, 1231, 1182, 1158, 1110, 1075, 1039, 968, 911, 825, 791, 730, 701, 665, 623, 543, 513, 495, and 455 cm^{-1}.

Data for slower eluting, minor isomer, 43-B:

^{1}H NMR (500 MHz, $CDCl_3$): δ 7.44 (dd, *J* = 7.5, 1.5 Hz, 1H, Ar*H11'*), 7.20 (s, 1H, Ar*H7*), 6.88 (ddd, *J* = 7.9, 7.9, 1.5 Hz, 1H, Ar*H9'*), 6.72 (ddd, *J* = 7.4, 7.4, 1.1 Hz, 1H, Ar*H10'*), 6.01 (d, *J* = 8.1 Hz, Ar*H8'*), 5.85 (ddd, *J* = 2.4, 2.4, 2.4 Hz, 1H, *H6'*), 4.18 (nfom, 1H, *H12'*), 4.07 (ddd, *J* = 10.3, 9.2, 9.2 Hz, 1H, $CH_3SO_2NCH_aH_bCH_2$), 4.00 (ddd, *J* = 10.5, 9.5, 9.5 Hz, 1H, $CH_3SO_2NCH_aH_bCH_2$), 3.23 (m, 2H, NMsCH_2CH_2), 2.89 (s, 3H, CH_3SO_2N), 2.26–2.11 (m, 4H, *H2'* and *H4'*), 2.13 (s, 3H, NArCH_3), 2.09 (t, *J* = 2.9 Hz, 2H, *H13'*), 1.92 (s, 3H, NArC≡CCH_3), 1.06 [s, 3H, (CH_3)C3'], and 0.95 [s, 3H, (CH_3)C3'].

^{13}C NMR (125 MHz, $CDCl_3$): δ 196.2, 167.7, 142.5, 141.3, 140.2, 135.3, 133.4, 128.5, 127.1, 126.3, 123.1, 118.9, 114.8, 114.1, 111.6, 95.3, 85.8, 75.9, 50.7, 50.6, 42.1, 34.6, 32.2, 29.2, 28.4, 28.0, 26.1, 25.6, 16.1, and 4.7.

HRMS (APCI-Orbitrap): Calculated for $C_{30}H_{33}N_2O_4S^+$ [M+H]: 517.2156, found 517.2161.

IR (thin film): 3054, 2959, 2927, 2870, 1649, 1616, 1490, 1455, 1379, 1348, 1265, 1231, 1181, 1158, 1111, 1075, 1039, 992, 968, 911, 826, 792, 730, 701, 657, 622, 564, 542, 513, 495, and 455 cm^{-1}.

4-(Tert-butyl)-1-(5-methyl-1-(methylsulfonyl)-4-(prop-1-yn-1-yl)indolin-6-yl)-1,6-dihydro-2H-2,6-methanobenzo[d][1,3]oxazocine-5-carbonitrile atropisomer-maj (44-A) and

4-(Tert-butyl)-1-(5-methyl-1-(methylsulfonyl)-4-(prop-1-yn-1-yl)indolin-6-yl)-1,6-dihydro-2H-2,6-methanobenzo[d][1,3]oxazocine-5-carbonitrile atropisomer-min (44-B)

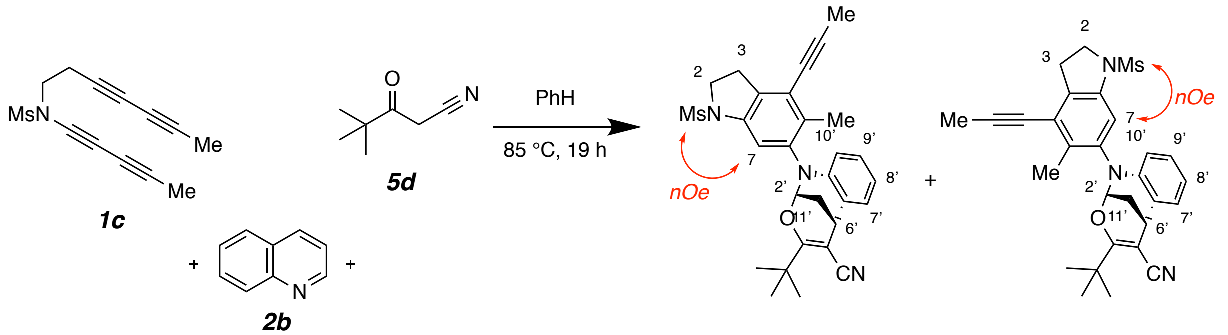

*53% (overall), **44-A** and **44-B***
1:0.56 (ratio of isomers from NMR)

Tetrayne **1c** (20 mg, 0.081 mmol), quinoline (**2b**, 20 µL, 0.162 mmol, 2 equiv), and pivaloylacetonitrile (**5d**, 50.7 mg, 0.405 mmol, 5 equiv) were combined in a culture tube, dissolved in benzene (6 mL, 0.02M), and sealed in a vial with a Teflon-lined screw-cap. The solution was heated overnight (18-19 h) in an oil bath at 85 °C, cooled, and passed through a plug of silica (1:1, Hex:EtOAc). The residue was purified by MPLC (1:1, Hex:EtOAc) to give a coeluting mixture of isomers, which also contained residual pivaloylacetonitrile (**5d**), as a transparent oil. This mixture was repurified by MPLC (2:1, Hex:EtOAc) to give coeluting mixture of atropisomeric diastereomers, **44-A** and **44-B** (22 mg, 0.044 mmol, 53% overall) as a transparent oil. There was no evidence of shouldering or other peak asymmetry in the MPLC chromatogram. The characterization spectral data were collected using the mixture of **44-A** and **44-B**. Differential nOe was used to confirm that both structures share the same constitution (i.e., represent only a single regioisomer).

^{1}H NMR for the major diastereomer, 44-A (500 MHz, $CDCl_3$): δ 7.27 (dd, *J* = 7.1, 1.9 Hz, 1H, Ar*H9'*), 7.08 (s, 1H, Ar*H7*), 6.96 (ddd, *J* = 7.8, 7.8, 1.4 Hz, 1H, ArH*7'*), 6.77 (ddd, *J* = 7.5, 7.5, 1.2 Hz, 1H, Ar*H8'*), 6.04 (dd, J = 8.2, 0.9 Hz, Ar*H10'*), 5.46 (ddd, *J* = 2.3, 2.3, 2.3 Hz, 1H, *H2'*), 4.09-3.95 (m, 2H, MsNCH_2), 3.59 (ddd, *J* = 3, 3, 3 Hz, 1H, *H6'*), 3.26–3.21 (m, 2H, $MsNCH_2CH_2$), 2.80 (s, 3H, CH_3SO_2N), 2.19 (s, 3H, $NArCH_3$), 2.15 (s, 3H, NArC≡CCH_3), 2.13 (ddd, *J* = includes 2.6, 2.6 Hz, 1H, $H11'_a$), 2.09 (ddd, *J* = 12.0, 2.4, 2.4 Hz, 1H, $H11'_b$), and 1.29 [s, 9H, $(CH_3)_3$].

^{1}H NMR of the identifiable resonances for the minor diastereomer (500 MHz, $CDCl_3$): 7.27 (dd, *J* = 6.9, 1.9 Hz, 1H, Ar*H9'*), 7.15 (s, 1H, Ar*H7*), 6.97 (ddd, *J* = 7.6, 7.6, 1.7 Hz, 1H, Ar*H7'*), 6.78 (ddd, *J* = 7.6, 7.6, 1.2 Hz, 1H, Ar*H8'*), 6.04 (dd, J = 8, 0.8 Hz, Ar*H10'*), 5.91 (ddd, *J* = 2.4, 2.4, 2.4 Hz, 1H, *H2'*), 4.09-3.95 (m, 2H, MsNCH_2), 3.61 (ddd, *J* = 3, 3, 3 Hz, 1H, *H6'*), 3.26–3.21

(m, 2H, $MsNCH_2CH_2$), 2.89 (s, 3H, CH_3SO_2N), ca. 2.2–2.14 (m, 2H, *H11'₂*), 2.13 (s, 3H, $NArCH_3$), 1.96 (s, 3H, $NArC{\equiv}CCH_3$), and 1.24 [s, 9H, $(CH_3)_3$].

Because of partial overlap of some of the proton resonances in the NMR spectrum recorded in $CDCl_3$, the spectrum was also taken in C_6D_6.

^{1}H NMR for the major diastereomer, 44-A (500 MHz, C_6D_6): δ 7.37 (s, 1H, Ar*H7*), 7.29 (dd, *J* = 7.4, 1.2 Hz, 1H, Ar*H7'*), 6.82 (ddd, *J* = 8.2, 7.5, 1.5 Hz, 1H, Ar*H9'*), 6.68 (ddd, *J* = 7.5, 7.5 1.3 Hz, 1H, ArH*8'*), 6.11 (dd, *J* = 8.4, 0.8 Hz, 1H, Ar*H10'*), 4.98 (nfom, 1H, *H2'*), 3.54–3.48 (m, 1H, $H2_a$$H2_b$), 3.42–3.33 (m, 1H, $H2_a$$H2_b$), 3.24 (ddd, *J* = 3, 3, 3 Hz, 1H, *H6'*), 2.77–2.62 (m, 2H, *H3*), 2.13 (s, 3H, CH_3SO_2N), 2.06 (s, 3H, $NArCH_3$), 1.74 (s, 3H, $NArC{\equiv}CCH_3$), 1.47 (ddd, *J* =12.9, 3.8, 3.8 Hz, 1H, *C11'*$H_a$$H_b$), 1.40 (ddd, *J* = 12.5, 2.5, 2.5 Hz, 1H, *C11'*$H_a$$H_b$), and 1.36 [s, 9H, $(CH_3)_3$]

^{1}H NMR of the identifiable resonances for the minor diastereomer, 44-B (500 MHz, C_6D_6): 7.37 (s, 1H, Ar*H7*), 7.23 (dd, *J* = 7.4, 1.4 Hz, 1H, Ar*H7'*), 6.88 (ddd, *J* = 8.3, 7.4, 1.6 Hz, 1H, Ar*H9'*), 6.70 (ddd, *J* = 8.3, 7.4, 1.1 Hz, 1H, ArH*8'*), 6.17 (dd, *J* = 8.1, 0.8 Hz, 1H, Ar*H10'*), 5.51 (ddd, *J* = 2.4, 2.4, 2.4 Hz, 1H, *H2'*), 3.54–3.48 (m, 1H, $H2_a$$H2_b$), 3.42–3.33 (m, 1H, $H2_a$$H2_b$), 3.14 (nfom, 1H, *H6'*), 2.77–2.62 (m, 2H, *H3*), 2.19 (s, 3H, CH_3SO_2N), 2.02 (s, 3H, $NArCH_3$), 1.66 (s, 3H, $NArC{\equiv}CCH_3$), 1.18 [s, 9H, $(CH_3)_3$], 1.27 (ddd, *J* =12.8, 3.5, 3.5 Hz, 1H, *C11'*$H_a$$H_b$), and 1.07 (ddd, *J* = 12.9, 2.4, 2.4 Hz, 1H, *C11'*$H_a$$H_b$)

^{13}C NMR for major diastereomer, 44-A (125 MHz, $CDCl_3$): δ 173.8, 141.5, 141.0, 140.4, 134.1, 134.0, 128.1, 127.4, 125.2, 123.4, 120.3, 119.0, 115.4, 112.3, 95.5, 86.3, 83.1, 75.8, 50.5, 38.1, 34.5, 33.5, 28.9, 28.3, 25.1, 15.4, and 4.7.

^{13}C NMR identifiable resonances for minor diastereomer, 44-B (125 MHz, $CDCl_3$): δ 173.6, 142.2, 141.3, 139.8, 134.9, 133.5, 128.1, 127.6, 124.9, 123.1, 120.5, 119.2, 113.5, 112.2, 95.6, 85.6, 85.0, 75.8, 50.6, 37.8, 34.8, 33.3, 28.9, 28.4, 25.3, 16.6, and 4.7.

HRMS (APCI-Orbitrap): Calculated for $C_{29}H_{32}N_3O_3S^+$ [$M+H^+$]: 502.2159, found 502.2153.

IR (thin film): 3042, 2971, 2918, 2873, 2200, 1735, 1595, 1491, 1457, 1398, 1349, 1315, 1266, 1241, 1158, 1137, 1111, 1083, 1061, 1024, 967, 911, 876, 844, 812, 733, 702, 654, 613, 584, 566, 543, 514, 500, and 479 cm^{-1}.

Reverse phase liquid chromatography: Only one peak corresponding to **44** was observed, consistent with the assumption that the atropisomers are interconverting sufficiently rapidly to coeluted.

(e) Products obtained from triflate salt formations and their functionalizations

1-(6,7-Dimethoxy-2-methyl-9-oxo-1-(trimethylsilyl)-9H-fluoren-3-yl)pyridin-1-ium triflate (45)

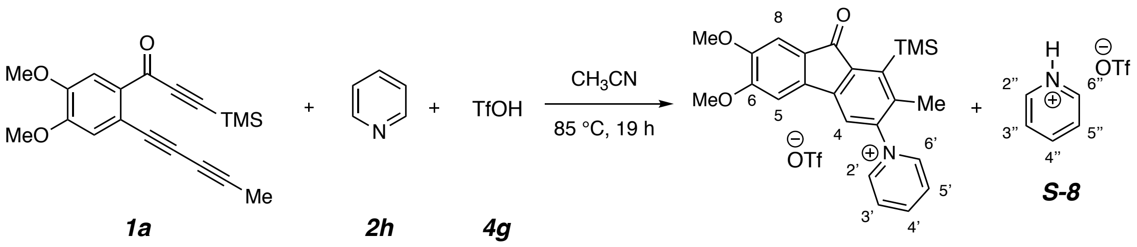

1a *2h* *4g* *45* *S-8*

Triynone **1a** (100 mg, 0.308 mmol) and pyridine (**2h**, 80 μL, 0.924 mmol, 3 equiv) were combined in a culture tube and dissolved in CH_3CN (20 mL). Triflic acid (**4g**, 54, μL, 0.616 mmol, 2 equiv) was added to this solution, and culture tube was sealed with a Teflon-lined screw cap. The solution was heated overnight (16 h) in an oil bath at 85 °C, cooled, and concentrated to give a brown colored mixture of **45**, **S-8**, and residual pyridine (**2h**). This mixture was heated under vacuum (~0.1 torr) at 50 °C to provide a mixture of **45** and **S-8** as a pale brown powder.

Data for 45:

^{1}H NMR (500 MHz, DMSO): δ 9.33 (dd, J = 6.6, 1.3 Hz, 2H, Ar*H2'*), 8.89 (tt, J = 8.0, 1.7 Hz, 1H, Ar*H4'*), 8.41 (dd, J = 7.7, 6.5 Hz, 2H, Ar*H3'*), 7.98 (s, 1H, Ar*H4*), 7.41 (s, 1H, Ar*H5*), 7.21 (s, 1H, Ar*H8*), 3.89 (s, 3H, OCH_3), 3.85 (s, 3H, OCH_3), 2.08 (s, 3H, $ArCH_3$), and 0.43 [s, 9H, $Si(CH_3)_3$].

^{13}C NMR (125 MHz, DMSO): δ 192.6, 155.2, 150.2, 147.6, 145.8, 145.7, 145.5, 143.1, 143.1, 141.8, 137.8, 136.9, 125.4, 119.1, 107.2, 104.5, 56.2, 55.9, 18.9, and 2.6.

HRMS (ESI-TOF): Calculated for $C_{24}H_{26}NO_3Si^+$ [M]: 404.1676, found 404.1673.

Data for S-8:

^{1}H NMR (500 MHz, DMSO): δ 8.92 (dd, J = 6.8, 1.7 Hz, 1H, Ar*H6"*), 8.57 (tt, J = 7.9, 1.8 Hz, 1H, Ar*H4"*), and 8.05 (dd, J = 7.6, 6.6 Hz, 2H, Ar*H3"*).

^{13}C NMR (125 MHz, DMSO): δ 142.7, 128.6, and 127.0.

6,7-Dimethoxy-2-methyl-3-(piperidin-1-yl)-1-(trimethylsilyl)-9H-fluoren-9-one (49)

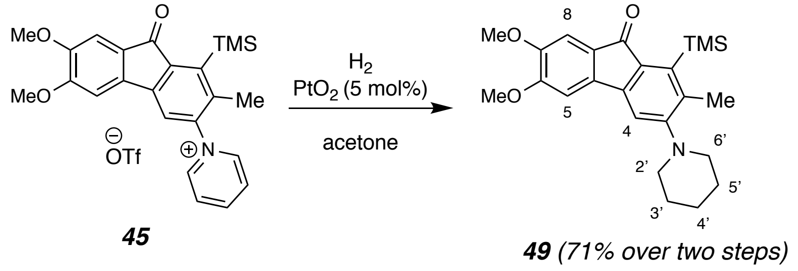

An oven dried 20 mL culture tube having a magnetic stir bar was evacuated with vacuum and purged with N_2 gas. Pyridinium triflate salt **45** (25 mg, 0.045 mmol, 1 equiv) was added, and the salt was dissolved in 5 mL of acetone. Adams catalyst (0.5 mg, 5 mol%) was added, and this solution was stirred under one atmosphere of H_2 at room temperature. The reaction progress was monitored by crude mass spectrometric analysis. After the consumption of the starting material (ca. 2 h), the reaction mixture was filtered through Celite® and concentrated under reduced pressure. The crude residue was dissolved in DCM and washed with sat. aq. $NaHCO_3$. The aqueous phase was washed with DCM (3x, ~30 mL). The combined organic layers were dried over $MgSO_4$, filtered, and concentrated under vacuum. This residue was passed through a plug of silica gel (100 % EtOAc) to obtain **49** (13 mg, 71% yield) as an orange colored crystalline powder.

Data for 49:

^{1}H NMR (500 MHz, $CDCl_3$): δ 7.11 (s, 1H, Ar*H8*), 7.02 (s, 1H, Ar*H4*), 6.92 (s, 1H, Ar*H5*), 4.01 (s, 3H, OCH_3), 3.90 (s, 3H, OCH_3), 2.95 (br t, J = 4.7 Hz, 4H, *H2'* and *H6'*), 2.38 (s, 3H, ArCH_3), 1.74 (pent, J = 5.9 Hz, 4H, *H3'* and *H5'*), 1.62 (br pent, J = 6.8 Hz, 2H, *H4'*), and 0.42 [s, 9H, $Si(CH_3)_3$].

^{13}C NMR (125 MHz, $CDCl_3$): δ 194.1, 157.7, 154.0, 149.4, 144.0, 143.5, 138.7, 137.0, 134.2, 127.5, 110.8, 106.8, 102.6, 56.5, 56.3, 53.3, 26.5, 24.5, 20.3, and 2.8.

HRMS (APCI-Orbitrap): Calculated for $C_{24}H_{32}NO_3Si^+$ [M+H$^+$]: 410.2146, found 410.2143.

IR (thin film): 2973, 2937, 2845, 1692, 1585, 1544, 1495, 1459, 1411, 1385, 1357, 1311, 1242, 1220, 1150, 1135, 1090, 1045, 1017, 1000, 856, 797, 759, 733, 701, 676, 636, 607, 584, 542, 456, and 415 cm^{-1}.

mp: 162-165 °C

9-(6,7-Dimethoxy-2-methyl-9-oxo-1-(trimethylsilyl)-9*H*-fluoren-3-yl)-2-methyl-3a,4,7,7a-tetrahydro-1*H*-4,7-(epiminomethano)isoindole-1,3(2*H*)-dione (50):

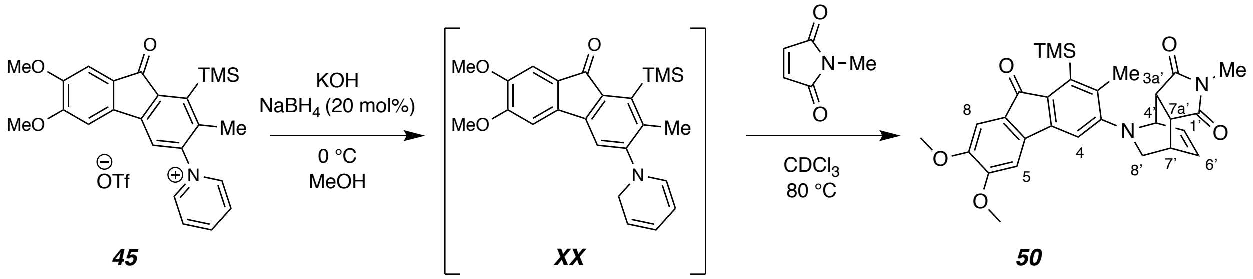

An oven dried 30 mL culture tube having a magnetic stir bar was evacuated with vacuum and purged with N_2 gas. Pyridinium triflate salt **45** (25 mg, 0.045 mmol, 1 equiv) was added, and the salt was dissolved in 8 mL of methanol. The solution was cooled to 0 °C, and powdered potassium hydroxide (10 mg, 0.181 mmol, 2 equiv) and $NaBH_4$ (0.700 mg, 20 mol%) were added in sequence. The reaction mixture was warmed to room temperature and stirred under a nitrogen atmosphere until the reaction was judged to completed by crude mass spectrometric analysis (ca. 2 h). The solution was concentrated under reduced pressure to obtain a crude material, which was dissolved in EtOAc and washed with sat. aq. $NaHCO_3$ (10 mL). The aqueous phase was extracted with EtOAc (3x, ~30 mL). The combined organic layers were dried over $MgSO_4$, filtered, and concentrated under vacuum to obtain a crude product (**XX**). This dihydropyridine **50** showed signs of decomposition upon storage and handling and, therefore, was directly used for the next step without chromatographic purification.

The crude product **XX** and *N*-methylmaleimide (**3i**, 25 mg, 0.225 mmol, 5 equiv) were combined in a culture tube, dissolved in $CDCl_3$ (4 mL), and sealed with a Teflon-lined screw cap. The solution was heated overnight (12 h) in an oil bath at 80 °C, cooled, and passed through a plug of silica (1:1, Hex:EtOAc). The residue was purified by MPLC (1:1, Hex:EtOAc) to give **50** (10 mg, 43%, 0.019 mmol) as a yellow oil. A small portion of this material was separately repurified by HPLC (1:1, Hex:EtOAc).

Data for Diels–Alder adduct 50:

^{1}H NMR (500 MHz, $CDCl_3$): δ 7.11 (s, 1H, Ar*H8*), 6.90 (s, 1H, Ar*H5* or Ar*H4*), 6.88 (s, 1H, Ar*H5* or Ar*H4*), 6.66 (ddd, *J* = 8.2, 5.3, 1.5 Hz, 1H, *H1'* or *H6'*), 6.40 (ddd, *J* = 8.0, 6.4, 1.4 Hz, 1H, *H1'* or *H6'*), 4.62 (ddd, *J* = 5.4, 4.1, 1.4 Hz, 1H, *H4'*), 4.03 (s, 3H, OCH_3), 3.90 (s, 3H, OCH_3), 3.62 (dd, *J* = 9.6, 1.8 Hz, 1H, *H8'*), 3.53 (dd, *J* = 8.0, 4.1 Hz, 1H, *H3a'*), 3.39–3.36 (m, Σ*J* = 15.8 Hz (which accommodates the following values that should be within this resonance: 6.4, 3.1, 2.6, 1.8, and 1.5 seen in each of five other resonances coupled to this bridgehead proton) 1H, *H7'*), 3.13 (dd, *J* = 8.0, 3.1 Hz, 1H, *H7a'*), 2.60 (dd, *J* = 9.7, 2.6 Hz, 1H, *H8'*), 2.95 (s, 3H, NCH_3), 2.32 (s, 3H, ArCH_3), and 0.41 [s, 9H, Si(CH_3)$_3$].

^{13}C NMR (125 MHz, $CDCl_3$): δ 193.7, 178.1, 177.0, 155.5, 154.1, 149.7, 144.6, 143.8, 138.2, 135.4, 134.6, 132.6, 132.0, 127.6, 111.0, 106.8, 102.5, 56.6, 56.3, 52.6, 52.2, 46.4, 41.7, 33.7, 25.0, 22.3, and 2.6.

HRMS (APCI-Orbitrap): Calculated for $C_{29}H_{33}N_2O_5Si^+$ [M+H$^+$]: 517.2153, found 517.2147.

IR (thin film): 3057, 2942, 2900, 2873, 1775, 1694, 1585, 1545, 1493, 1465, 1437, 1412, 1383, 1353, 1313, 1269, 1243, 1214, 1151, 1127, 1094, 1018, 1001, 914, 843, 796, 755, 728, 700, 616, 602, 582, 557, 517, and 433 cm^{-1}.

1-(5-Methyl-1-(methylsulfonyl)-4-(prop-1-yn-1-yl)indolin-6-yl)pyridin-1-ium triflate (46):

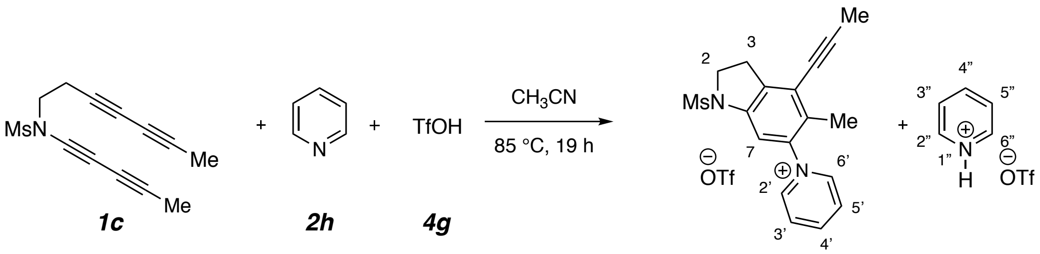

1c ***2h*** ***4g*** ***46*** ***S-8***

In a 20 mL culture tube, pyridine (**2h**, 65 μL, 0.982 mmol, 4 equiv) was dissolved in CH_3CN (8 mL). Triflic acid (**4g**, 27 µL, 0.303 mmol, 1.5 equiv) was added and then tetrayne **1c** (50 mg, 0.202 mmol, 1 equiv) were added to this solution. The culture tube was sealed with a Teflon-lined screw cap. The solution was heated overnight (16 h) in an oil bath at 85 °C, cooled, and concentrated to give a dark brown colored sticky solid. This mixture was heated under vacuum (~0.1 torr) at 50 °C to remove excess pyridine and provide the residual pyridinium triflate salts **46** and **S-8**.

Data for 46:

^{1}H NMR (500 MHz, CD_3CN): δ 8.79 (dd, J = 6.4, 1.4 Hz, 2H, Ar*H2'*), 8.72 (tt, J = 7.9, 1.5 Hz, 1H, Ar*H4'*), 8.21 (dd, J = 7.7, 6.4 Hz, 2H, Ar*H3'*), 7.37 (s, 1H, Ar*H7*), 4.08 (t, J = 8.6 Hz, 2H, *H2*), 3.27 (t, J = 8.7 Hz, 2H, *H3*), 2.97 (s, 3H, CH_3SO_2N), 2.14 (s, 3H, $NArCH_3$), and 2.06 (s, 3H, $C{\equiv}CCH_3$).

^{13}C NMR (125 MHz, CD_3CN): δ 148.3, 146.9, 146.8, 143.8, 142.1, 139.1, 130.1, 129.1, 128.1, 98.6, 75.2, 51.2, 35.5, 28.9, 15.4, and 4.5.

HRMS (ESI-TOF): Calculated for $C_{18}H_{19}N_2O_2S^+$ [M]: 327.1162, found 327.1158.

Data for S-8:

^{1}H NMR (500 MHz, CD_3CN): δ 10.42 [s, 1H, (pyr)$^+$-*H*], 8.73 (dd, J = 6.4, 1.5 Hz, 1H, Ar*H6"*), 8.49 (tt, J = 8.0, 1.6 Hz, 1H, Ar*H4"*), and 7.96 (dd, J = 7.7, 6.4 Hz, 2H, Ar*H3"*).

^{13}C NMR (125 MHz, CD_3CN): δ 129.6, 118.4, and 110.8.

2,2,2-Trifluoro-1-(6-methyl-1-(5-methyl-1-(methylsulfonyl)-4-(prop-1-yn-1-yl)indolin-6-yl)-1,6-dihydropyridin-3-yl)ethan-1-one (51)

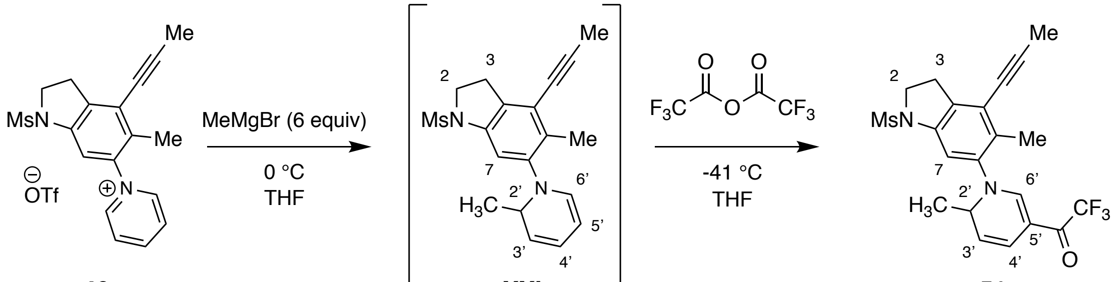

46 *XXI* *51*

In a 100 mL round bottom flask, crude salt **46** was added. This salt was dissolved in 15 mL of THF, and then cooled to 0 °C using an ice bath. Methylmagnesium bromide (0.67 mL, 3M solution in THF, 6 equiv, 2.02 mmol) was added dropwise over 10 minutes. The reaction mixture was stirred for two hours at 0 °C. Formation of the product was confirmed by TLC and mass spectrometric analysis Satd. aq. $NaHCO_3$ (10 mL) and diethyl ether (10 mL) were added. The aqueous phase was extracted with diethyl ether (3x, ~45 mL). The combined organic layers were dried over $MgSO_4$, filtered, and concentrated under vacuum to obtain the crude product mixture containing the dihydropyridine (**XXI**). **XXI** proved to be very sensitive towards silica gel chromatography. This compound was carried into the next reaction without purification.

^{1}H NMR data for the acid sensitive intermediate XXI:

^{1}H NMR (500 MHz, $CDCl_3$): δ 7.24 (s, 1H, Ar*H7*), 6.03 (d, *J* = 7.2 Hz, 1H, *H6'*), 5.94 (dd, *J* = 9.3, 5.5 Hz, 1H, *H4'*), 5.17 (dd, *J* = 9.2, 5.3 Hz, 1H, *H3'*), 4.86 (ddd, *J* = 7.0, 5.5, 1.3 Hz, 1H, Ar*H5'*), 4.31 (pent, *J* = 6.3 Hz, 1H, *H2'*), 4.01 (ddd, *J* = 10.3, 10.3, 8.2 Hz, 1H, $CH_3SO_2NCH_aH_bCH_2$), 3.95 (ddd, *J* = 10.8, 10.8, 8.3 Hz, 1H, $CH_3SO_2NCH_aH_bCH_2$), 3.15 (t, *J* = 8.4 Hz, 2H, $NMsCH_2CH_2$), 2.85 (s, 3H, CH_3SO_2N), 2.35 (s, 3H, $NArCH_3$), 2.13 (s, 3H, $C\equiv CCH_3$), and 1.06 [d, *J* = 6.3 Hz, 3H, $N(CH)CH_3$].

Crude **XXI** was dissolved in THF (15 mL), cooled to -41 °C, and trifluoroacetic anhydride (40 μL, 0.283 mmol, 1.4 equiv) was added. This solution was warmed to room temperature. Saturated Na_2CO_3 (10 mL) was added and the mixture was extracted with DCM (3x, ~45 mL). The combined organic layers were washed with brine (20 mL), dried over $MgSO_4$, filtered, and concentrated under vacuum to obtain the crude trifluoromethyl ketone **51**. This was passed through a plug of silica (1:1, Hex:EtOAc). The residue was purified by MPLC (1:1, Hex:EtOAc) to give impure **51** as a yellow oil. This impure product was repurified by MPLC (2:1, Hex:EtOAc) to give pure **51** (50 mg, 0.114 mmol, 56% overall yield) as a bright yellow oil.

Data for 51:

^{1}H NMR (500 MHz, $CDCl_3$): δ 7.36 (br s, 1H, Ar*H6'*), 7.23 (s, 1H, Ar*H7*), 6.58 (br d, *J* = 9.7 Hz, *H4'*), 5.35 (br d, *J* = 9.8 Hz, *H3'*), 4.59 (br s, 1H, *H2'*), 4.10–4.00 (m, 2H, CH_3SO_3N*CH_2*), 3.24 (t, *J* = 8.6 Hz, 2H, $NMsCH_2$*CH_2*), 2.93 (s, 3H, CH_3SO_2N), 2.35 (s, 3H, $NArCH_3$), 2.17 (s, 3H, C≡C*H_3*), and 1.26 [d, *J* = 6.4 Hz, 3H, N(CH)*CH_3*].

^{13}C NMR (126 MHz, $CDCl_3$): δ 151.5 (*C6'*), 142.6, 140.8, 134.7, 131.5, 123.9, 118 (q, CF_3, *J* = 290 Hz), 119.0 (*C4'*), 118.7 (*C3'*, from HSQC), 116.8, 111.0 (*C7*, from HSQC), 96.5, 75.3, 57.3 (C2'), 50.4, 35.2, 28.3, 21.8, 15.7, and 4.7. (resonance for the ketone carbonyl carbon not observed)

HRMS (APCI-Orbitrap): Calculated for $C_{21}H_{22}F_3N_2O_3S^+$ [M+H$^+$]: 439.1295, found 439.1298.

IR (thin film): 3058, 2973, 2924, 2853, 2232, 1633, 1597, 1559, 1534, 1447, 1415, 1347, 1323, 1294, 1219, 1182, 1157, 1127, 1022, 965, 908, 872, 797, 756, 727, 663, 614, 568, 543, 513, and 426 cm^{-1}.

2-(5-Methyl-1-(methylsulfonyl)-4-(prop-1-yn-1-yl)indolin-6-yl)-1-vinyl-1,2-dihydroisoquinoline (52):

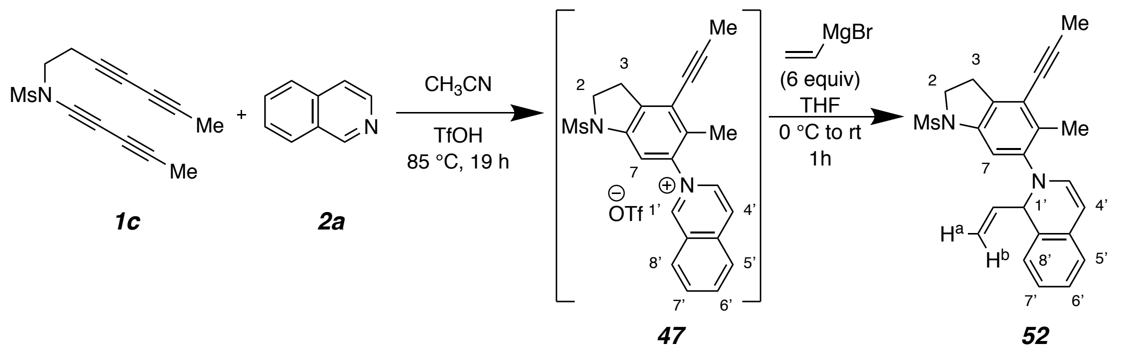

In a 20 mL culture tube, isoquinoline (**2a**, 42 μL, 0.363 mmol, 3 equiv) was dissolved in CH_3CN (8 mL). Triflic acid (**4g**, 16 μL, 0.182 mmol, 1.5 equiv) was added to this solution, followed by the addition of tetrayne **1c** (30 mg, 0.121 mmol, 1 equiv). The culture tube was sealed with a Teflon-lined screw cap. This solution was heated overnight (16 h) in an oil bath at 85 °C, cooled, and concentrated to give a red colored sticky solid **47**. The formation of the salt **47** was confirmed by ^{1}H NMR and HRMS analysis.

NMR and HRMS analysis of the intermediate salt, 47:

^{1}H NMR (500 MHz, DMSO): δ 10.22 (s, 1H, Ar*H1'*), 8.87 (d, J = 6.8 Hz, 1H, Ar*H3'* or Ar*H4'*), 8.73 (d, J = 6.8 Hz, 1H, Ar*H3'* or Ar*H4'*), 8.56 (d, J = 8.2 Hz, 1H, Ar*H8'* or Ar*H5'*), 8.46 (d, J = 8.2 Hz, 1H, Ar*H8'* or Ar*H5'*), 8.37 (t, J = 8.1 Hz, 1H, Ar*H6'*), 8.16 (t, J = 7.5 Hz, 1H, Ar*H7'*), 7.55 (s, 1H, Ar*H7*), 4.09 (t, J = 8.6 Hz, 2H, *H2*), 3.26 (t, J = 8.6 Hz, 2H, *H3*), 3.15 (s, 3H, CH_3SO_2N), 2.19 (s, 3H, $NArCH_3$), and 2.12 (s, 3H, $C{\equiv}CCH_3$).

HRMS (ESI-TOF): Calculated for $C_{22}H_{21}N_2O_2S^+$ [M]: 377.1318, found 377.1317.

In a 100 mL round bottom flask, the triflate salt **47** was dissolved in 15 mL of THF and cooled to 0 °C using an ice bath. Vinylmagnesium bromide (0.73 mL, 1 M solution in THF, 6 equiv, 0.726 mmol) was added dropwise over 10 minutes. The reaction mixture was stirred for one hour at 0 °C. Formation of the product was confirmed by TLC and mass spectrometric analysis. Satd. aq. $NaHCO_3$ (10 mL) EtOAc (10 mL) were added. The aqueous phase was washed with EtOAc (3x, ~30 mL). The combined organic layers were dried over $MgSO_4$, filtered, and concentrated under vacuum. This crude product mixture was passed through a plug of silica (1:1, Hex:EtOAc). The residue was purified by MPLC (2:1, Hex:EtOAc) to give **52** (39 mg, 79%, 0.096 mmol) as an orange oil, which solidified into an amorphous solid in the freezer (-10 °C).

Data for 52:

^{1}H NMR (500 MHz, $CDCl_3$): δ 7.30 (s, 1H, Ar*H7*), 7.17 (ddd, *J* = 8.8, 7.5, 1.5 Hz, 1H, Ar*H6'*), 7.07 (ddd, *J* = 8.8, 7.5, 1.3 Hz, 1H, Ar*H7'*), 6.99 (br d, *J* = 7.4 Hz, 1H, Ar*H8'*), 6.97 (br d, *J* = 7.3 Hz, 1H, Ar*H5'*), 6.24 (d, *J* = 7.4 Hz, 1H, *H3'*), 6.11 (ddd, *J* = 17.3, 10.3, 7.3 Hz, 1H, =C*H*C1'), 5.52 (d, *J* = 7.4 Hz, 1H, *H4'*), 5.04 (d, *J* = 7.5 Hz, 1H, *H1'*), 4.93 (d, 1H, *J* = 10.1 Hz, H^a), 4.91 (d, 1H, *J* = 17.1 Hz, H^b), 4.00 (ddd, *J* = 10.4, 10.4, 8.5 Hz, 1H, $CH_3SO_2NCH_aH_bCH_2$), 3.98 (ddd, *J* = 10.7, 10.7, 8.8 Hz, 1H, $CH_3SO_2NCH_aH_bCH_2$), 3.16 (t, *J* = 8.5 Hz, 2H, $NMsCH_2CH_2$), 2.83 (s, 3H, CH_3SO_2N), 2.32 (s, 3H, $NArCH_3$), and 2.12 (s, 3H, C≡CH_3).

^{13}C NMR (125 MHz, $CDCl_3$): δ 145.6, 140.0, 136.3, 134.9, 132.3, 131.3, 131.1, 129.1, 127.8, 126.3, 125.5, 123.4, 122.8, 115.0, 112.3, 99.9, 94.8, 76.1, 65.6, 50.6, 34.4, 28.2, 16.4, and 4.7.

HRMS (APCI-Orbitrap): Calculated for $C_{24}H_{25}N_2O_2S^+$ [M+H$^+$]: 405.1631, found 405.1635.

IR (thin film): 3057, 3008, 2919, 2853, 2233, 1676, 1656, 1626, 1594, 1454, 1344, 1279, 1267, 1234, 1155, 1113, 1061, 966, 906, 790, 731, 697, 659, 569, 544, and 514 cm^{-1}.

1-Methyl-2-(5-methyl-1-(methylsulfonyl)-4-(prop-1-yn-1-yl)indolin-6-yl)-1,2-dihydrophthalazine (53)

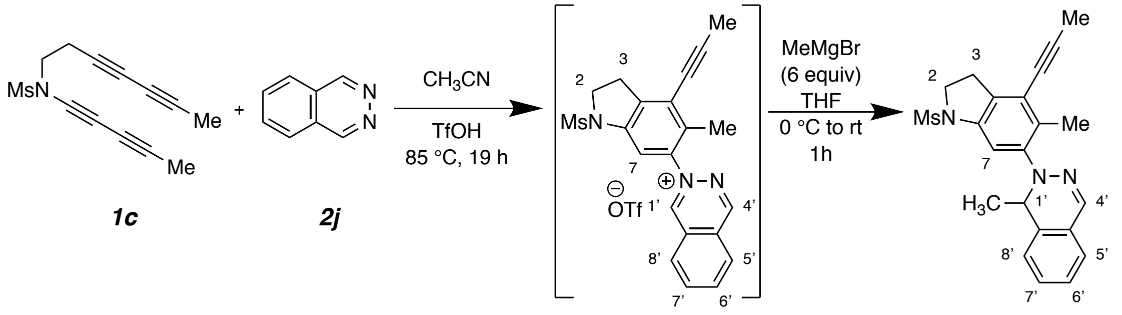

1c ***2j*** ***48*** ***53***

In a 20 mL culture tube, phthalazine (**2j**, 39 mg, 0.303 mmol, 3 equiv) was dissolved in CH_3CN (8 mL). Triflic acid (**4g**, 14 μL, 0.152 mmol, 1.5 equiv) was added followed by the tetrayne **1c** (25 mg, 0.101 mmol, 1 equiv). The culture tube was sealed with a Teflon-lined screw cap. This solution was heated overnight (16 h) in an oil bath at 85 °C, cooled, and concentrated to give a red colored sticky solid. The formation of the salt **48** was confirmed by the ^{1}H NMR spectrum of this crude material and further supported by HRMS analysis.

Data for salt 48:

^{1}H NMR (500 MHz, DMSO): δ 10.89 (s, 1H, Ar*H1'*), 10.20 (s, 1H, Ar*H4'*), 8.70 (d, *J* = 8.1 Hz, 1H, Ar*H8'* or Ar*H5'*), 8.67 (d, *J* = 8.2 Hz, 1H, Ar*H8'* or Ar*H5'*), 8.62 (dd, *J* = 8.0, 8.0 Hz, 1H, Ar*H7'* or Ar*H6'*), 8.49 (dd, *J* = 7.9, 7.9 Hz, 1H, Ar*H7'* or Ar*H6'*), 7.65 (s, 1H, Ar*H7*), 4.09 (t, *J* = 8.5 Hz, 2H, *H2*), 3.27 (t, *J* = 8.5 Hz, 2H, *H3*), 3.12 (s, 3H, CH_3SO_2N), 2.21 (s, 3H, $NArCH_3$), and 2.19 (s, 3H, $C{\equiv}CCH_3$).

HRMS (ESI-TOF): Calculated for $C_{21}H_{20}N_3O_2S^+$ [M]: 378.1271, found 378.1268.

In a 20 mL culture tube, the crude salt **48** was partially dissolved in THF (10 mL) to give a heterogeneous mixture. This was cooled to 0 °C using an ice bath, and MeMgBr (0.2 mL, 3M solution in ether, 6 equiv, 0.152 mmol) was added dropwise over 10 minutes. After the addition, the reaction mixture became less heterogeneous and turned to a bright red color. The ice bath was removed, and the reaction mixture was stirred for one hour. The formation of the product was confirmed by both thin layer chromatography and crude mass spectrometric analysis. The reaction mixture was washed with satd aq $NaHCO_3$ (10 mL) and diluted with EtOAc (10 mL). The aqueous phase was washed with EtOAc (3x, ~30 mL). The combined organic layers were dried over $MgSO_4$, filtered, and concentrated under vacuum to obtain a crude product **53**. This was passed through a plug of silica (1:1, Hex:EtOAc). The residue was purified by MPLC (1:1, Hex:EtOAc) to give **53** (23 mg, 58%, 0.058 mmol) as an orange oil.

Data for 53:

^{1}H NMR (500 MHz, $CDCl_3$): δ 7.56 (s, 1H, Ar*H4'*), 7.52 (s, 1H, Ar*H7*), 7.37 (ddd, *J* = 8.7, 7.4, 1.2 Hz, 1H, Ar*H6'*), 7.33 (ddd, *J* = 8.6, 7.5, 1.4 Hz, 1H, Ar*H7'*), 7.21 (d, *J* = 7.4 Hz, 1H, Ar*H8'*), 7.08 (d, *J* = 7.3 Hz, 1H, Ar*H5'*), 4.65 (q, *J* = 6.5 Hz, 1H, *H1'*), 4.04 (ddd, *J* = 9.9, 9.9, 7.1 Hz, 1H, $CH_3SO_2NCH_aH_bCH_2$), 3.95 (ddd, *J* = 9.4, 9.4, 7.9 Hz, 1H, $CH_3SO_2NCH_aH_bCH_2$), 3.23–3.12 (m, 2H, $NMsCH_2CH_2$), 2.88 (s, 3H, CH_3SO_2N), 2.36 (s, 3H, $NArCH_3$), 2.13 (s, 3H, $C{\equiv}CCH_3$), and 1.09 [d, *J* = 6.6 Hz, 3H, N(CH)CH_3].

^{13}C NMR (125 MHz, $CDCl_3$): 146.8, 140.1, 137.7, 134.8, 131.0, 130.4, 128.8, 128.0, 124.85, 124.84, 124.4, 122.4, 112.5, 94.4, 76.3, 55.3, 50.7, 34.5, 28.2, 16.01, 15.99, and 4.7.

HRMS (APCI-Orbitrap): Calculated for $C_{22}H_{24}N_3O_2S^+$ [M+H$^+$]: 394.1584, found 394.1580.

IR (thin film): 3056, 2973, 2854, 2229, 1733, 1591, 1450, 1346, 1320, 1265, 1156, 1099, 1070, 1038, 1001, 964, 909, 875, 804, 730, 702, 652, 627, 595, 567, 541, 513, 478, and 411 cm^{-1}.

(f) Product obtained from carbene capture with *p*-bromobenzaldehyde (Endnote #17):

3-((4-Bromophenyl)(pyridin-2-yl)methoxy)-6,7-dimethoxy-2-methyl-1-(trimethylsilyl)-9H-fluoren-9-one (v) (Endnote #17)

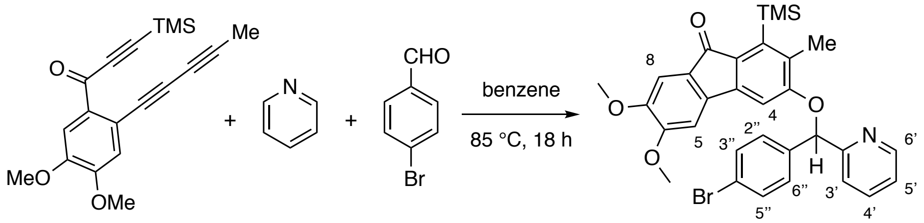

1a *2h* *3e* *v (53%)*

An oven-dried, 25 mL culture tube having a magnetic stir bar was evacuated and purged with N_2 gas. Triynone **1a** (30 mg, 0.093 mmol, 1 equiv) and *p*-bromobenzaldehyde (**3e**, 86 mg, 0.463 mmol, 5 equiv) were added and the headspace was refilled with N_2. Benzene (8 mL, 0.01M) was added and nitrogen was bubbled through the solution. Pyridine (**2h**, 23 μL, 0.277, 3 equiv) was added and the culture tube was sealed with a Teflon-lined screw cap. The solution was heated for 18 h in an oil bath at 85 °C, cooled, and passed through a plug of silica (EtOAc eluant). The residue was purified by MPLC (1:1, Hex:EtOAc) to give **v** (29 mg, 0.049 mmol, 53%) as an orange oil.

^{1}H NMR (500 MHz, $CDCl_3$): δ 8.60 (ddd, *J* = 4.9, 1.7, 0.9 Hz, 1H, *H6'*), 7.71 (ddd, *J* = 7.7, 7.7, 1.7 Hz, 1H, *H4'*), 7.51 (nfod, *J* = 8.5 Hz, 2H, *H2''/H6''* or *H3''/H5''*), 7.51 (overlapped d, 1H, *H3'*), 7.45 (nfod, *J* = 8.5 Hz, 2H, *H2''/H6''* or *H3''/H5''*), 7.23 (ddd, *J* = 7.5, 4.9, 1.1 Hz, 1H, *H5'*), 7.07 (s, 1H, Ar*H8*), 6.84 (s, 1H, Ar*H4*), 6.75 (s, 1H, Ar*H5*), 6.47 (s, 1H, C*H*OAr), 3.97 (s, 3H, C6OCH_3), 3.88 (s, 3H, C7OCH_3), 2.47 (s, 3H, ArCH_3), and 0.43 [s, 9H, Si(CH_3)$_3$].

^{13}C NMR (125 MHz, $CDCl_3$): δ 193.6, 160.3, 159.2, 154.0, 149.7, 149.3, 145.1, 143.5, 139.1, 137.9, 137.8, 133.1, 132.3, 132.0, 128.2, 127.3, 123.3, 122.3, 120.6, 106.8, 105.2, 102.8, 82.0, 56.6, 56.3, 17.4, and 2.8.

IR (neat): 3054, 3002, 2937, 2901, 2841, 1700, 1585, 1558, 1493, 1467, 1391, 1355, 1311, 1230, 1216, 1147, 1087, 1010, 993, 865, 841, 796, 766, 733, 632, 596, and 537 cm^{-1}.

HRMS (APCI-Orbitrap): Calculated for $C_{31}H_{30}{}^{79}BrNNaO_4Si^+$ [M+Na$^+$] 610.1020, found 610.1023.

III. Discussion of Computational Results

The Gaussian 09 software package was used to perform the DFT computations.[7] The geometries were optimized using the M06-2X functional;[8] the double-ζ split-valence 6-311+G(d, p) basis set was used. The SMD solvation model[9] with benzene as solvent was applied during the both frequency calculation as well as the geometry optimization. Harmonic vibrational frequency calculations were done at 298 K and were used for thermal correction of enthalpies. The "Sum of electronic and thermal Free Energies=" value was used as the free energy (G) of the transition state structures as well as that of the reactants and products. Each optimized transition structure geometry showed only one imaginary frequency.

NMR chemical shift calculations were performed following a reported protocol[10] at the following level of theory: SMD(chloroform)/B3LYP/6-311+G(2d,p)//M062X/6-31+G(d,p). The shifts for model benzotriazocine **S-9** and benzodiazocines **S-10** and **S-11** were examined. The chemical shifts for the key proton and carbon resonances within the eight membered ring of each were shown to be a good match between the data for **10** and structure **S-9** and the data for **11** matched far better (and quite well on an absolute basis) with the structure **S-10** rather than its regioisomeric analog **S-11**.

Energies and Geometries of the species in Figures 2b, S1, and S2.

Figure 2b (repeated from manuscript).

3,6-dimethylbenzyne (**VII**)

isoquinoline (**2a**)

zwitterion **VIII**

TS$_{\mathbf{protonshift}}$

carbene **X**

TS$_{\mathbf{ringclosing}}$

azetidine **IX**

TS$_{\mathbf{ringopening}}$

benzoazocine **XI**

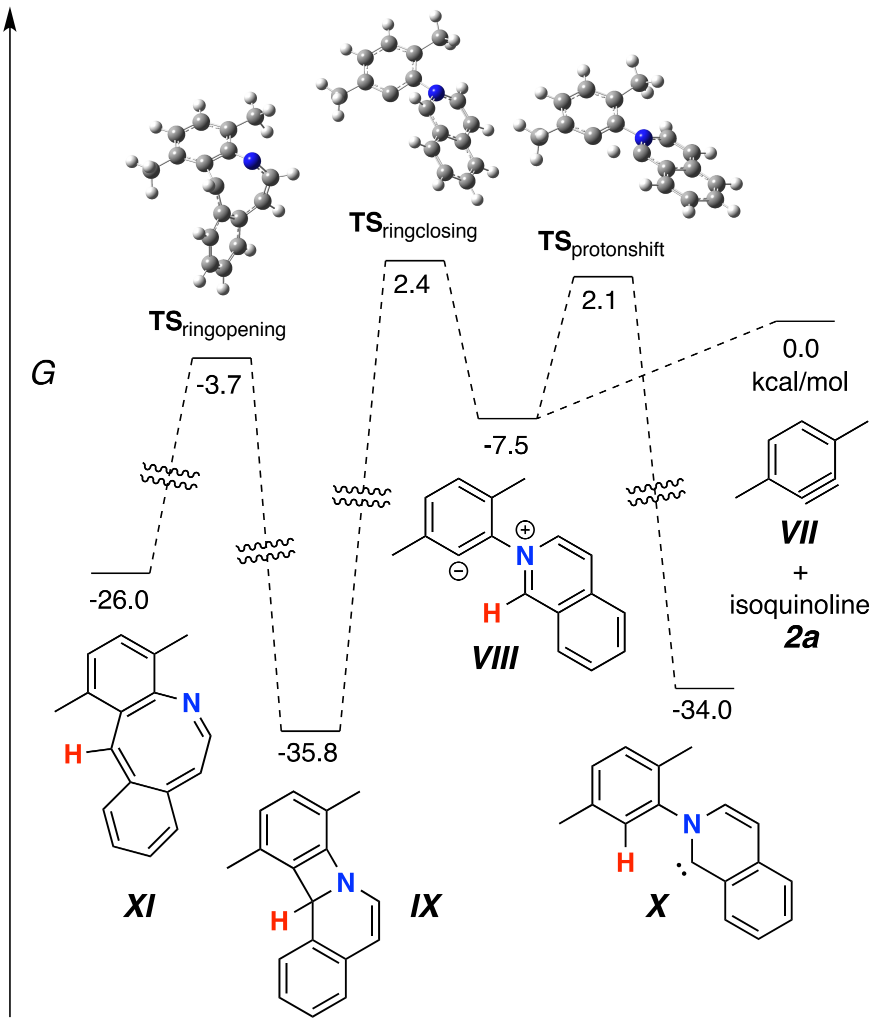

Figure S1. DFT computed PES for reaction of quinoline (2b) and model benzyne VII.

3,6-dimethylbenzyne (**VII**)

quinoline (**2b**)

quinoline zwitterion **SI**

TS$_{quinolinecarbene}$

quinoline carbene **SII**

TS$_{quinolineringclosing}$

quinoline azetidine **SIII**

TS$_{quinolineringopening}$

quinoline azocine **SIV**

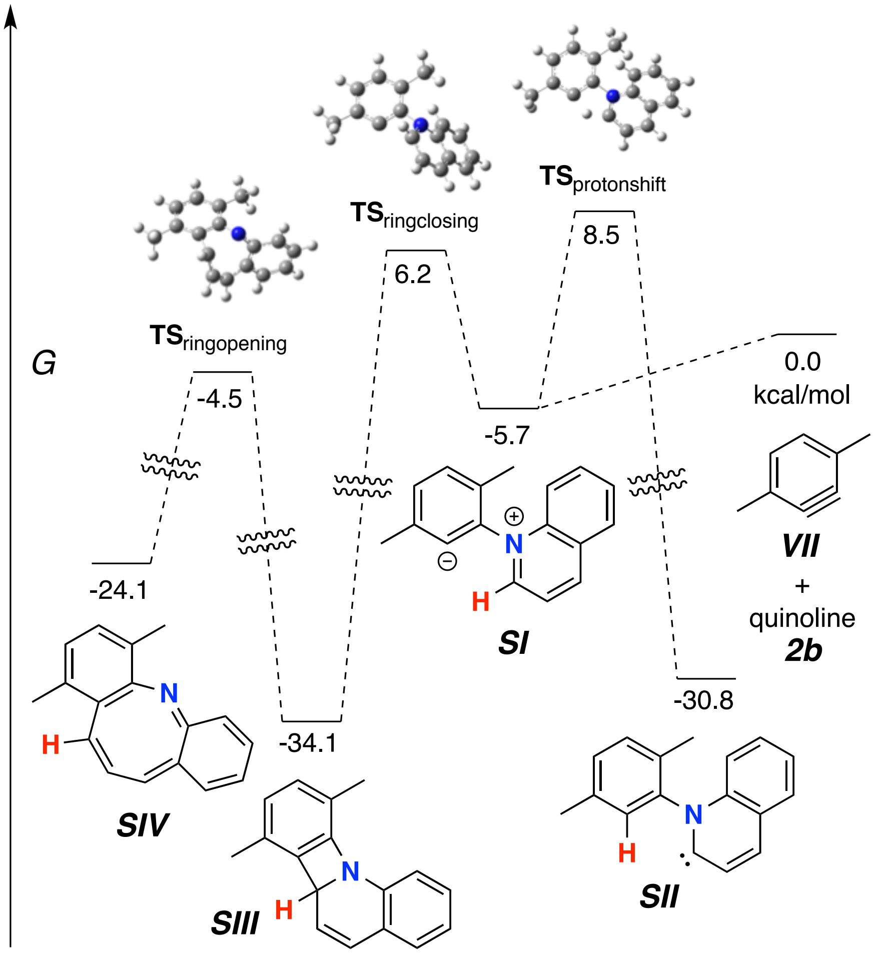

Figure S2. DFT computed PES for reaction of 2,4,6-triazine (2d) and model benzyne VII.

3,6-dimethylbenzyne (**VII**)

triazine (**2d**)

triazine zwitterion **SV**

TS$_{\mathbf{triazinecarbene}}$

triazine carbene **SVI**

TS$_{\mathbf{triazineringclosing}}$

triazine azetidine **SVII**

TS$_{\mathbf{triazineringopening}}$

triazine azocine **SVIII**

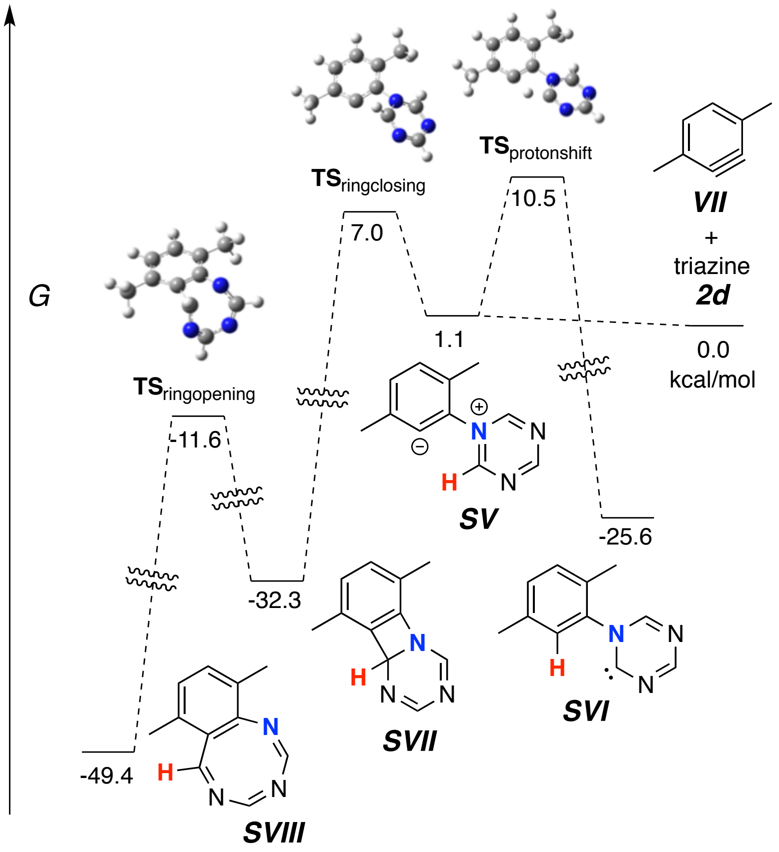

Geometry and free energy for 3,6-dimethylbenzyne (VII)

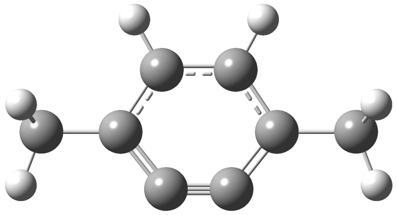

Sum of electronic and thermal Free Energies = -309.384039 a.u.

Center Number	Atomic Number	Atomic Type	Coordinates (Angstroms) X	Y	Z
1	6	0	0.620715	-1.182510	0.000003
2	6	0	-0.620715	-1.182510	-0.000004
3	6	0	-1.480728	-0.097659	-0.000012
4	6	0	-0.700246	1.080424	-0.000004
5	6	0	0.700246	1.080424	0.000005
6	6	0	1.480728	-0.097659	0.000001
7	1	0	-1.220246	2.034668	-0.000012
8	1	0	1.220246	2.034668	0.000006
9	6	0	-2.981786	-0.094617	0.000004
10	1	0	-3.367096	0.421123	0.882560
11	1	0	-3.367110	0.422213	-0.881906
12	1	0	-3.369692	-1.111832	-0.000612
13	6	0	2.981786	-0.094617	0.000001
14	1	0	3.367103	0.421632	-0.882254
15	1	0	3.367103	0.421704	0.882212
16	1	0	3.369692	-1.111832	0.000042

Geometry and free energy for isoquinoline (2a)

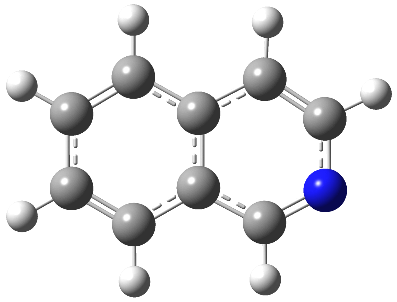

Sum of electronic and thermal Free Energies = -401.753046 a.u.

Center Number	Atomic Number	Atomic Type	Coordinates (Angstroms) X	Y	Z
1	6	0	-2.405445	-0.707170	-0.000001
2	6	0	-1.222054	-1.396244	-0.000004
3	6	0	0.007660	-0.692327	-0.000002
4	6	0	0.010126	0.722934	0.000002
5	6	0	-1.229794	1.412534	0.000005
6	6	0	-2.406029	0.710303	0.000004
7	1	0	-3.348235	-1.240630	-0.000002
8	1	0	-1.208261	-2.480851	-0.000008
9	6	0	1.269999	1.371582	0.000004
10	1	0	-1.229396	2.496844	0.000009
11	1	0	-3.351763	1.239422	0.000006
12	6	0	2.411434	0.616995	0.000001
13	1	0	1.324344	2.453980	0.000007
14	1	0	3.387390	1.090132	0.000002
15	7	0	2.420831	-0.744465	-0.000004
16	6	0	1.264091	-1.355851	-0.000005
17	1	0	1.280173	-2.444176	-0.000009

Geometry and free energy for zwitterion (VIII)

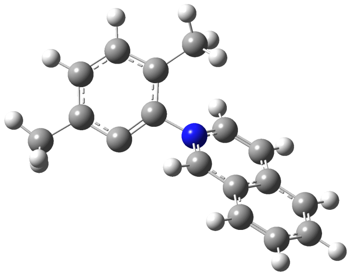

Sum of electronic and thermal Free Energies = -711.149014 a.u.

Center Number	Atomic Number	Atomic Type	Coordinates (Angstroms) X	Y	Z
1	6	0	-3.644227	1.081056	0.469811
2	6	0	-2.267361	1.263247	0.432154
3	6	0	-1.536573	0.147952	-0.022036
4	6	0	-2.002217	-1.111469	-0.364113
5	6	0	-3.411682	-1.217792	-0.282007
6	6	0	-4.210453	-0.139851	0.096922
7	1	0	-4.277022	1.890441	0.820008
8	1	0	-5.291982	-0.247418	0.131767
9	6	0	0.712874	-0.613754	0.393583
10	6	0	2.114054	-0.555946	0.266598
11	6	0	2.945249	-1.551479	0.838120
12	6	0	2.686953	0.526455	-0.448192
13	6	0	0.463635	1.359336	-0.844317
14	6	0	4.303749	-1.461655	0.695649
15	1	0	2.489771	-2.374952	1.375820
16	6	0	4.093457	0.592409	-0.579703
17	6	0	1.806164	1.486422	-1.011578
18	1	0	-0.259497	2.033098	-1.278896
19	6	0	4.878345	-0.381814	-0.017635
20	1	0	4.947780	-2.218633	1.125349
21	1	0	4.536655	1.415828	-1.127448
22	1	0	2.195493	2.311431	-1.594349
23	1	0	5.955745	-0.330062	-0.120321
24	7	0	-0.065199	0.317662	-0.127138
25	1	0	0.194723	-1.420739	0.896534
26	6	0	-4.066172	-2.538710	-0.622665
27	1	0	-3.645677	-3.337632	-0.006892
28	1	0	-5.148478	-2.514622	-0.471632
29	1	0	-3.868025	-2.803838	-1.664410
30	6	0	-1.667688	2.559386	0.931697
31	1	0	-0.700714	2.406472	1.416921
32	1	0	-1.523600	3.290488	0.130327
33	1	0	-2.337422	3.013732	1.663468

Geometry and free energy for $TS_{protonshift}$

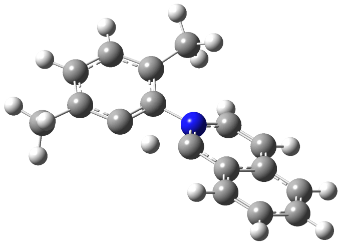

Sum of electronic and thermal Free Energies = -711.133741 a.u.

Center Number	Atomic Number	Atomic Type	Coordinates (Angstroms) X	Y	Z
1	6	0	-3.780294	-0.908987	-0.168947
2	6	0	-2.442000	-1.311097	-0.191252
3	6	0	-1.523552	-0.277954	0.033217
4	6	0	-1.820870	1.067794	0.160013
5	6	0	-3.175431	1.418596	0.154268
6	6	0	-4.144696	0.420275	0.020808
7	1	0	-4.549288	-1.656483	-0.336125
8	1	0	-5.199762	0.680462	0.030642
9	6	0	0.648721	0.615111	-0.173478
10	6	0	2.061679	0.558782	-0.120071
11	6	0	2.836634	1.715751	-0.386542
12	6	0	2.714696	-0.662844	0.190630
13	6	0	0.546817	-1.680279	0.433851
14	6	0	4.205424	1.650633	-0.349493
15	1	0	2.321172	2.639793	-0.620416
16	6	0	4.127422	-0.705469	0.221622
17	6	0	1.899344	-1.787604	0.490598
18	1	0	-0.109687	-2.488624	0.707655
19	6	0	4.851623	0.429850	-0.044955
20	1	0	4.799632	2.532572	-0.554920
21	1	0	4.626284	-1.638351	0.458881
22	1	0	2.347891	-2.726140	0.790627
23	1	0	5.934499	0.394971	-0.020813
24	7	0	-0.041492	-0.496124	0.065993
25	1	0	-0.360720	1.445583	-0.103360
26	6	0	-3.587253	2.865199	0.292618
27	1	0	-3.250323	3.268736	1.250728
28	1	0	-3.124144	3.469342	-0.491366
29	1	0	-4.670667	2.987885	0.228955
30	6	0	-2.131114	-2.749620	-0.542363
31	1	0	-1.262694	-2.833322	-1.199217
32	1	0	-1.950878	-3.370392	0.340567
33	1	0	-2.983779	-3.181989	-1.066948

Geometry and free energy for carbene (X)

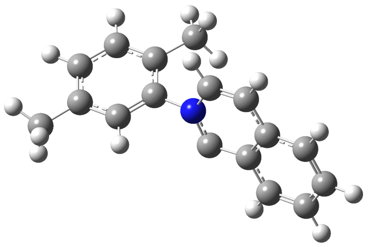

Sum of electronic and thermal Free Energies = -711.191244 a.u.

Center Number	Atomic Number	Atomic Type	Coordinates (Angstroms) X	Y	Z
1	6	0	-3.487426	1.203239	-0.658733
2	6	0	-2.100626	1.258646	-0.524549
3	6	0	-1.475183	0.129286	0.006559
4	6	0	-2.182902	-1.002508	0.378136
5	6	0	-3.569653	-1.047344	0.239477
6	6	0	-4.210076	0.076797	-0.279571
7	1	0	-4.008067	2.057824	-1.077740
8	1	0	-5.288522	0.069394	-0.397554
9	6	0	0.710327	-0.591279	-0.666833
10	6	0	2.128215	-0.479474	-0.395793
11	6	0	3.017386	-1.197303	-1.227913
12	6	0	2.667453	0.309454	0.650129
13	6	0	0.429792	0.925836	1.230796
14	6	0	4.377497	-1.134717	-1.030720
15	1	0	2.588207	-1.795581	-2.023361
16	6	0	4.063867	0.364384	0.842714
17	6	0	1.751564	1.025541	1.480335
18	1	0	-0.329327	1.427790	1.816686
19	6	0	4.900931	-0.346462	0.013464
20	1	0	5.050462	-1.689282	-1.673585
21	1	0	4.466700	0.971327	1.646520
22	1	0	2.106135	1.636231	2.301148
23	1	0	5.973567	-0.301730	0.163789
24	7	0	-0.036514	0.137810	0.183566
25	1	0	-1.636468	-1.855002	0.768046
26	6	0	-4.337097	-2.287043	0.618554
27	1	0	-4.227049	-3.056867	-0.149788
28	1	0	-3.969649	-2.704105	1.558059
29	1	0	-5.400469	-2.072120	0.729856
30	6	0	-1.310257	2.463440	-0.962369
31	1	0	-0.923949	3.026199	-0.107898
32	1	0	-0.452555	2.164842	-1.569580
33	1	0	-1.936298	3.133442	-1.551652

Geometry and free energy for $TS_{ringclosing}$

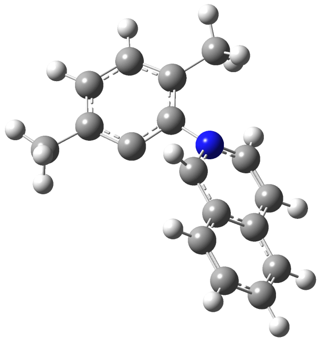

Sum of electronic and thermal Free Energies = -711.133298 a.u.

Center Number	Atomic Number	Atomic Type	Coordinates (Angstroms) X	Y	Z
1	6	0	3.838613	-0.513412	0.013935
2	6	0	2.640486	-1.186192	0.244523
3	6	0	1.495834	-0.400499	0.107209
4	6	0	1.454332	0.970053	-0.196405
5	6	0	2.689508	1.620330	-0.311480
6	6	0	3.861001	0.854367	-0.264716
7	1	0	4.773068	-1.061250	0.082042
8	1	0	4.819023	1.333900	-0.448671
9	6	0	-0.555136	0.270527	0.684110
10	6	0	-1.951728	0.344285	0.378269
11	6	0	-2.725731	1.437995	0.808682
12	6	0	-2.552010	-0.707430	-0.352643
13	6	0	-0.426292	-1.846796	-0.433746
14	6	0	-4.067140	1.499152	0.502619
15	1	0	-2.246257	2.234075	1.367624
16	6	0	-3.925063	-0.626221	-0.647968
17	6	0	-1.740863	-1.827966	-0.745013
18	1	0	0.251930	-2.621940	-0.762828
19	6	0	-4.665791	0.460225	-0.231698
20	1	0	-4.662438	2.344251	0.825348
21	1	0	-4.392715	-1.427840	-1.208607
22	1	0	-2.173872	-2.640116	-1.313284
23	1	0	-5.721754	0.513142	-0.469327
24	7	0	0.150792	-0.837009	0.297055
25	1	0	-0.160385	0.841596	1.507116
26	6	0	2.755364	3.110043	-0.546691
27	1	0	2.254191	3.646999	0.262497
28	1	0	3.786678	3.463271	-0.606138
29	1	0	2.242545	3.374550	-1.474255
30	6	0	2.604422	-2.639131	0.643858
31	1	0	1.799507	-2.833952	1.356645
32	1	0	2.448036	-3.291611	-0.220855
33	1	0	3.548066	-2.931981	1.106229

Geometry and free energy for intermediate (IX)

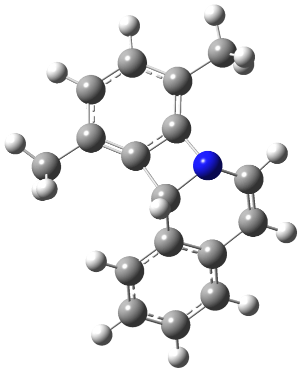

Sum of electronic and thermal Free Energies = -711.194186 a.u.

Center Number	Atomic Number	Atomic Type	Coordinates (Angstroms) X	Y	Z
1	6	0	3.448168	0.339680	-0.691527
2	6	0	2.782272	-0.826302	-0.279205
3	6	0	1.543601	-0.559512	0.273822
4	6	0	0.990725	0.706001	0.412170
5	6	0	1.651318	1.856902	0.032034
6	6	0	2.914810	1.621729	-0.541924
7	1	0	4.431560	0.236710	-1.140167
8	1	0	3.498829	2.471109	-0.880753
9	6	0	-0.239453	0.086857	1.075349
10	6	0	-1.584089	0.163844	0.396242
11	6	0	-2.233275	1.384464	0.263776
12	6	0	-2.198907	-1.014191	-0.056881
13	6	0	-0.198609	-2.346357	0.386974
14	6	0	-3.502412	1.455098	-0.305771
15	1	0	-1.747968	2.287708	0.620471
16	6	0	-3.474603	-0.931527	-0.621600
17	6	0	-1.479833	-2.291751	0.003529
18	1	0	0.390995	-3.255453	0.335144
19	6	0	-4.124445	0.291075	-0.745069
20	1	0	-4.002454	2.411651	-0.398970
21	1	0	-3.953153	-1.839116	-0.974262
22	1	0	-1.984198	-3.189961	-0.329821
23	1	0	-5.113514	0.335875	-1.185329
24	7	0	0.464024	-1.236548	0.917942
25	1	0	-0.333405	0.339950	2.136581
26	6	0	1.074300	3.238203	0.174478
27	1	0	0.314794	3.419826	-0.590865
28	1	0	0.599900	3.366435	1.150396
29	1	0	1.849303	3.998021	0.066829
30	6	0	3.349910	-2.210047	-0.420502
31	1	0	3.277687	-2.757868	0.522585
32	1	0	2.807202	-2.782376	-1.178031
33	1	0	4.399391	-2.171668	-0.714764

Geometry and free energy for $TS_{ringopening}$

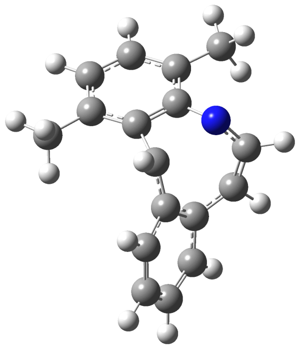

Sum of electronic and thermal Free Energies = -711.143061 a.u.

Center Number	Atomic Number	Atomic Type	Coordinates (Angstroms) X	Y	Z
1	6	0	3.182474	0.441385	-0.875488
2	6	0	2.636985	-0.677731	-0.245415
3	6	0	1.471026	-0.418325	0.477487
4	6	0	0.878813	0.811982	0.518039
5	6	0	1.413832	1.939183	-0.099181
6	6	0	2.599002	1.713047	-0.800909
7	1	0	4.104144	0.322973	-1.436913
8	1	0	3.076746	2.542118	-1.312514
9	6	0	-0.386314	0.561858	1.249486
10	6	0	-1.555501	0.163881	0.546496
11	6	0	-2.747779	0.900198	0.797451
12	6	0	-1.612096	-0.905961	-0.409002
13	6	0	0.078720	-2.271023	0.753300
14	6	0	-3.846709	0.779117	-0.010888
15	1	0	-2.735468	1.635299	1.594939
16	6	0	-2.755845	-0.973045	-1.256477
17	6	0	-0.816707	-2.081713	-0.282151
18	1	0	0.119209	-3.260272	1.214047
19	6	0	-3.829850	-0.142780	-1.082043
20	1	0	-4.717409	1.400917	0.157518
21	1	0	-2.781682	-1.743638	-2.019181
22	1	0	-1.195844	-2.961322	-0.794090
23	1	0	-4.688286	-0.221997	-1.738550
24	7	0	0.756270	-1.280789	1.354508
25	1	0	-0.571538	1.132958	2.160600
26	6	0	0.736091	3.279706	-0.034149
27	1	0	0.661658	3.633458	0.997872
28	1	0	1.285894	4.025090	-0.609494
29	1	0	-0.280407	3.218248	-0.433087
30	6	0	3.254580	-2.045274	-0.300857
31	1	0	3.325848	-2.474820	0.701826
32	1	0	2.645289	-2.726096	-0.902204
33	1	0	4.253617	-2.004420	-0.736531

Geometry and free energy for benzoazocine (XI)

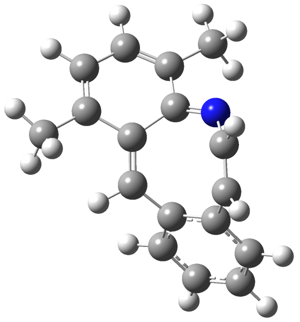

Sum of electronic and thermal Free Energies = -711.178595 a.u.

Center Number	Atomic Number	Atomic Type	Coordinates (Angstroms) X	Y	Z
1	6	0	3.229846	-0.445655	-0.977060
2	6	0	2.181430	-1.271148	-0.791771
3	6	0	1.132659	-0.821279	0.147726
4	6	0	1.008795	0.654897	0.315881
5	6	0	2.271389	1.419963	0.293654
6	6	0	3.319786	0.863010	-0.343355
7	1	0	4.065744	-0.775109	-1.586309
8	1	0	4.251032	1.412581	-0.429989
9	6	0	-0.173968	1.295102	0.249188
10	6	0	-1.509652	0.747562	-0.088206
11	6	0	-2.168909	1.341184	-1.172394
12	6	0	-2.162081	-0.254689	0.643083
13	6	0	-0.356999	-1.445831	1.876066
14	6	0	-3.437129	0.930659	-1.556042
15	1	0	-1.670359	2.135504	-1.718224
16	6	0	-3.452431	-0.639514	0.263573
17	6	0	-1.553197	-0.857022	1.846657
18	1	0	-0.014539	-1.910478	2.797905
19	6	0	-4.084077	-0.066973	-0.831556
20	1	0	-3.923576	1.395999	-2.404865
21	1	0	-3.959310	-1.403678	0.842950
22	1	0	-2.153475	-0.874845	2.750808
23	1	0	-5.080002	-0.388242	-1.112362
24	7	0	0.488388	-1.714828	0.796962
25	1	0	-0.145187	2.381859	0.283717
26	6	0	2.319392	2.808248	0.865460
27	1	0	3.348245	3.167513	0.894834
28	1	0	1.740169	3.510651	0.258928
29	1	0	1.908886	2.832747	1.877562
30	6	0	2.101303	-2.661983	-1.341931
31	1	0	2.925964	-2.850586	-2.029919
32	1	0	2.135802	-3.393298	-0.531580
33	1	0	1.154944	-2.816002	-1.866030

Geometry and free energy for quinoline (2b)

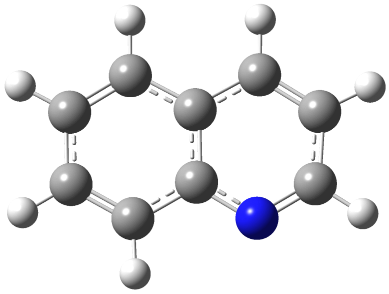

Sum of electronic and thermal Free Energies = -401.75446 a.u.

Center Number	Atomic Number	Atomic Type	Coordinates (Angstroms) X	Y	Z
1	6	0	2.417126	0.615772	0.000000
2	6	0	1.260889	1.350027	0.000000
3	6	0	0.000000	0.701207	0.000000
4	6	0	-0.044434	-0.718016	0.000000
5	6	0	1.168692	-1.452583	0.000000
6	6	0	2.372627	-0.800098	0.000000
7	1	0	3.377172	1.118162	0.000000
8	1	0	1.272387	2.433220	0.000000
9	6	0	-1.318609	-1.336093	0.000000
10	1	0	1.123878	-2.536436	0.000000
11	1	0	3.297482	-1.364278	0.000000
12	6	0	-2.438806	-0.553285	0.000000
13	6	0	-2.283570	0.855545	0.000000
14	1	0	-1.386235	-2.419036	0.000000
15	1	0	-3.432330	-0.983061	0.000000
16	1	0	-3.166880	1.488600	0.000000
17	7	0	-1.126995	1.469710	0.000000

Geometry and free energy for quinoline zwitterion (SI)

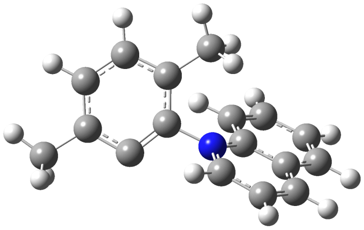

Sum of electronic and thermal Free Energies = -711.147606 a.u.

Center Number	Atomic Number	Atomic Type	Coordinates (Angstroms) X	Y	Z
1	6	0	2.758562	-1.157685	1.150972
2	6	0	1.452005	-0.690214	1.182621
3	6	0	1.128533	0.230463	0.168098
4	6	0	1.933716	0.766391	-0.818843
5	6	0	3.251984	0.239214	-0.778632
6	6	0	3.644870	-0.703619	0.168587
7	1	0	3.092633	-1.862869	1.905462
8	1	0	0.459581	2.554290	0.729709
9	1	0	4.662459	-1.087085	0.166064
10	6	0	-2.202983	-2.149007	-0.980669
11	6	0	-1.121739	-1.386354	-0.622365
12	6	0	-1.331510	-0.062132	-0.177618
13	6	0	-2.641464	0.475929	-0.136178
14	6	0	-3.734025	-0.349085	-0.504081
15	6	0	-3.519507	-1.636852	-0.912493
16	1	0	-2.044026	-3.161713	-1.330545
17	1	0	-0.112441	-1.770647	-0.687341
18	6	0	-2.811348	1.827014	0.243044
19	1	0	-4.734485	0.065747	-0.462443
20	1	0	-4.354208	-2.265011	-1.197484
21	6	0	-1.720628	2.596571	0.546351
22	6	0	-0.448728	2.010004	0.507472
23	1	0	-3.812369	2.243028	0.274480
24	1	0	-1.810355	3.638078	0.820800
25	7	0	-0.273357	0.738498	0.192988
26	6	0	4.259725	0.727143	-1.796311
27	1	0	4.382691	1.810358	-1.714855
28	1	0	5.237998	0.255843	-1.670234
29	1	0	3.902970	0.525892	-2.809510
30	6	0	0.489641	-1.132107	2.258547
31	1	0	-0.064693	-0.287121	2.677083
32	1	0	-0.247202	-1.849220	1.883438
33	1	0	1.032327	-1.613104	3.073449

Geometry and free energy for $TS_{quinolinecarbene}$

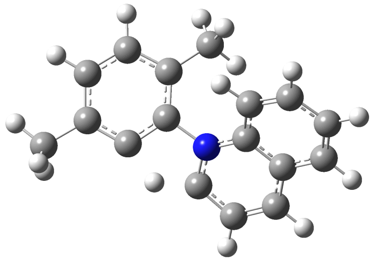

Sum of electronic and thermal Free Energies = -711.124955 a.u.

Center Number	Atomic Number	Atomic Type	Coordinates (Angstroms) X	Y	Z
1	6	0	-2.687515	-1.727417	0.466042
2	6	0	-1.364186	-1.295949	0.603532
3	6	0	-1.112928	-0.019752	0.089982
4	6	0	-2.070317	0.886822	-0.343432
5	6	0	-3.384565	0.418811	-0.432558
6	6	0	-3.670458	-0.903489	-0.071557
7	1	0	-2.955506	-2.711245	0.838369
8	1	0	-1.163051	2.030663	0.053515
9	1	0	-4.686053	-1.280799	-0.154868
10	6	0	2.702738	-1.826891	-1.011816
11	6	0	1.480482	-1.262579	-0.743211
12	6	0	1.420231	0.004792	-0.127088
13	6	0	2.614570	0.724094	0.116941
14	6	0	3.857526	0.094489	-0.135193
15	6	0	3.902726	-1.163085	-0.677154
16	1	0	2.745695	-2.793699	-1.498894
17	1	0	0.564617	-1.760690	-1.028349
18	6	0	2.520093	2.084951	0.507100
19	1	0	4.768245	0.641673	0.080504
20	1	0	4.854785	-1.637105	-0.880720
21	6	0	1.296996	2.696729	0.558788
22	6	0	0.116959	1.940281	0.401219
23	1	0	3.434965	2.635364	0.703596
24	1	0	1.213288	3.756737	0.762318
25	7	0	0.223917	0.630532	0.167178
26	6	0	-4.489351	1.333575	-0.905599
27	1	0	-4.459264	2.278497	-0.358465
28	1	0	-4.364793	1.571346	-1.965270
29	1	0	-5.475303	0.883642	-0.770451
30	6	0	-0.396658	-2.140665	1.401570
31	1	0	0.061736	-2.936164	0.808396
32	1	0	0.410090	-1.544789	1.831076
33	1	0	-0.934924	-2.615460	2.223604

Geometry and free energy for quinoline carbene (SII)

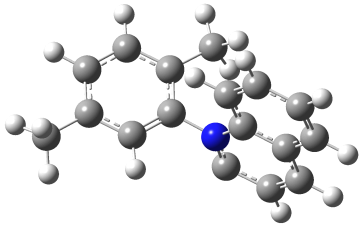

Sum of electronic and thermal Free Energies = -711.187546 a.u.

Center Number	Atomic Number	Atomic Type	Coordinates (Angstroms) X	Y	Z
1	6	0	2.821911	0.068886	1.546610
2	6	0	1.500865	-0.339621	1.367960
3	6	0	1.038748	-0.413605	0.054534
4	6	0	1.839341	-0.100247	-1.031933
5	6	0	3.158049	0.311192	-0.843301
6	6	0	3.635373	0.389694	0.464727
7	1	0	3.221922	0.127802	2.553344
8	1	0	1.424973	-0.192379	-2.030849
9	1	0	4.660795	0.698081	0.640221
10	6	0	-2.017274	2.440712	0.195723
11	6	0	-1.003633	1.510130	0.137226
12	6	0	-1.312946	0.159973	-0.125015
13	6	0	-2.651278	-0.223527	-0.330622
14	6	0	-3.666974	0.754316	-0.267258
15	6	0	-3.358638	2.067251	-0.006533
16	1	0	-1.774449	3.476836	0.399821
17	1	0	0.024223	1.811094	0.291438
18	6	0	-2.918952	-1.597810	-0.598380
19	1	0	-4.693949	0.445211	-0.428515
20	1	0	-4.140926	2.814401	0.044235
21	6	0	-1.896955	-2.493143	-0.646234
22	6	0	-0.512872	-2.147108	-0.429518
23	1	0	-3.950072	-1.900447	-0.760274
24	1	0	-2.111958	-3.535790	-0.852406
25	7	0	-0.328823	-0.833529	-0.187394
26	6	0	4.027628	0.678508	-2.017521
27	1	0	3.901744	1.733583	-2.276062
28	1	0	5.081930	0.514808	-1.790458
29	1	0	3.768752	0.087826	-2.897502
30	6	0	0.610460	-0.705733	2.523662
31	1	0	-0.256614	-0.040943	2.582126
32	1	0	0.232326	-1.724363	2.406973
33	1	0	1.155955	-0.640230	3.464906

Geometry and free energy for $TS_{quinolineringclosing}$

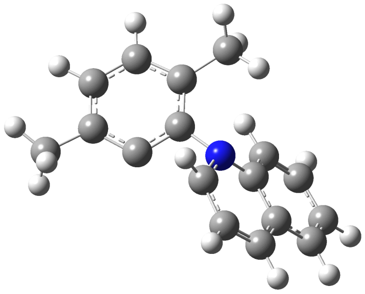

Sum of electronic and thermal Free Energies = -711.128667 a.u.

Center Number	Atomic Number	Atomic Type	Coordinates (Angstroms) X	Y	Z
1	6	0	-2.870211	1.655390	0.107010
2	6	0	-1.570363	1.446206	0.572516
3	6	0	-1.041956	0.186937	0.292062
4	6	0	-1.742371	-0.889963	-0.286947
5	6	0	-3.076831	-0.662291	-0.632955
6	6	0	-3.596753	0.633337	-0.498796
7	1	0	-3.335600	2.623735	0.263683
8	1	0	-0.784992	-1.833083	1.635772
9	1	0	-4.603347	0.843774	-0.851228
10	6	0	2.788925	1.808272	-0.973059
11	6	0	1.573732	1.411482	-0.450817
12	6	0	1.438769	0.126915	0.099524
13	6	0	2.525145	-0.774756	0.034191
14	6	0	3.753701	-0.334774	-0.489646
15	6	0	3.894924	0.946500	-0.976627
16	1	0	2.882211	2.802574	-1.393539
17	1	0	0.724834	2.078157	-0.480960
18	6	0	2.315741	-2.138290	0.426345
19	1	0	4.583291	-1.032766	-0.519224
20	1	0	4.842478	1.279163	-1.381044
21	6	0	1.101431	-2.559356	0.856960
22	6	0	0.034841	-1.624724	0.970664
23	1	0	3.151825	-2.826783	0.366248
24	1	0	0.924696	-3.584117	1.154032
25	7	0	0.250239	-0.302072	0.672679
26	6	0	-3.931820	-1.776714	-1.186400
27	1	0	-3.903679	-2.644421	-0.523347
28	1	0	-4.971528	-1.464751	-1.303316
29	1	0	-3.557862	-2.103223	-2.159792
30	6	0	-0.867781	2.491572	1.404495
31	1	0	-1.523058	2.831254	2.209298
32	1	0	0.046171	2.099980	1.853624
33	1	0	-0.601854	3.370564	0.809926

Geometry and free energy for quinoline azetidine (SIII)

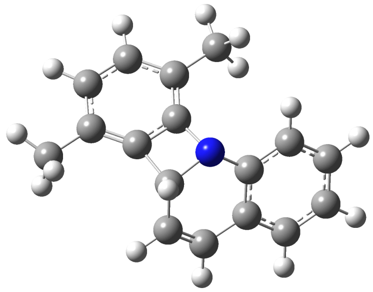

Sum of electronic and thermal Free Energies = -711.192771 a.u.

Center Number	Atomic Number	Atomic Type	Coordinates (Angstroms) X	Y	Z
1	6	0	-2.602715	1.859380	-0.582675
2	6	0	-1.269474	1.839422	-0.138110
3	6	0	-0.884677	0.592221	0.327711
4	6	0	-1.710489	-0.523445	0.341749
5	6	0	-3.025217	-0.504902	-0.073186
6	6	0	-3.448337	0.749212	-0.548657
7	1	0	-2.995499	2.795200	-0.968757
8	1	0	-0.742516	-1.749198	1.916347
9	1	0	-4.466091	0.857306	-0.908595
10	6	0	3.623202	1.201515	0.201972
11	6	0	2.326116	1.125729	0.702238
12	6	0	1.547653	0.000407	0.457437
13	6	0	2.076647	-1.077774	-0.271321
14	6	0	3.385369	-0.994914	-0.745810
15	6	0	4.157959	0.139423	-0.519791
16	1	0	4.219409	2.085969	0.393014
17	1	0	1.910356	1.933392	1.292860
18	6	0	1.236106	-2.254256	-0.525501
19	1	0	3.792851	-1.828170	-1.309059
20	1	0	5.170505	0.192136	-0.900556
21	6	0	0.022665	-2.393814	0.009338
22	6	0	-0.564686	-1.353678	0.910650
23	1	0	1.641629	-3.025565	-1.172186
24	1	0	-0.581126	-3.273466	-0.188196
25	7	0	0.221658	-0.066587	0.951844
26	6	0	-3.916920	-1.716050	-0.062583
27	1	0	-3.647834	-2.401266	-0.871052
28	1	0	-3.824431	-2.262270	0.878666
29	1	0	-4.961926	-1.433668	-0.195142
30	6	0	-0.385712	3.055104	-0.168467
31	1	0	-0.066257	3.331845	0.840231
32	1	0	0.513999	2.879087	-0.761671
33	1	0	-0.919614	3.903280	-0.598779

Geometry and free energy for $TS_{quinolineringopening}$

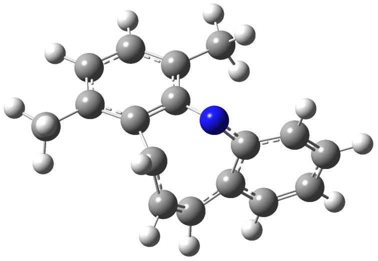

Sum of electronic and thermal Free Energies = -711.145699 a.u.

Center Number	Atomic Number	Atomic Type	Coordinates (Angstroms) X	Y	Z
1	6	0	-2.169604	2.035077	-0.655468
2	6	0	-0.906732	1.811005	-0.105633
3	6	0	-0.751287	0.552982	0.480847
4	6	0	-1.721353	-0.411760	0.444615
5	6	0	-2.988294	-0.198991	-0.090232
6	6	0	-3.185595	1.070537	-0.637882
7	1	0	-2.375705	3.002142	-1.103972
8	1	0	-1.476077	-2.160824	1.787997
9	1	0	-4.147945	1.311400	-1.077217
10	6	0	3.830085	0.539412	0.448470
11	6	0	2.675037	0.482863	1.185707
12	6	0	1.497076	-0.082056	0.629944
13	6	0	1.613862	-0.849320	-0.573401
14	6	0	2.821683	-0.757363	-1.319004
15	6	0	3.892512	-0.052947	-0.835719
16	1	0	4.697734	1.056193	0.841710
17	1	0	2.590562	0.964372	2.152789
18	6	0	0.758570	-1.970276	-0.772144
19	1	0	2.893812	-1.291605	-2.260552
20	1	0	4.806065	0.013923	-1.414172
21	6	0	-0.234416	-2.401384	0.081491
22	6	0	-1.024006	-1.619402	0.955788
23	1	0	1.121690	-2.693319	-1.497860
24	1	0	-0.378055	-3.478721	0.136774
25	7	0	0.313481	0.056537	1.272651
26	6	0	-4.038798	-1.274590	-0.113852
27	1	0	-4.234962	-1.656969	0.891280
28	1	0	-4.976147	-0.896296	-0.523001
29	1	0	-3.714729	-2.120093	-0.727536
30	6	0	0.190876	2.836689	-0.097098
31	1	0	0.586942	2.966671	0.913995
32	1	0	1.027660	2.524937	-0.729510
33	1	0	-0.172906	3.799582	-0.457849

Geometry and free energy for quinoline azocine (SIV)

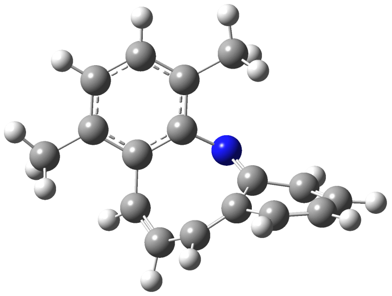

Sum of electronic and thermal Free Energies = -711.176878 a.u.

Center Number	Atomic Number	Atomic Type	Coordinates (Angstroms) X	Y	Z
1	6	0	-2.440367	1.952022	0.616013
2	6	0	-1.162588	1.855899	0.081542
3	6	0	-0.751142	0.613897	-0.438309
4	6	0	-1.602534	-0.489757	-0.405133
5	6	0	-2.905507	-0.366042	0.111862
6	6	0	-3.307539	0.862283	0.620509
7	1	0	-2.771017	2.902648	1.021749
8	1	0	-1.780095	-2.356753	-1.552570
9	1	0	-4.305809	0.969278	1.030874
10	6	0	3.947972	0.472905	-0.400296
11	6	0	2.849365	0.505108	-1.176970
12	6	0	1.553329	0.102602	-0.615808
13	6	0	1.641291	-0.831114	0.538700
14	6	0	2.803199	-0.673735	1.413565
15	6	0	3.906633	-0.053554	0.957997
16	1	0	4.885580	0.869556	-0.773383
17	1	0	2.846157	0.954727	-2.162525
18	6	0	0.899863	-1.958922	0.602150
19	1	0	2.780171	-1.145430	2.390091
20	1	0	4.793701	0.017086	1.575549
21	6	0	-0.065479	-2.453064	-0.381500
22	6	0	-1.136741	-1.813796	-0.865018
23	1	0	1.157076	-2.664008	1.391679
24	1	0	0.098560	-3.482648	-0.691744
25	7	0	0.493727	0.613345	-1.113575
26	6	0	-3.833374	-1.553604	0.138361
27	1	0	-4.069140	-1.897705	-0.873018
28	1	0	-3.381972	-2.395422	0.669680
29	1	0	-4.770340	-1.297387	0.633164
30	6	0	-0.228822	3.034357	0.043310
31	1	0	0.681528	2.835031	0.615640
32	1	0	0.084305	3.243628	-0.982852
33	1	0	-0.710149	3.921078	0.456844

Geometry and free energy for triazine (2d)

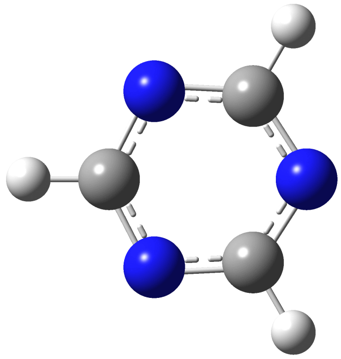

Sum of electronic and thermal Free Energies = -280.291265 a.u.

Center Number	Atomic Number	Atomic Type	Coordinates (Angstroms) X	Y	Z
1	6	0	0.645485	1.118087	0.000000
2	6	0	-1.290578	0.000000	0.000000
3	6	0	0.645485	-1.118087	0.000000
4	7	0	1.366120	0.000000	0.000000
5	1	0	-2.376376	0.000000	0.000000
6	1	0	1.187790	2.058726	0.000000
7	1	0	1.187790	-2.058726	0.000000
8	7	0	-0.683171	1.183299	0.000000
9	7	0	-0.683171	-1.183299	0.000000

Geometry and free energy for triazine zwitterion (SV)

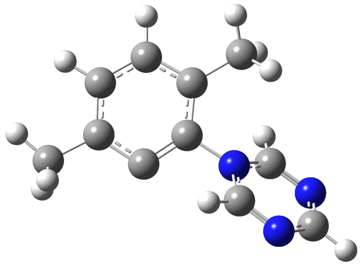

Sum of electronic and thermal Free Energies = -589.673566 a.u.

Center Number	Atomic Number	Atomic Type	Coordinates (Angstroms) X	Y	Z
1	6	0	-1.575454	-1.131604	-0.732944
2	6	0	-3.617690	-0.676074	0.077730
3	6	0	-1.897588	0.574972	0.781456
4	7	0	-1.061921	-0.116784	-0.005488
5	1	0	-4.677905	-0.901740	0.106394
6	1	0	-0.861153	-1.689753	-1.324643
7	1	0	-1.459963	1.347472	1.401392
8	6	0	2.523329	-0.868768	0.133902
9	6	0	1.124681	-1.086082	0.108735
10	6	0	0.404611	0.089278	-0.013750
11	6	0	0.867669	1.411362	-0.163600
12	6	0	2.249383	1.536933	-0.122567
13	6	0	3.063612	0.412637	0.042256
14	1	0	2.696384	2.518266	-0.244770
15	1	0	4.141285	0.551822	0.074170
16	6	0	0.001658	2.623865	-0.421576
17	1	0	-0.881632	2.384024	-1.019674
18	1	0	-0.339446	3.099074	0.503211
19	1	0	0.573139	3.372825	-0.971059
20	6	0	3.447570	-2.057879	0.264681
21	1	0	3.244945	-2.781544	-0.528482
22	1	0	4.500346	-1.769603	0.214541
23	1	0	3.273908	-2.570548	1.214117
24	7	0	-3.189464	0.326706	0.842922
25	7	0	-2.857272	-1.428797	-0.716167

Geometry and free energy for $TS_{triazinecarbene}$

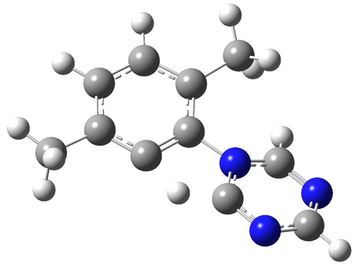

Sum of electronic and thermal Free Energies = -589.658644 a.u.

Center Number	Atomic Number	Atomic Type	Coordinates (Angstroms) X	Y	Z
1	6	0	1.048308	-1.009113	0.062419
2	6	0	2.447514	-0.927683	0.080660
3	6	0	3.064297	0.324408	0.056717
4	6	0	2.317125	1.497399	-0.037890
5	6	0	0.923765	1.478218	-0.075275
6	1	0	4.148115	0.395279	0.083022
7	1	0	-0.217343	-1.804490	-0.291802
8	1	0	2.826465	2.452587	-0.111618
9	6	0	-1.427517	-1.322221	-0.344995
10	6	0	-2.057715	0.803892	0.413843
11	6	0	-3.599969	-0.762677	-0.023155
12	1	0	-1.768433	1.772667	0.796726
13	1	0	-4.646604	-1.045673	-0.073203
14	6	0	3.278004	-2.187426	0.121921
15	1	0	4.347892	-1.970190	0.110136
16	1	0	3.051352	-2.764707	1.021346
17	1	0	3.043843	-2.823317	-0.735257
18	6	0	0.175247	2.773318	-0.286499
19	1	0	-0.658554	2.658642	-0.983687
20	1	0	-0.213910	3.189691	0.647490
21	1	0	0.852767	3.515795	-0.708612
22	7	0	-1.103132	-0.036901	0.008791
23	6	0	0.366648	0.195769	0.021797
24	7	0	-3.335136	0.476604	0.387409
25	7	0	-2.701708	-1.691073	-0.352171

Geometry and free energy for triazine carbene (SVI)

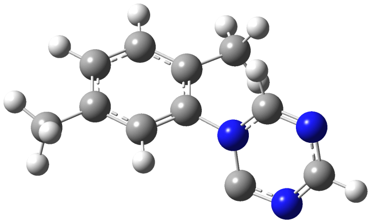

Sum of electronic and thermal Free Energies = -589.71606 a.u.

Center Number	Atomic Number	Atomic Type	Coordinates (Angstroms) X	Y	Z
1	6	0	1.603222	-0.965505	-1.086568
2	6	0	3.622363	-0.622972	0.034321
3	6	0	1.817542	0.229809	1.009857
4	7	0	1.079917	-0.225356	-0.023036
5	1	0	4.700051	-0.763642	0.052345
6	1	0	-0.815981	-2.009543	0.166592
7	1	0	1.292383	0.744273	1.810148
8	6	0	-2.593027	-0.782519	0.103742
9	6	0	-1.217119	-1.004311	0.094435
10	6	0	-0.337521	0.060286	-0.022658
11	6	0	-0.767937	1.381943	-0.155573
12	6	0	-2.146888	1.589229	-0.141180
13	6	0	-3.041036	0.532873	-0.008449
14	1	0	-2.525494	2.599718	-0.249828
15	1	0	-4.106866	0.734294	-0.004235
16	6	0	-3.551754	-1.938831	0.211259
17	1	0	-3.540341	-2.533717	-0.705372
18	1	0	-3.277926	-2.599006	1.036476
19	1	0	-4.571126	-1.588690	0.375000
20	6	0	0.193911	2.526674	-0.346291
21	1	0	0.678886	2.816898	0.589919
22	1	0	0.978553	2.269625	-1.061347
23	1	0	-0.336587	3.400531	-0.723722
24	7	0	3.108549	0.061041	1.078563
25	7	0	2.959236	-1.137229	-0.976006

Geometry and free energy for $TS_{triazineringclosing}$

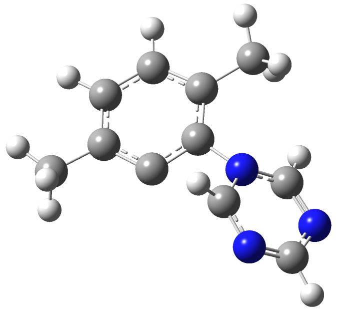

Sum of electronic and thermal Free Energies = -589.664162 a.u.

Center Number	Atomic Number	Atomic Type	Coordinates (Angstroms) X	Y	Z
1	6	0	1.395181	-1.005227	0.762582
2	6	0	3.417994	-0.865329	-0.224218
3	6	0	2.030251	0.875770	-0.489648
4	7	0	1.091445	0.226339	0.201326
5	1	0	4.367395	-1.329834	-0.470051
6	1	0	0.818359	-1.322834	1.612802
7	1	0	1.746131	1.825351	-0.929839
8	6	0	-2.223070	-1.084525	-0.139734
9	6	0	-0.826002	-0.974285	-0.062392
10	6	0	-0.334784	0.327620	0.072399
11	6	0	-1.062826	1.518638	0.110716
12	6	0	-2.435592	1.350504	-0.044439
13	6	0	-2.998929	0.078837	-0.176861
14	1	0	-3.078178	2.224834	-0.037394
15	1	0	-4.075236	-0.001719	-0.304753
16	6	0	-2.876540	-2.442515	-0.223575
17	1	0	-3.965006	-2.365309	-0.256626
18	1	0	-2.538064	-2.975321	-1.115101
19	1	0	-2.599528	-3.052192	0.639711
20	6	0	-0.445664	2.876060	0.330448
21	1	0	-1.185183	3.567203	0.736267
22	1	0	0.390754	2.828763	1.032282
23	1	0	-0.076191	3.308036	-0.604728
24	7	0	3.235345	0.389278	-0.686208
25	7	0	2.581015	-1.564227	0.514276

Geometry and free energy for triazine azetidine (SVII)

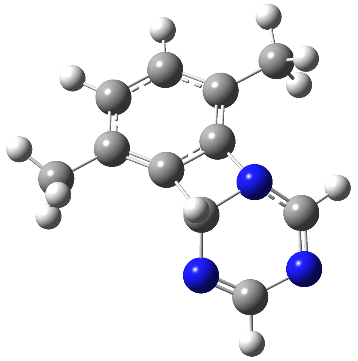

Sum of electronic and thermal Free Energies = -589.726798 a.u.

Center Number	Atomic Number	Atomic Type	Coordinates (Angstroms) X	Y	Z
1	6	0	1.013238	-0.875532	0.671307
2	6	0	2.974637	-0.828502	-0.520099
3	6	0	2.157539	1.218823	0.038808
4	7	0	1.072252	0.630426	0.605331
5	1	0	3.756591	-1.316633	-1.096415
6	1	0	1.117332	-1.217995	1.705526
7	1	0	2.134781	2.303817	-0.050933
8	6	0	-1.644852	-1.362461	-0.009447
9	6	0	-0.444198	-0.728861	0.236875
10	6	0	-0.311885	0.655065	0.227490
11	6	0	-1.329125	1.552611	-0.024923
12	6	0	-2.547537	0.908663	-0.301899
13	6	0	-2.701537	-0.478423	-0.296519
14	1	0	-3.413118	1.525038	-0.523443
15	1	0	-3.679635	-0.892373	-0.518263
16	6	0	-1.808907	-2.855988	-0.012393
17	1	0	-1.416221	-3.280801	-0.939549
18	1	0	-1.259862	-3.311811	0.813613
19	1	0	-2.860057	-3.133726	0.071535
20	6	0	-1.152314	3.043982	-0.015248
21	1	0	-0.583186	3.362747	0.861314
22	1	0	-0.611332	3.380420	-0.904105
23	1	0	-2.118401	3.548949	0.001388
24	7	0	3.188924	0.557120	-0.364807
25	7	0	1.982075	-1.539533	-0.152580

Geometry and free energy for $TS_{triazineringopening}$

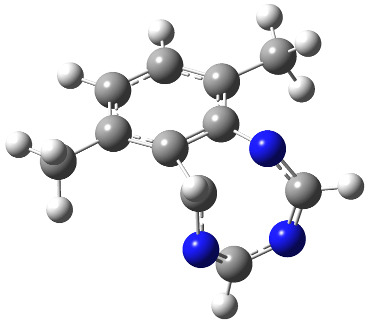

Sum of electronic and thermal Free Energies = -589.693727 a.u.

Center Number	Atomic Number	Atomic Type	Coordinates (Angstroms) X	Y	Z
1	6	0	0.751609	-1.319027	0.868952
2	6	0	2.062167	-1.215380	-1.037101
3	6	0	2.364281	0.665363	0.249009
4	7	0	1.263768	0.556844	1.030783
5	1	0	2.367096	-1.732740	-1.941520
6	1	0	0.820054	-1.784933	1.850854
7	1	0	3.182204	1.272799	0.642817
8	6	0	-1.788816	-0.976480	0.028081
9	6	0	-0.512994	-0.626091	0.442340
10	6	0	-0.029318	0.652202	0.428496
11	6	0	-0.760130	1.746586	-0.018387
12	6	0	-2.052988	1.417259	-0.438619
13	6	0	-2.553920	0.110455	-0.412290
14	1	0	-2.698805	2.212847	-0.796651
15	1	0	-3.568332	-0.067207	-0.753204
16	6	0	-2.293643	-2.392043	0.035044
17	1	0	-2.310842	-2.795796	1.050424
18	1	0	-3.303751	-2.447697	-0.371095
19	1	0	-1.645065	-3.036357	-0.563488
20	6	0	-0.195560	3.136865	-0.038736
21	1	0	0.302531	3.361014	0.907856
22	1	0	0.548297	3.245513	-0.833233
23	1	0	-0.979867	3.875893	-0.204644
24	7	0	2.612781	0.019653	-0.858196
25	7	0	1.458072	-1.905295	-0.119565

Geometry and free energy for triazine azocine (SVIII)

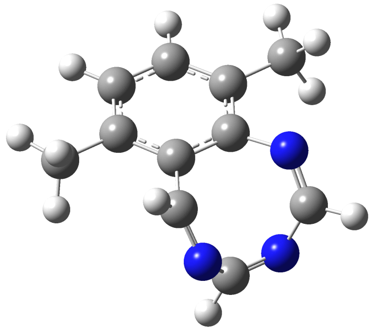

Sum of electronic and thermal Free Energies = -589.754073 a.u.

Center Number	Atomic Number	Atomic Type	Coordinates (Angstroms) X	Y	Z
1	6	0	-0.931685	-1.538401	-0.723808
2	6	0	-2.327737	-0.727156	0.934183
3	6	0	-2.171035	1.221845	-0.277150
4	7	0	-1.039918	1.365125	-0.833811
5	1	0	-2.532594	-1.260405	1.861585
6	1	0	-0.788883	-2.229469	-1.556257
7	1	0	-3.025438	1.750548	-0.697970
8	6	0	1.440069	-1.314111	-0.008660
9	6	0	0.224945	-0.689964	-0.333909
10	6	0	0.108921	0.708348	-0.336339
11	6	0	1.209177	1.507120	0.016733
12	6	0	2.392691	0.874190	0.375263
13	6	0	2.513897	-0.512849	0.358469
14	1	0	3.246199	1.480929	0.659381
15	1	0	3.455907	-0.976055	0.629952
16	6	0	1.562503	-2.816847	-0.033845
17	1	0	0.824114	-3.288964	0.619021
18	1	0	1.408097	-3.211561	-1.042284
19	1	0	2.553753	-3.125384	0.297374
20	6	0	1.083552	3.004857	0.000325
21	1	0	0.741720	3.348924	-0.978413
22	1	0	0.346275	3.344838	0.732017
23	1	0	2.040291	3.474489	0.229498
24	7	0	-2.446020	0.535819	0.910867
25	7	0	-2.071381	-1.559528	-0.160125

NMR Chemical Shift Calculation of Model Benzotriazocine S-9

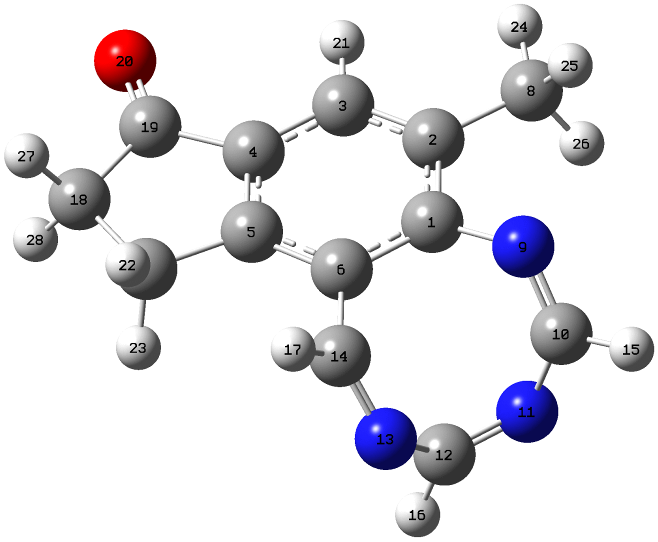

S-9

Gaussian atom numbers	Proton chemical shift	Proton experimental values	Gaussian atom numbers	Carbon chemical shift	Carbon experimental values
15	7.90	8.04	1	151.67	
16	7.69	7.97	2	132.35	
17	8.23	8.62	3	124.07	
21	7.54		4	130.44	
22	2.96		5	152.27	
23	2.96		6	122.50	
24	2.09		7	24.81	
25	2.20		8	18.17	
26	2.20		10	156.83	156.0
27	2.61		12	161.27	159.7
28	2.61		14	165.88	163.8
			18	36.37	
			19	203.03	

NMR Chemical Shift Calculation of Model Benzodiazocine S-10

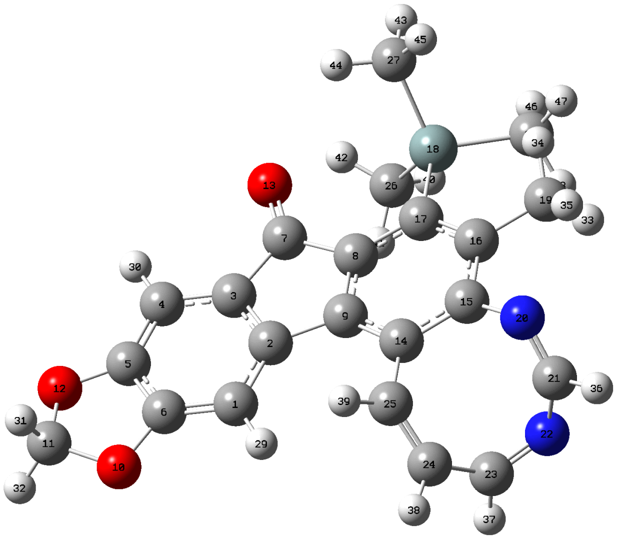

S-10

Gaussian atom numbers	Computed Proton chemical shift	Proton experimental values	Gaussian atom numbers	Computed Carbon chemical shift	Carbon experimental values	Key computed ^{1}H and ^{13}C shifts for compound
29	6.75		1	101.99		
30	6.84		2	139.64		
31	5.79		3	127.27		
32	5.83		4	102.14		
33	2.09		5	145.60		
34	2.14		6	150.88		
35	2.58		7	190.33		
36	7.87	8.10	8	134.27		7.88
37	7.71	7.86	9	138.57		5.32
38	6.45	6.50	11	99.71		6.97
39	6.88	6.99	14	122.26		8.34
40	0.33		15	148.78		
41	0.27		16	136.07		
42	0.55		17	142.39		
43	0.15		19	19.89		
44	0.52		21	157.85	157.3	162.12
45	0.60		23	165.47	164.0	109.09
46	0.25		24	130.36	130.7	145.61
47	0.75		25	136.65	134.6	161.13
48	0.58		26	2.26		
			27	2.76		
			28	3.62		

NMR Chemical Shift Calculation of Model, Isomeric Benzodiazocine S-11

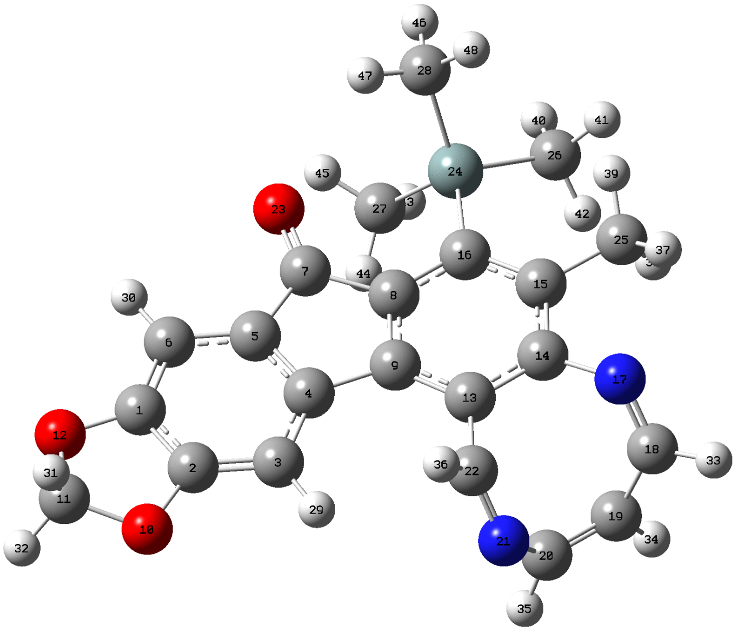

S-11

Gaussian atom numbers	Proton chemical shift	Proton experimental values	Gaussian atom numbers	Carbon chemical shift	Carbon experimental values
29	6.72		1	146.11	
30	6.84		2	151.17	
31	5.74		3	102.09	
32	5.89		4	138.50	
33	7.88		5	127.24	
34	5.32		6	102.29	
35	6.97		7	189.97	
36	8.34		8	134.28	
37	2.34		9	138.25	
38	1.97		11	99.86	
39	2.09		13	118.67	
40	0.21		14	150.79	
41	0.56		15	135.27	
42	0.64		16	143.69	
43	0.09		18	162.12	
44	0.33		19	109.09	
45	0.73		20	145.61	
46	0.21		22	161.13	
47	0.89		25	19.02	
48	0.39		26	3.80	
			27	1.86	
			28	3.16	

VII. References for the Supplementary Information

1 T. R. Hoye, P. R. Hanson and J. R. Vyvyan, *J. Org. Chem.,* 1994, **59**, 4096–4103.

2 T. R. Hoye and H. Zhao, *J. Org. Chem.,* 2002, **67**, 4014–4016.

3 T. Wang, R. R. Naredla, S. K. Thompson and T. R. Hoye, *Nature*, 2016, **532**, 484–488.

4 (a) J. Chen, V. Palani and T. R. Hoye, *J. Am. Chem. Soc.,* 2016, **138**, 4318–4321 and (b) S. P. Ross and T. R. Hoye, *Org. Lett.*, 2018, **20**, 100–103.

5 I. Cikotiene, R. Buksnaitiene and R. Sazinas, *Tetrahedron*, 2011, **67**, 706–717.

6 H. Valizadeh, A. Shomali and H. Gholipour, *J. Heterocyclic Chem.*, 2011, **48**, 1440–1444.

7 M. J. Frisch, G. W. Trucks, H. B. Schlegel, G. E. Scuseria, M. A. Robb, J. R. Cheeseman, G. Scalmani, V. Barone, B. Mennucci, G. A. Petersson, H. Nakatsuji, M. Caricato, X. Li, H. P. Hratchian, A. F. Izmaylov, J. Bloino, G. Zheng, J. L. Sonnenberg, M. Hada, M. Ehara, K. Toyota, R. Fukuda, J. Hasegawa, M. Ishida, T. Nakajima, Y. Honda, O. Kitao, H. Nakai, T. Vreven, J. A. Montgomery, Jr., J. E. Peralta, F. Ogliaro, M. Bearpark, J. J. Heyd, E. Brothers, K. N. Kudin, V. N. Staroverov, R. Kobayashi, J. Normand, K. Raghavachari, A. Rendell, J. C. Burant, S. S. Iyengar, J. Tomasi, M. Cossi, N. Rega, J. M. Millam, M. Klene, J. E. Knox, J. B. Cross, V. Bakken, C. Adamo, J. Jaramillo, R. Gomperts, R. E. Stratmann, O. Yazyev, A. J. Austin, R. Cammi, C. Pomelli, J. W. Ochterski, R. L. Martin, K. Morokuma, V. G. Zakrzewski, G. A. Voth, P. Salvador, J. J. Dannenberg, S. Dapprich, A. D. Daniels, O□. Farkas, J. B. Foresman, J. V. Ortiz, J. Cioslowski, D. J. Fox. *Gaussian 09*, revision D.01; Gaussian, Inc.: Wallingford, CT, 2009.

8 Y. Zhao and D. G. Truhlar, *Theor. Chem. Acc.* 2008, **120**, 215–241.

9 A. V. Marenich, C. J. Cramer and D. G. Truhlar, *J. Phys. Chem. B*, 2009, **113**, 6378–6396.

10 P. H. Willoughby, M. J. Jansma and T. R. Hoye, *Nature Protocols*, 2014, **9**, 643–660.

V. Copies of ^{1}H and ^{13}C NMR spectra

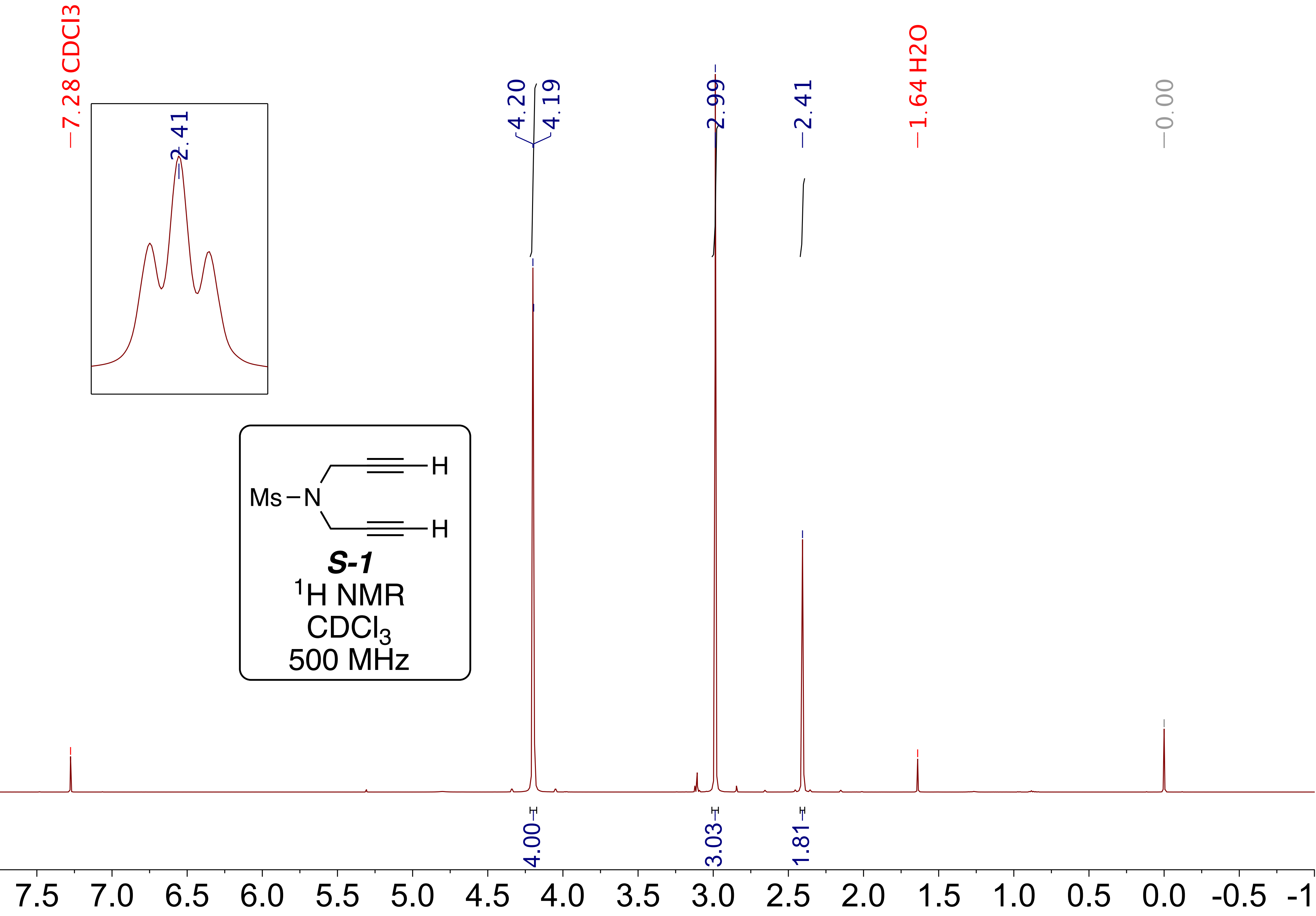
7.28 CDCl3
2.41
4.20
4.19
2.99
2.41
1.64 H2O
0.00
Ms
N
H
H
S-1
1H NMR
CDCl3
500 MHz
4.00
3.03
1.81
7.5 7.0 6.5 6.0 5.5 5.0 4.5 4.0 3.5 3.0 2.5 2.0 1.5 1.0 0.5 0.0 -0.5 -1

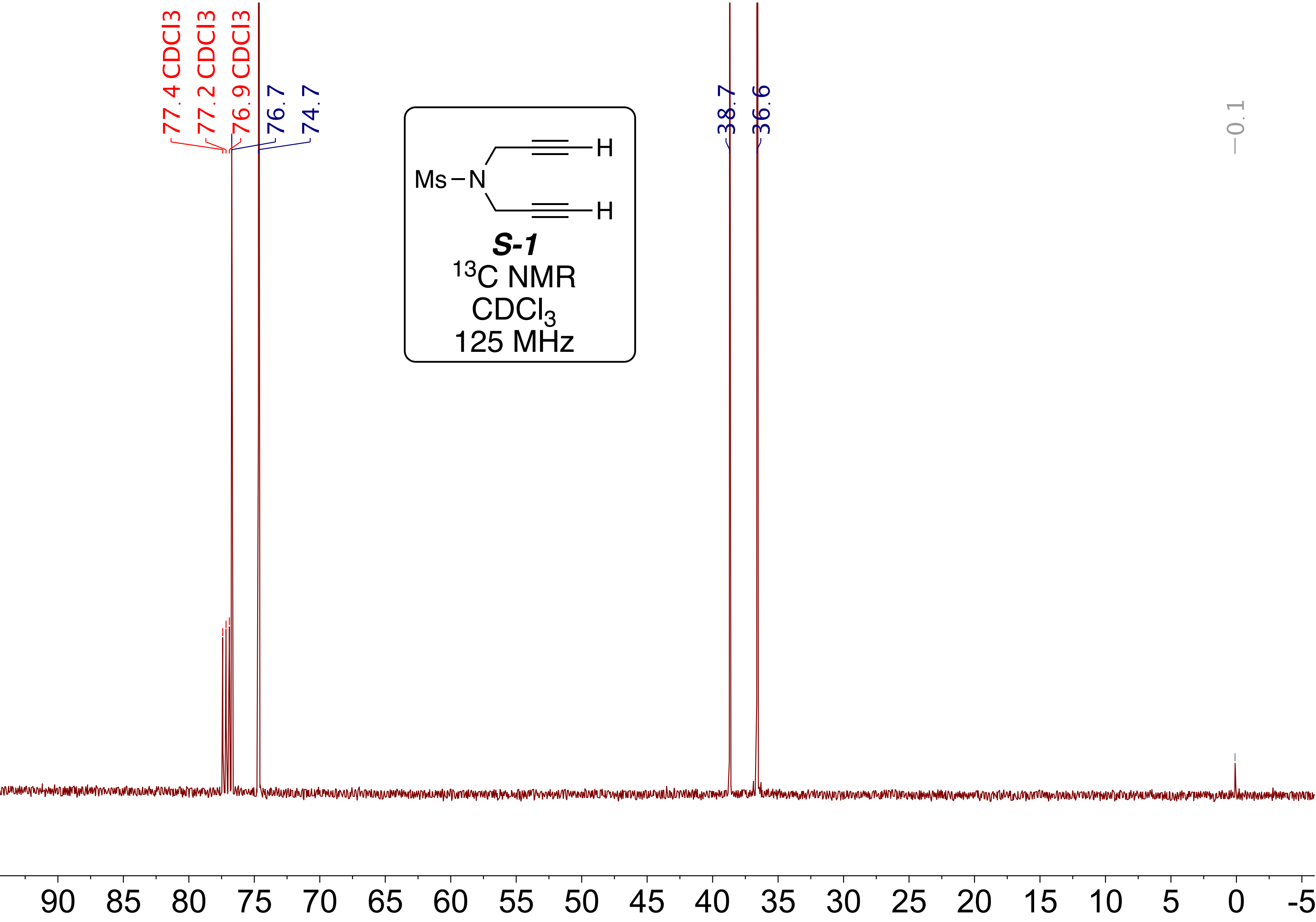
77.4 CDCl3
77.2 CDCl3
76.9 CDCl3
76.7
74.7
38.7
36.6
-0.1
Ms
N
H
H
S-1
13C NMR
CDCl3
125 MHz
90
85
80
75
70
65
60
55
50
45
40
35
30
25
20
15
10
5
0
-5

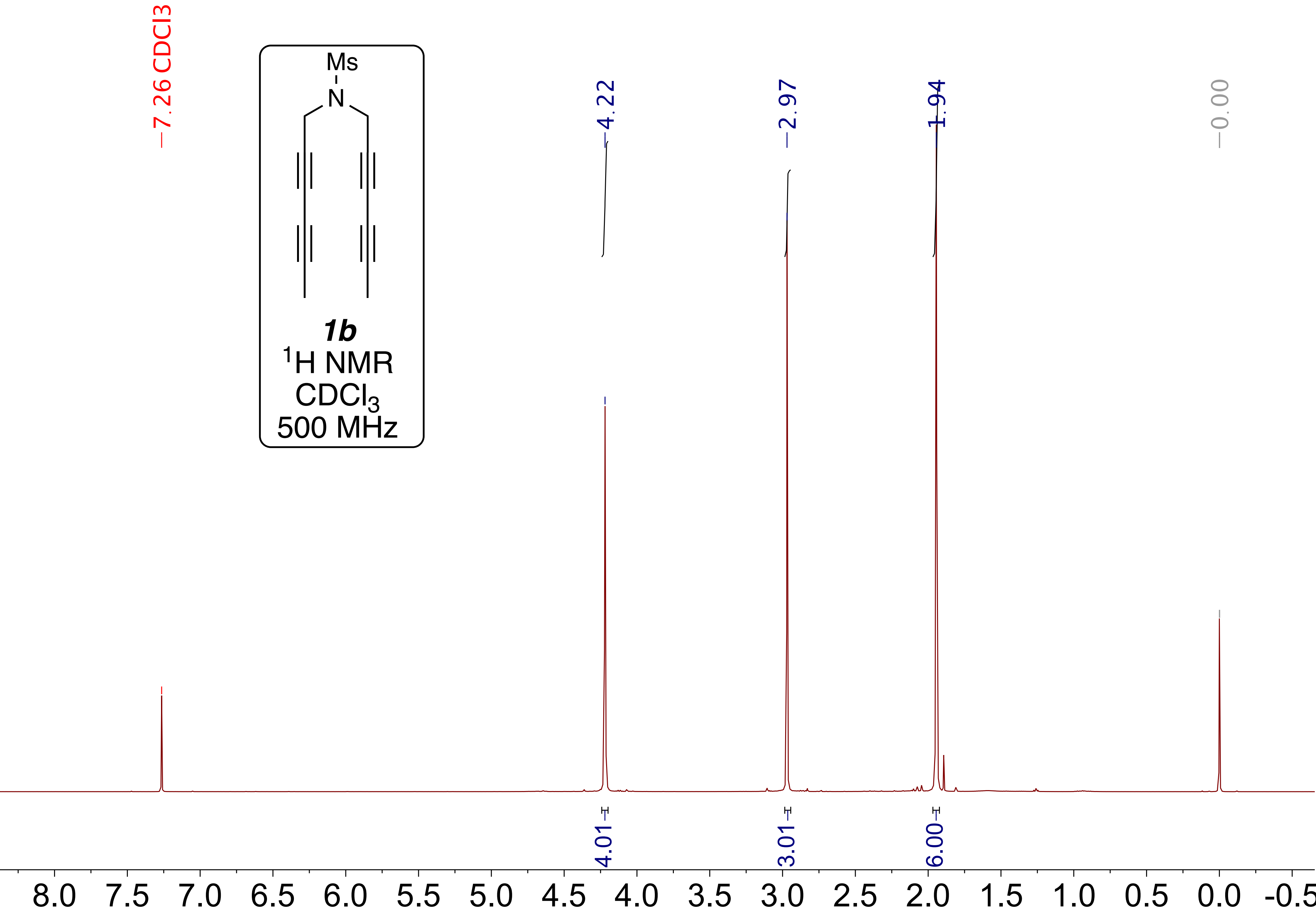
7.26 CDCl3
Ms
N
1b
1H NMR
CDCl3
500 MHz
4.22
2.97
1.94
0.00
4.01
3.01
6.00
8.0
7.5
7.0
6.5
6.0
5.5
5.0
4.5
4.0
3.5
3.0
2.5
2.0
1.5
1.0
0.5
0.0
-0.5

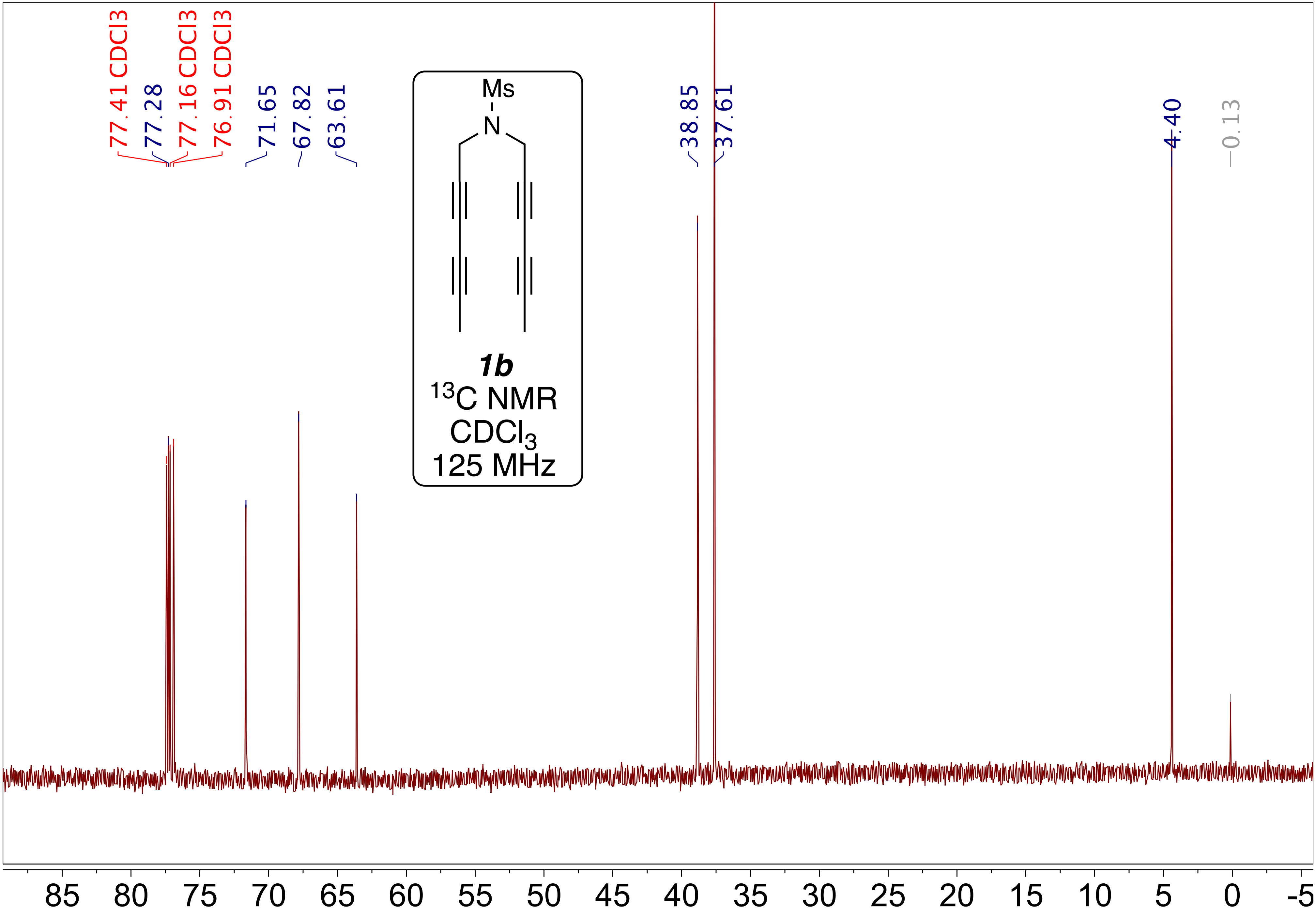
77.41 CDCl3
77.28
77.16 CDCl3
76.91 CDCl3
71.65
67.82
63.61
38.85
37.61
4.40
0.13
Ms
N
1b
13C NMR
CDCl3
125 MHz
85 80 75 70 65 60 55 50 45 40 35 30 25 20 15 10 5 0 -5

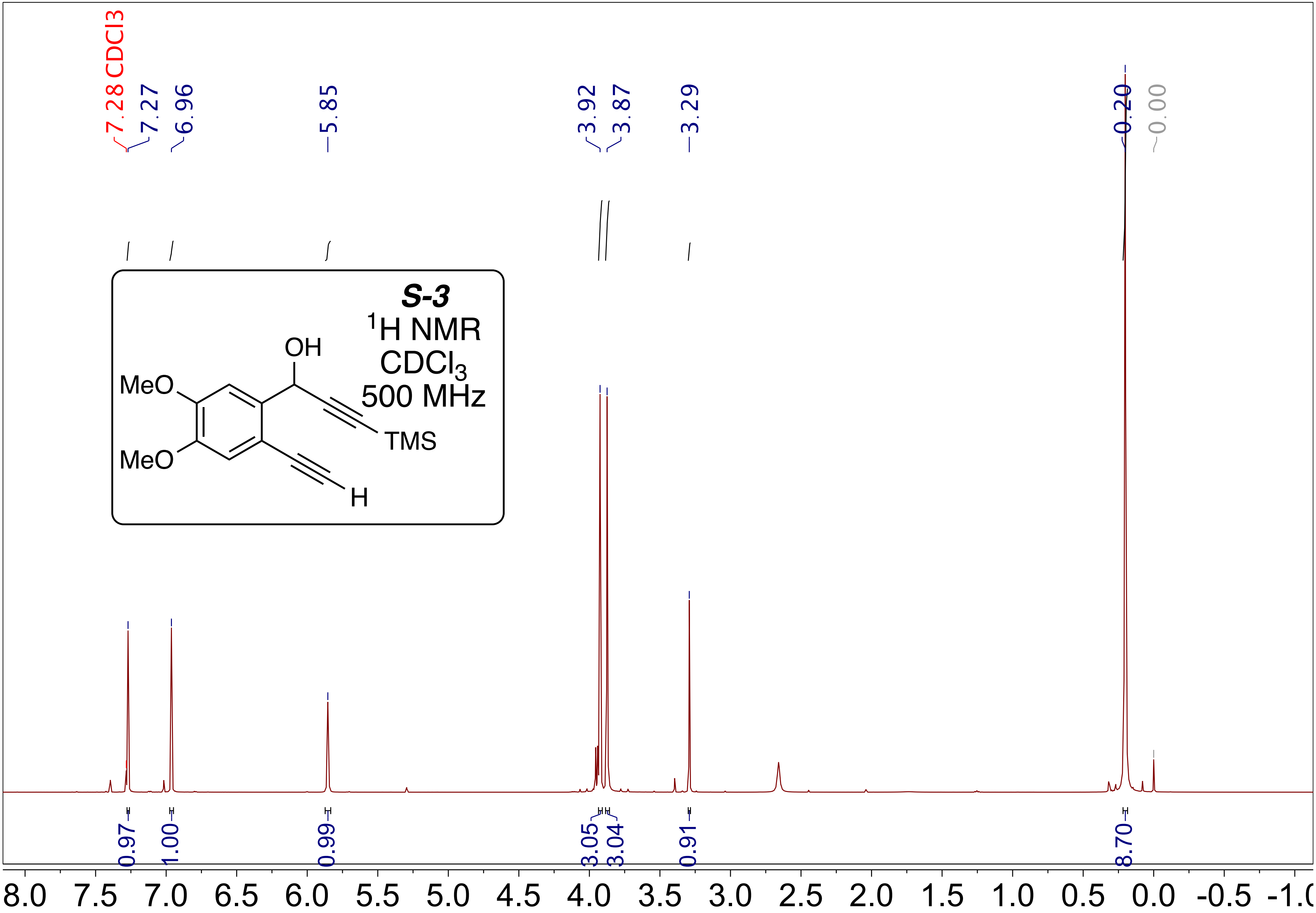
7.28 CDCl3
7.27
6.96
5.85
3.92
3.87
3.29
0.20
0.00
S-3
^{1}H NMR
$CDCl_3$
500 MHz
OH
MeO
MeO
TMS
H
0.97
1.00
0.99
3.05
3.04
0.91
8.70
8.0 7.5 7.0 6.5 6.0 5.5 5.0 4.5 4.0 3.5 3.0 2.5 2.0 1.5 1.0 0.5 0.0 -0.5 -1.0

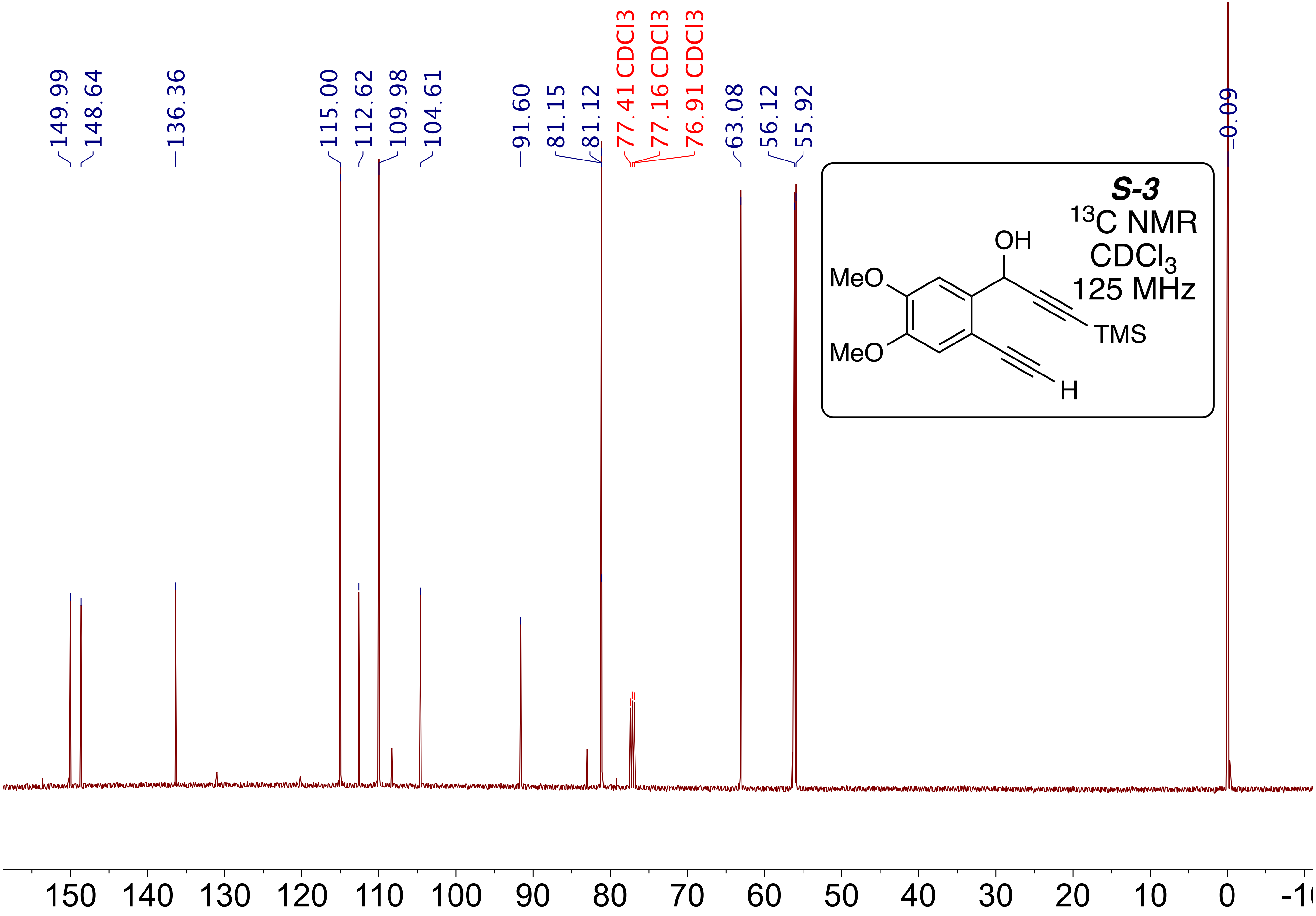
149.99
148.64
136.36
115.00
112.62
109.98
104.61
91.60
81.15
81.12
77.41 CDCl3
77.16 CDCl3
76.91 CDCl3
63.08
56.12
55.92
-0.09
S-3
13C NMR
CDCl3
125 MHz
OH
MeO
MeO
TMS
H
150
140
130
120
110
100
90
80
70
60
50
40
30
20
10
0

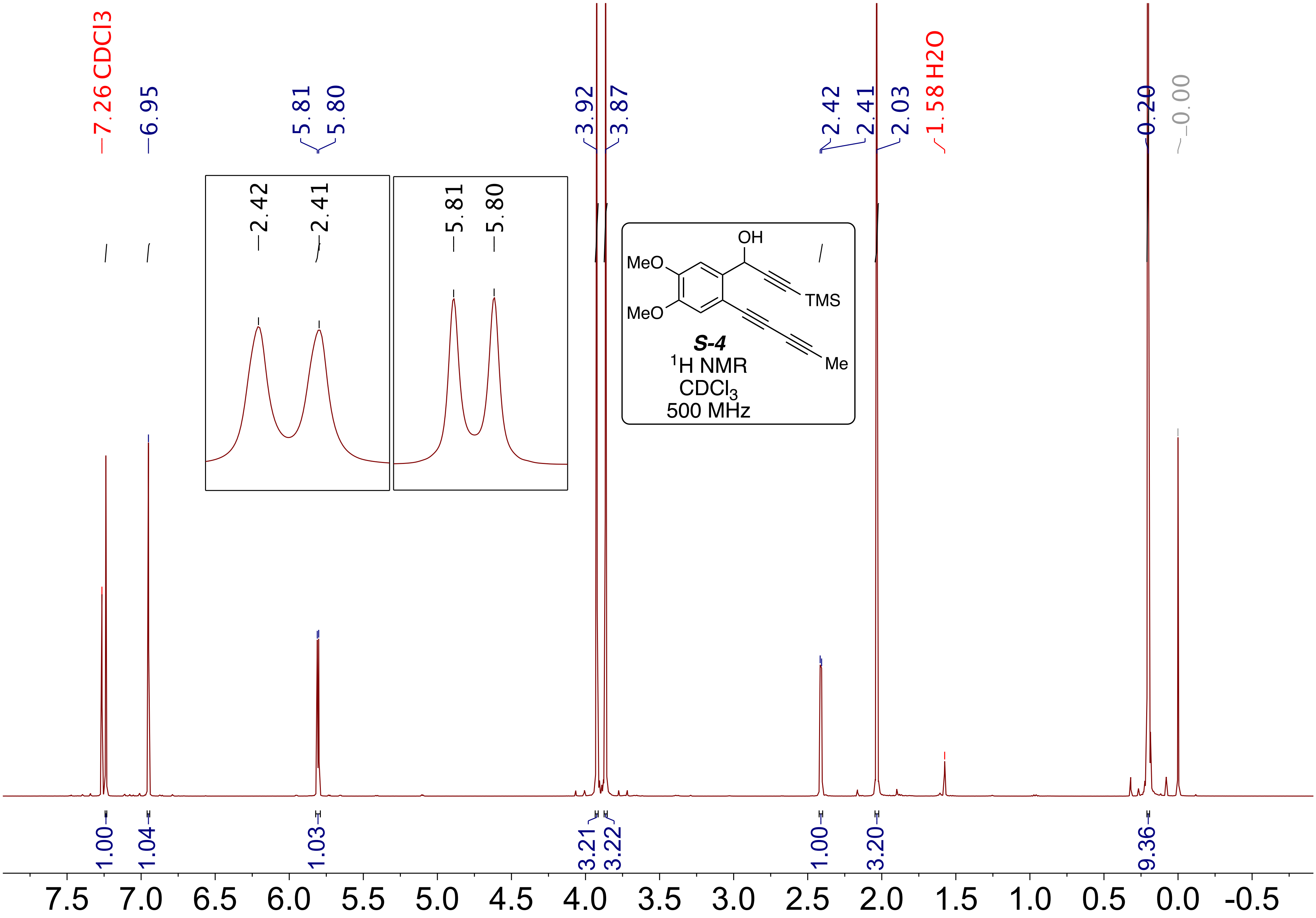
7.26 CDCl3
6.95
5.81
5.80
3.92
3.87
2.42
2.41
2.03
1.58 H2O
0.20
0.00
OH
MeO
MeO
TMS
Me
S-4
1H NMR
CDCl3
500 MHz
1.00
1.04
1.03
3.21
3.22
1.00
3.20
9.36
7.5 7.0 6.5 6.0 5.5 5.0 4.5 4.0 3.5 3.0 2.5 2.0 1.5 1.0 0.5 0.0 -0.5

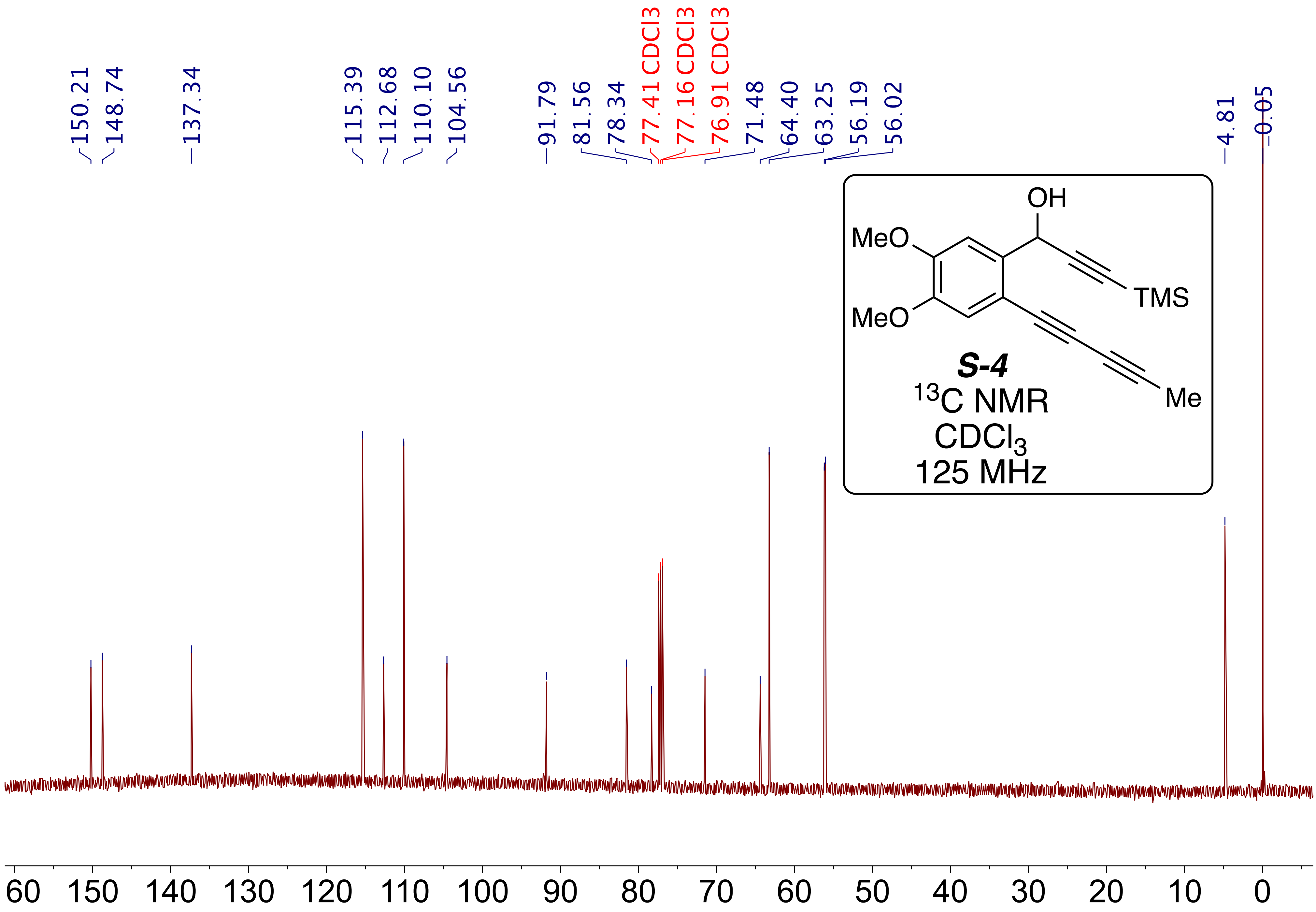
150.21
148.74
137.34
115.39
112.68
110.10
104.56
91.79
81.56
78.34
77.41 CDCl3
77.16 CDCl3
76.91 CDCl3
71.48
64.40
63.25
56.19
56.02
4.81
-0.05
OH
MeO
MeO
TMS
Me
S-4
13C NMR
CDCl3
125 MHz
160 150 140 130 120 110 100 90 80 70 60 50 40 30 20 10 0

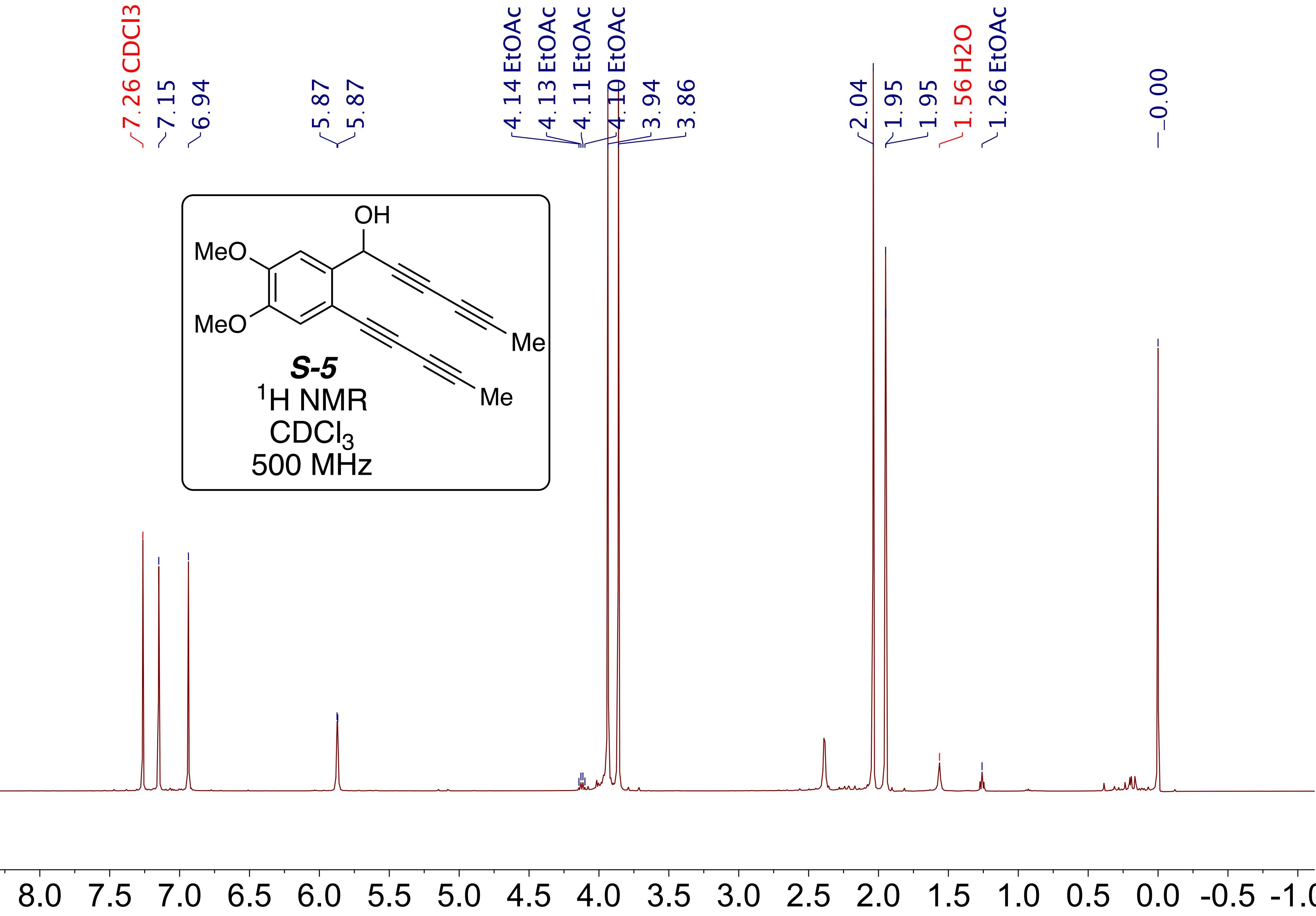
7.26 CDCl3
7.15
6.94
5.87
5.87
4.14 EtOAc
4.13 EtOAc
4.11 EtOAc
4.10 EtOAc
3.94
3.86
2.04
1.95
1.95
1.56 H2O
1.26 EtOAc
0.00
OH
MeO
MeO
Me
Me
S-5
1H NMR
CDCl3
500 MHz
8.0 7.5 7.0 6.5 6.0 5.5 5.0 4.5 4.0 3.5 3.0 2.5 2.0 1.5 1.0 0.5 0.0 -0.5 -1.0

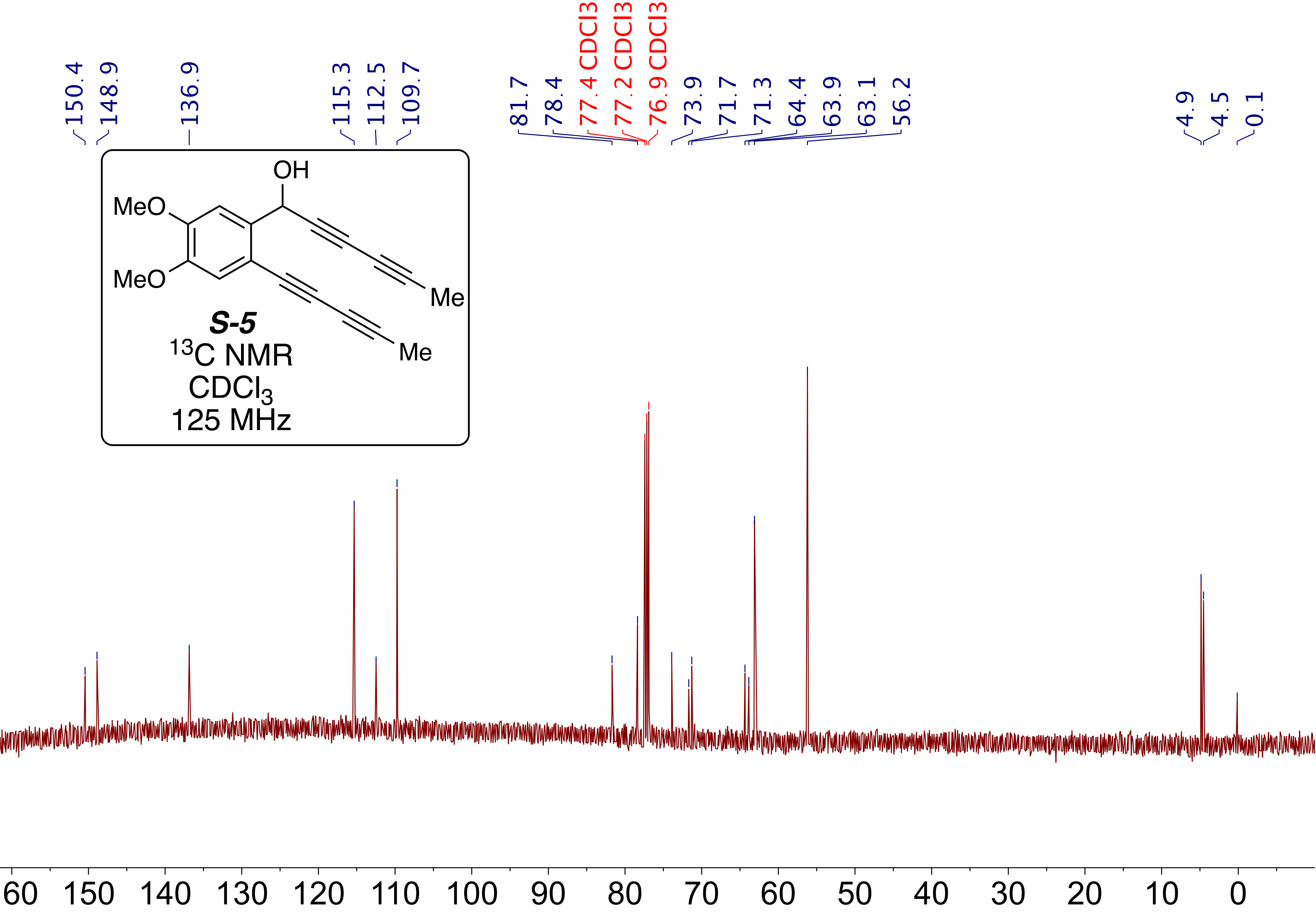
150.4
148.9
136.9
115.3
112.5
109.7
81.7
78.4
77.4 CDCl3
77.2 CDCl3
76.9 CDCl3
73.9
71.7
71.3
64.4
63.9
63.1
56.2
4.9
4.5
0.1
OH
MeO
MeO
Me
Me
S-5
13C NMR
CDCl3
125 MHz
160 150 140 130 120 110 100 90 80 70 60 50 40 30 20 10 0

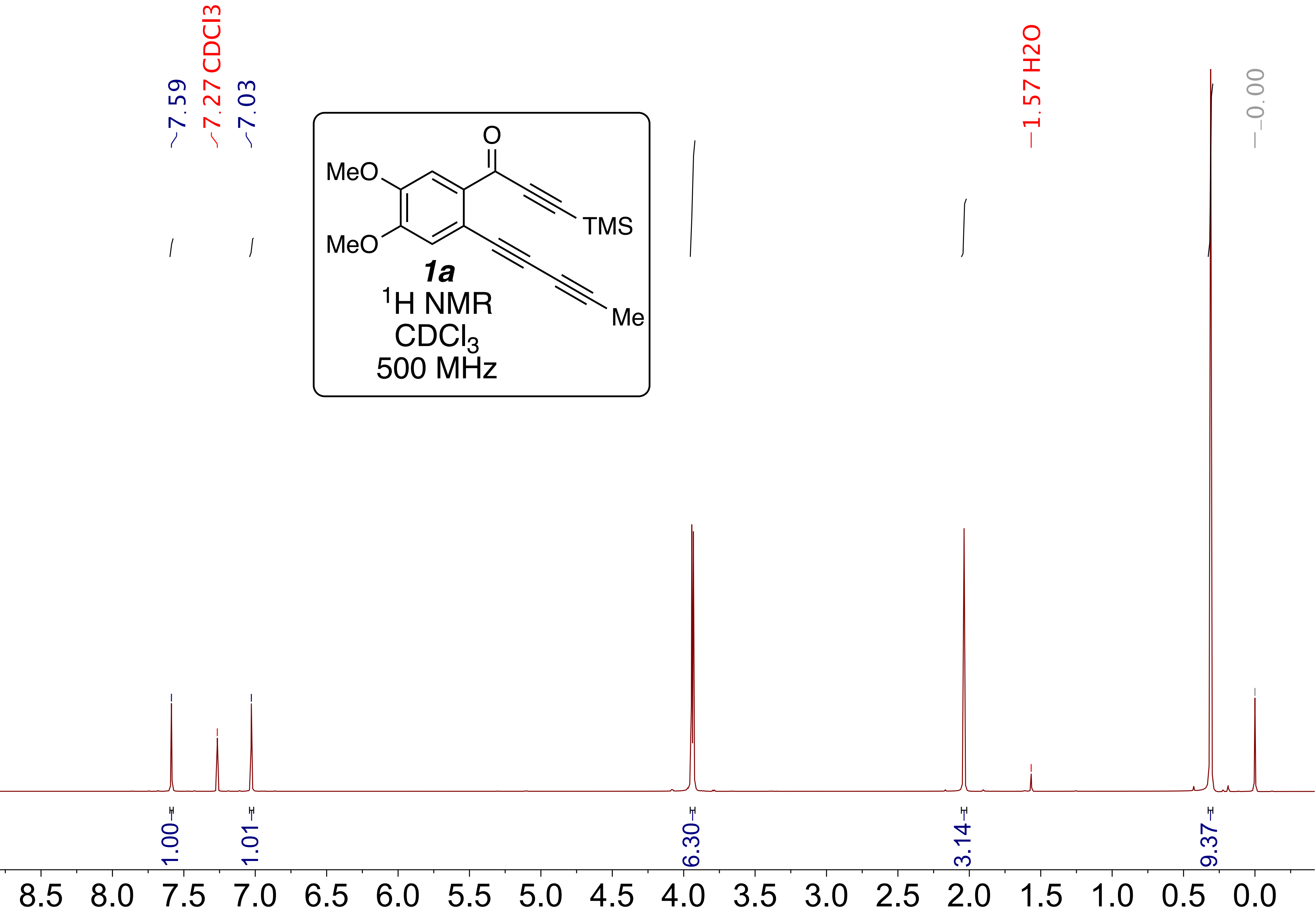

7.59
7.27 CDCl3
7.03
1.57 H2O
0.00
MeO
MeO
O
TMS
Me
1a
1H NMR
CDCl3
500 MHz
1.00
1.01
6.30
3.14
9.37
8.5 8.0 7.5 7.0 6.5 6.0 5.5 5.0 4.5 4.0 3.5 3.0 2.5 2.0 1.5 1.0 0.5 0.0

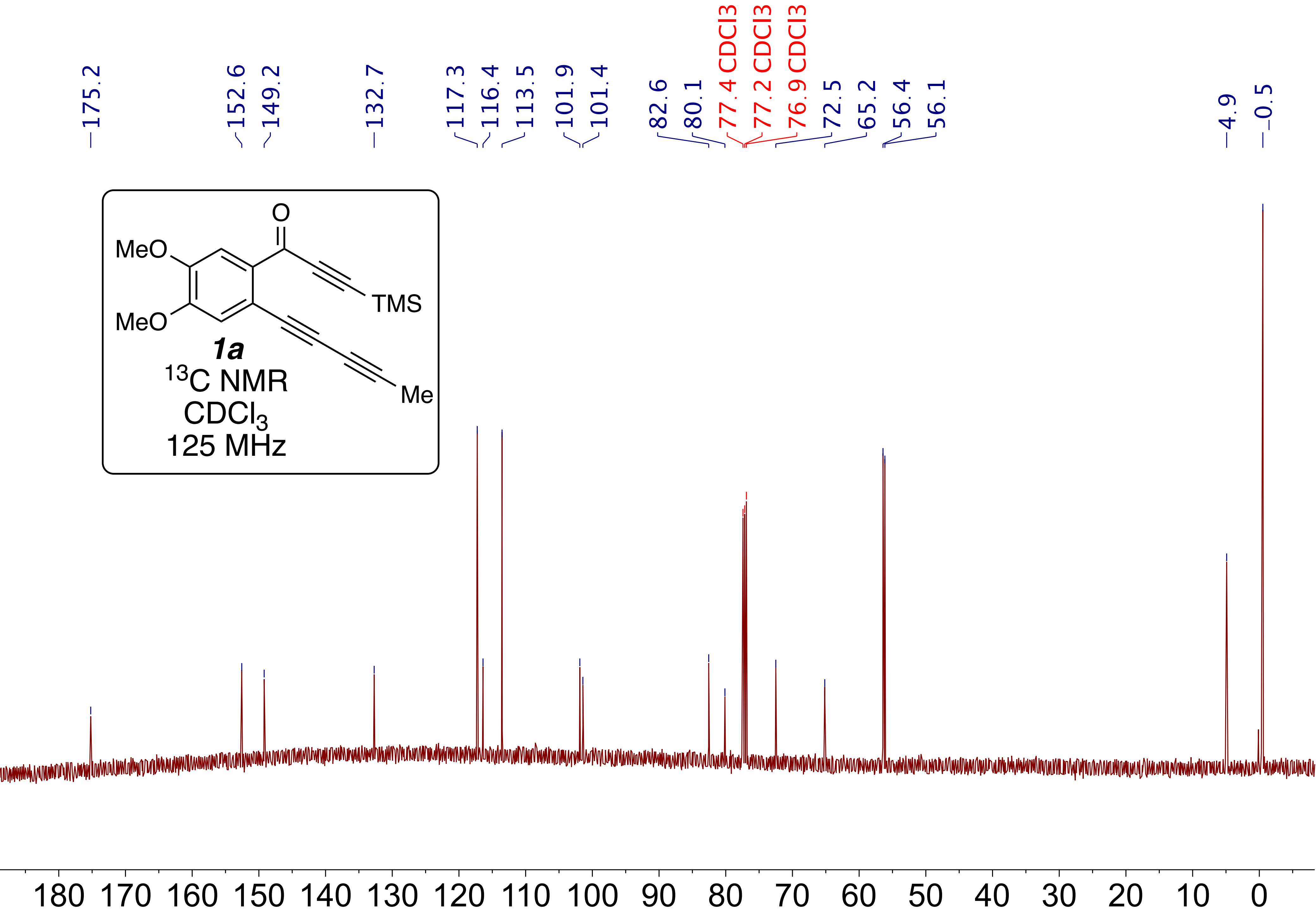
175.2
152.6
149.2
132.7
117.3
116.4
113.5
101.9
101.4
82.6
80.1
77.4 CDCl3
77.2 CDCl3
76.9 CDCl3
72.5
65.2
56.4
56.1
4.9
-0.5
MeO
MeO
O
TMS
Me
1a
13C NMR
CDCl3
125 MHz
180 170 160 150 140 130 120 110 100 90 80 70 60 50 40 30 20 10 0

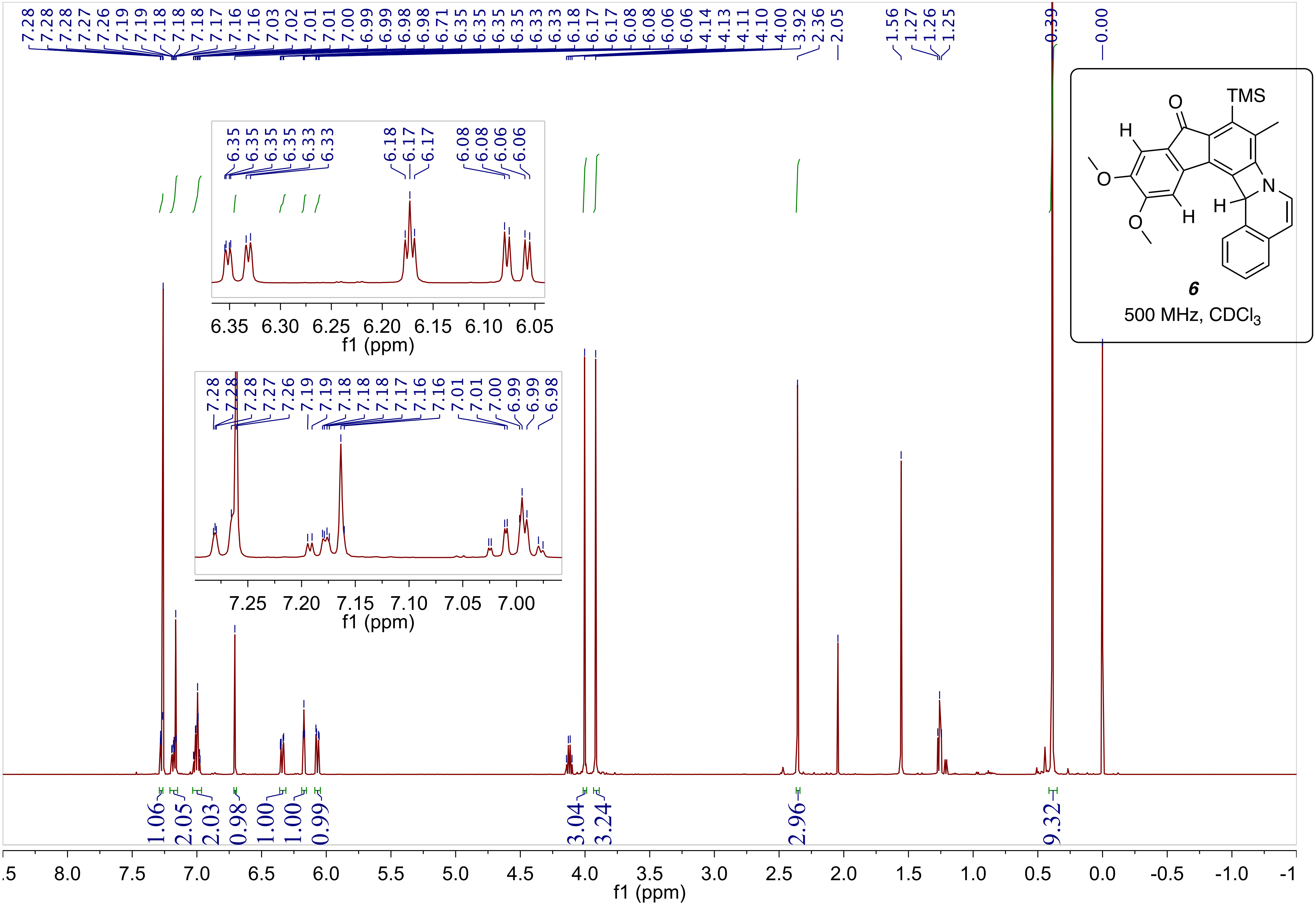
TMS
O
H
O
N
H
H
O
6
500 MHz, $CDCl_3$
f1 (ppm)

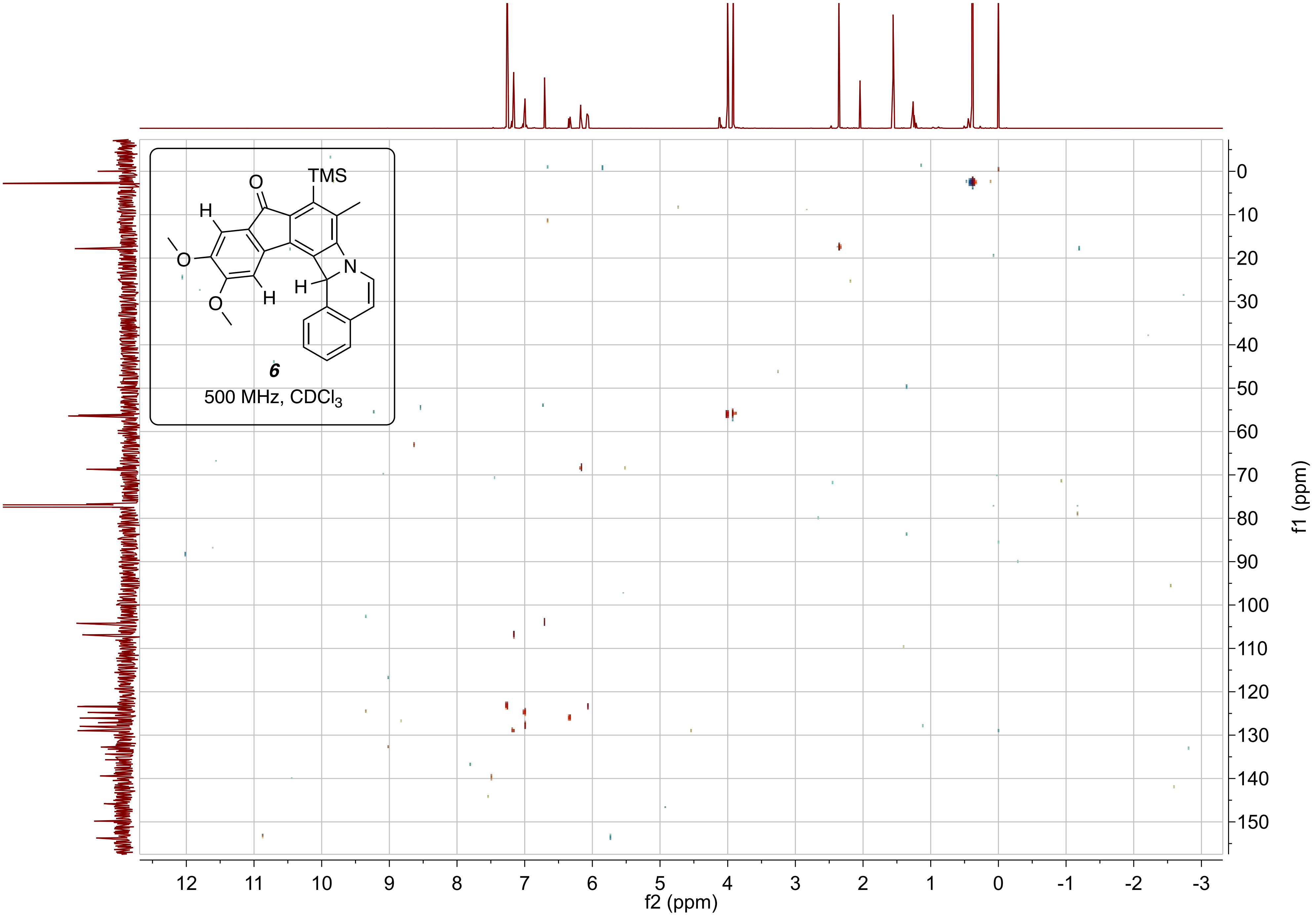
TMS
O
H
O
O
H
N
H
6
500 MHz, CDCl3
f2 (ppm)
f1 (ppm)

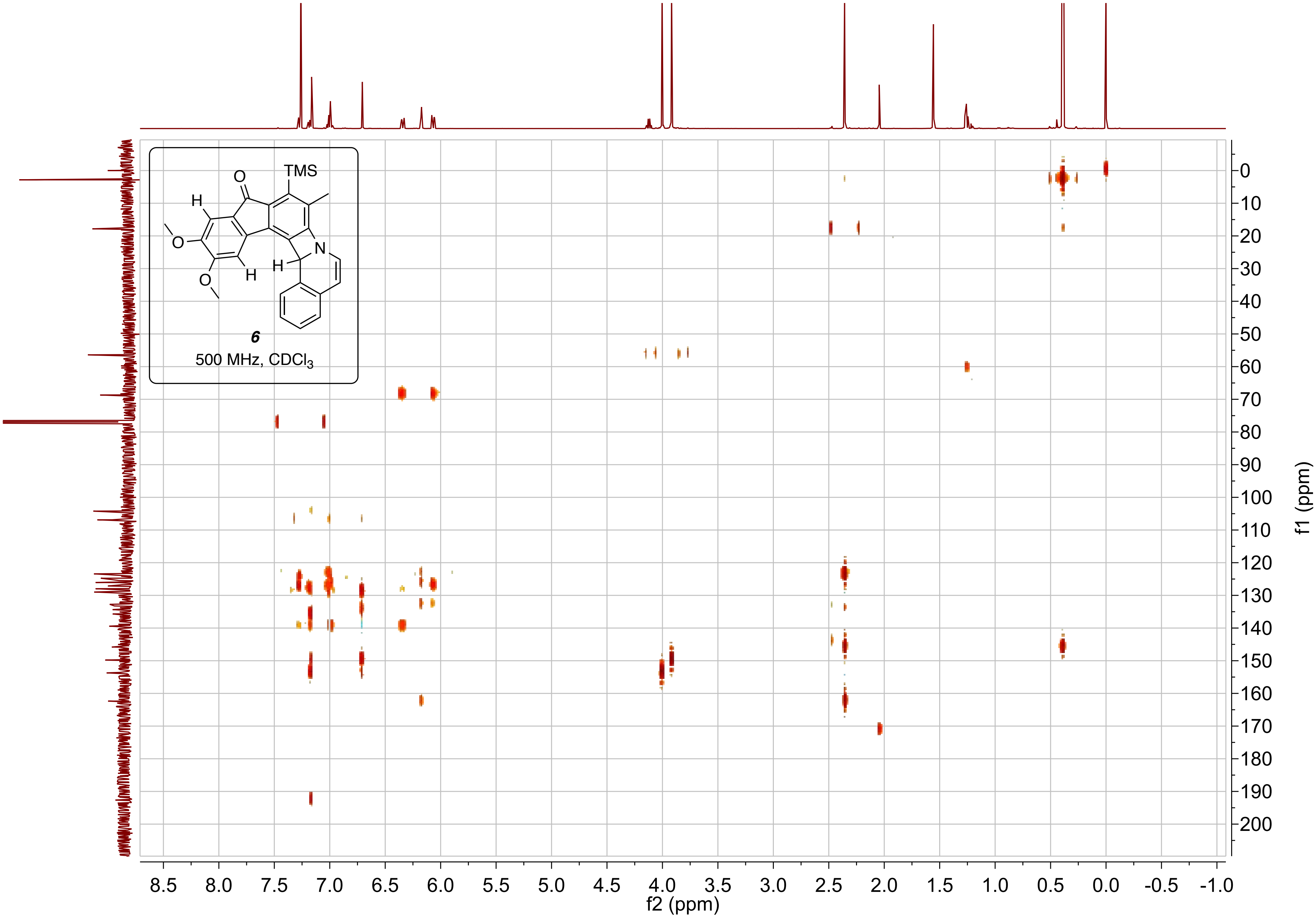
TMS
O
H
N
H
H
O
O
6
500 MHz, CDCl3
f2 (ppm)
f1 (ppm)

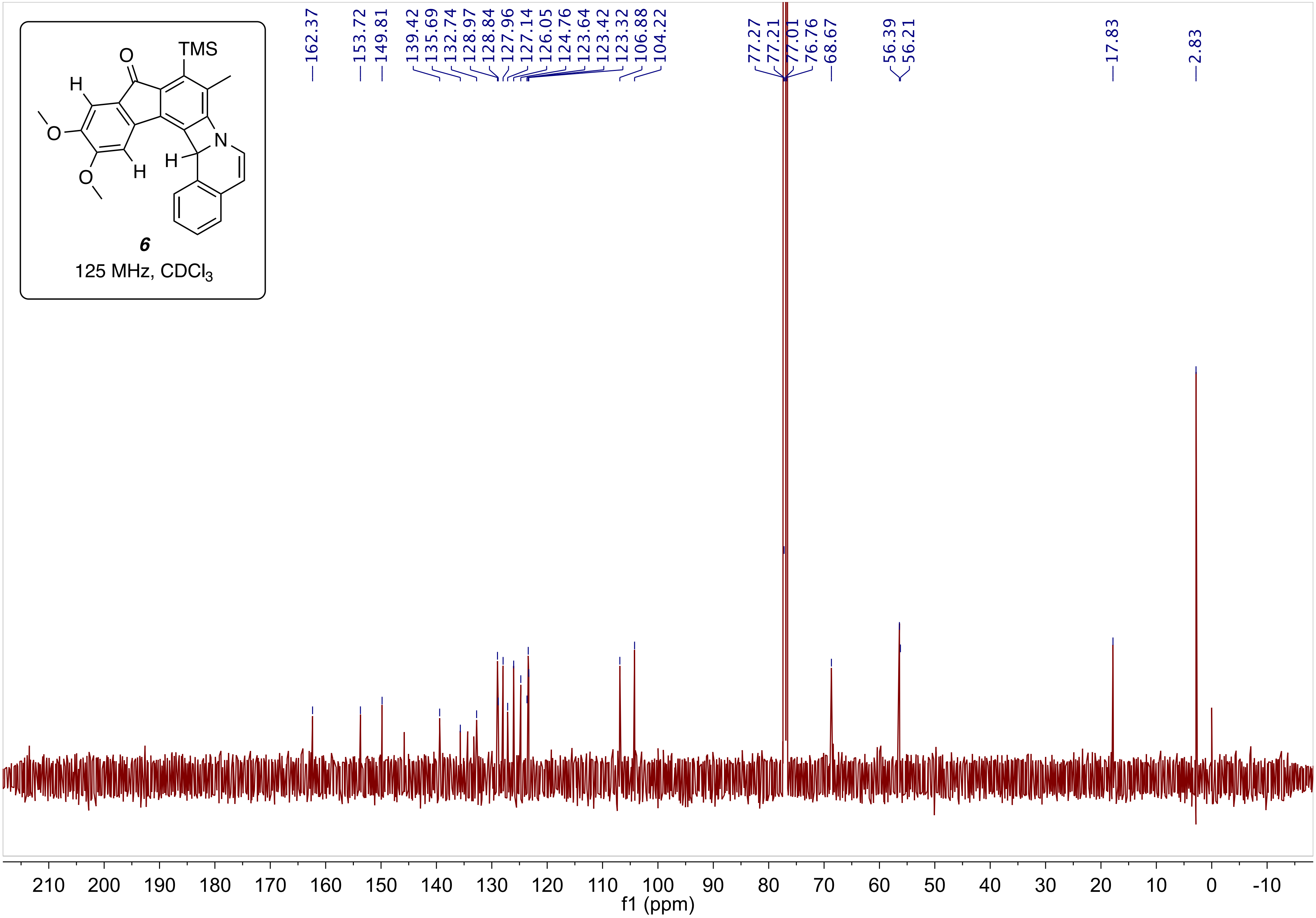

TMS
O
H
O
O
H
H
N
6
125 MHz, $CDCl_3$
162.37
153.72
149.81
139.42
135.69
132.74
128.97
128.84
127.96
127.14
126.05
124.76
123.64
123.42
123.32
106.88
104.22
77.27
77.21
77.01
76.76
68.67
56.39
56.21
17.83
2.83
210 200 190 180 170 160 150 140 130 120 110 100 90 80 70 60 50 40 30 20 10 0 -10
f1 (ppm)

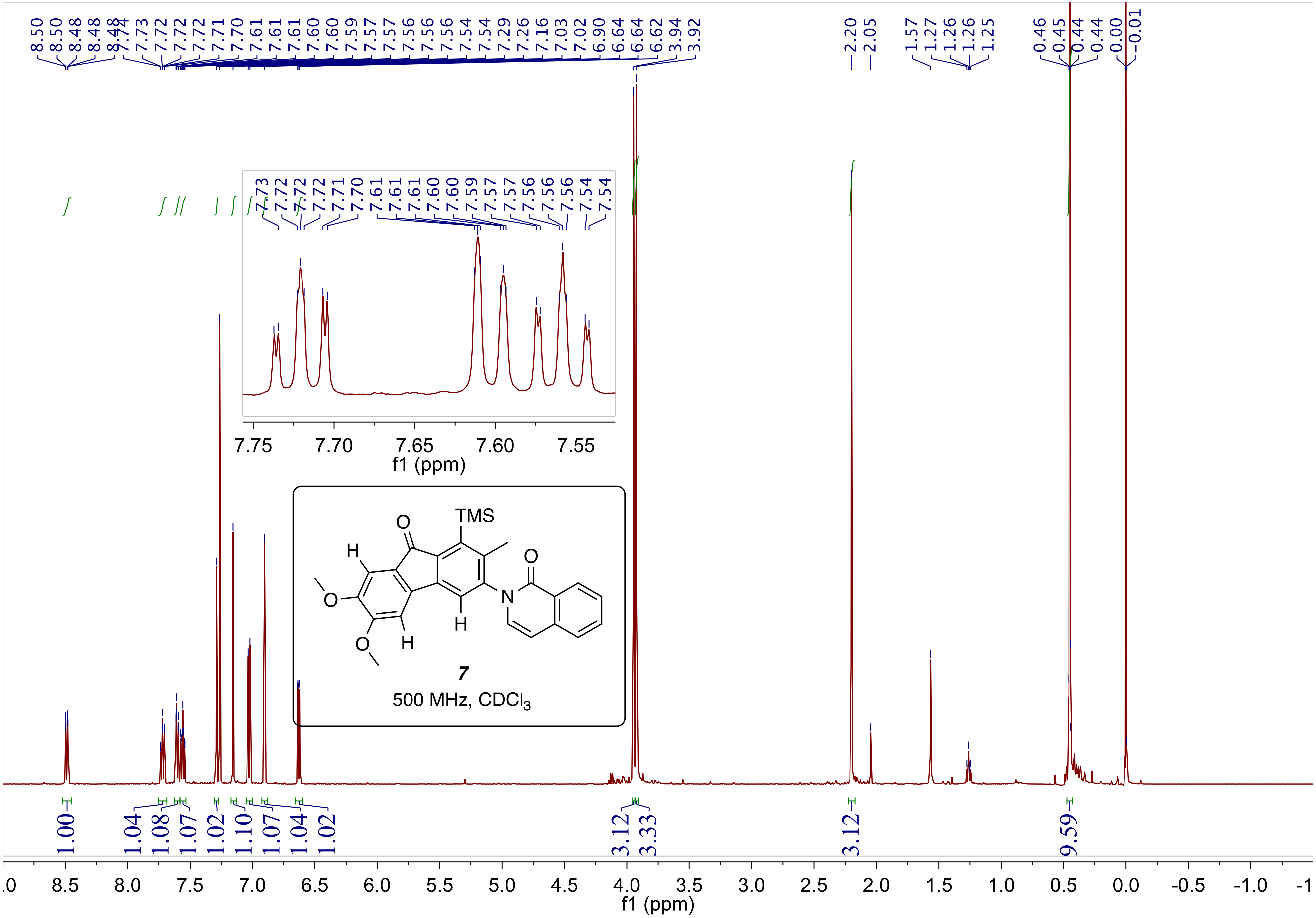
TMS
O
H
O
N
H
O
H
O
7
500 MHz, $CDCl_3$
f1 (ppm)

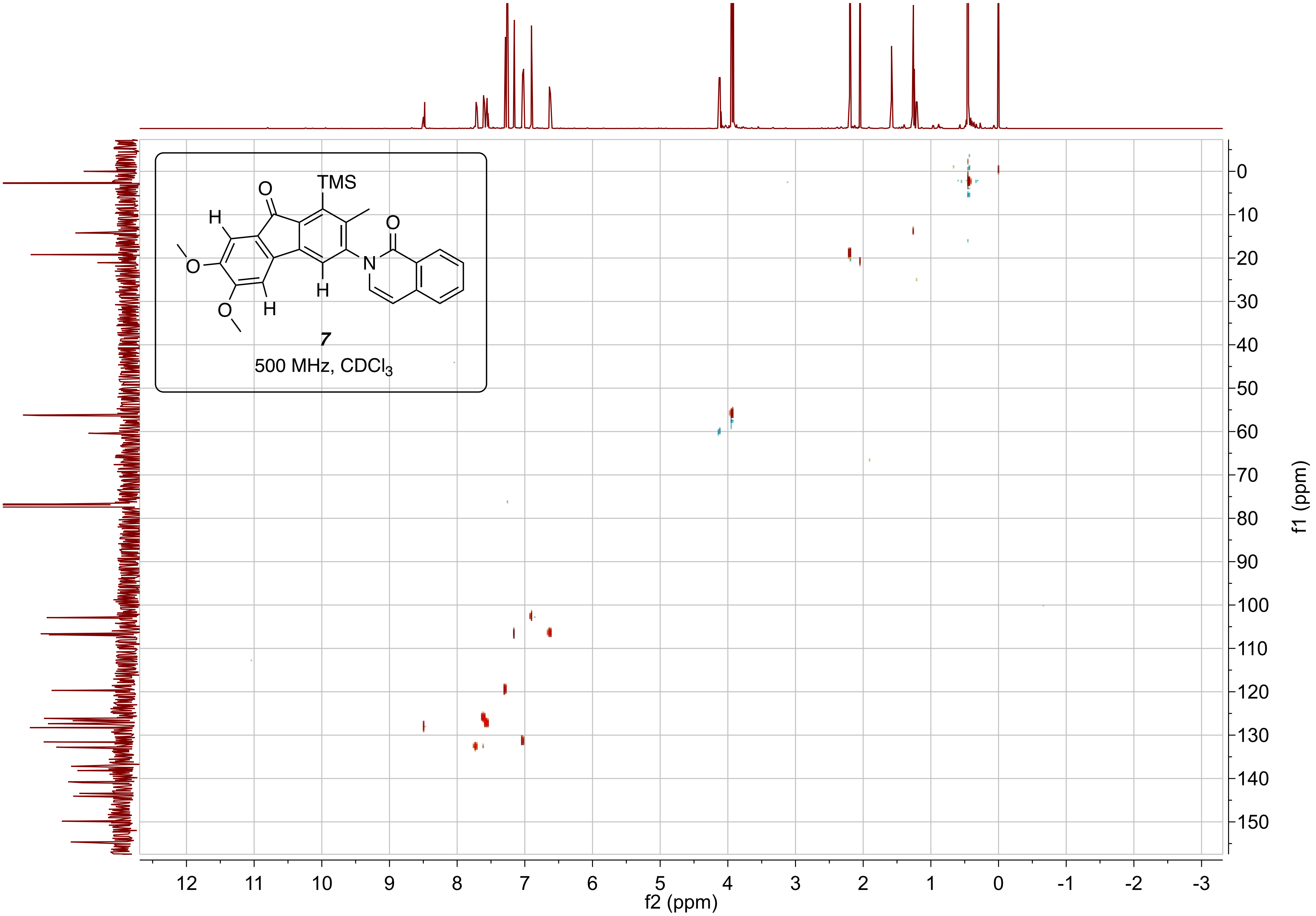
TMS
O
O
H
O
N
O
H
H
7
500 MHz, CDCl3
f2 (ppm)
f1 (ppm)

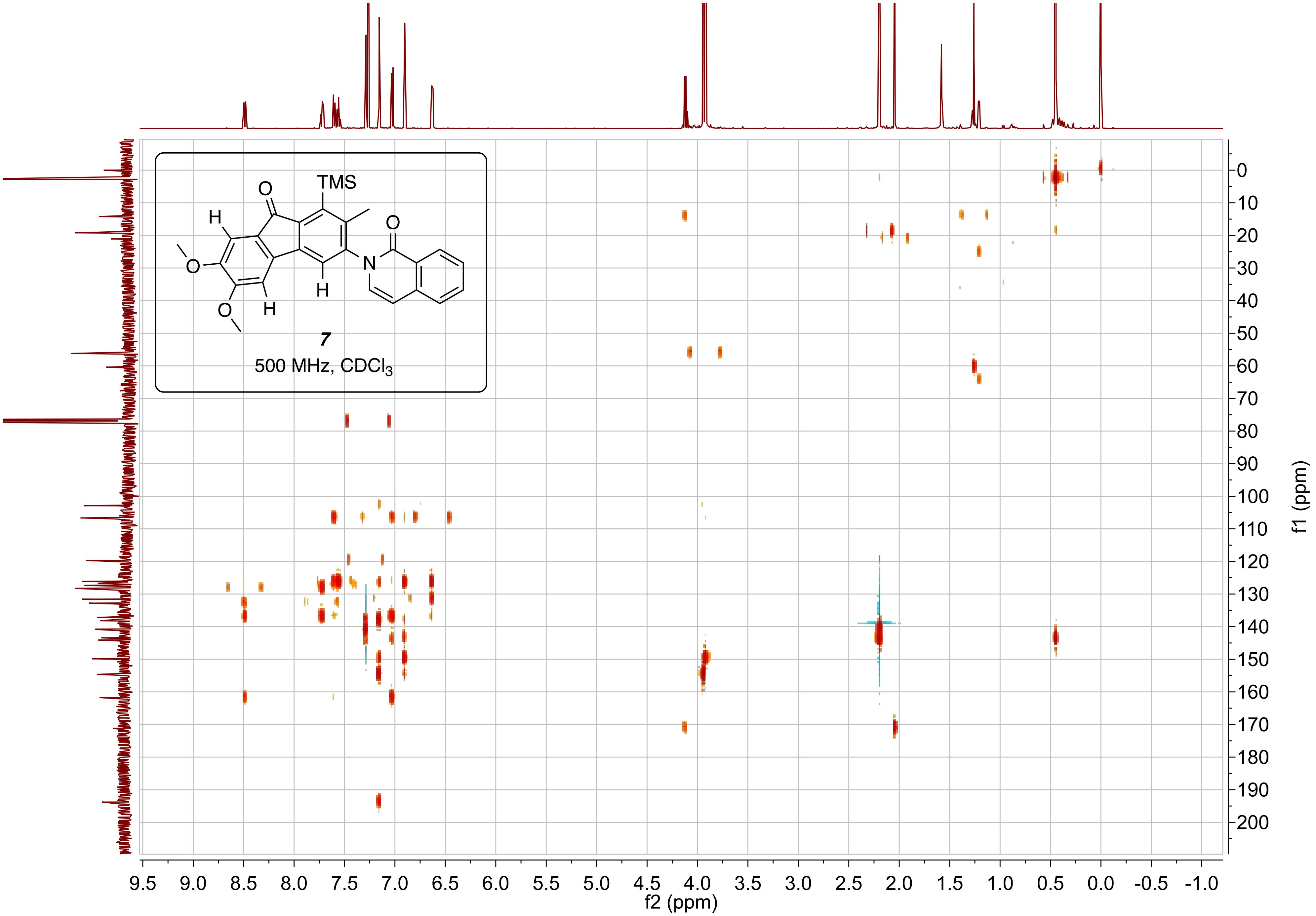
TMS
7
500 MHz, CDCl3
f2 (ppm)
f1 (ppm)

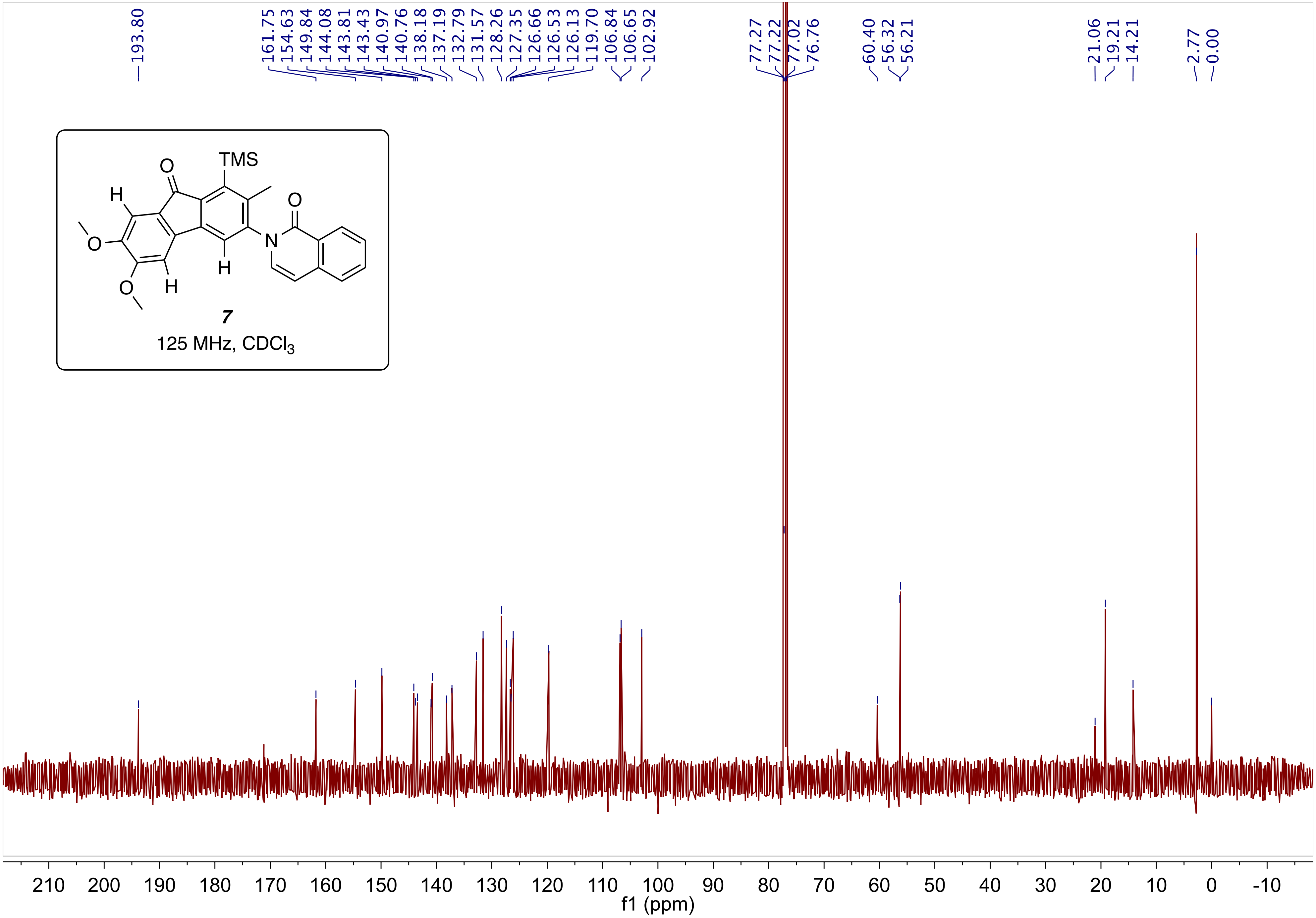
193.80
161.75
154.63
149.84
144.08
143.81
143.43
140.97
140.76
138.18
137.19
132.79
131.57
128.26
127.35
126.66
126.53
126.13
119.70
106.84
106.65
102.92
77.27
77.22
77.02
76.76
60.40
56.32
56.21
21.06
19.21
14.21
2.77
0.00
TMS
O
H
O
O
N
O
H
H
7
125 MHz, CDCl3
210 200 190 180 170 160 150 140 130 120 110 100 90 80 70 60 50 40 30 20 10 0 -10
f1 (ppm)

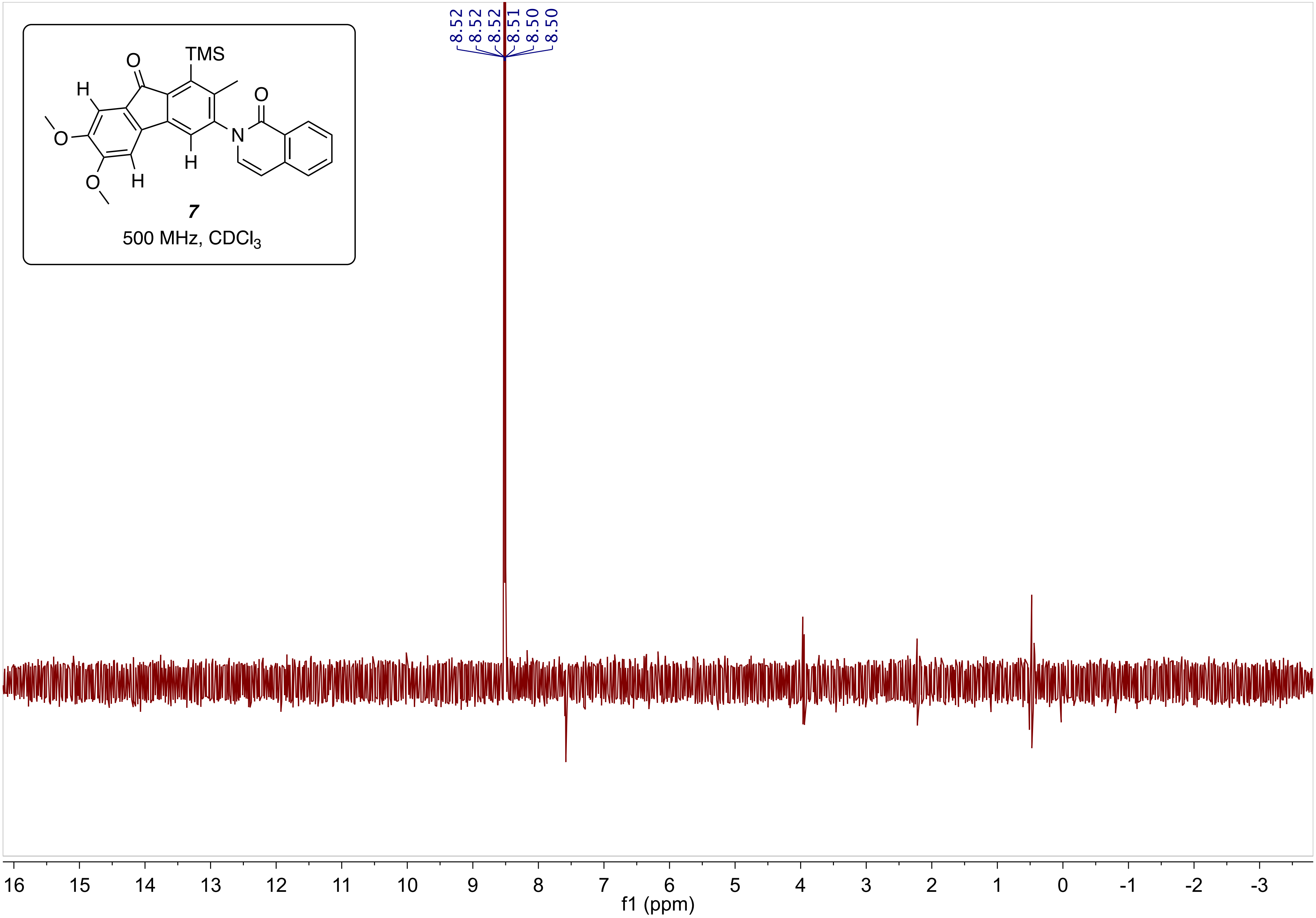
TMS
O
H
O
N
O
H
O
H
7
500 MHz, CDCl3
8.52
8.52
8.52
8.51
8.50
8.50
16
15
14
13
12
11
10
9
8
7
6
5
4
3
2
1
0
-1
-2
-3
f1 (ppm)

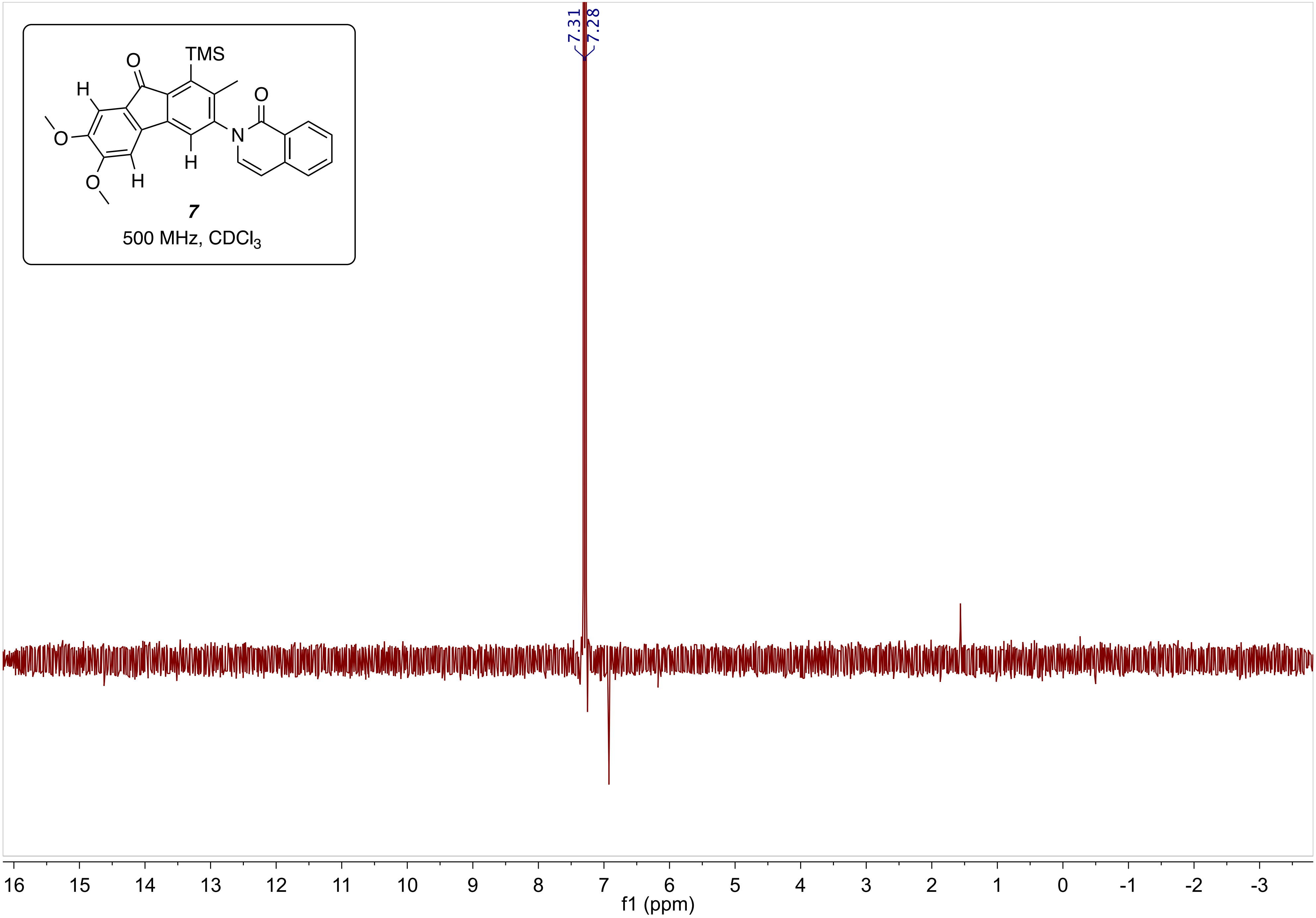
TMS
O
H
O
N
O
O
H
H
7
500 MHz, $CDCl_3$
7.31
7.28
16 15 14 13 12 11 10 9 8 7 6 5 4 3 2 1 0 -1 -2 -3
f1 (ppm)

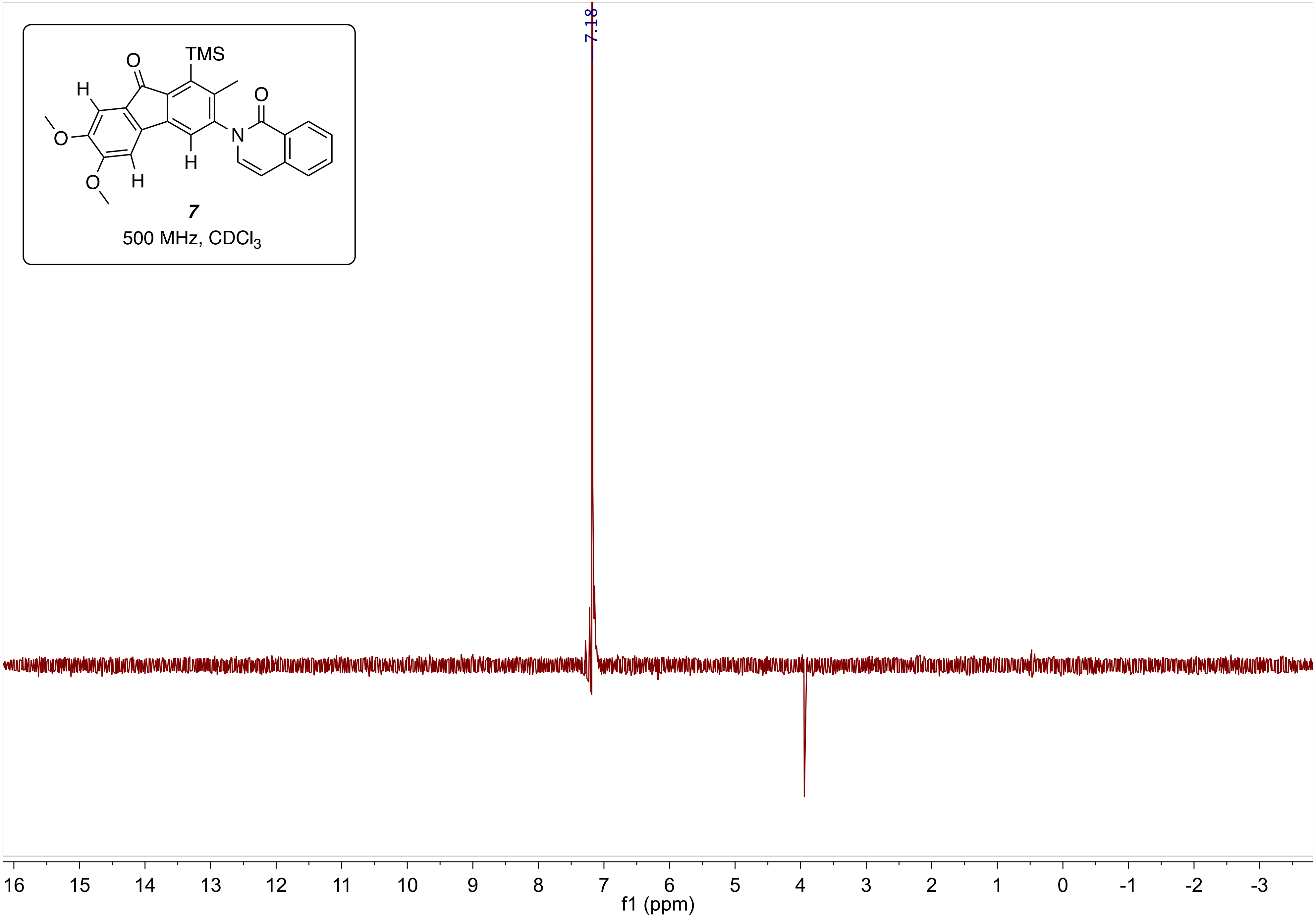
TMS
O
O
H
O
N
O
H
H
7
500 MHz, CDCl3
7.18
16
15
14
13
12
11
10
9
8
7
6
5
4
3
2
1
0
-1
-2
-3
f1 (ppm)

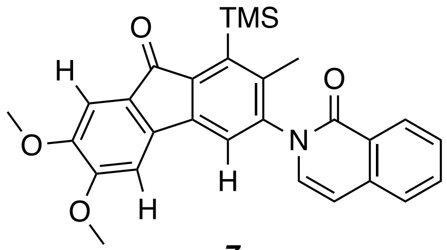

7

500 MHz, $CDCl_3$

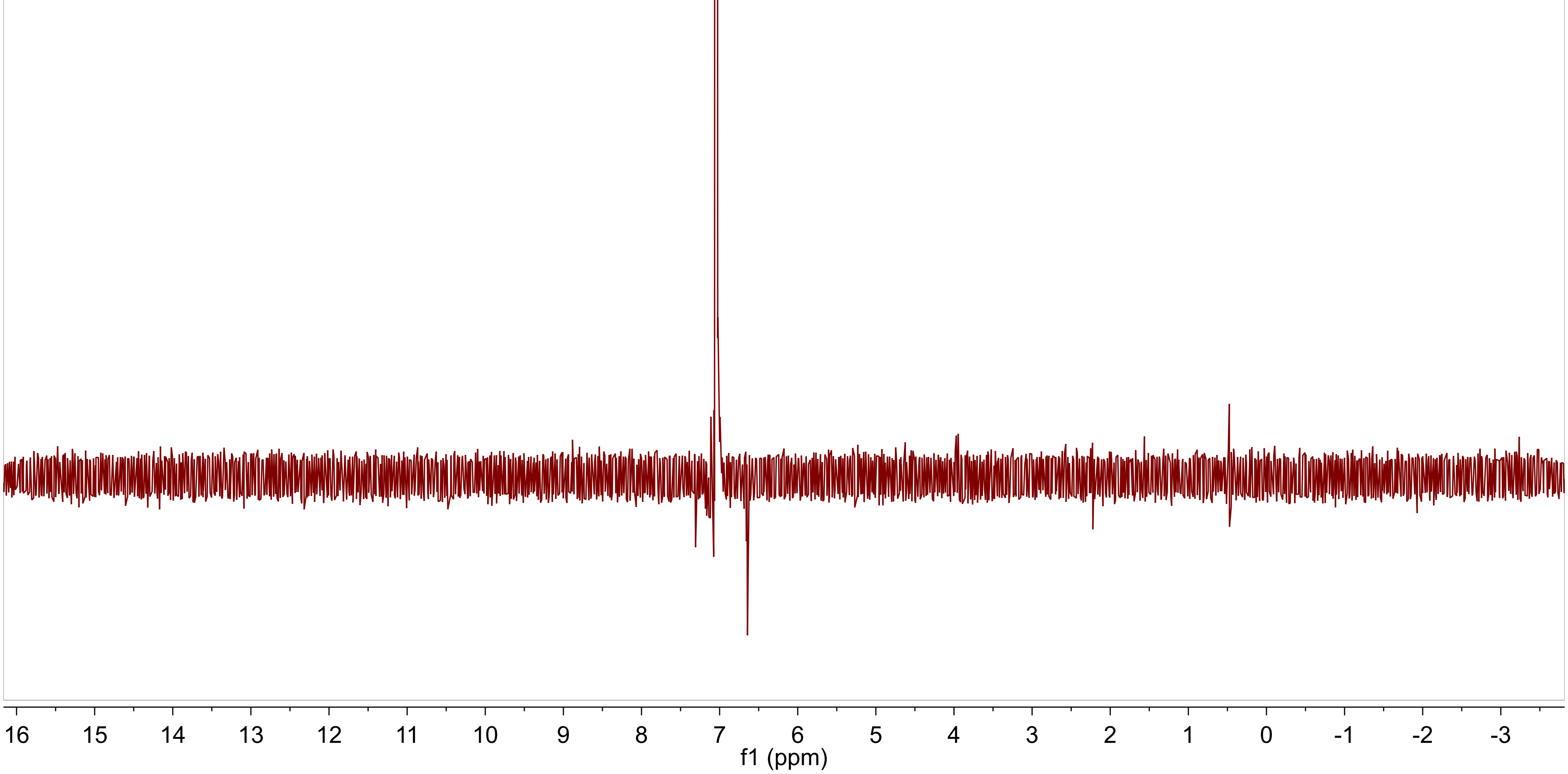

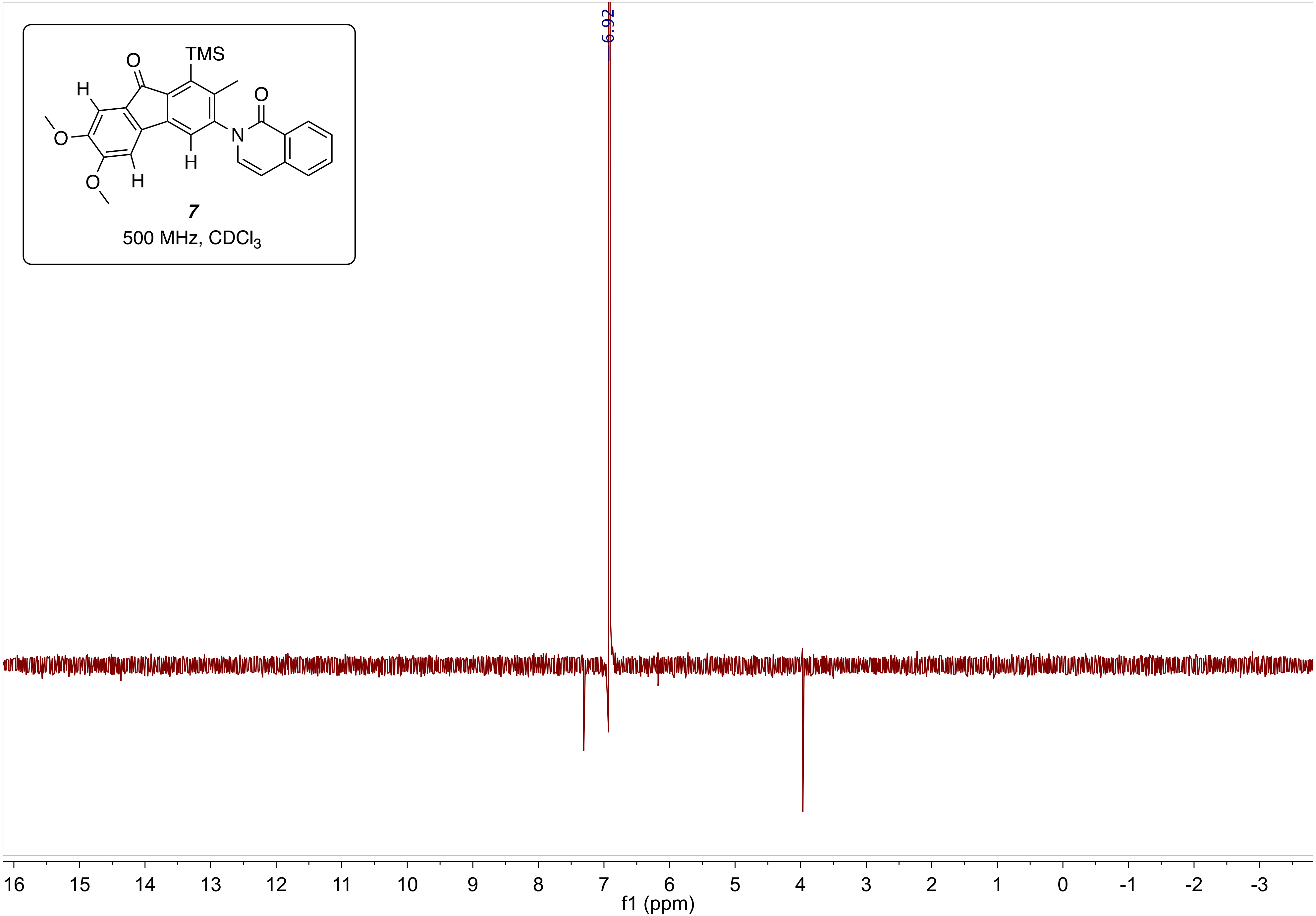
TMS
O
H
O
N
O
H
O
H
7
500 MHz, CDCl3
6.92
16
15
14
13
12
11
10
9
8
7
6
5
4
3
2
1
0
-1
-2
-3
f1 (ppm)

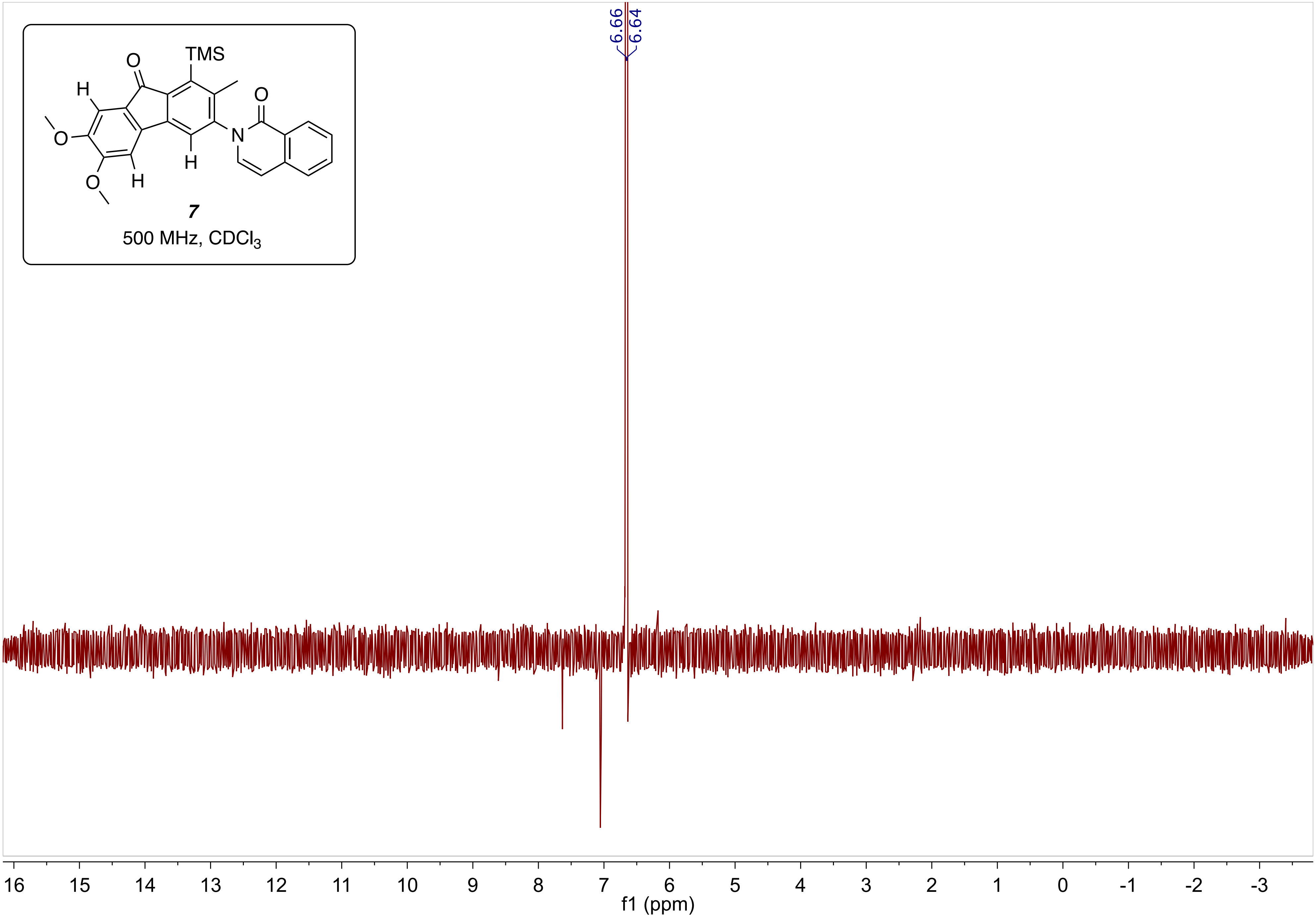

TMS
O
H
O
N
O
H
O
H
7
500 MHz, $CDCl_3$
6.66
6.64
16 15 14 13 12 11 10 9 8 7 6 5 4 3 2 1 0 -1 -2 -3
f1 (ppm)

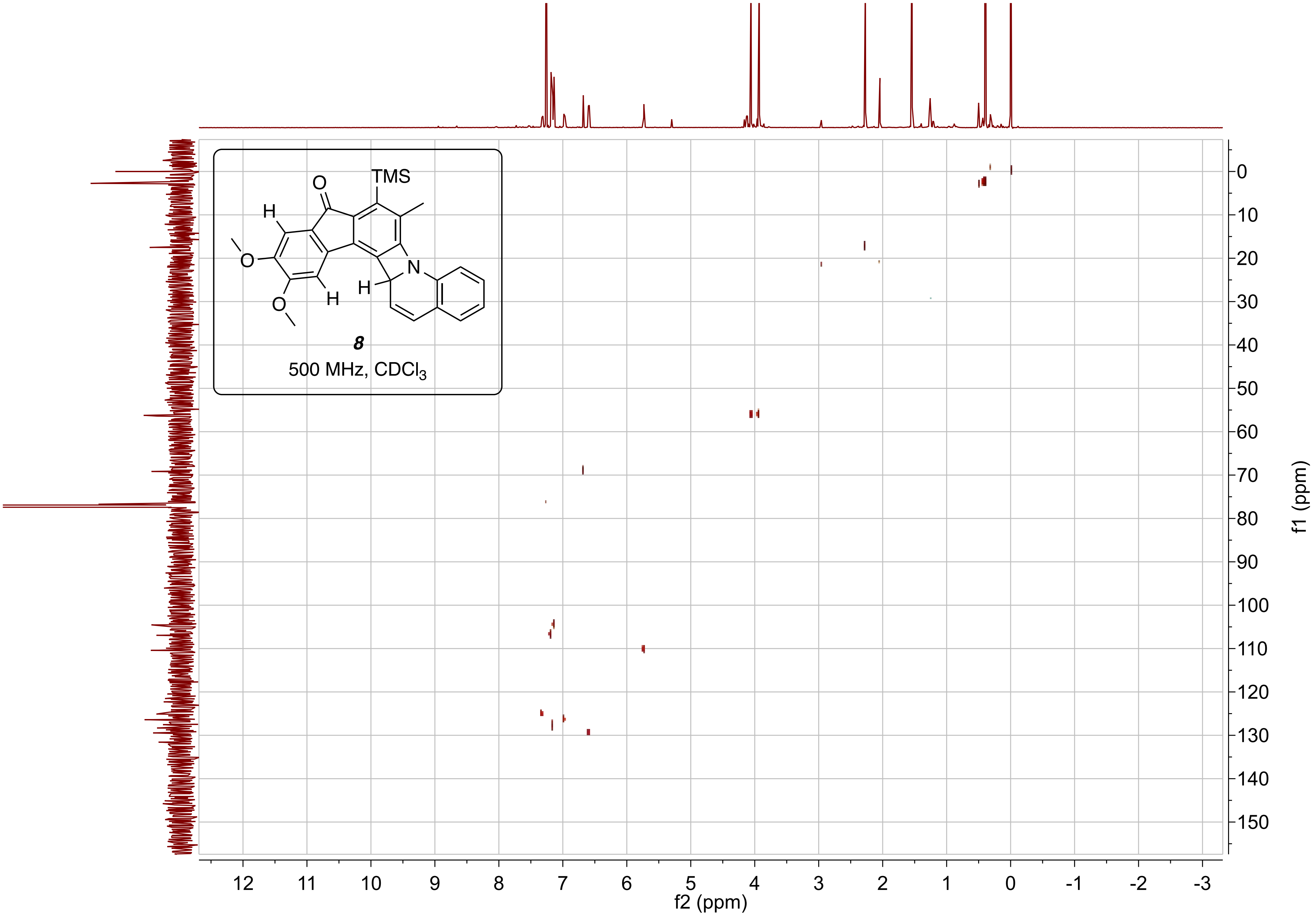

TMS
O
H
O
O
N
H
H
8
500 MHz, CDCl3
f2 (ppm)
f1 (ppm)

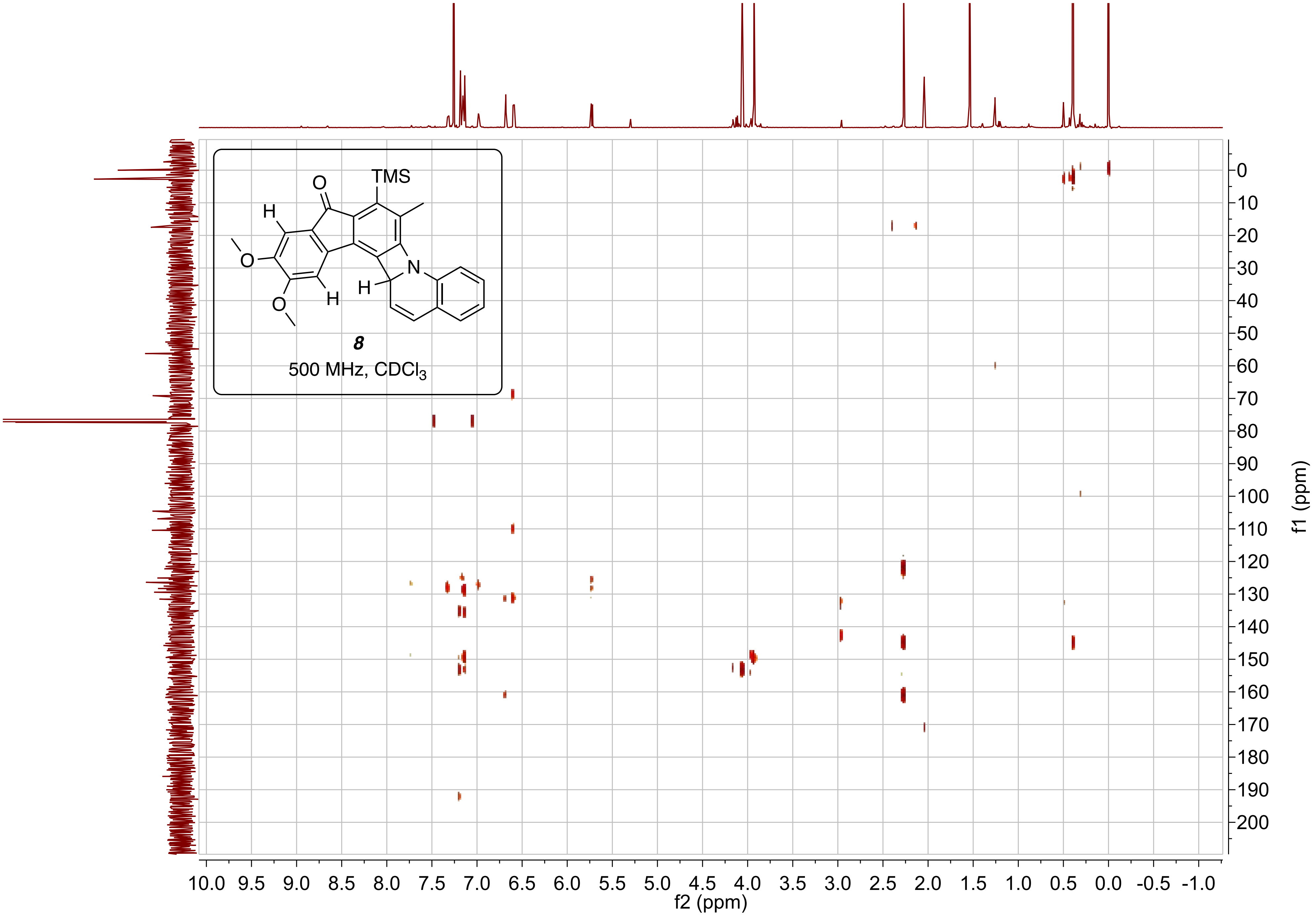
O
TMS
H
O
N
H
H
O
8
500 MHz, CDCl3
f2 (ppm)
f1 (ppm)

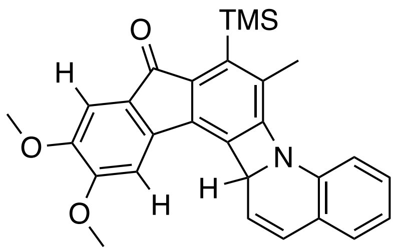

8

125 MHz, $CDCl_3$

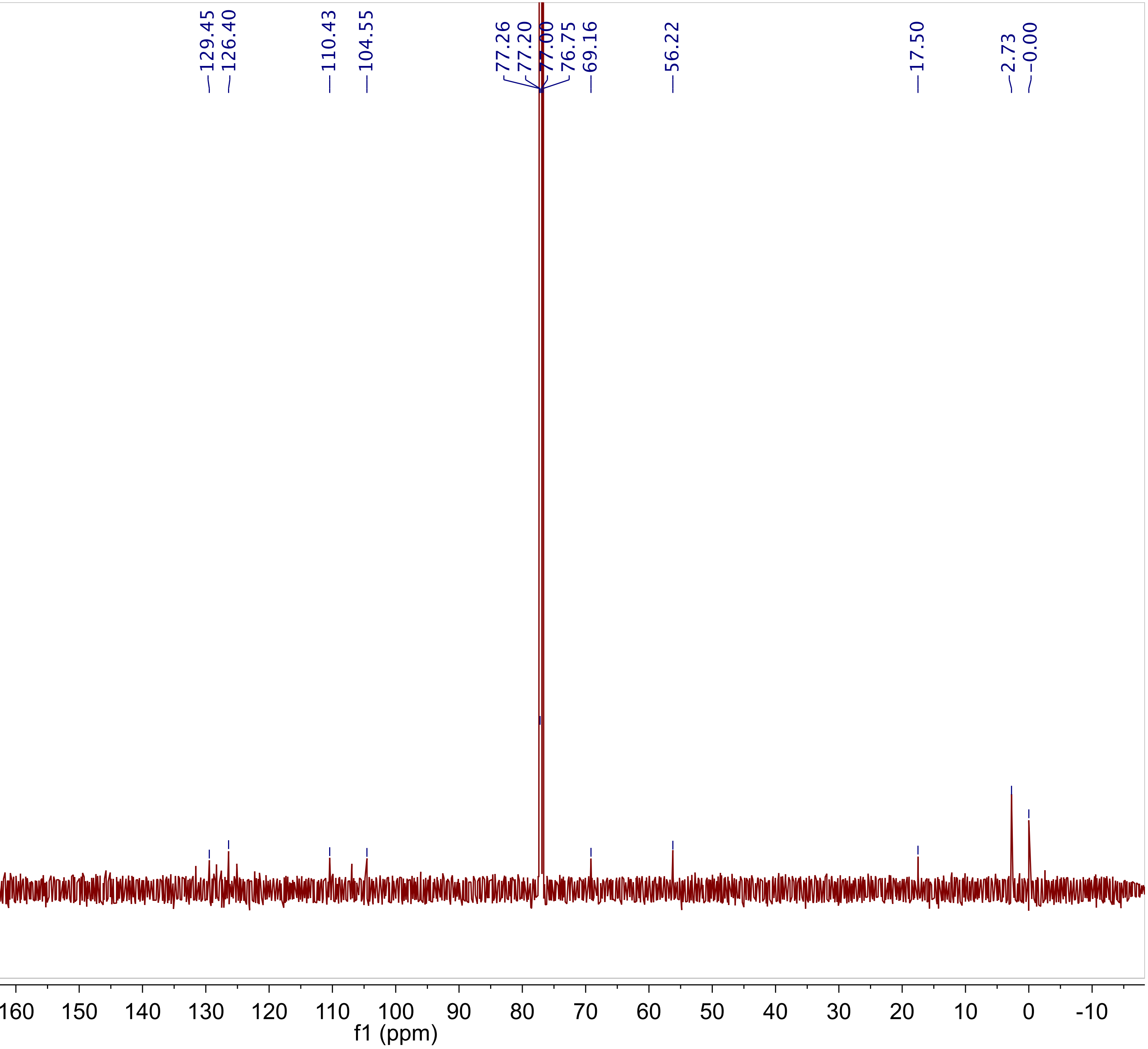

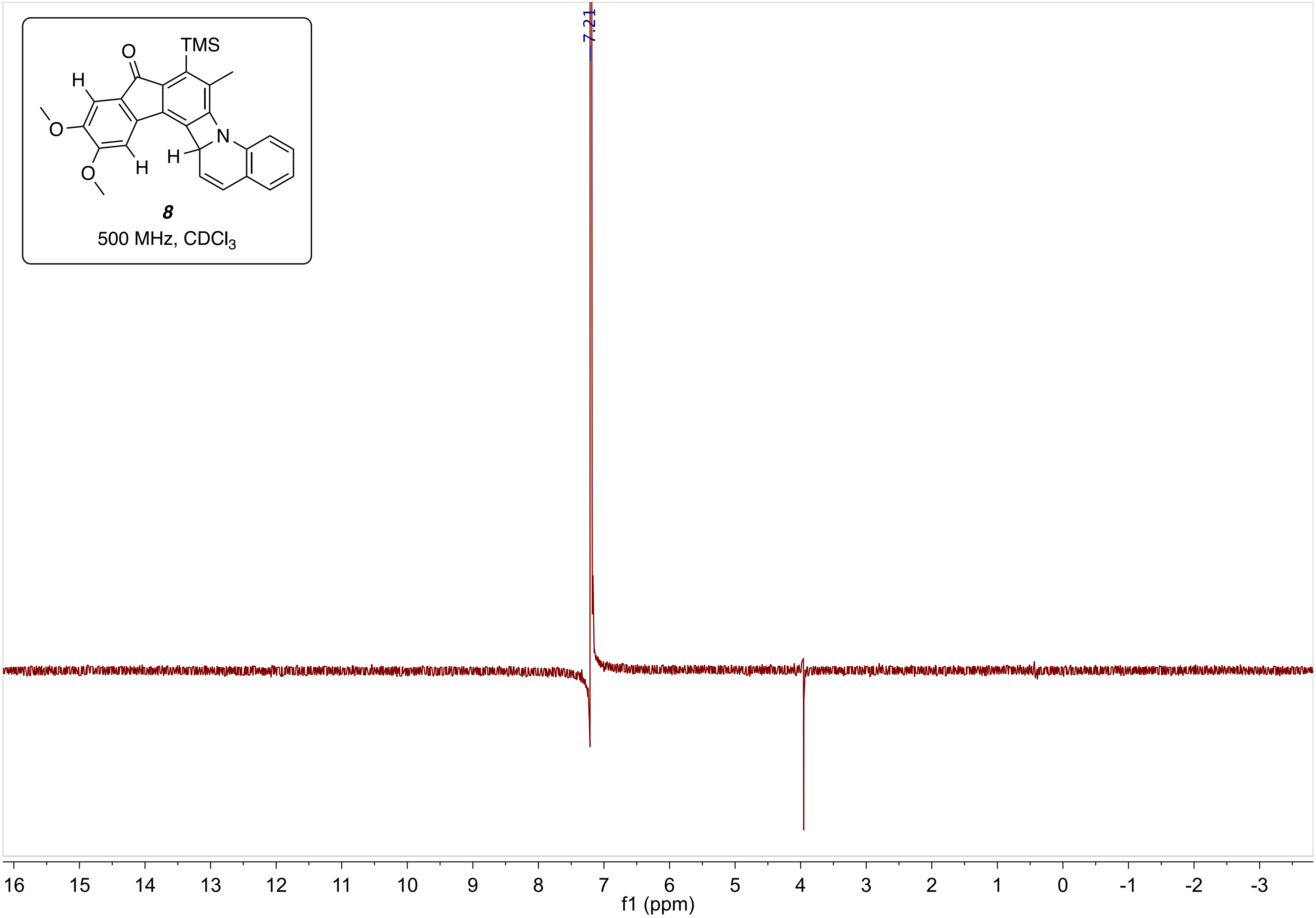
TMS
O
H
O
O
H
H
N
8
500 MHz, CDCl3
7.21
16 15 14 13 12 11 10 9 8 7 6 5 4 3 2 1 0 -1 -2 -3
f1 (ppm)

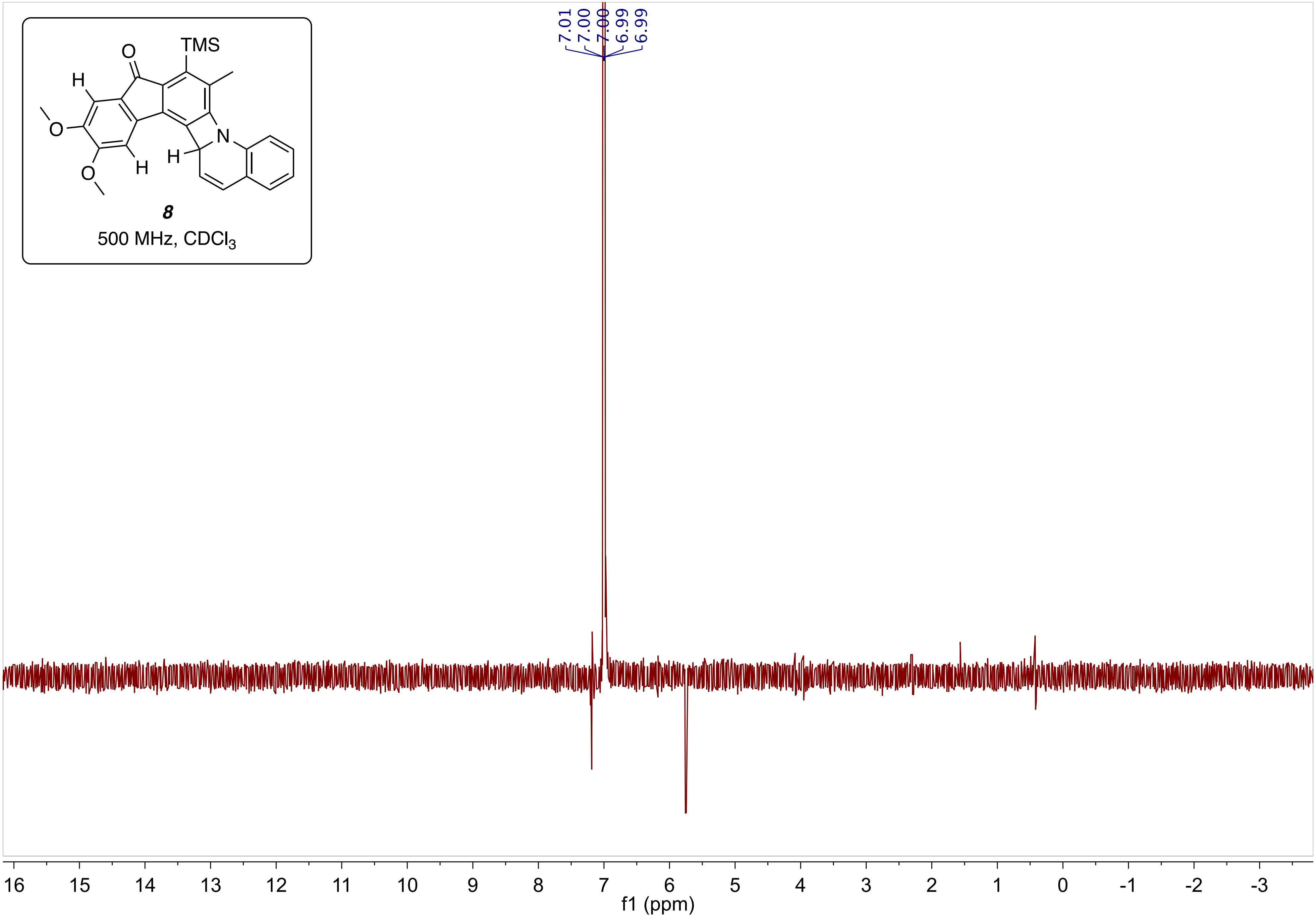

8

500 MHz, $CDCl_3$

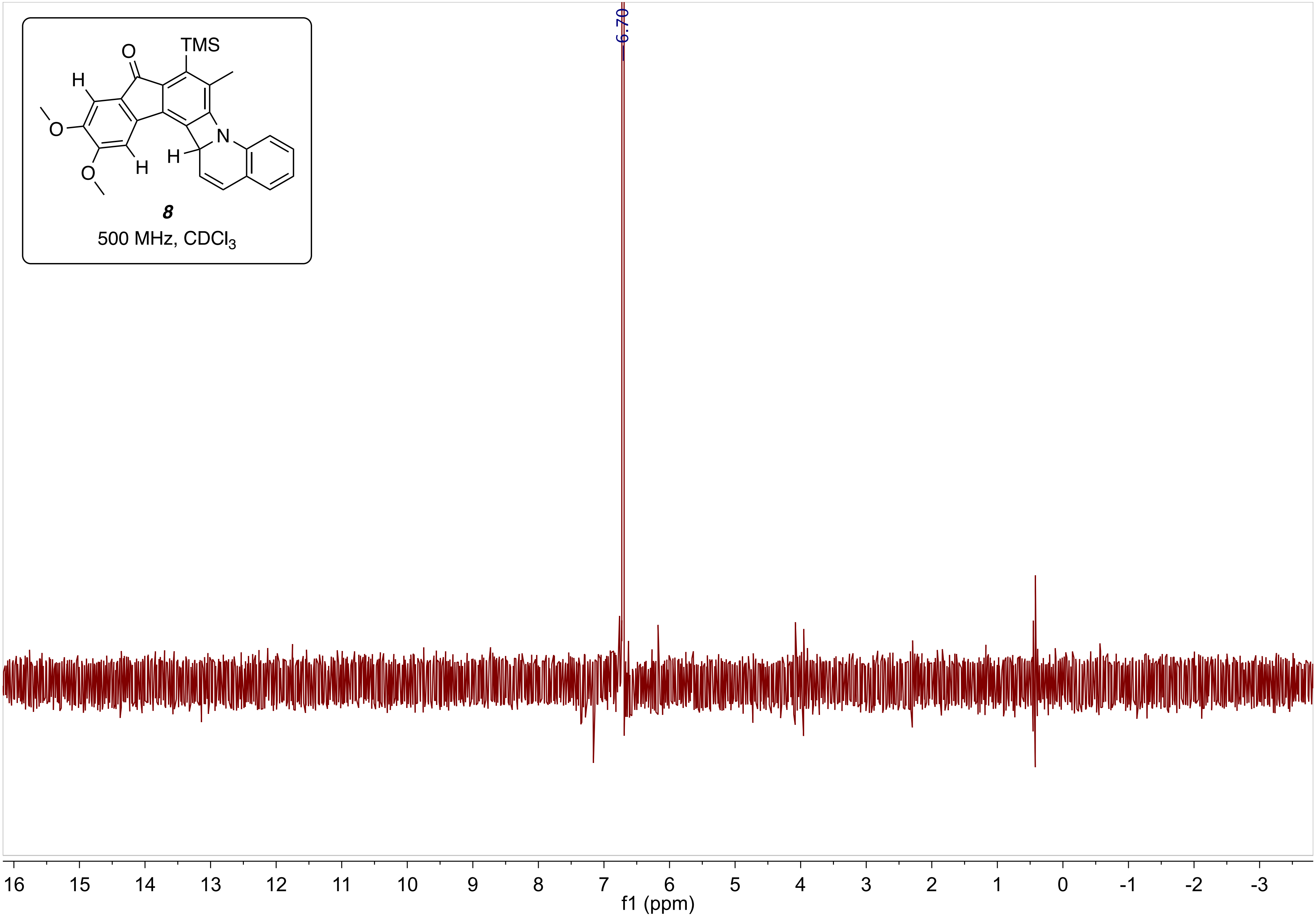
TMS
O
N
H
8
500 MHz, CDCl3
6.70
16 15 14 13 12 11 10 9 8 7 6 5 4 3 2 1 0 -1 -2 -3
f1 (ppm)

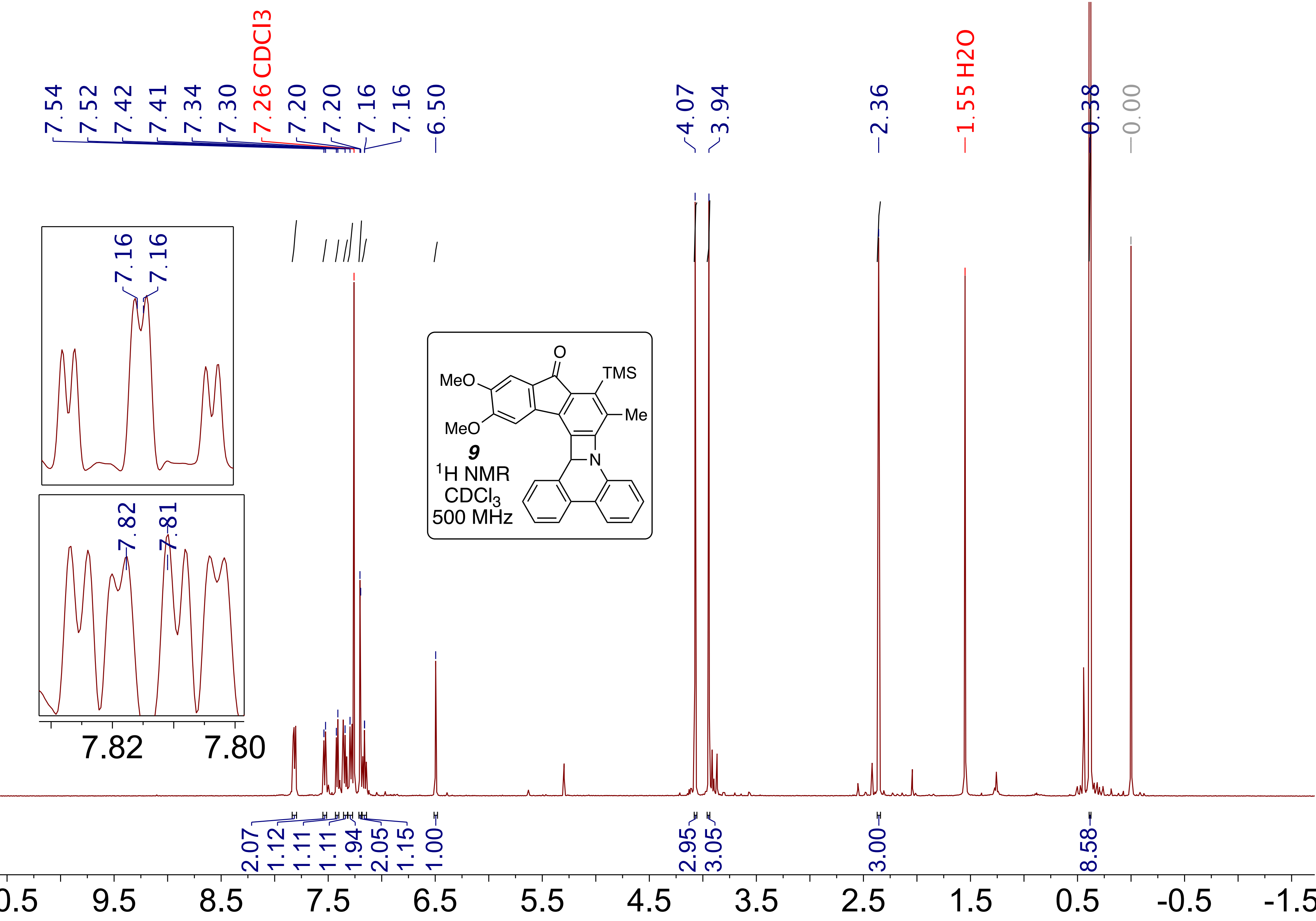
7.54
7.52
7.42
7.41
7.34
7.30
7.26 CDCl3
7.20
7.20
7.16
7.16
6.50
4.07
3.94
2.36
1.55 H2O
0.38
0.00
7.16
7.16
7.82
7.81
7.82
7.80
O
TMS
MeO
Me
MeO
N
9
1H NMR
CDCl3
500 MHz
2.07
1.12
1.11
1.11
1.94
2.05
1.15
1.00
2.95
3.05
3.00
8.58
0.5
9.5
8.5
7.5
6.5
5.5
4.5
3.5
2.5
1.5
0.5
-0.5
-1.5

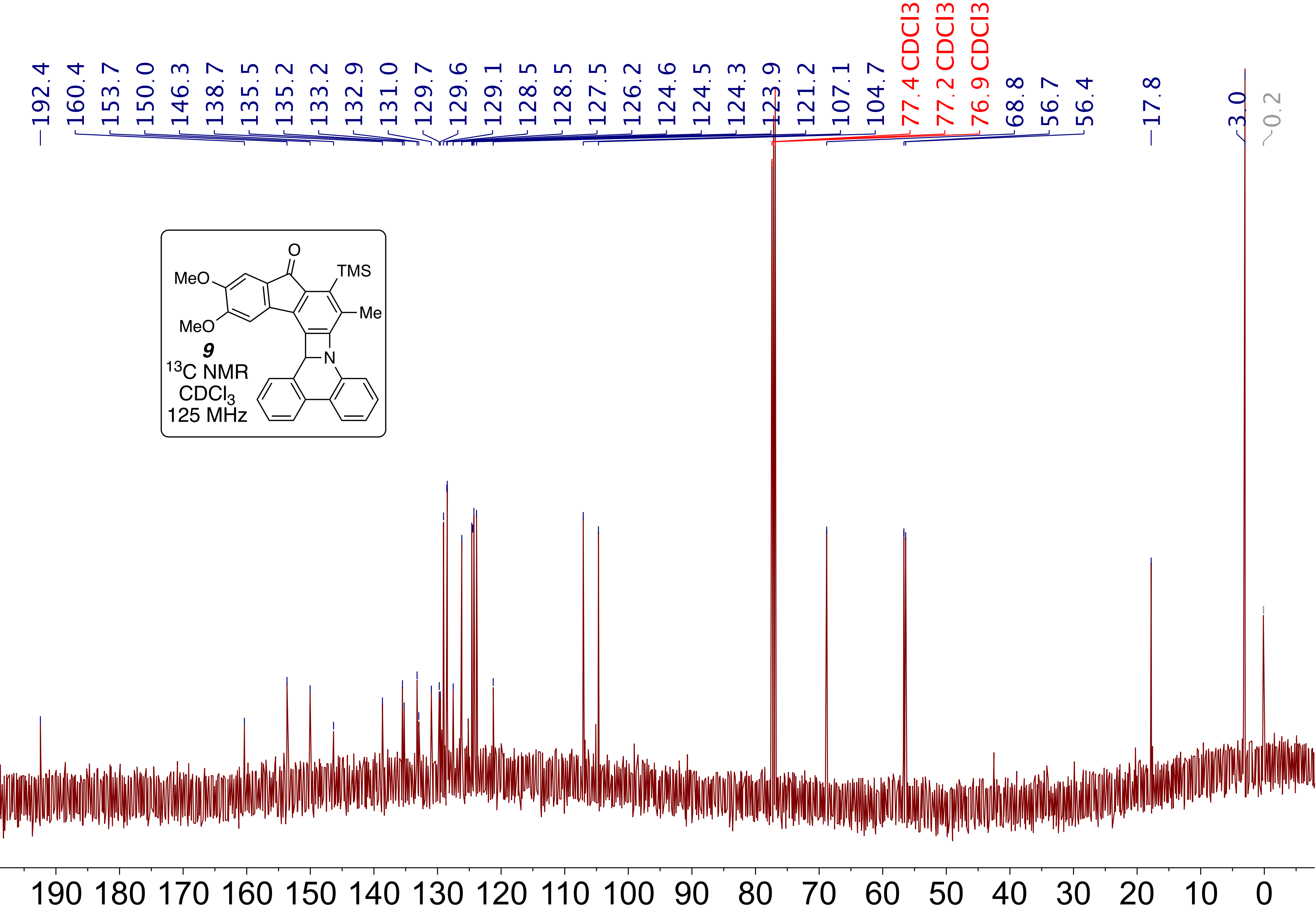
192.4
160.4
153.7
150.0
146.3
138.7
135.5
135.2
133.2
132.9
131.0
129.7
129.6
129.1
128.5
128.5
127.5
126.2
124.6
124.5
124.3
123.9
121.2
107.1
104.7
77.4 CDCl3
77.2 CDCl3
76.9 CDCl3
68.8
56.7
56.4
17.8
3.0
0.2
O
TMS
MeO
Me
MeO
N
9
13C NMR
CDCl3
125 MHz
190 180 170 160 150 140 130 120 110 100 90 80 70 60 50 40 30 20 10 0

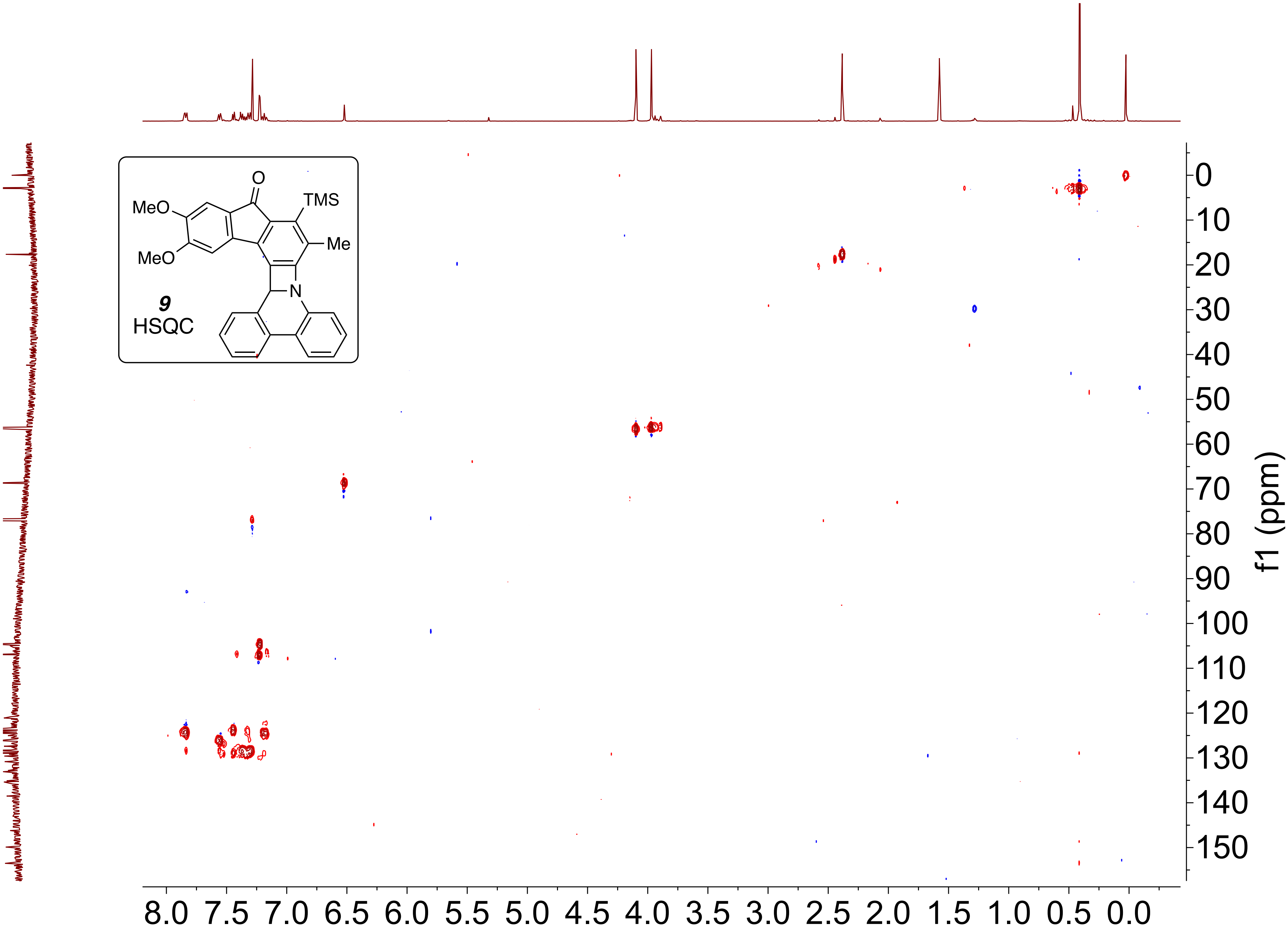
O
MeO
TMS
Me
MeO
N
9
HSQC
0
10
20
30
40
50
60
70
80
90
100
110
120
130
140
150
f1 (ppm)
8.0 7.5 7.0 6.5 6.0 5.5 5.0 4.5 4.0 3.5 3.0 2.5 2.0 1.5 1.0 0.5 0.0

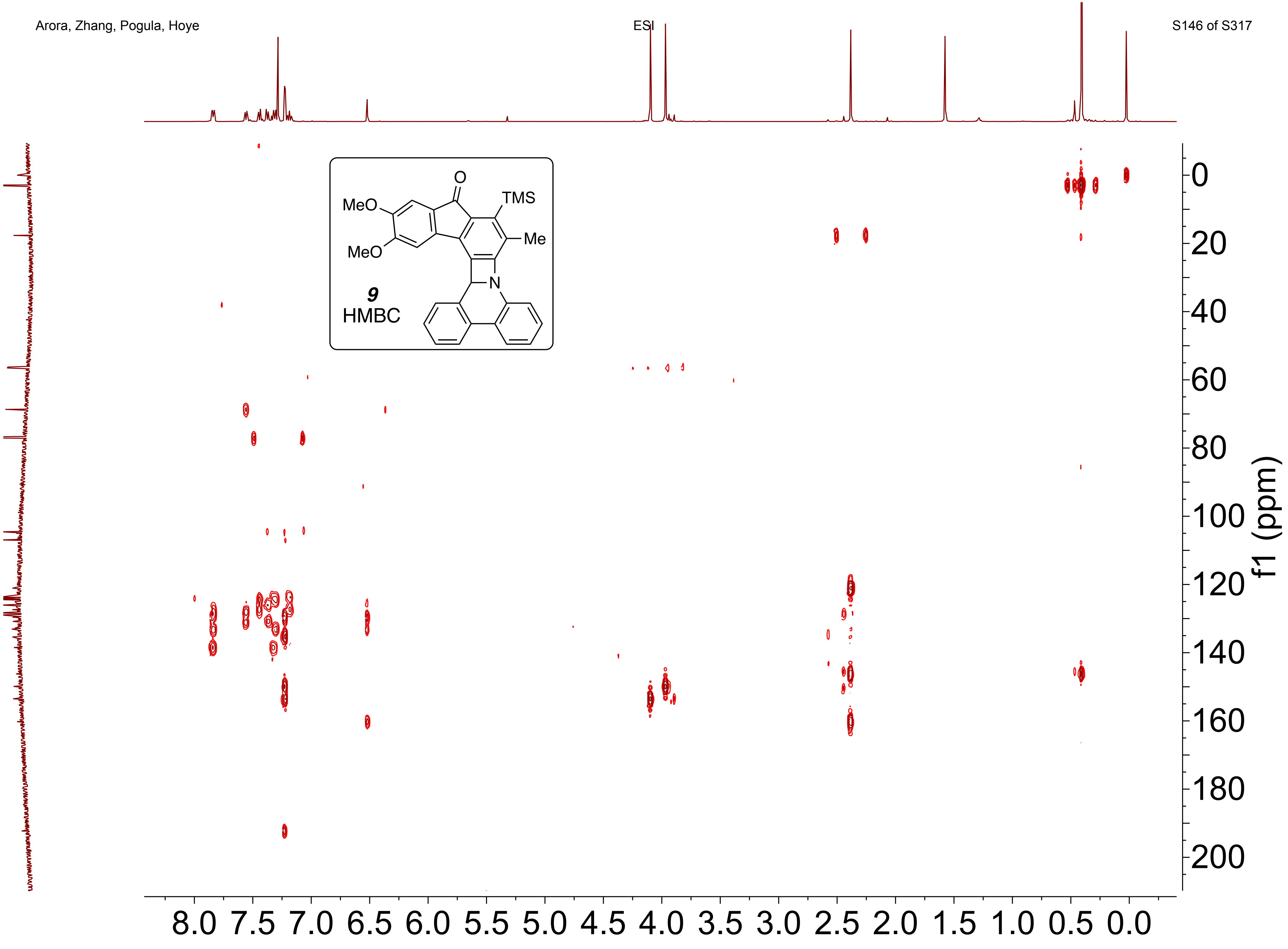
MeO
MeO
O
TMS
Me
N
9
HMBC
f1 (ppm)
0
20
40
60
80
100
120
140
160
180
200
8.0 7.5 7.0 6.5 6.0 5.5 5.0 4.5 4.0 3.5 3.0 2.5 2.0 1.5 1.0 0.5 0.0

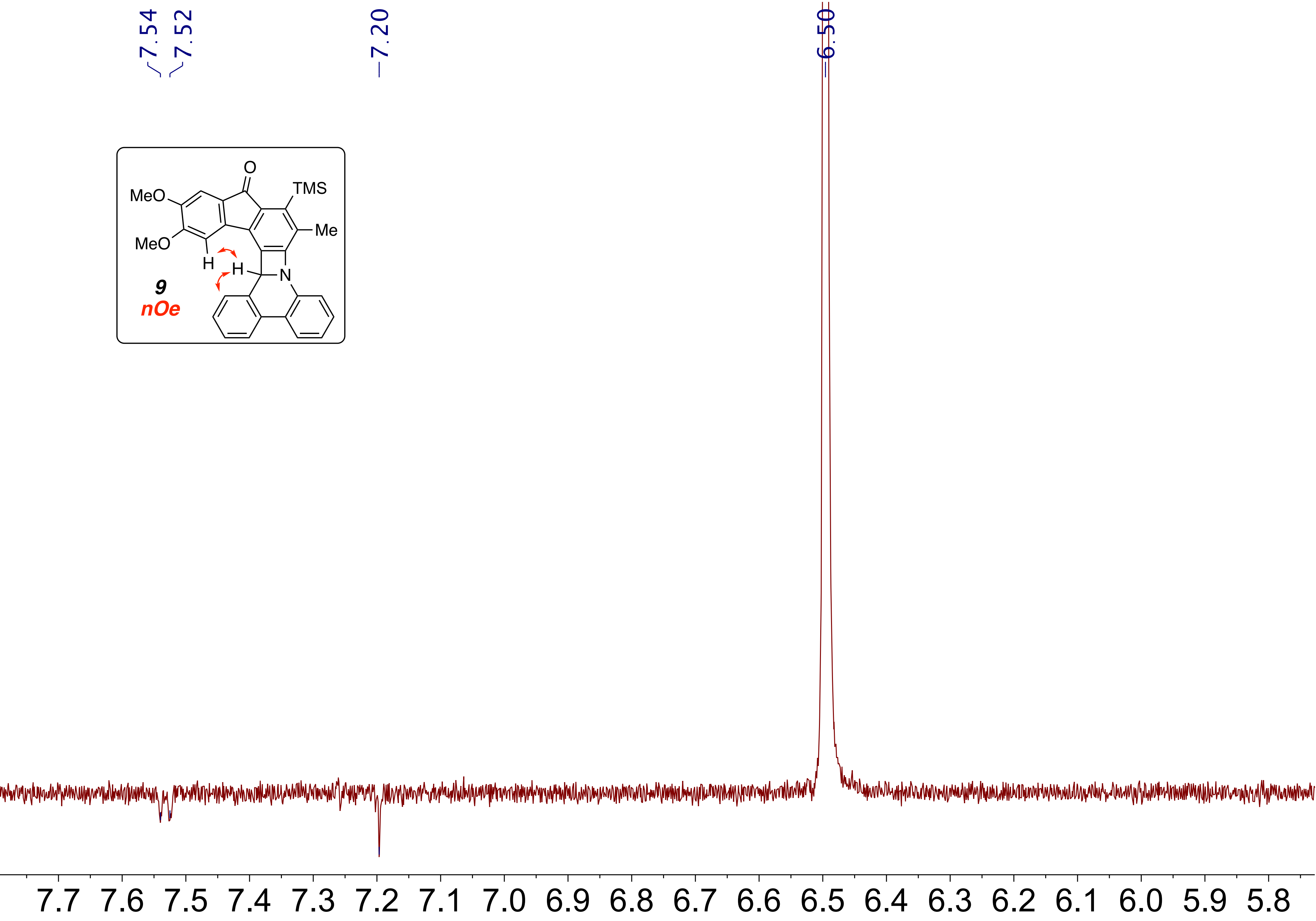
7.54
7.52
7.20
6.50
O
TMS
MeO
Me
MeO
H
H
N
9
nOe
7.7 7.6 7.5 7.4 7.3 7.2 7.1 7.0 6.9 6.8 6.7 6.6 6.5 6.4 6.3 6.2 6.1 6.0 5.9 5.8

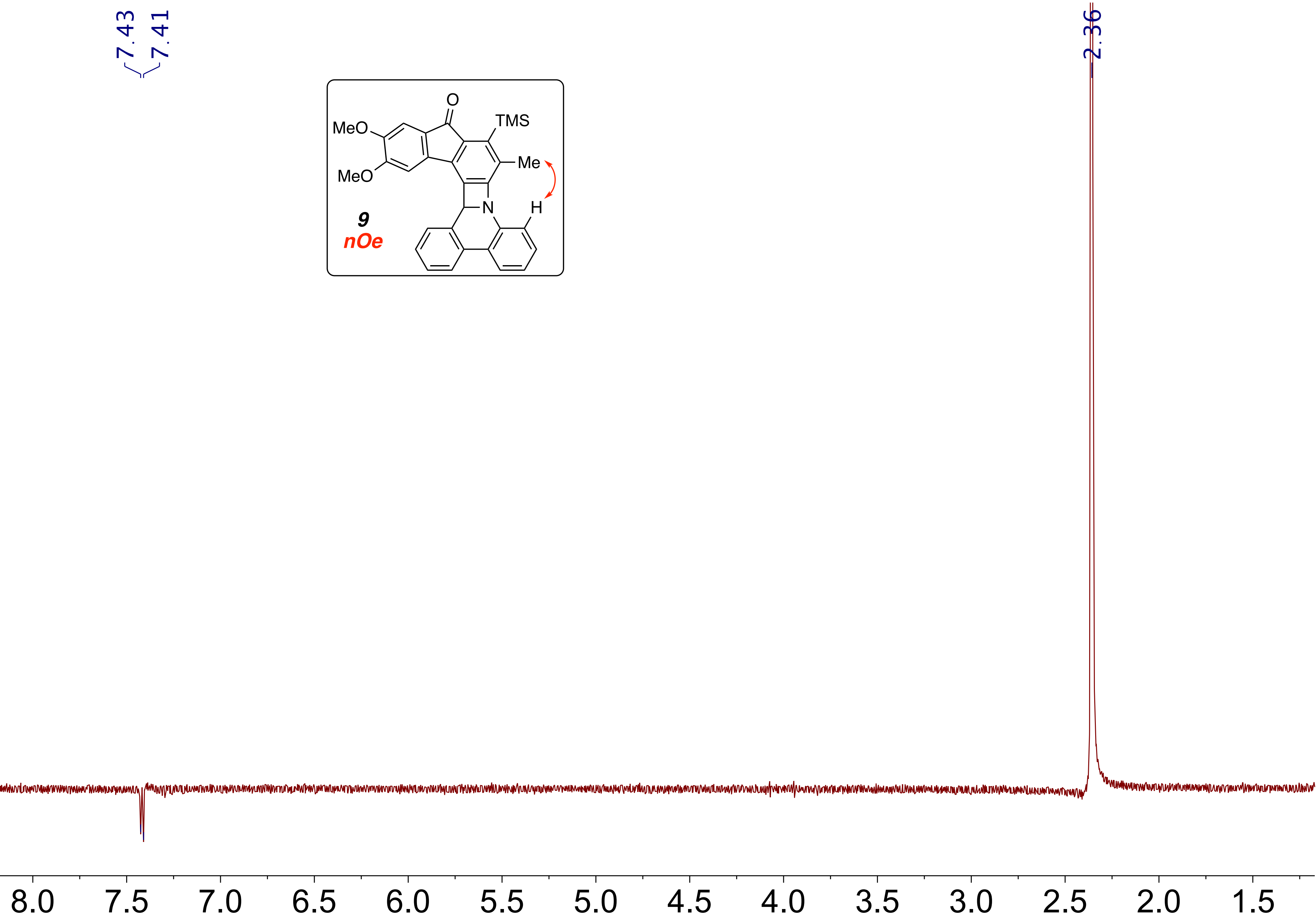
7.43
7.41
2.36
O
TMS
MeO
MeO
Me
N
H
9
nOe
8.0
7.5
7.0
6.5
6.0
5.5
5.0
4.5
4.0
3.5
3.0
2.5
2.0
1.5

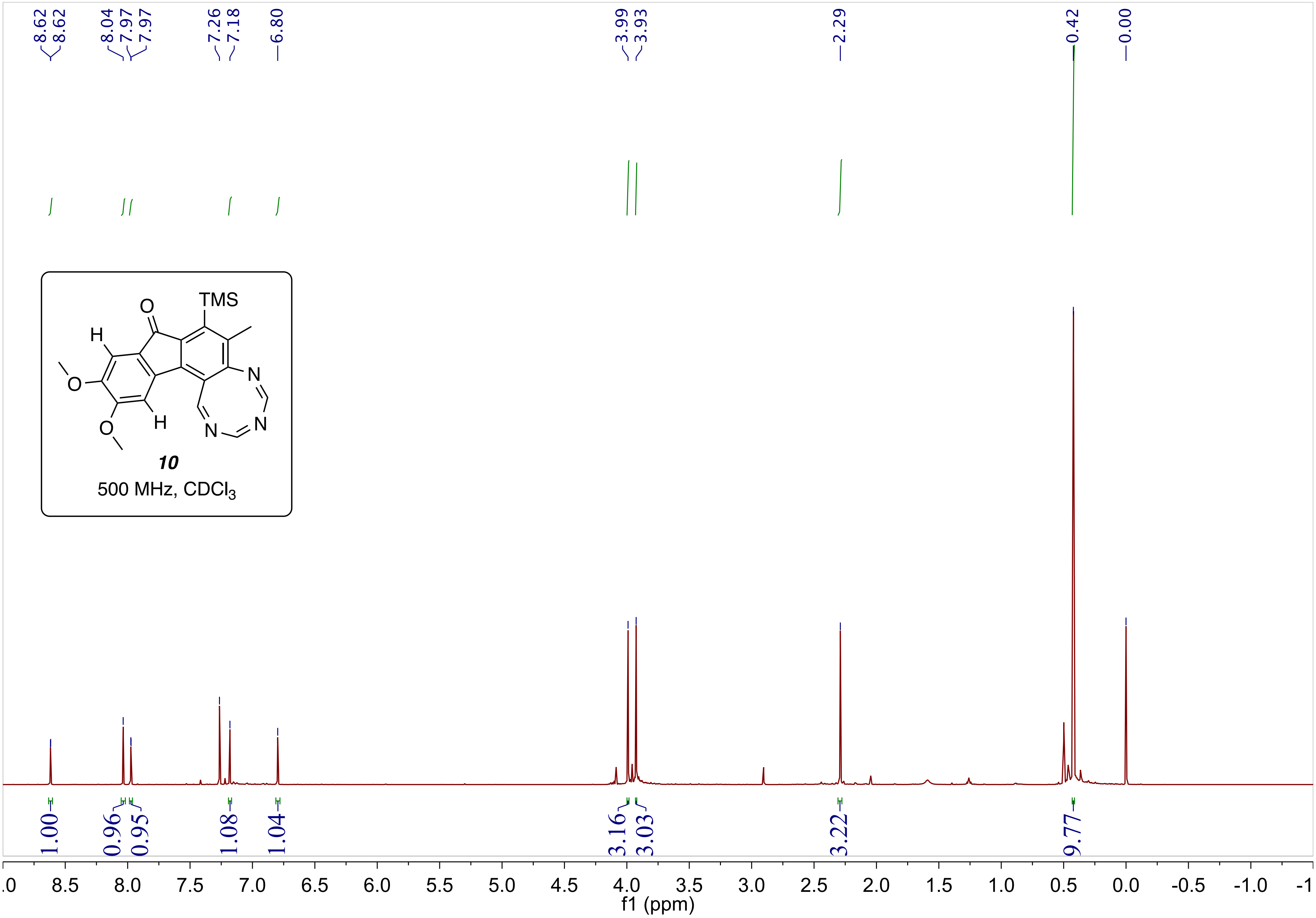
8.62
8.62
8.04
7.97
7.97
7.26
7.18
6.80
3.99
3.93
2.29
0.42
0.00
O
TMS
H
O
O
H
N
N
N
10
500 MHz, CDCl3
1.00
0.96
0.95
1.08
1.04
3.16
3.03
3.22
9.77
f1 (ppm)

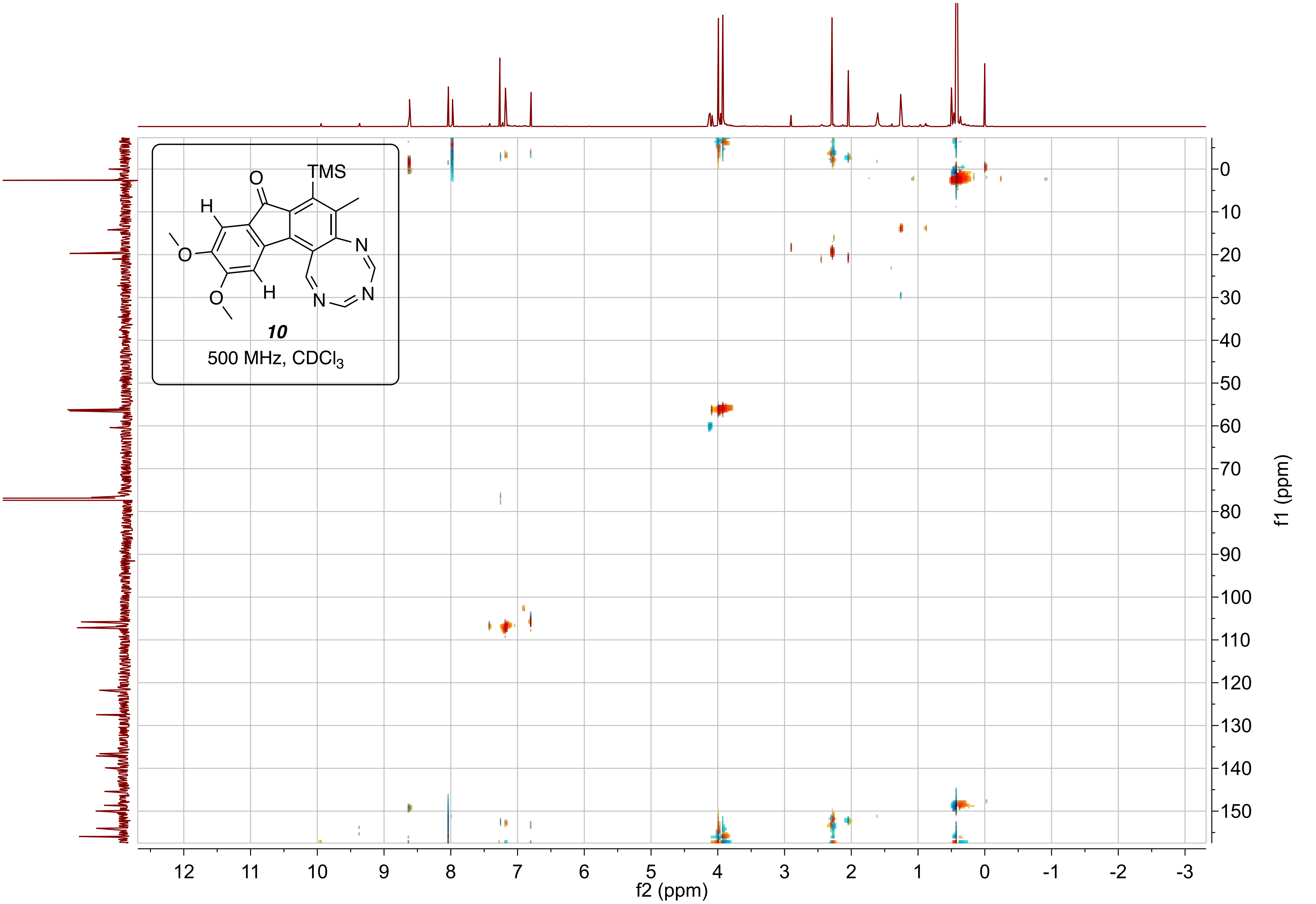

TMS
O
H
O
O
H
N
N
N
10
500 MHz, CDCl3
f2 (ppm)
f1 (ppm)

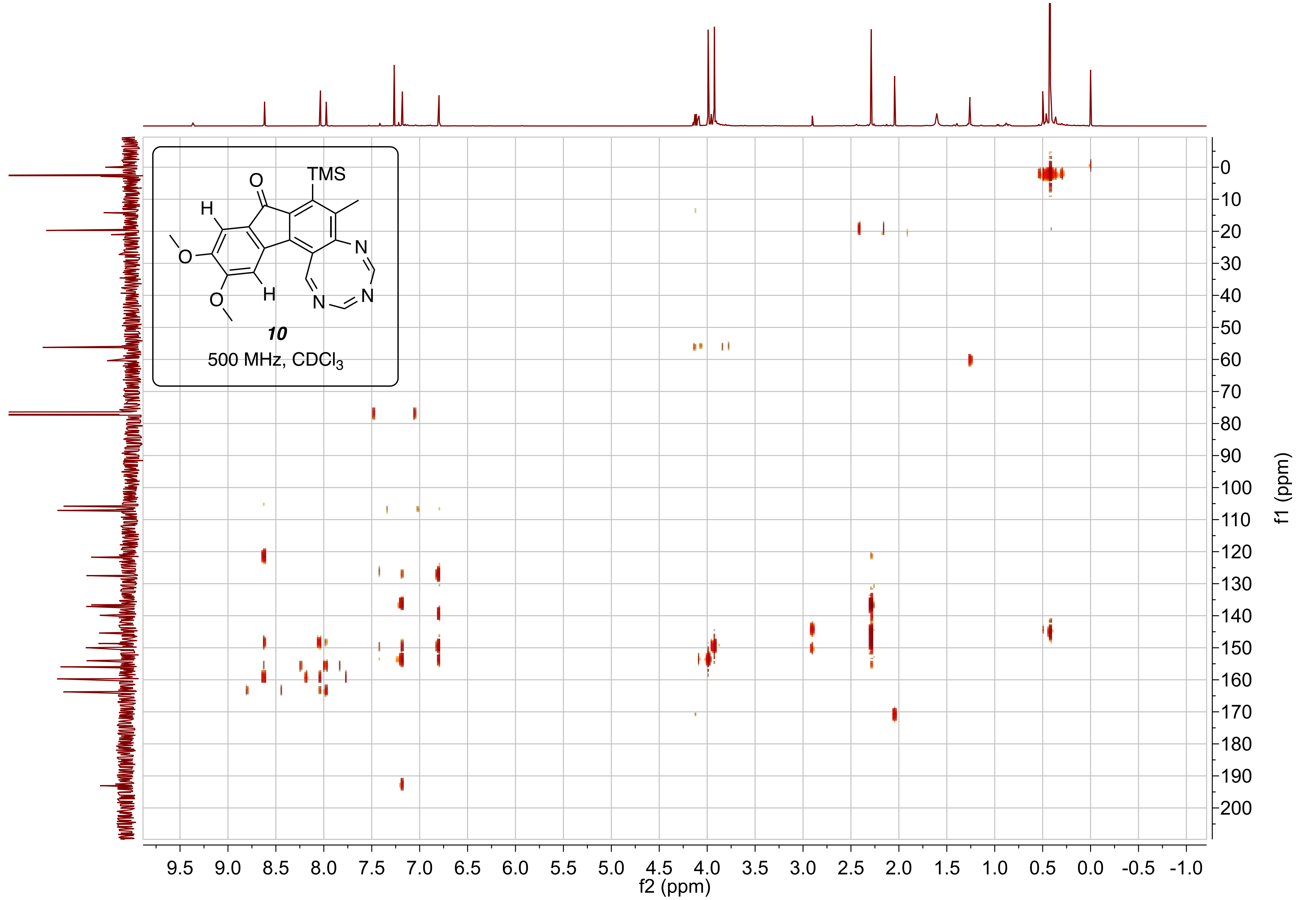

TMS
O
H
H
O
O
N
N
N
N
10
500 MHz, CDCl3
f2 (ppm)
f1 (ppm)

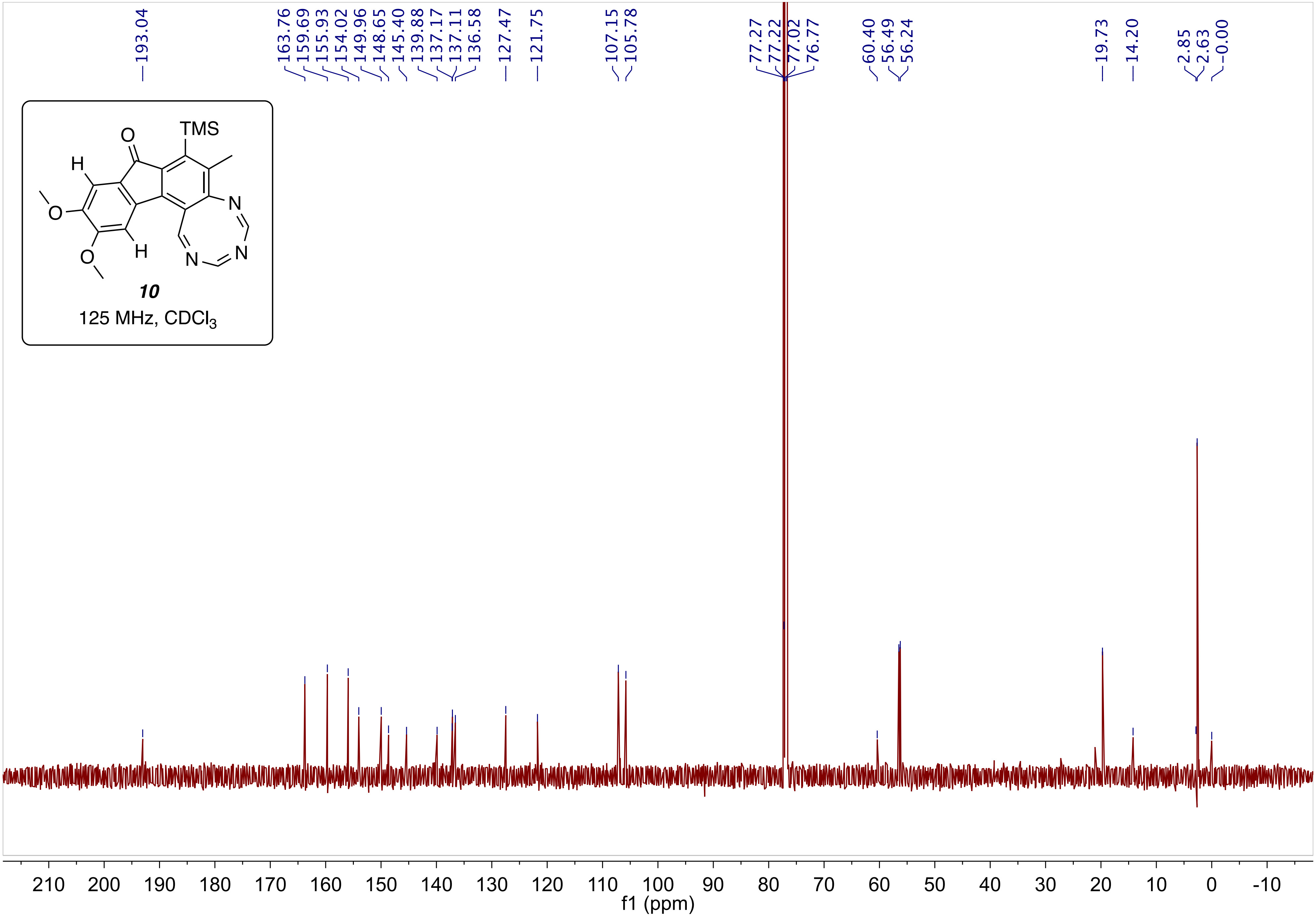
193.04
163.76
159.69
155.93
154.02
149.96
148.65
145.40
139.88
137.17
137.11
136.58
127.47
121.75
107.15
105.78
77.27
77.22
77.02
76.77
60.40
56.49
56.24
19.73
14.20
2.85
2.63
-0.00
TMS
O
H
N
N
N
H
O
O
10
125 MHz, CDCl3
210 200 190 180 170 160 150 140 130 120 110 100 90 80 70 60 50 40 30 20 10 0 -10
f1 (ppm)

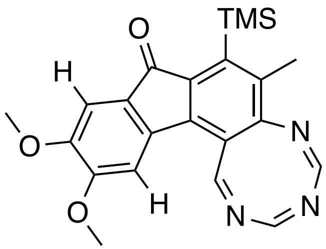

10

500 MHz, $CDCl_3$

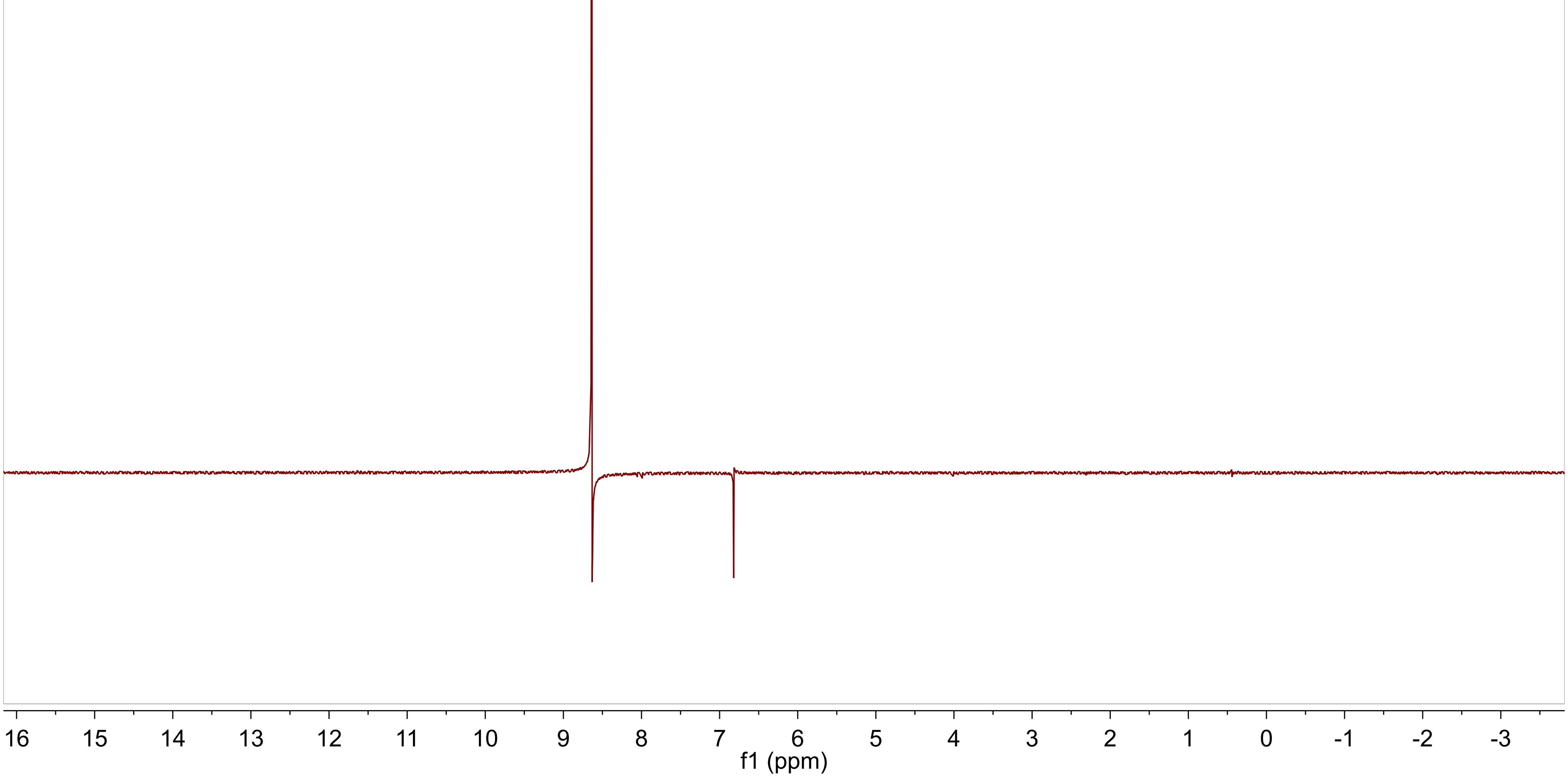

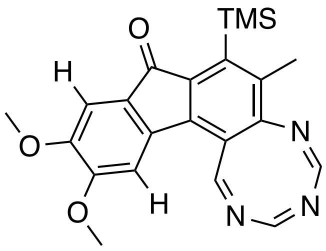
TMS
O
H
N
N
N
O
O
H
10
500 MHz, CDCl3

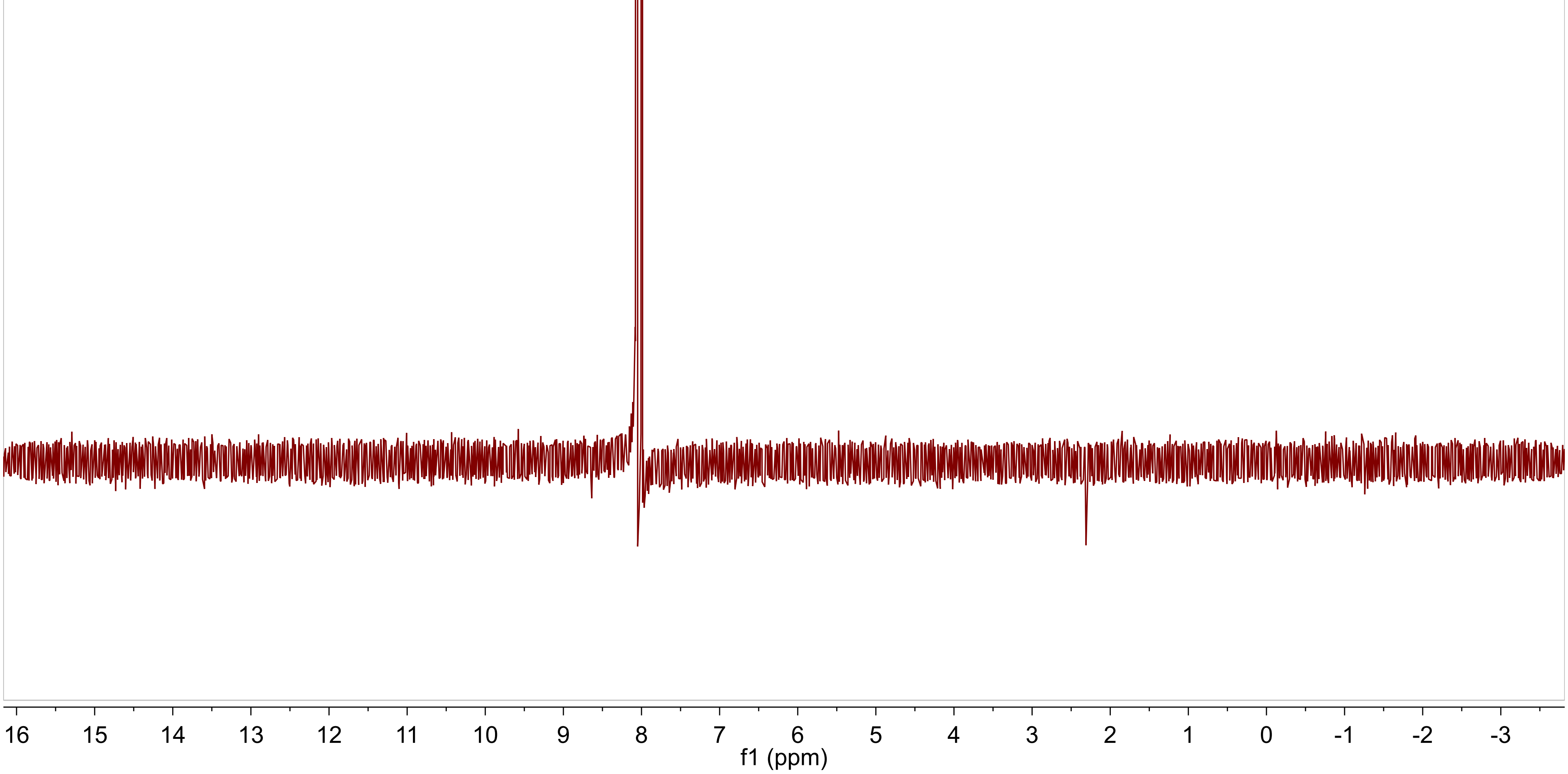
8.05
16 15 14 13 12 11 10 9 8 7 6 5 4 3 2 1 0 -1 -2 -3
f1 (ppm)

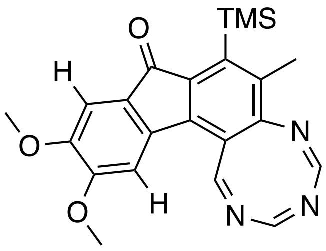

10

500 MHz, $CDCl_3$

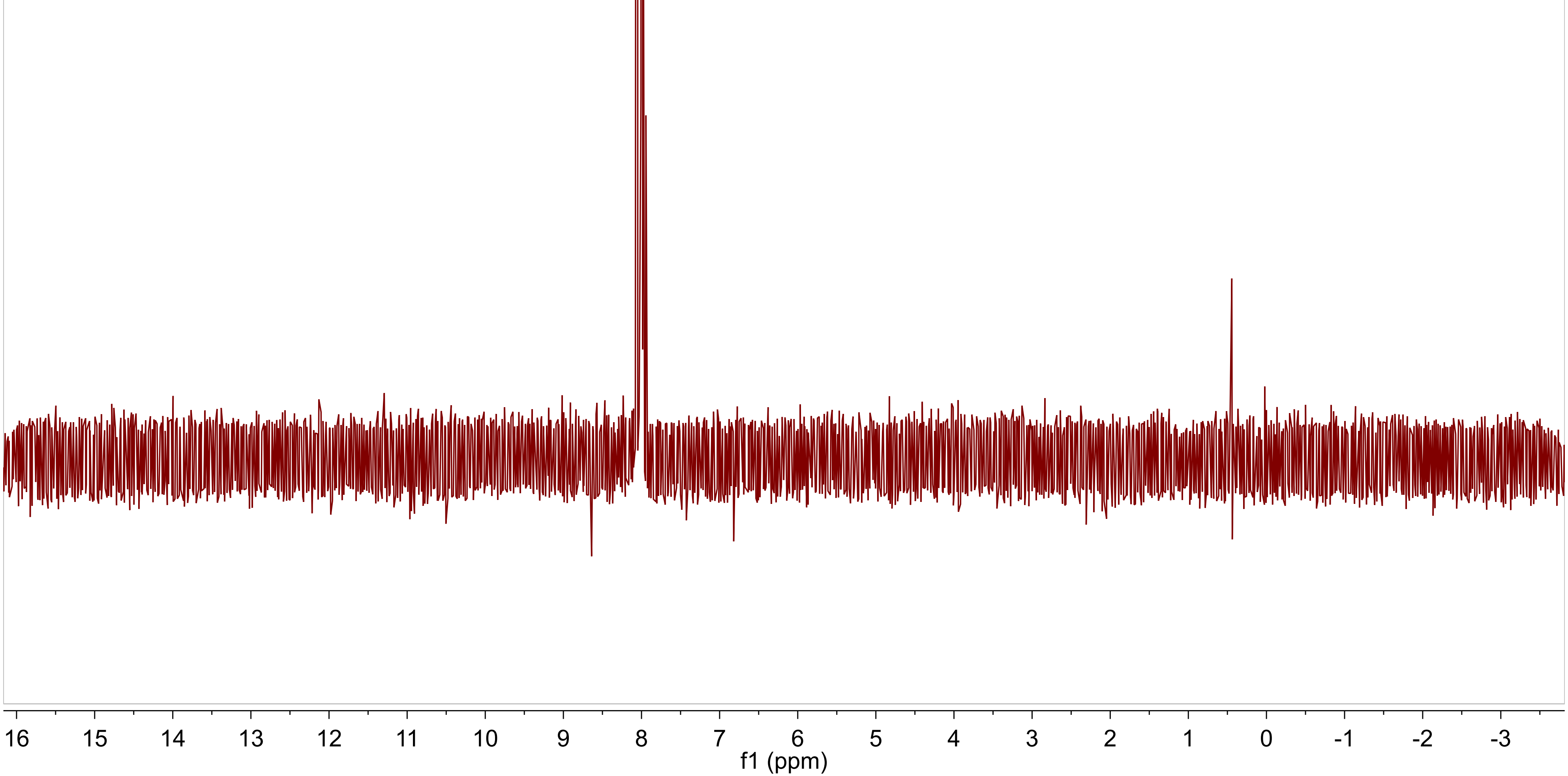

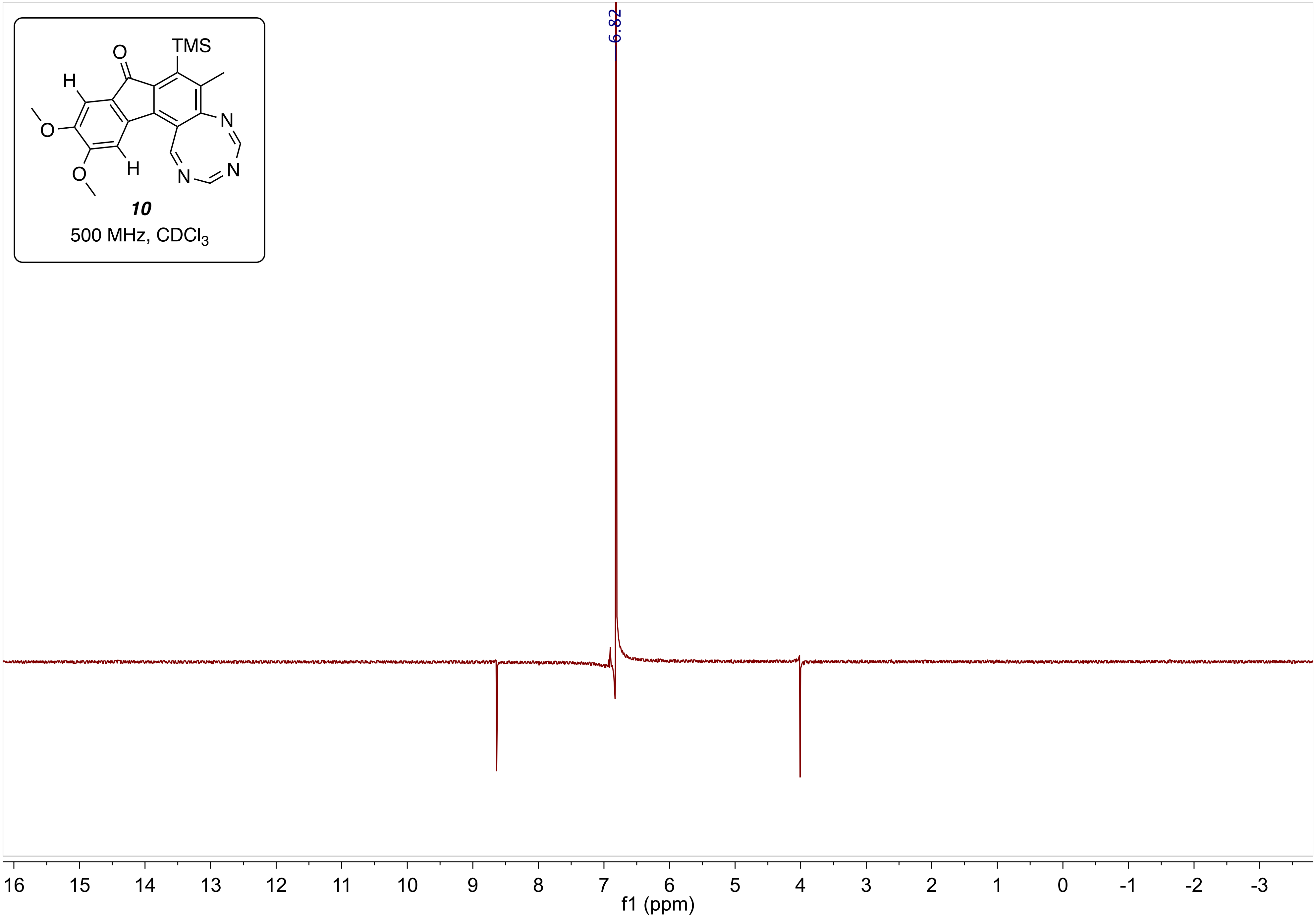
6.82
TMS
O
H
N
N
N
O
O
H
10
500 MHz, $CDCl_3$
16 15 14 13 12 11 10 9 8 7 6 5 4 3 2 1 0 -1 -2 -3
f1 (ppm)

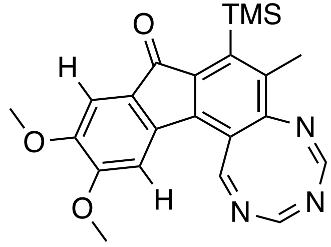

10

500 MHz, $CDCl_3$

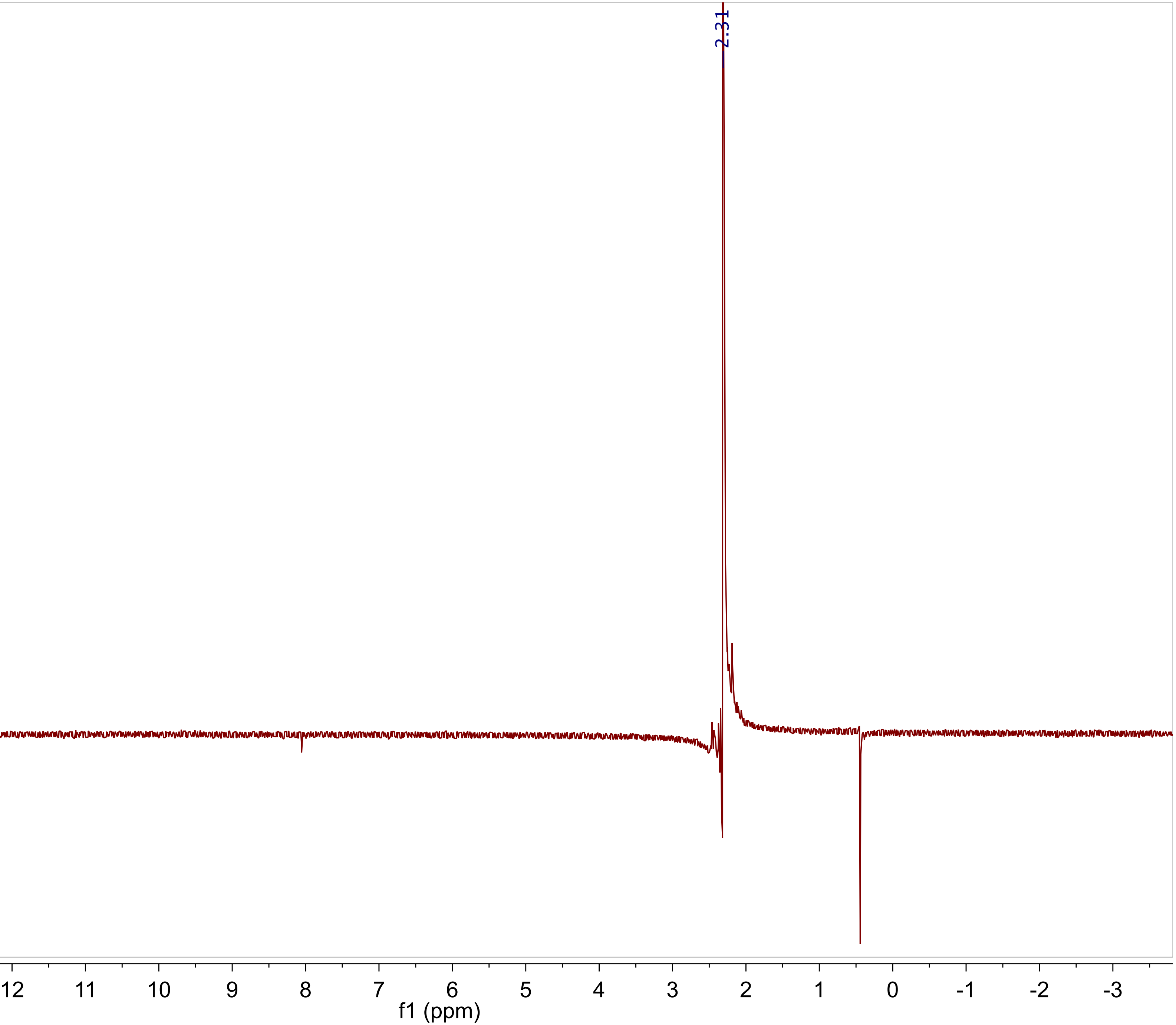

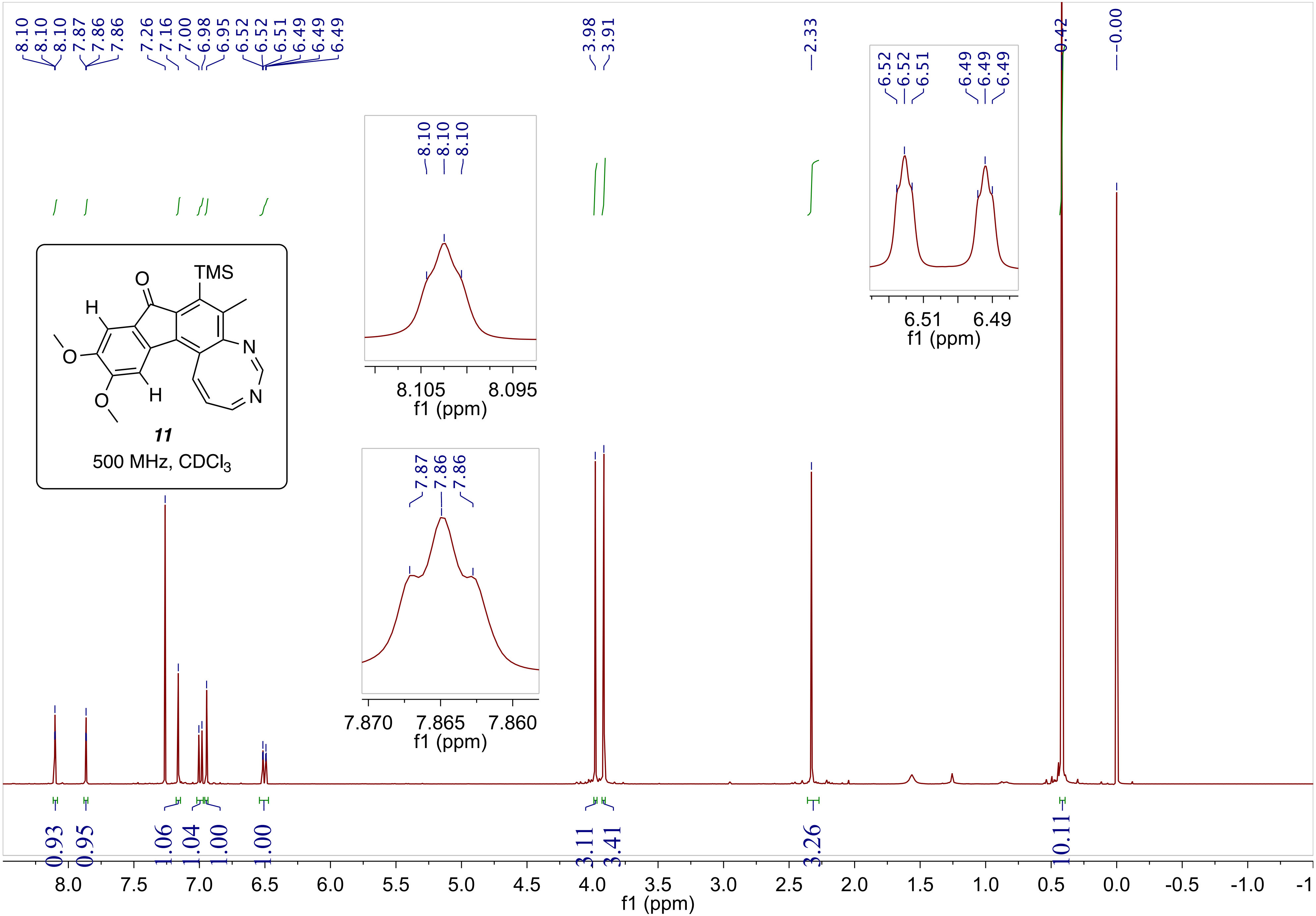

8.10 8.10 8.10 7.87 7.86 7.86 7.26 7.16 7.00 6.98 6.95 6.52 6.52 6.51 6.49 6.49 6.49
3.98 3.91
2.33
0.42
-0.00
TMS
O
H
N
N
H
O
O
11
500 MHz, CDCl3
8.105
8.095
7.870
7.865
7.860
6.51
6.49
f1 (ppm)
0.93 0.95 1.06 1.04 1.00 1.00 3.11 3.41 3.26 10.11
8.0 7.5 7.0 6.5 6.0 5.5 5.0 4.5 4.0 3.5 3.0 2.5 2.0 1.5 1.0 0.5 0.0 -0.5 -1.0 -1

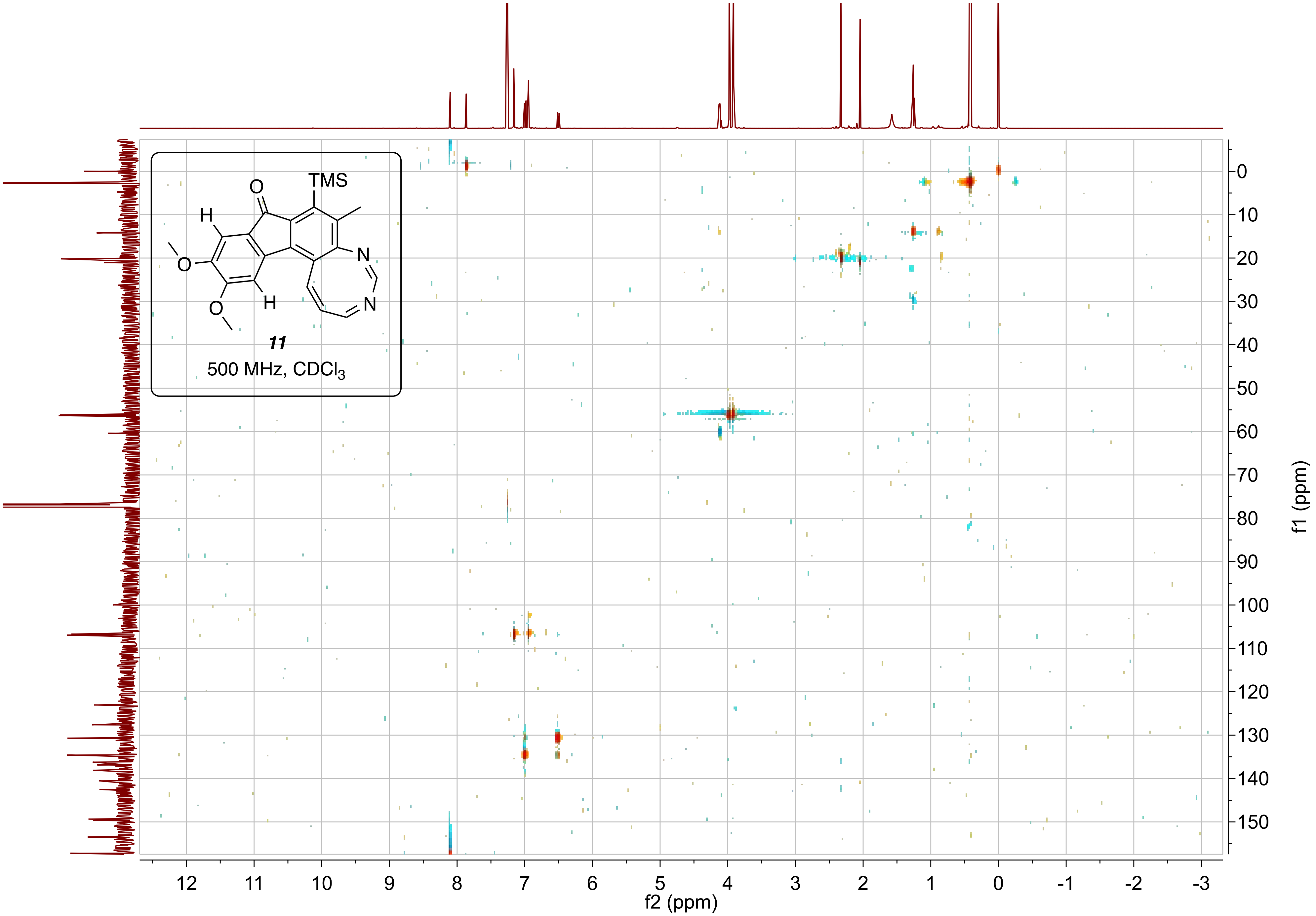

O
TMS
H
N
O
N
O
H
11
500 MHz, CDCl3
f2 (ppm)
f1 (ppm)

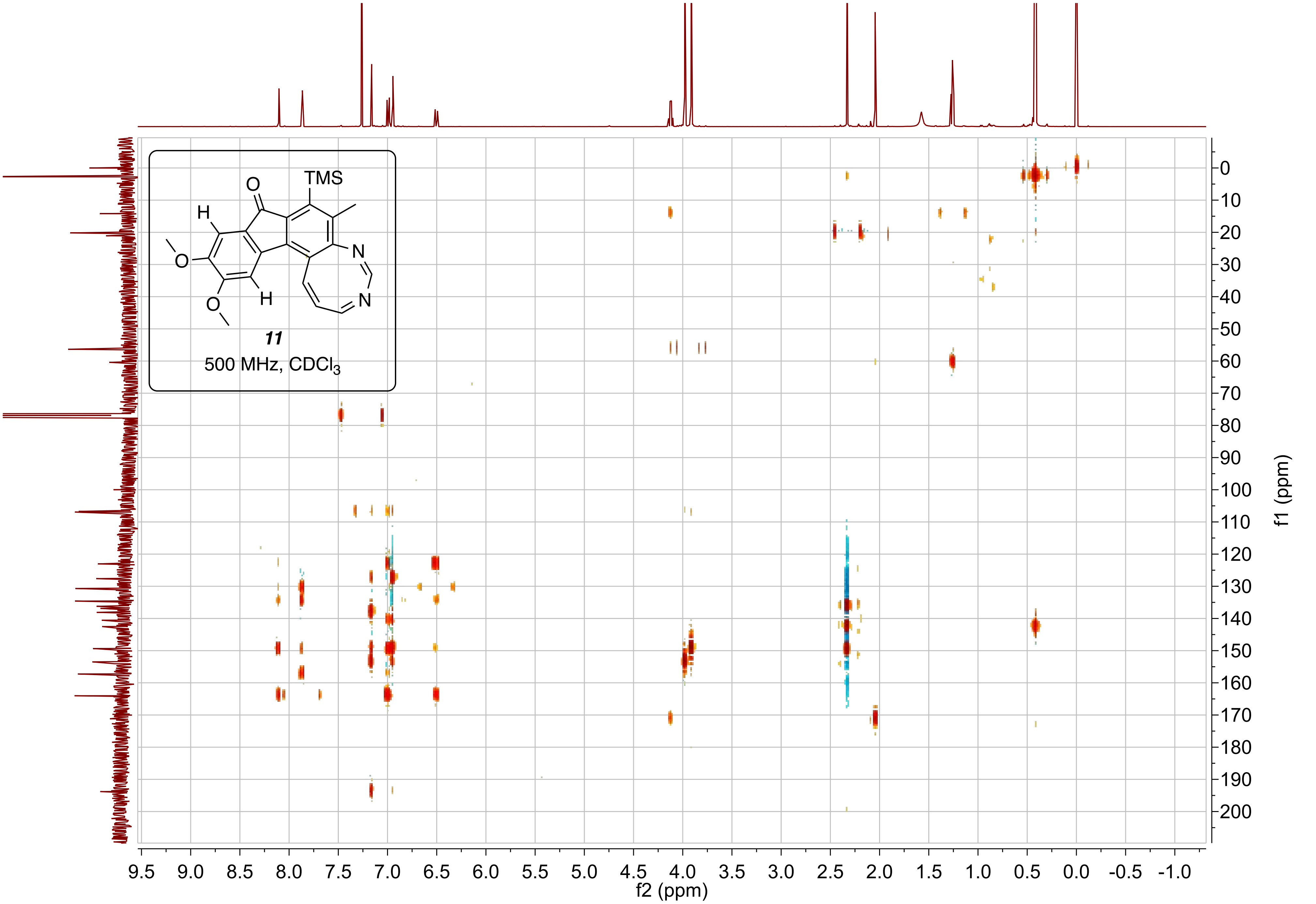

TMS
O
H
O
O
H
N
N
11
500 MHz, CDCl3
f2 (ppm)
f1 (ppm)

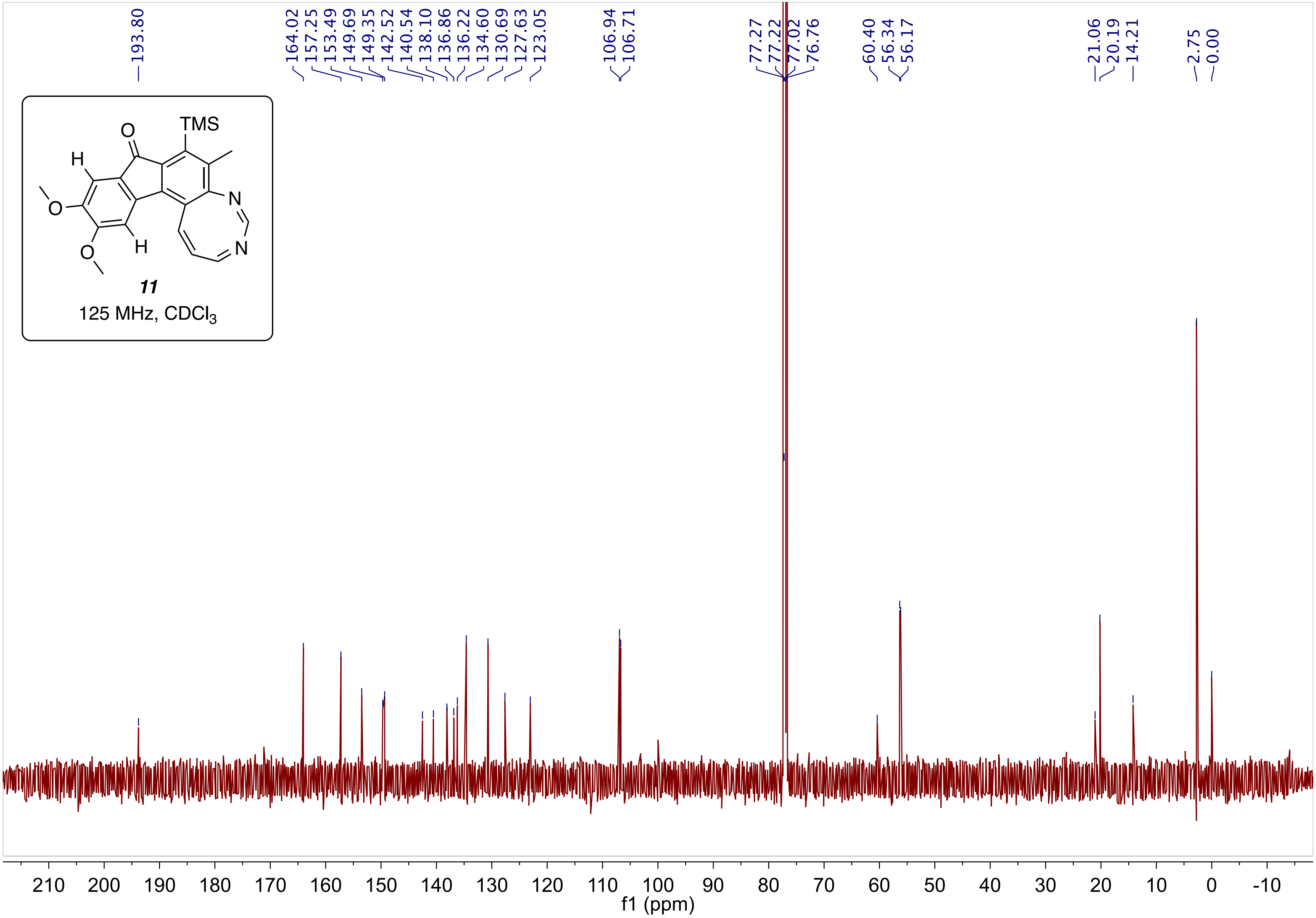
193.80
164.02
157.25
153.49
149.69
149.35
142.52
140.54
138.10
136.86
136.22
134.60
130.69
127.63
123.05
106.94
106.71
77.27
77.22
77.02
76.76
60.40
56.34
56.17
21.06
20.19
14.21
2.75
0.00
TMS
O
H
N
N
H
O
O
11
125 MHz, CDCl3
210 200 190 180 170 160 150 140 130 120 110 100 90 80 70 60 50 40 30 20 10 0 -10
f1 (ppm)

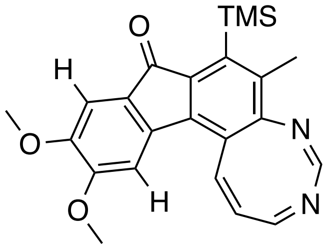

11
500 MHz, $CDCl_3$

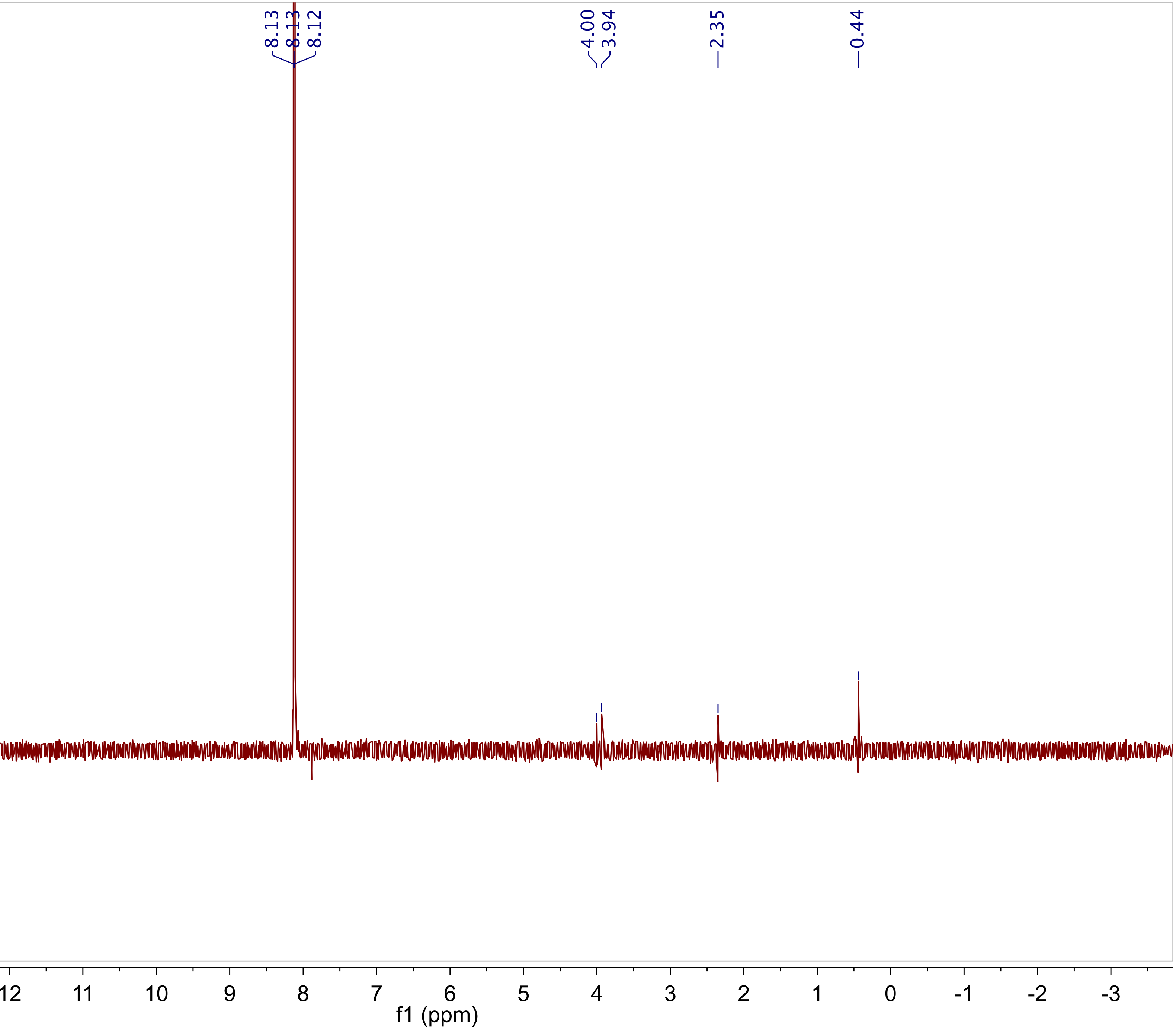

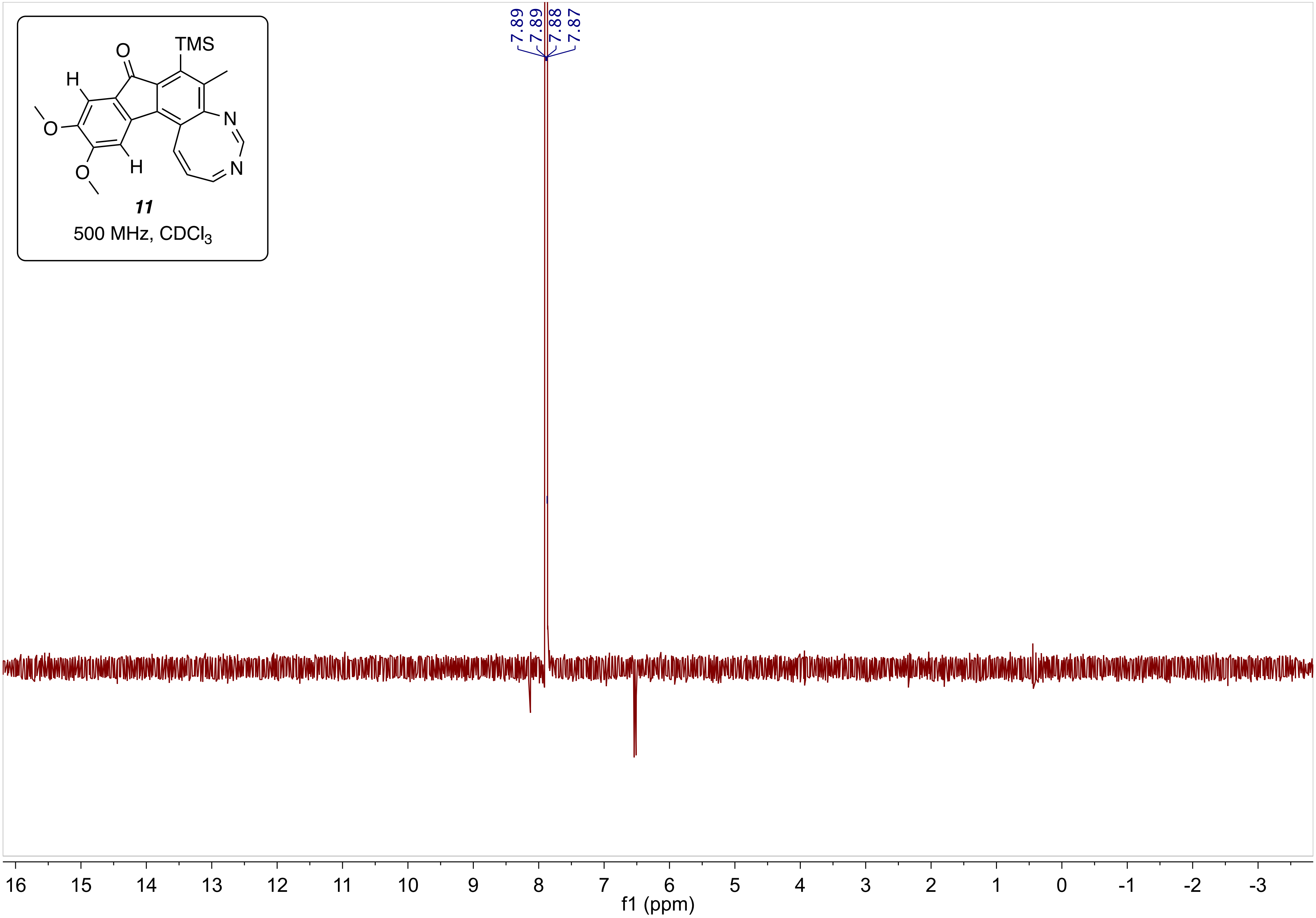
O
TMS
H
O
N
N
O
H
11
500 MHz, CDCl3
7.89
7.89
7.88
7.87
16
15
14
13
12
11
10
9
8
7
6
5
4
3
2
1
0
-1
-2
-3
f1 (ppm)

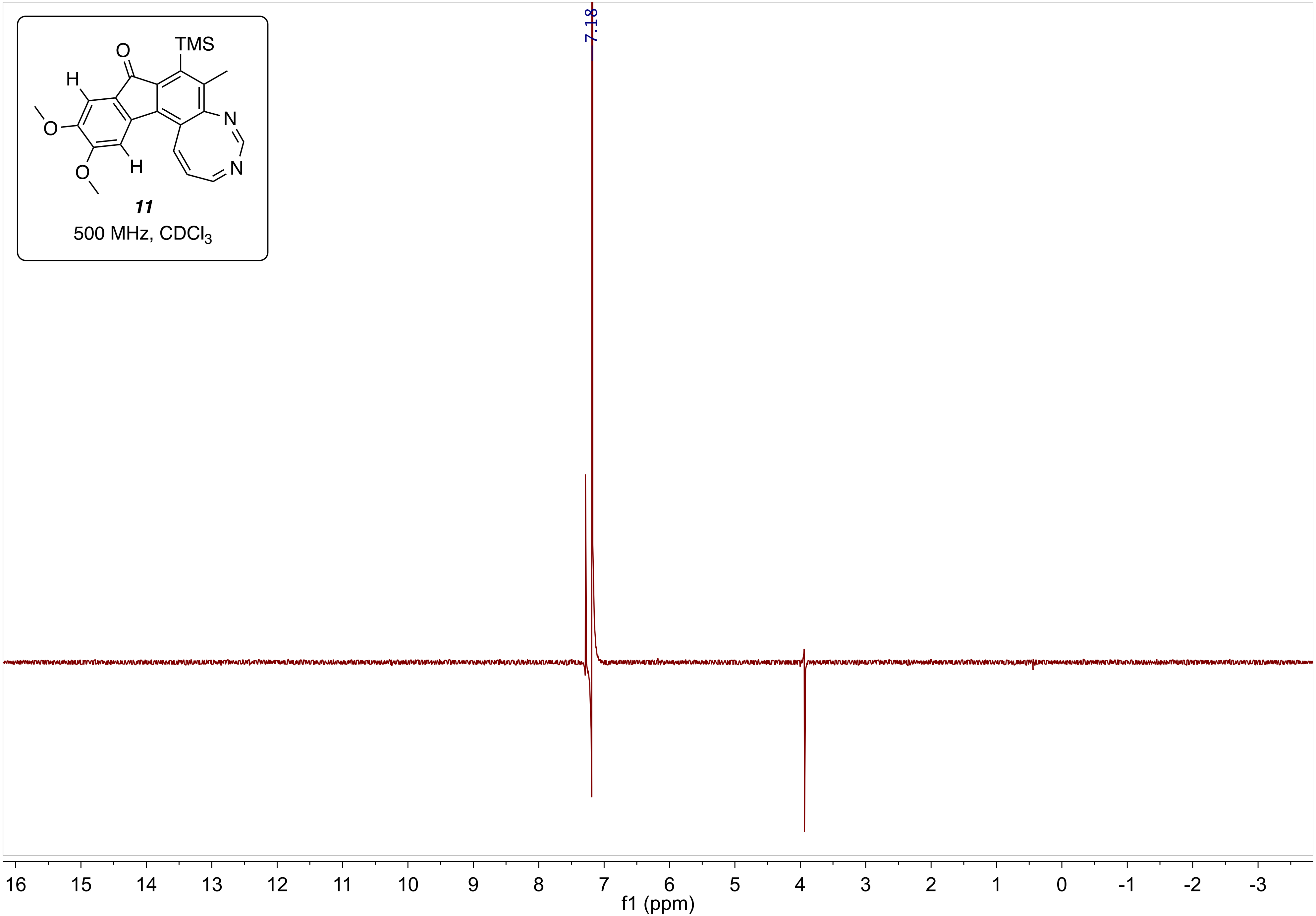
TMS
O
H
O
O
H
N
N
11
500 MHz, CDCl3
7.18
16 15 14 13 12 11 10 9 8 7 6 5 4 3 2 1 0 -1 -2 -3
f1 (ppm)

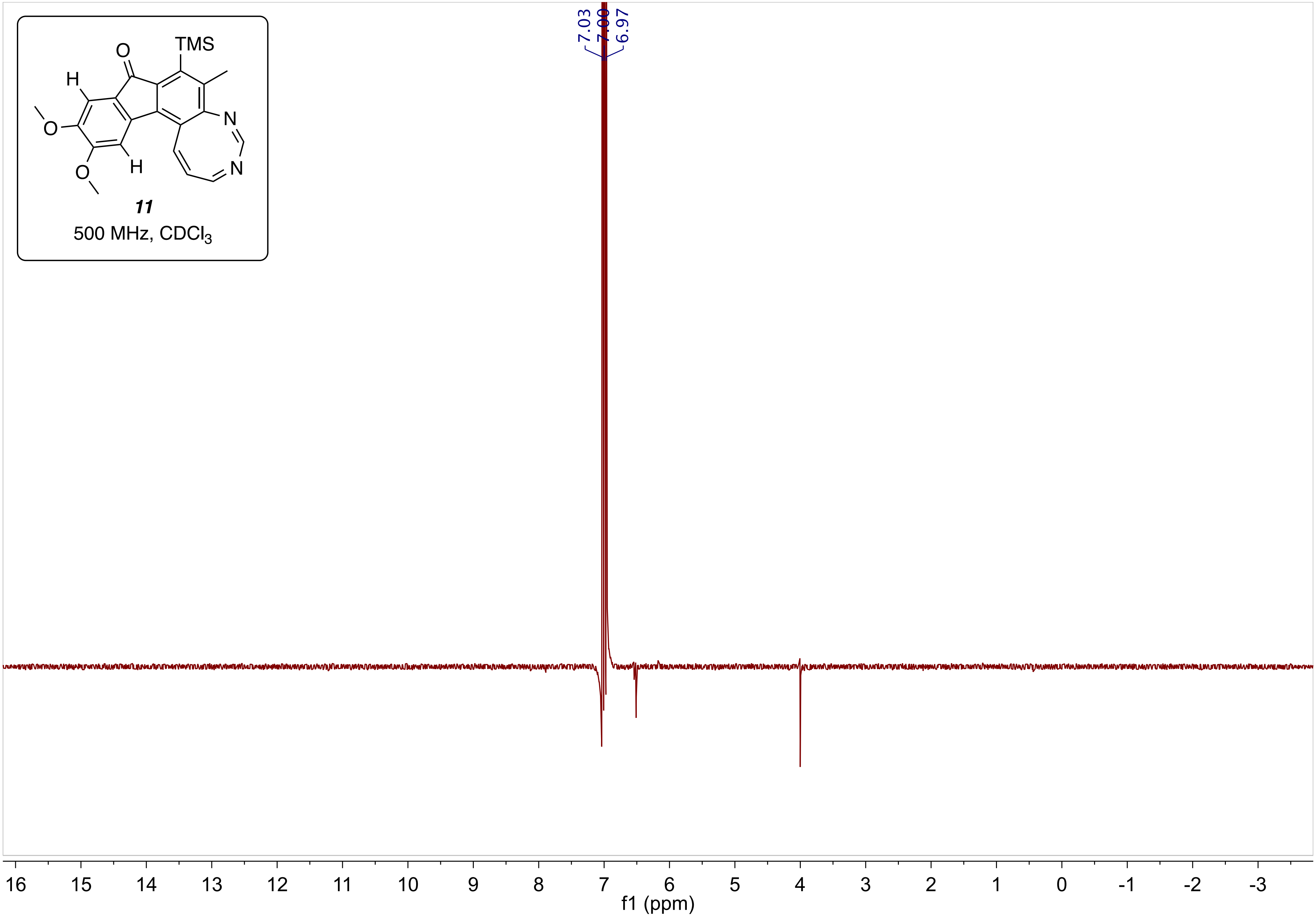

11

500 MHz, $CDCl_3$

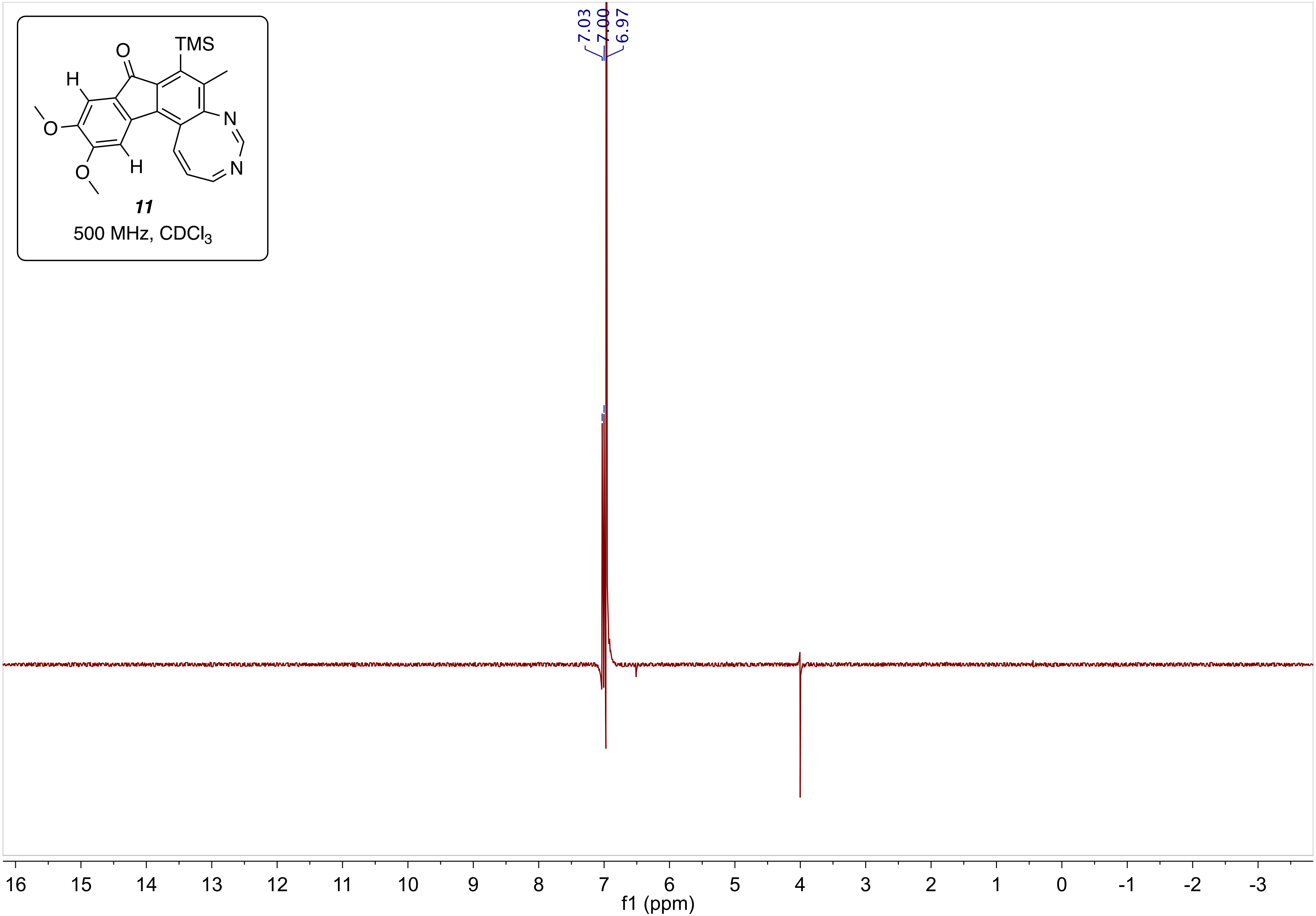
TMS
O
H
O
O
H
N
N
11
500 MHz, CDCl3
7.03
7.00
6.97
16
15
14
13
12
11
10
9
8
7
6
5
4
3
2
1
0
-1
-2
-3
f1 (ppm)

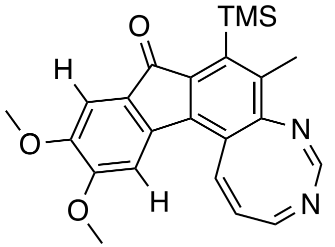

11

500 MHz, $CDCl_3$

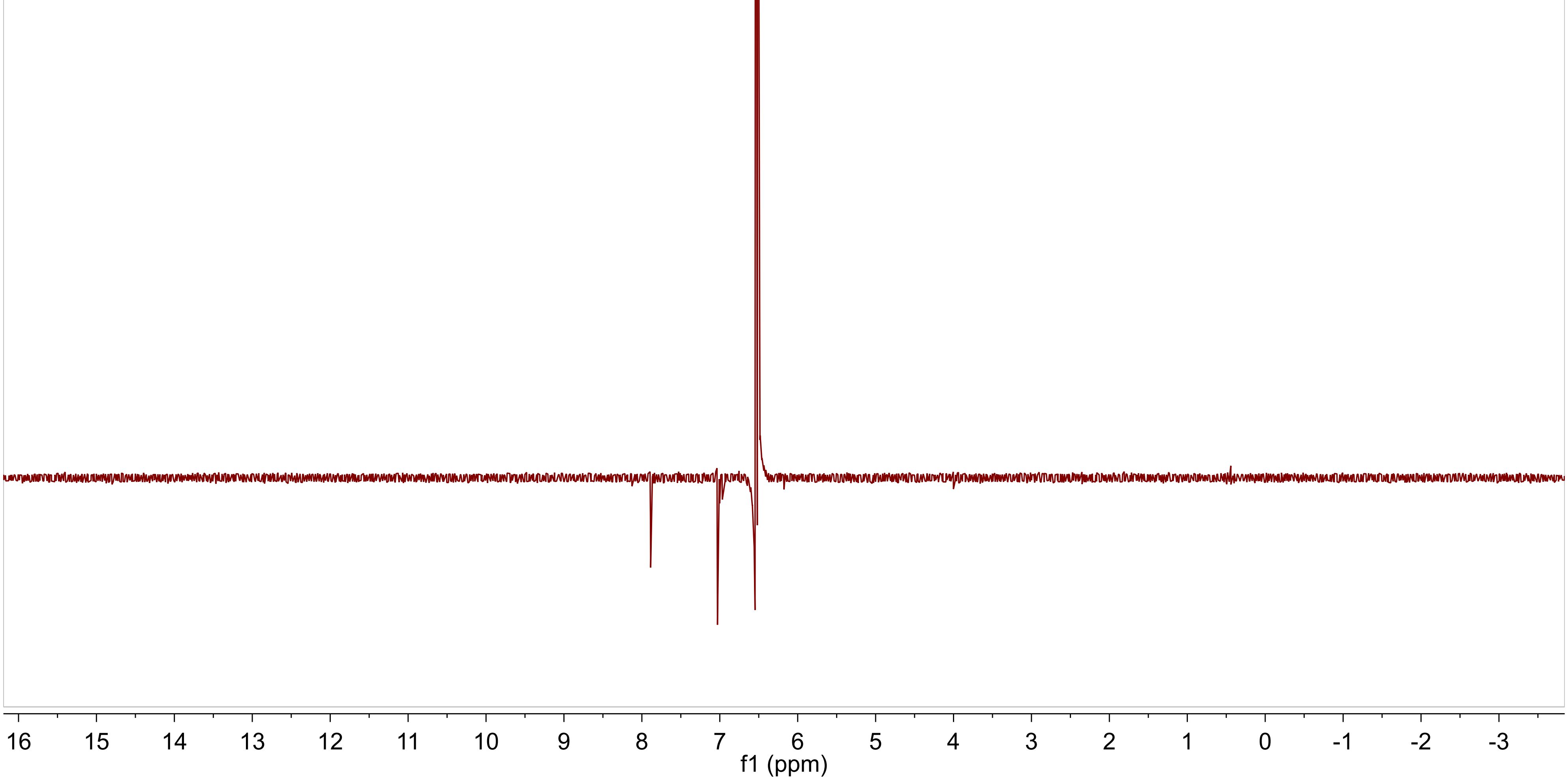

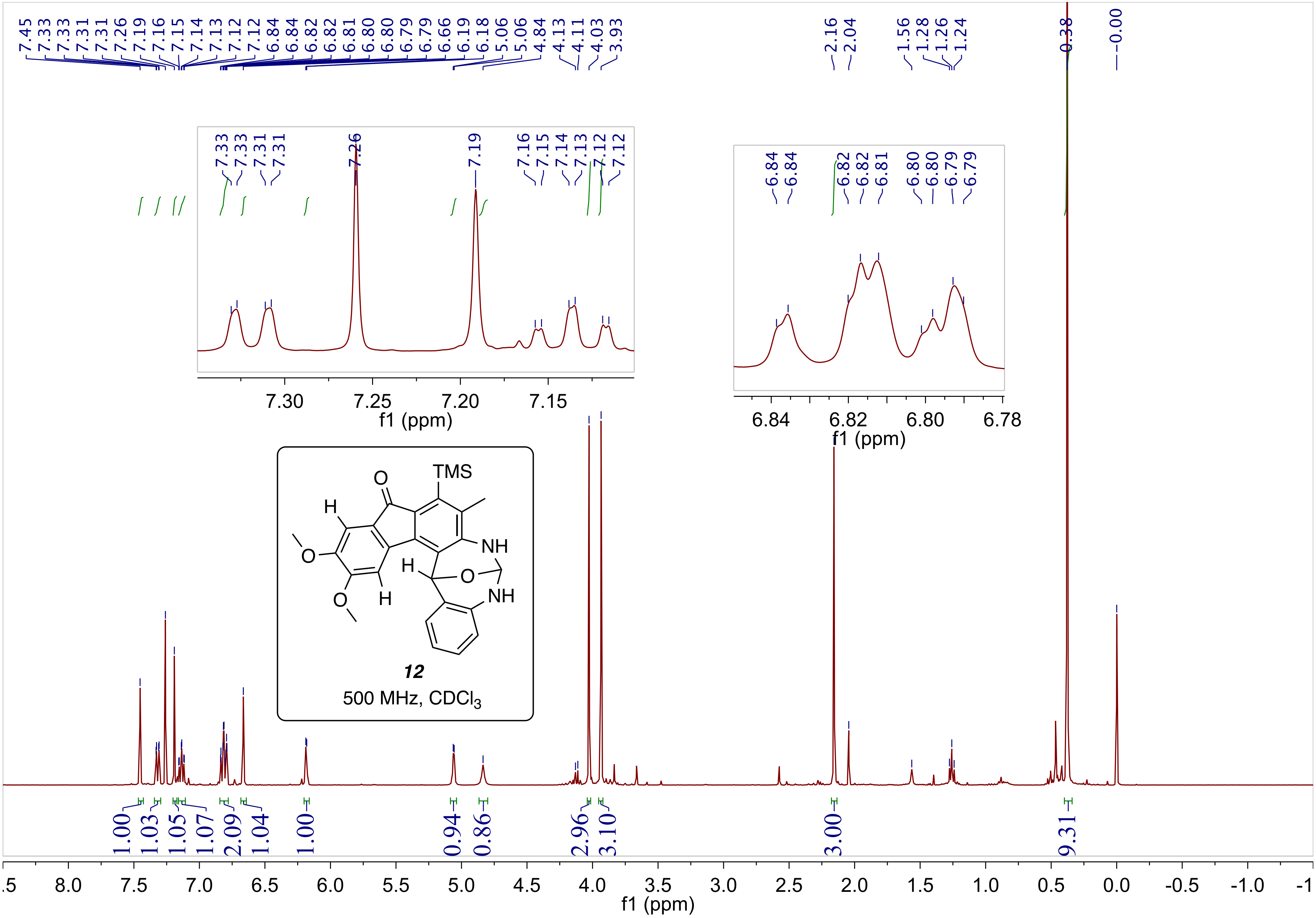
7.45 7.33 7.33 7.31 7.31 7.26 7.19 7.16 7.15 7.14 7.13 7.12 7.12 6.84 6.84 6.82 6.82 6.81 6.80 6.80 6.79 6.79 6.66 6.19 6.18 5.06 5.06 4.84 4.13 4.11 4.03 3.93
2.16 2.04 1.56 1.28 1.26 1.24 0.38 -0.00
1.00 1.03 1.05 1.07 2.09 1.04 1.00 0.94 0.86 2.96 3.10 3.00 9.31
f1 (ppm)
TMS
O
H
NH
O
H
NH
12
500 MHz, $CDCl_3$

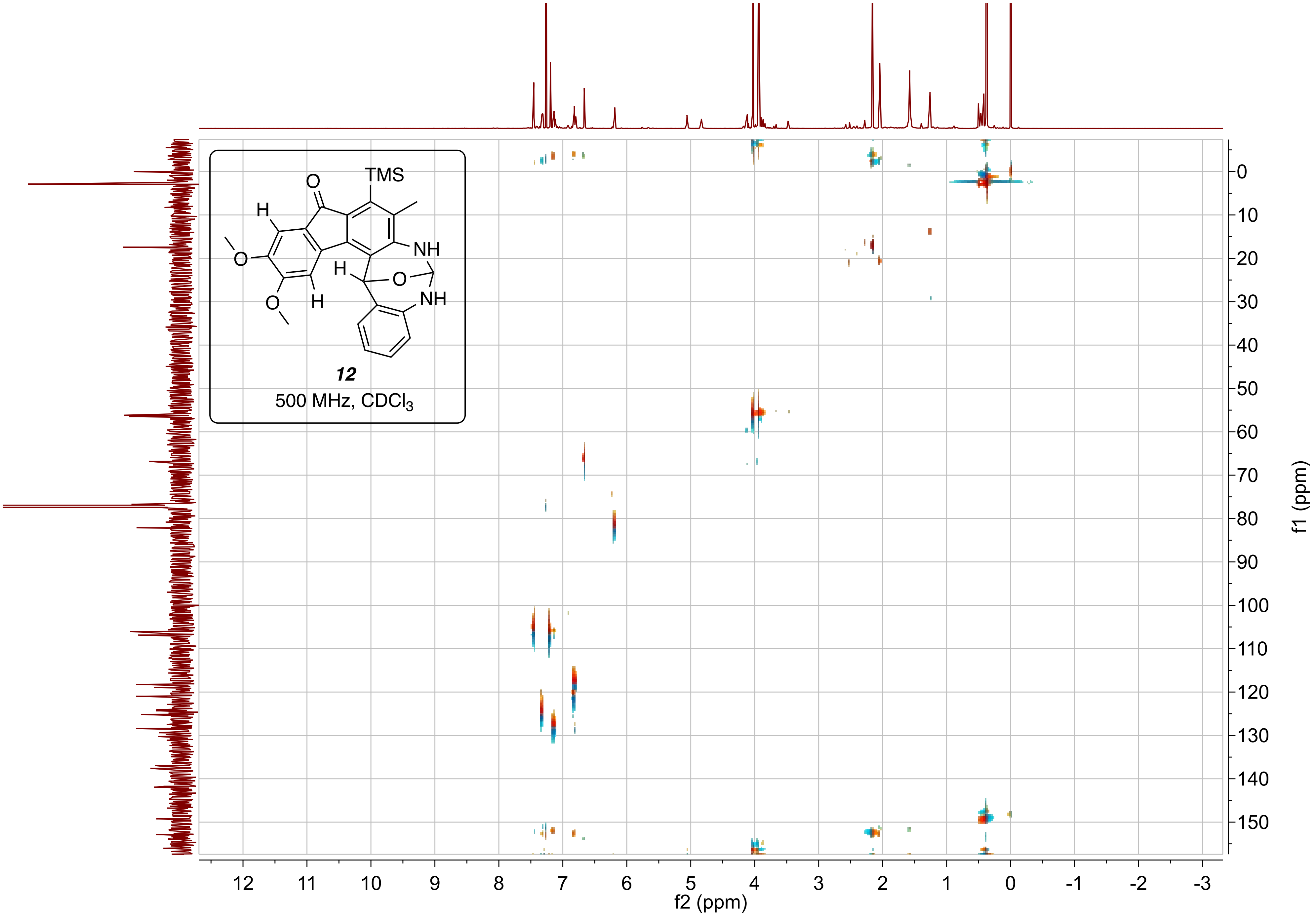
O
TMS
H
O
NH
H
O
O
H
NH
12
500 MHz, CDCl3
f2 (ppm)
f1 (ppm)

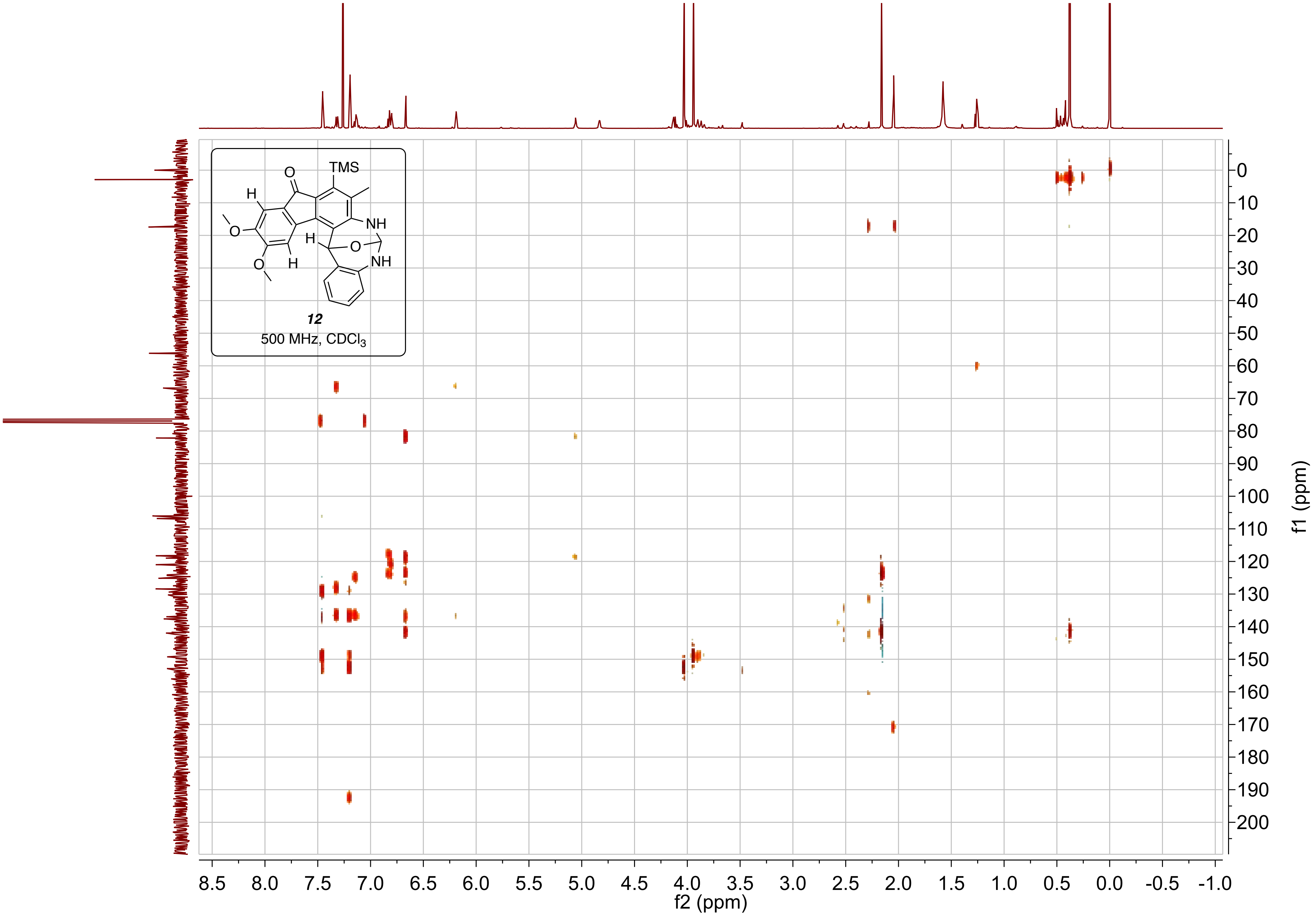
TMS
O
H
NH
O
H
O
NH
O
H
12
500 MHz, CDCl3
f2 (ppm)
f1 (ppm)

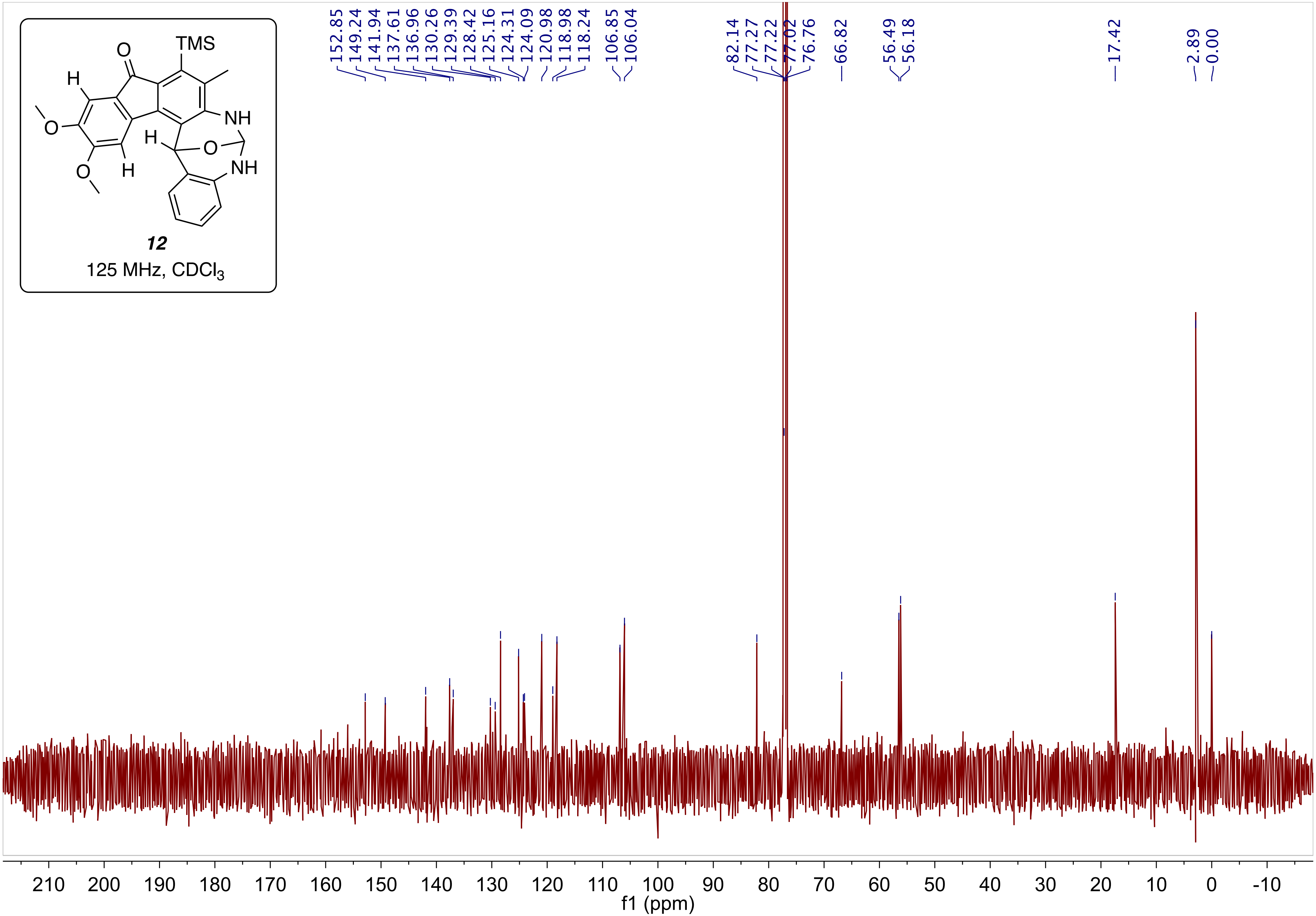
152.85
149.24
141.94
137.61
136.96
130.26
129.39
128.42
125.16
124.31
124.09
120.98
118.98
118.24
106.85
106.04
82.14
77.27
77.22
77.02
76.76
66.82
56.49
56.18
17.42
2.89
0.00
TMS
O
H
NH
O
NH
H
O
O
H
12
125 MHz, CDCl3
210 200 190 180 170 160 150 140 130 120 110 100 90 80 70 60 50 40 30 20 10 0 -10
f1 (ppm)

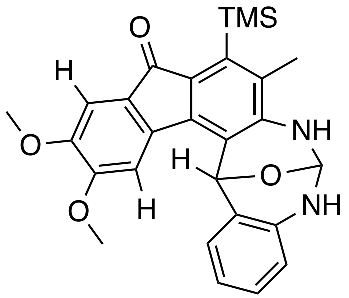

12

500 MHz, $CDCl_3$

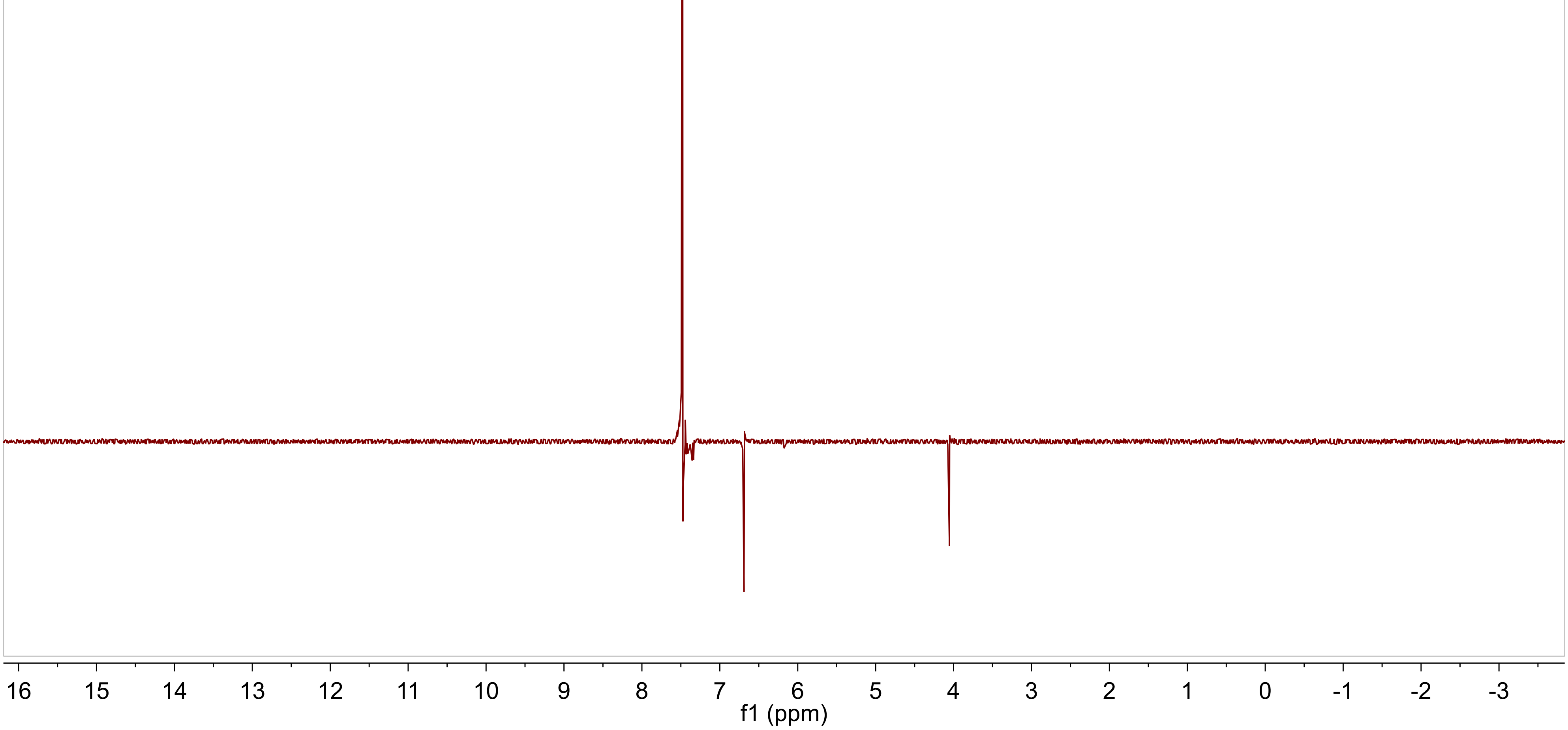

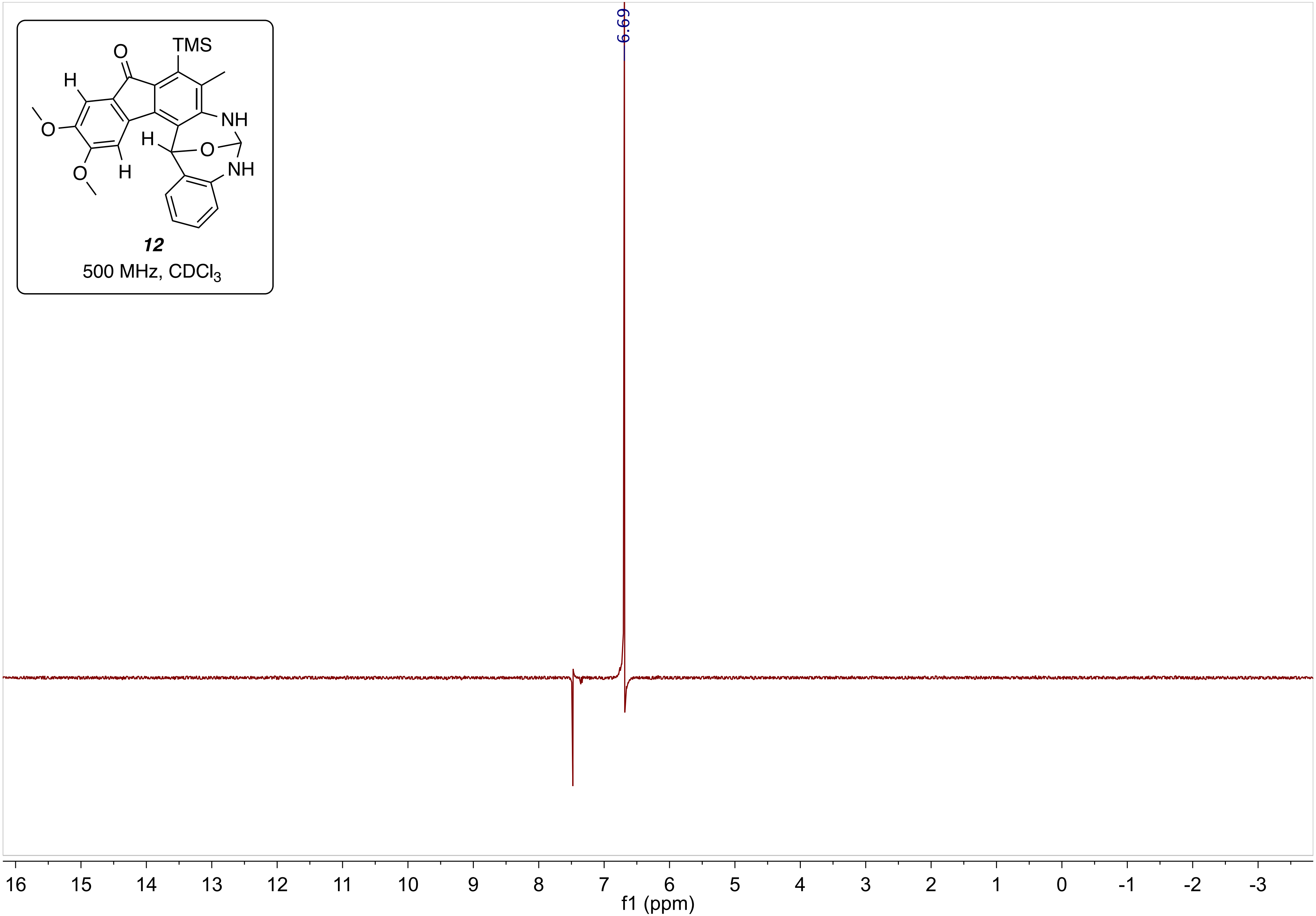
TMS
O
H
NH
O
NH
12
500 MHz, $CDCl_3$
6.69
16 15 14 13 12 11 10 9 8 7 6 5 4 3 2 1 0 -1 -2 -3
f1 (ppm)

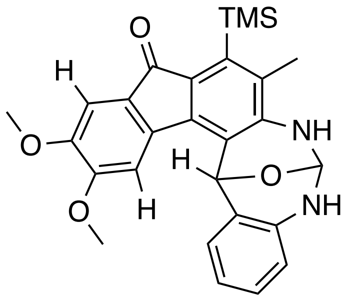

12

500 MHz, $CDCl_3$

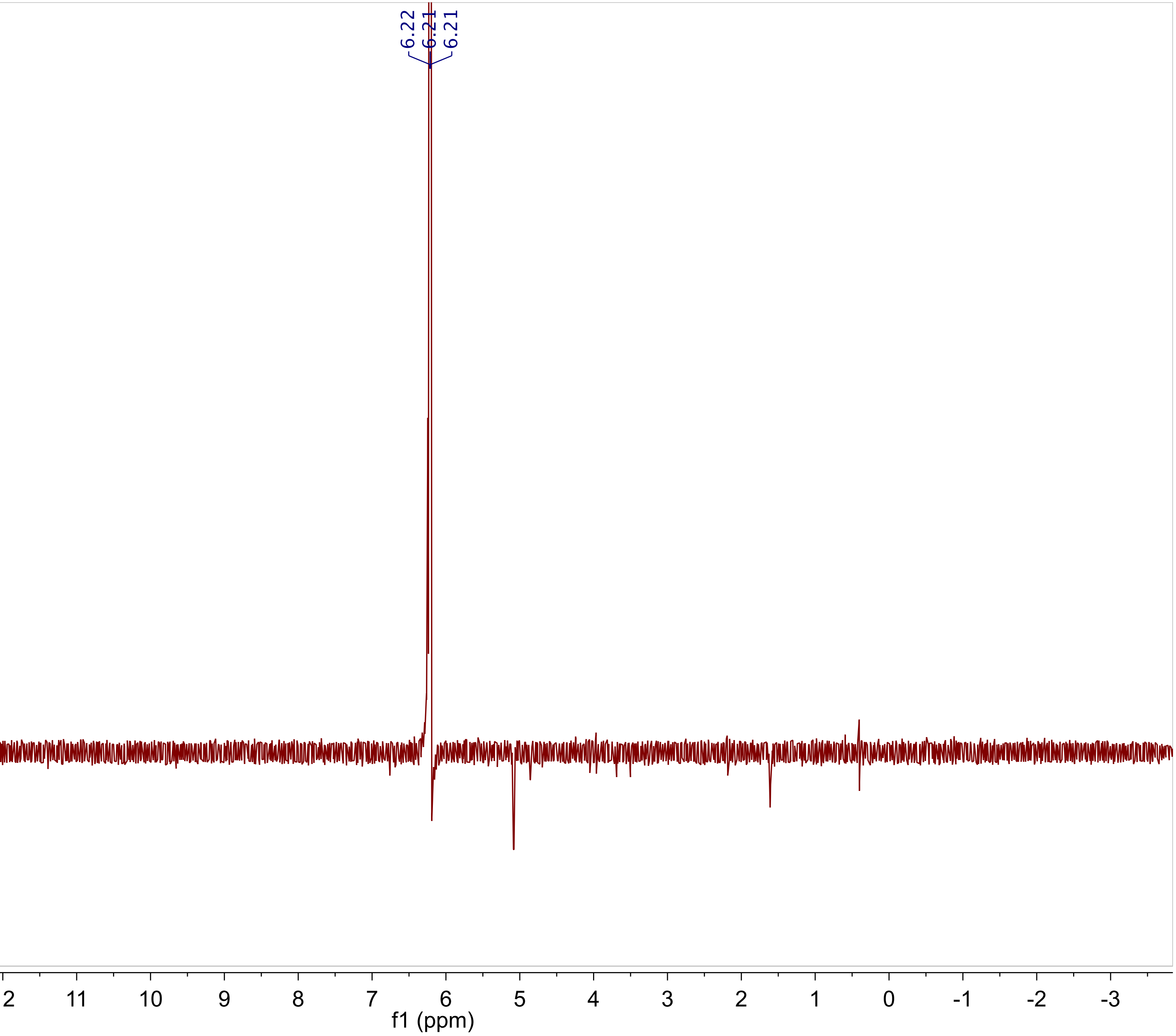

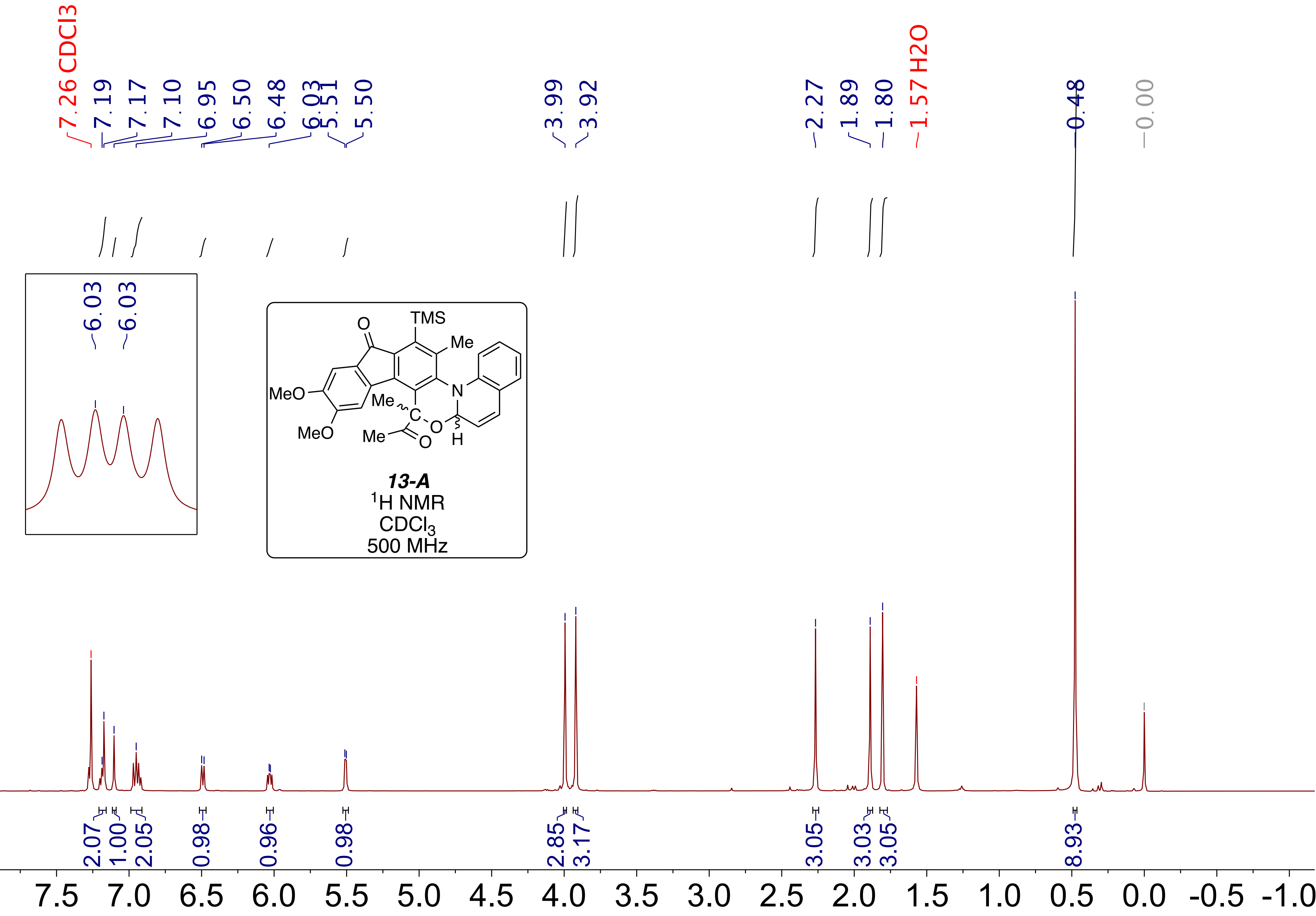
7.26 CDCl3
7.19
7.17
7.10
6.95
6.50
6.48
6.03
5.51
5.50
3.99
3.92
2.27
1.89
1.80
1.57 H2O
0.48
0.00
6.03
6.03
TMS
O
Me
MeO
MeO
Me
C
O
N
H
Me
O
13-A
1H NMR
CDCl3
500 MHz
2.07
1.00
2.05
0.98
0.96
0.98
2.85
3.17
3.05
3.03
3.05
8.93
7.5 7.0 6.5 6.0 5.5 5.0 4.5 4.0 3.5 3.0 2.5 2.0 1.5 1.0 0.5 0.0 -0.5 -1.0

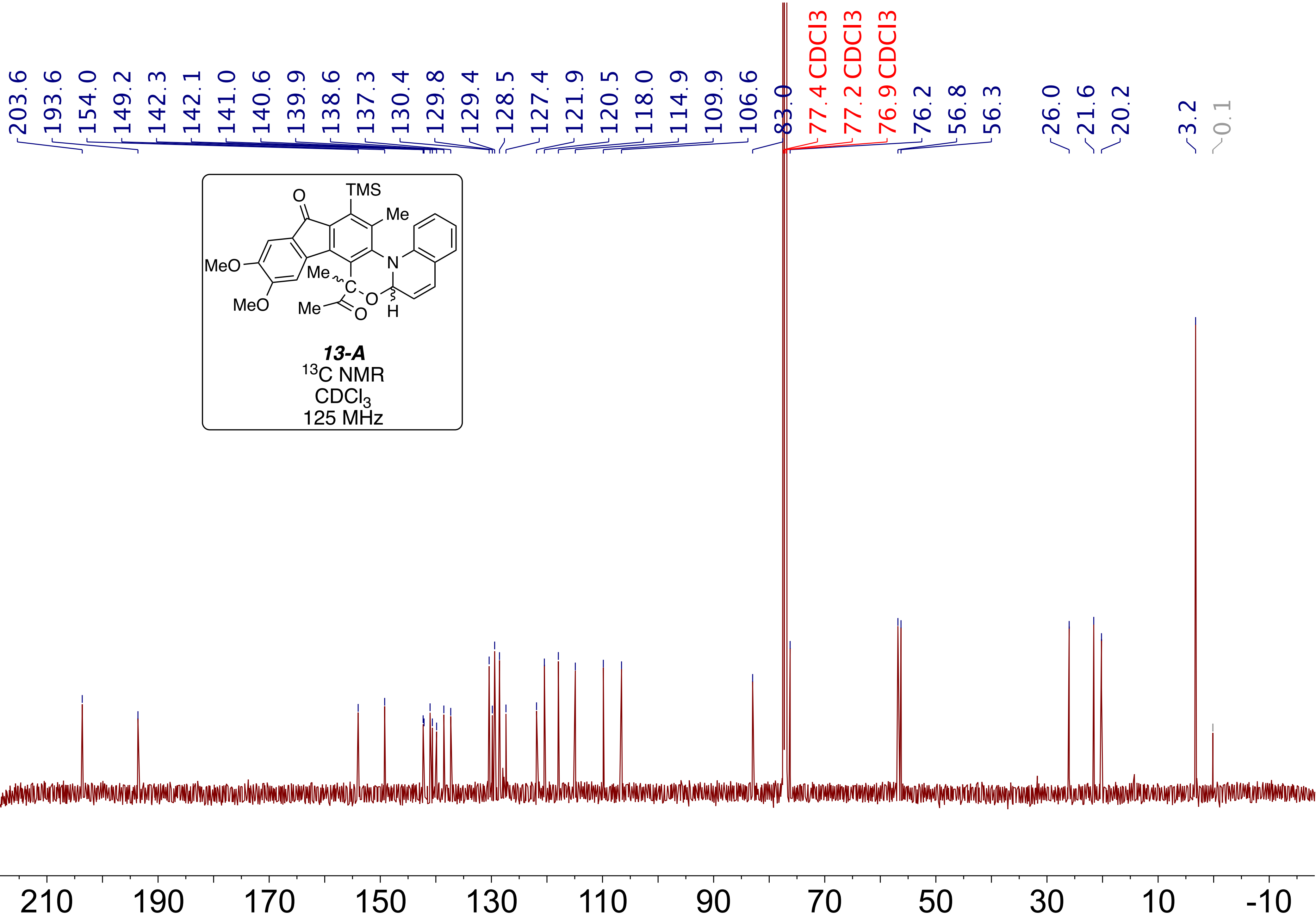
203.6
193.6
154.0
149.2
142.3
142.1
141.0
140.6
139.9
138.6
137.3
130.4
129.8
129.4
128.5
127.4
121.9
120.5
118.0
114.9
109.9
106.6
83.0
77.4 CDCl3
77.2 CDCl3
76.9 CDCl3
76.2
56.8
56.3
26.0
21.6
20.2
3.2
-0.1
O
TMS
Me
MeO
MeO
Me
N
C
O
H
Me
O
13-A
13C NMR
CDCl3
125 MHz
210 190 170 150 130 110 90 70 50 30 10 -10

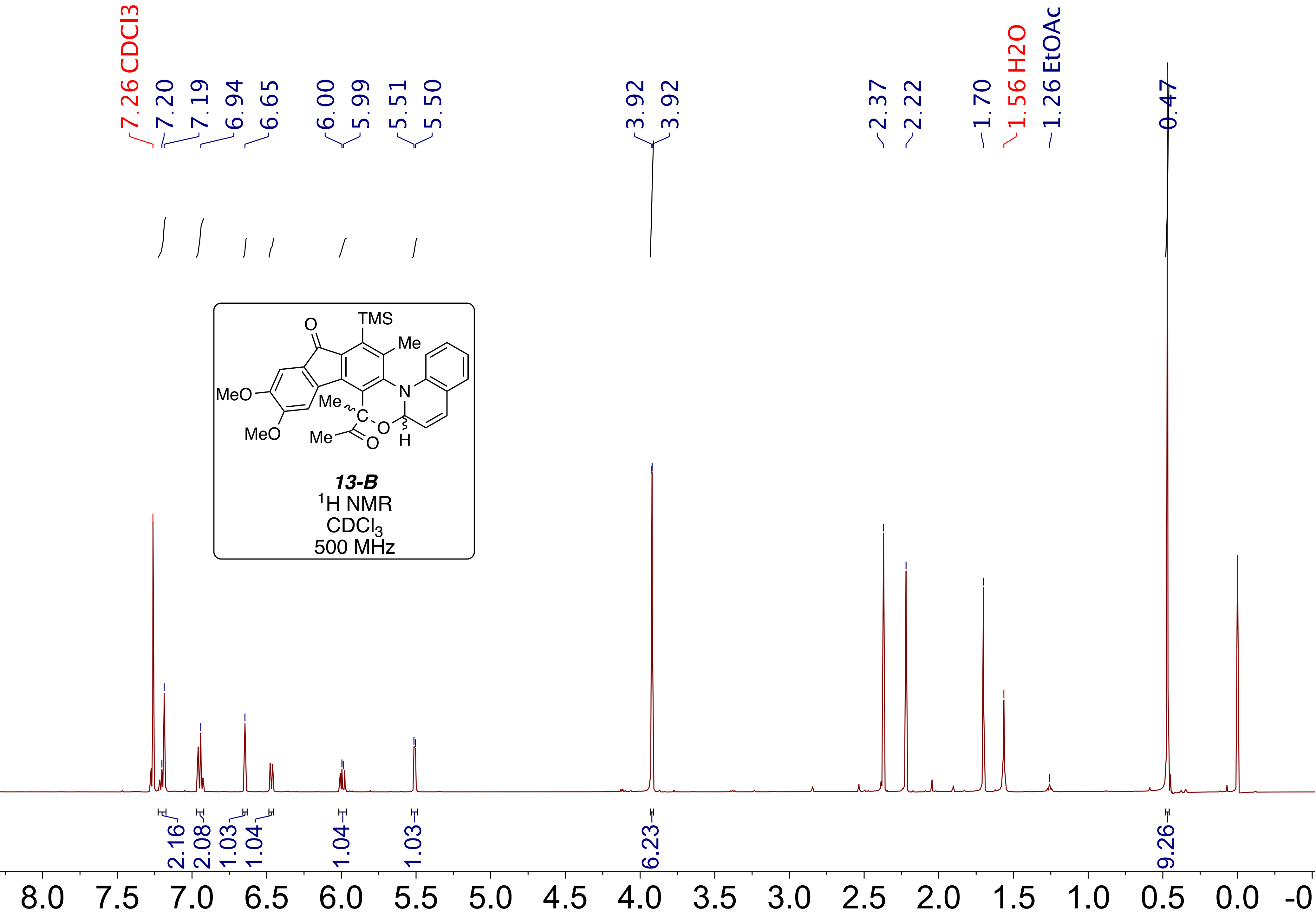
7.26 CDCl3
7.20
7.19
6.94
6.65
6.00
5.99
5.51
5.50
3.92
3.92
2.37
2.22
1.70
1.56 H2O
1.26 EtOAc
0.47
TMS
Me
O
MeO
MeO
Me
C
O
N
H
Me
O
13-B
^{1}H NMR
$CDCl_3$
500 MHz
2.16
2.08
1.03
1.04
1.04
1.03
6.23
9.26
8.0
7.5
7.0
6.5
6.0
5.5
5.0
4.5
4.0
3.5
3.0
2.5
2.0
1.5
1.0
0.5
0.0

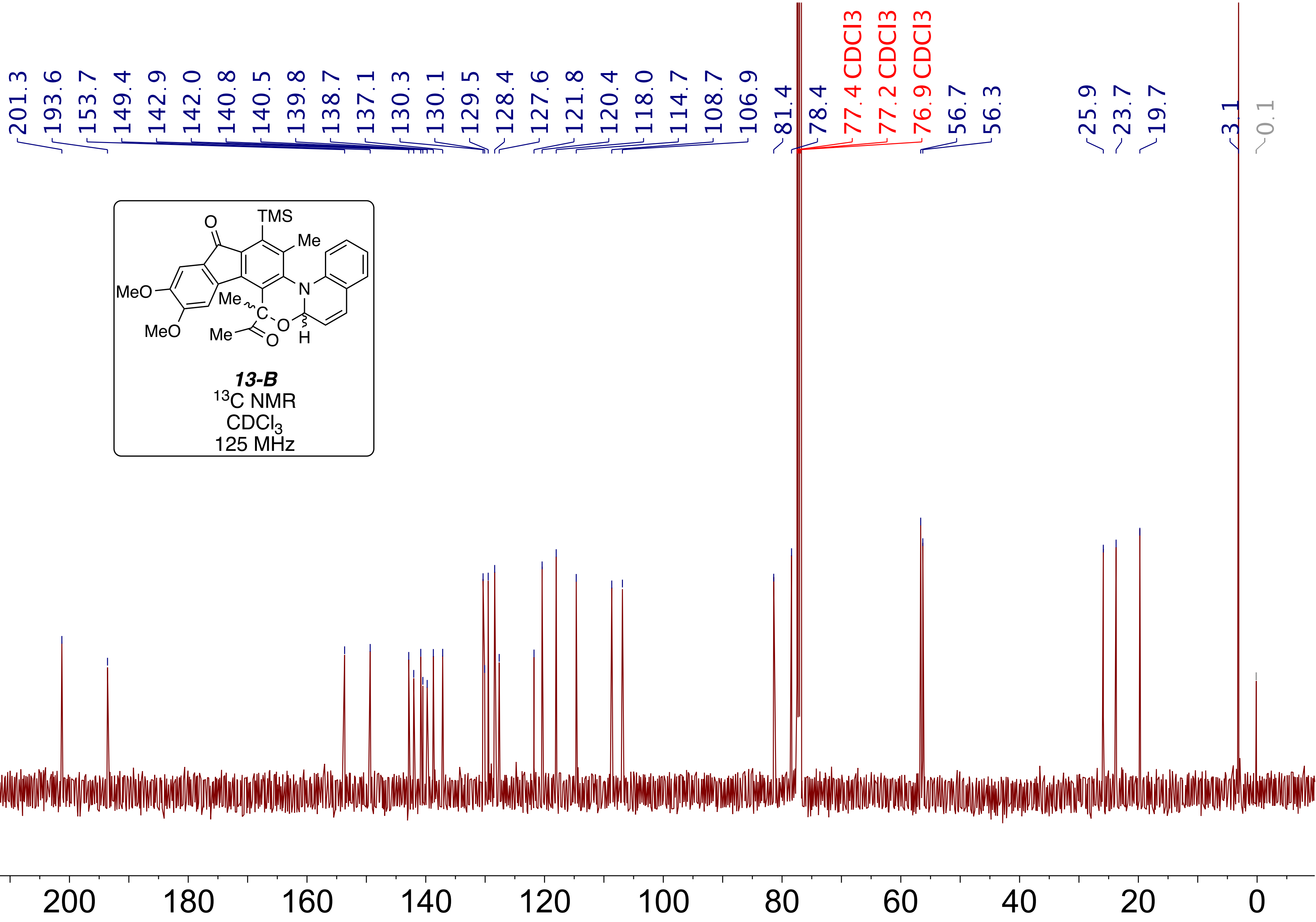
201.3
193.6
153.7
149.4
142.9
142.0
140.8
140.5
139.8
138.7
137.1
130.3
130.1
129.5
128.4
127.6
121.8
120.4
118.0
114.7
108.7
106.9
81.4
78.4
77.4 CDCl3
77.2 CDCl3
76.9 CDCl3
56.7
56.3
25.9
23.7
19.7
3.1
0.1
TMS
O
Me
N
MeO
Me
C
O
H
MeO
Me
O
13-B
^{13}C NMR
$CDCl_3$
125 MHz
200
180
160
140
120
100
80
60
40
20
0

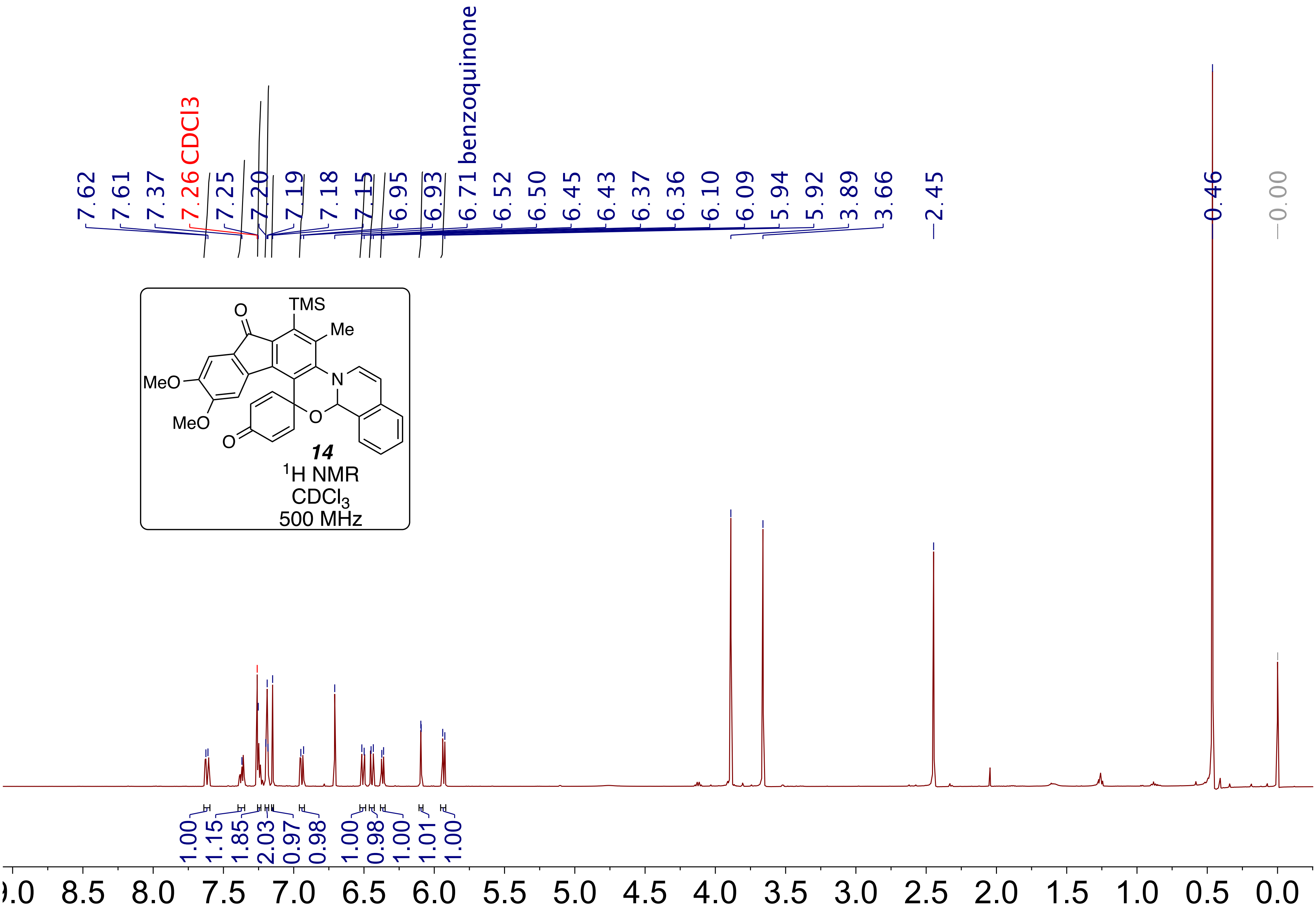
7.62
7.61
7.37
7.26 CDCl3
7.25
7.20
7.19
7.18
7.15
6.95
6.93
6.71 benzoquinone
6.52
6.50
6.45
6.43
6.37
6.36
6.10
6.09
5.94
5.92
3.89
3.66
2.45
0.46
0.00
TMS
Me
MeO
MeO
N
O
O
O
14
^{1}H NMR
$CDCl_3$
500 MHz
1.00
1.15
1.85
2.03
0.97
0.98
1.00
0.98
1.00
1.01
1.00
9.0 8.5 8.0 7.5 7.0 6.5 6.0 5.5 5.0 4.5 4.0 3.5 3.0 2.5 2.0 1.5 1.0 0.5 0.0

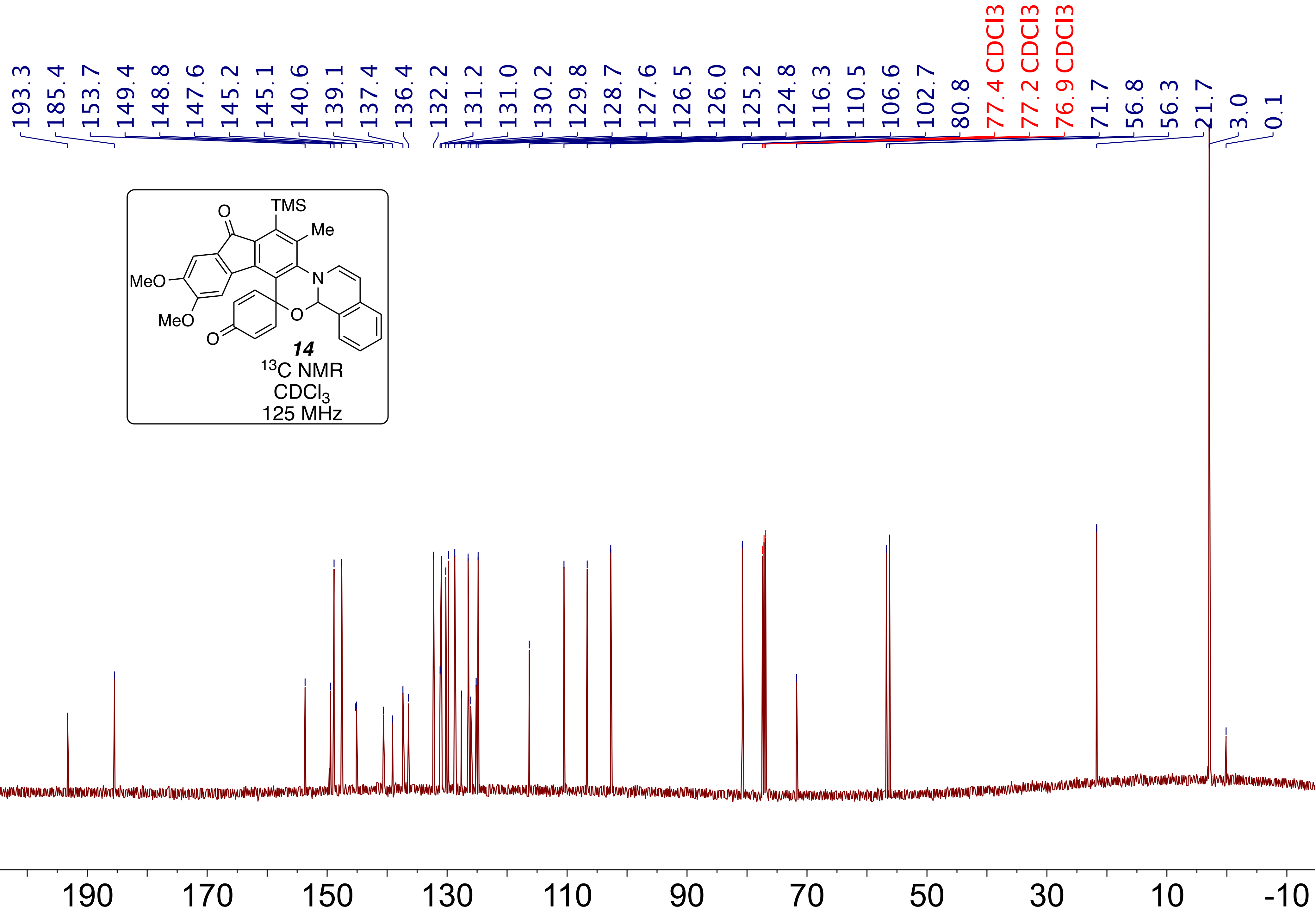
193.3
185.4
153.7
149.4
148.8
147.6
145.2
145.1
140.6
139.1
137.4
136.4
132.2
131.2
131.0
130.2
129.8
128.7
127.6
126.5
126.0
125.2
124.8
116.3
110.5
106.6
102.7
80.8
77.4 CDCl3
77.2 CDCl3
76.9 CDCl3
71.7
56.8
56.3
21.7
3.0
0.1
TMS
Me
O
N
MeO
MeO
O
O
14
13C NMR
CDCl3
125 MHz
190
170
150
130
110
90
70
50
30
10
-10

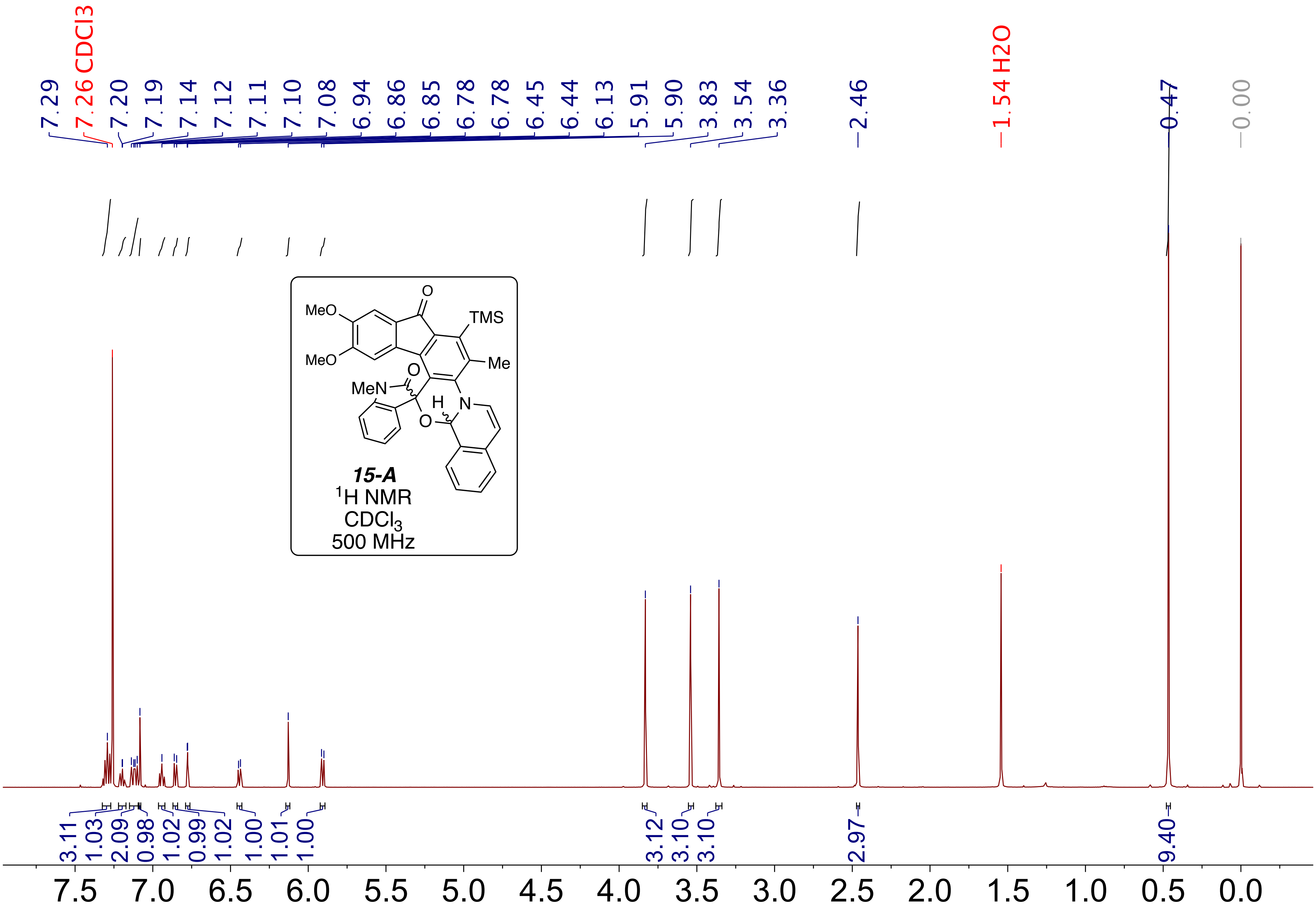
7.29
7.26 CDCl3
7.20
7.19
7.14
7.12
7.11
7.10
7.08
6.94
6.86
6.85
6.78
6.78
6.45
6.44
6.13
5.91
5.90
3.83
3.54
3.36
2.46
1.54 H2O
0.47
0.00
MeO
MeO
O
TMS
Me
MeN
O
H
N
O
15-A
1H NMR
CDCl3
500 MHz
3.11
1.03
2.09
0.98
1.02
0.99
1.02
1.00
1.01
1.00
3.12
3.10
3.10
2.97
9.40
7.5
7.0
6.5
6.0
5.5
5.0
4.5
4.0
3.5
3.0
2.5
2.0
1.5
1.0
0.5
0.0

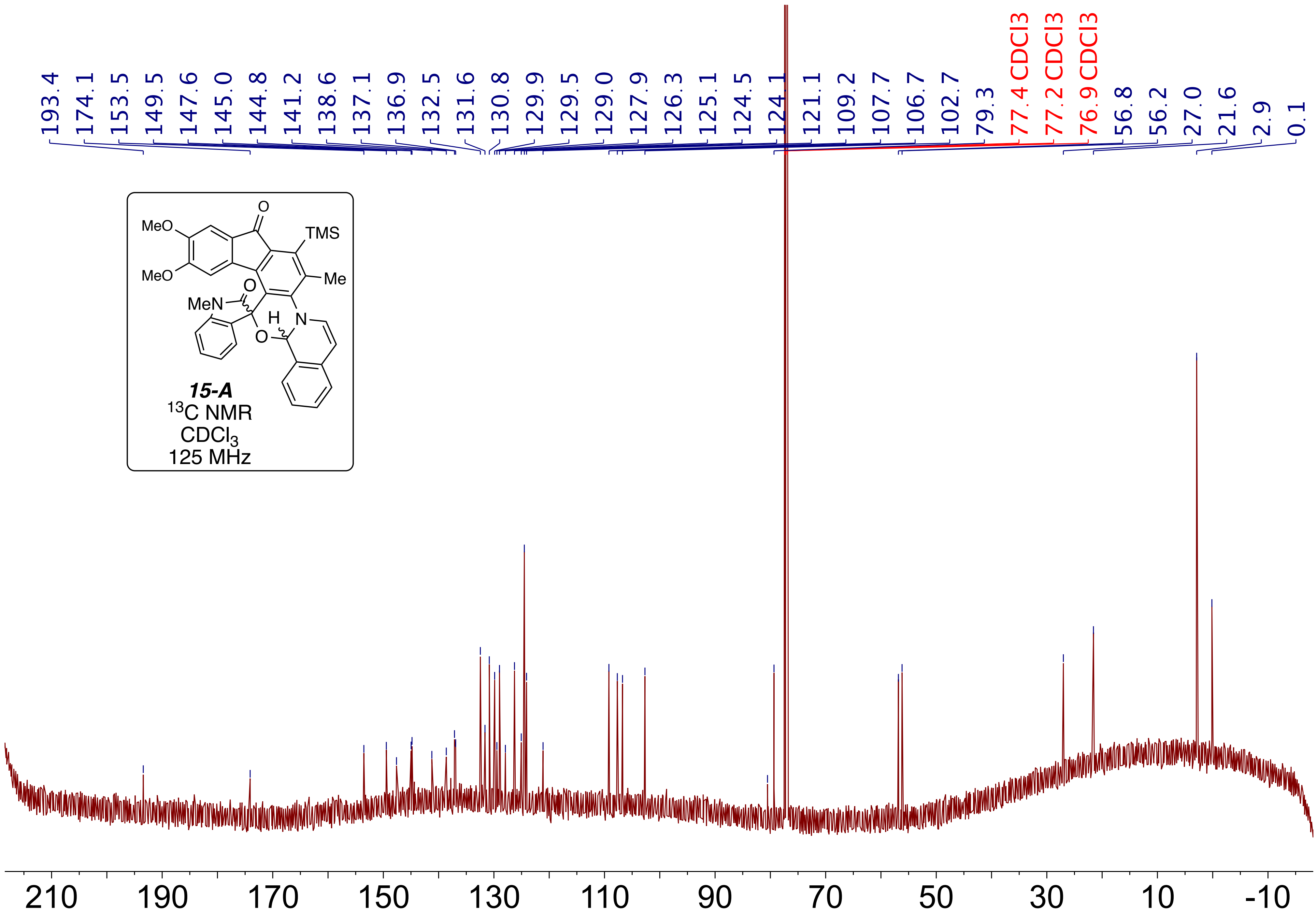
193.4
174.1
153.5
149.5
147.6
145.0
144.8
141.2
138.6
137.1
136.9
132.5
131.6
130.8
129.9
129.5
129.0
127.9
126.3
125.1
124.5
124.1
121.1
109.2
107.7
106.7
102.7
79.3
77.4 CDCl3
77.2 CDCl3
76.9 CDCl3
56.8
56.2
27.0
21.6
2.9
0.1
MeO
MeO
O
TMS
Me
MeN
O
H
N
O
15-A
^{13}C NMR
$CDCl_3$
125 MHz
210
190
170
150
130
110
90
70
50
30
10
-10

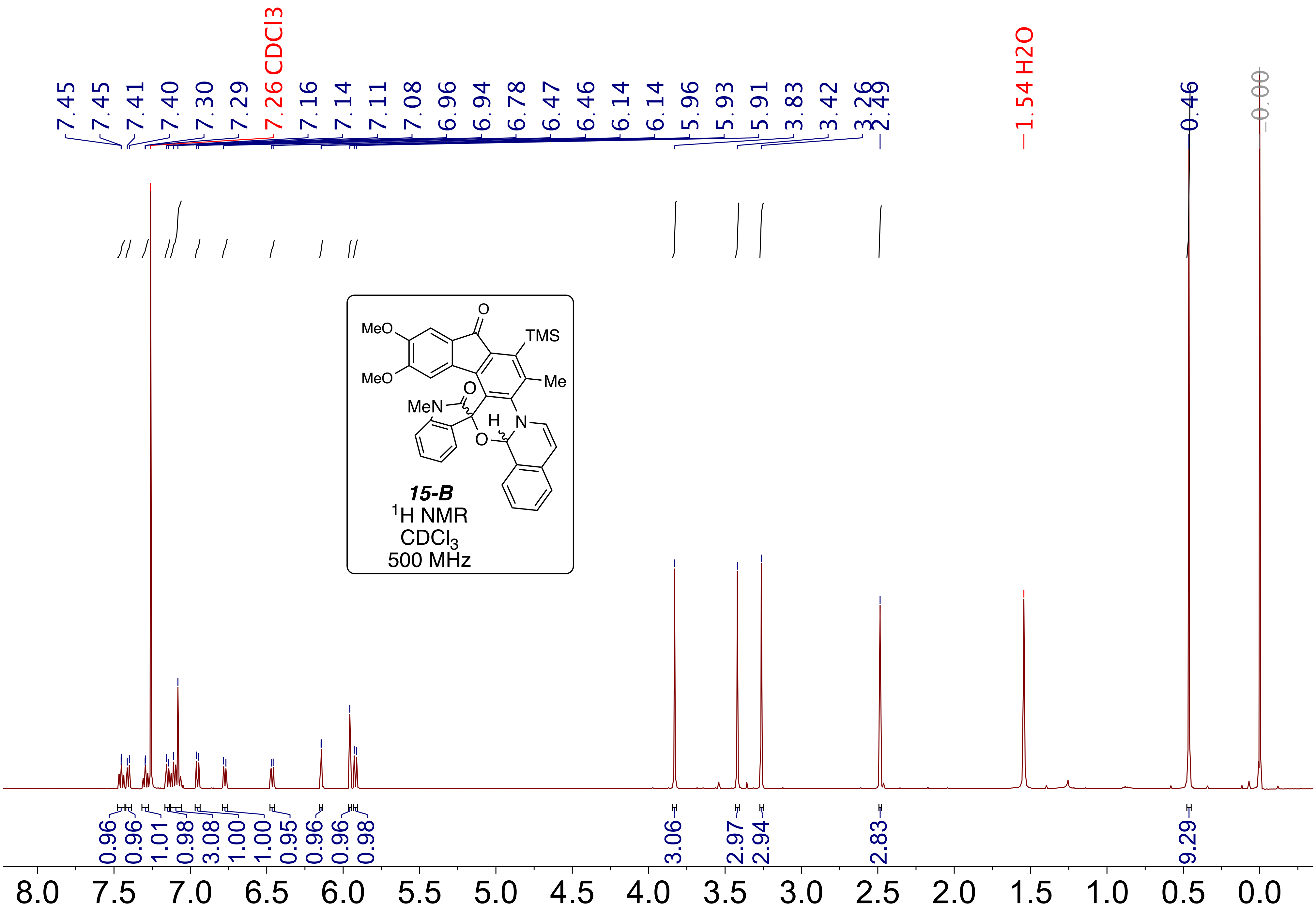

7.45 7.45 7.41 7.40 7.30 7.29 7.26 CDCl3 7.16 7.14 7.11 7.08 6.96 6.94 6.78 6.47 6.46 6.14 6.14 5.96 5.93 5.91 3.83 3.42 3.26 2.49 1.54 H2O 0.46 -0.00
MeO
MeO
O
TMS
Me
MeN
O
H
N
O
15-B
1H NMR
CDCl3
500 MHz
0.96 0.96 1.01 0.98 3.08 1.00 1.00 0.95 0.96 0.96 0.98 3.06 2.97 2.94 2.83 9.29
8.0 7.5 7.0 6.5 6.0 5.5 5.0 4.5 4.0 3.5 3.0 2.5 2.0 1.5 1.0 0.5 0.0

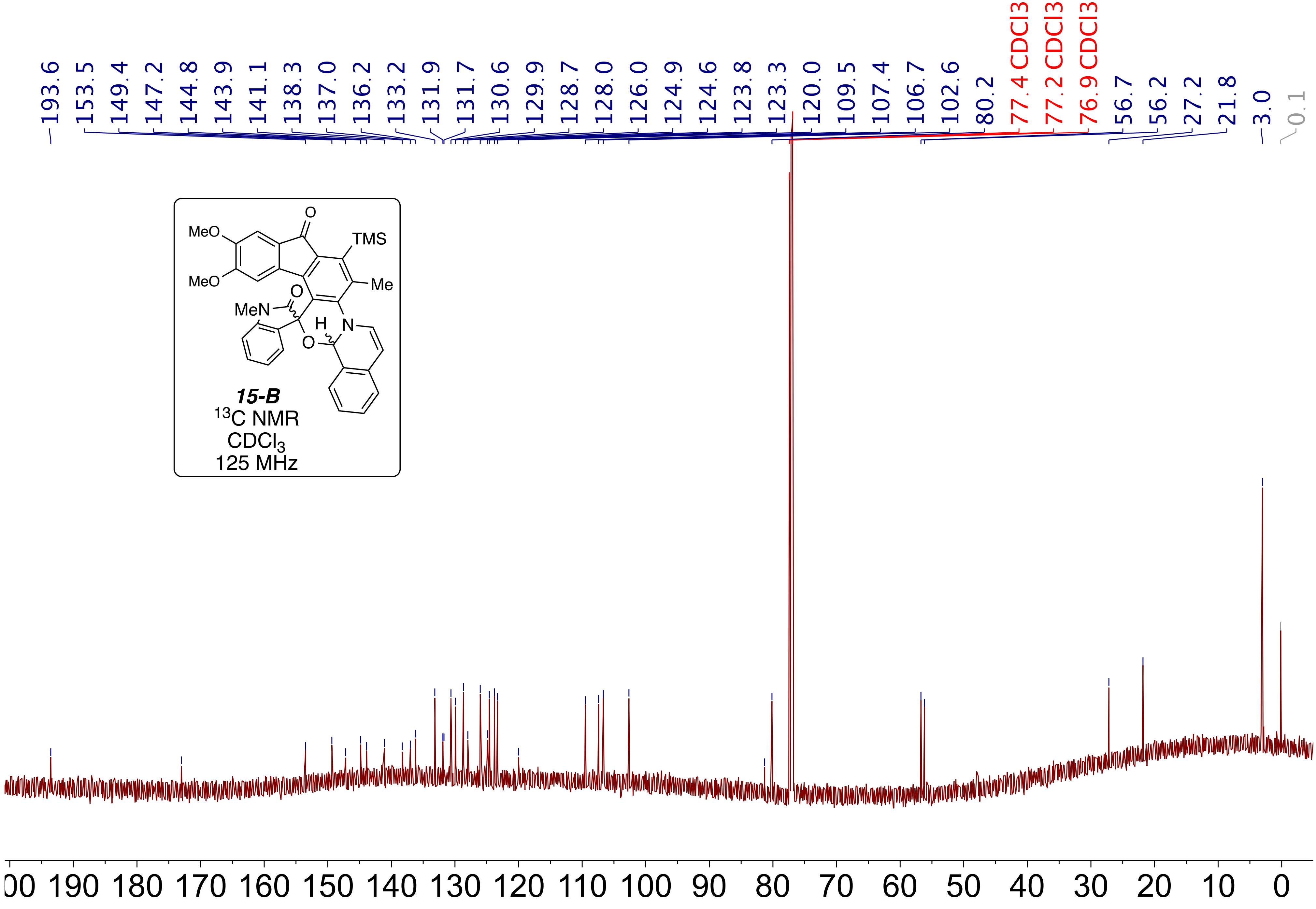

193.6
153.5
149.4
147.2
144.8
143.9
141.1
138.3
137.0
136.2
133.2
131.9
131.7
130.6
129.9
128.7
128.0
126.0
124.9
124.6
123.8
123.3
120.0
109.5
107.4
106.7
102.6
80.2
77.4 CDCl3
77.2 CDCl3
76.9 CDCl3
56.7
56.2
27.2
21.8
3.0
0.1
MeO
MeO
O
TMS
Me
MeN
O
H
N
O
15-B
13C NMR
CDCl3
125 MHz
00 190 180 170 160 150 140 130 120 110 100 90 80 70 60 50 40 30 20 10 0

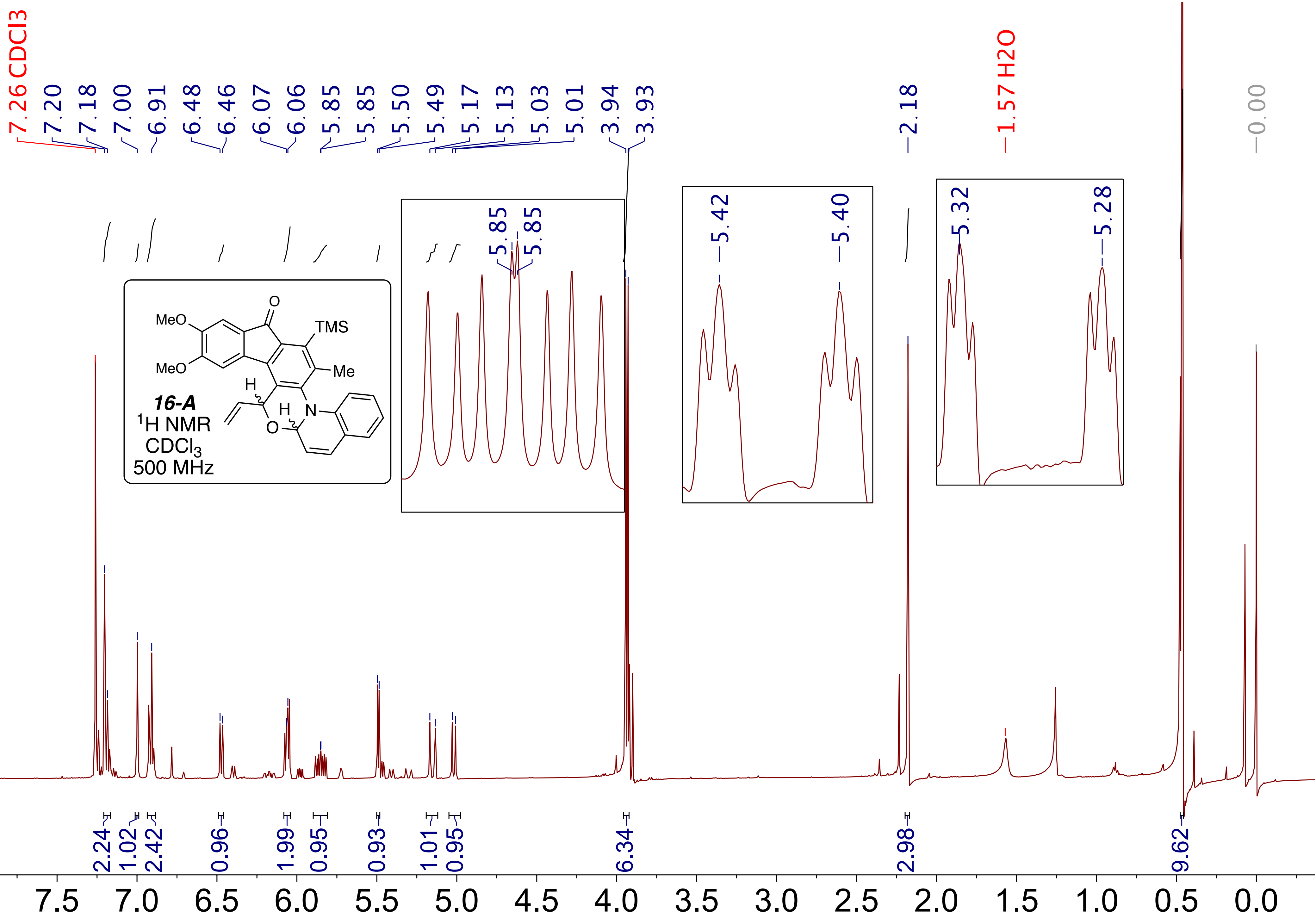
7.26 CDCl3
7.20
7.18
7.00
6.91
6.48
6.46
6.07
6.06
5.85
5.85
5.50
5.49
5.17
5.13
5.03
5.01
3.94
3.93
2.18
1.57 H2O
0.00
5.85
5.85
5.42
5.40
5.32
5.28
MeO
MeO
O
TMS
Me
H
H
N
O
16-A
1H NMR
CDCl3
500 MHz
2.24
1.02
2.42
0.96
1.99
0.95
0.93
1.01
0.95
6.34
2.98
9.62
7.5
7.0
6.5
6.0
5.5
5.0
4.5
4.0
3.5
3.0
2.5
2.0
1.5
1.0
0.5
0.0

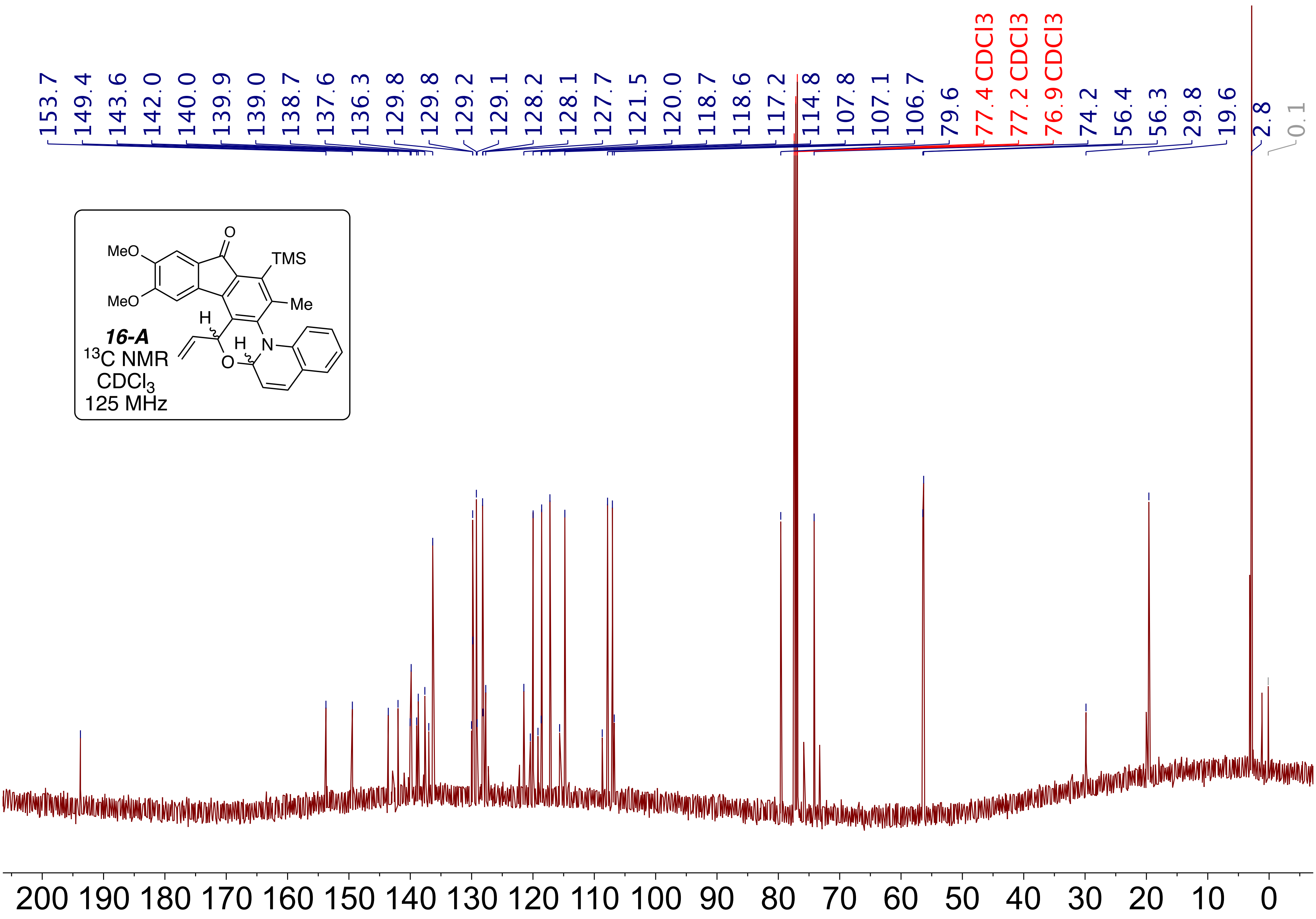
153.7
149.4
143.6
142.0
140.0
139.9
139.0
138.7
137.6
136.3
129.8
129.8
129.2
129.1
128.2
128.1
127.7
121.5
120.0
118.7
118.6
117.2
114.8
107.8
107.1
106.7
79.6
77.4 CDCl3
77.2 CDCl3
76.9 CDCl3
74.2
56.4
56.3
29.8
19.6
2.8
0.1
MeO
MeO
O
TMS
Me
H
H
N
O
16-A
13C NMR
CDCl3
125 MHz
200 190 180 170 160 150 140 130 120 110 100 90 80 70 60 50 40 30 20 10 0

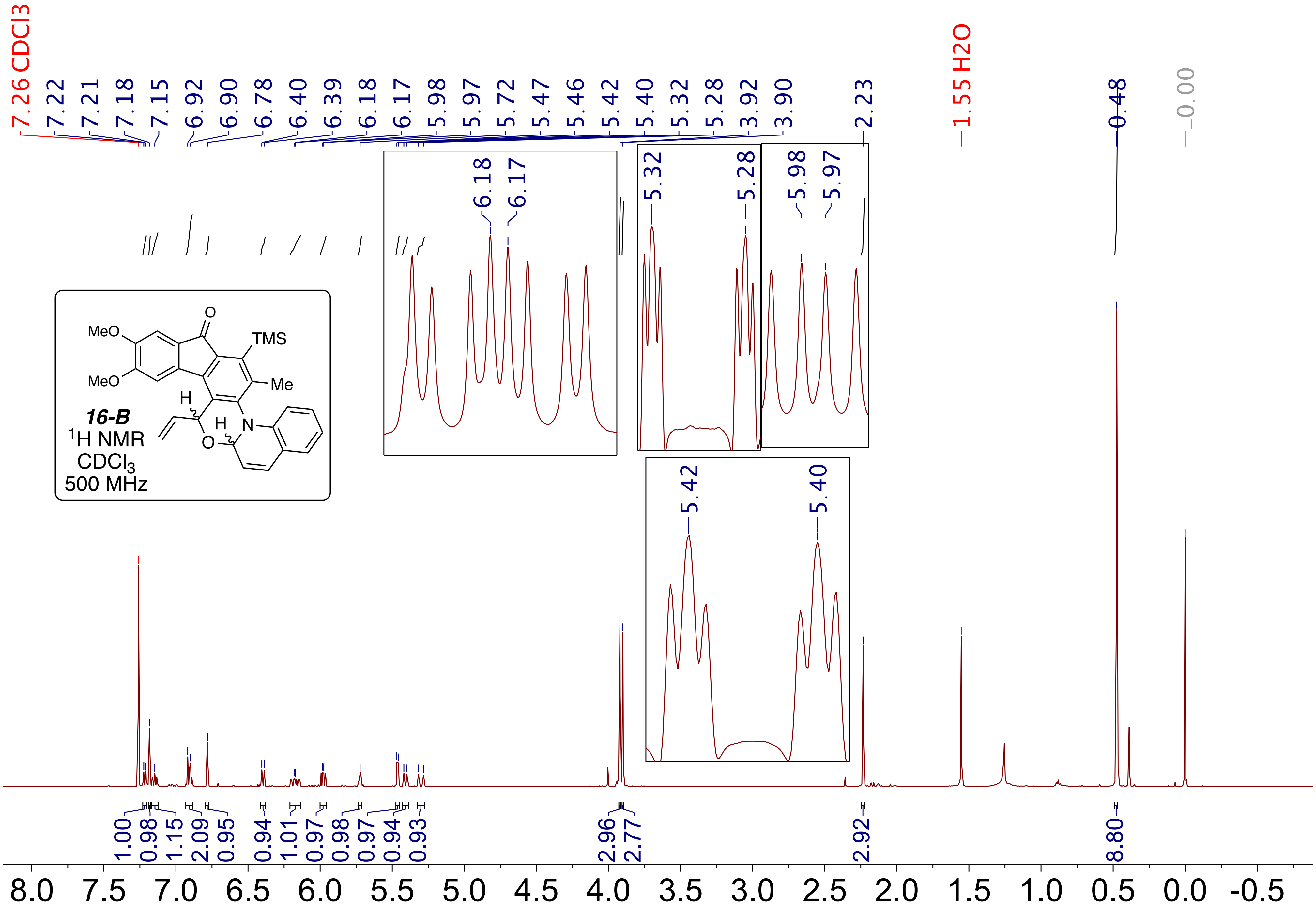
16-B
^{1}H NMR
CDCl3
500 MHz
MeO
MeO
TMS
Me
O
H
N
7.26 CDCl3
7.22
7.21
7.18
7.15
6.92
6.90
6.78
6.40
6.39
6.18
6.17
5.98
5.97
5.72
5.47
5.46
5.42
5.40
5.32
5.28
3.92
3.90
2.23
1.55 H2O
0.48
0.00
1.00
0.98
1.15
2.09
0.95
0.94
1.01
0.97
0.98
0.97
0.94
0.93
2.96
2.77
2.92
8.80
8.0
7.5
7.0
6.5
6.0
5.5
5.0
4.5
4.0
3.5
3.0
2.5
2.0
1.5
1.0
0.5
0.0
-0.5

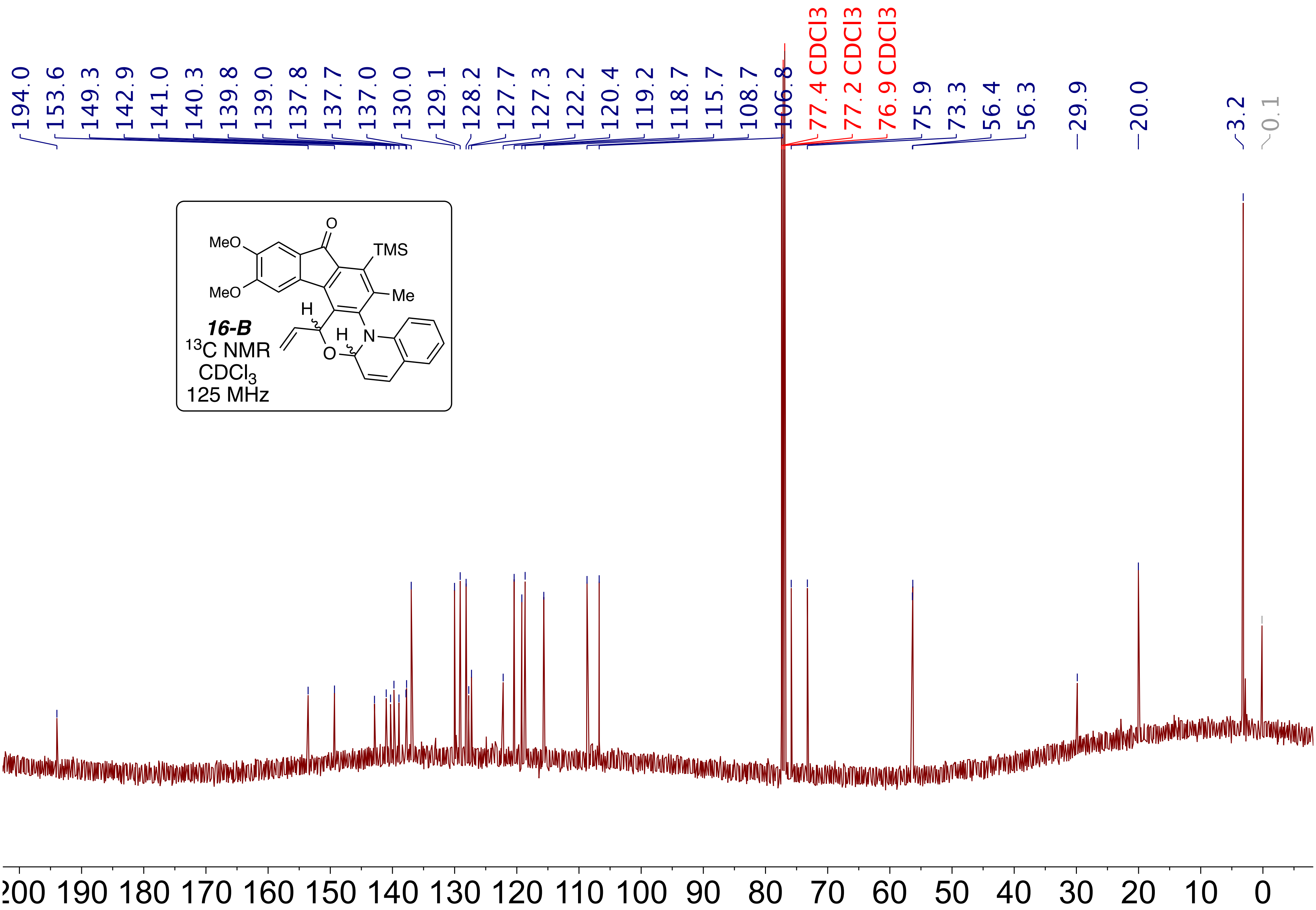
194.0
153.6
149.3
142.9
141.0
140.3
139.8
139.0
137.8
137.7
137.0
130.0
129.1
128.2
127.7
127.3
122.2
120.4
119.2
118.7
115.7
108.7
106.8
77.4 CDCl3
77.2 CDCl3
76.9 CDCl3
75.9
73.3
56.4
56.3
29.9
20.0
3.2
0.1
MeO
MeO
O
TMS
Me
H
H
N
O
16-B
13C NMR
CDCl3
125 MHz
200 190 180 170 160 150 140 130 120 110 100 90 80 70 60 50 40 30 20 10 0

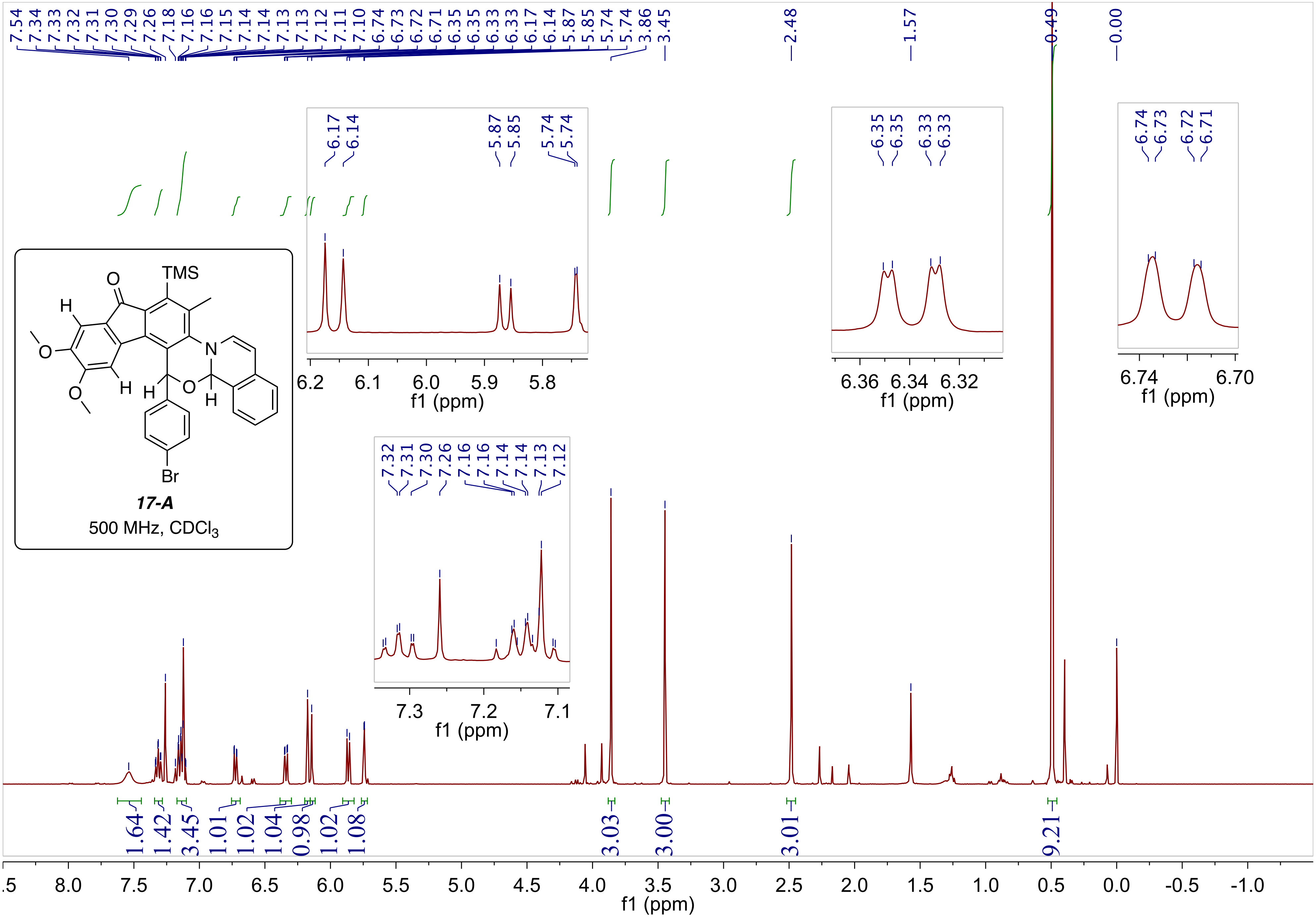
TMS
O
H
N
O
O
H
H
O
H
Br
17-A
500 MHz, CDCl3
f1 (ppm)
1.64
1.42
3.45
1.01
1.02
1.04
0.98
1.02
1.08
3.03
3.00
3.01
9.21

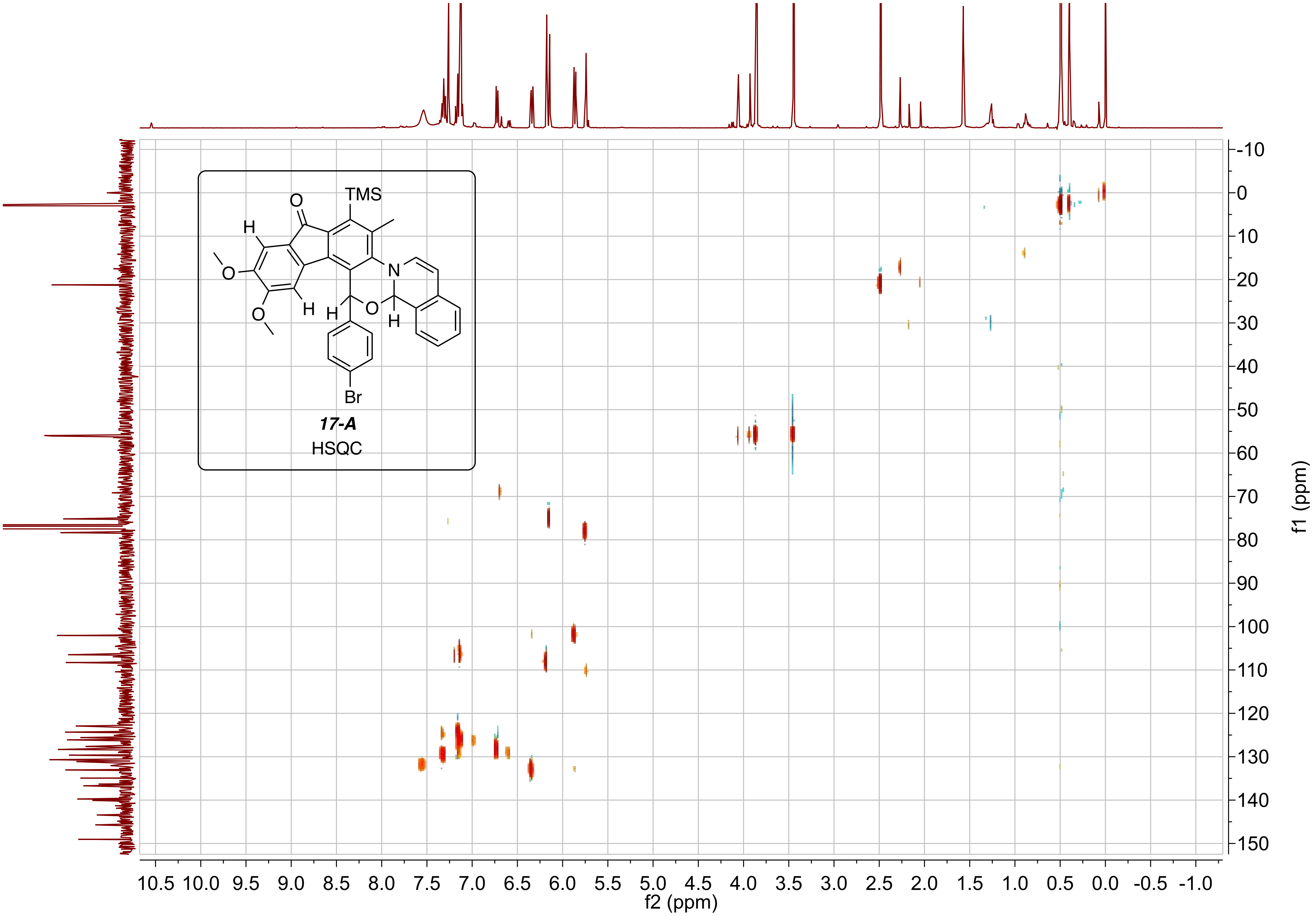
TMS
O
N
H
Br
17-A
HSQC
f2 (ppm)
f1 (ppm)

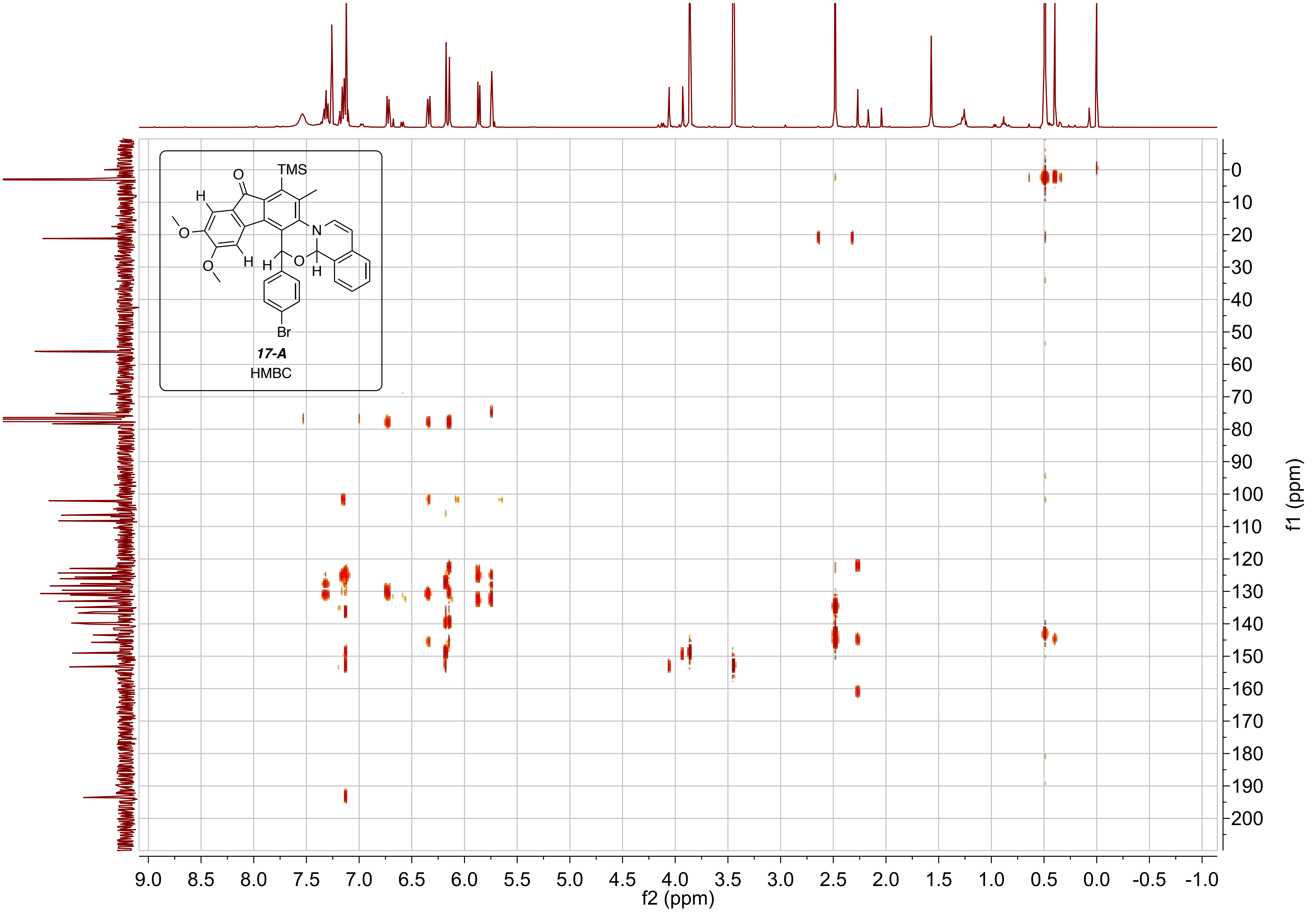
TMS
O
H
O
O
N
H
H
O
H
Br
17-A
HMBC
f2 (ppm)
f1 (ppm)

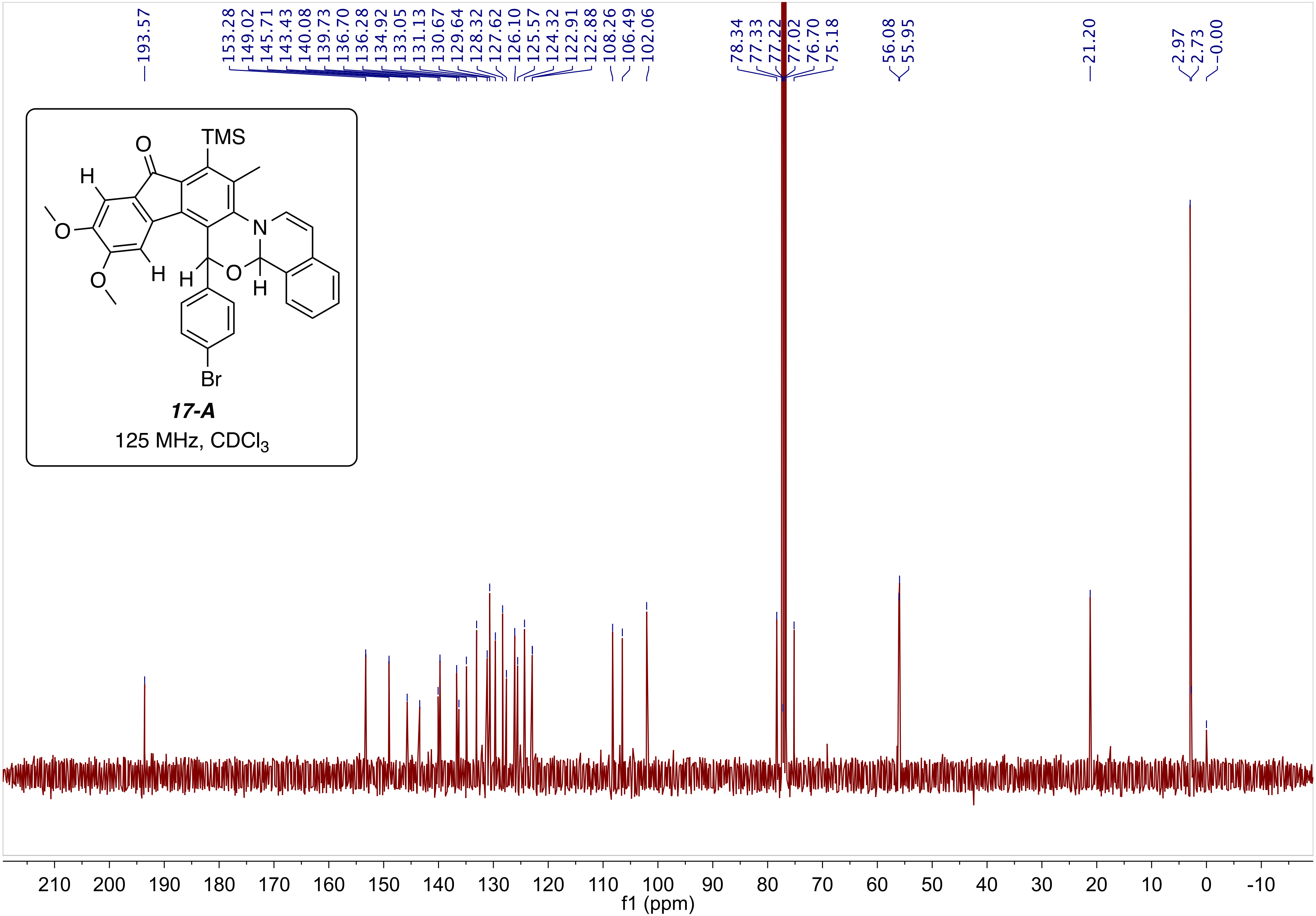
193.57
153.28
149.02
145.71
143.43
140.08
139.73
136.70
136.28
134.92
133.05
131.13
130.67
129.64
128.32
127.62
126.10
125.57
124.32
122.91
122.88
108.26
106.49
102.06
78.34
77.33
77.22
77.02
76.70
75.18
56.08
55.95
21.20
2.97
2.73
-0.00
TMS
O
H
N
H
O
H
H
Br
17-A
125 MHz, $CDCl_3$
210 200 190 180 170 160 150 140 130 120 110 100 90 80 70 60 50 40 30 20 10 0 -10
f1 (ppm)

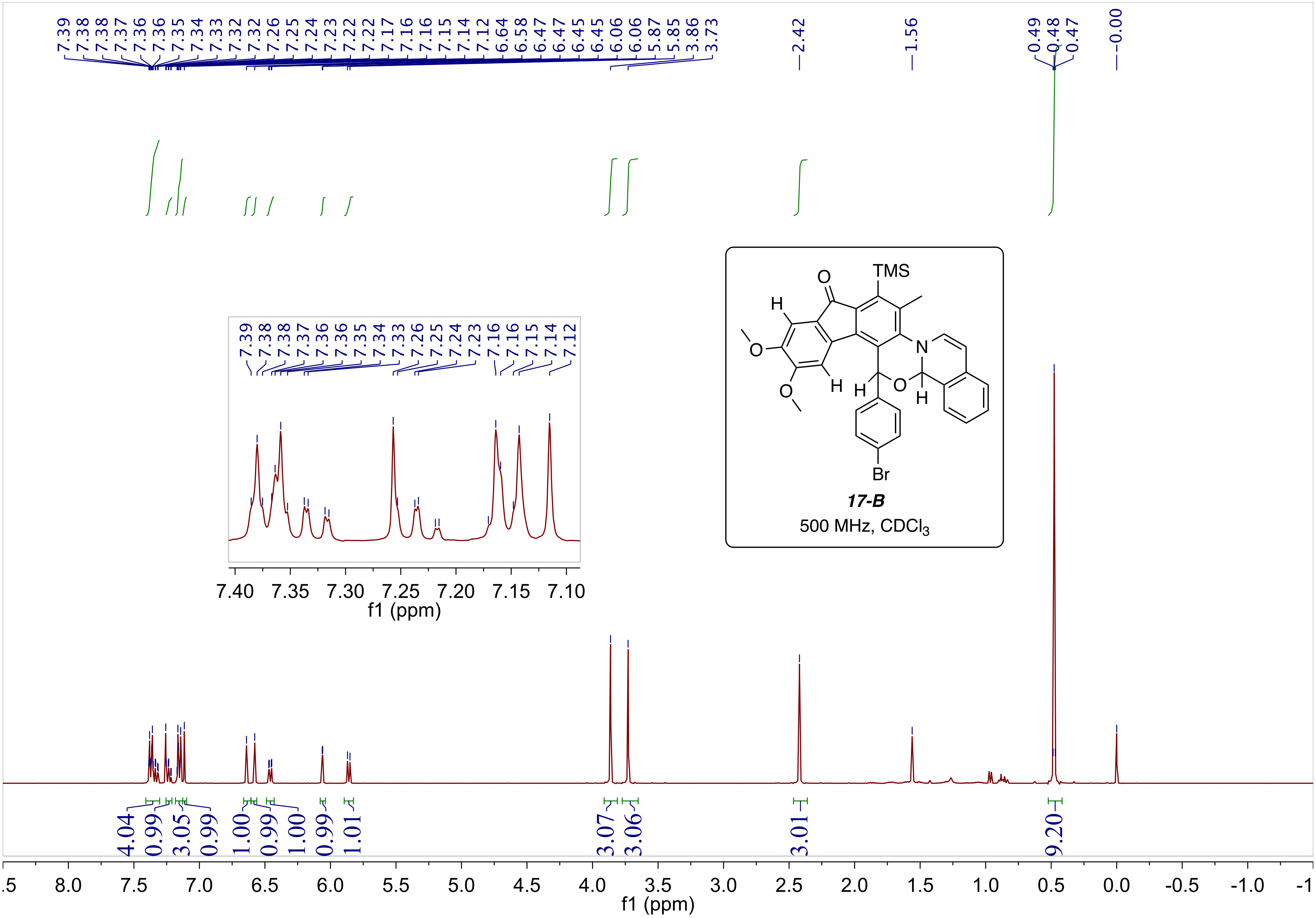

7.39 7.38 7.38 7.37 7.36 7.36 7.35 7.34 7.33 7.32 7.32 7.26 7.25 7.24 7.23 7.22 7.22 7.17 7.16 7.16 7.15 7.14 7.12 6.64 6.58 6.47 6.47 6.45 6.45 6.06 6.06 5.87 5.85 3.86 3.73
2.42
1.56
0.49 0.48 0.47
-0.00
4.04 0.99 3.05 0.99 1.00 0.99 1.00 0.99 1.01 3.07 3.06 3.01 9.20
f1 (ppm)
TMS
O
H
N
Br
17-B
500 MHz, CDCl3

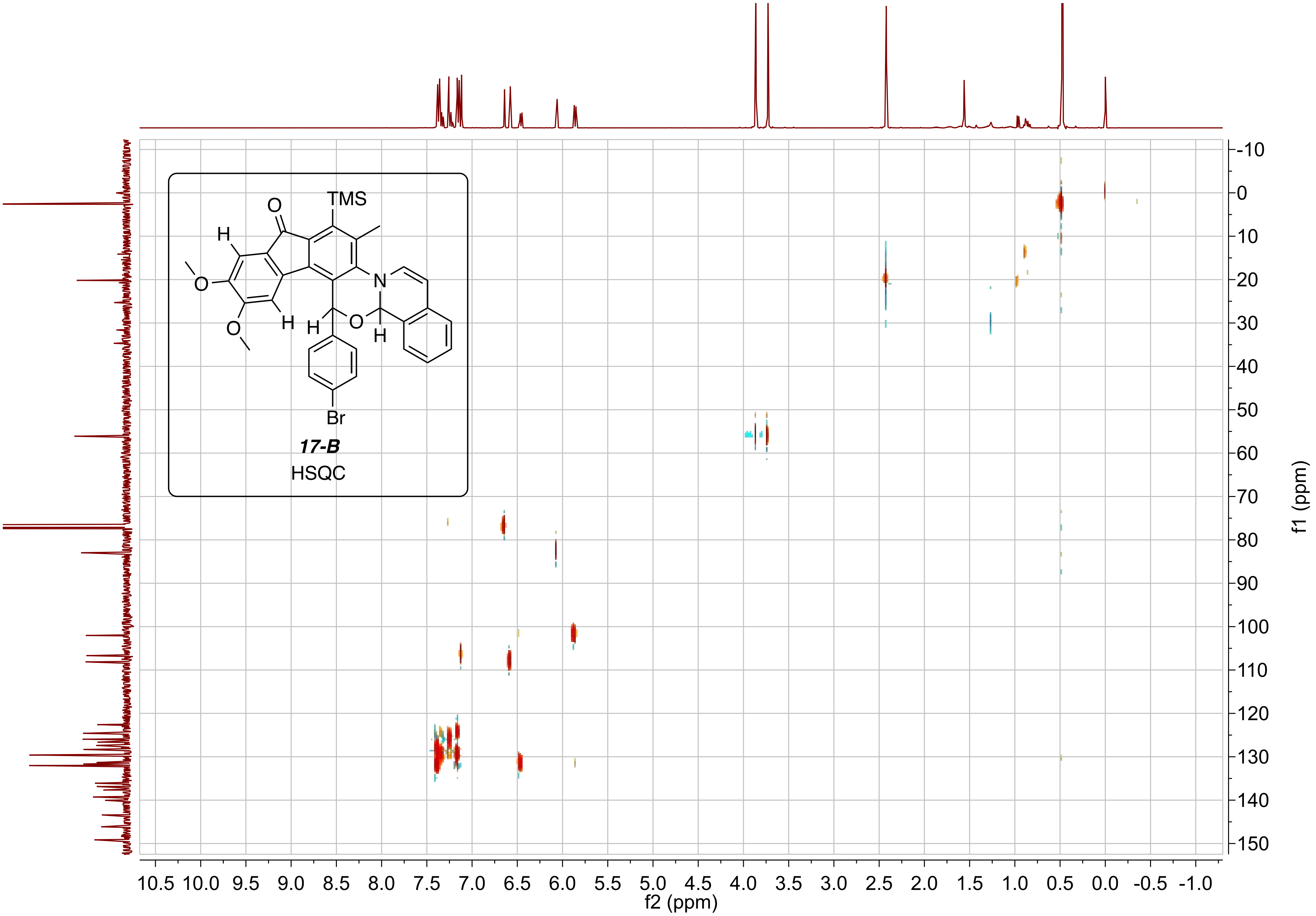
TMS
O
H
O
O
H
H
O
N
H
Br
17-B
HSQC
f2 (ppm)
f1 (ppm)

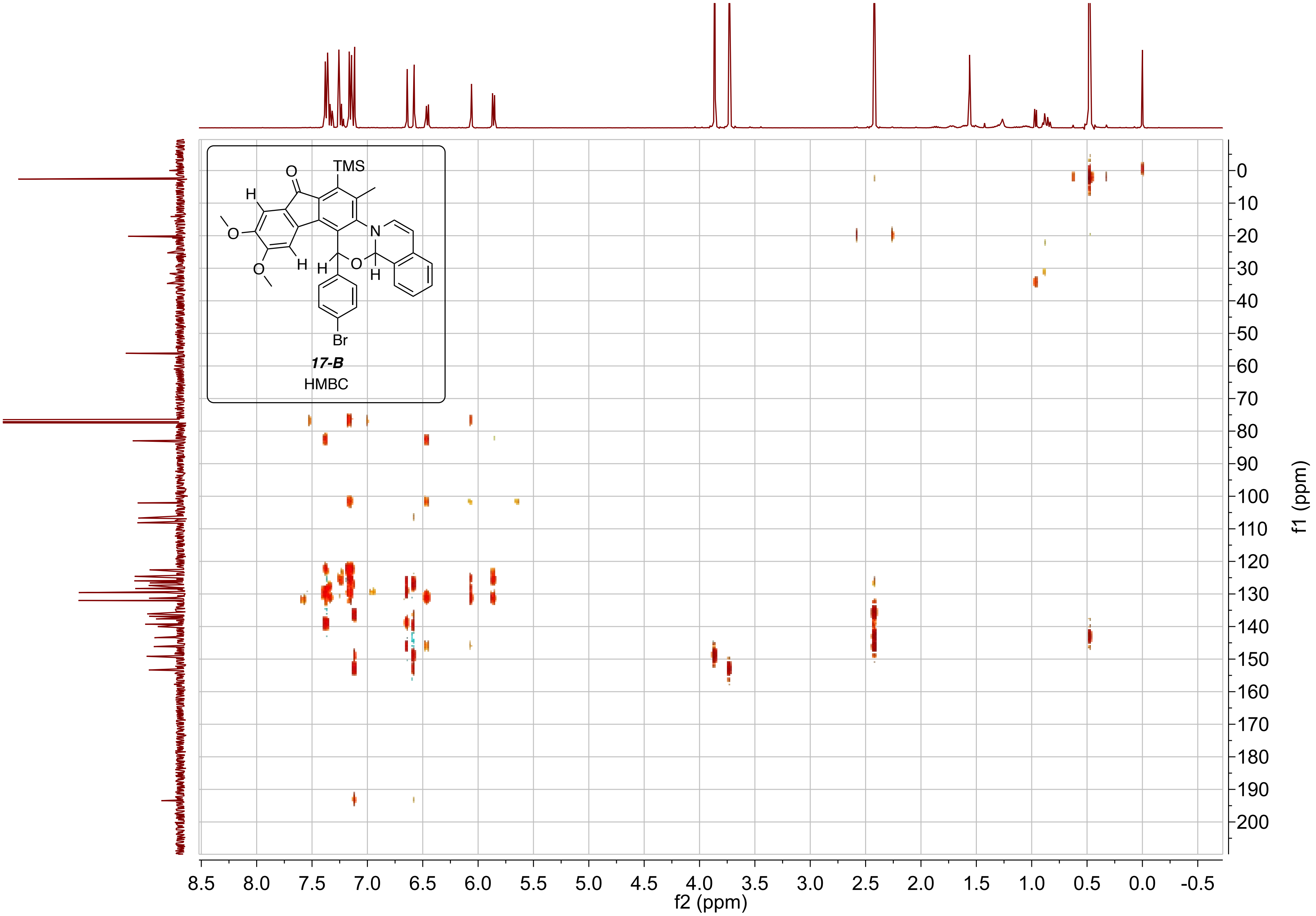
TMS
O
H
N
H
H
O
H
O
Br
17-B
HMBC
f2 (ppm)
f1 (ppm)

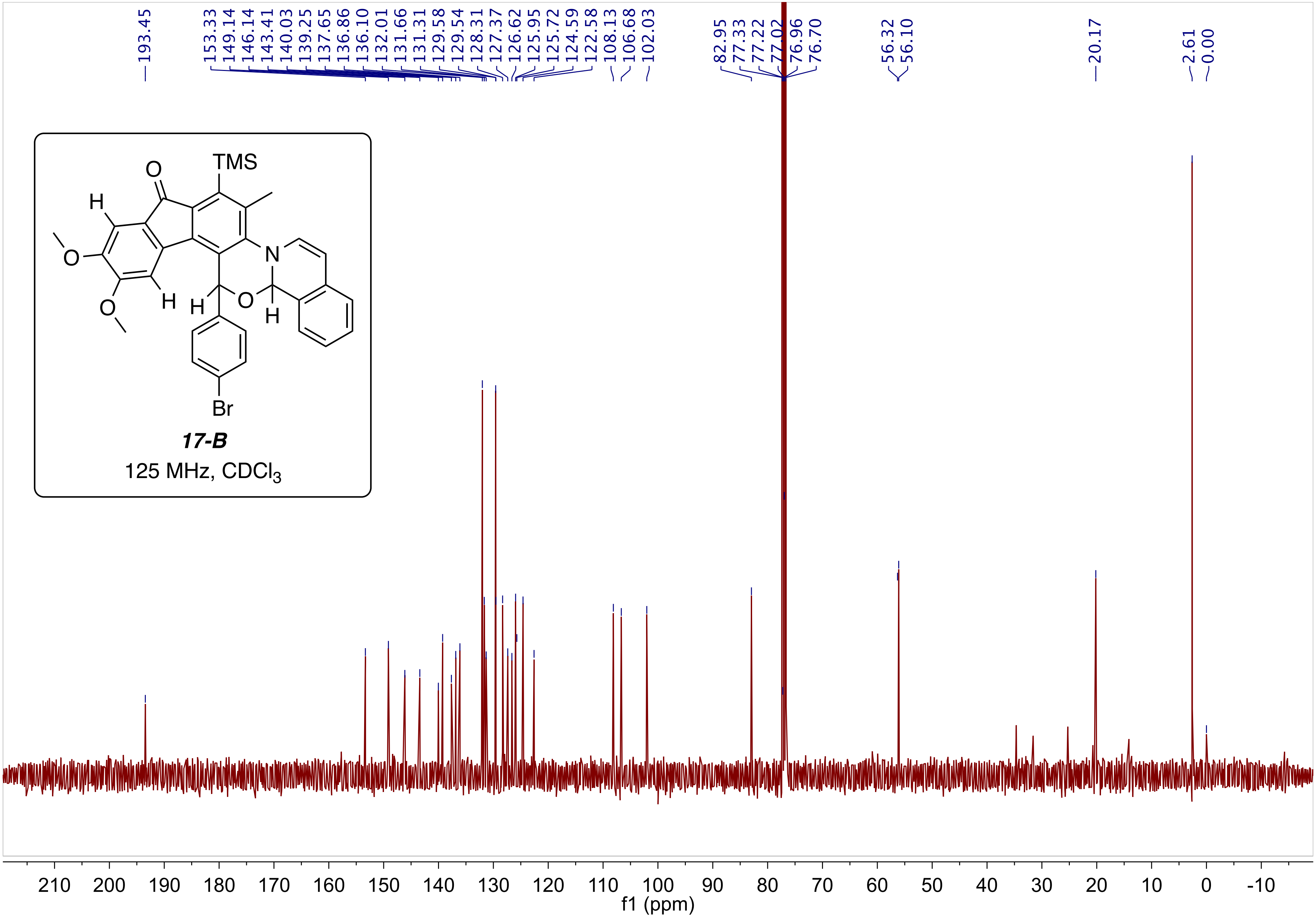
193.45
153.33
149.14
146.14
143.41
140.03
139.25
137.65
136.86
136.10
132.01
131.66
131.31
129.58
129.54
128.31
127.37
126.62
125.95
125.72
124.59
122.58
108.13
106.68
102.03
82.95
77.33
77.22
77.02
76.96
76.70
56.32
56.10
20.17
2.61
0.00
TMS
O
H
N
Br
17-B
125 MHz, $CDCl_3$
210 200 190 180 170 160 150 140 130 120 110 100 90 80 70 60 50 40 30 20 10 0 -10
f1 (ppm)

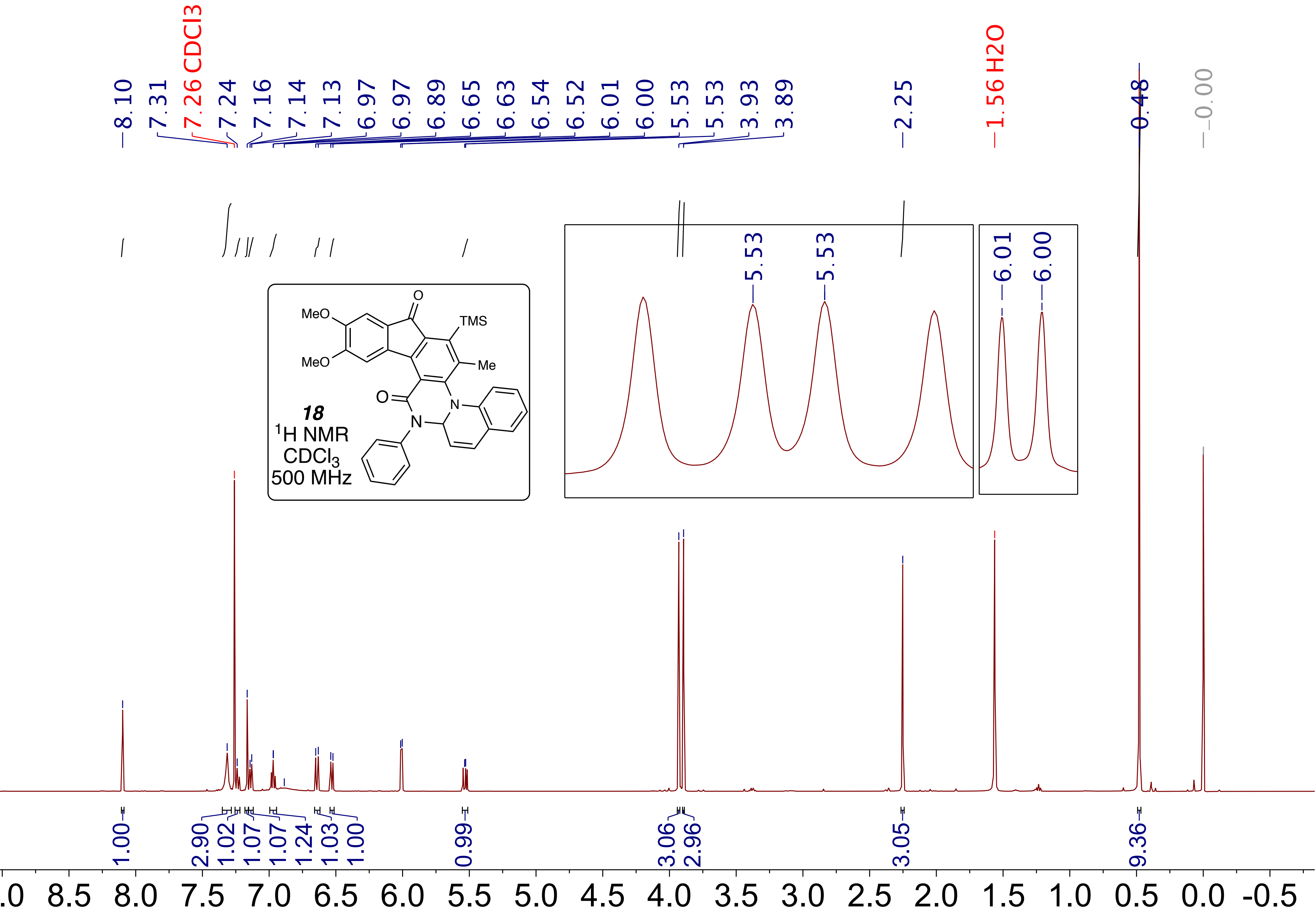

8.10
7.31
7.26 CDCl3
7.24
7.16
7.14
7.13
6.97
6.97
6.89
6.65
6.63
6.54
6.52
6.01
6.00
5.53
5.53
3.93
3.89
2.25
1.56 H2O
0.48
0.00
MeO
MeO
O
TMS
Me
O
N
N
18
1H NMR
CDCl3
500 MHz
1.00
2.90
1.02
1.07
1.07
1.24
1.03
1.00
0.99
3.06
2.96
3.05
9.36
8.5 8.0 7.5 7.0 6.5 6.0 5.5 5.0 4.5 4.0 3.5 3.0 2.5 2.0 1.5 1.0 0.5 0.0 -0.5

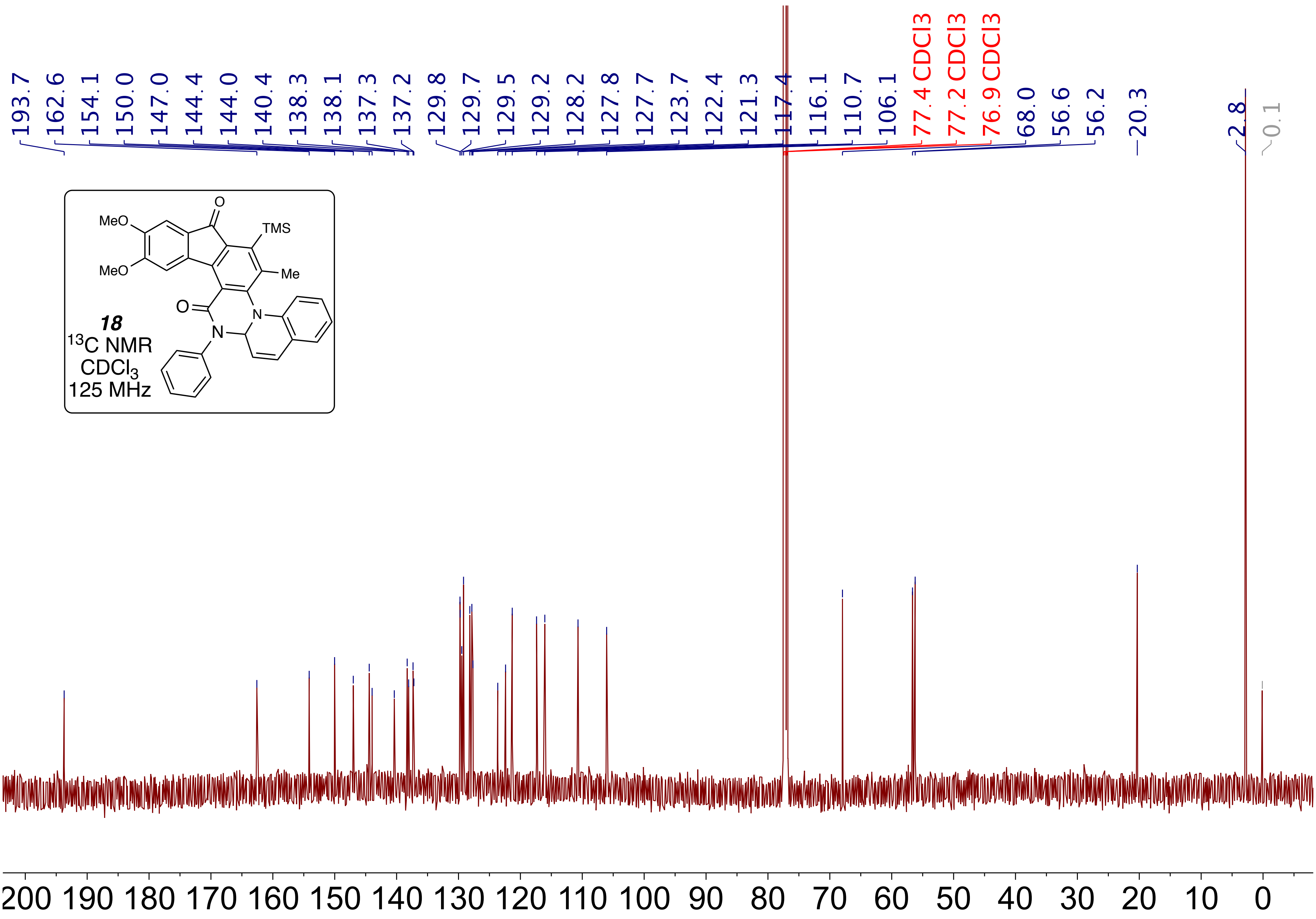
193.7
162.6
154.1
150.0
147.0
144.4
144.0
140.4
138.3
138.1
137.3
137.2
129.8
129.7
129.5
129.2
128.2
127.8
127.7
123.7
122.4
121.3
117.4
116.1
110.7
106.1
77.4 CDCl3
77.2 CDCl3
76.9 CDCl3
68.0
56.6
56.2
20.3
2.8
0.1
MeO
MeO
O
TMS
Me
O
N
N
18
13C NMR
CDCl3
125 MHz
200 190 180 170 160 150 140 130 120 110 100 90 80 70 60 50 40 30 20 10 0

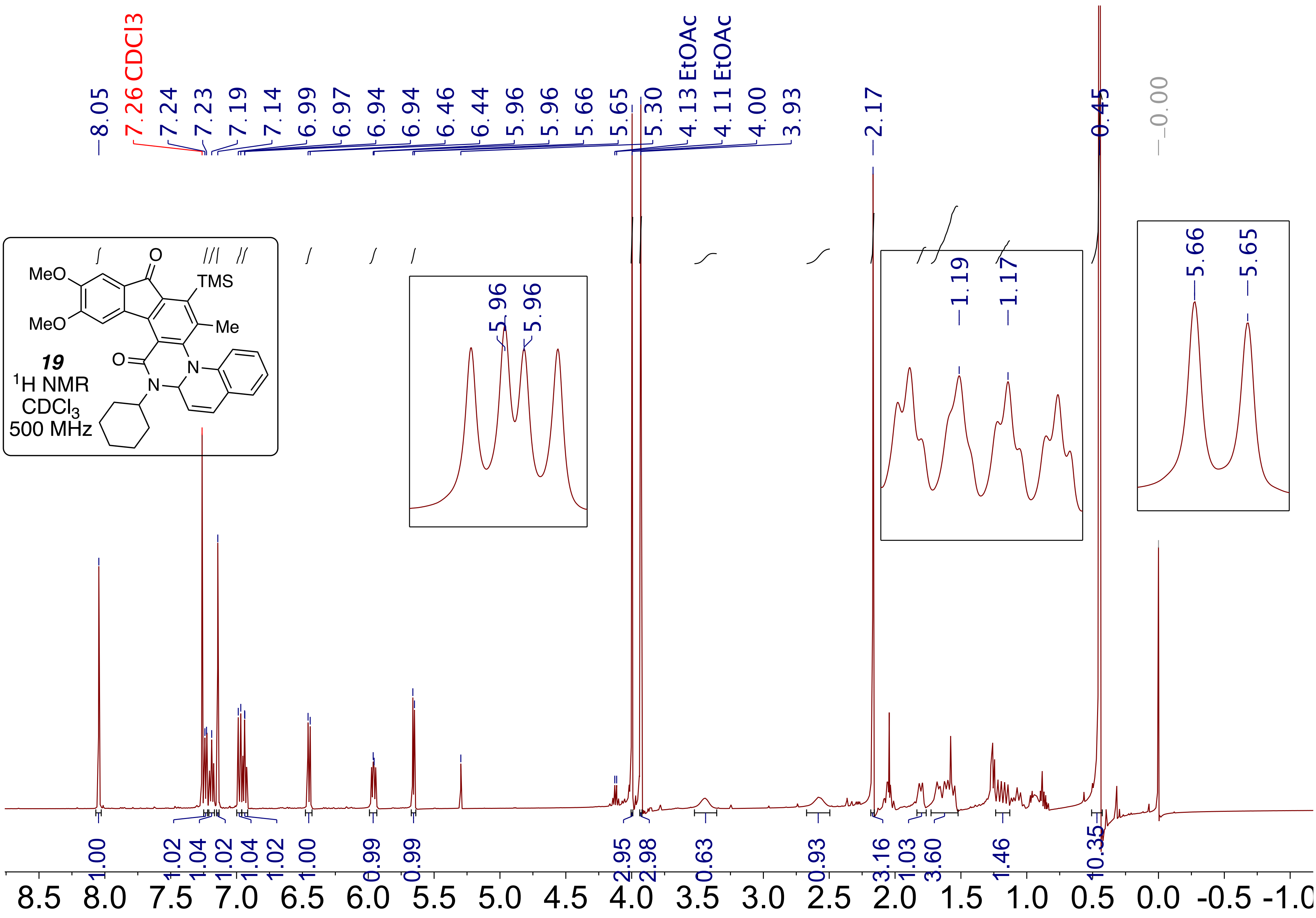

19
1H NMR
CDCl3
500 MHz
MeO
MeO
O
TMS
Me
O
N
N
8.05
7.26 CDCl3
7.24
7.23
7.19
7.14
6.99
6.97
6.94
6.94
6.46
6.44
5.96
5.96
5.66
5.65
5.30
4.13 EtOAc
4.11 EtOAc
4.00
3.93
2.17
0.45
0.00
1.19
1.17
1.00
1.02
1.04
1.02
1.04
1.02
1.00
0.99
0.99
2.95
2.98
0.63
0.93
3.16
1.03
3.60
1.46
10.35
8.5 8.0 7.5 7.0 6.5 6.0 5.5 5.0 4.5 4.0 3.5 3.0 2.5 2.0 1.5 1.0 0.5 0.0 -0.5 -1.0

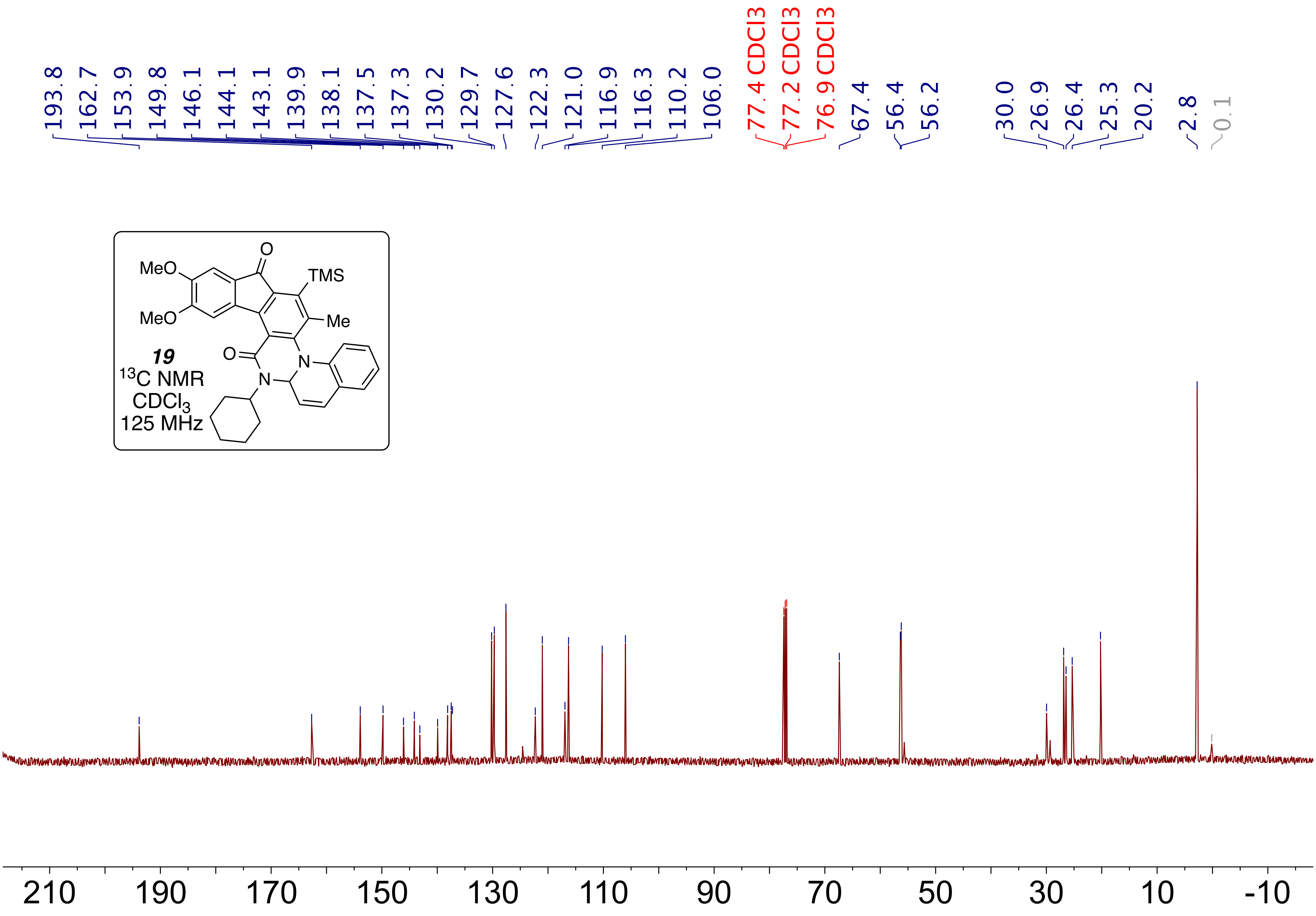
193.8
162.7
153.9
149.8
146.1
144.1
143.1
139.9
138.1
137.5
137.3
130.2
129.7
127.6
122.3
121.0
116.9
116.3
110.2
106.0
77.4 CDCl3
77.2 CDCl3
76.9 CDCl3
67.4
56.4
56.2
30.0
26.9
26.4
25.3
20.2
2.8
0.1
MeO
MeO
O
TMS
Me
O
N
N
19
^{13}C NMR
$CDCl_3$
125 MHz
210
190
170
150
130
110
90
70
50
30
10
-10

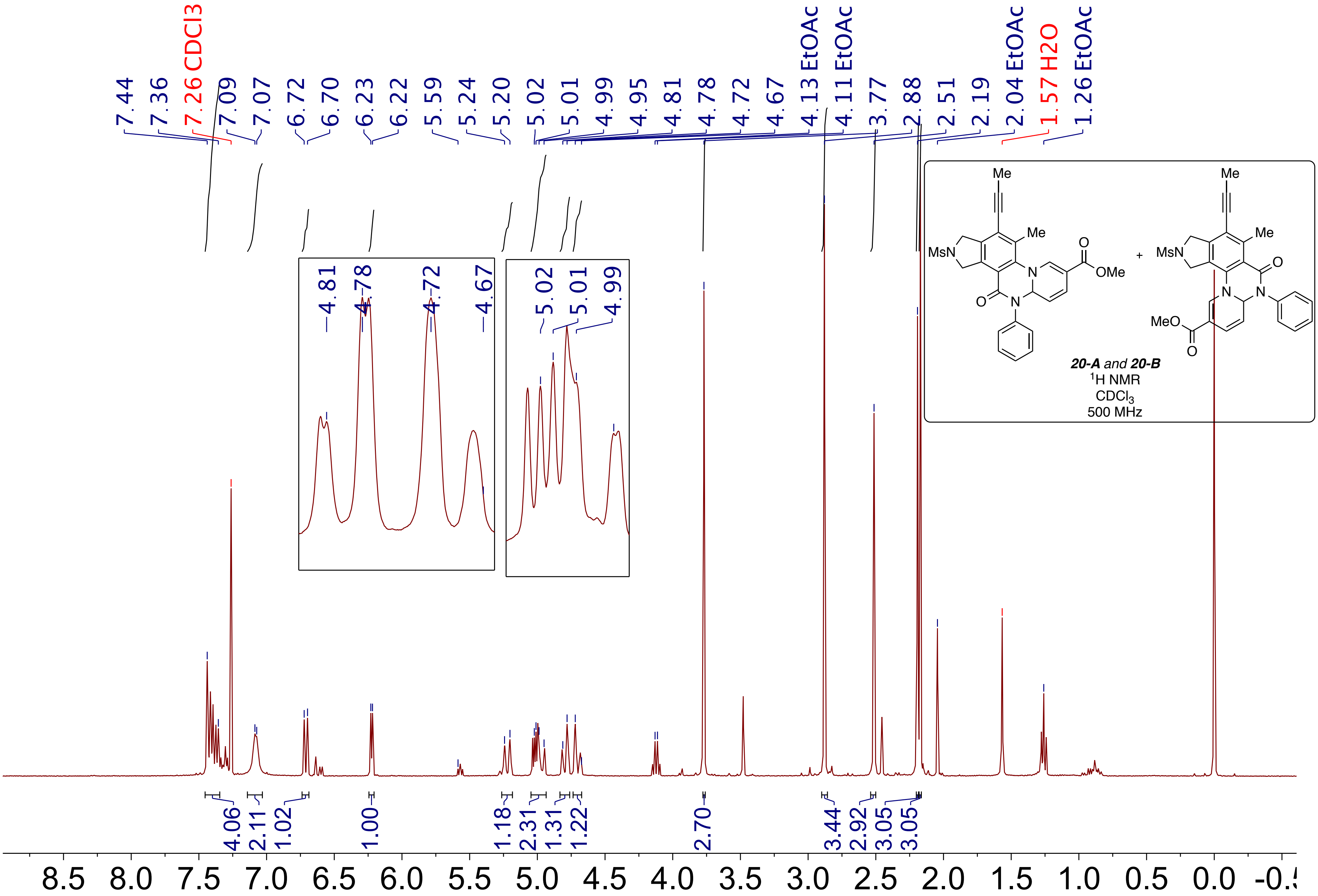
7.44
7.36
7.26 CDCl3
7.09
7.07
6.72
6.70
6.23
6.22
5.59
5.24
5.20
5.02
5.01
4.99
4.95
4.81
4.78
4.72
4.67
4.13 EtOAc
4.11 EtOAc
3.77
2.88
2.51
2.19
2.04 EtOAc
1.57 H2O
1.26 EtOAc
20-A and 20-B
1H NMR
CDCl3
500 MHz
4.06
2.11
1.02
1.00
1.18
2.31
1.31
1.22
2.70
3.44
2.92
3.05
3.05
8.5 8.0 7.5 7.0 6.5 6.0 5.5 5.0 4.5 4.0 3.5 3.0 2.5 2.0 1.5 1.0 0.5 0.0

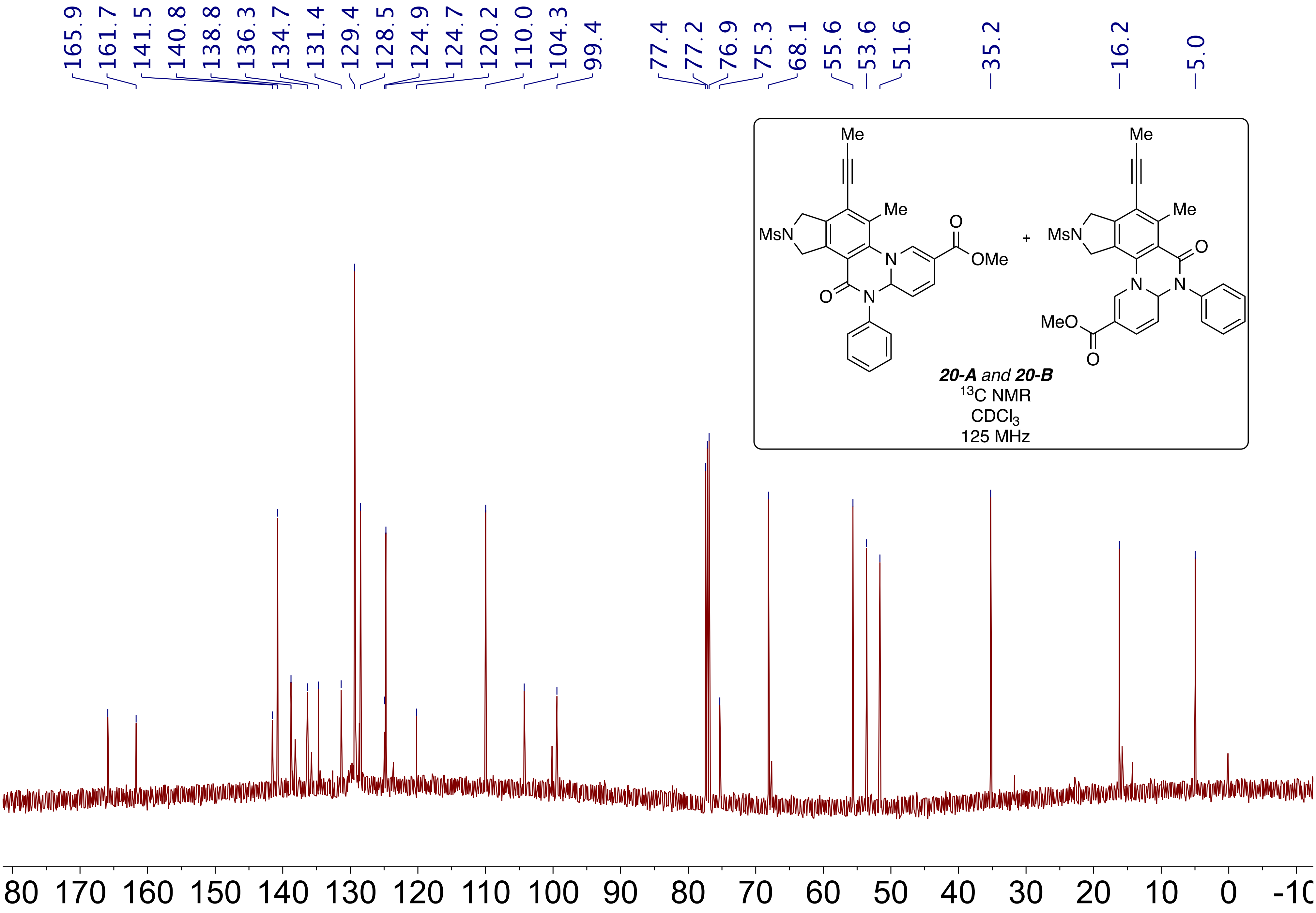
165.9
161.7
141.5
140.8
138.8
136.3
134.7
131.4
129.4
128.5
124.9
124.7
120.2
110.0
104.3
99.4
77.4
77.2
76.9
75.3
68.1
55.6
53.6
51.6
35.2
16.2
5.0
Me
Me
MsN
N
O
OMe
O
N
+
Me
Me
MsN
O
N
N
MeO
O
20-A and 20-B
^{13}C NMR
$CDCl_3$
125 MHz
180 170 160 150 140 130 120 110 100 90 80 70 60 50 40 30 20 10 0 -10

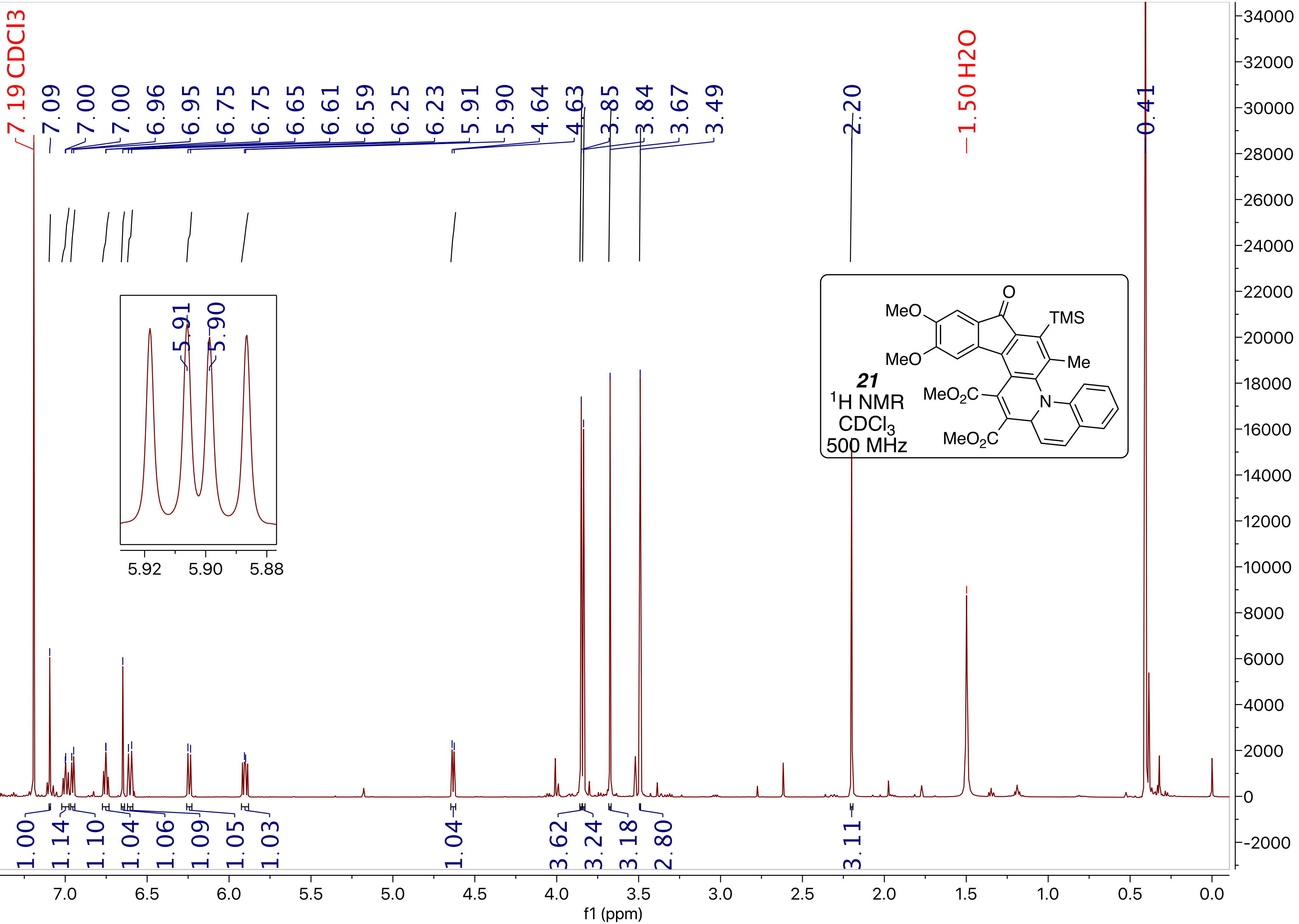
7.19 CDCl3
7.09
7.00
7.00
6.96
6.95
6.75
6.75
6.65
6.61
6.59
6.25
6.23
5.91
5.90
4.64
4.63
3.85
3.84
3.67
3.49
2.20
1.50 H2O
0.41
5.91
5.90
5.92
5.90
5.88
MeO
MeO
O
TMS
Me
MeO2C
N
MeO2C
21
1H NMR
CDCl3
500 MHz
1.00
1.14
1.10
1.04
1.06
1.09
1.05
1.03
1.04
3.62
3.24
3.18
2.80
3.11
f1 (ppm)

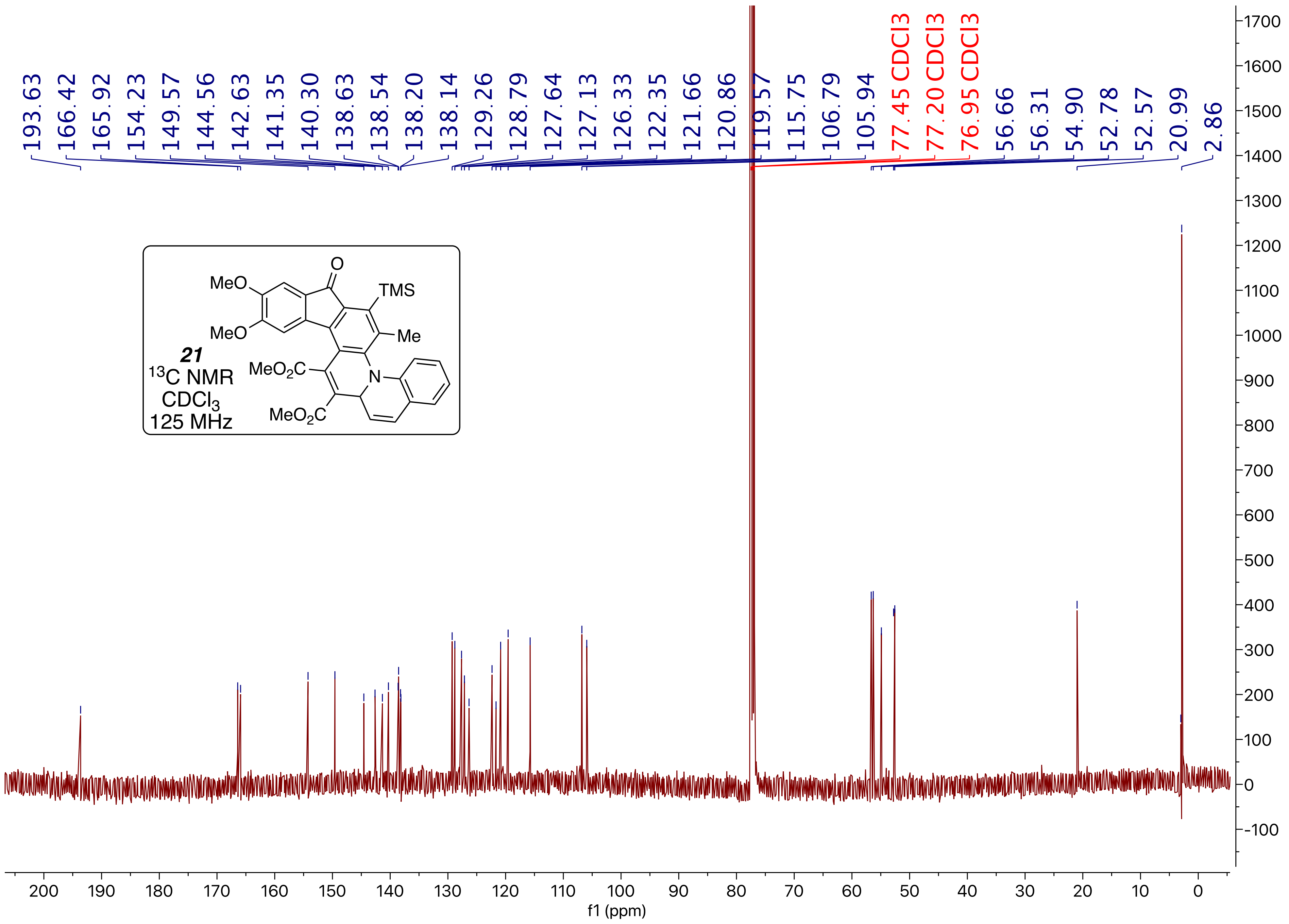
193.63
166.42
165.92
154.23
149.57
144.56
142.63
141.35
140.30
138.63
138.54
138.20
138.14
129.26
128.79
127.64
127.13
126.33
122.35
121.66
120.86
119.57
115.75
106.79
105.94
77.45 CDCl3
77.20 CDCl3
76.95 CDCl3
56.66
56.31
54.90
52.78
52.57
20.99
2.86
MeO
MeO
O
TMS
Me
MeO2C
MeO2C
N
21
13C NMR
CDCl3
125 MHz
f1 (ppm)

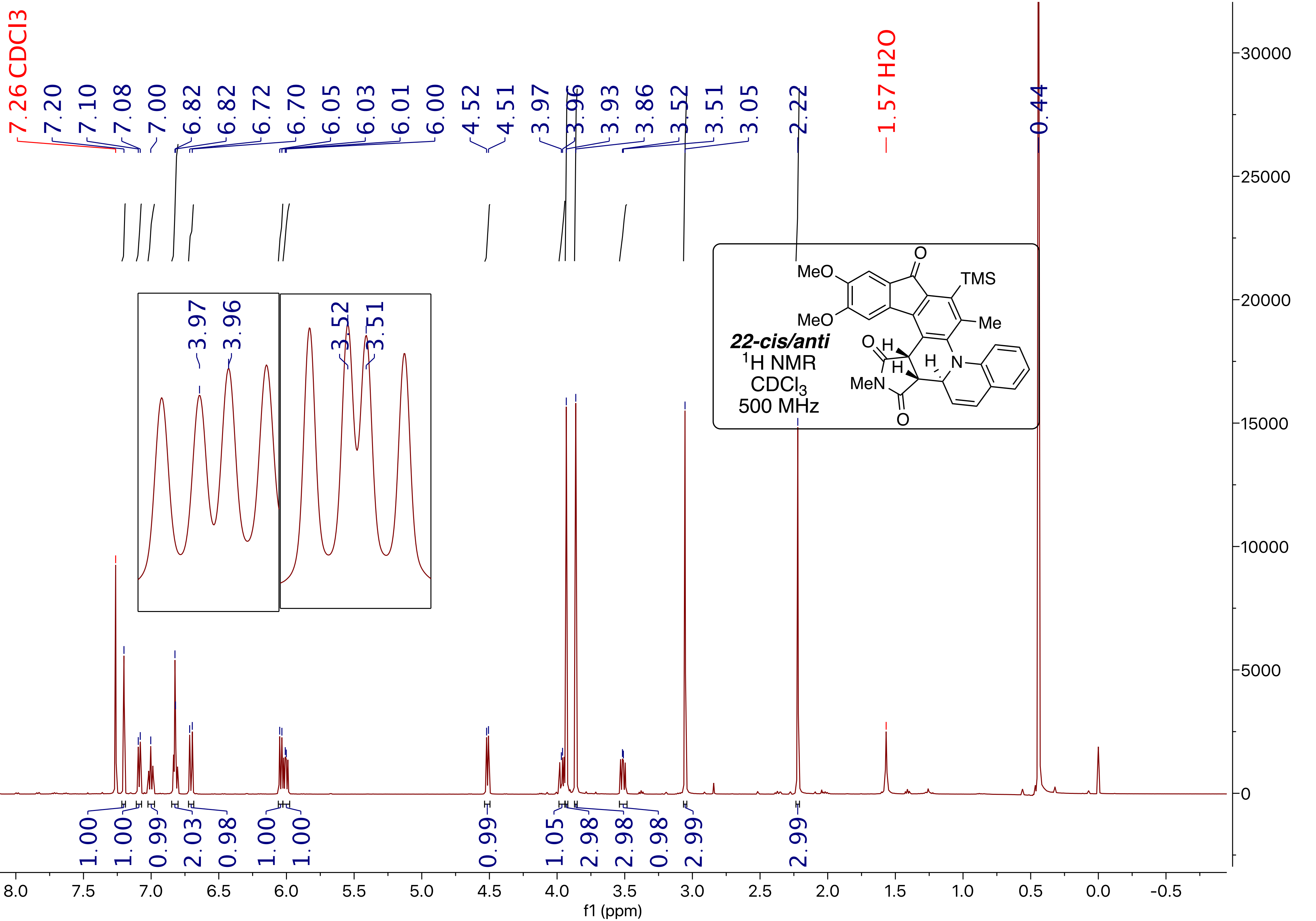
7.26 CDCl3
7.20 7.10 7.08 7.00 6.82 6.82 6.72 6.70 6.05 6.03 6.01 6.00 4.52 4.51 3.97 3.96 3.93 3.86 3.52 3.51 3.05 2.22
1.57 H2O
0.44
22-cis/anti
1H NMR
CDCl3
500 MHz
MeO
MeO
O
TMS
Me
N
MeN
1.00 1.00 0.99 2.03 0.98 1.00 1.00 0.99 1.05 2.98 2.98 0.98 2.99 2.99
f1 (ppm)

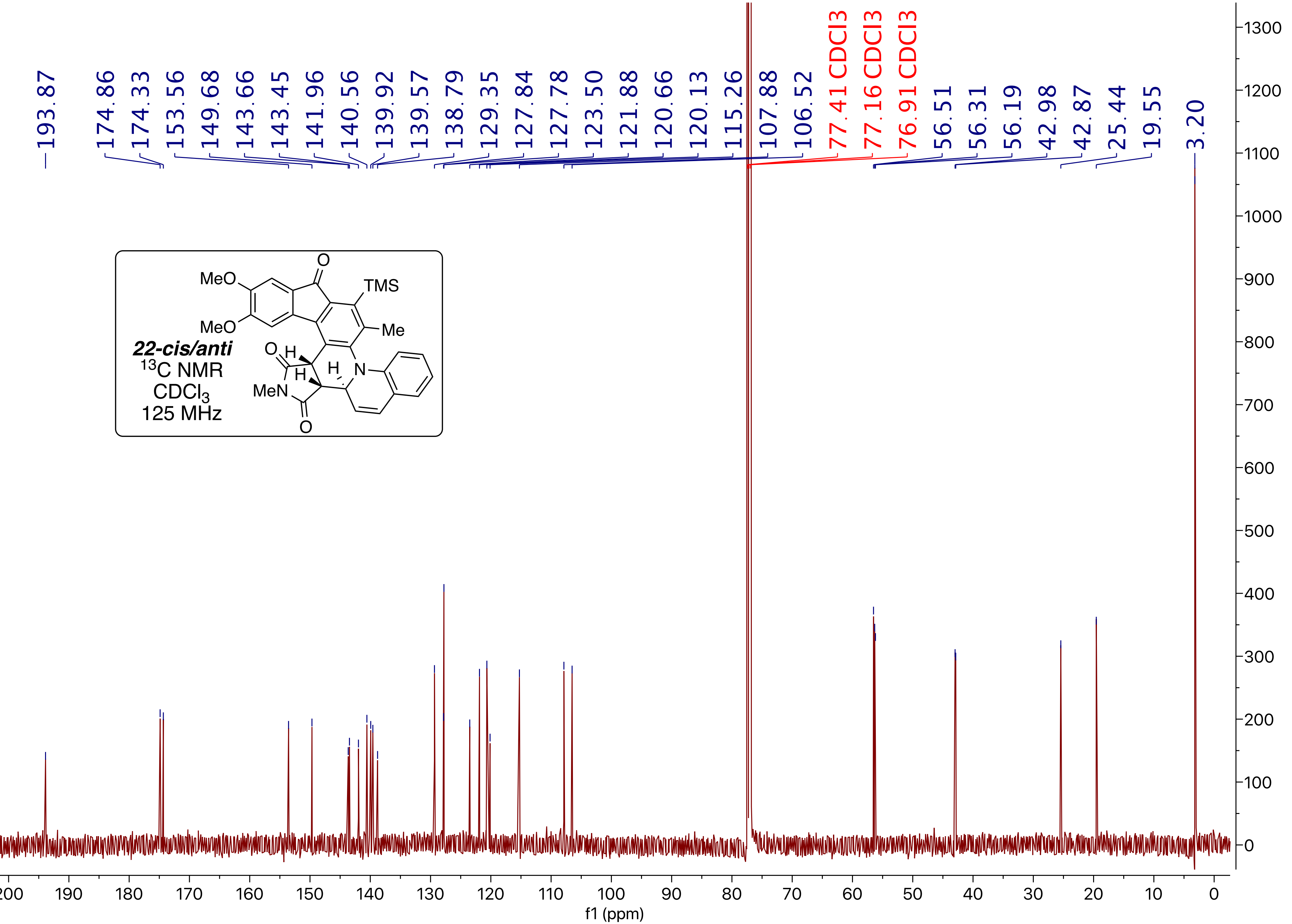
193.87
174.86
174.33
153.56
149.68
143.66
143.45
141.96
140.56
139.92
139.57
138.79
129.35
127.84
127.78
123.50
121.88
120.66
120.13
115.26
107.88
106.52
77.41 CDCl3
77.16 CDCl3
76.91 CDCl3
56.51
56.31
56.19
42.98
42.87
25.44
19.55
3.20
MeO
MeO
O
TMS
Me
N
H
H
H
O
MeN
O
22-cis/anti
13C NMR
CDCl3
125 MHz
f1 (ppm)

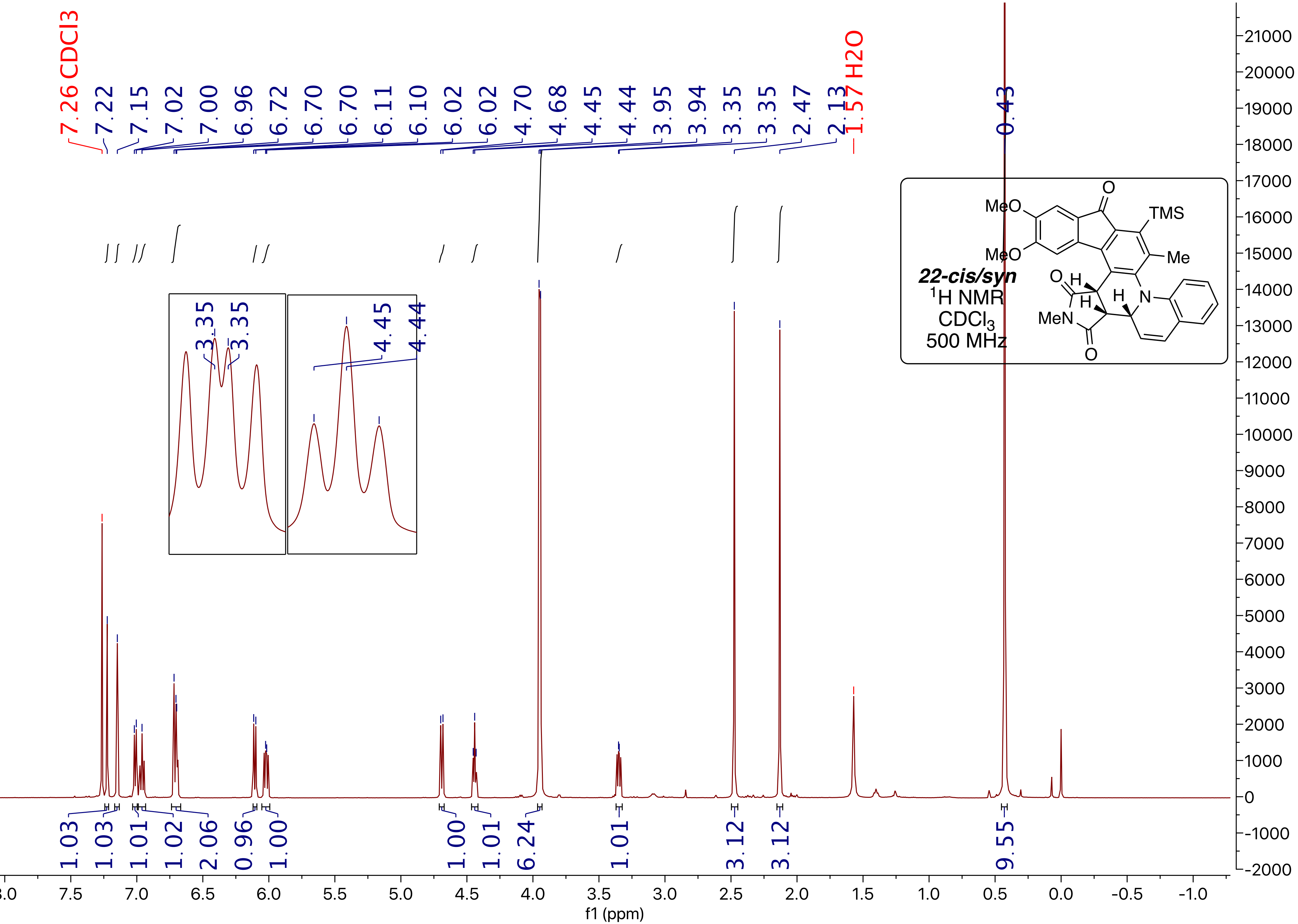
7.26 CDCl3
7.22
7.15
7.02
7.00
6.96
6.72
6.70
6.70
6.11
6.10
6.02
6.02
4.70
4.68
4.45
4.44
3.95
3.94
3.35
3.35
2.47
2.13
1.57 H2O
0.43
3.35
3.35
4.45
4.44
MeO
MeO
O
TMS
Me
O
H
H
H
N
MeN
O
22-cis/syn
1H NMR
CDCl3
500 MHz
1.03
1.03
1.01
1.02
2.06
0.96
1.00
1.00
1.01
6.24
1.01
3.12
3.12
9.55
f1 (ppm)

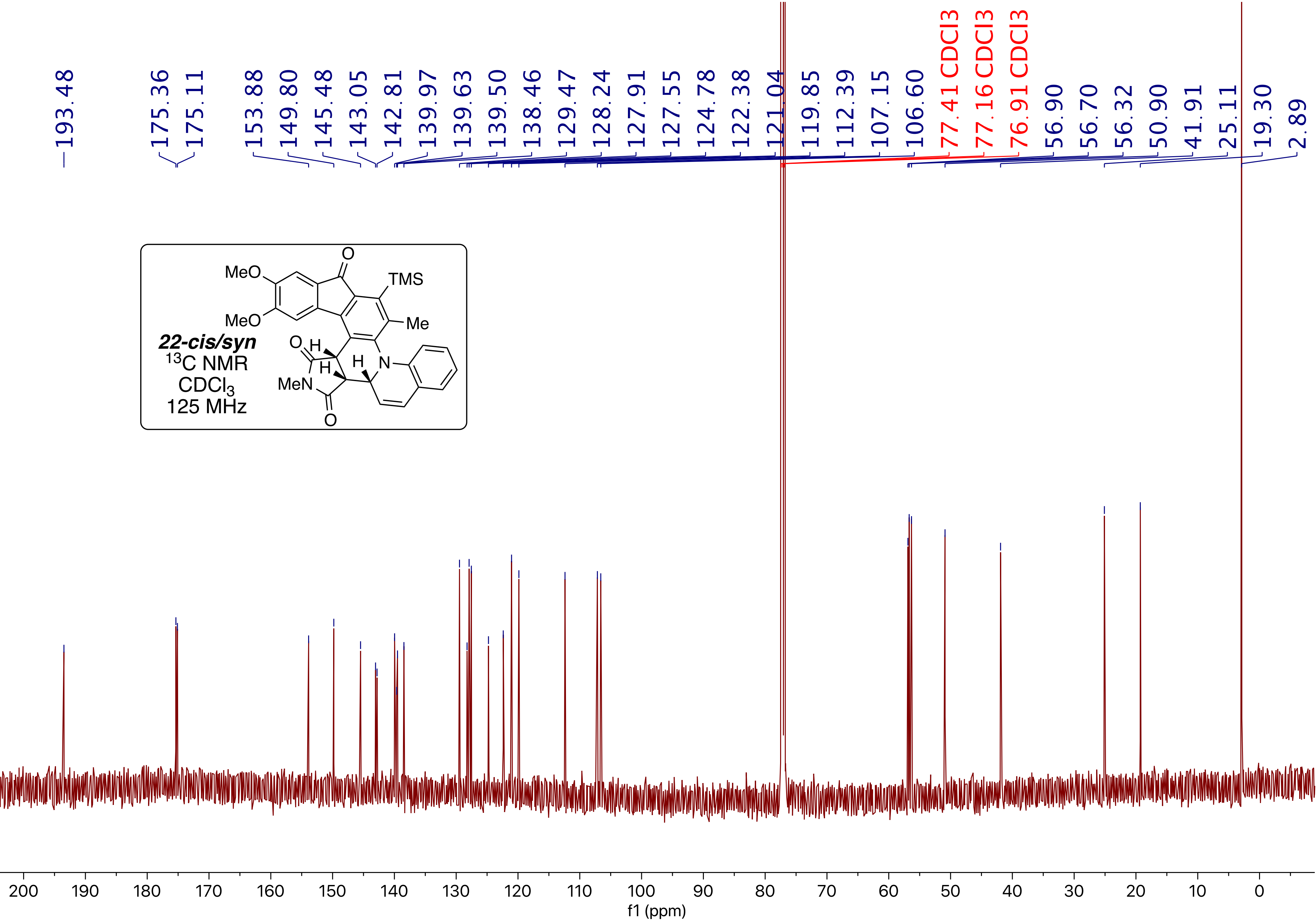
193.48
175.36
175.11
153.88
149.80
145.48
143.05
142.81
139.97
139.63
139.50
138.46
129.47
128.24
127.91
127.55
124.78
122.38
121.04
119.85
112.39
107.15
106.60
77.41 CDCl3
77.16 CDCl3
76.91 CDCl3
56.90
56.70
56.32
50.90
41.91
25.11
19.30
2.89
MeO
MeO
O
TMS
Me
O
H
H
H
N
MeN
O
22-cis/syn
13C NMR
CDCl3
125 MHz
f1 (ppm)

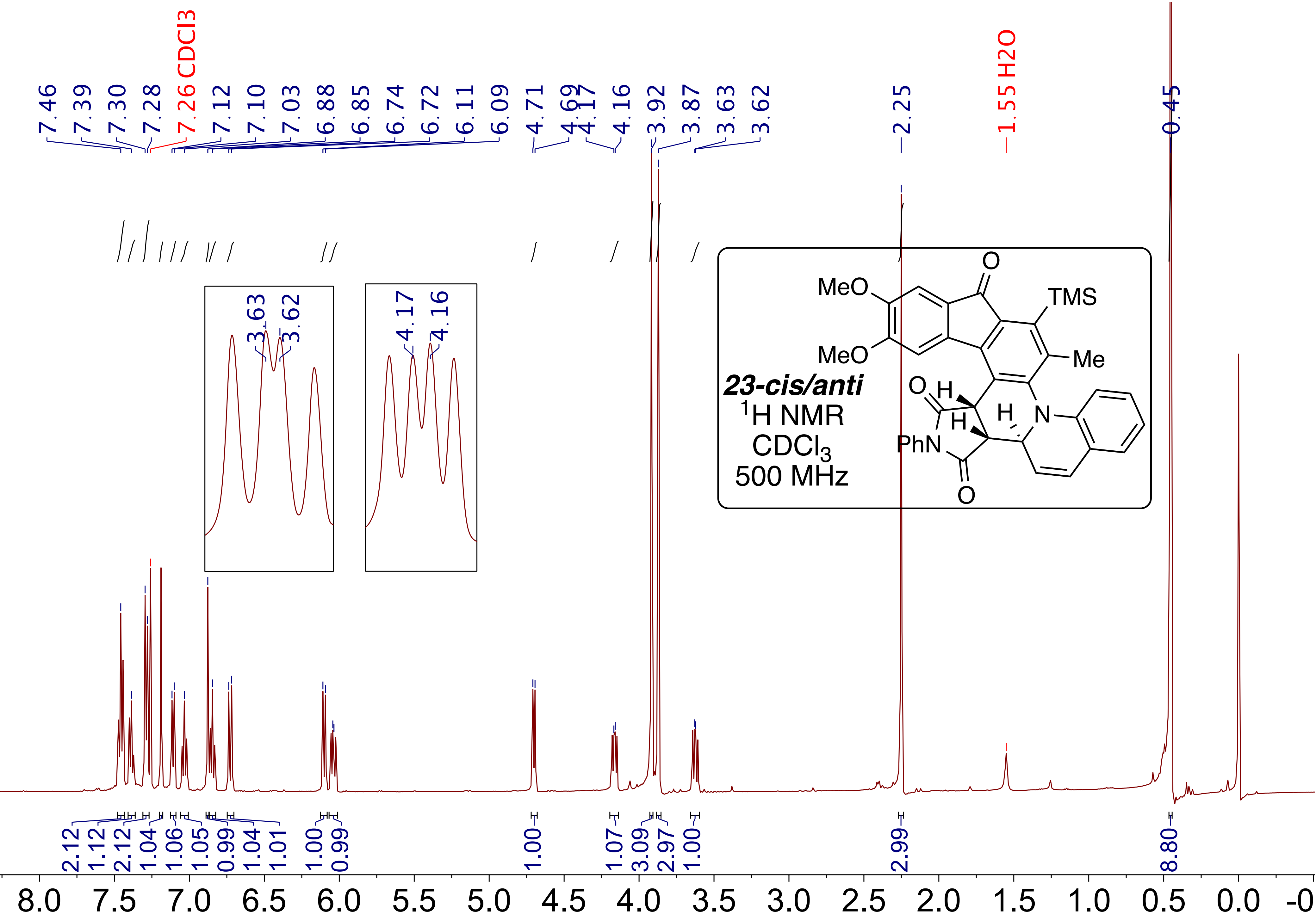
7.46 7.39 7.30 7.28 7.26 CDCl3 7.12 7.10 7.03 6.88 6.85 6.74 6.72 6.11 6.09 4.71 4.69 4.17 4.16 3.92 3.87 3.63 3.62
2.25
1.55 H2O
0.45
3.63 3.62
4.17 4.16
MeO
MeO
O
TMS
Me
O
H
H
H
N
PhN
O
23-cis/anti
¹H NMR
CDCl3
500 MHz
2.12 1.12 2.12 1.04 1.06 1.05 0.99 1.04 1.01 1.00 0.99 1.00 1.07 3.09 2.97 1.00 2.99 8.80
8.0 7.5 7.0 6.5 6.0 5.5 5.0 4.5 4.0 3.5 3.0 2.5 2.0 1.5 1.0 0.5 0.0 -0

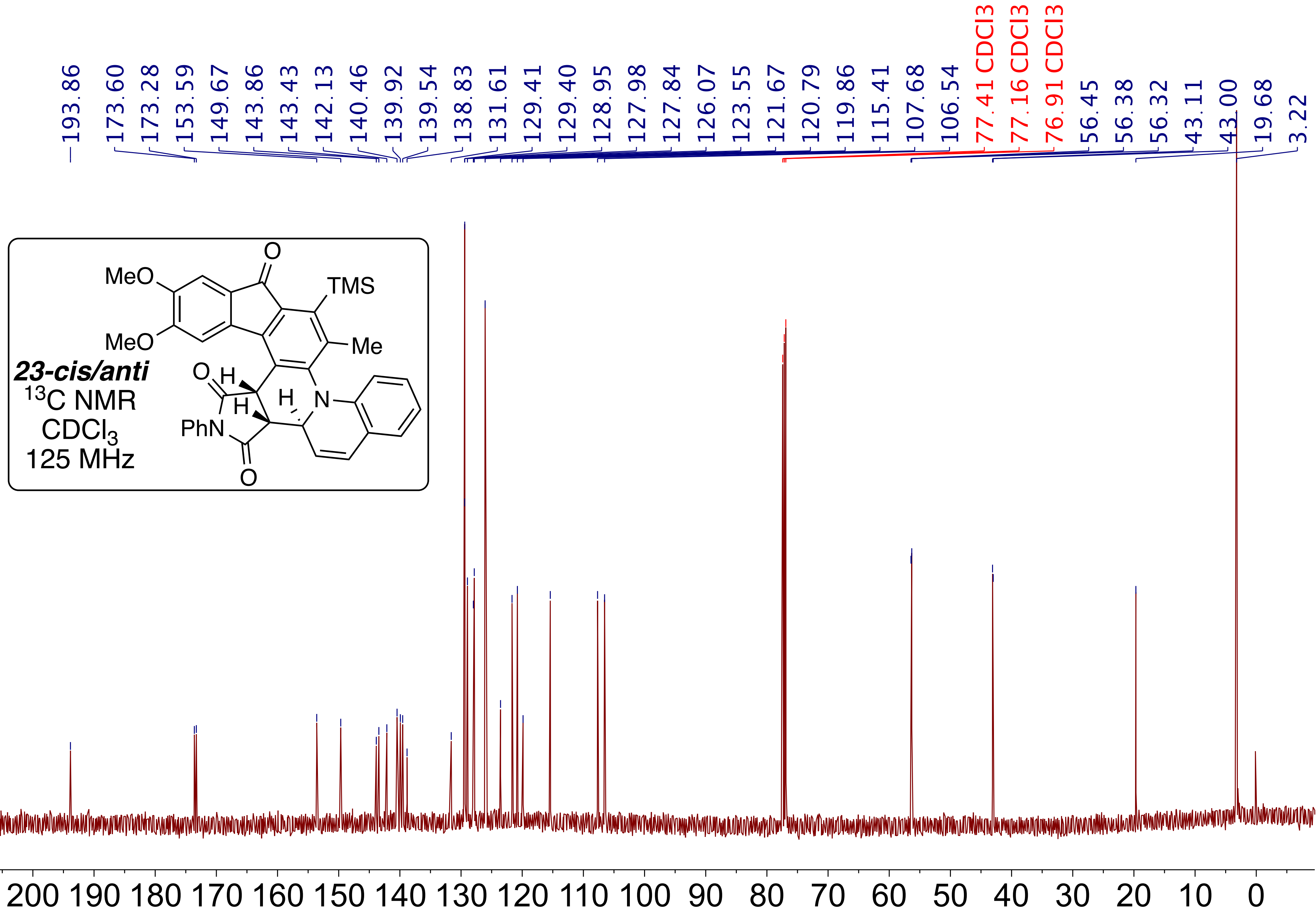
193.86
173.60
173.28
153.59
149.67
143.86
143.43
142.13
140.46
139.92
139.54
138.83
131.61
129.41
129.40
128.95
127.98
127.84
126.07
123.55
121.67
120.79
119.86
115.41
107.68
106.54
77.41 CDCl3
77.16 CDCl3
76.91 CDCl3
56.45
56.38
56.32
43.11
43.00
19.68
3.22
MeO
MeO
O
TMS
Me
O
H
H
H
N
PhN
O
23-cis/anti
13C NMR
CDCl3
125 MHz
200 190 180 170 160 150 140 130 120 110 100 90 80 70 60 50 40 30 20 10 0

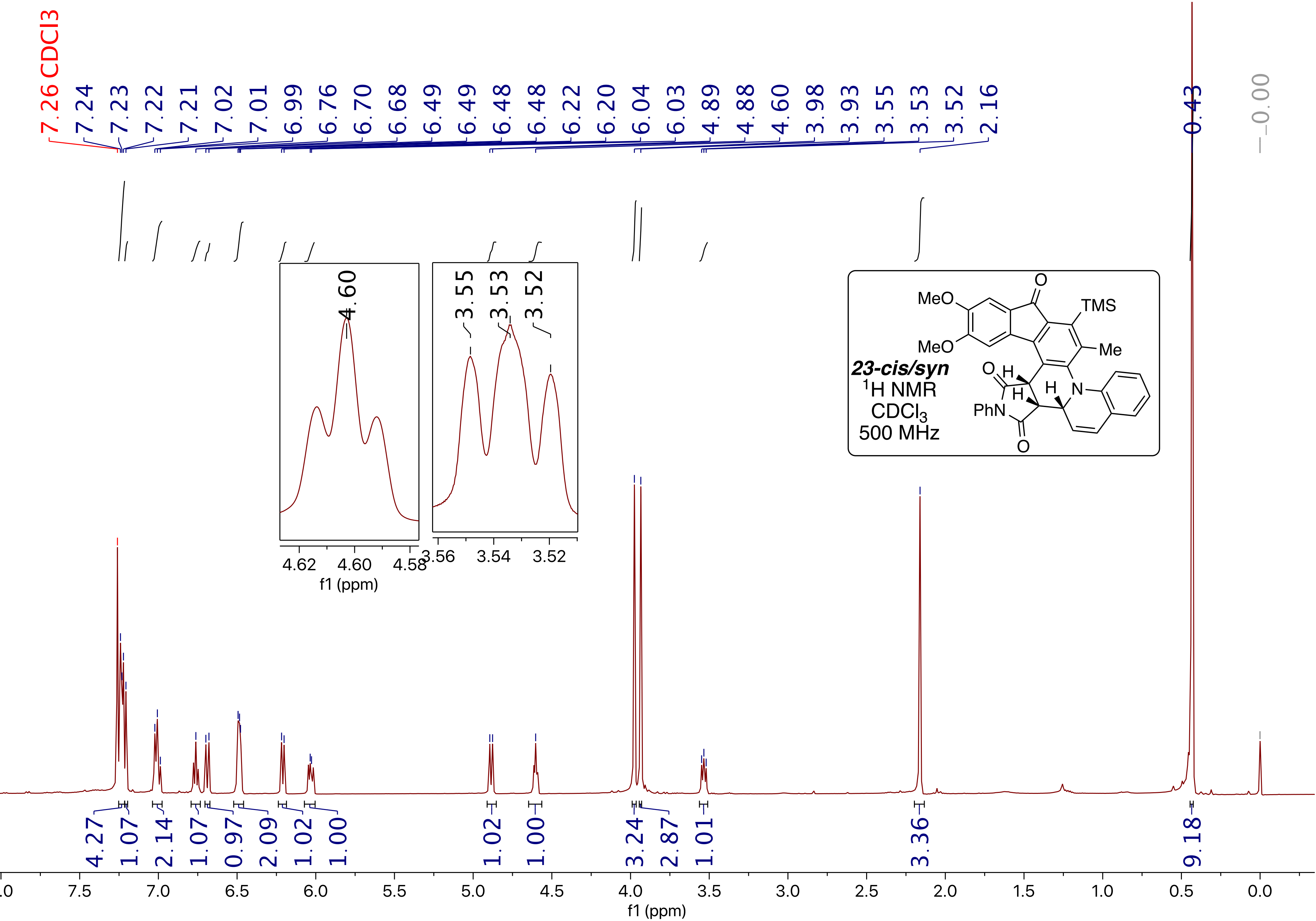
7.26 CDCl3
7.24 7.23 7.22 7.21 7.02 7.01 6.99 6.76 6.70 6.68 6.49 6.49 6.48 6.48 6.22 6.20 6.04 6.03 4.89 4.88 4.60 3.98 3.93 3.55 3.53 3.52 2.16
0.43
0.00
4.60
3.55 3.53 3.52
4.62 4.60 4.58
3.56 3.54 3.52
f1 (ppm)
MeO
MeO
O
TMS
Me
O
H
H
H
N
PhN
O
23-cis/syn
^{1}H NMR
$CDCl_3$
500 MHz
4.27 1.07 2.14 1.07 0.97 2.09 1.02 1.00 1.02 1.00 3.24 2.87 1.01 3.36 9.18
8.0 7.5 7.0 6.5 6.0 5.5 5.0 4.5 4.0 3.5 3.0 2.5 2.0 1.5 1.0 0.5 0.0
f1 (ppm)

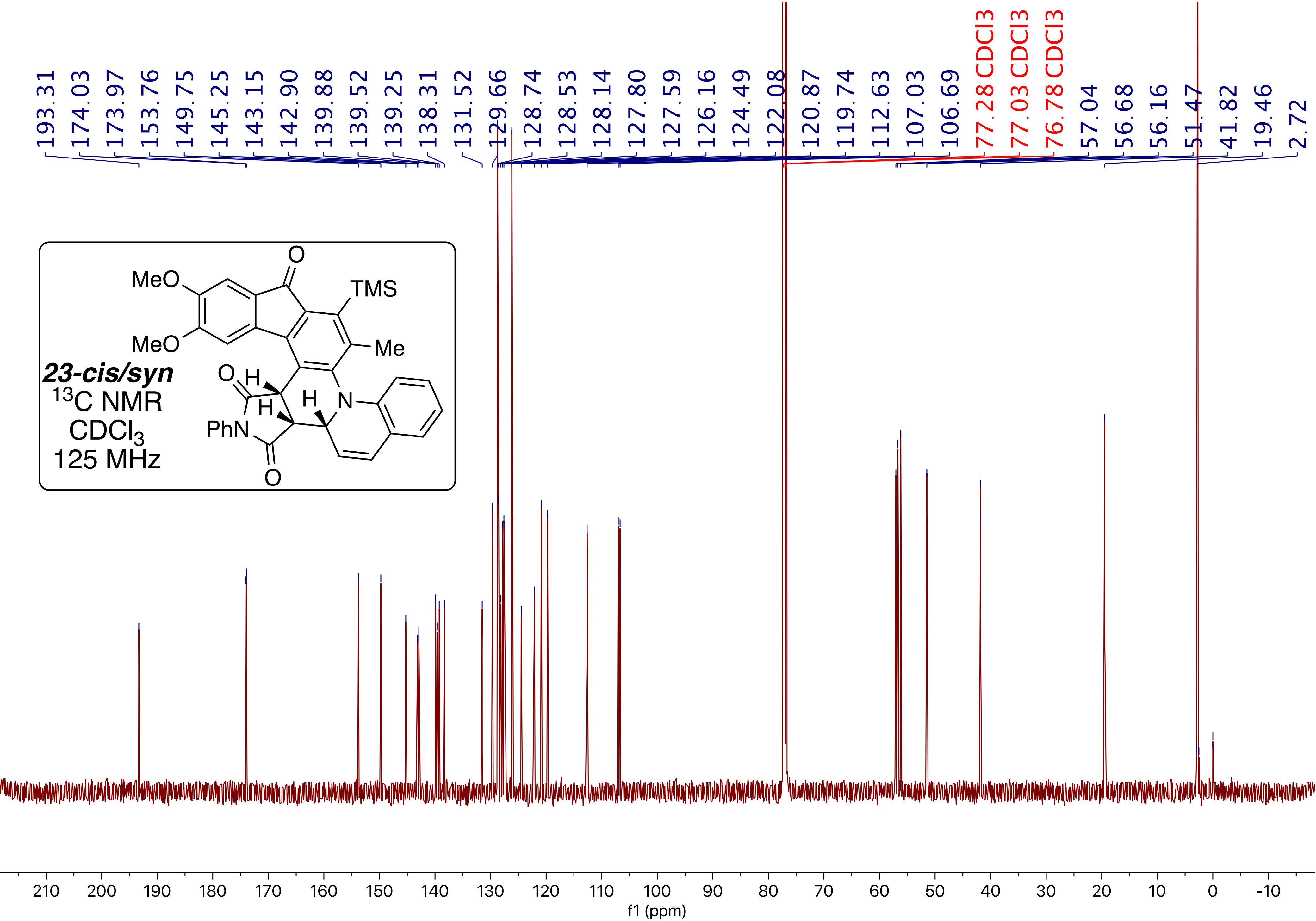
193.31
174.03
173.97
153.76
149.75
145.25
143.15
142.90
139.88
139.52
139.25
138.31
131.52
129.66
128.74
128.53
128.14
127.80
127.59
126.16
124.49
122.08
120.87
119.74
112.63
107.03
106.69
77.28 CDCl3
77.03 CDCl3
76.78 CDCl3
57.04
56.68
56.16
51.47
41.82
19.46
2.72
MeO
MeO
O
TMS
Me
O
H
H
H
N
PhN
O
23-cis/syn
13C NMR
CDCl3
125 MHz
210 200 190 180 170 160 150 140 130 120 110 100 90 80 70 60 50 40 30 20 10 0 -10
f1 (ppm)

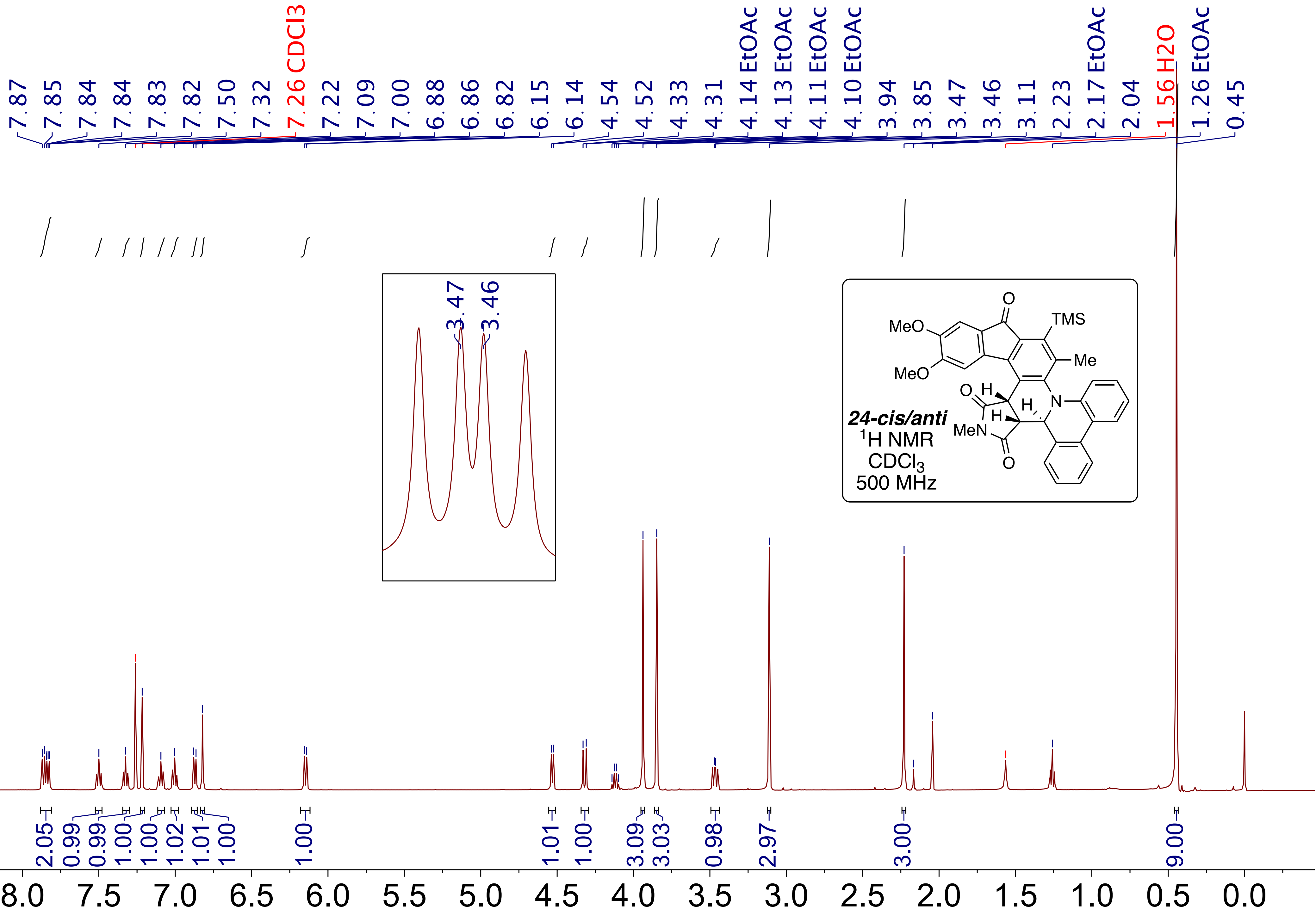
24-cis/anti
1H NMR
CDCl3
500 MHz
MeO
MeO
O
TMS
Me
N
MeN
7.26 CDCl3
1.56 H2O
EtOAc
2.05
0.99
0.99
1.00
1.00
1.02
1.01
1.00
1.00
1.01
1.00
3.09
3.03
0.98
2.97
3.00
9.00
3.47
3.46
8.0 7.5 7.0 6.5 6.0 5.5 5.0 4.5 4.0 3.5 3.0 2.5 2.0 1.5 1.0 0.5 0.0

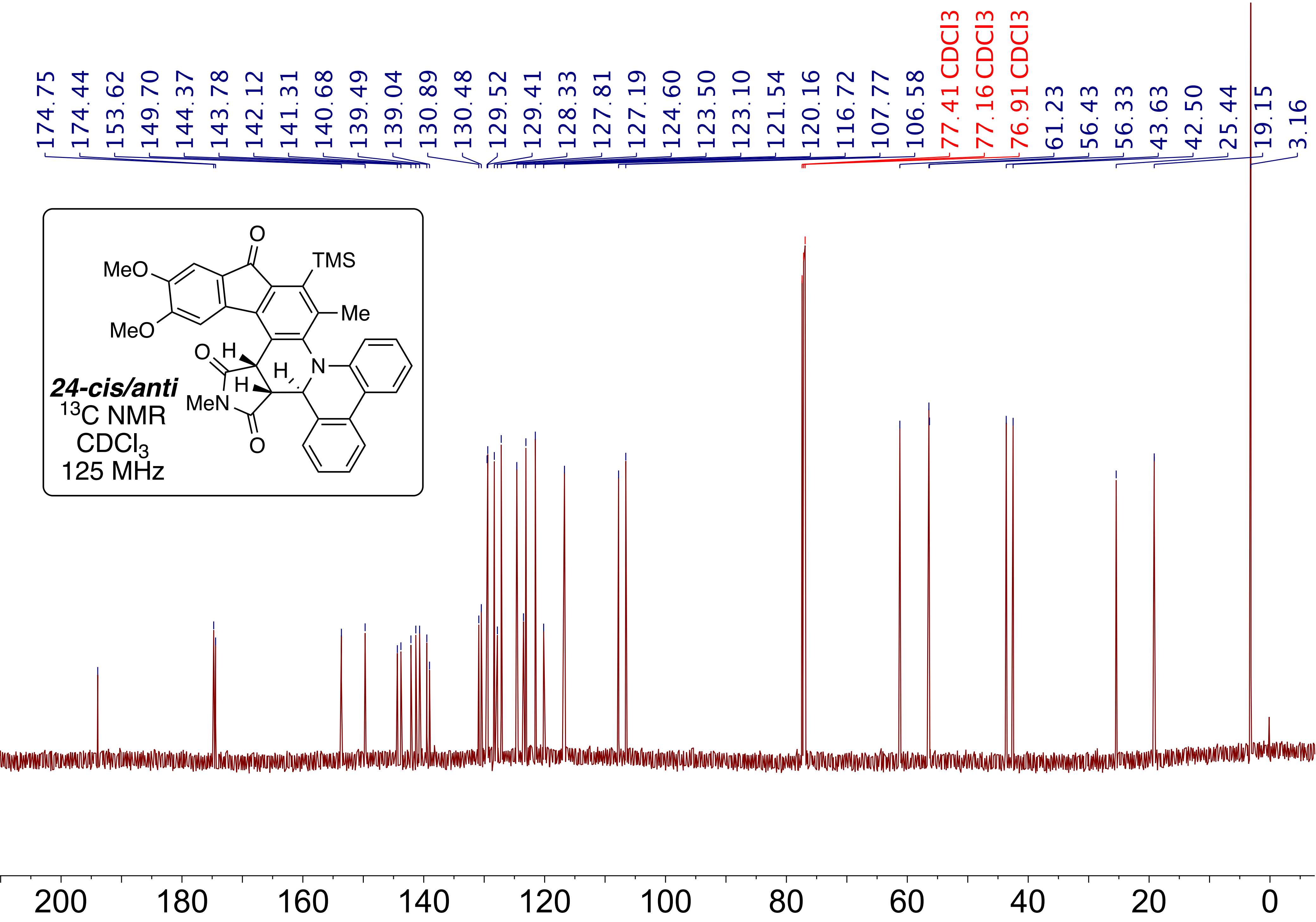
174.75
174.44
153.62
149.70
144.37
143.78
142.12
141.31
140.68
139.49
139.04
130.89
130.48
129.52
129.41
128.33
127.81
127.19
124.60
123.50
123.10
121.54
120.16
116.72
107.77
106.58
77.41 CDCl3
77.16 CDCl3
76.91 CDCl3
61.23
56.43
56.33
43.63
42.50
25.44
19.15
3.16
MeO
MeO
O
TMS
Me
N
H
H
H
O
O
MeN
24-cis/anti
13C NMR
CDCl3
125 MHz
200
180
160
140
120
100
80
60
40
20
0

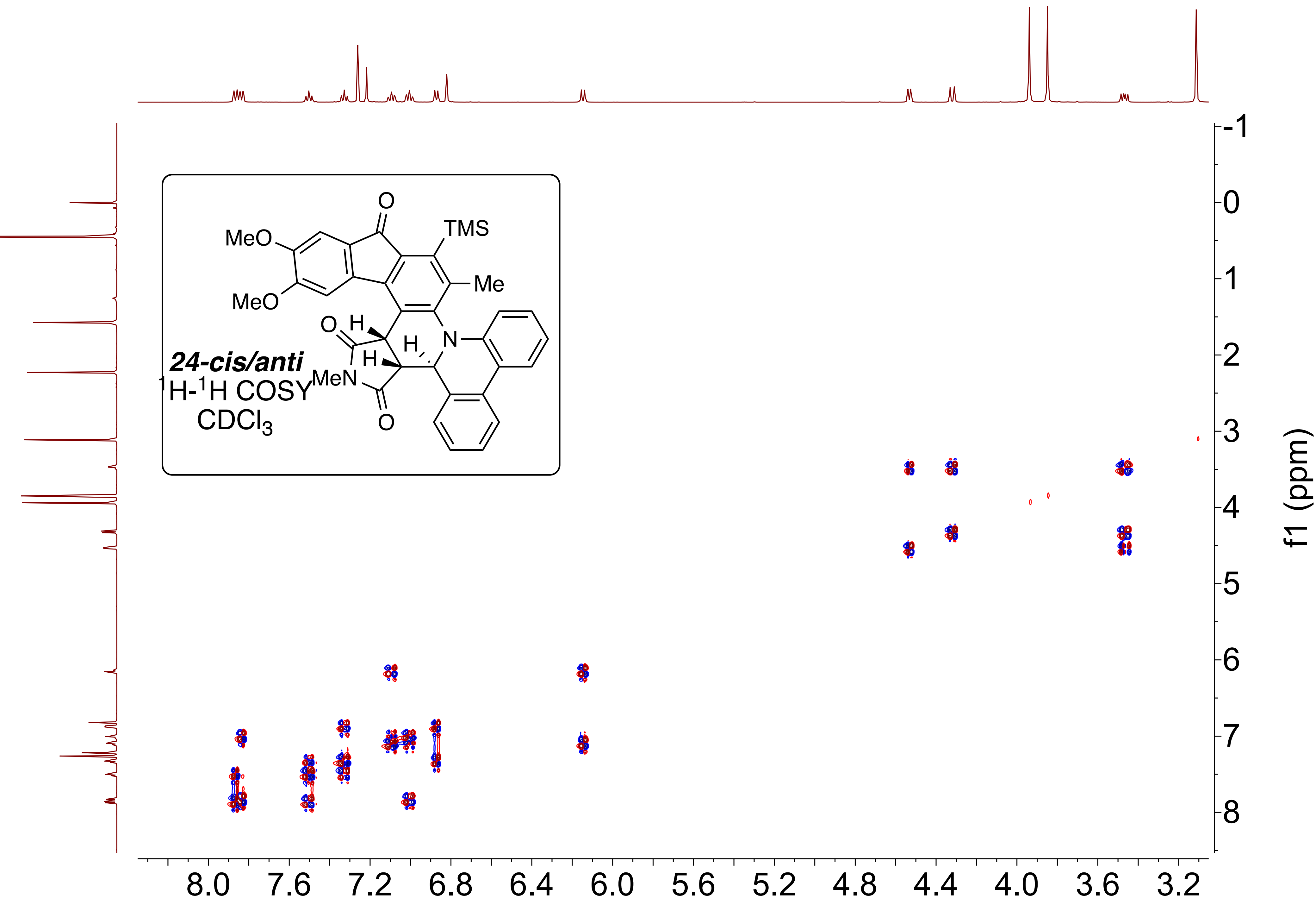

24-cis/anti
^{1}H-^{1}H COSY
$CDCl_3$
MeO
MeO
O
TMS
Me
N
H
H
H
O
MeN
O
f1 (ppm)
-1
0
1
2
3
4
5
6
7
8
8.0
7.6
7.2
6.8
6.4
6.0
5.6
5.2
4.8
4.4
4.0
3.6
3.2

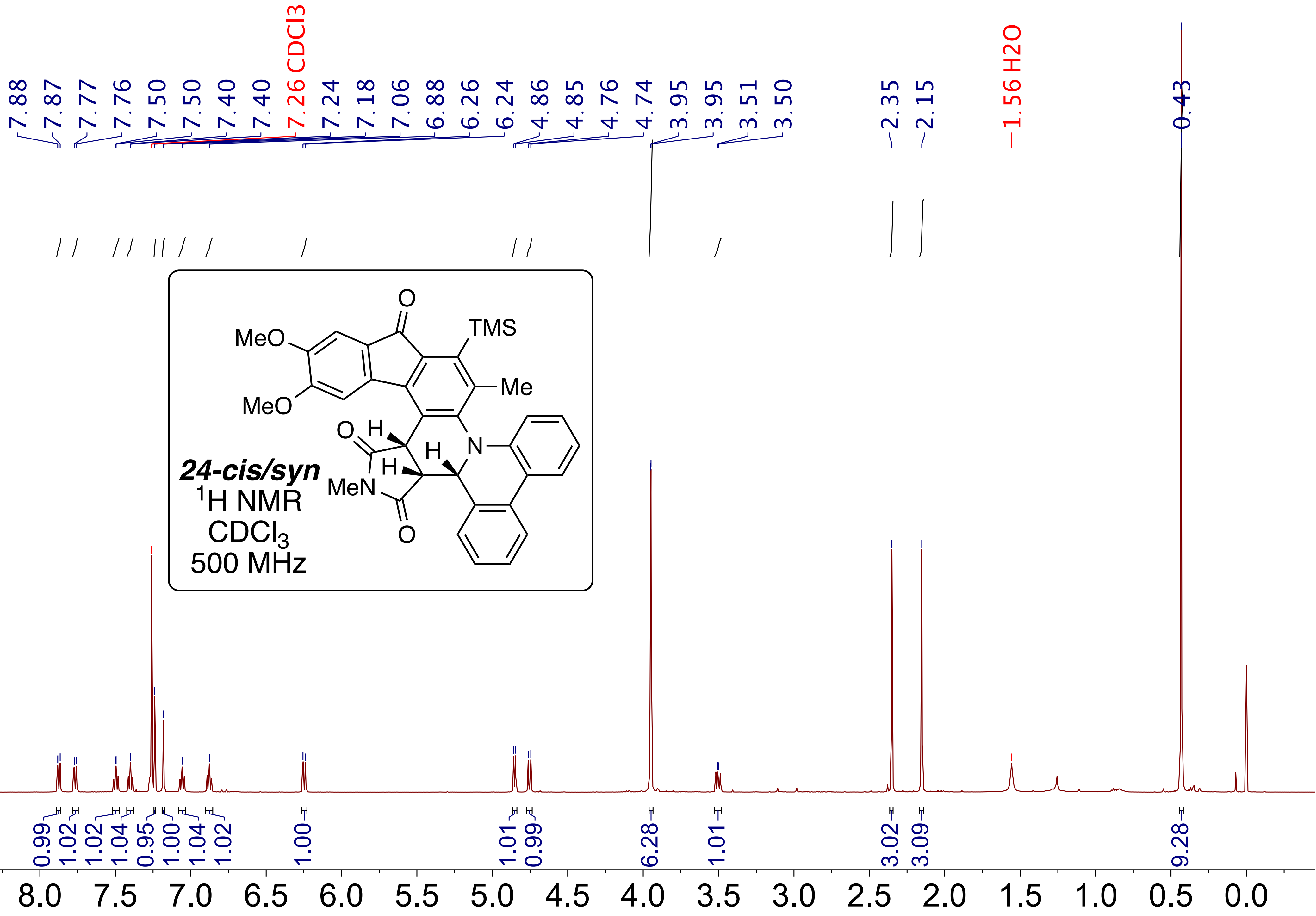

7.88 7.87 7.77 7.76 7.50 7.50 7.40 7.40 7.26 CDCl3 7.24 7.18 7.06 6.88 6.26 6.24 4.86 4.85 4.76 4.74 3.95 3.95 3.51 3.50 2.35 2.15 1.56 H2O 0.43
MeO
MeO
O
TMS
Me
N
O
H
H
H
MeN
O
24-cis/syn
^{1}H NMR
$CDCl_3$
500 MHz
0.99 1.02 1.02 1.04 1.04 0.95 1.00 1.04 1.02 1.00 1.01 0.99 6.28 1.01 3.02 3.09 9.28
8.0 7.5 7.0 6.5 6.0 5.5 5.0 4.5 4.0 3.5 3.0 2.5 2.0 1.5 1.0 0.5 0.0

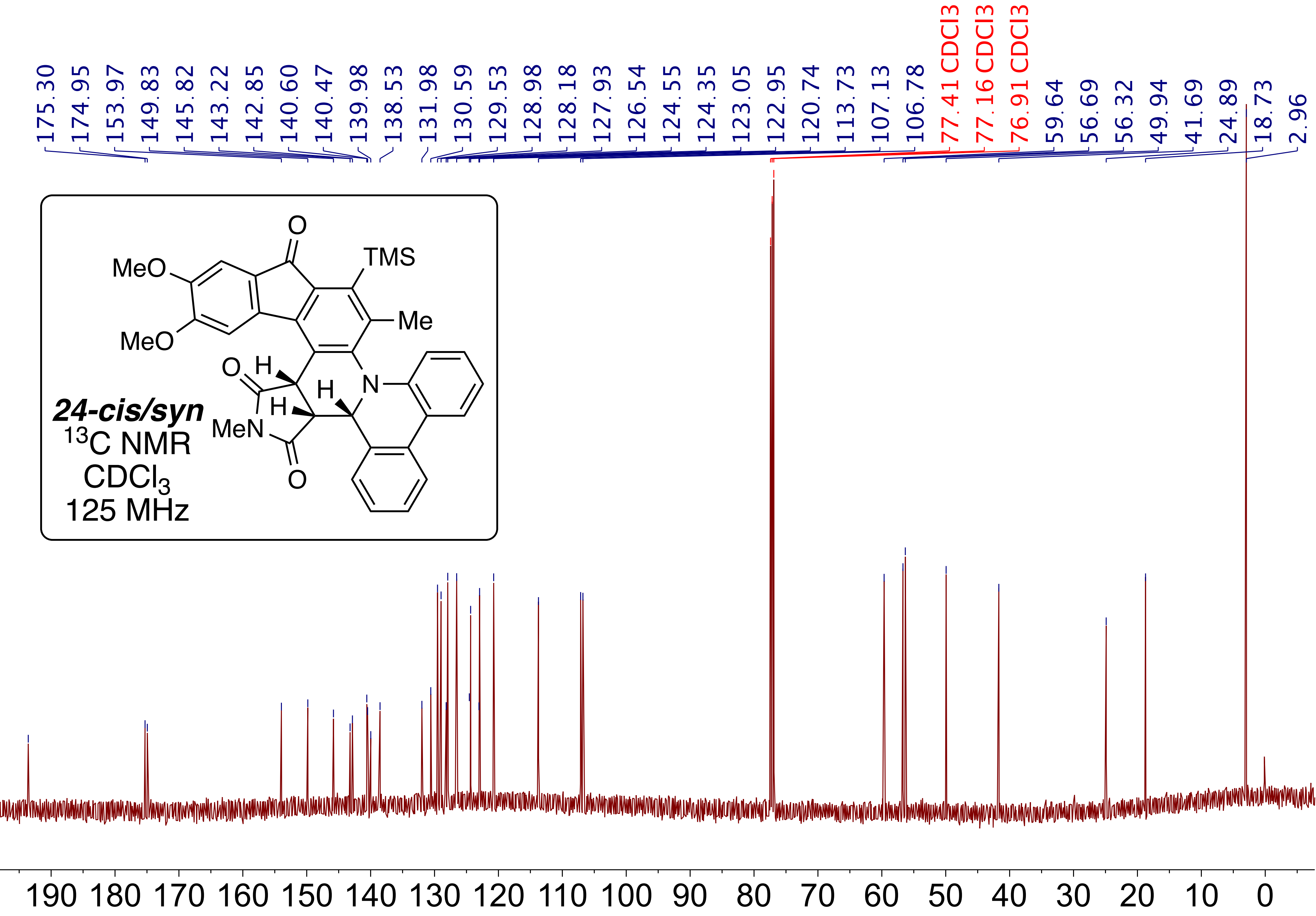
175.30
174.95
153.97
149.83
145.82
143.22
142.85
140.60
140.47
139.98
138.53
131.98
130.59
129.53
128.98
128.18
127.93
126.54
124.55
124.35
123.05
122.95
120.74
113.73
107.13
106.78
77.41 CDCl3
77.16 CDCl3
76.91 CDCl3
59.64
56.69
56.32
49.94
41.69
24.89
18.73
2.96
24-cis/syn
13C NMR
CDCl3
125 MHz
MeO
MeO
O
TMS
Me
N
MeN
H
H
H
O
O
190 180 170 160 150 140 130 120 110 100 90 80 70 60 50 40 30 20 10 0

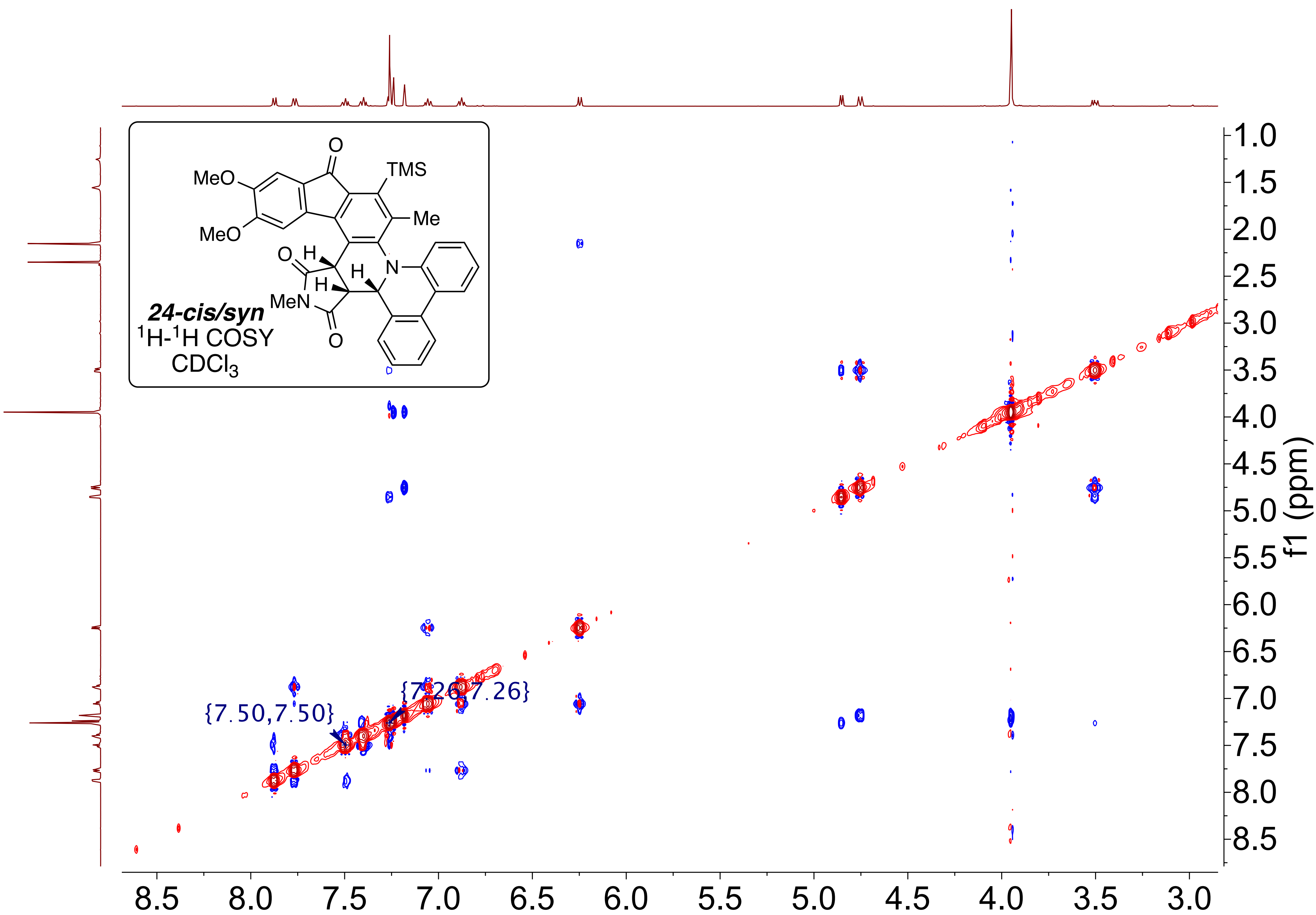
MeO
MeO
O
TMS
Me
O
H
H
H
N
MeN
O
24-cis/syn
^{1}H-^{1}H COSY
$CDCl_3$
{7.50,7.50}
{7.26,7.26}
f1 (ppm)
8.5
8.0
7.5
7.0
6.5
6.0
5.5
5.0
4.5
4.0
3.5
3.0
2.5
2.0
1.5
1.0

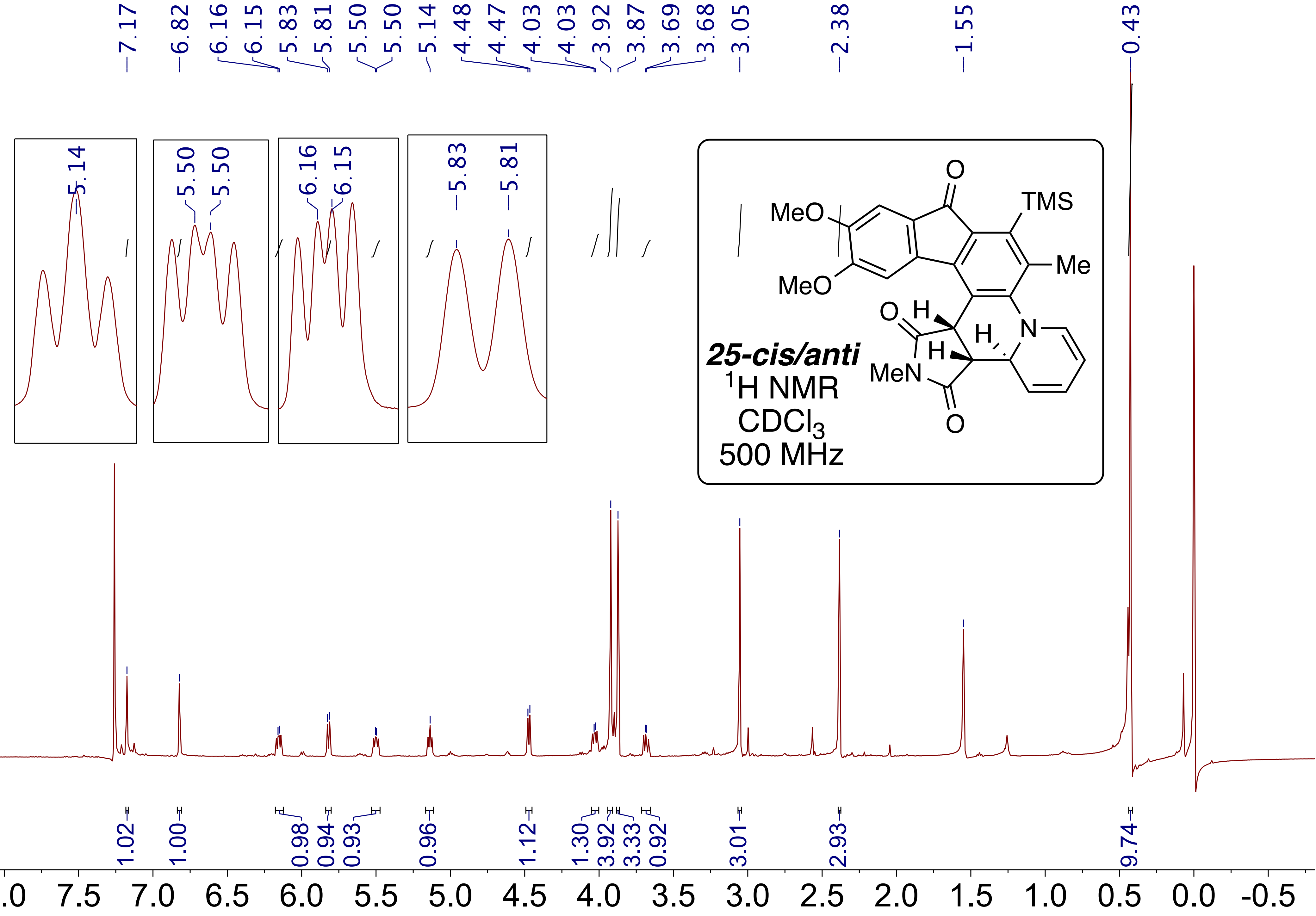
7.17
6.82
6.16
6.15
5.83
5.81
5.50
5.50
5.14
4.48
4.47
4.03
4.03
3.92
3.87
3.69
3.68
3.05
2.38
1.55
0.43
25-cis/anti
1H NMR
CDCl3
500 MHz
O
TMS
MeO
MeO
Me
O
H
H
N
H
MeN
O
1.02
1.00
0.98
0.94
0.93
0.96
1.12
1.30
3.92
3.33
0.92
3.01
2.93
9.74

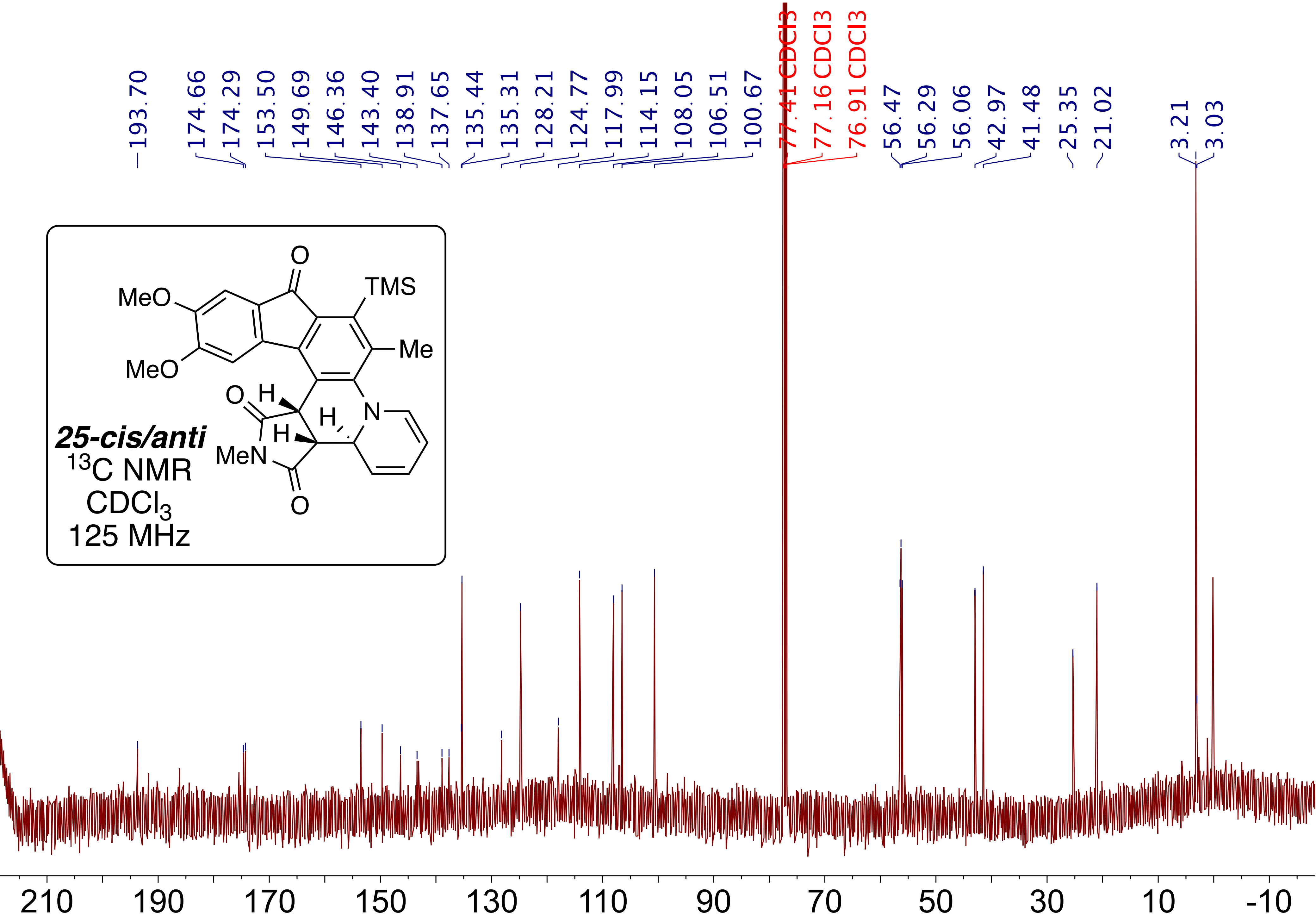
193.70
174.66
174.29
153.50
149.69
146.36
143.40
138.91
137.65
135.44
135.31
128.21
124.77
117.99
114.15
108.05
106.51
100.67
77.41 CDCl3
77.16 CDCl3
76.91 CDCl3
56.47
56.29
56.06
42.97
41.48
25.35
21.02
3.21
3.03
MeO
MeO
O
TMS
Me
N
O
H
H
H
MeN
O
25-cis/anti
^{13}C NMR
$CDCl_3$
125 MHz
210
190
170
150
130
110
90
70
50
30
10
-10

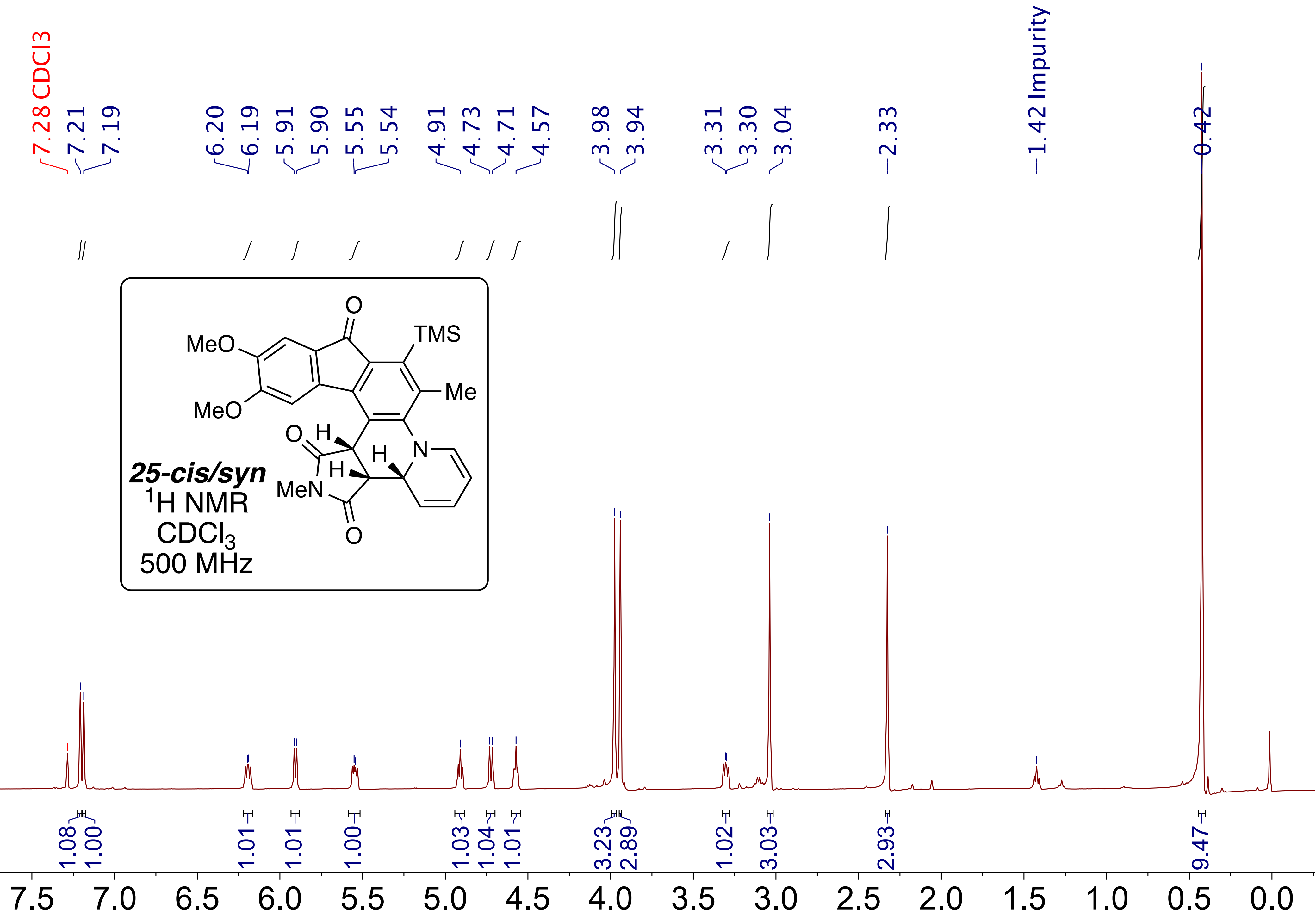
7.28 CDCl3
7.21
7.19
6.20
6.19
5.91
5.90
5.55
5.54
4.91
4.73
4.71
4.57
3.98
3.94
3.31
3.30
3.04
2.33
1.42 Impurity
0.42
O
TMS
MeO
Me
MeO
O
H
H
N
H
MeN
O
25-cis/syn
1H NMR
CDCl3
500 MHz
1.08
1.00
1.01
1.01
1.00
1.03
1.04
1.01
3.23
2.89
1.02
3.03
2.93
9.47
7.5
7.0
6.5
6.0
5.5
5.0
4.5
4.0
3.5
3.0
2.5
2.0
1.5
1.0
0.5
0.0

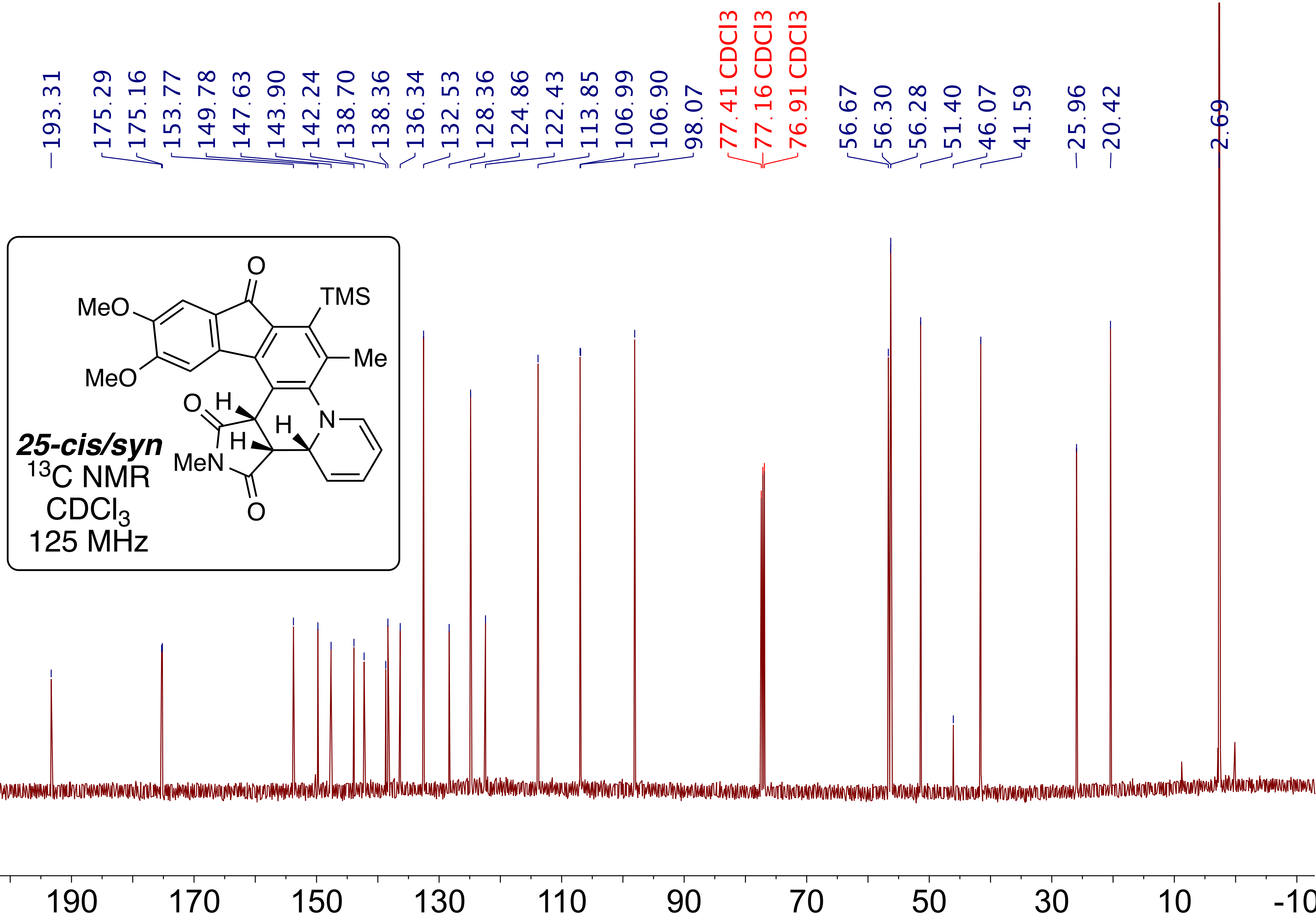
193.31
175.29
175.16
153.77
149.78
147.63
143.90
142.24
138.70
138.36
136.34
132.53
128.36
124.86
122.43
113.85
106.99
106.90
98.07
77.41 CDCl3
77.16 CDCl3
76.91 CDCl3
56.67
56.30
56.28
51.40
46.07
41.59
25.96
20.42
2.69
MeO
MeO
O
TMS
Me
N
H
H
H
O
O
MeN
25-cis/syn
^{13}C NMR
$CDCl_3$
125 MHz
190
170
150
130
110
90
70
50
30
10
-10

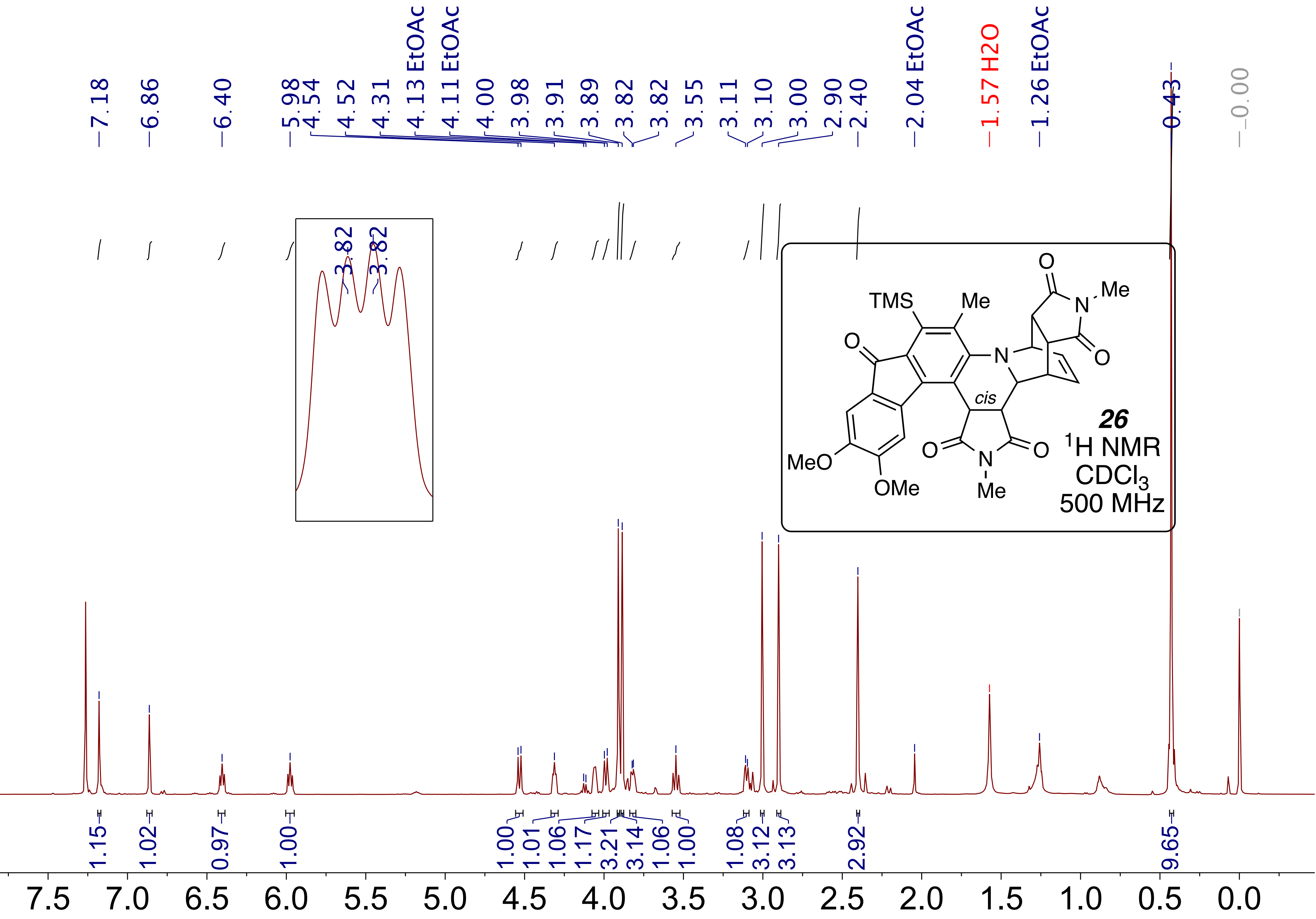
7.18
6.86
6.40
5.98
4.54
4.52
4.31
4.13 EtOAc
4.11 EtOAc
4.00
3.98
3.91
3.89
3.82
3.82
3.55
3.11
3.10
3.00
2.90
2.40
2.04 EtOAc
1.57 H2O
1.26 EtOAc
0.43
0.00
3.82
3.82
TMS
Me
O
N
Me
O
O
N
cis
O
O
N
Me
MeO
OMe
26
1H NMR
CDCl3
500 MHz
1.15
1.02
0.97
1.00
1.00
1.01
1.06
1.17
3.21
3.14
1.06
1.00
1.08
3.12
3.13
2.92
9.65
7.5
7.0
6.5
6.0
5.5
5.0
4.5
4.0
3.5
3.0
2.5
2.0
1.5
1.0
0.5
0.0

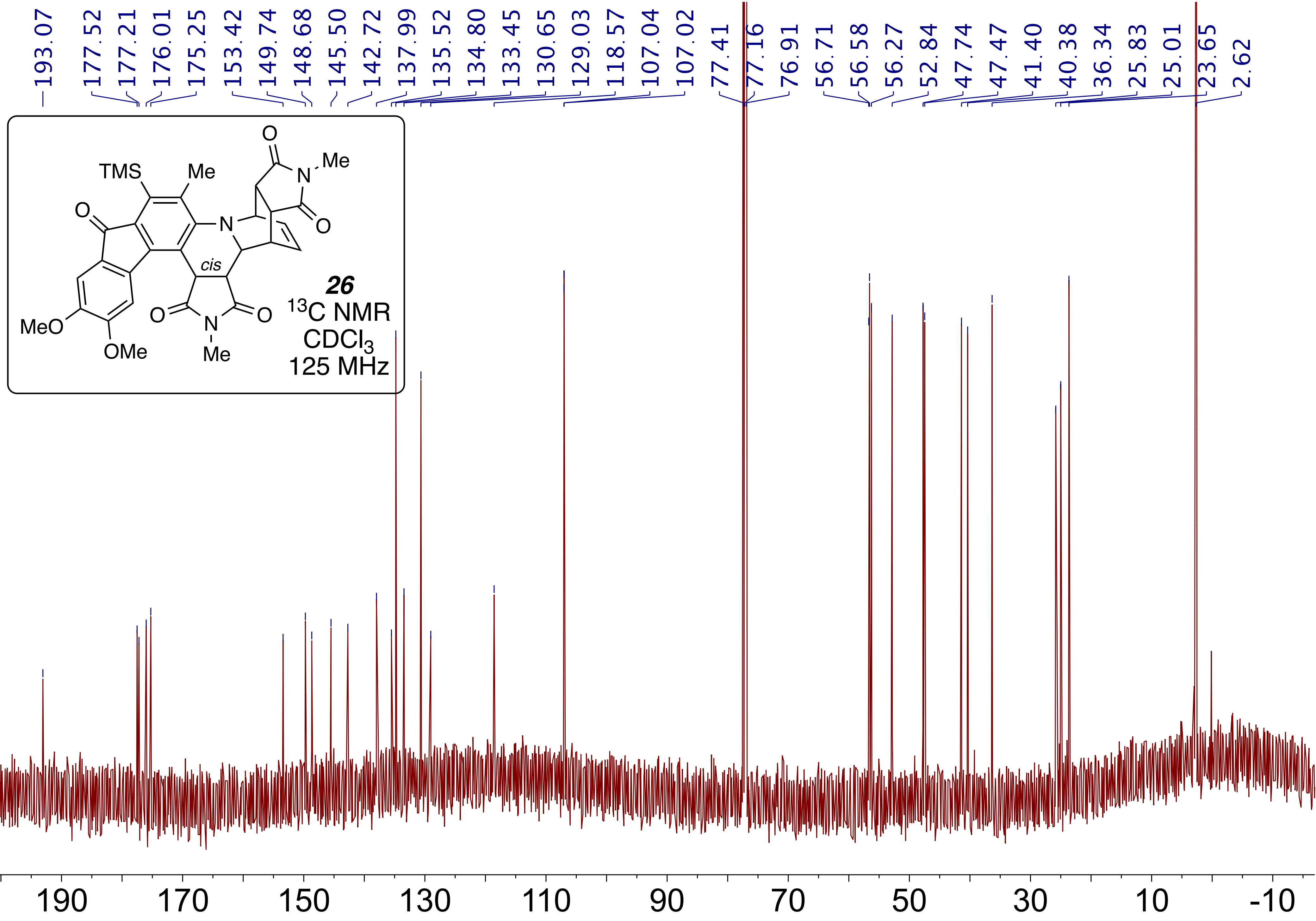
193.07
177.52
177.21
176.01
175.25
153.42
149.74
148.68
145.50
142.72
137.99
135.52
134.80
133.45
130.65
129.03
118.57
107.04
107.02
77.41
77.16
76.91
56.71
56.58
56.27
52.84
47.74
47.47
41.40
40.38
36.34
25.83
25.01
23.65
2.62
TMS
Me
O
N
Me
O
O
N
cis
MeO
OMe
O
N
O
Me
26
^{13}C NMR
$CDCl_3$
125 MHz
190
170
150
130
110
90
70
50
30
10
-10

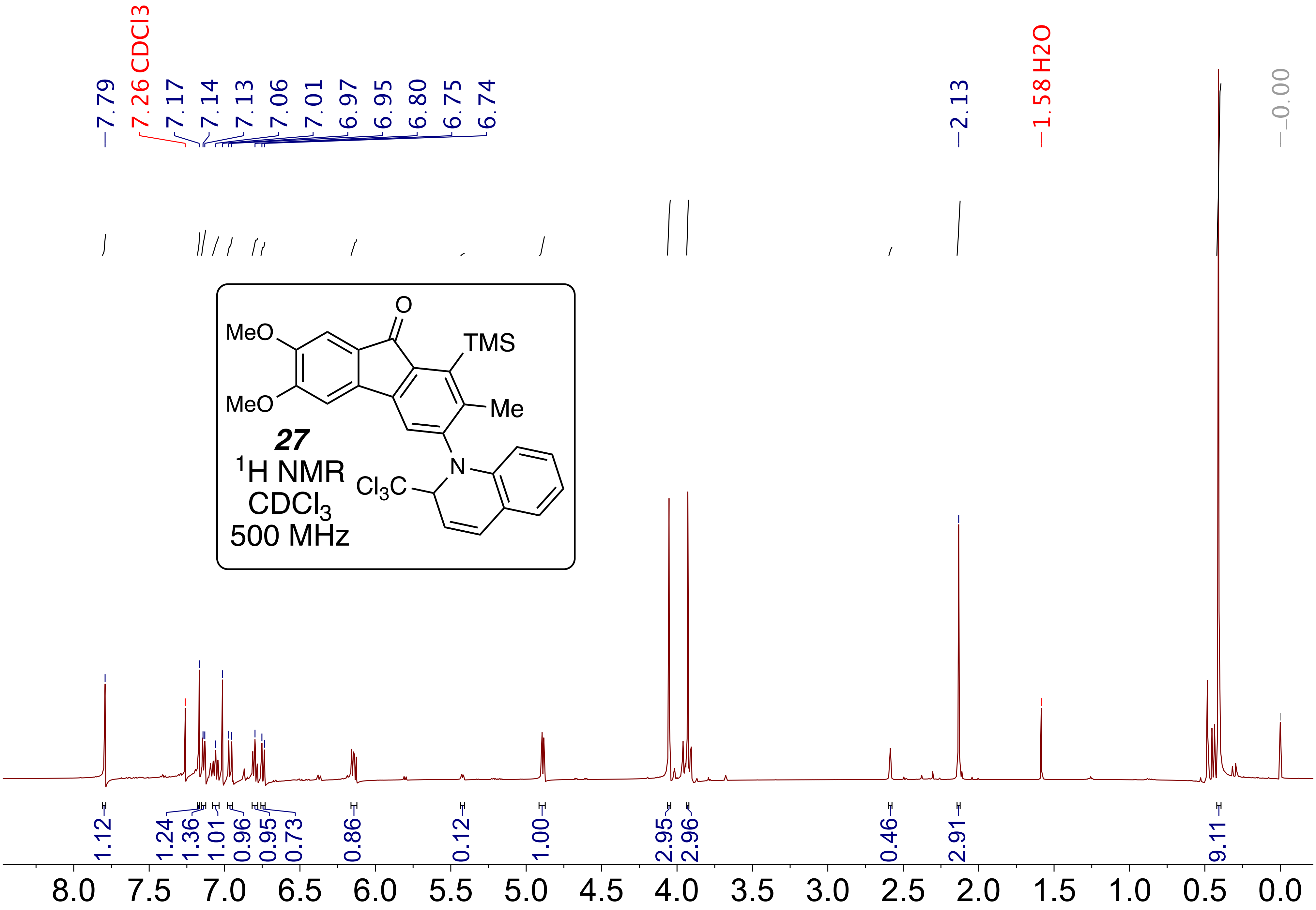
7.79
7.26 CDCl3
7.17
7.14
7.13
7.06
7.01
6.97
6.95
6.80
6.75
6.74
2.13
1.58 H2O
0.00
MeO
MeO
O
TMS
Me
N
Cl3C
27
1H NMR
CDCl3
500 MHz
1.12
1.24
1.36
1.01
0.96
0.95
0.73
0.86
0.12
1.00
2.95
2.96
0.46
2.91
9.11
8.0 7.5 7.0 6.5 6.0 5.5 5.0 4.5 4.0 3.5 3.0 2.5 2.0 1.5 1.0 0.5 0.0

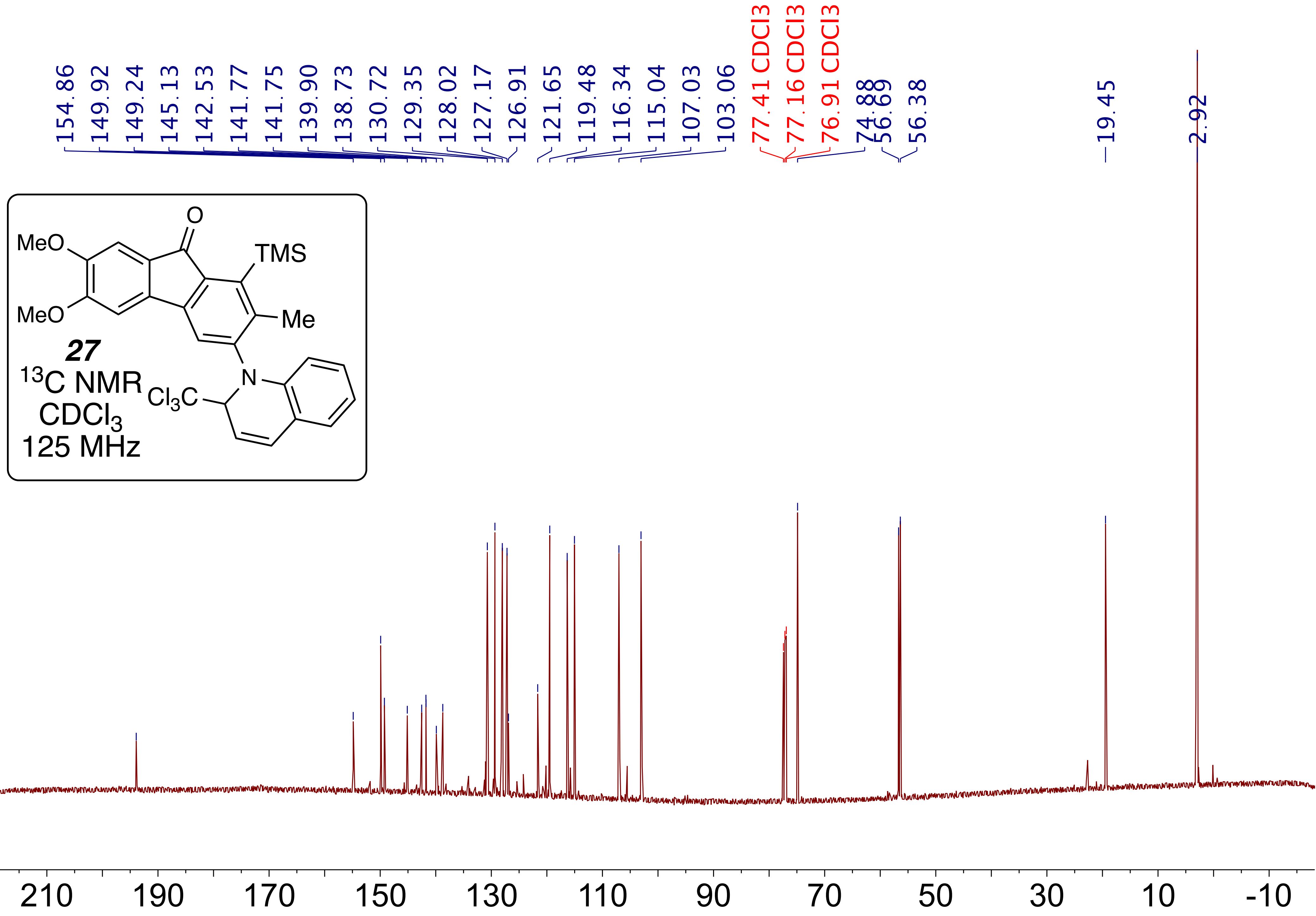

154.86
149.92
149.24
145.13
142.53
141.77
141.75
139.90
138.73
130.72
129.35
128.02
127.17
126.91
121.65
119.48
116.34
115.04
107.03
103.06
77.41 CDCl3
77.16 CDCl3
76.91 CDCl3
74.88
56.69
56.38
19.45
2.92
O
MeO
MeO
TMS
Me
N
Cl3C
27
13C NMR
CDCl3
125 MHz
210
190
170
150
130
110
90
70
50
30
10
-10

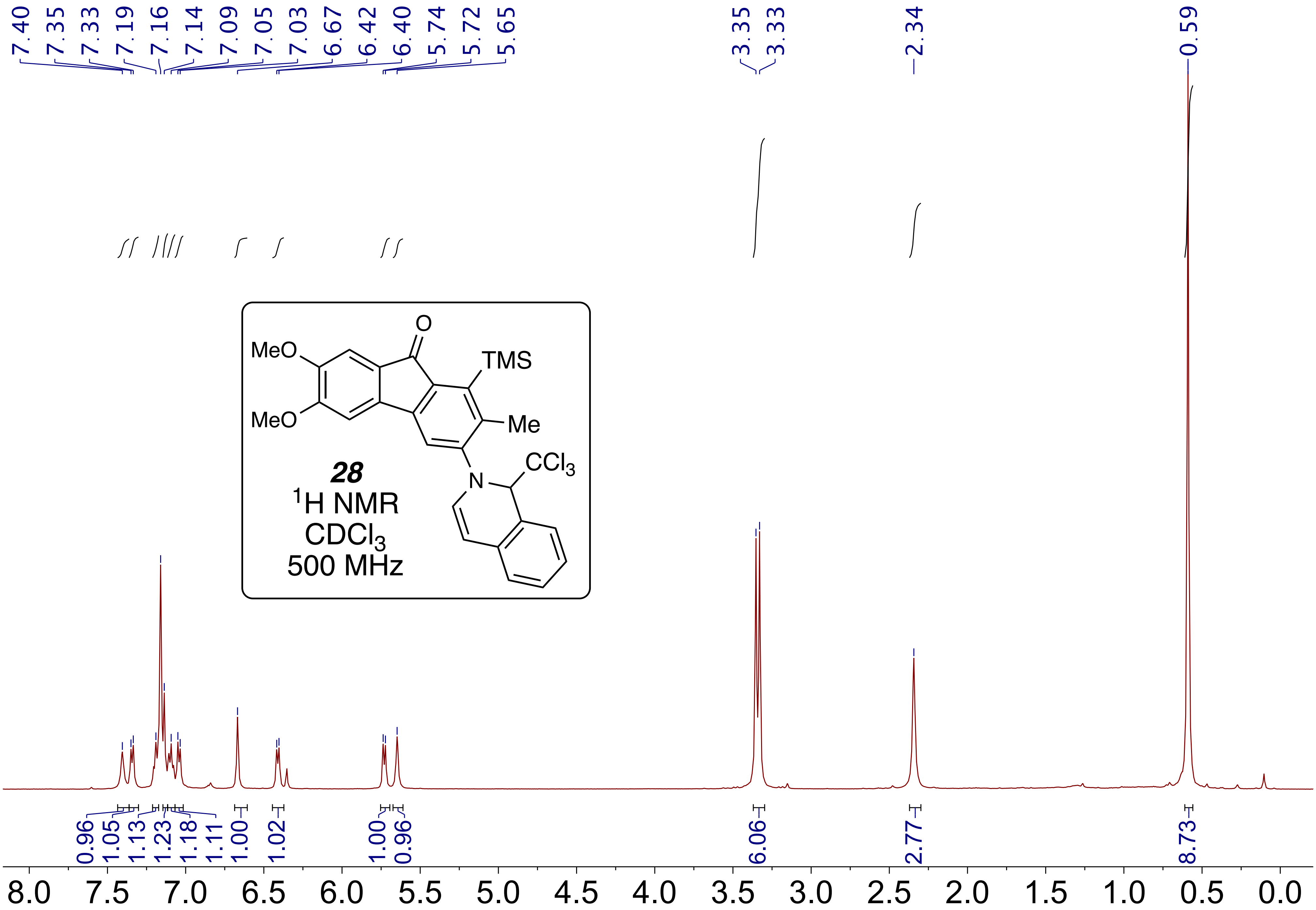
7.40 7.35 7.33 7.19 7.16 7.14 7.09 7.05 7.03 6.67 6.42 6.40 5.74 5.72 5.65
3.35 3.33
2.34
0.59
MeO
MeO
O
TMS
Me
CCl3
N
28
1H NMR
CDCl3
500 MHz
0.96 1.05 1.13 1.23 1.18 1.11 1.00 1.02 1.00 0.96 6.06 2.77 8.73
8.0 7.5 7.0 6.5 6.0 5.5 5.0 4.5 4.0 3.5 3.0 2.5 2.0 1.5 1.0 0.5 0.0

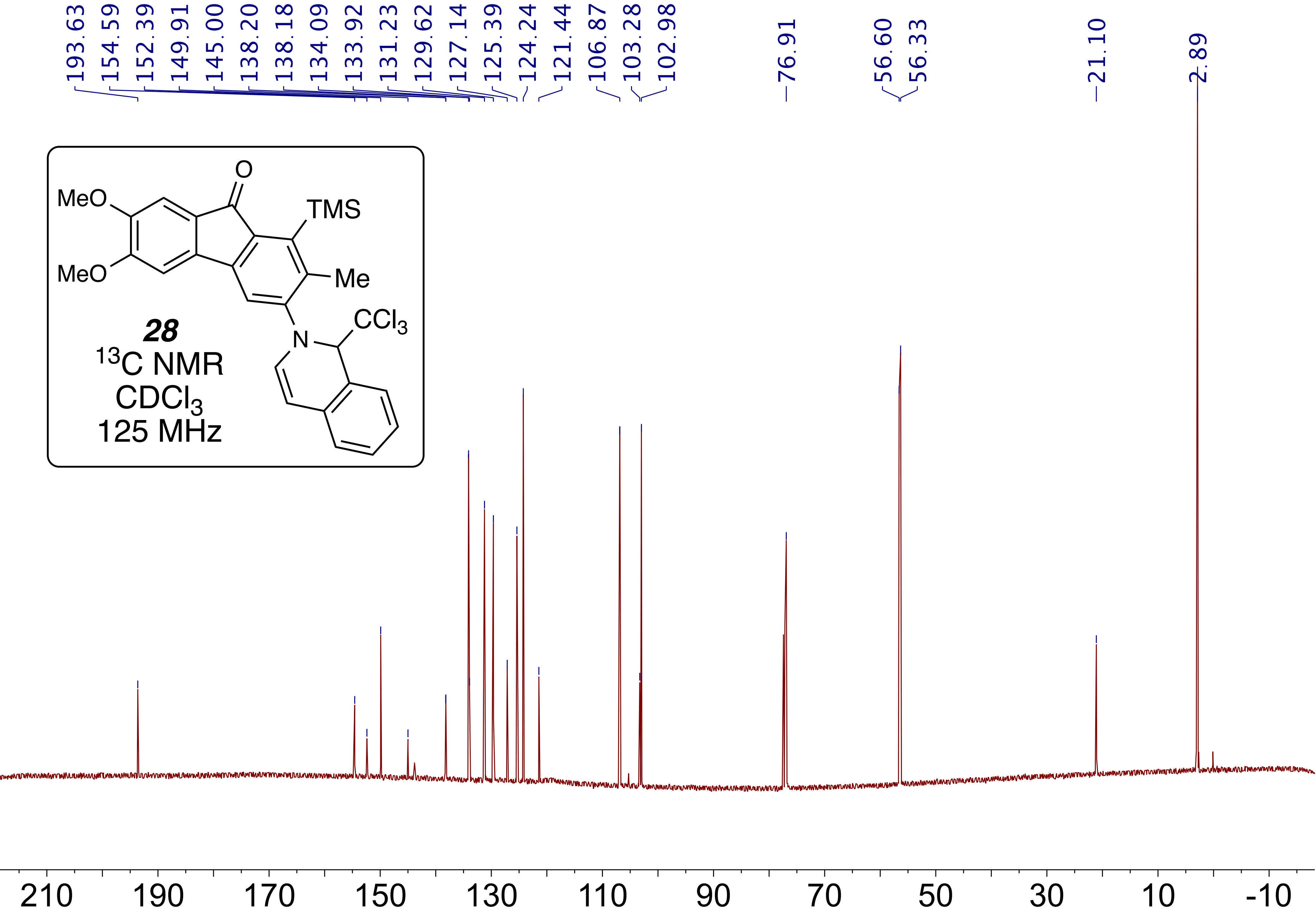
193.63
154.59
152.39
149.91
145.00
138.20
138.18
134.09
133.92
131.23
129.62
127.14
125.39
124.24
121.44
106.87
103.28
102.98
76.91
56.60
56.33
21.10
2.89
MeO
MeO
O
TMS
Me
N
CCl3
28
13C NMR
CDCl3
125 MHz
210
190
170
150
130
110
90
70
50
30
10
-10

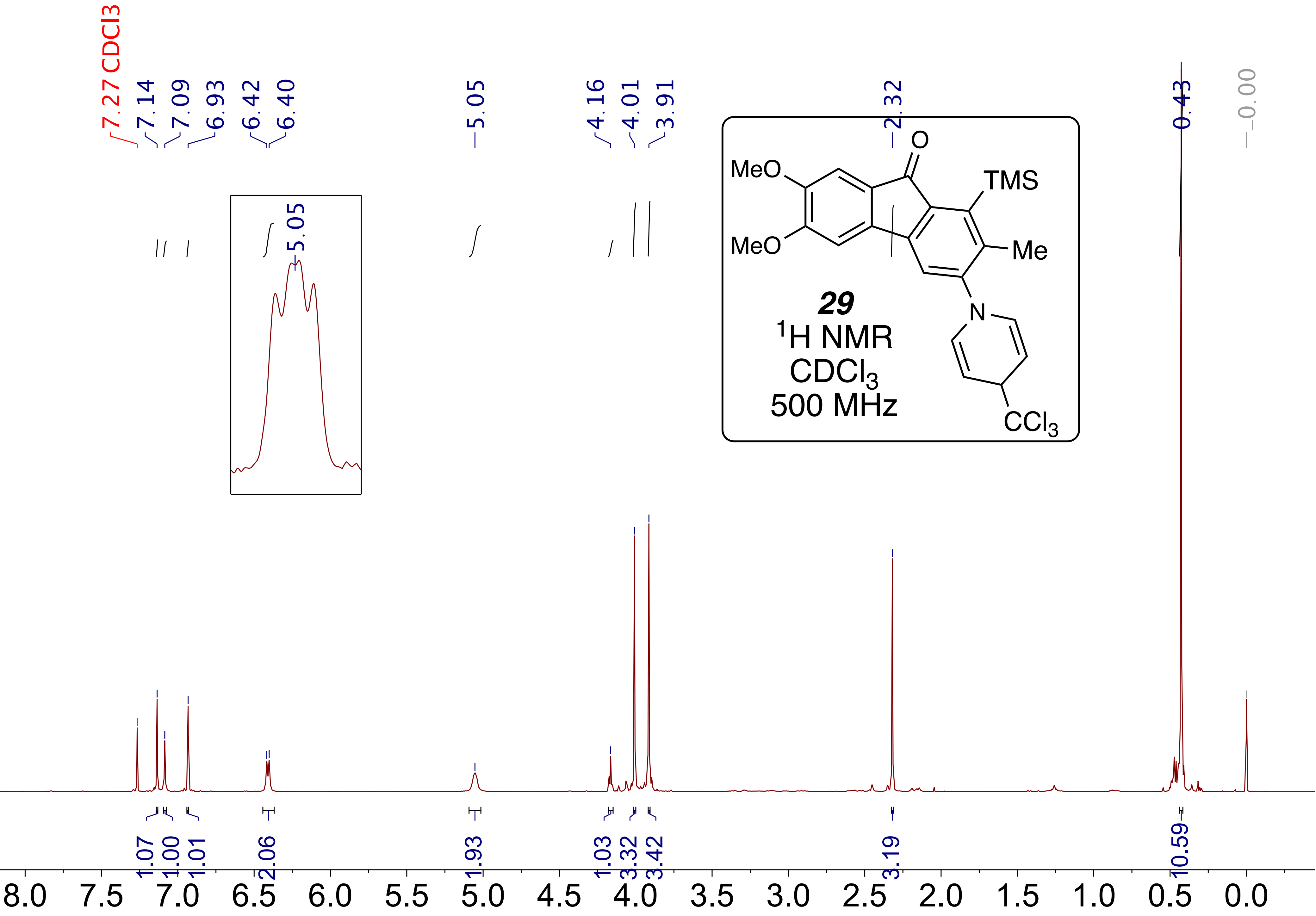
7.27 CDCl3
7.14
7.09
6.93
6.42
6.40
5.05
4.16
4.01
3.91
2.32
0.43
0.00
5.05
O
MeO
MeO
TMS
Me
N
CCl$_3$
29
^{1}H NMR
CDCl$_3$
500 MHz
1.07
1.00
1.01
2.06
1.93
1.03
3.32
3.42
3.19
10.59
8.0
7.5
7.0
6.5
6.0
5.5
5.0
4.5
4.0
3.5
3.0
2.5
2.0
1.5
1.0
0.5
0.0

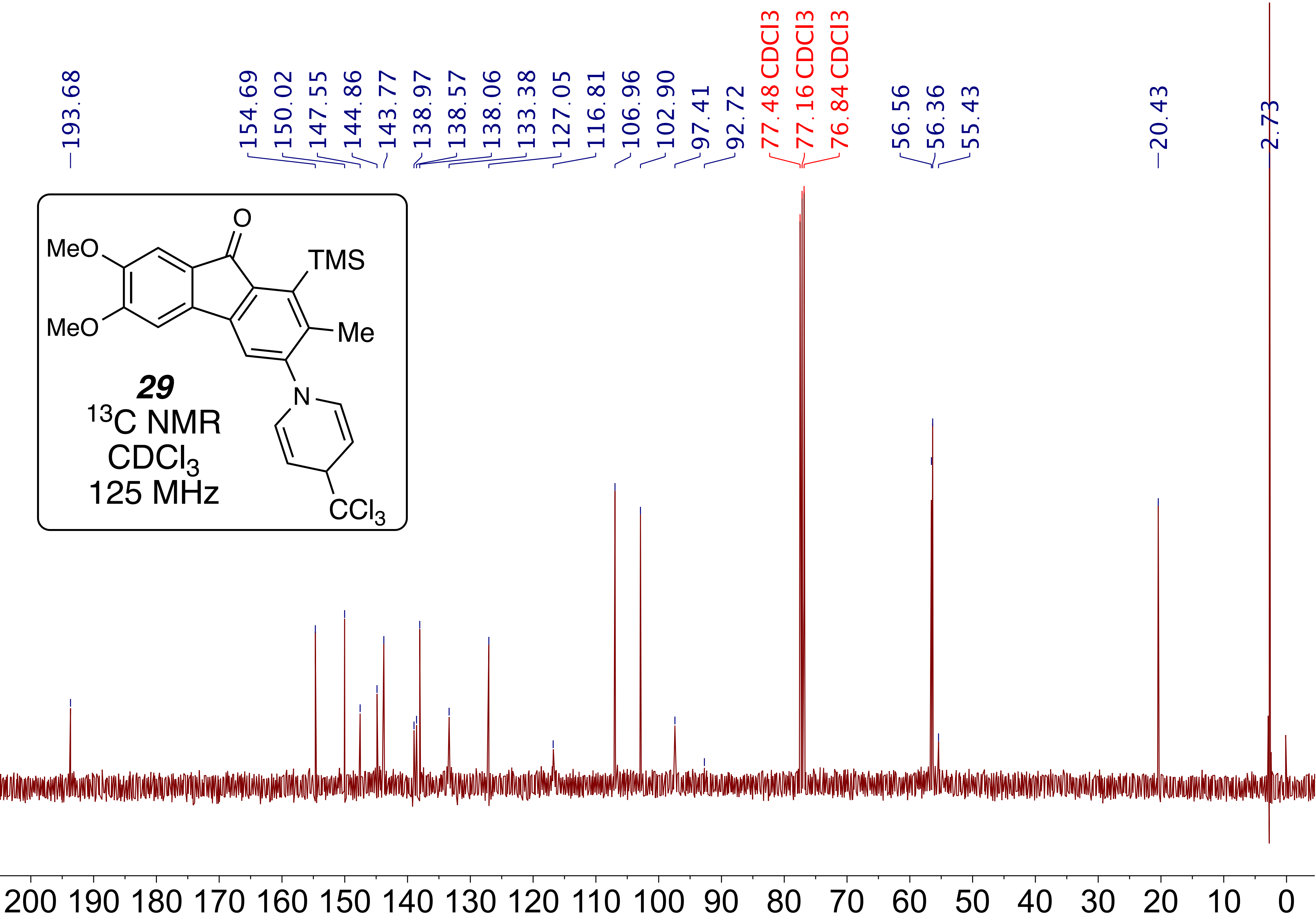
193.68
154.69
150.02
147.55
144.86
143.77
138.97
138.57
138.06
133.38
127.05
116.81
106.96
102.90
97.41
92.72
77.48 CDCl3
77.16 CDCl3
76.84 CDCl3
56.56
56.36
55.43
20.43
2.73
MeO
MeO
O
TMS
Me
N
CCl3
29
13C NMR
CDCl3
125 MHz
200 190 180 170 160 150 140 130 120 110 100 90 80 70 60 50 40 30 20 10 0

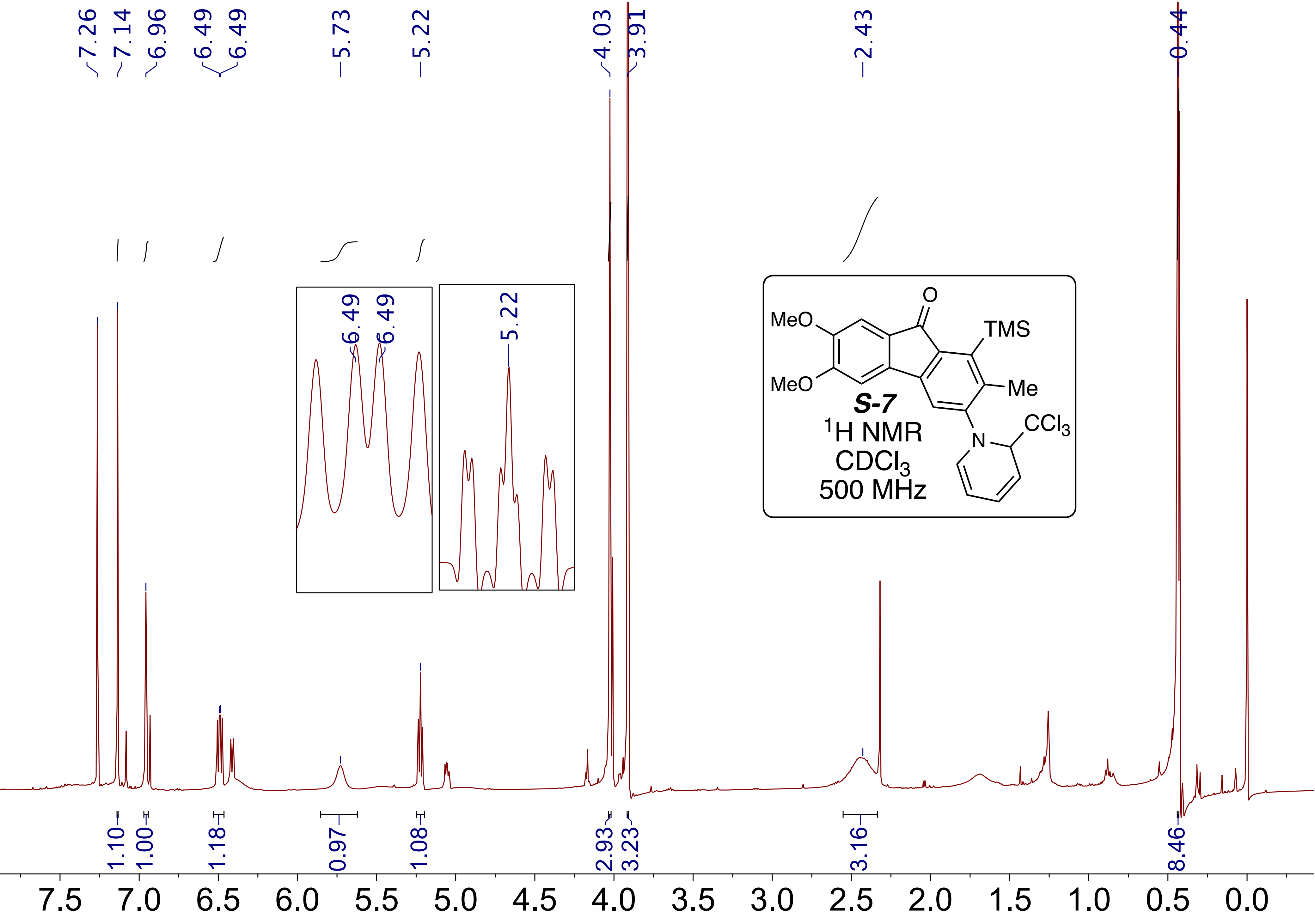

7.26
7.14
6.96
6.49
6.49
5.73
5.22
4.03
3.91
2.43
0.44
6.49
6.49
5.22
MeO
MeO
O
TMS
Me
N
CCl3
S-7
1H NMR
CDCl3
500 MHz
1.10
1.00
1.18
0.97
1.08
2.93
3.23
3.16
8.46
7.5
7.0
6.5
6.0
5.5
5.0
4.5
4.0
3.5
3.0
2.5
2.0
1.5
1.0
0.5
0.0

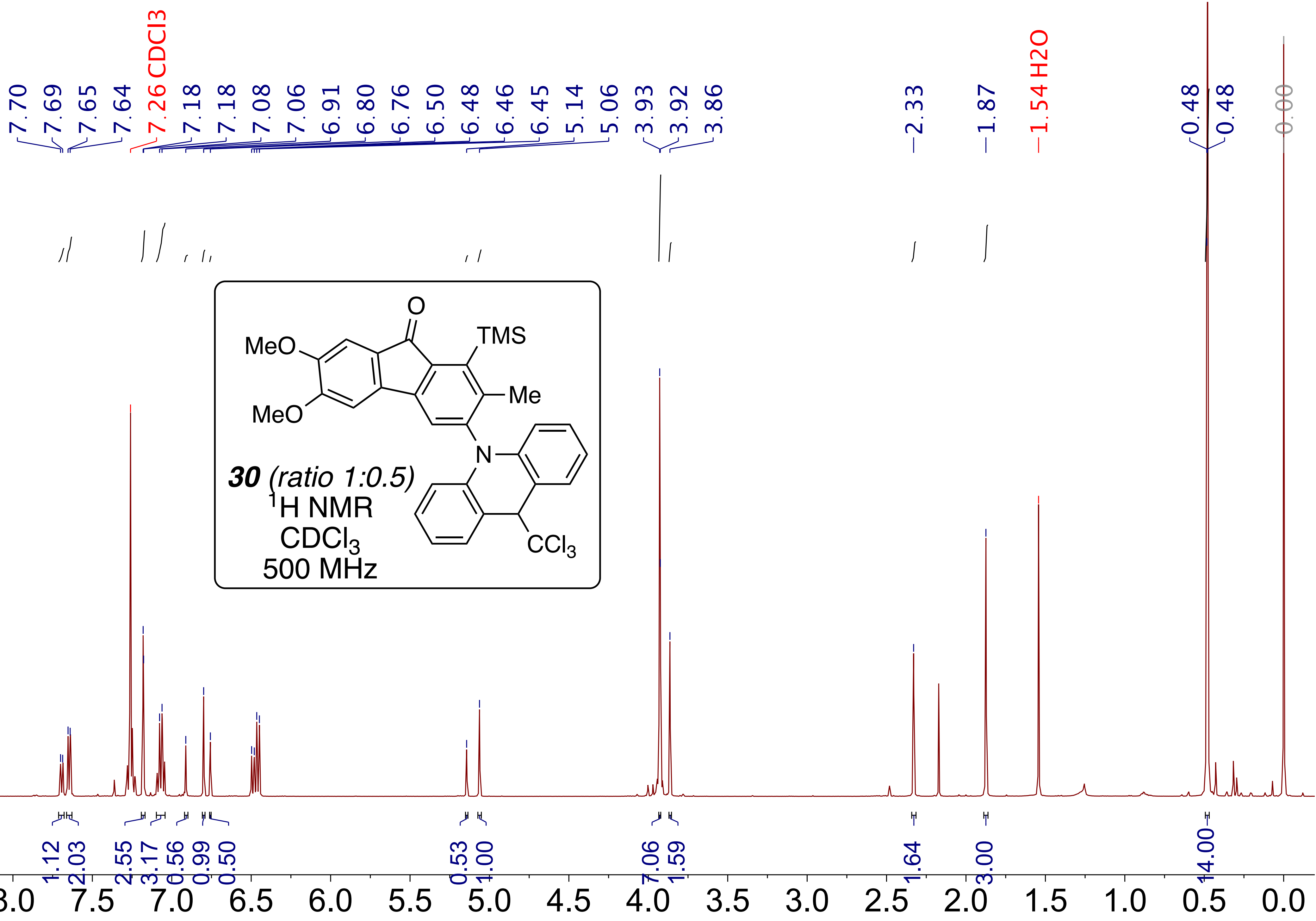
7.70 7.69 7.65 7.64 7.26 CDCl3 7.18 7.18 7.08 7.06 6.91 6.80 6.76 6.50 6.48 6.46 6.45 5.14 5.06 3.93 3.92 3.86
2.33
1.87
1.54 H2O
0.48 0.48
0.00
MeO
MeO
O
TMS
Me
N
CCl3
30 (ratio 1:0.5)
1H NMR
CDCl3
500 MHz
1.12 2.03 2.55 3.17 0.56 0.99 0.50 0.53 1.00 7.06 1.59 1.64 3.00 14.00
8.0 7.5 7.0 6.5 6.0 5.5 5.0 4.5 4.0 3.5 3.0 2.5 2.0 1.5 1.0 0.5 0.0

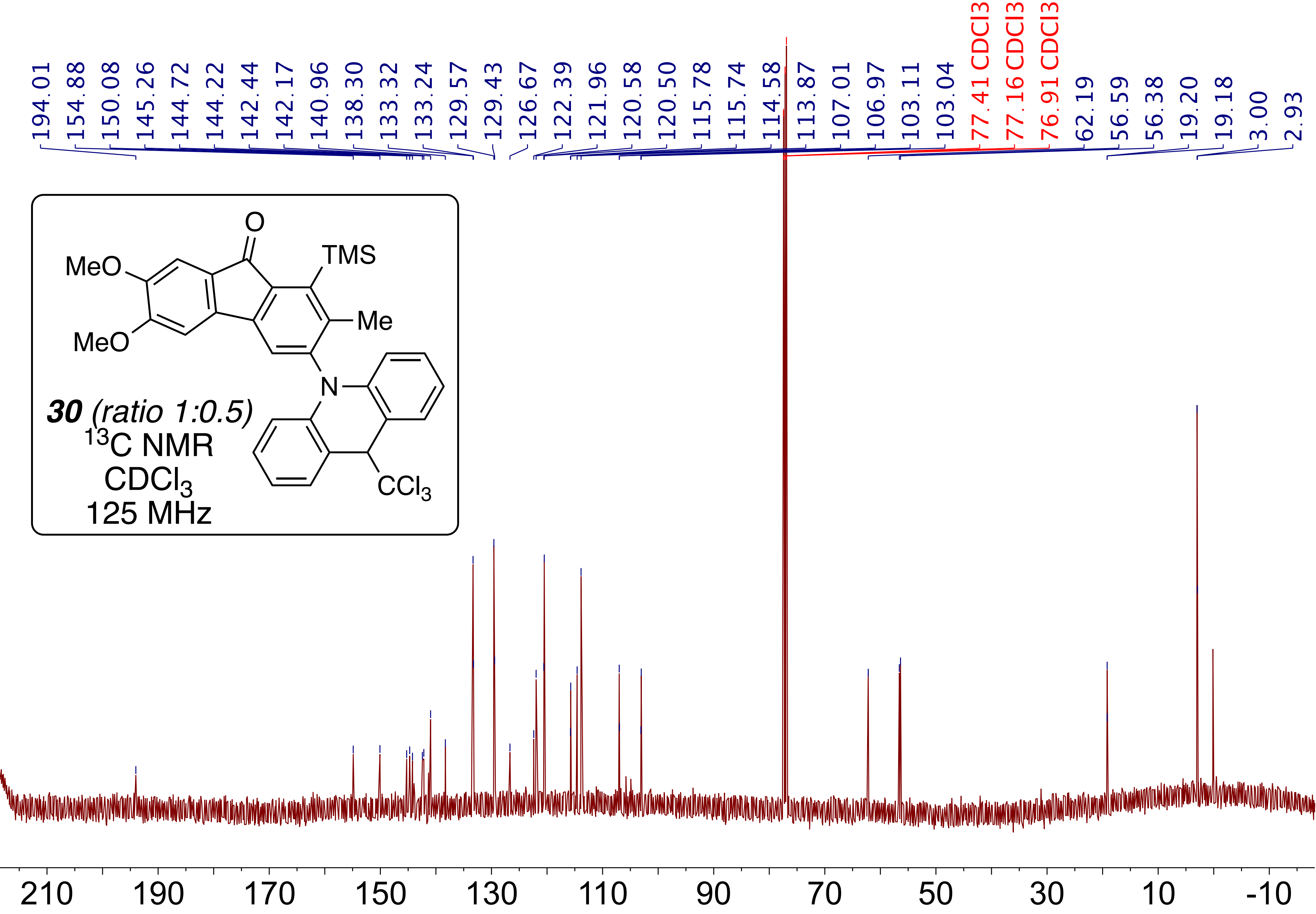
194.01
154.88
150.08
145.26
144.72
144.22
142.44
142.17
140.96
138.30
133.32
133.24
129.57
129.43
126.67
122.39
121.96
120.58
120.50
115.78
115.74
114.58
113.87
107.01
106.97
103.11
103.04
77.41 CDCl3
77.16 CDCl3
76.91 CDCl3
62.19
56.59
56.38
19.20
19.18
3.00
2.93
O
TMS
MeO
Me
MeO
N
CCl3
30 (ratio 1:0.5)
13C NMR
CDCl3
125 MHz
210
190
170
150
130
110
90
70
50
30
10
-10

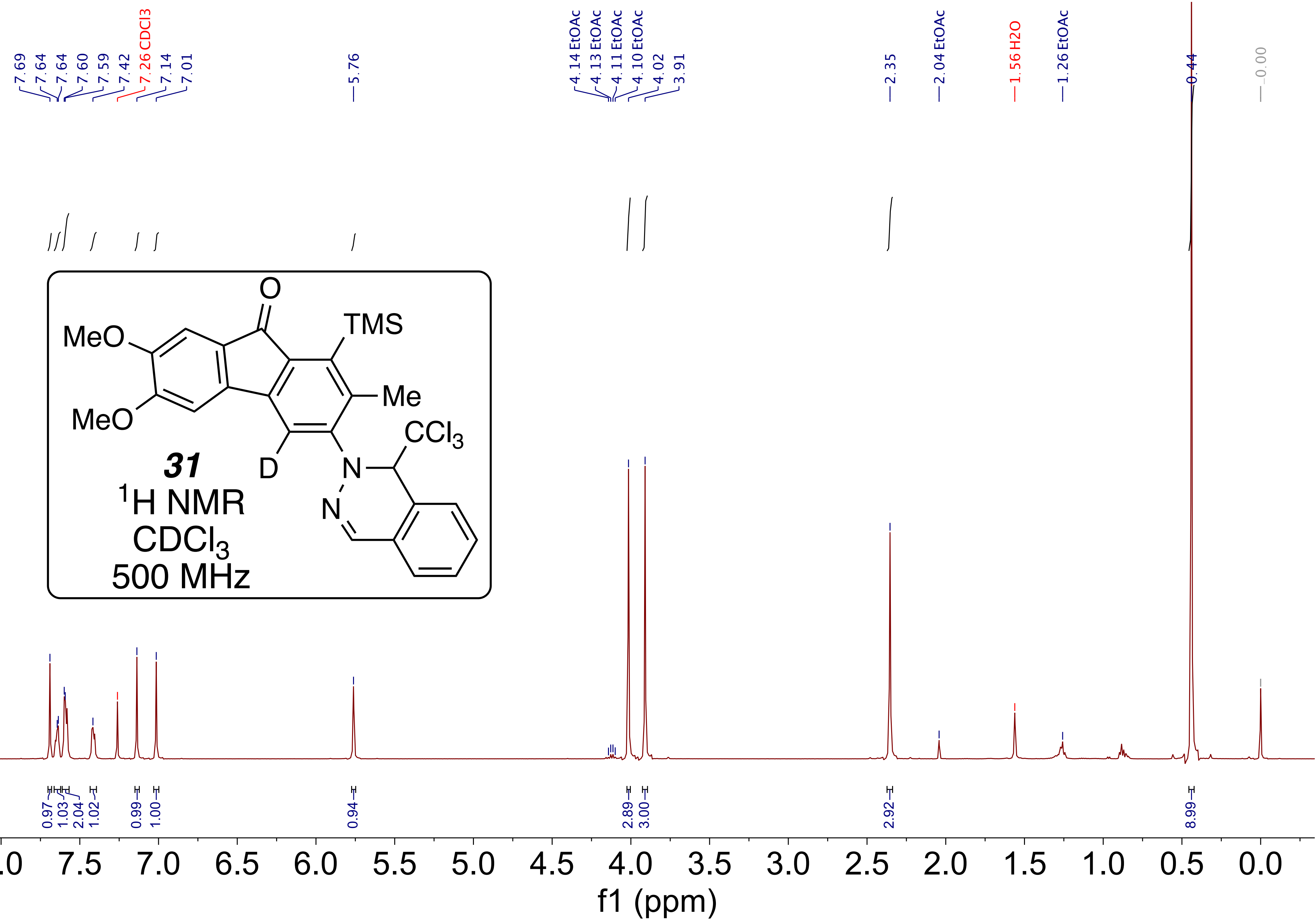
7.69
7.64
7.64
7.60
7.59
7.42
7.26 CDCl3
7.14
7.01
5.76
4.14 EtOAc
4.13 EtOAc
4.11 EtOAc
4.10 EtOAc
4.02
3.91
2.35
2.04 EtOAc
1.56 H2O
1.26 EtOAc
0.44
0.00
O
MeO
MeO
TMS
Me
CCl3
D
N
N
31
1H NMR
CDCl3
500 MHz
0.97
1.03
2.04
1.02
0.99
1.00
0.94
2.89
3.00
2.92
8.99
f1 (ppm)

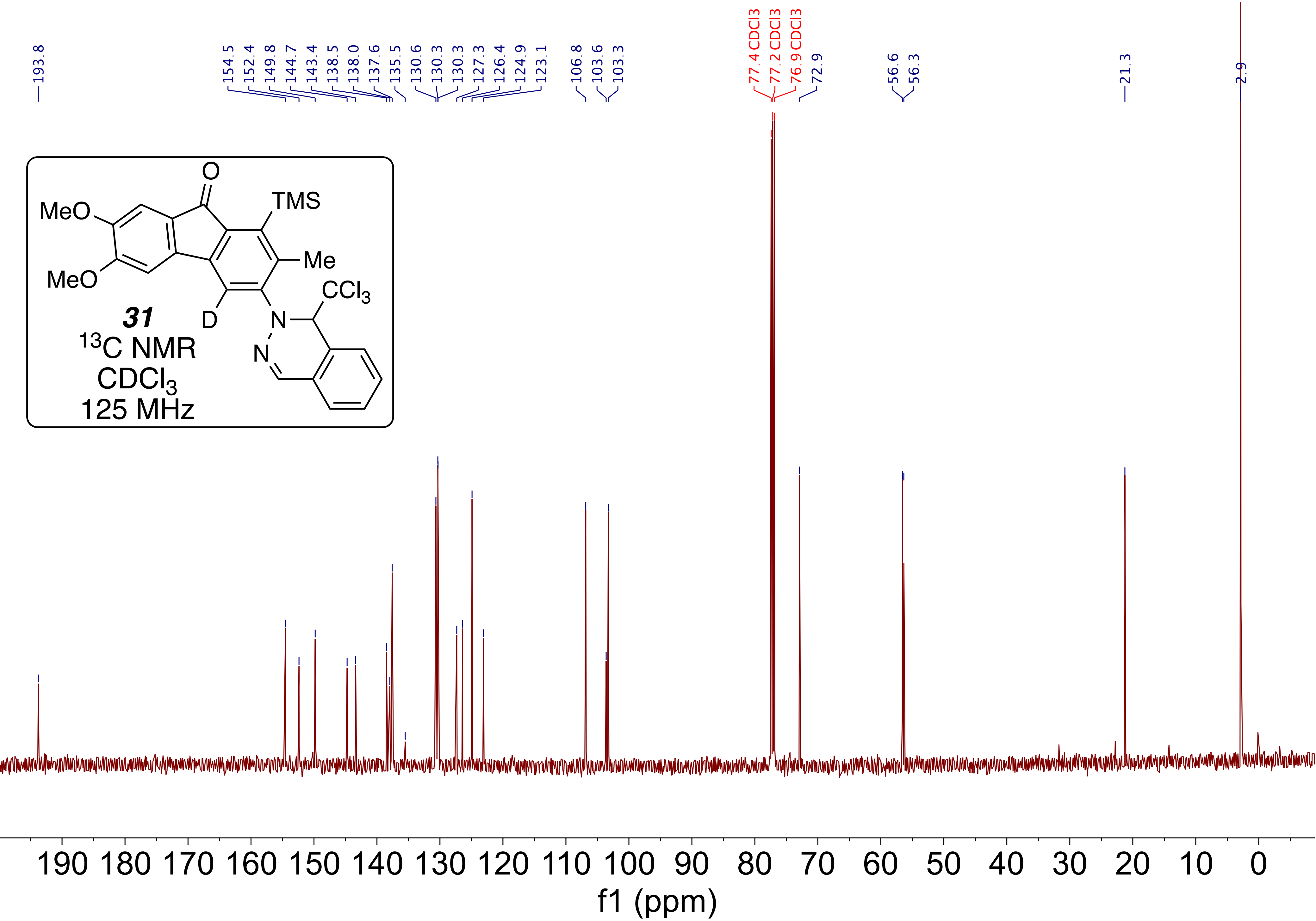
193.8
154.5
152.4
149.8
144.7
143.4
138.5
138.0
137.6
135.5
130.6
130.3
130.3
127.3
126.4
124.9
123.1
106.8
103.6
103.3
77.4 CDCl3
77.2 CDCl3
76.9 CDCl3
72.9
56.6
56.3
21.3
2.9
O
MeO
TMS
Me
MeO
CCl3
D
N
N
31
13C NMR
CDCl3
125 MHz
190 180 170 160 150 140 130 120 110 100 90 80 70 60 50 40 30 20 10 0
f1 (ppm)

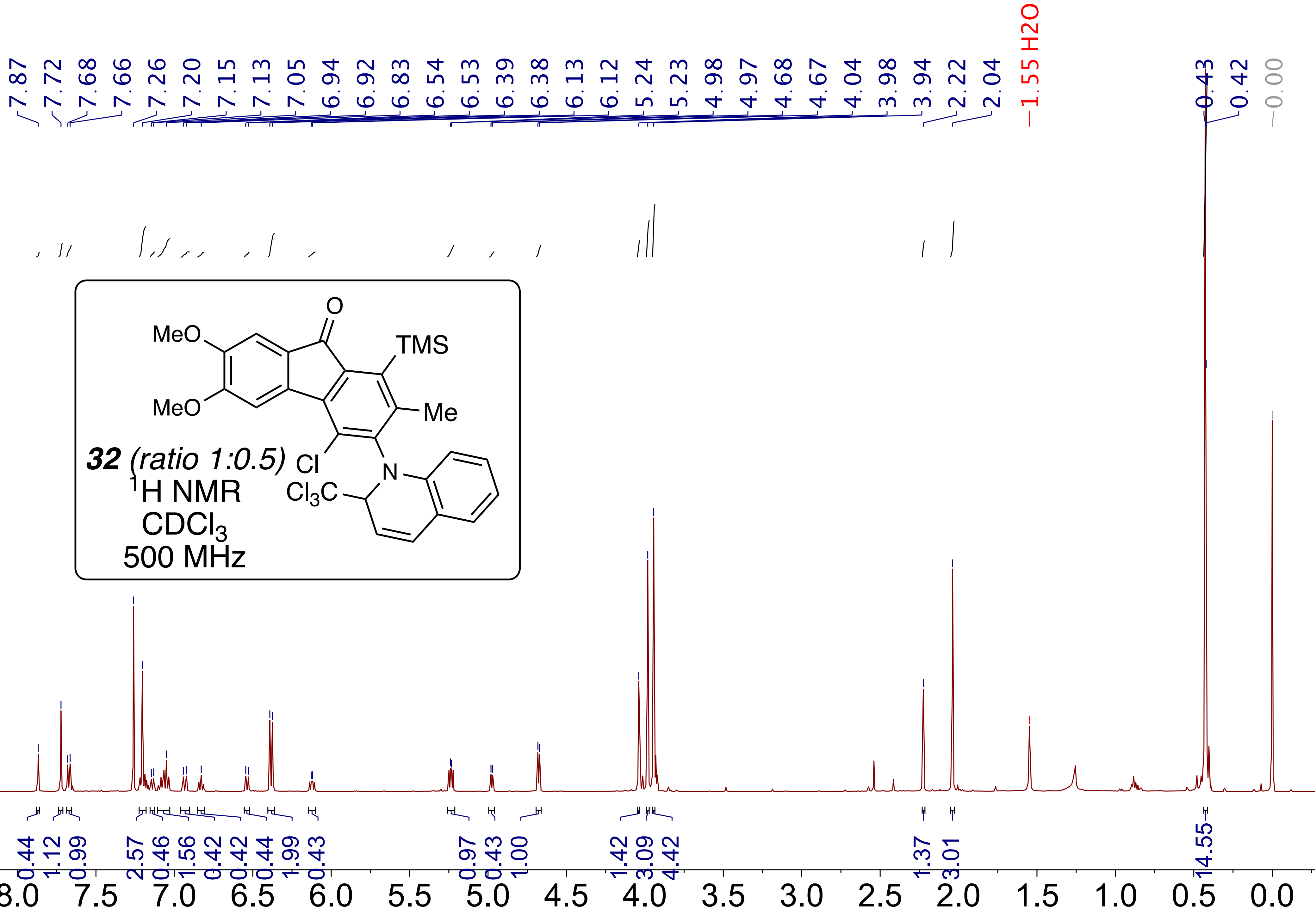
7.87 7.72 7.68 7.66 7.26 7.20 7.15 7.13 7.05 6.94 6.92 6.83 6.54 6.53 6.39 6.38 6.13 6.12 5.24 5.23 4.98 4.97 4.68 4.67 4.04 3.98 3.94 2.22 2.04
1.55 H2O
0.43 0.42 0.00
MeO
MeO
O
TMS
Me
Cl
Cl3C
N
32 (ratio 1:0.5)
1H NMR
CDCl3
500 MHz
0.44 1.12 0.99 2.57 0.46 1.56 0.42 0.42 0.44 1.99 0.43 0.97 0.43 1.00 1.42 3.09 4.42 1.37 3.01 14.55
8.0 7.5 7.0 6.5 6.0 5.5 5.0 4.5 4.0 3.5 3.0 2.5 2.0 1.5 1.0 0.5 0.0

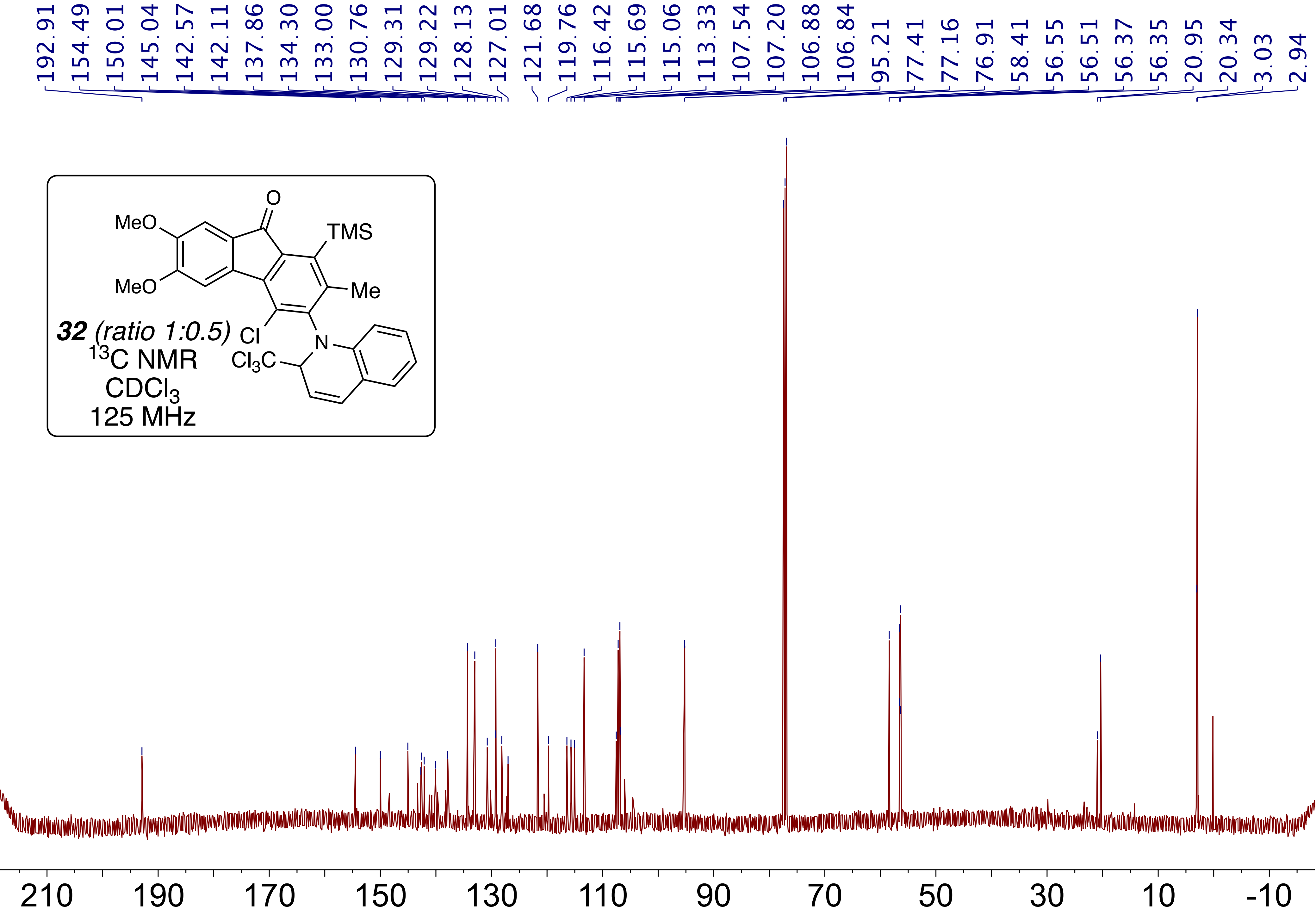

192.91
154.49
150.01
145.04
142.57
142.11
137.86
134.30
133.00
130.76
129.31
129.22
128.13
127.01
121.68
119.76
116.42
115.69
115.06
113.33
107.54
107.20
106.88
106.84
95.21
77.41
77.16
76.91
58.41
56.55
56.51
56.37
56.35
20.95
20.34
3.03
2.94
MeO
MeO
O
TMS
Me
Cl
N
Cl3C
32 (ratio 1:0.5)
13C NMR
CDCl3
125 MHz
210
190
170
150
130
110
90
70
50
30
10
-10

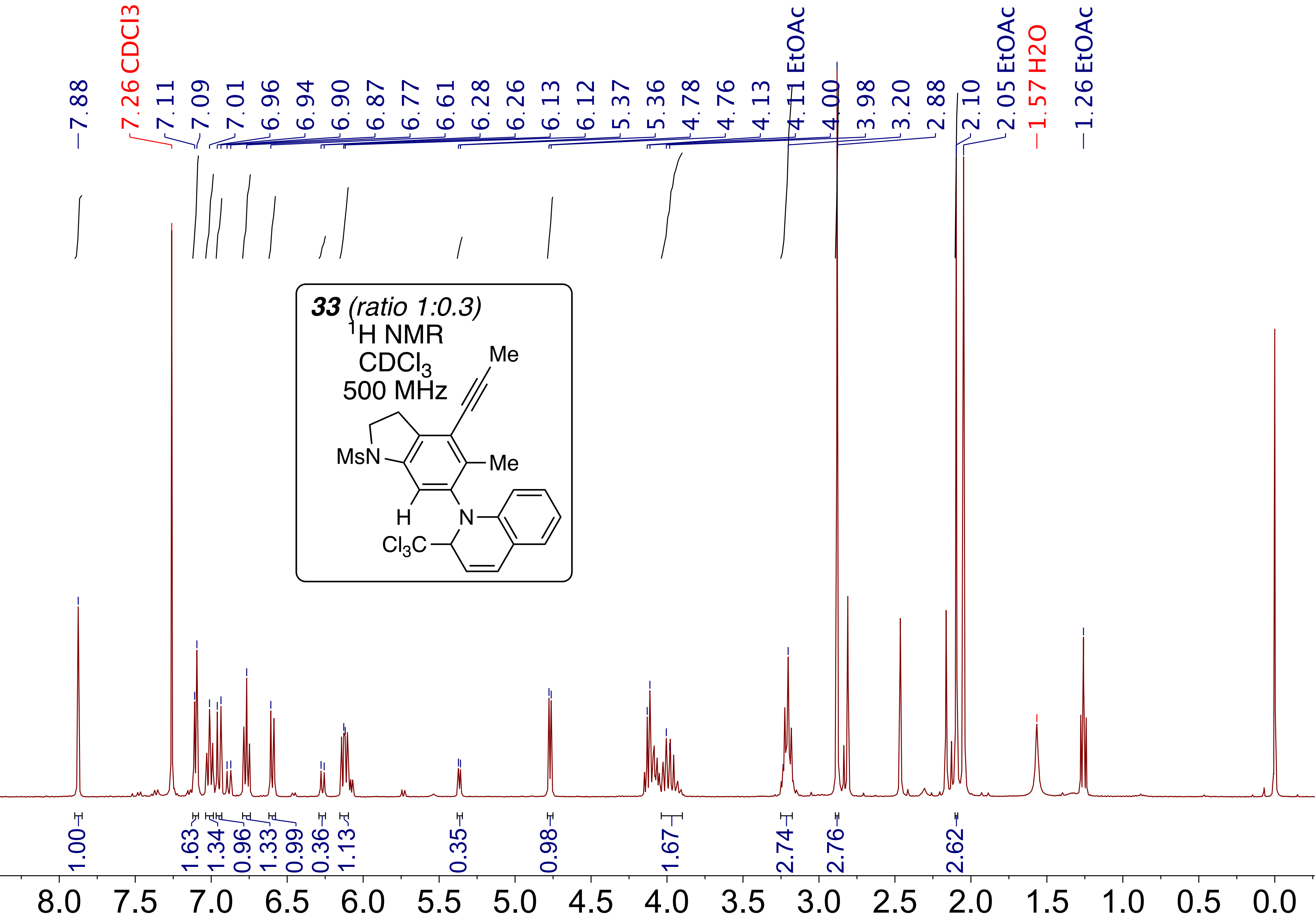
7.88
7.26 CDCl3
7.11
7.09
7.01
6.96
6.94
6.90
6.87
6.77
6.61
6.28
6.26
6.13
6.12
5.37
5.36
4.78
4.76
4.13
4.11 EtOAc
4.00
3.98
3.20
2.88
2.10
2.05 EtOAc
1.57 H2O
1.26 EtOAc
33 (ratio 1:0.3)
1H NMR
CDCl3
500 MHz
Me
MsN
Me
H
N
Cl3C
1.00
1.63
1.34
0.96
1.33
0.99
0.36
1.13
0.35
0.98
1.67
2.74
2.76
2.62
8.0
7.5
7.0
6.5
6.0
5.5
5.0
4.5
4.0
3.5
3.0
2.5
2.0
1.5
1.0
0.5
0.0

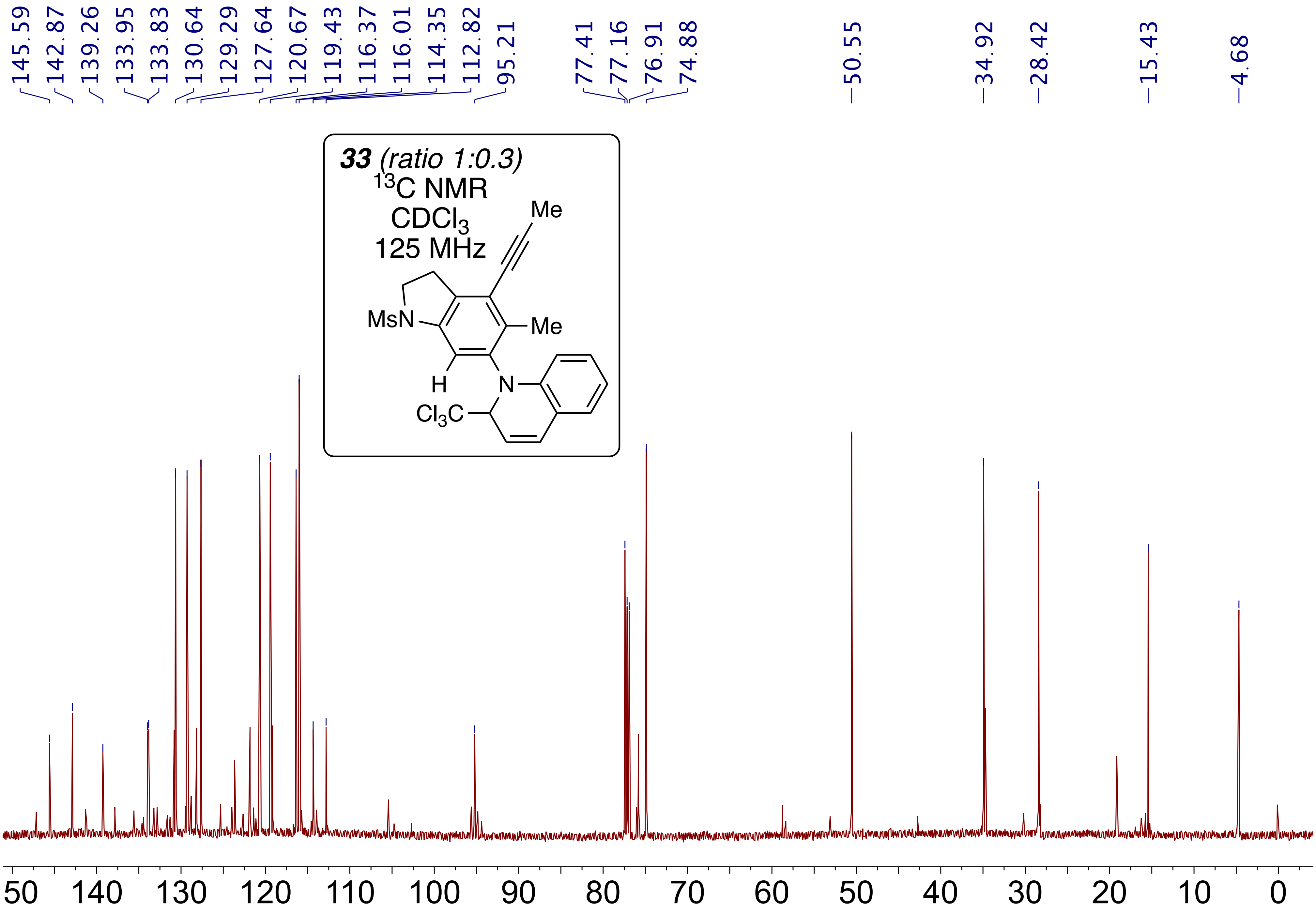
145.59
142.87
139.26
133.95
133.83
130.64
129.29
127.64
120.67
119.43
116.37
116.01
114.35
112.82
95.21
77.41
77.16
76.91
74.88
50.55
34.92
28.42
15.43
4.68
33 (ratio 1:0.3)
13C NMR
CDCl3
125 MHz
Me
MsN
Me
H
N
Cl3C
50
140
130
120
110
100
90
80
70
60
50
40
30
20
10
0

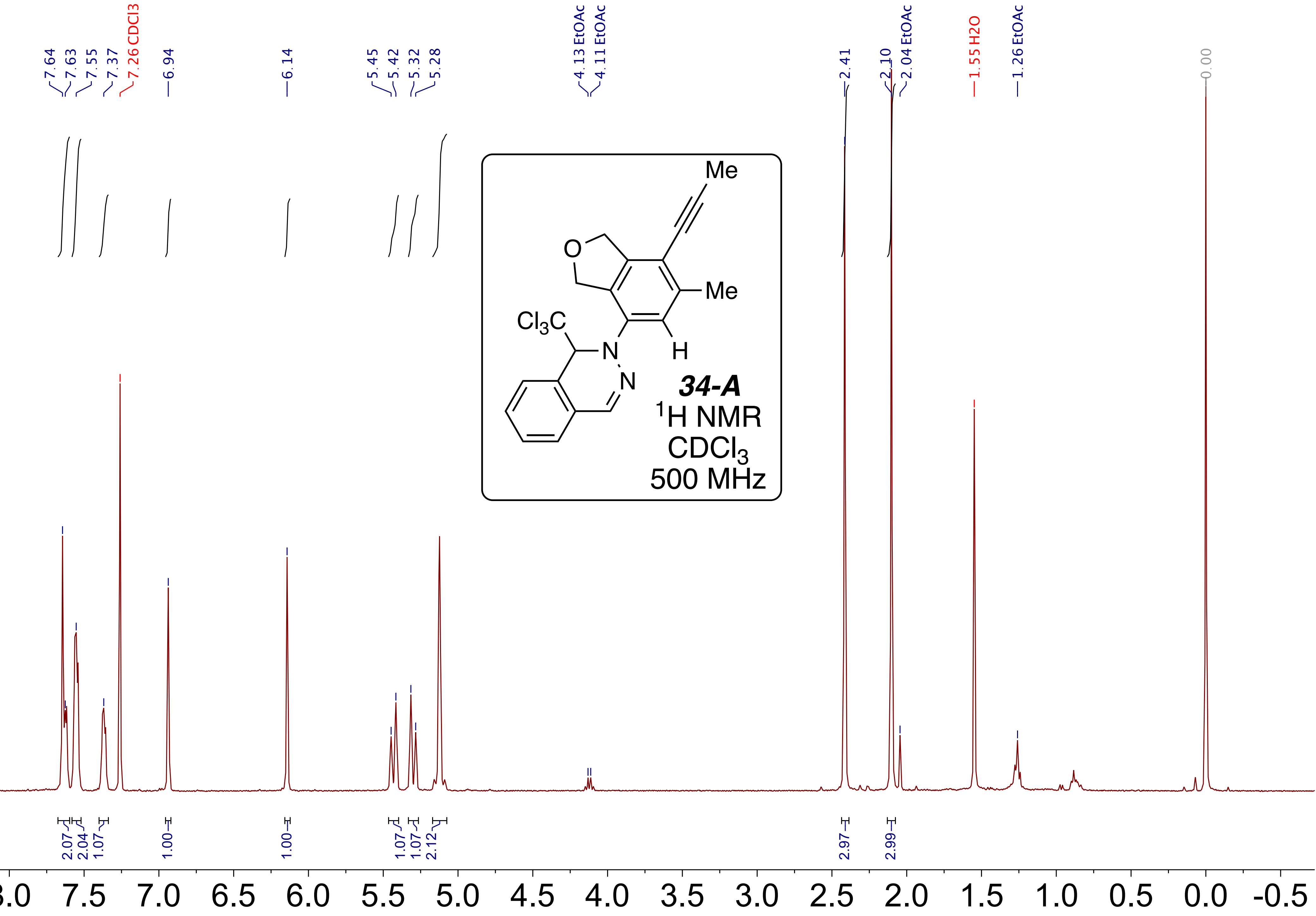
7.64
7.63
7.55
7.37
7.26 CDCl3
6.94
6.14
5.45
5.42
5.32
5.28
4.13 EtOAc
4.11 EtOAc
2.41
2.10
2.04 EtOAc
1.55 H2O
1.26 EtOAc
0.00
Me
O
Me
Cl3C
N
N
H
34-A
1H NMR
CDCl3
500 MHz
2.07
2.04
1.07
1.00
1.00
1.07
1.07
2.12
2.97
2.99
8.0 7.5 7.0 6.5 6.0 5.5 5.0 4.5 4.0 3.5 3.0 2.5 2.0 1.5 1.0 0.5 0.0 -0.5

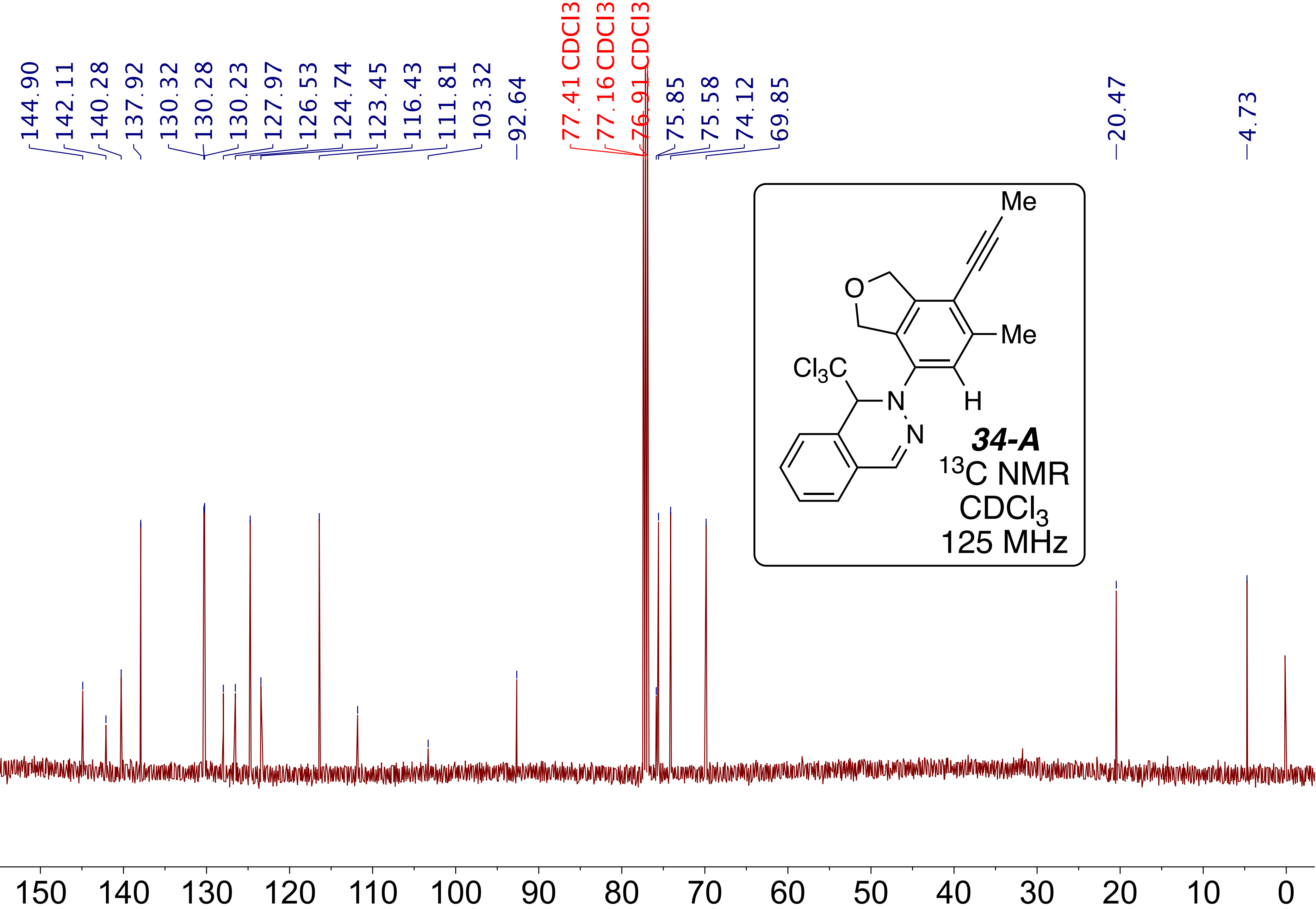

144.90
142.11
140.28
137.92
130.32
130.28
130.23
127.97
126.53
124.74
123.45
116.43
111.81
103.32
92.64
77.41 CDCl3
77.16 CDCl3
76.91 CDCl3
75.85
75.58
74.12
69.85
20.47
4.73
Me
O
Me
Cl3C
N
N
H
34-A
13C NMR
CDCl3
125 MHz
150
140
130
120
110
100
90
80
70
60
50
40
30
20
10
0

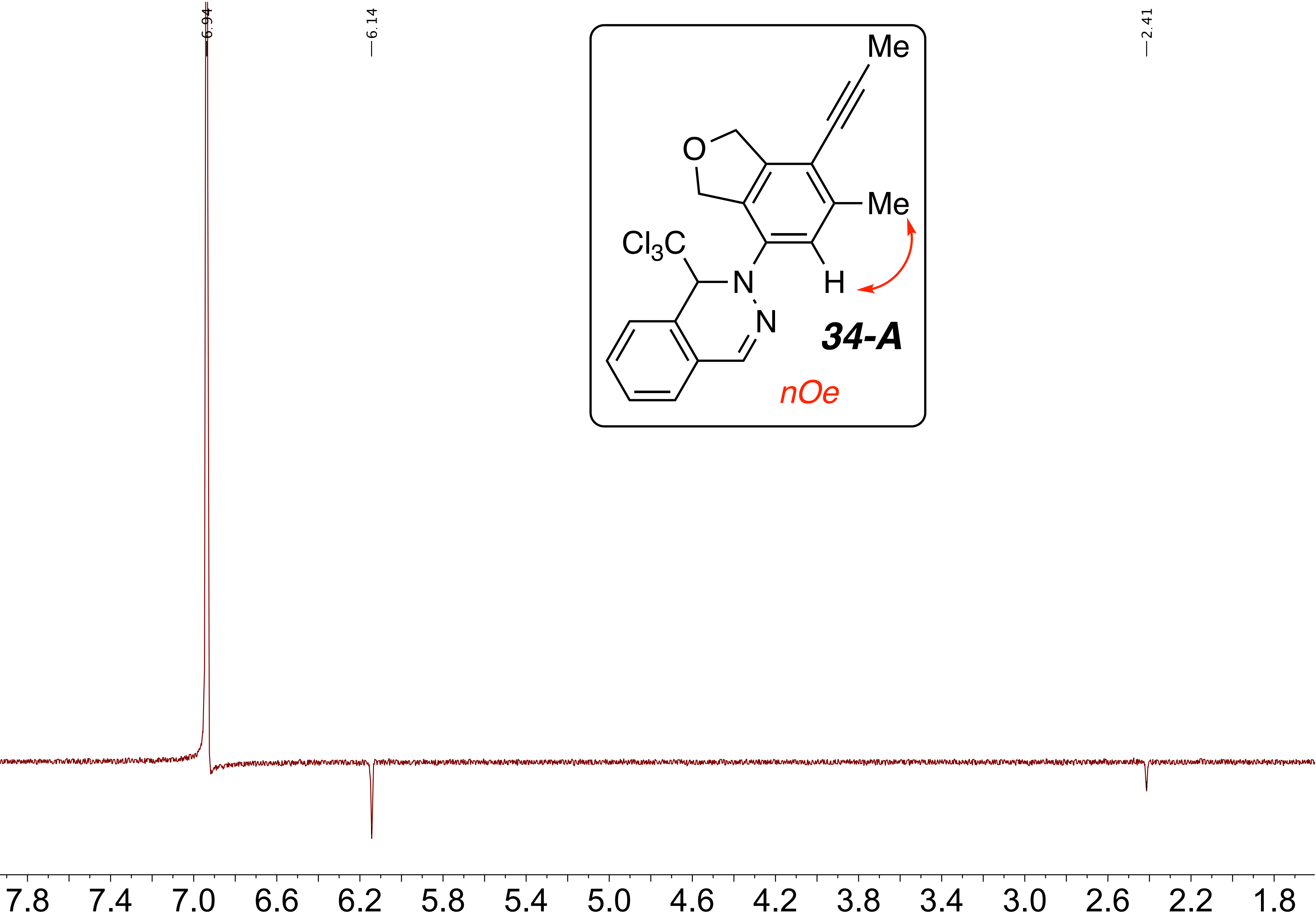
6.94
6.14
2.41
Me
O
Me
Cl3C
N
H
N
34-A
nOe
7.8
7.4
7.0
6.6
6.2
5.8
5.4
5.0
4.6
4.2
3.8
3.4
3.0
2.6
2.2
1.8

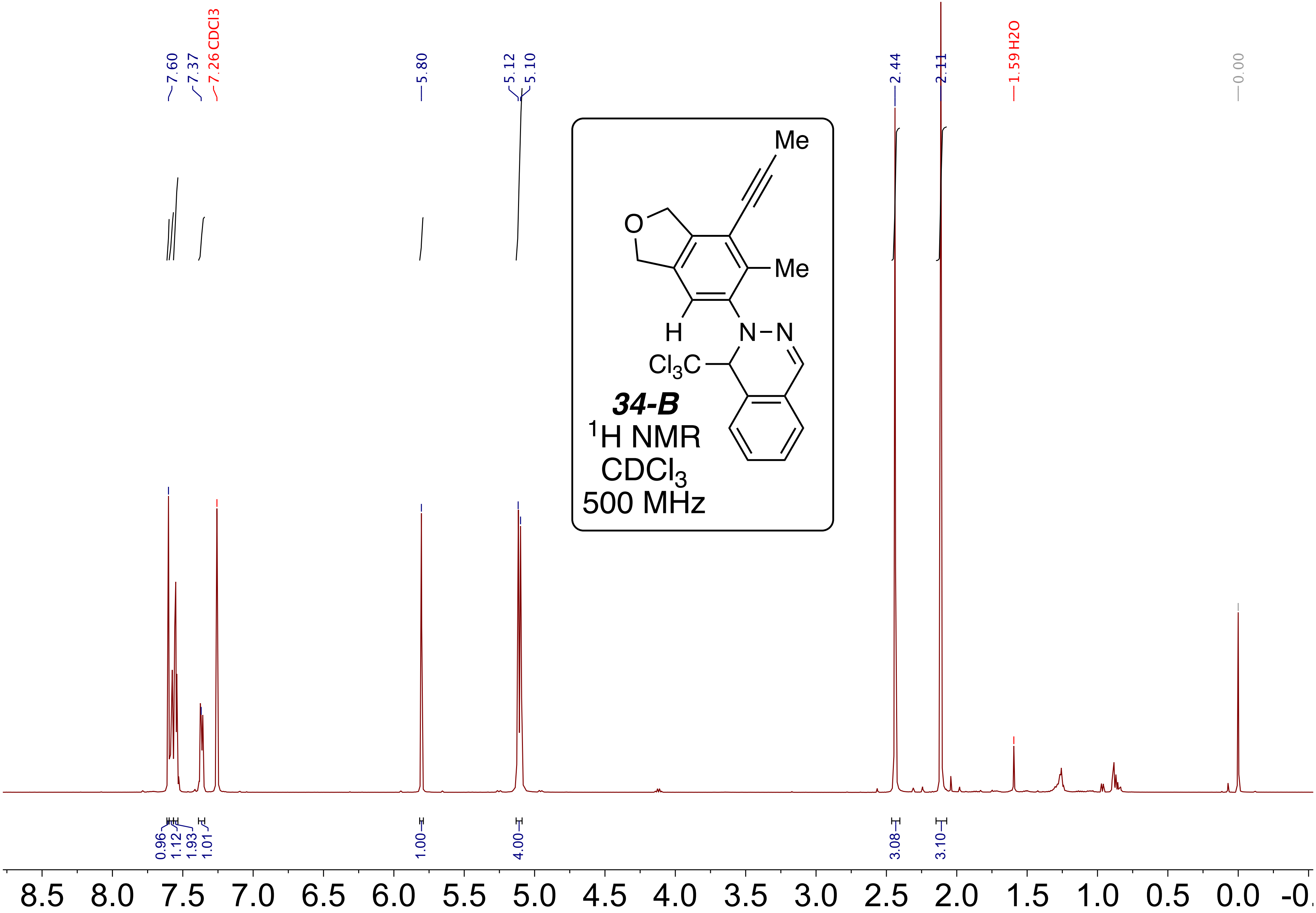
7.60
7.37
7.26 CDCl3
5.80
5.12
5.10
2.44
2.11
1.59 H2O
0.00
Me
O
Me
H
N−N
Cl3C
34-B
1H NMR
CDCl3
500 MHz
0.96
1.12
1.93
1.01
1.00
4.00
3.08
3.10
8.5
8.0
7.5
7.0
6.5
6.0
5.5
5.0
4.5
4.0
3.5
3.0
2.5
2.0
1.5
1.0
0.5
0.0

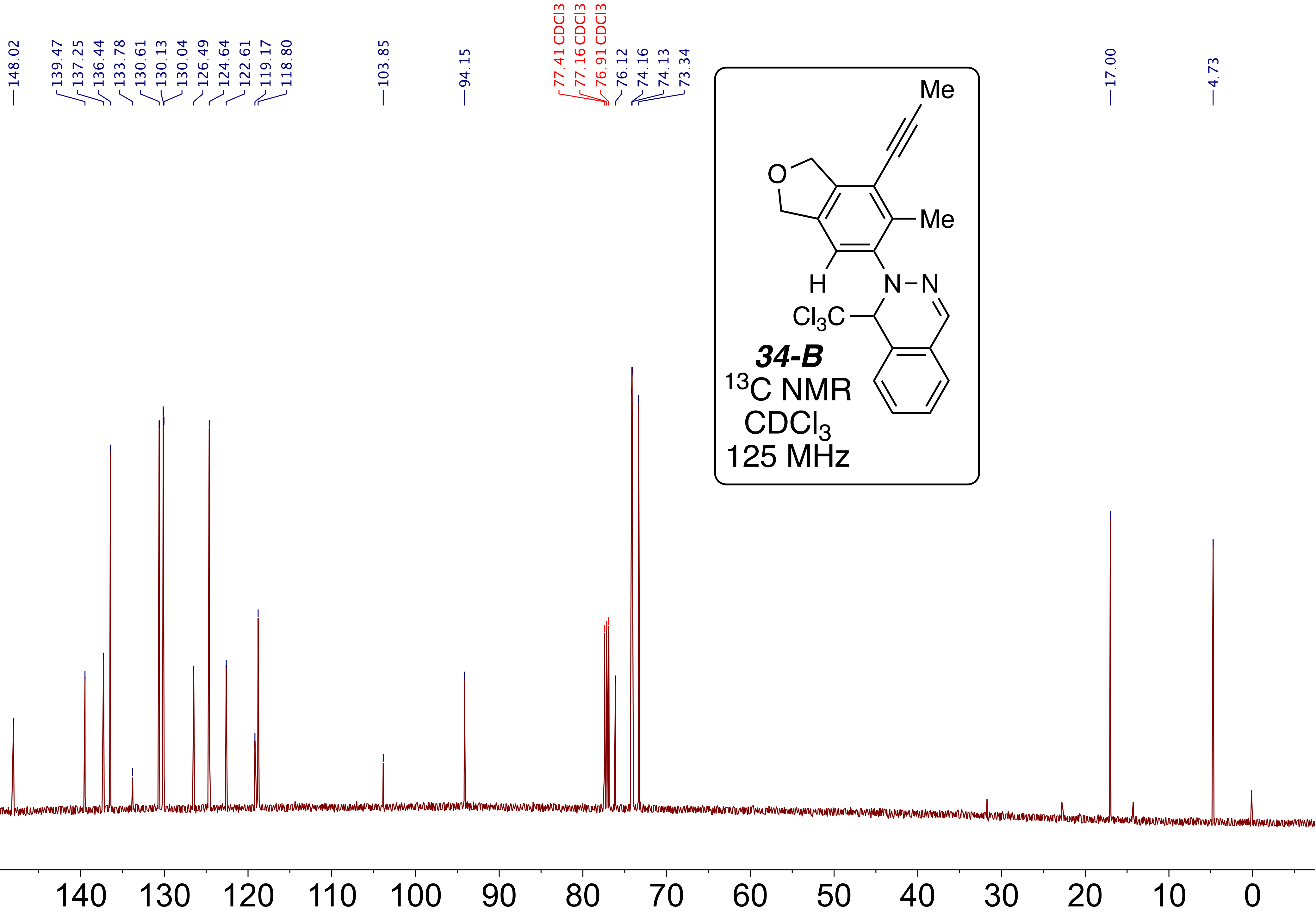
148.02
139.47
137.25
136.44
133.78
130.61
130.13
130.04
126.49
124.64
122.61
119.17
118.80
103.85
94.15
77.41 CDCl3
77.16 CDCl3
76.91 CDCl3
76.12
74.16
74.13
73.34
17.00
4.73
Me
O
Me
H
N
N
Cl_3C
34-B
^{13}C NMR
$CDCl_3$
125 MHz
140
130
120
110
100
90
80
70
60
50
40
30
20
10
0

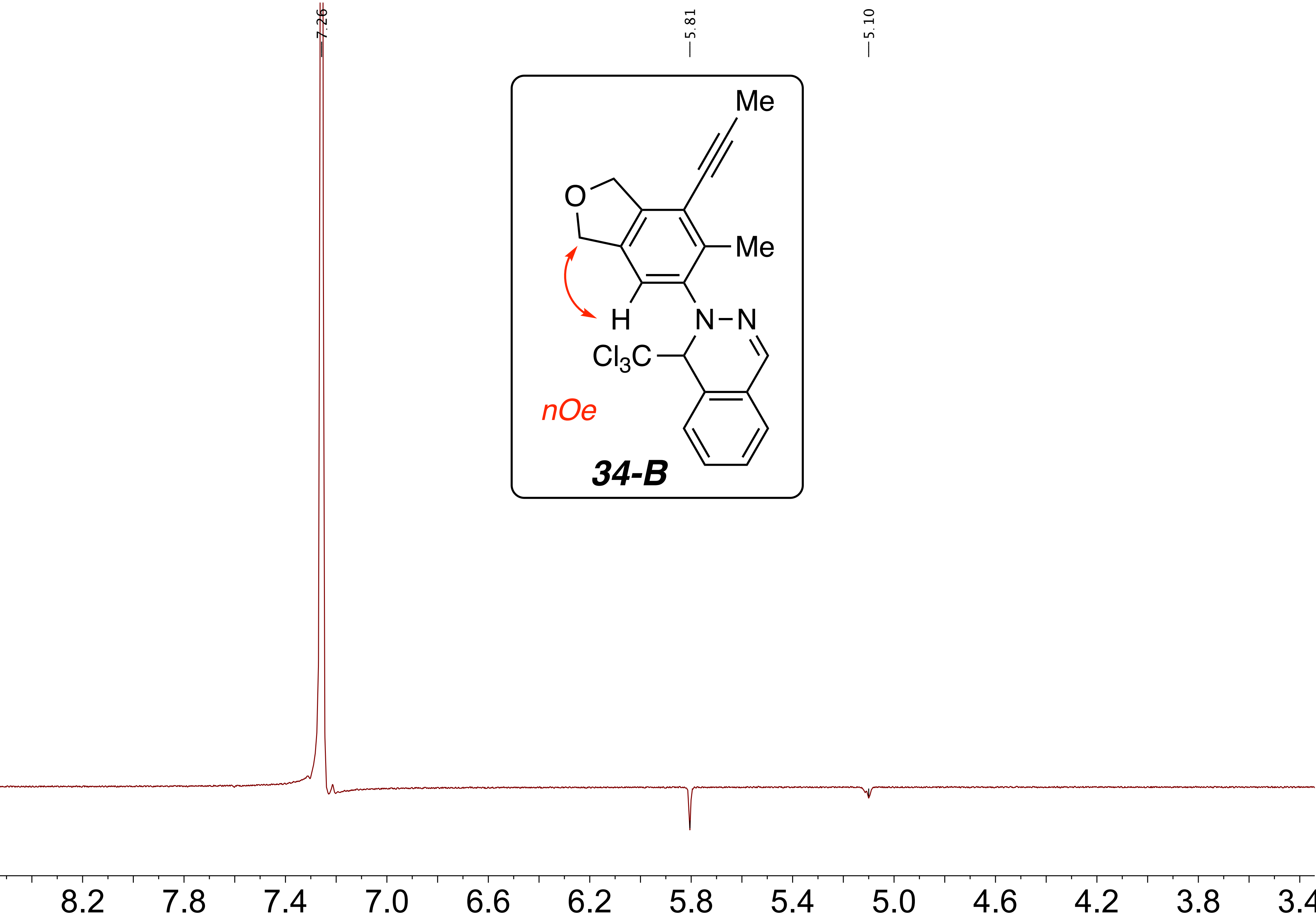
7.26
5.81
5.10
Me
O
Me
H
N
N
Cl3C
nOe
34-B
8.2
7.8
7.4
7.0
6.6
6.2
5.8
5.4
5.0
4.6
4.2
3.8
3.4

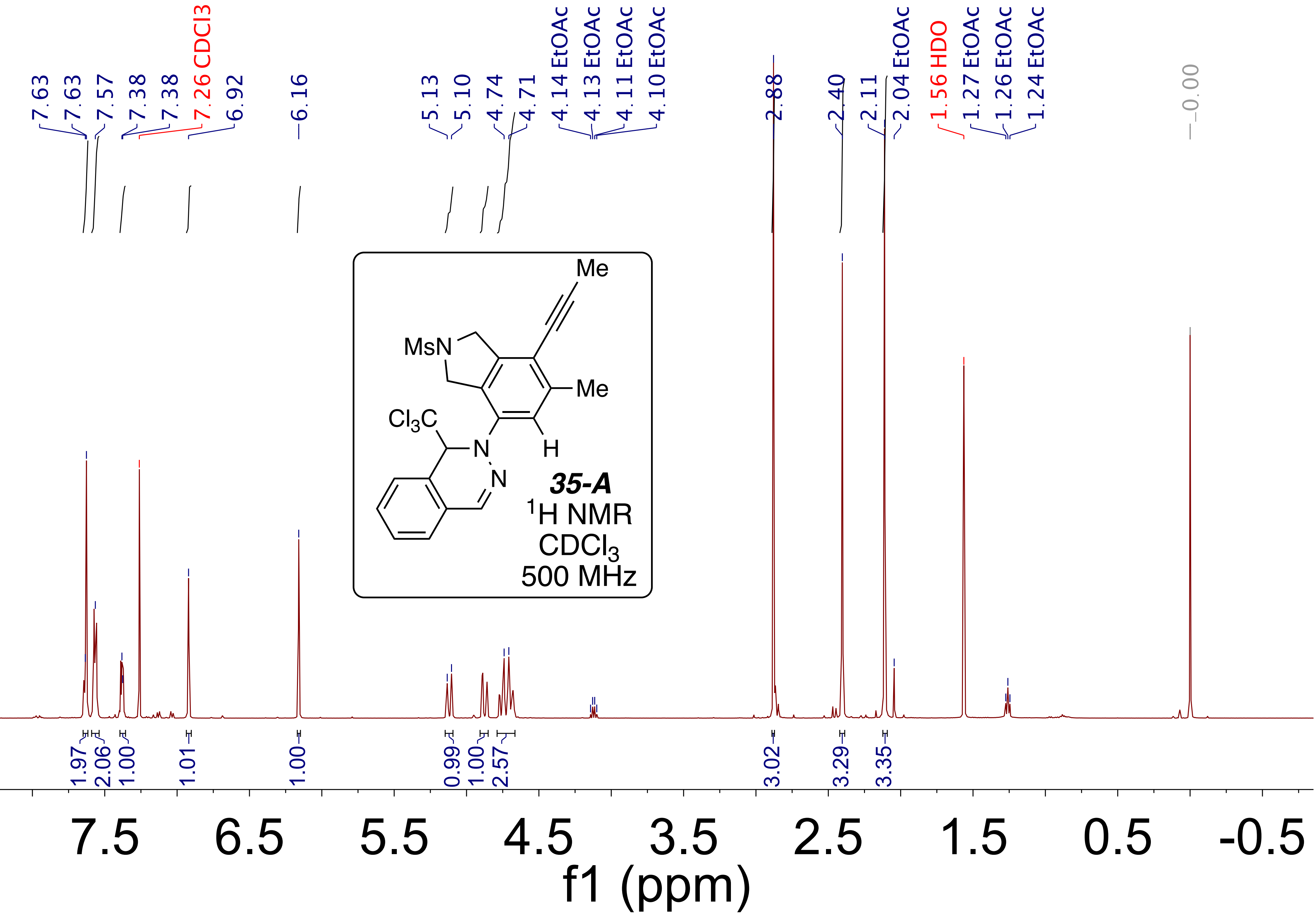

7.63
7.63
7.57
7.38
7.38
7.26 CDCl3
6.92
6.16
5.13
5.10
4.74
4.71
4.14 EtOAc
4.13 EtOAc
4.11 EtOAc
4.10 EtOAc
2.88
2.40
2.11
2.04 EtOAc
1.56 HDO
1.27 EtOAc
1.26 EtOAc
1.24 EtOAc
0.00
MsN
Me
Me
Cl3C
N
N
H
35-A
1H NMR
CDCl3
500 MHz
1.97
2.06
1.00
1.01
1.00
0.99
1.00
2.57
3.02
3.29
3.35
7.5
6.5
5.5
4.5
3.5
2.5
1.5
0.5
-0.5
f1 (ppm)

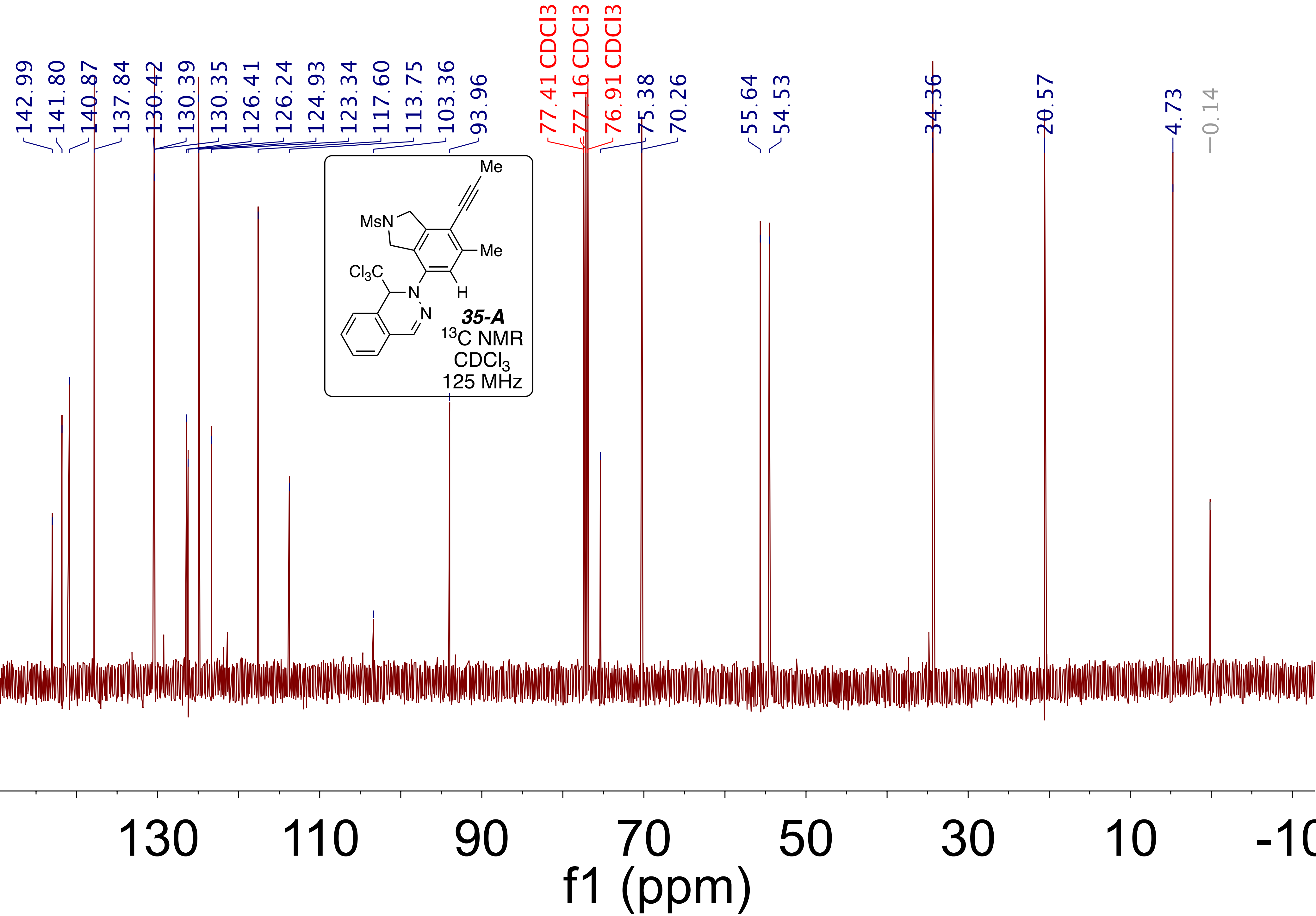
142.99
141.80
140.87
137.84
130.42
130.39
130.35
126.41
126.24
124.93
123.34
117.60
113.75
103.36
93.96
77.41 CDCl3
77.16 CDCl3
76.91 CDCl3
75.38
70.26
55.64
54.53
34.36
20.57
4.73
-0.14
Me
MsN
Me
Cl_3C
N
N
H
35-A
^{13}C NMR
$CDCl_3$
125 MHz
130
110
90
70
50
30
10
-10
f1 (ppm)

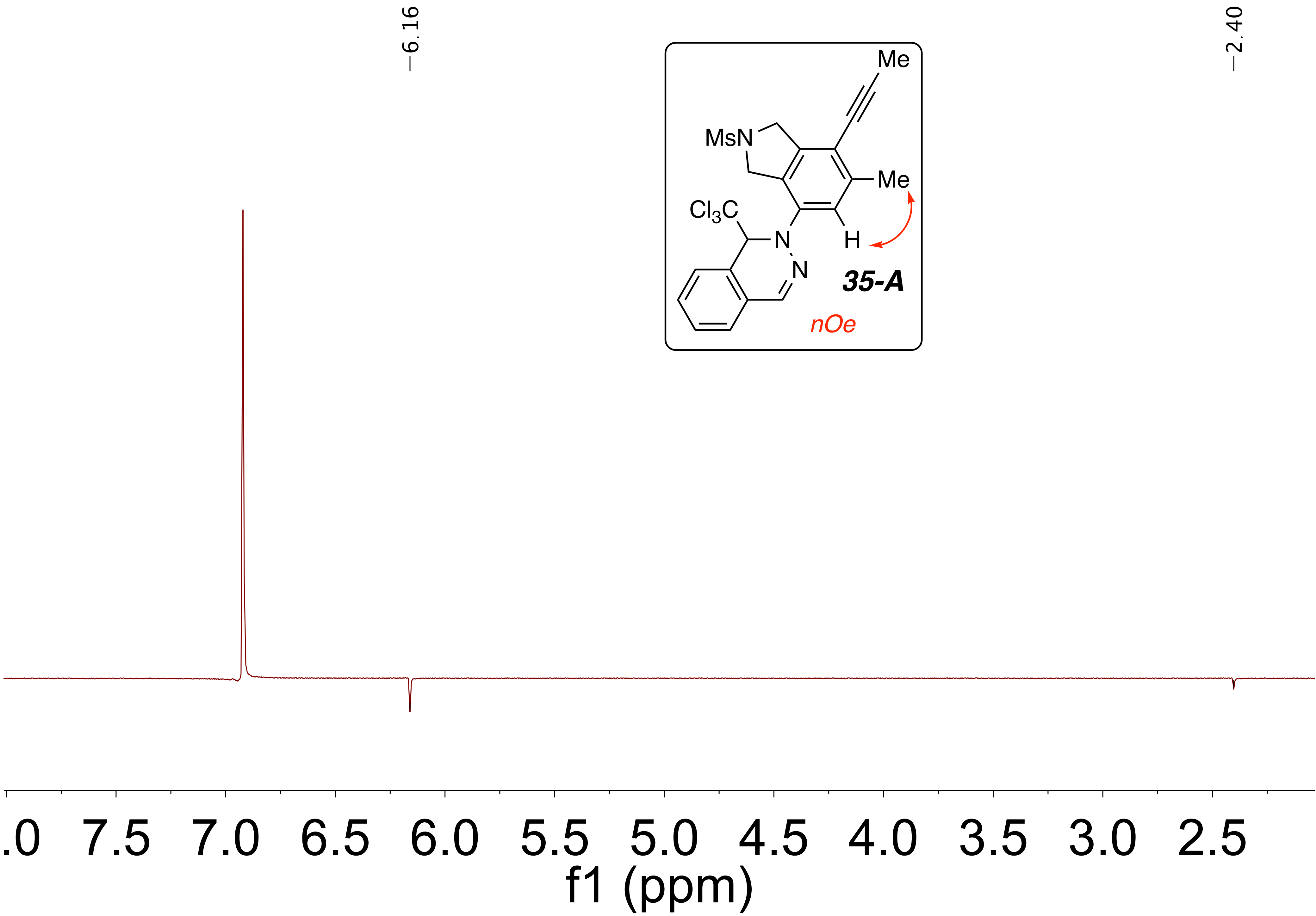
6.16
2.40
Me
MsN
Me
Cl_3C
N
N
H
35-A
nOe
.0
7.5
7.0
6.5
6.0
5.5
5.0
4.5
4.0
3.5
3.0
2.5
f1 (ppm)

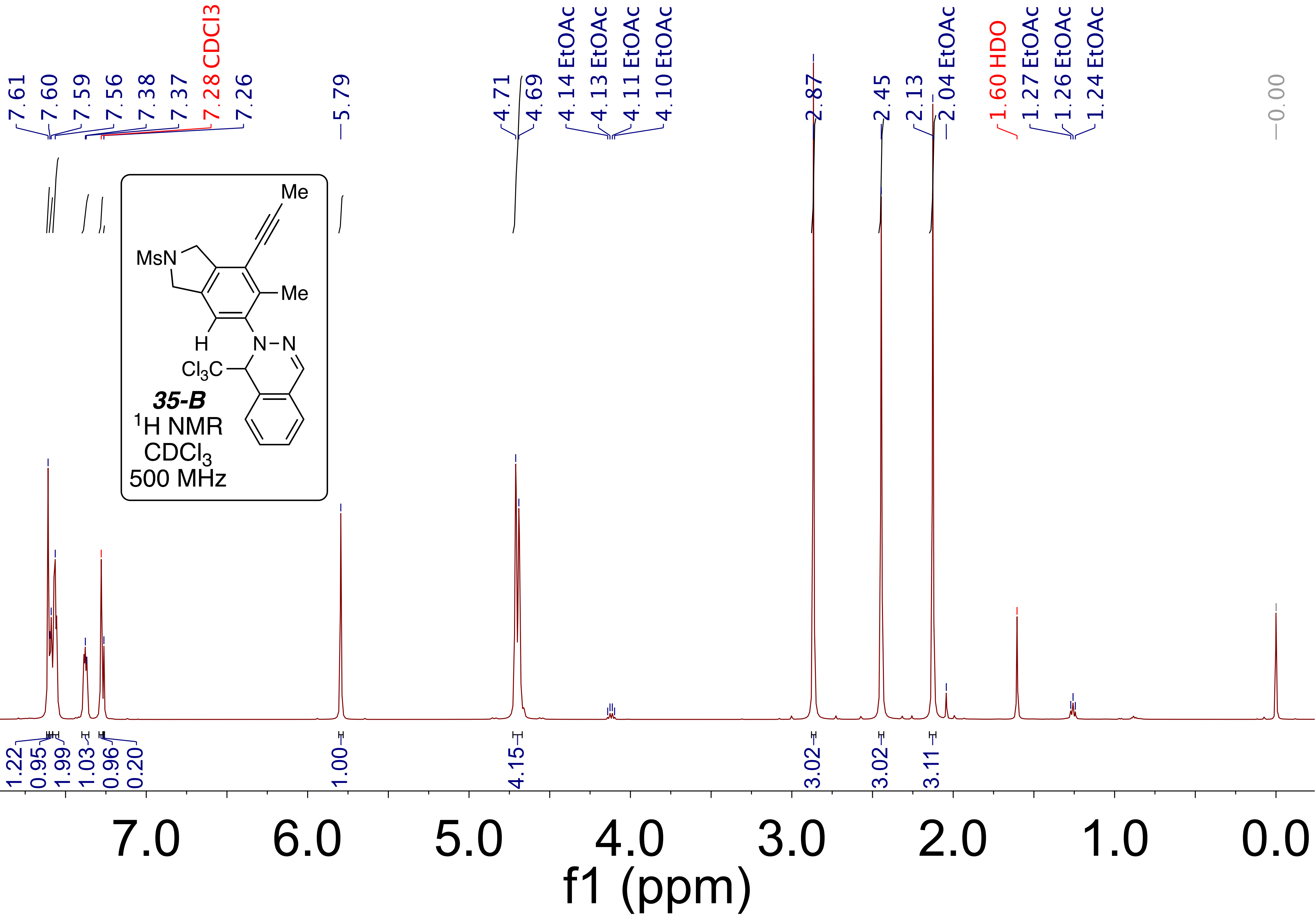
7.61
7.60
7.59
7.56
7.38
7.37
7.28 CDCl3
7.26
5.79
4.71
4.69
4.14 EtOAc
4.13 EtOAc
4.11 EtOAc
4.10 EtOAc
2.87
2.45
2.13
2.04 EtOAc
1.60 HDO
1.27 EtOAc
1.26 EtOAc
1.24 EtOAc
0.00
Me
MsN
Me
H
N–N
Cl_3C
35-B
^1H NMR
$CDCl_3$
500 MHz
1.22
0.95
1.99
1.03
0.96
0.20
1.00
4.15
3.02
3.02
3.11
7.0
6.0
5.0
4.0
3.0
2.0
1.0
0.0
f1 (ppm)

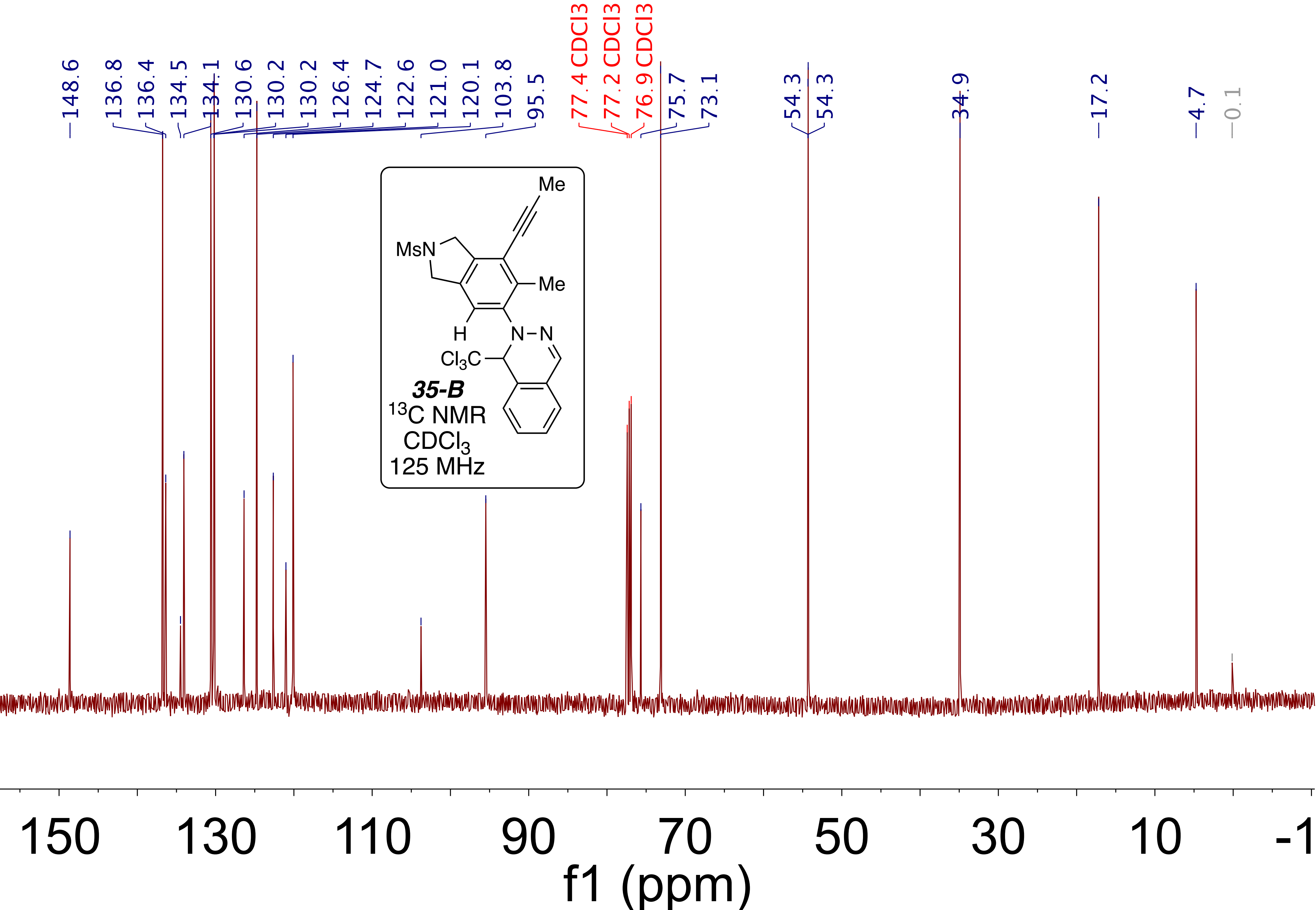
148.6
136.8
136.4
134.5
134.1
130.6
130.2
130.2
126.4
124.7
122.6
121.0
120.1
103.8
95.5
77.4 CDCl3
77.2 CDCl3
76.9 CDCl3
75.7
73.1
54.3
54.3
34.9
17.2
4.7
0.1
Me
MsN
Me
H
N
N
Cl_3C
35-B
^{13}C NMR
$CDCl_3$
125 MHz
150
130
110
90
70
50
30
10
-1
f1 (ppm)

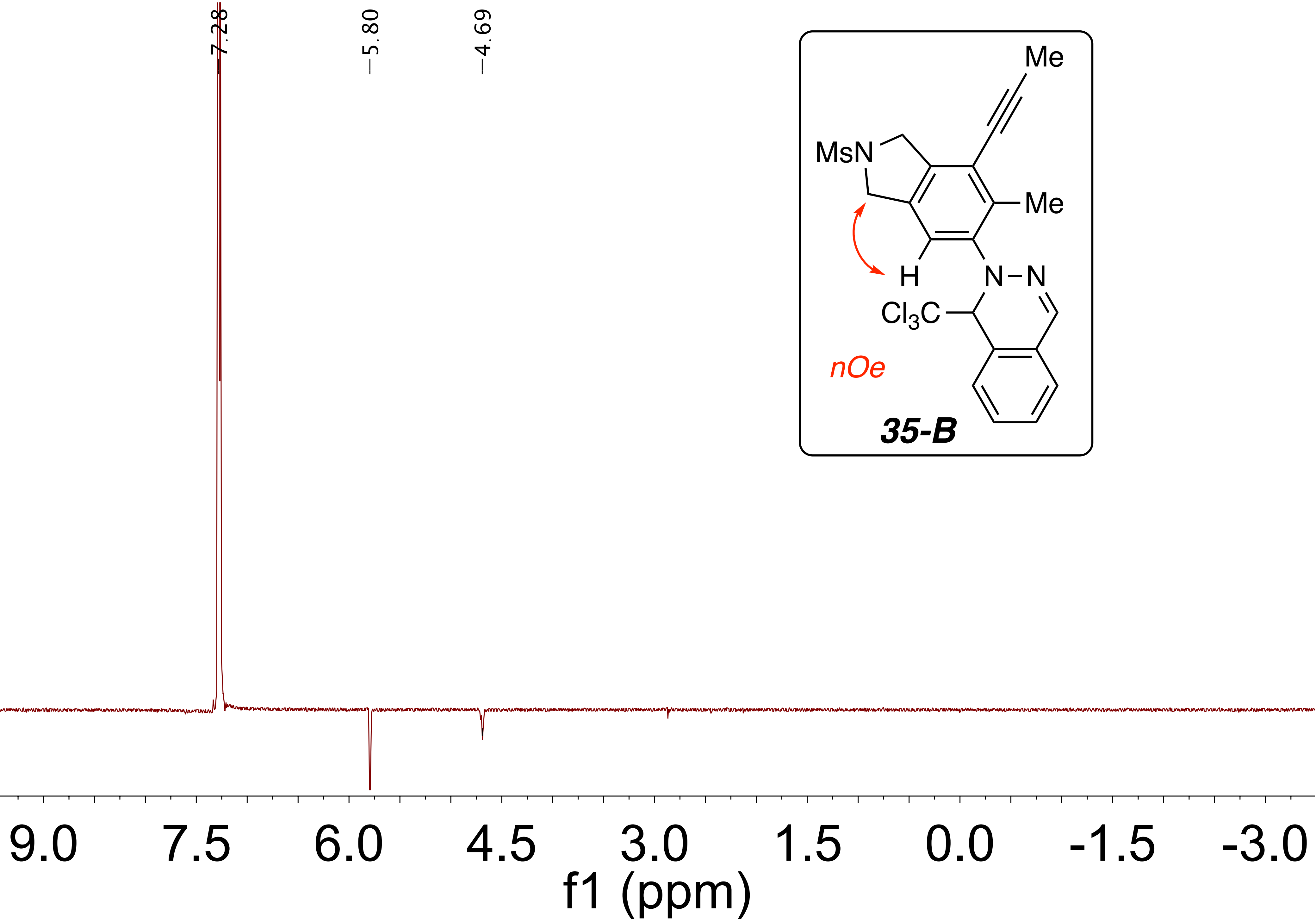
7.28
5.80
4.69
Me
MsN
Me
H
N
N
Cl3C
nOe
35-B
9.0
7.5
6.0
4.5
3.0
1.5
0.0
-1.5
-3.0
f1 (ppm)

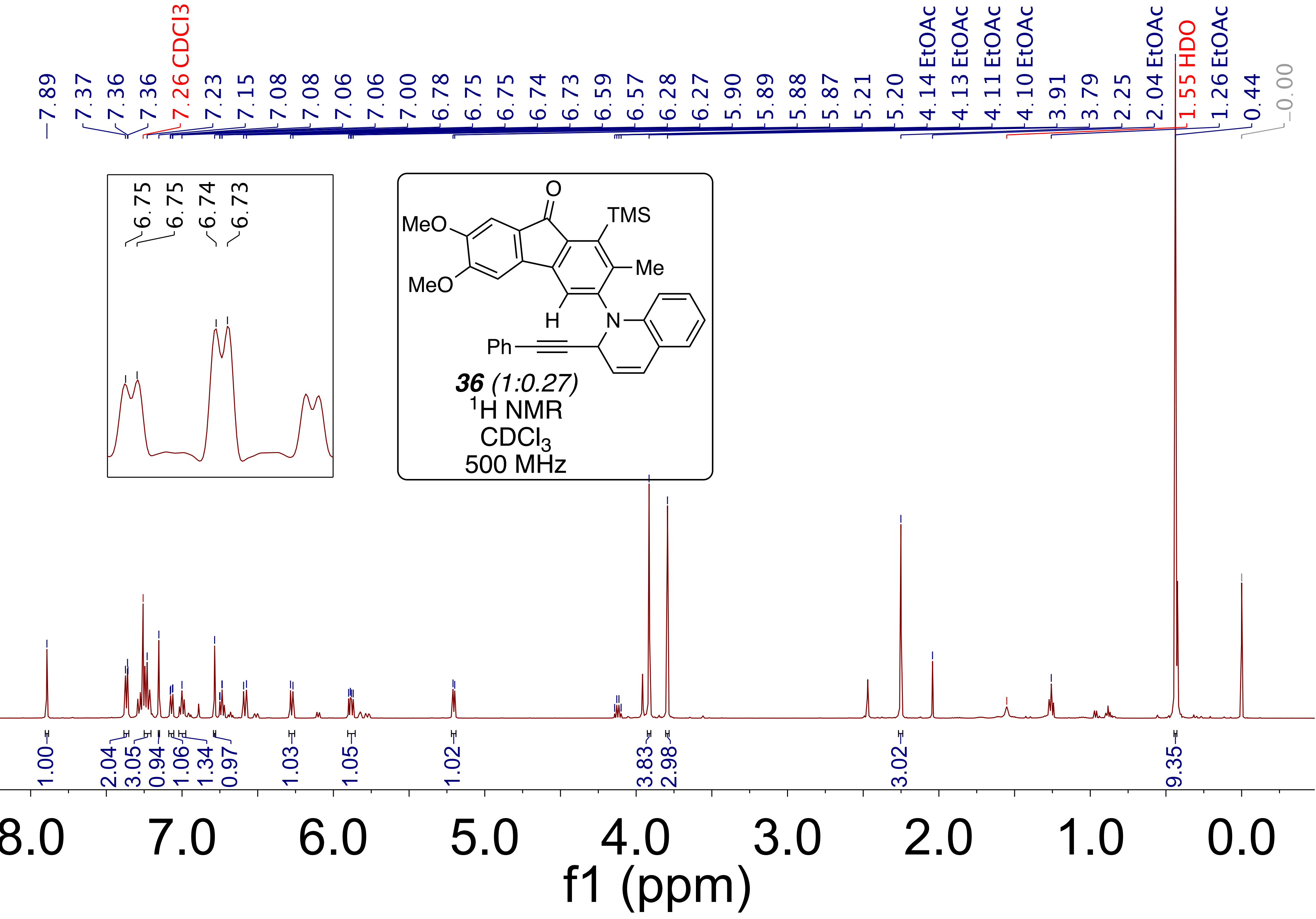
7.89
7.37
7.36
7.36
7.26 CDCl3
7.23
7.15
7.08
7.08
7.06
7.06
7.00
6.78
6.75
6.75
6.74
6.73
6.59
6.57
6.28
6.27
5.90
5.89
5.88
5.87
5.21
5.20
4.14 EtOAc
4.13 EtOAc
4.11 EtOAc
4.10 EtOAc
3.91
3.79
2.25
2.04 EtOAc
1.55 HDO
1.26 EtOAc
0.44
0.00
O
MeO
TMS
Me
MeO
H
N
Ph
36 (1:0.27)
1H NMR
CDCl3
500 MHz
1.00
2.04
3.05
0.94
1.06
1.34
0.97
1.03
1.05
1.02
3.83
2.98
3.02
9.35
8.0
7.0
6.0
5.0
4.0
3.0
2.0
1.0
0.0
f1 (ppm)

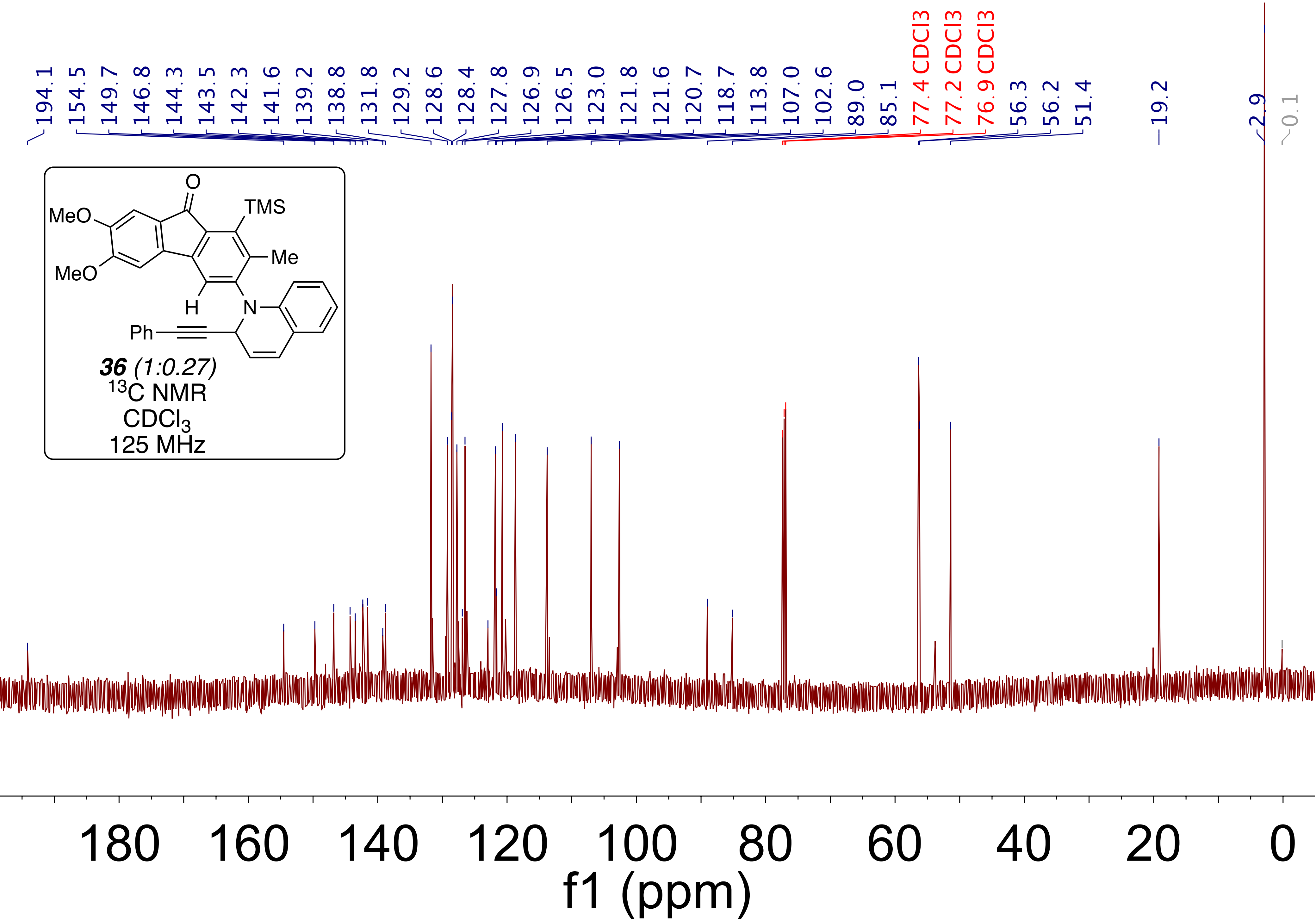
194.1
154.5
149.7
146.8
144.3
143.5
142.3
141.6
139.2
138.8
131.8
129.2
128.6
128.4
127.8
126.9
126.5
123.0
121.8
121.6
120.7
118.7
113.8
107.0
102.6
89.0
85.1
77.4 CDCl3
77.2 CDCl3
76.9 CDCl3
56.3
56.2
51.4
19.2
2.9
0.1
O
TMS
MeO
Me
MeO
H
N
Ph
36 (1:0.27)
^{13}C NMR
$CDCl_3$
125 MHz
180
160
140
120
100
80
60
40
20
0
f1 (ppm)

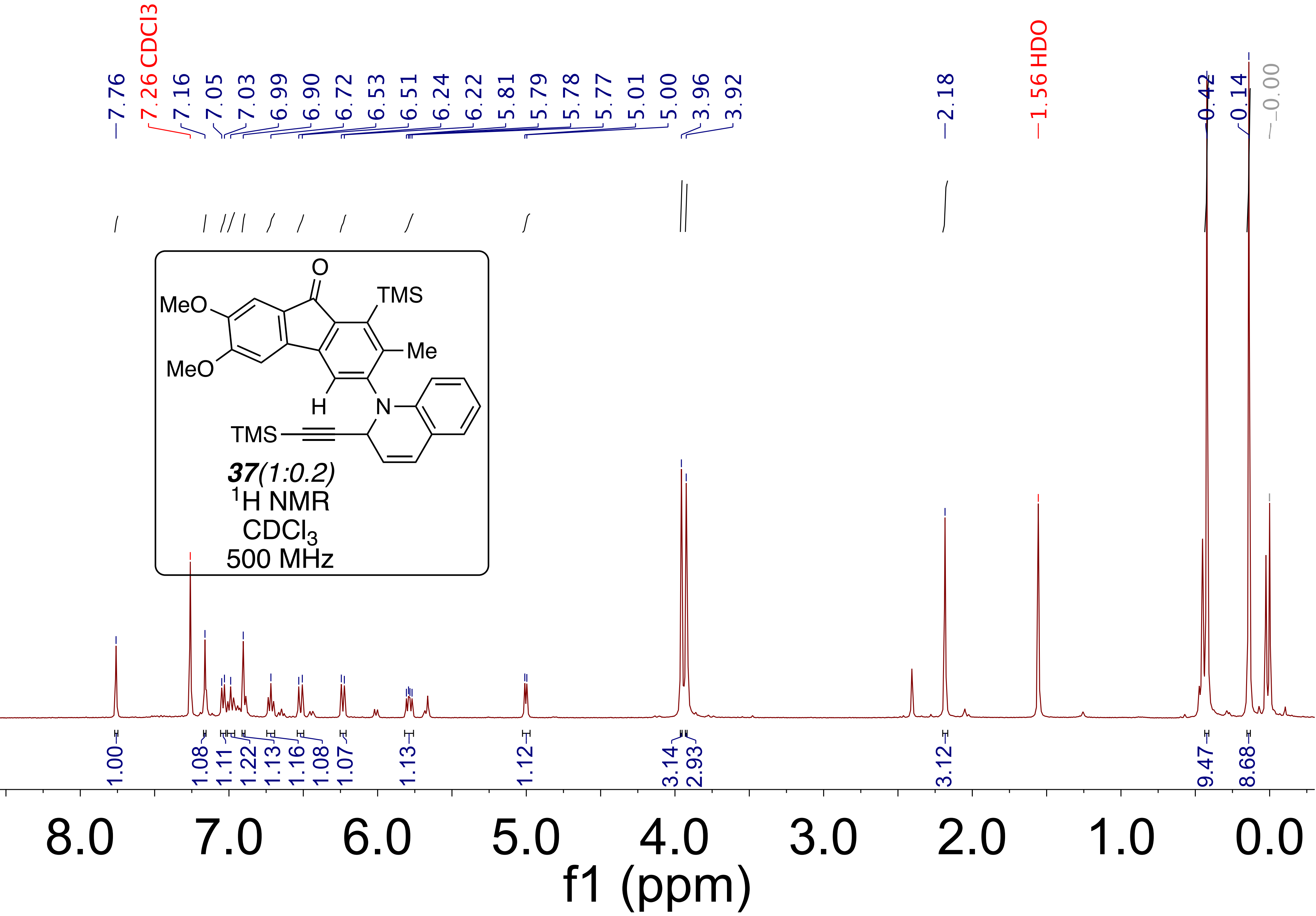
7.76
7.26 CDCl3
7.16
7.05
7.03
6.99
6.90
6.72
6.53
6.51
6.24
6.22
5.81
5.79
5.78
5.77
5.01
5.00
3.96
3.92
2.18
1.56 HDO
0.42
0.14
0.00
MeO
MeO
O
TMS
Me
H
N
TMS
37(1:0.2)
1H NMR
CDCl3
500 MHz
1.00
1.08
1.11
1.22
1.13
1.16
1.08
1.07
1.13
1.12
3.14
2.93
3.12
9.47
8.68
8.0
7.0
6.0
5.0
4.0
3.0
2.0
1.0
0.0
f1 (ppm)

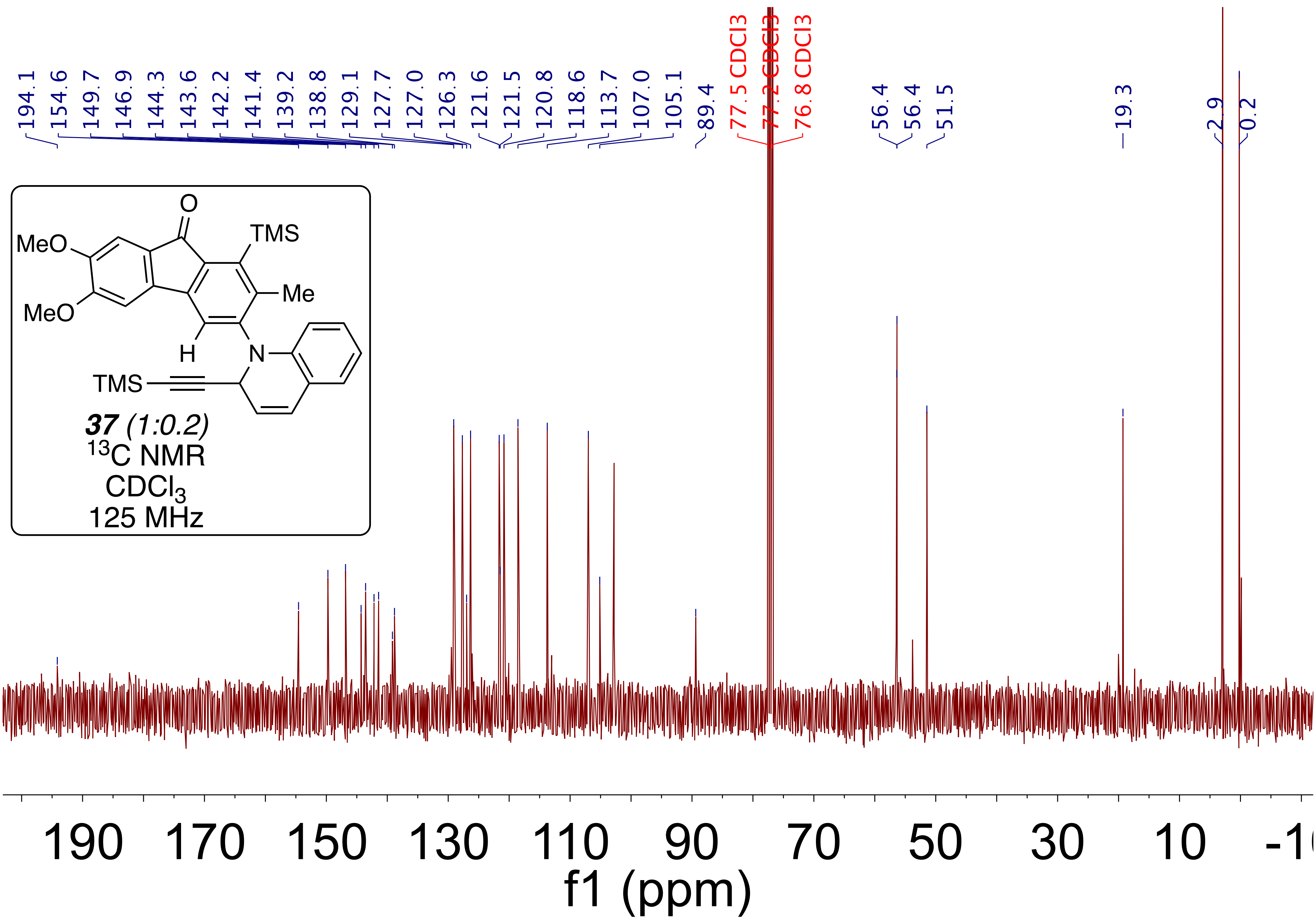
194.1
154.6
149.7
146.9
144.3
143.6
142.2
141.4
139.2
138.8
129.1
127.7
127.0
126.3
121.6
121.5
120.8
118.6
113.7
107.0
105.1
89.4
77.5 CDCl3
77.2 CDCl3
76.8 CDCl3
56.4
56.4
51.5
19.3
2.9
0.2
O
MeO
MeO
TMS
Me
H
N
TMS
37 (1:0.2)
^{13}C NMR
$CDCl_3$
125 MHz
190
170
150
130
110
90
70
50
30
10
f1 (ppm)

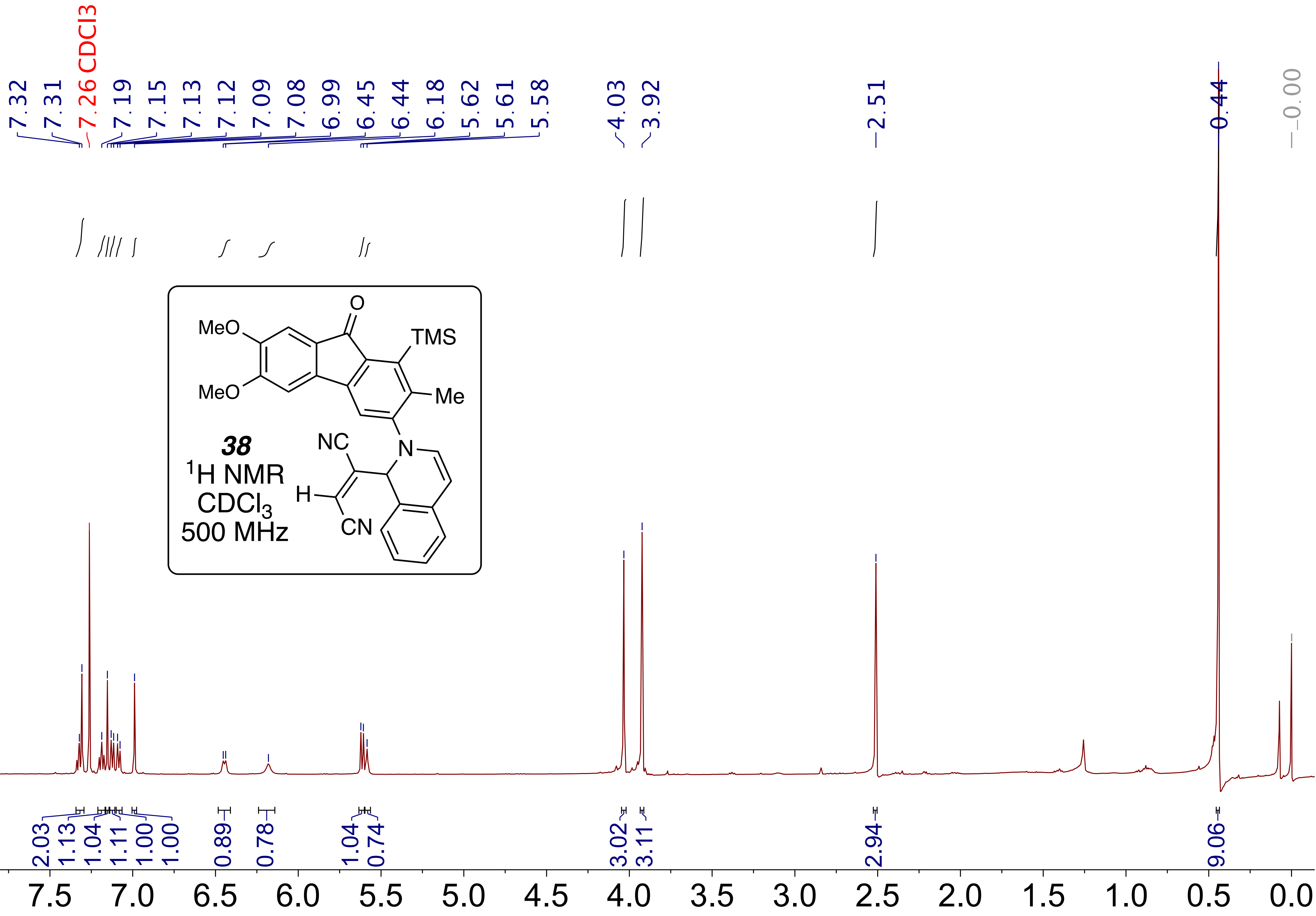
7.32
7.31
7.26 CDCl3
7.19
7.15
7.13
7.12
7.09
7.08
6.99
6.45
6.44
6.18
5.62
5.61
5.58
4.03
3.92
2.51
0.44
-0.00
MeO
MeO
O
TMS
Me
NC
N
H
CN
38
1H NMR
CDCl3
500 MHz
2.03
1.13
1.04
1.11
1.00
1.00
0.89
0.78
1.04
0.74
3.02
3.11
2.94
9.06
7.5
7.0
6.5
6.0
5.5
5.0
4.5
4.0
3.5
3.0
2.5
2.0
1.5
1.0
0.5
0.0

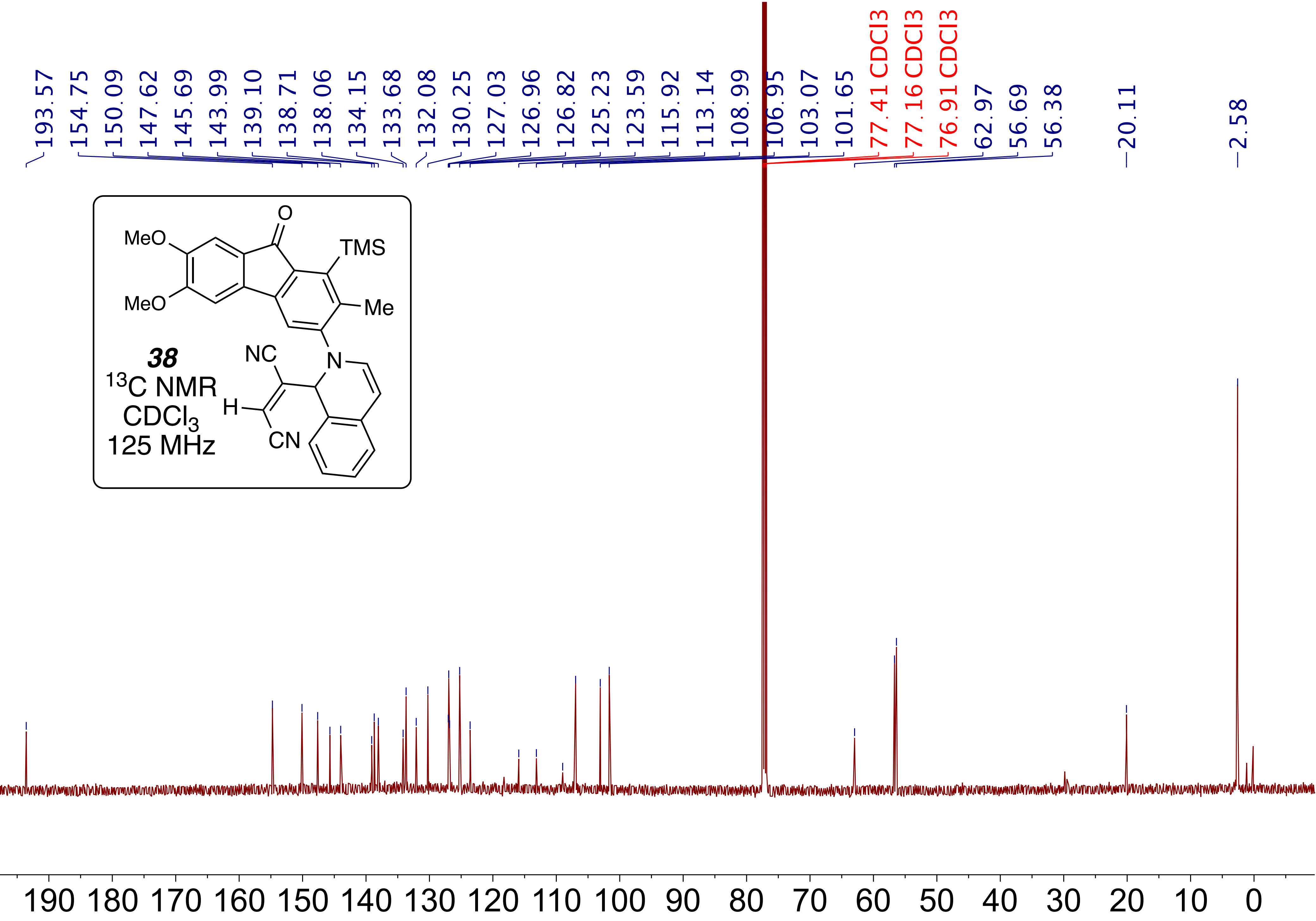
193.57
154.75
150.09
147.62
145.69
143.99
139.10
138.71
138.06
134.15
133.68
132.08
130.25
127.03
126.96
126.82
125.23
123.59
115.92
113.14
108.99
106.95
103.07
101.65
77.41 CDCl3
77.16 CDCl3
76.91 CDCl3
62.97
56.69
56.38
20.11
2.58
MeO
MeO
O
TMS
Me
N
NC
H
CN
38
^{13}C NMR
$CDCl_3$
125 MHz
190 180 170 160 150 140 130 120 110 100 90 80 70 60 50 40 30 20 10 0

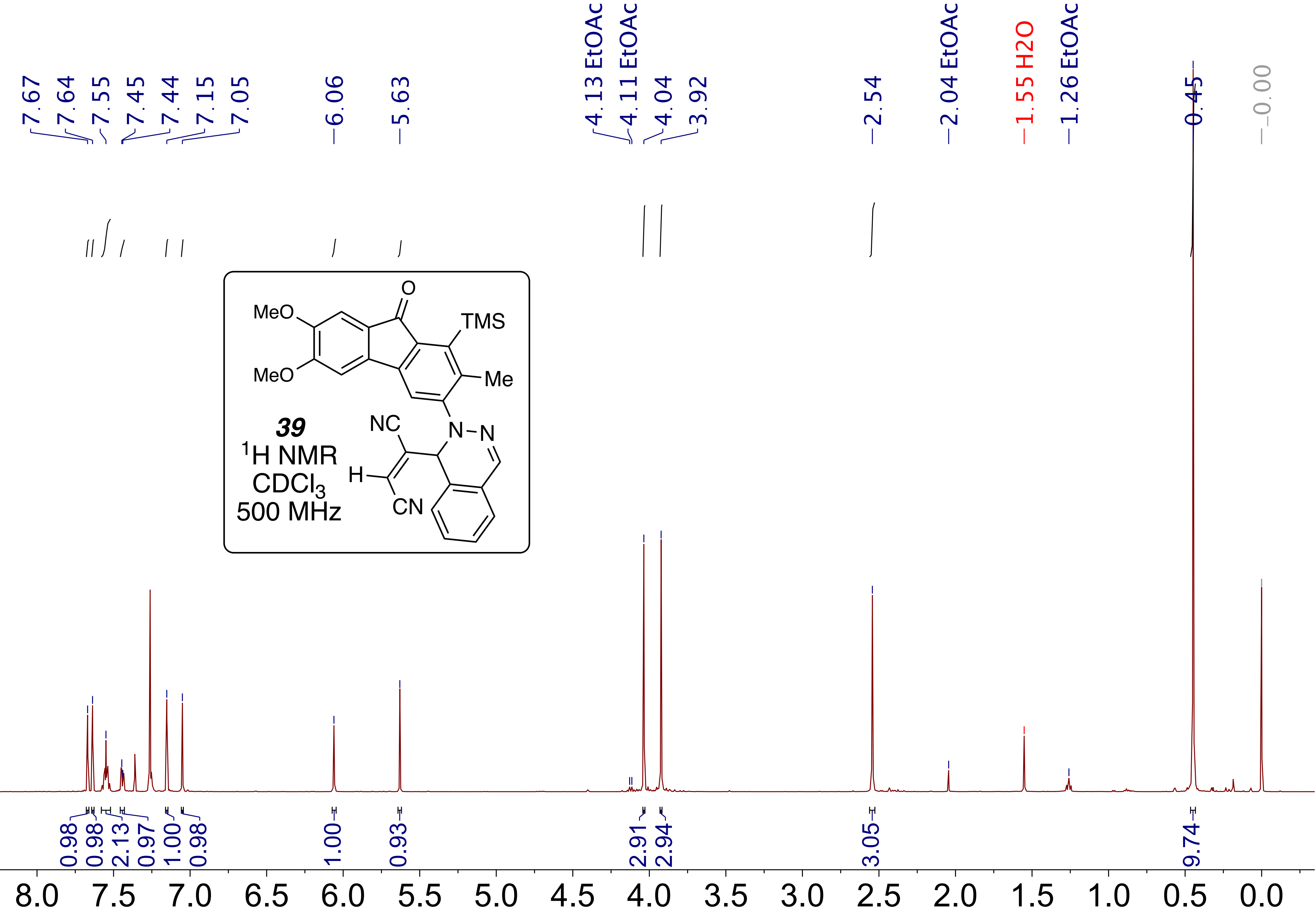
7.67
7.64
7.55
7.45
7.44
7.15
7.05
6.06
5.63
4.13 EtOAc
4.11 EtOAc
4.04
3.92
2.54
2.04 EtOAc
1.55 H2O
1.26 EtOAc
0.45
0.00
MeO
MeO
O
TMS
Me
NC
N–N
H
CN
39
1H NMR
CDCl3
500 MHz
0.98
0.98
2.13
0.97
1.00
0.98
1.00
0.93
2.91
2.94
3.05
9.74
8.0
7.5
7.0
6.5
6.0
5.5
5.0
4.5
4.0
3.5
3.0
2.5
2.0
1.5
1.0
0.5
0.0

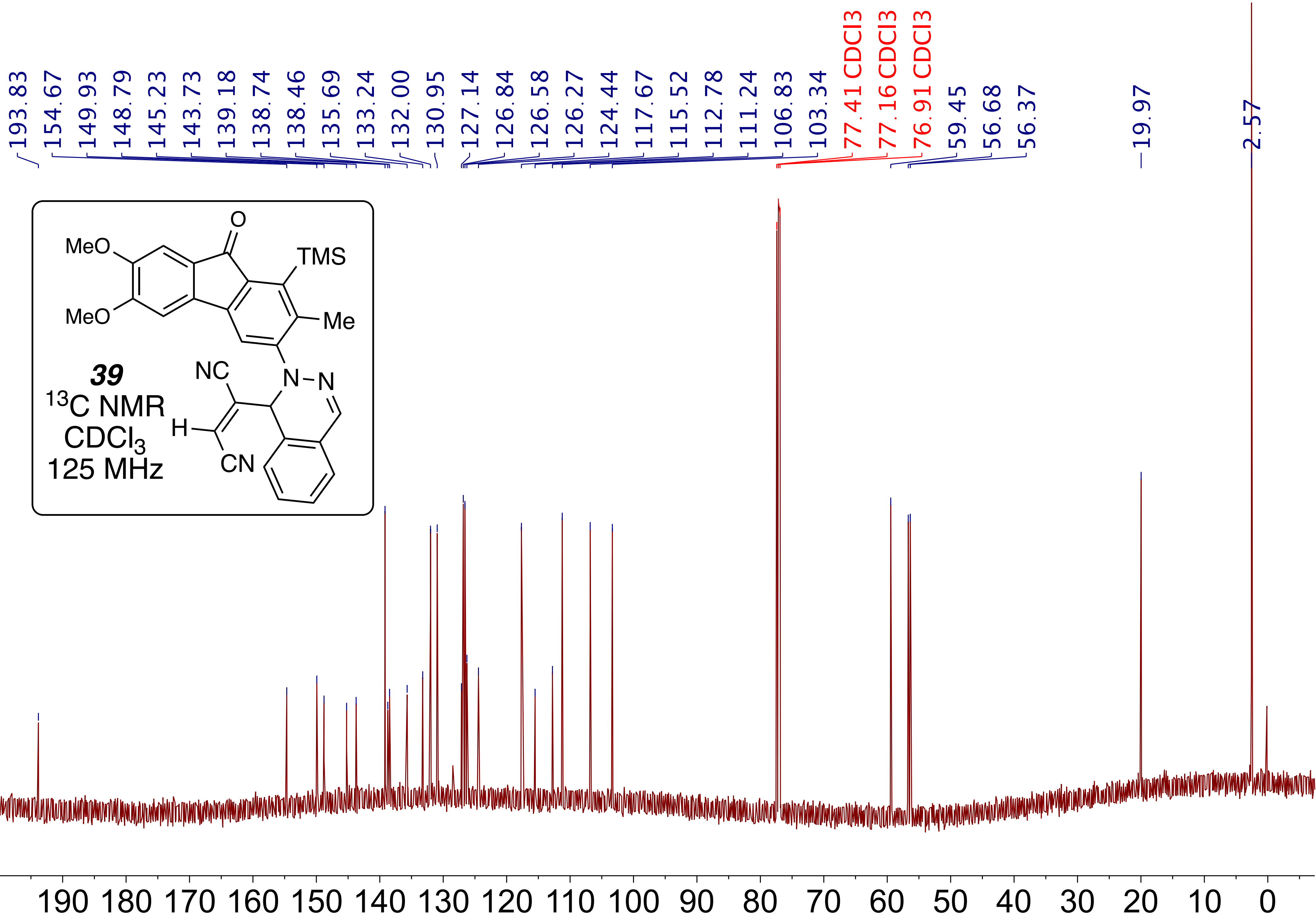
193.83
154.67
149.93
148.79
145.23
143.73
139.18
138.74
138.46
135.69
133.24
132.00
130.95
127.14
126.84
126.58
126.27
124.44
117.67
115.52
112.78
111.24
106.83
103.34
77.41 CDCl3
77.16 CDCl3
76.91 CDCl3
59.45
56.68
56.37
19.97
2.57
O
MeO
MeO
TMS
Me
NC
N–N
H
CN
39
13C NMR
CDCl3
125 MHz
190 180 170 160 150 140 130 120 110 100 90 80 70 60 50 40 30 20 10 0

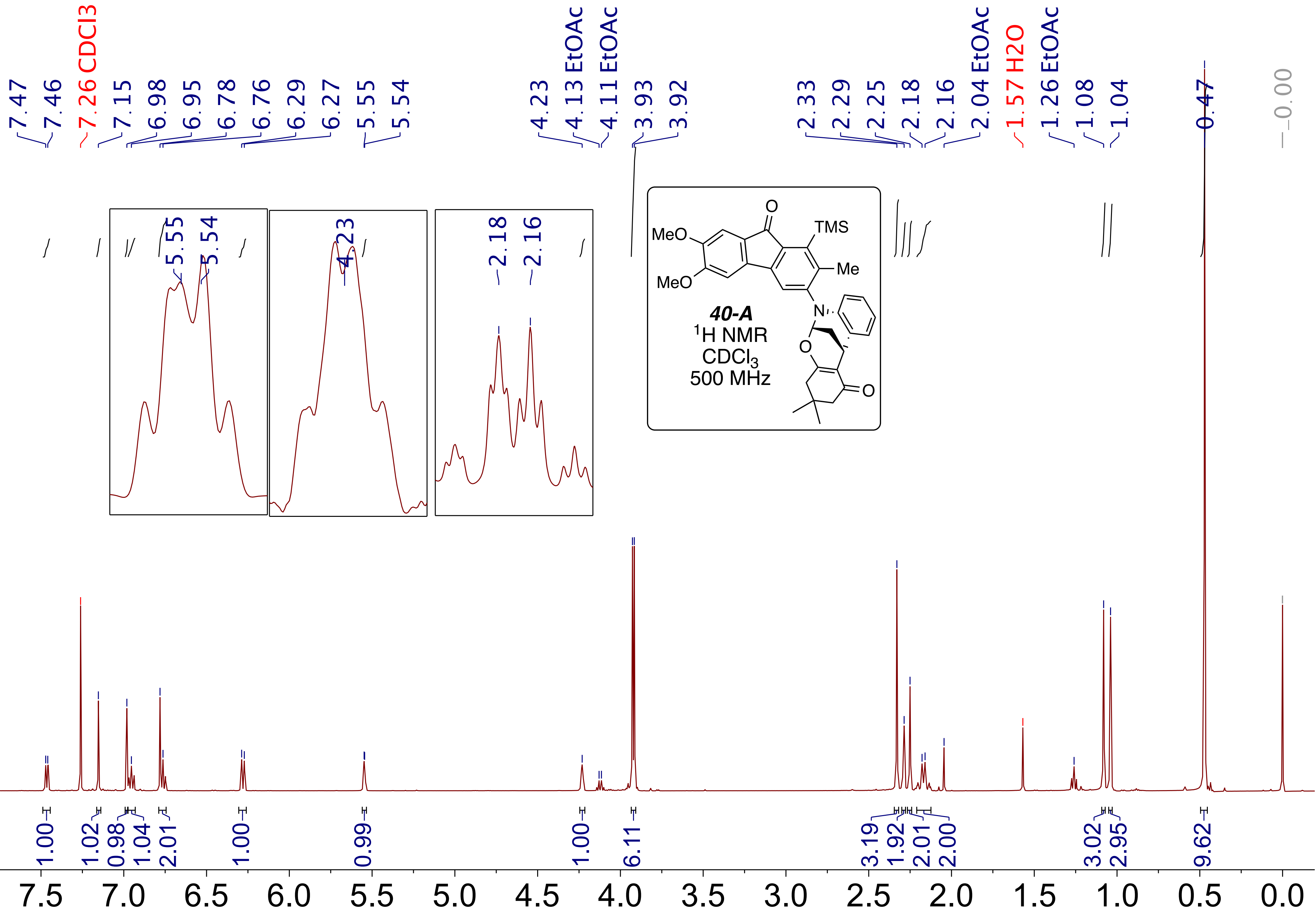
7.47
7.46
7.26 CDCl3
7.15
6.98
6.95
6.78
6.76
6.29
6.27
5.55
5.54
4.23
4.13 EtOAc
4.11 EtOAc
3.93
3.92
2.33
2.29
2.25
2.18
2.16
2.04 EtOAc
1.57 H2O
1.26 EtOAc
1.08
1.04
0.47
0.00
MeO
MeO
O
TMS
Me
N
O
O
40-A
^{1}H NMR
$CDCl_3$
500 MHz
1.00
1.02
0.98
1.04
2.01
1.00
0.99
1.00
6.11
3.19
1.92
2.01
2.00
3.02
2.95
9.62
7.5
7.0
6.5
6.0
5.5
5.0
4.5
4.0
3.5
3.0
2.5
2.0
1.5
1.0
0.5
0.0

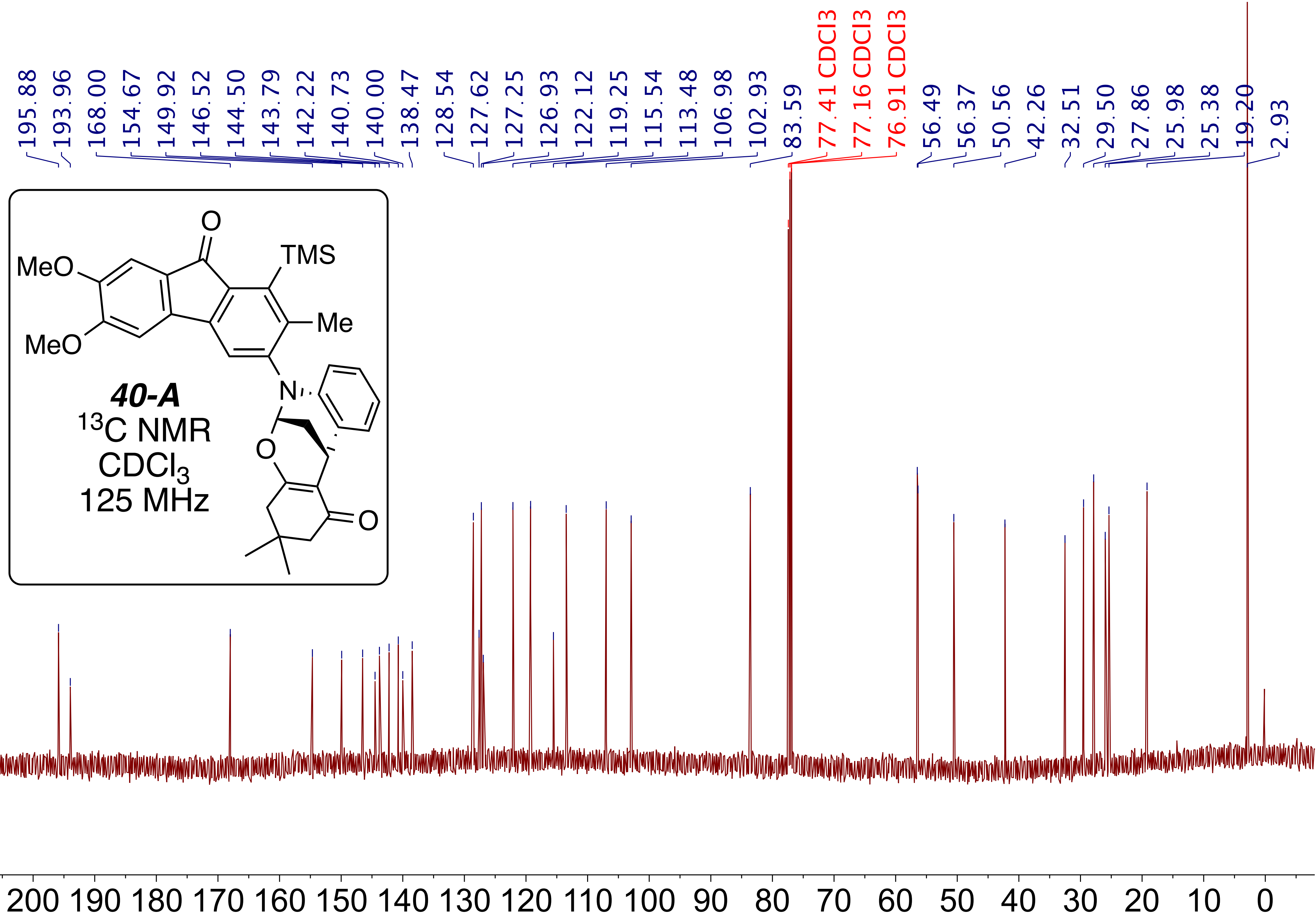
195.88
193.96
168.00
154.67
149.92
146.52
144.50
143.79
142.22
140.73
140.00
138.47
128.54
127.62
127.25
126.93
122.12
119.25
115.54
113.48
106.98
102.93
83.59
77.41 CDCl3
77.16 CDCl3
76.91 CDCl3
56.49
56.37
50.56
42.26
32.51
29.50
27.86
25.98
25.38
19.20
2.93
MeO
MeO
O
TMS
Me
N
O
O
40-A
13C NMR
CDCl3
125 MHz
200 190 180 170 160 150 140 130 120 110 100 90 80 70 60 50 40 30 20 10 0

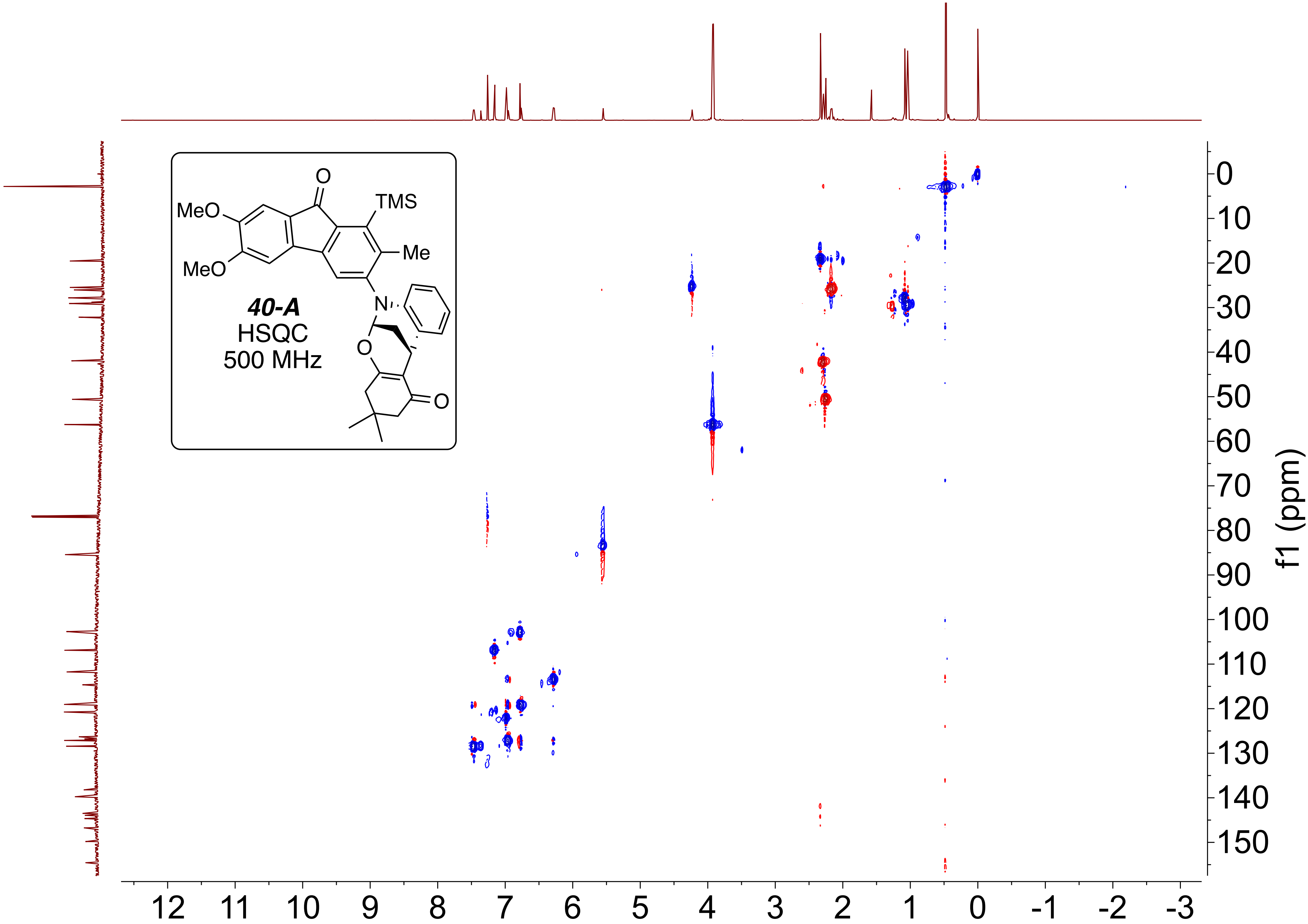
MeO
MeO
O
TMS
Me
N
O
O
40-A
HSQC
500 MHz
f1 (ppm)
0
10
20
30
40
50
60
70
80
90
100
110
120
130
140
150
12
11
10
9
8
7
6
5
4
3
2
1
0
-1
-2
-3

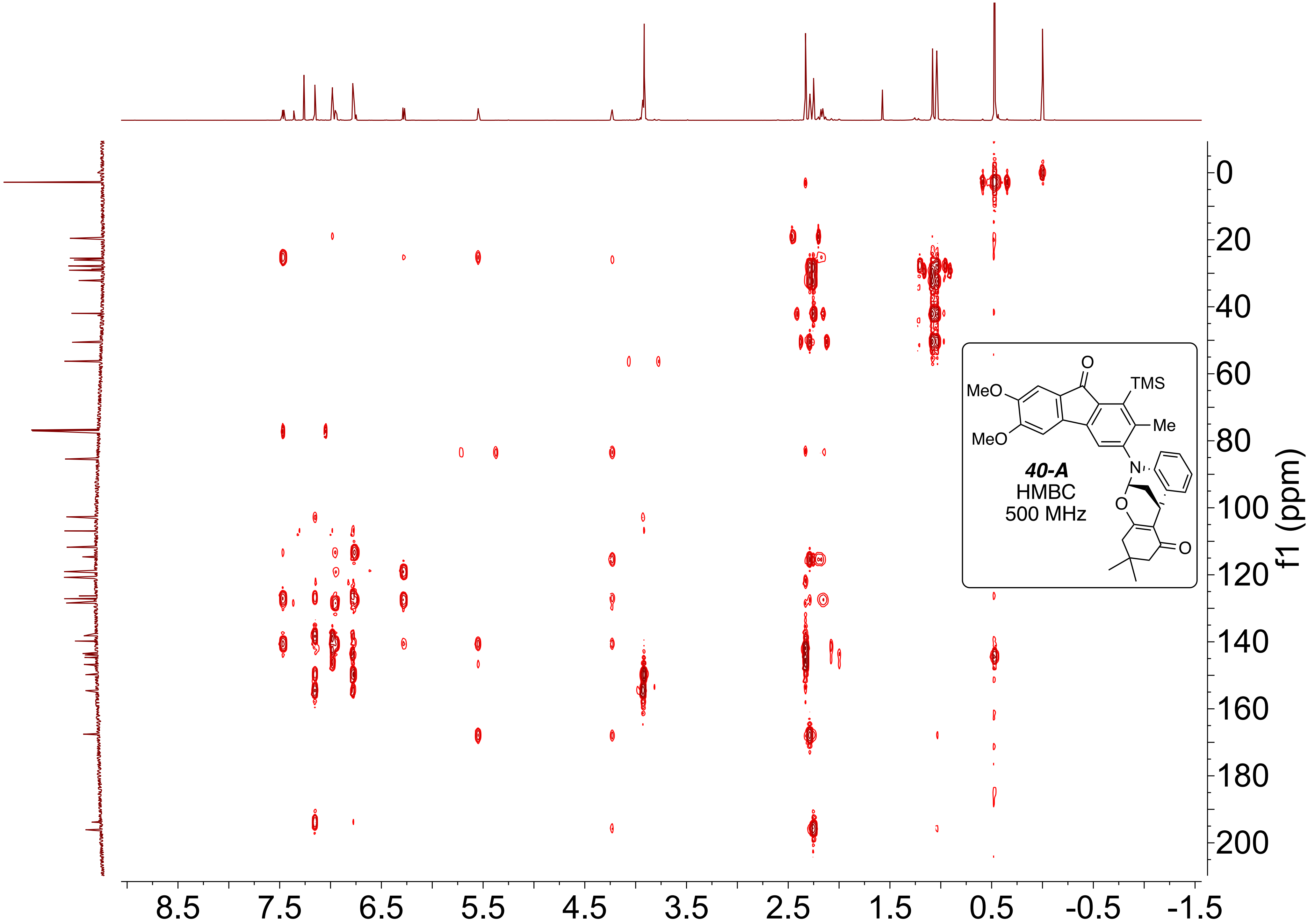
MeO
MeO
O
TMS
Me
N
O
O
40-A
HMBC
500 MHz
f1 (ppm)
0
20
40
60
80
100
120
140
160
180
200
8.5
7.5
6.5
5.5
4.5
3.5
2.5
1.5
0.5
-0.5
-1.5

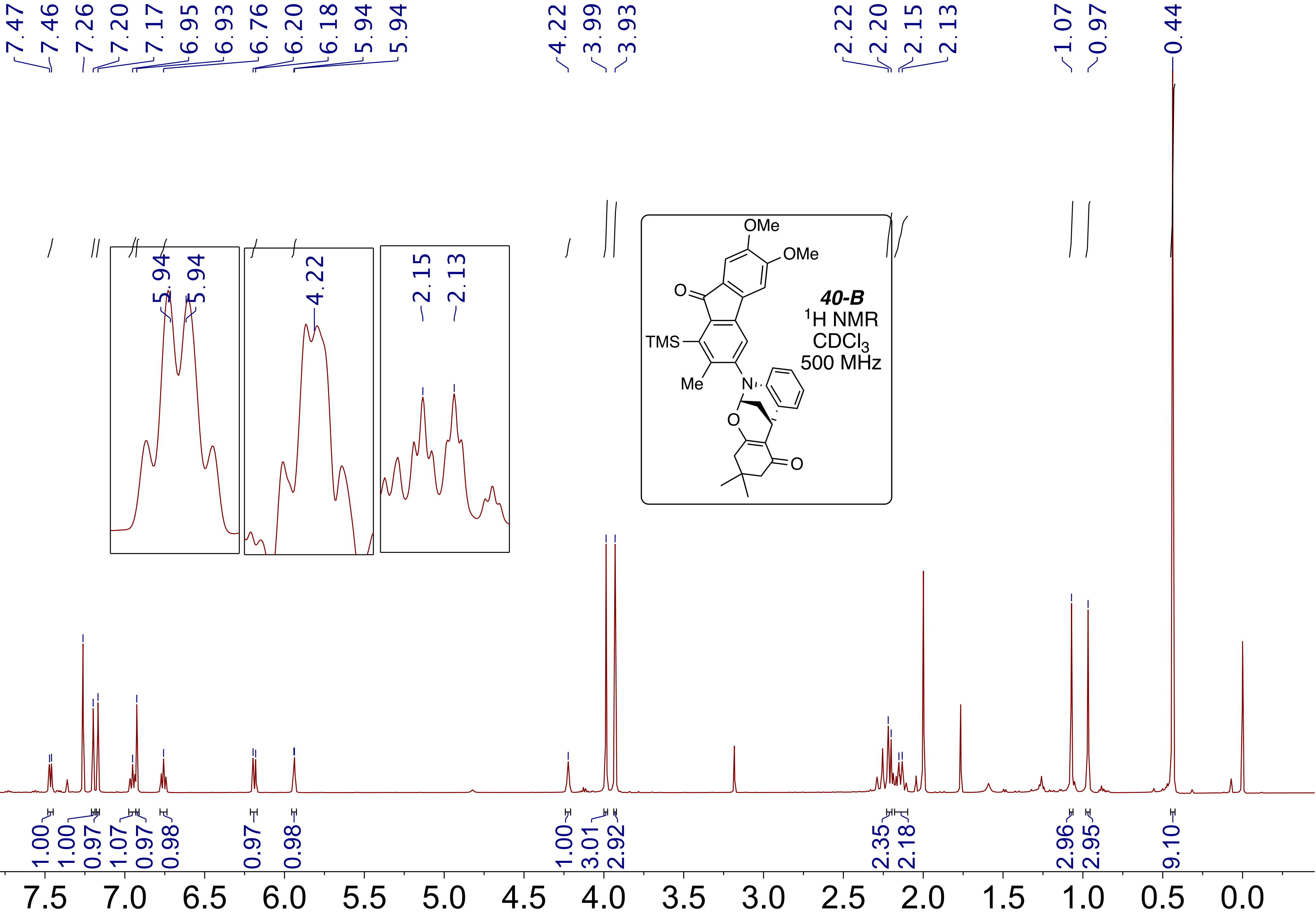
7.47
7.46
7.26
7.20
7.17
6.95
6.93
6.76
6.20
6.18
5.94
5.94
4.22
3.99
3.93
2.22
2.20
2.15
2.13
1.07
0.97
0.44
40-B
1H NMR
CDCl3
500 MHz
OMe
OMe
O
TMS
Me
N
O
O
1.00
1.00
0.97
1.07
0.97
0.98
0.97
0.98
1.00
3.01
2.92
2.35
2.18
2.96
2.95
9.10
7.5
7.0
6.5
6.0
5.5
5.0
4.5
4.0
3.5
3.0
2.5
2.0
1.5
1.0
0.5
0.0

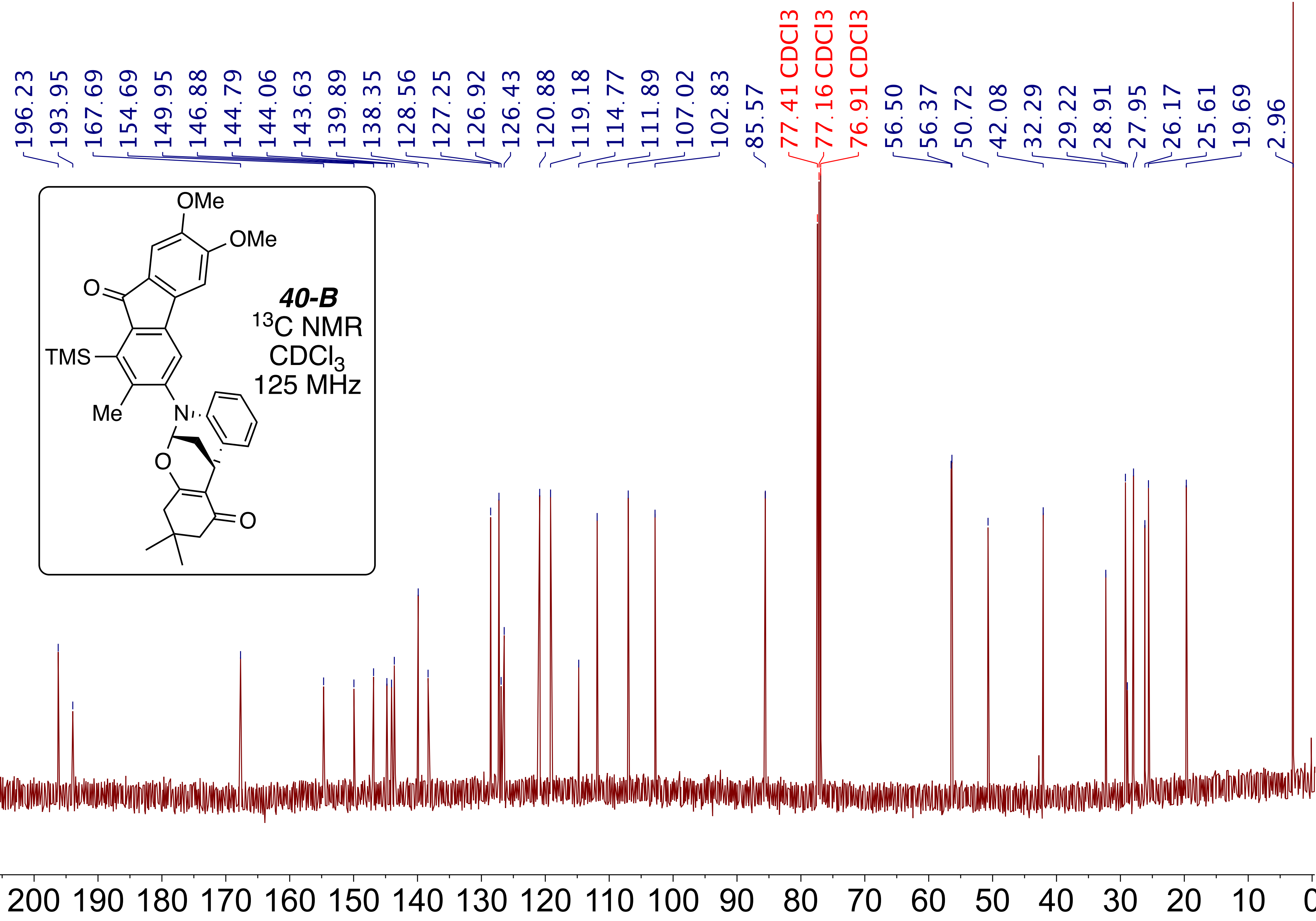
196.23
193.95
167.69
154.69
149.95
146.88
144.79
144.06
143.63
139.89
138.35
128.56
127.25
126.92
126.43
120.88
119.18
114.77
111.89
107.02
102.83
85.57
77.41 CDCl3
77.16 CDCl3
76.91 CDCl3
56.50
56.37
50.72
42.08
32.29
29.22
28.91
27.95
26.17
25.61
19.69
2.96
OMe
OMe
O
TMS
Me
N
O
O
40-B
^{13}C NMR
$CDCl_3$
125 MHz
200 190 180 170 160 150 140 130 120 110 100 90 80 70 60 50 40 30 20 10 0

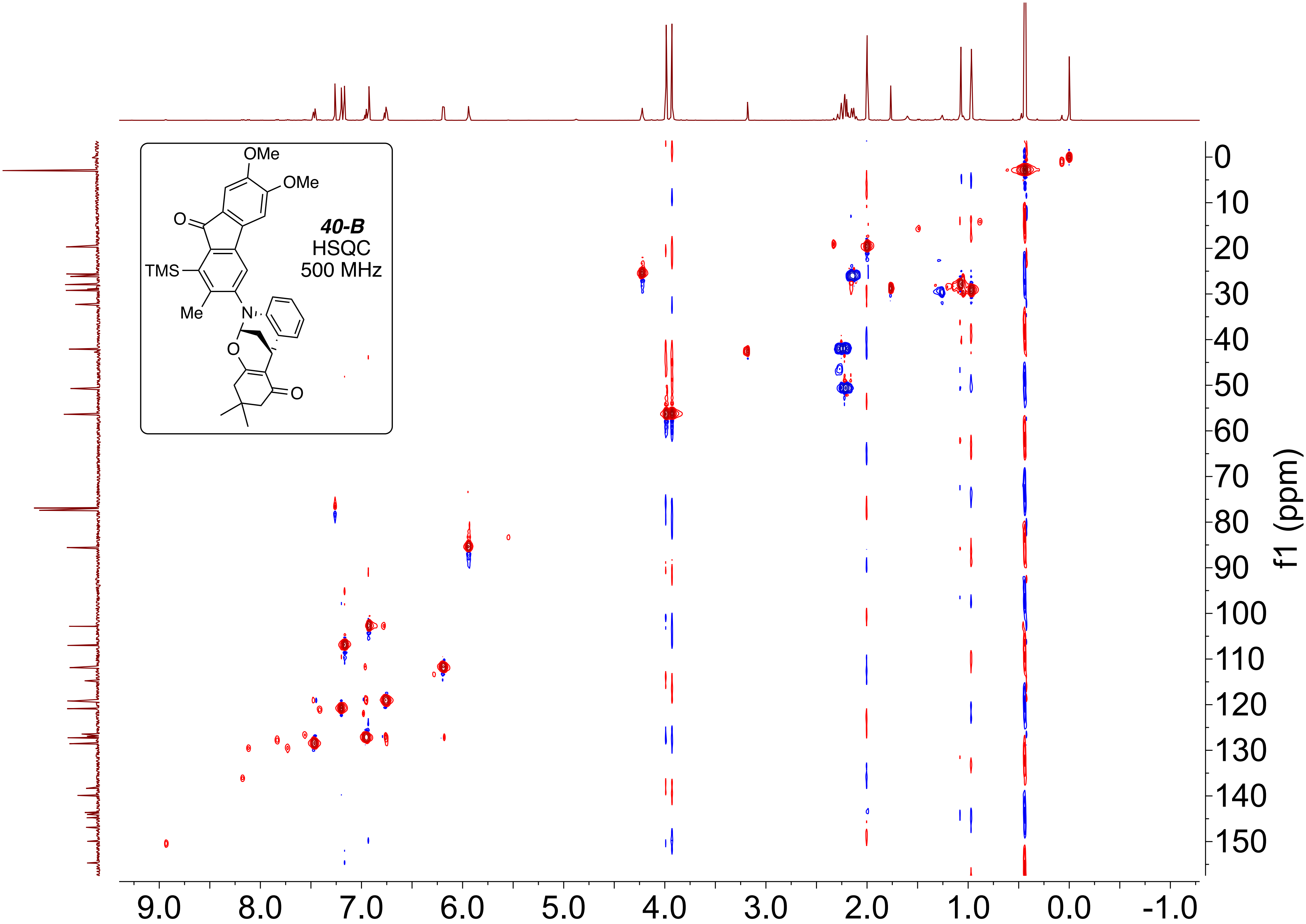
OMe
OMe
O
TMS
Me
N
O
O
40-B
HSQC
500 MHz
f1 (ppm)
0
10
20
30
40
50
60
70
80
90
100
110
120
130
140
150
9.0
8.0
7.0
6.0
5.0
4.0
3.0
2.0
1.0
0.0
-1.0

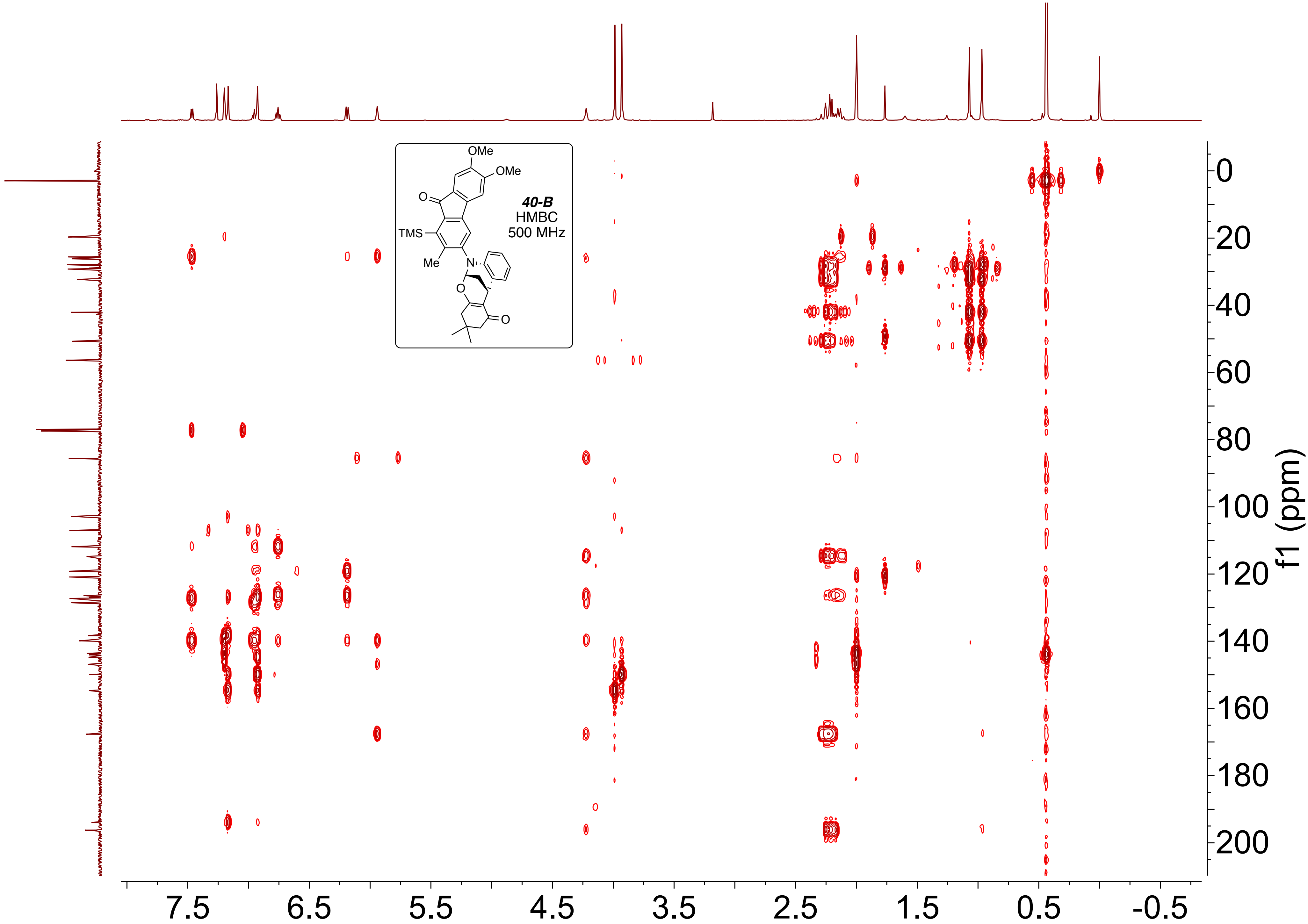
OMe
OMe
O
TMS
Me
N
O
O
40-B
HMBC
500 MHz
f1 (ppm)
0
20
40
60
80
100
120
140
160
180
200
7.5
6.5
5.5
4.5
3.5
2.5
1.5
0.5
-0.5

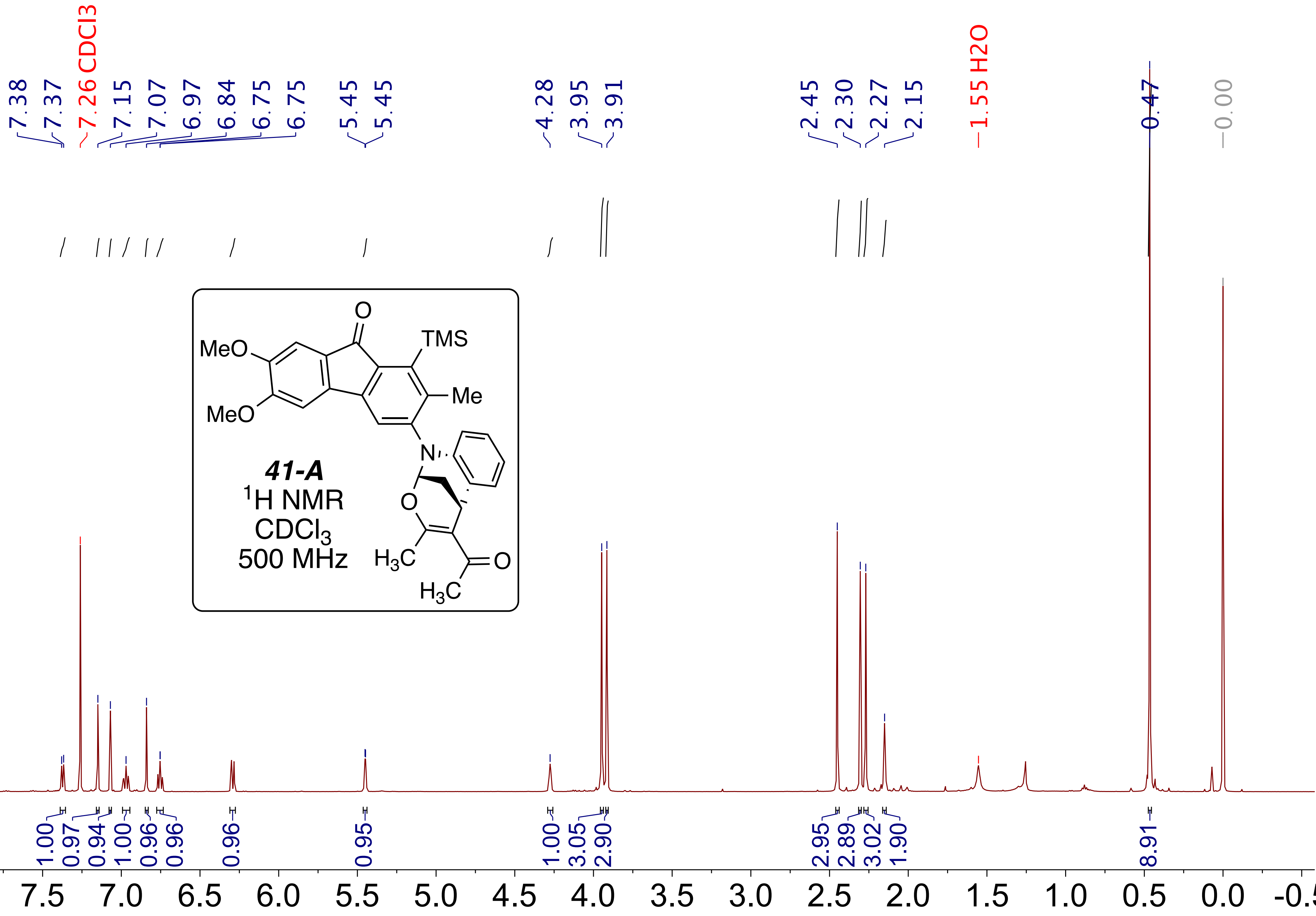
7.38
7.37
7.26 CDCl3
7.15
7.07
6.97
6.84
6.75
6.75
5.45
5.45
4.28
3.95
3.91
2.45
2.30
2.27
2.15
1.55 H2O
0.47
0.00
MeO
MeO
O
TMS
Me
N
O
H_3C
O
H_3C
41-A
^{1}H NMR
$CDCl_3$
500 MHz
1.00
0.97
0.94
1.00
0.96
0.96
0.96
0.95
1.00
3.05
2.90
2.95
2.89
3.02
1.90
8.91
7.5
7.0
6.5
6.0
5.5
5.0
4.5
4.0
3.5
3.0
2.5
2.0
1.5
1.0
0.5
0.0

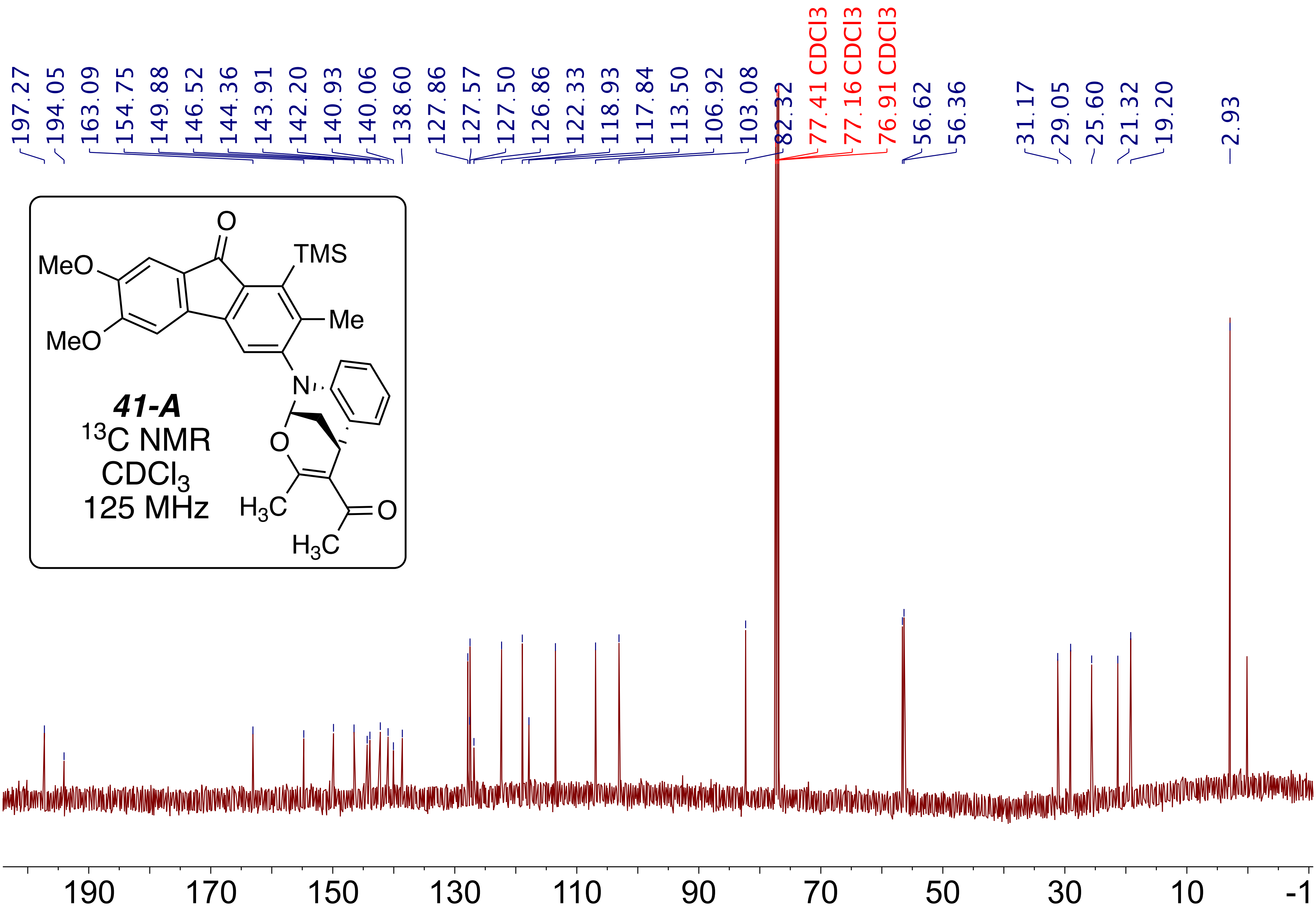
197.27
194.05
163.09
154.75
149.88
146.52
144.36
143.91
142.20
140.93
140.06
138.60
127.86
127.57
127.50
126.86
122.33
118.93
117.84
113.50
106.92
103.08
82.32
77.41 CDCl3
77.16 CDCl3
76.91 CDCl3
56.62
56.36
31.17
29.05
25.60
21.32
19.20
2.93
MeO
MeO
O
TMS
Me
N
O
H3C
H3C
O
41-A
13C NMR
CDCl3
125 MHz
190
170
150
130
110
90
70
50
30
10
-1

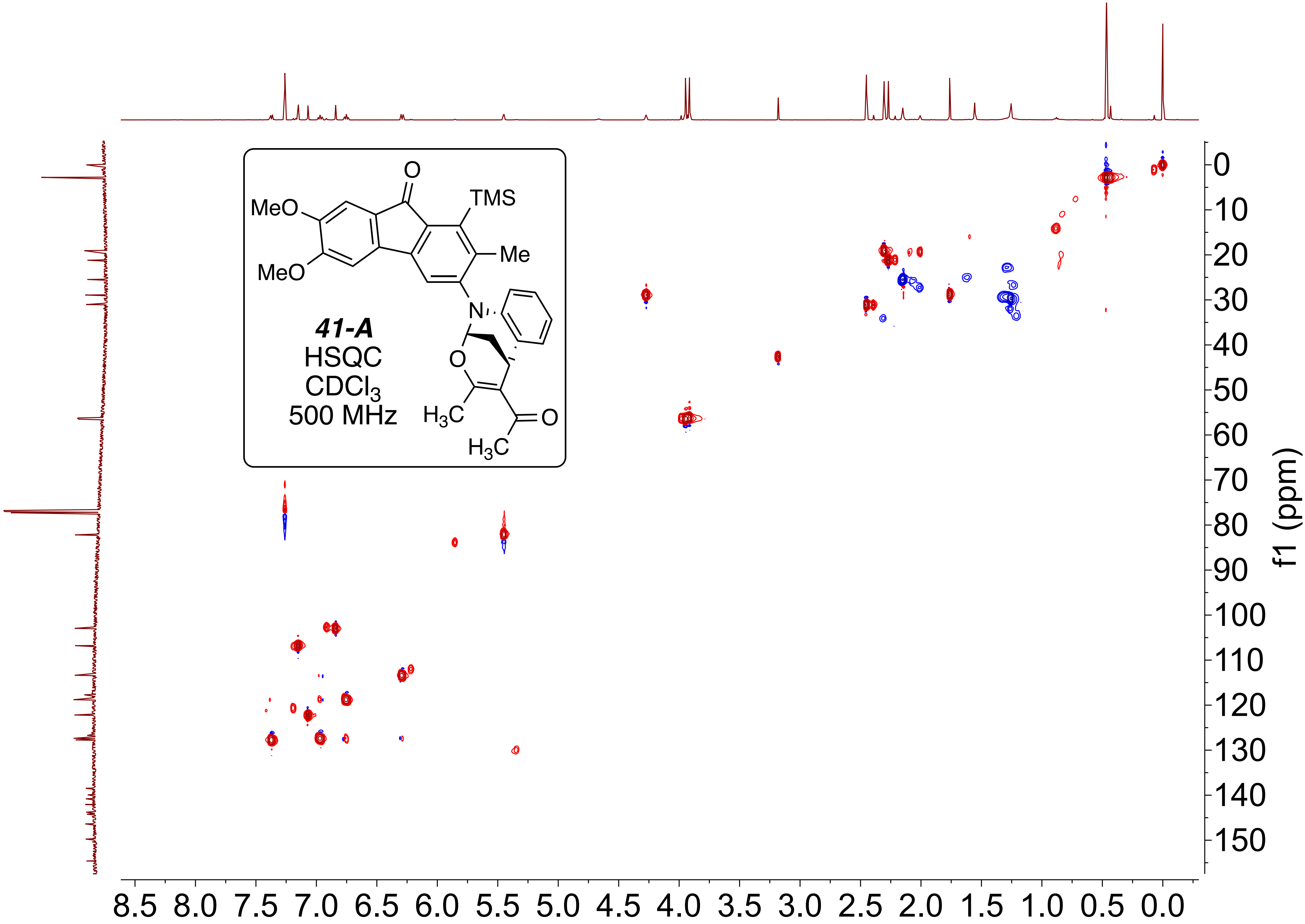

41-A
HSQC
$CDCl_3$
500 MHz
O
MeO
MeO
TMS
Me
N
O
H_3C
O
H_3C
8.5 8.0 7.5 7.0 6.5 6.0 5.5 5.0 4.5 4.0 3.5 3.0 2.5 2.0 1.5 1.0 0.5 0.0
0 10 20 30 40 50 60 70 80 90 100 110 120 130 140 150
f1 (ppm)

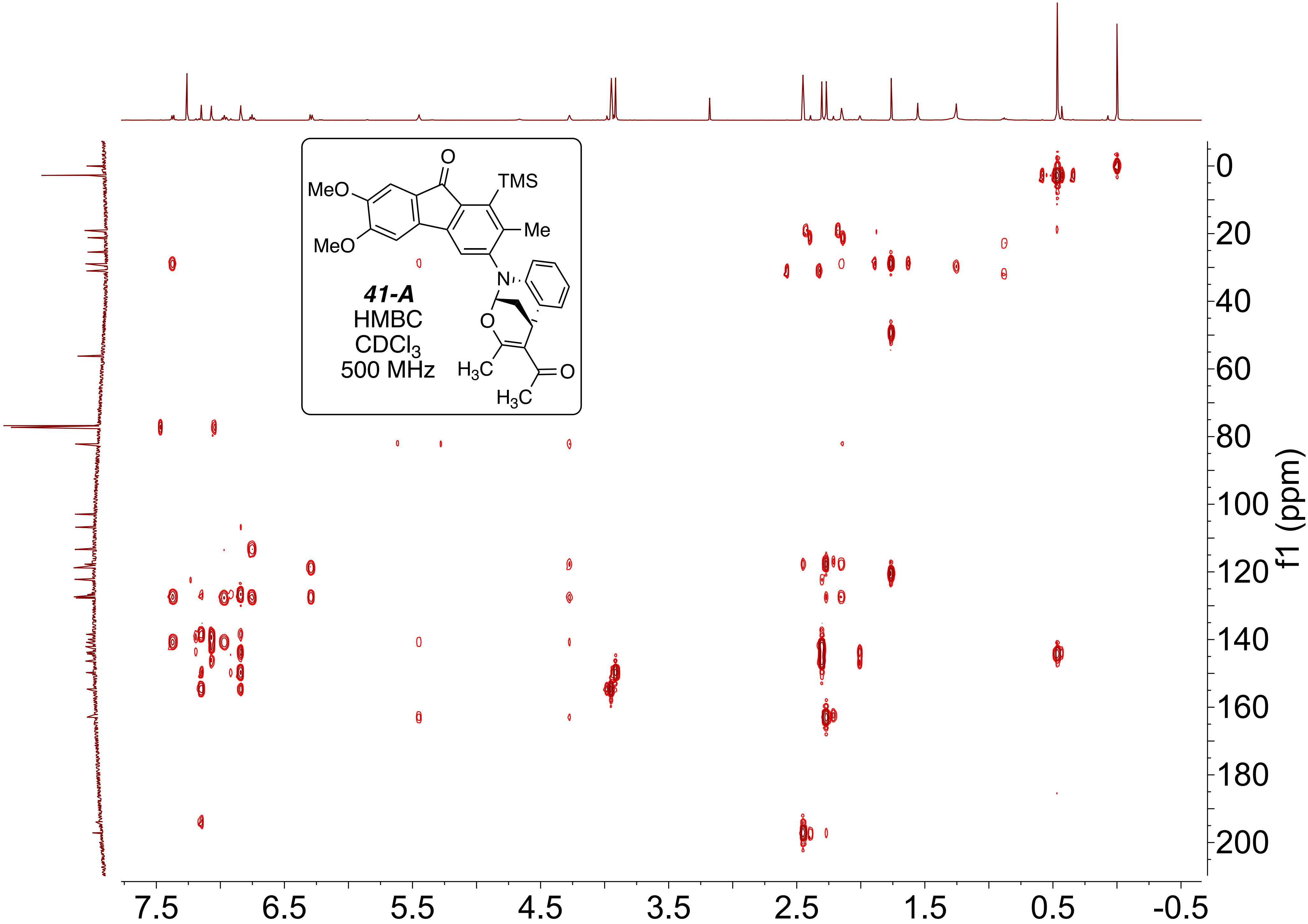
MeO
MeO
O
TMS
Me
N
O
H_3C
H_3C
O
41-A
HMBC
$CDCl_3$
500 MHz
f1 (ppm)
0
20
40
60
80
100
120
140
160
180
200
7.5
6.5
5.5
4.5
3.5
2.5
1.5
0.5
-0.5

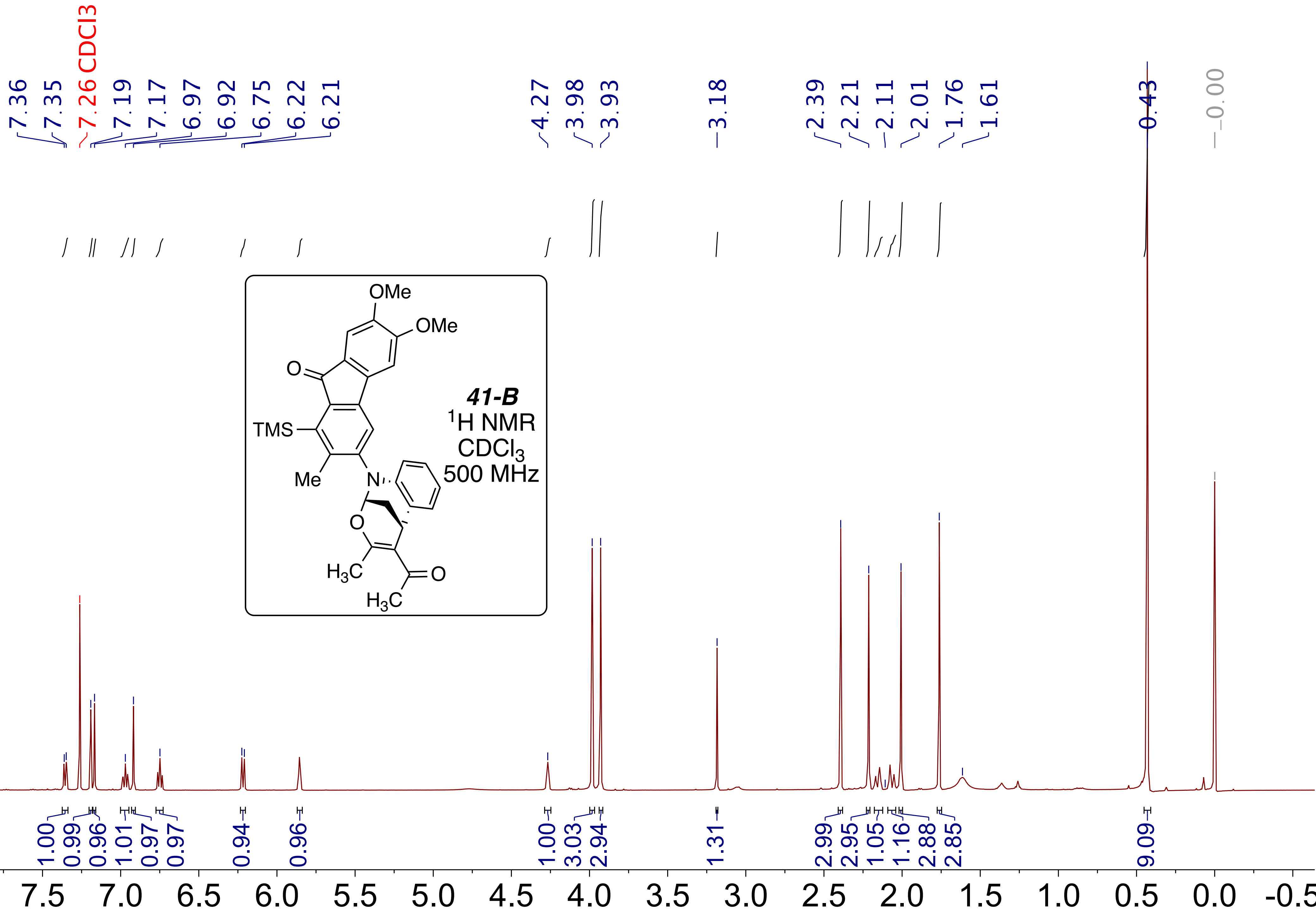
7.36
7.35
7.26 CDCl3
7.19
7.17
6.97
6.92
6.75
6.22
6.21
4.27
3.98
3.93
3.18
2.39
2.21
2.11
2.01
1.76
1.61
0.43
0.00
OMe
OMe
O
TMS
Me
N
O
H3C
O
H3C
41-B
1H NMR
CDCl3
500 MHz
1.00
0.99
0.96
1.01
0.97
0.97
0.94
0.96
1.00
3.03
2.94
1.31
2.99
2.95
1.05
1.16
2.88
2.85
9.09
7.5
7.0
6.5
6.0
5.5
5.0
4.5
4.0
3.5
3.0
2.5
2.0
1.5
1.0
0.5
0.0
-0.5

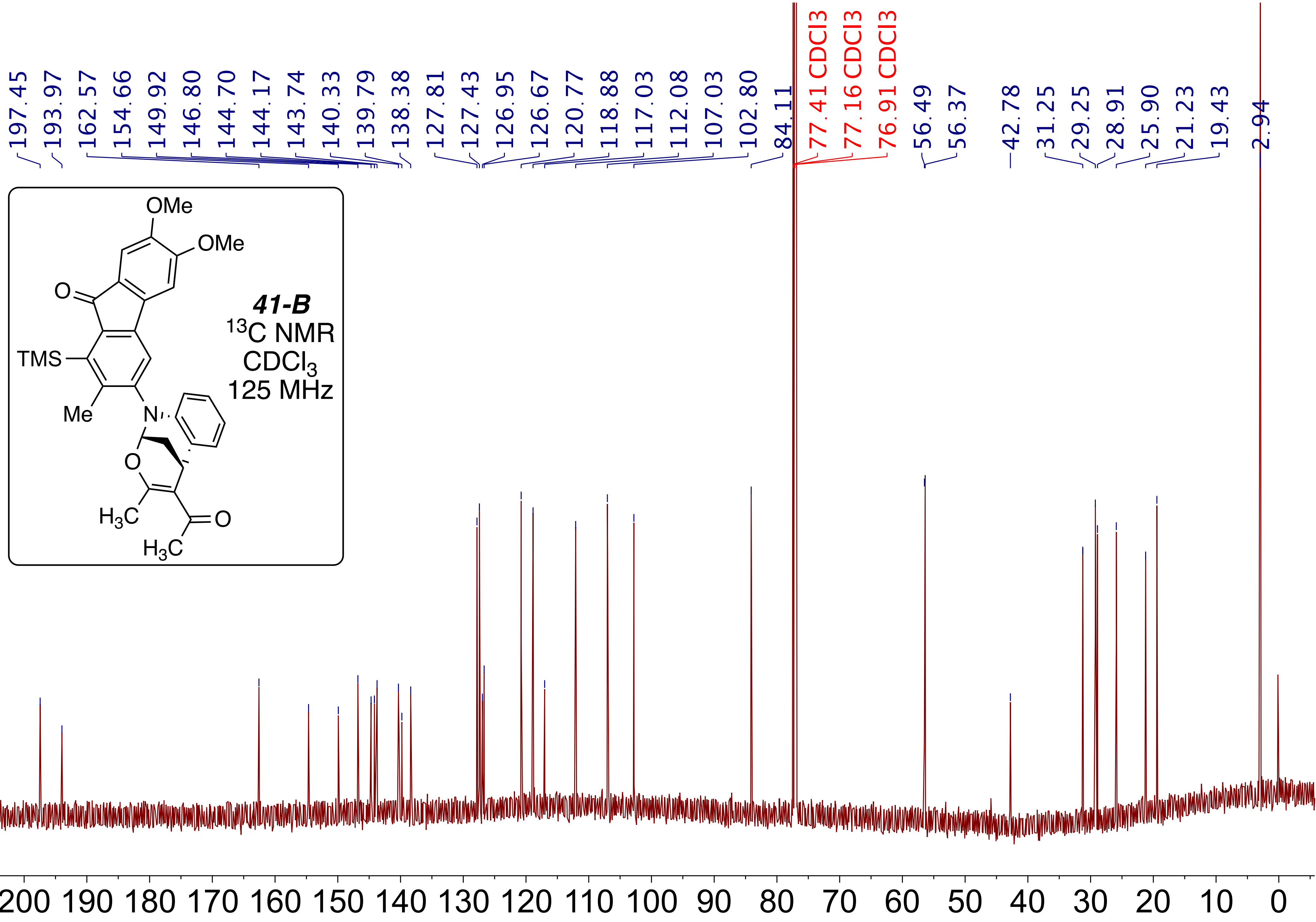
197.45
193.97
162.57
154.66
149.92
146.80
144.70
144.17
143.74
140.33
139.79
138.38
127.81
127.43
126.95
126.67
120.77
118.88
117.03
112.08
107.03
102.80
84.11
77.41 CDCl3
77.16 CDCl3
76.91 CDCl3
56.49
56.37
42.78
31.25
29.25
28.91
25.90
21.23
19.43
2.94
OMe
OMe
O
TMS
Me
N
O
H_3C
O
H_3C
41-B
^{13}C NMR
$CDCl_3$
125 MHz
200 190 180 170 160 150 140 130 120 110 100 90 80 70 60 50 40 30 20 10 0

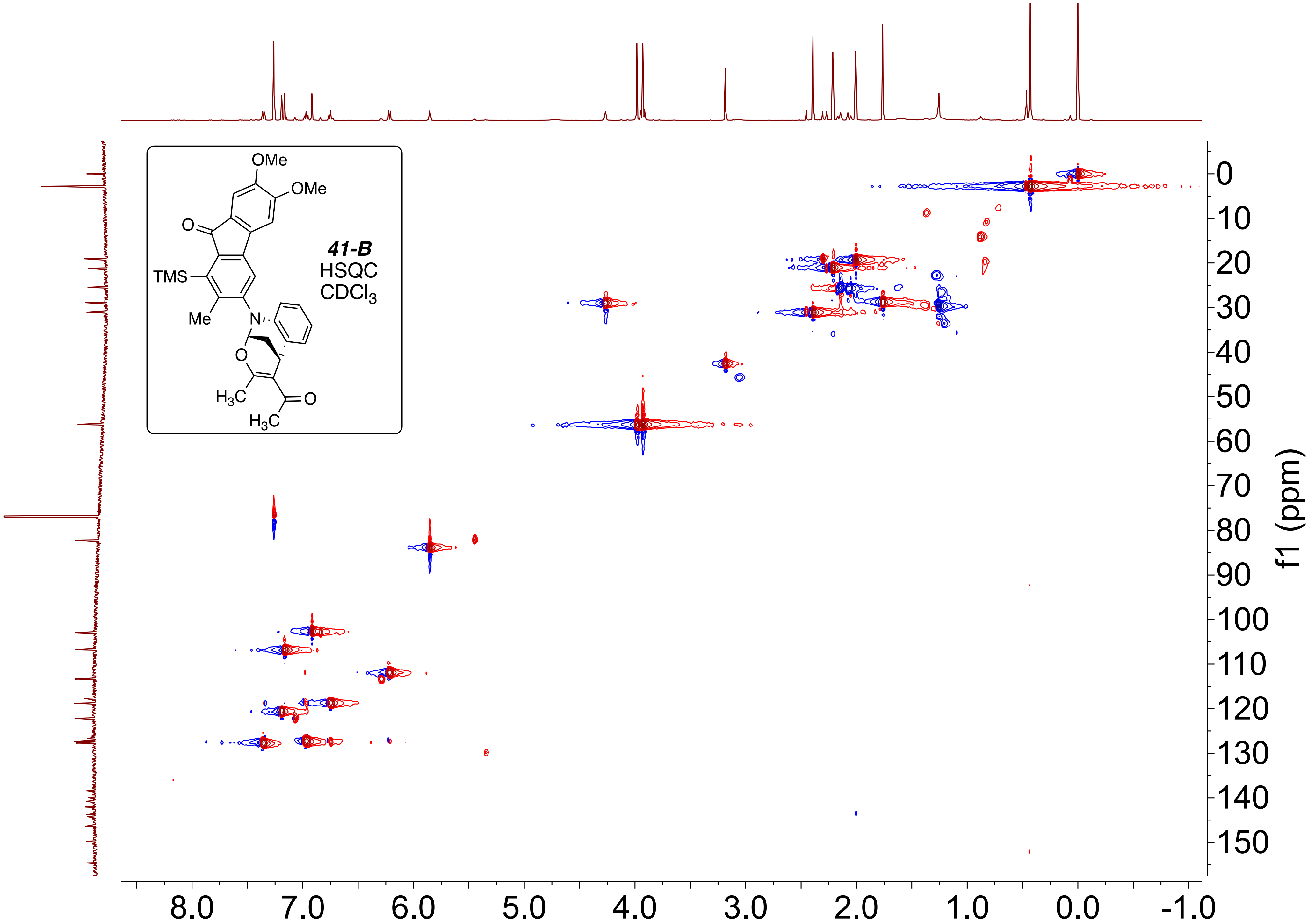
OMe
OMe
O
TMS
Me
N
O
H3C
O
H3C
41-B
HSQC
CDCl3
f1 (ppm)
0
10
20
30
40
50
60
70
80
90
100
110
120
130
140
150
8.0
7.0
6.0
5.0
4.0
3.0
2.0
1.0
0.0
-1.0

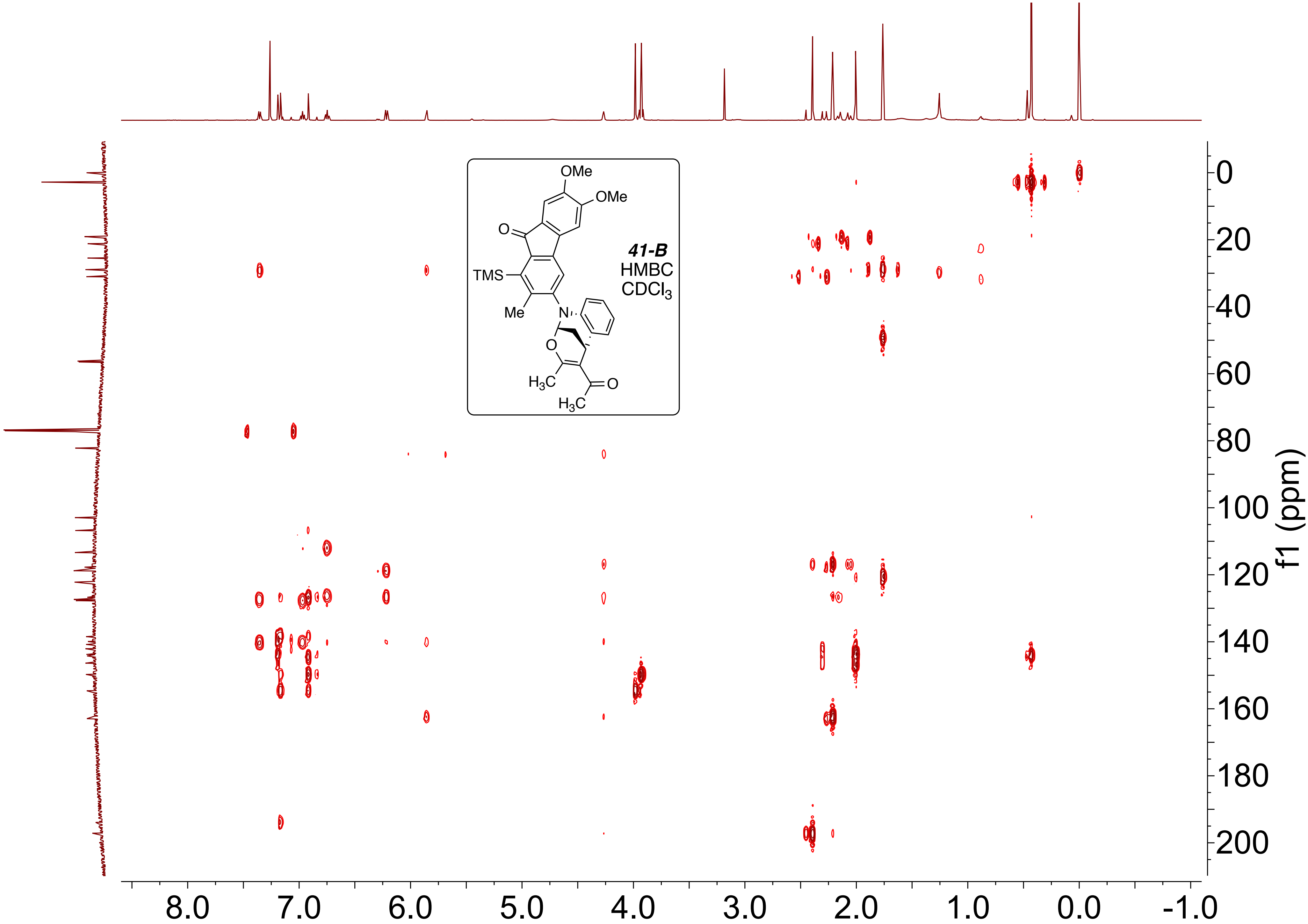
OMe
OMe
O
TMS
Me
N
O
H_3C
O
H_3C
41-B
HMBC
$CDCl_3$
f1 (ppm)
0
20
40
60
80
100
120
140
160
180
200
8.0
7.0
6.0
5.0
4.0
3.0
2.0
1.0
0.0
-1.0

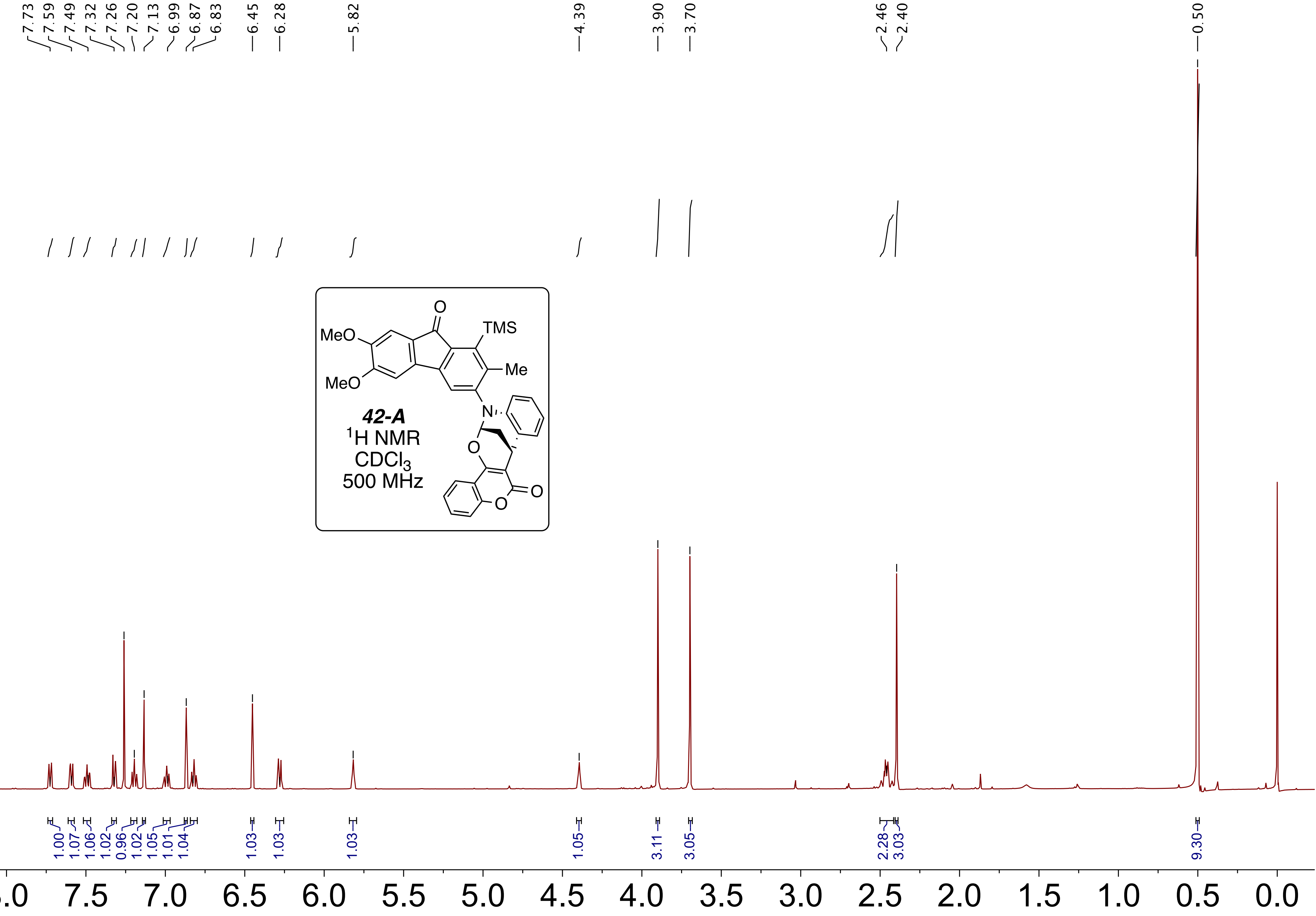
7.73
7.59
7.49
7.32
7.26
7.20
7.13
6.99
6.87
6.83
6.45
6.28
5.82
4.39
3.90
3.70
2.46
2.40
0.50
MeO
MeO
O
TMS
Me
N
O
O
O
42-A
1H NMR
CDCl3
500 MHz
1.00
1.07
1.06
1.02
0.96
1.02
1.05
1.01
1.04
1.03
1.03
1.03
1.05
3.11
3.05
2.28
3.03
9.30
.0
7.5
7.0
6.5
6.0
5.5
5.0
4.5
4.0
3.5
3.0
2.5
2.0
1.5
1.0
0.5
0.0

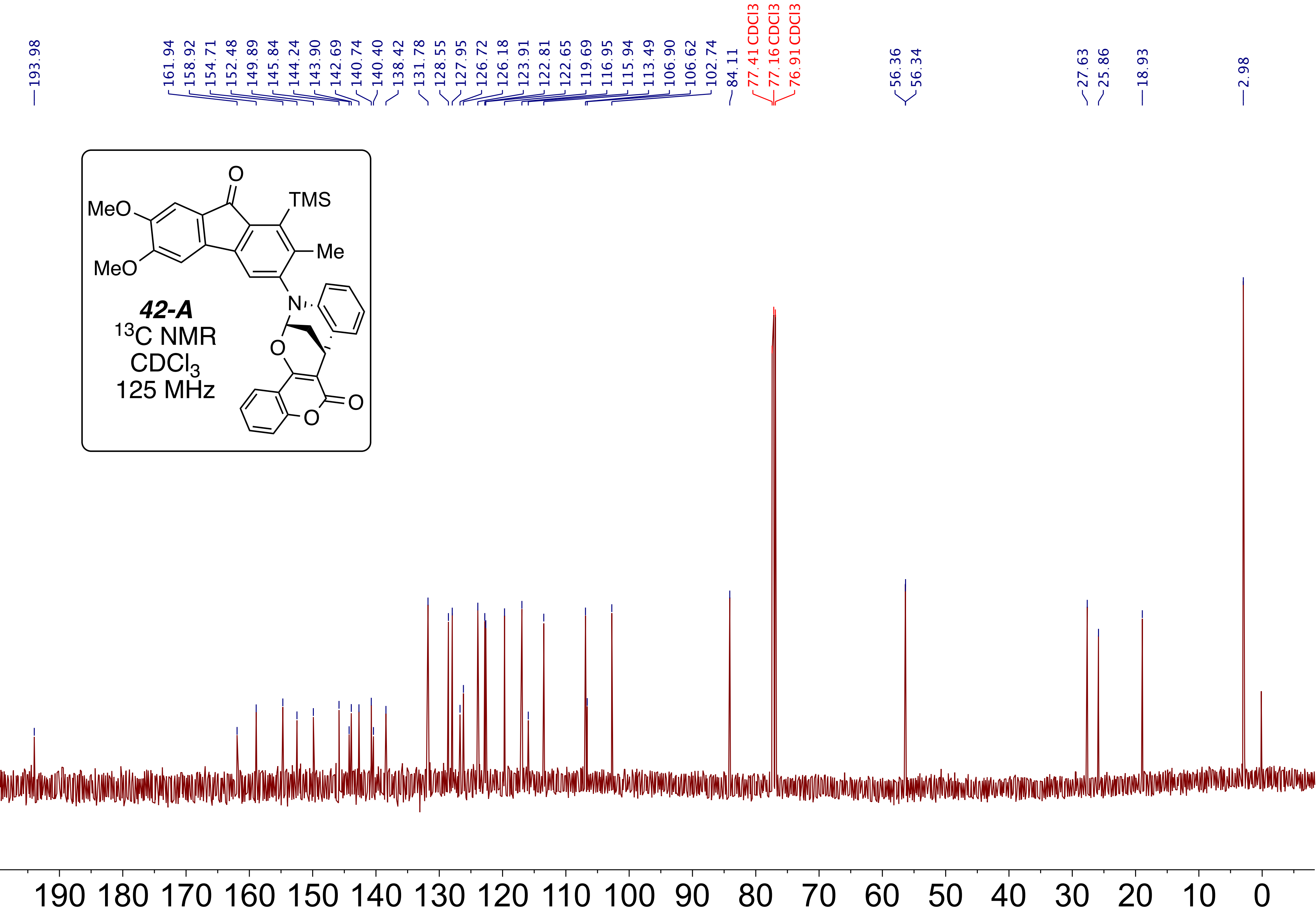
193.98
161.94
158.92
154.71
152.48
149.89
145.84
144.24
143.90
142.69
140.74
140.40
138.42
131.78
128.55
127.95
126.72
126.18
123.91
122.81
122.65
119.69
116.95
115.94
113.49
106.90
106.62
102.74
84.11
77.41 CDCl3
77.16 CDCl3
76.91 CDCl3
56.36
56.34
27.63
25.86
18.93
2.98
MeO
MeO
O
TMS
Me
N
O
O
O
42-A
13C NMR
CDCl3
125 MHz
190 180 170 160 150 140 130 120 110 100 90 80 70 60 50 40 30 20 10 0

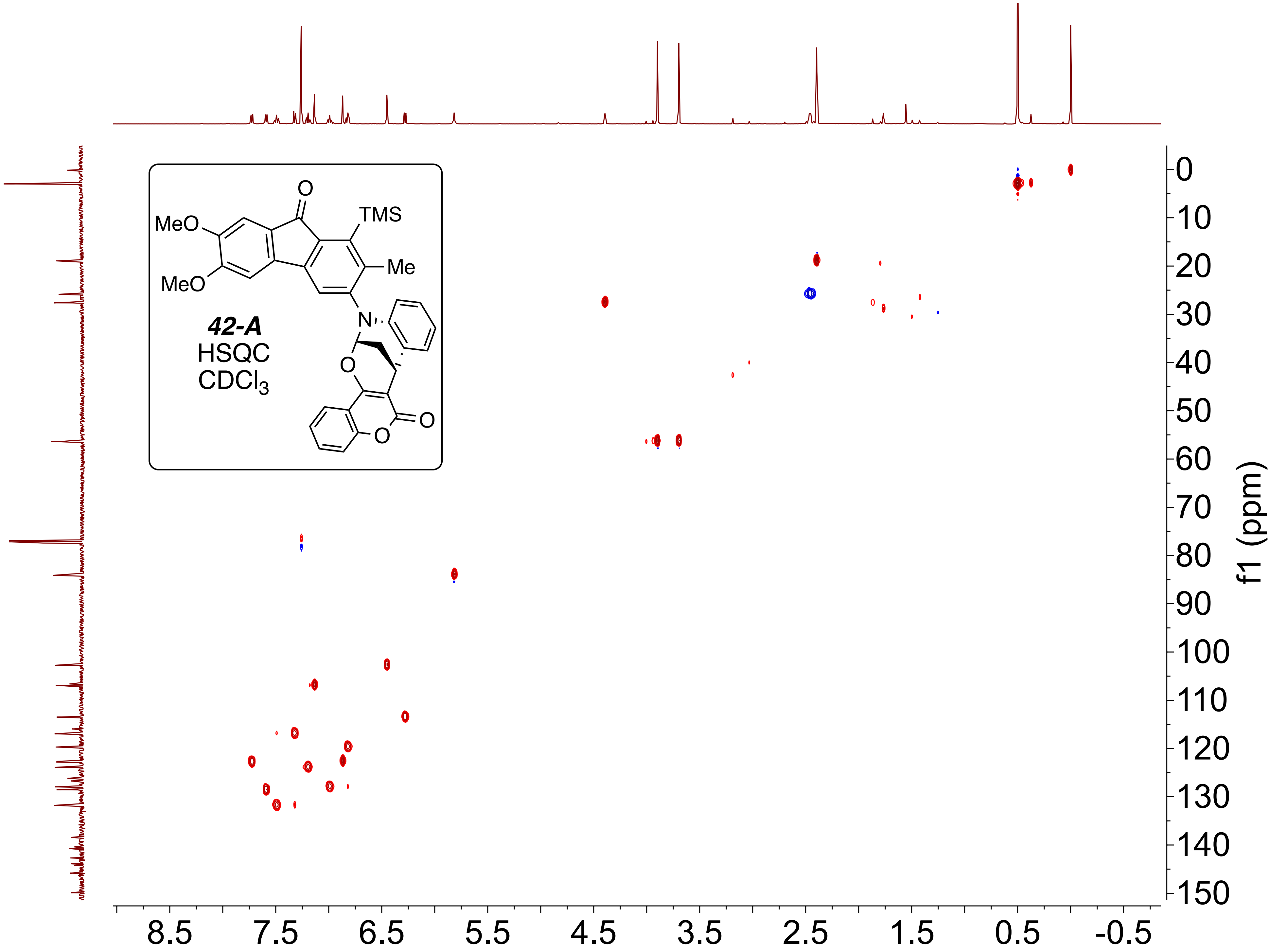

MeO
MeO
O
TMS
Me
N
O
O
O
42-A
HSQC
CDCl3
f1 (ppm)

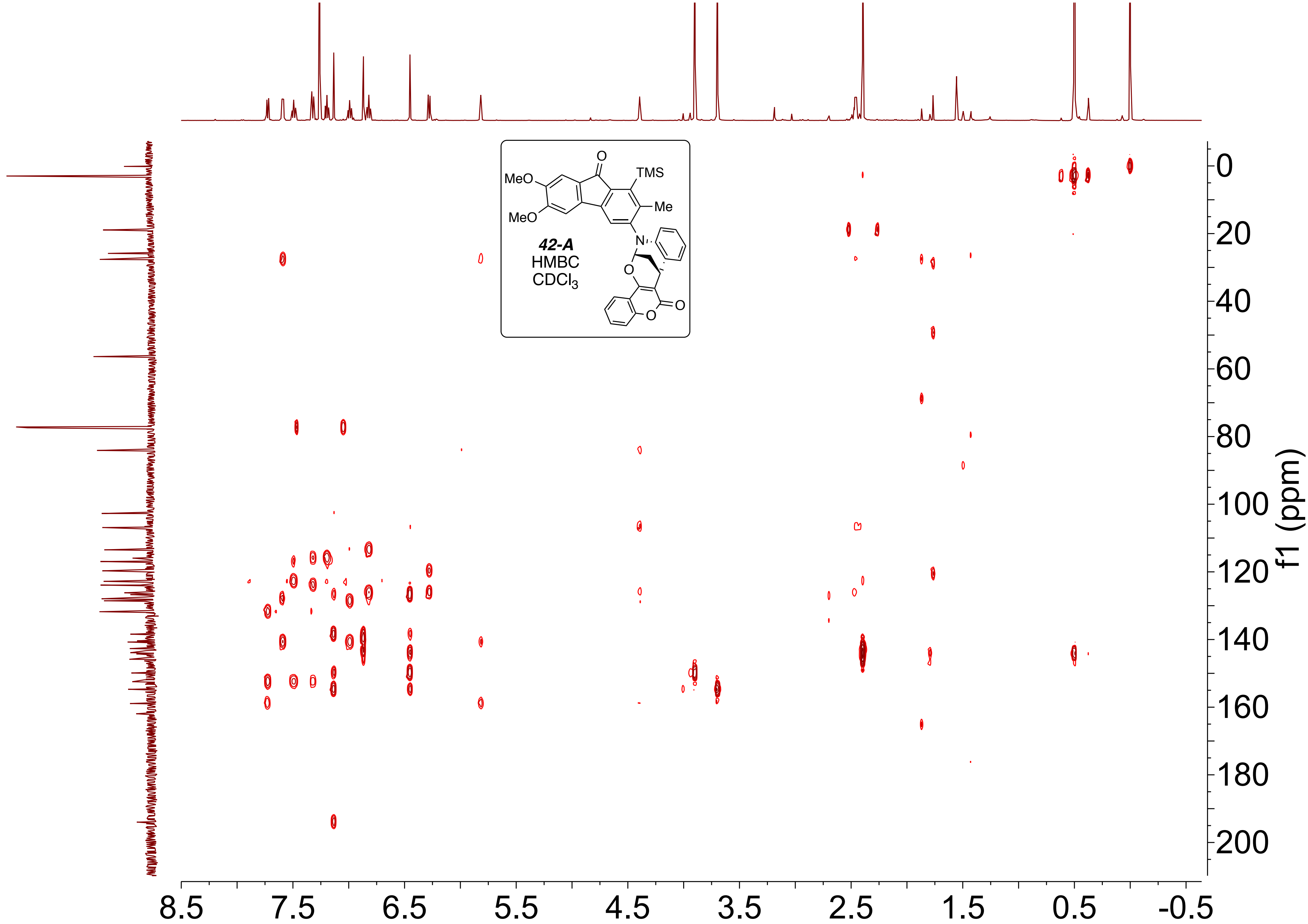
MeO
MeO
O
TMS
Me
N
O
O
O
42-A
HMBC
CDCl3
f1 (ppm)
0
20
40
60
80
100
120
140
160
180
200
8.5
7.5
6.5
5.5
4.5
3.5
2.5
1.5
0.5
-0.5

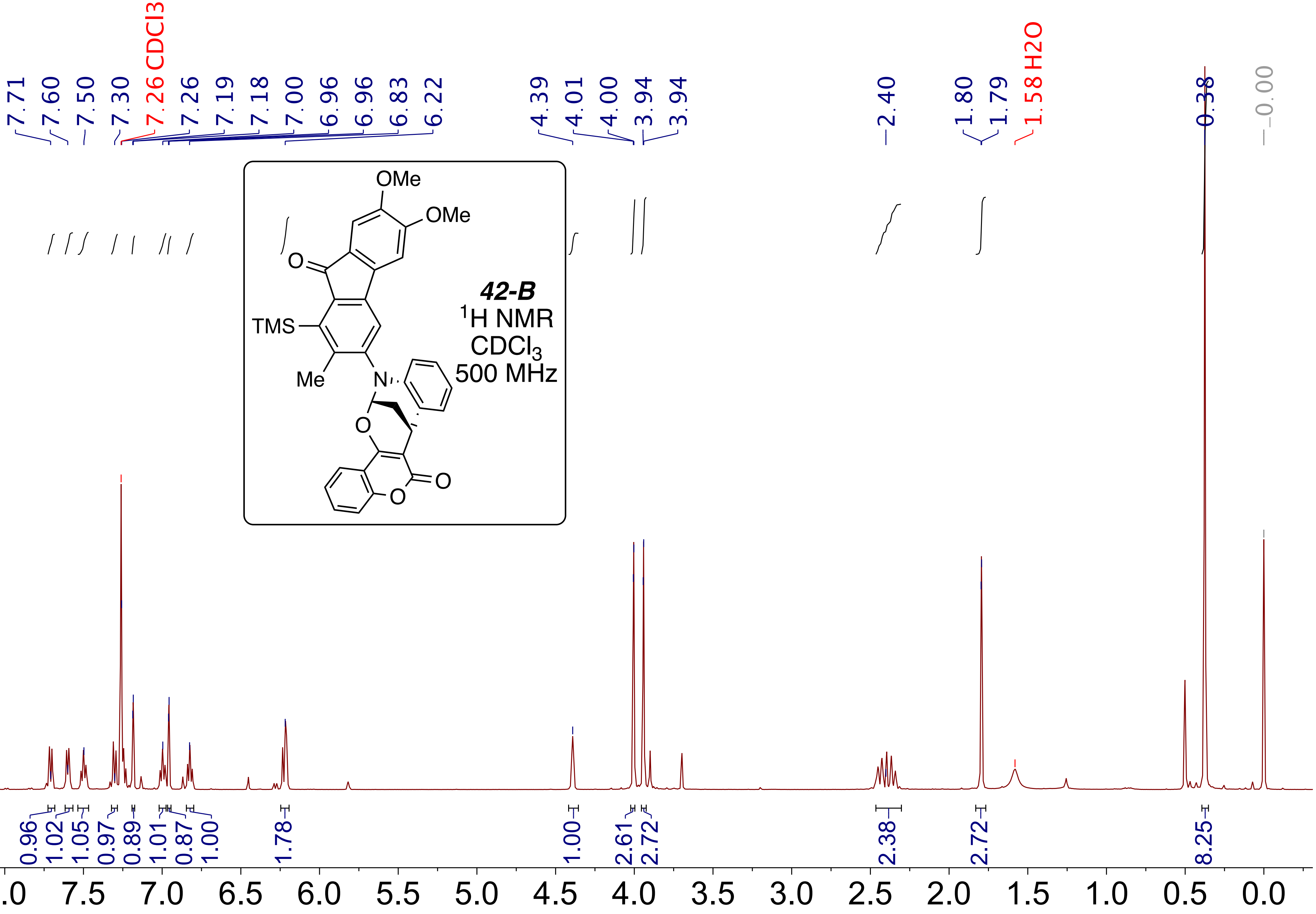
42-B
1H NMR
CDCl3
500 MHz
TMS
Me
OMe
OMe
7.71 7.60 7.50 7.30 7.26 CDCl3 7.26 7.19 7.18 7.00 6.96 6.96 6.83 6.22
4.39 4.01 4.00 3.94 3.94
2.40 1.80 1.79 1.58 H2O 0.38 0.00
0.96 1.02 1.05 0.97 0.89 1.01 0.87 1.00 1.78 1.00 2.61 2.72 2.38 2.72 8.25
7.5 7.0 6.5 6.0 5.5 5.0 4.5 4.0 3.5 3.0 2.5 2.0 1.5 1.0 0.5 0.0

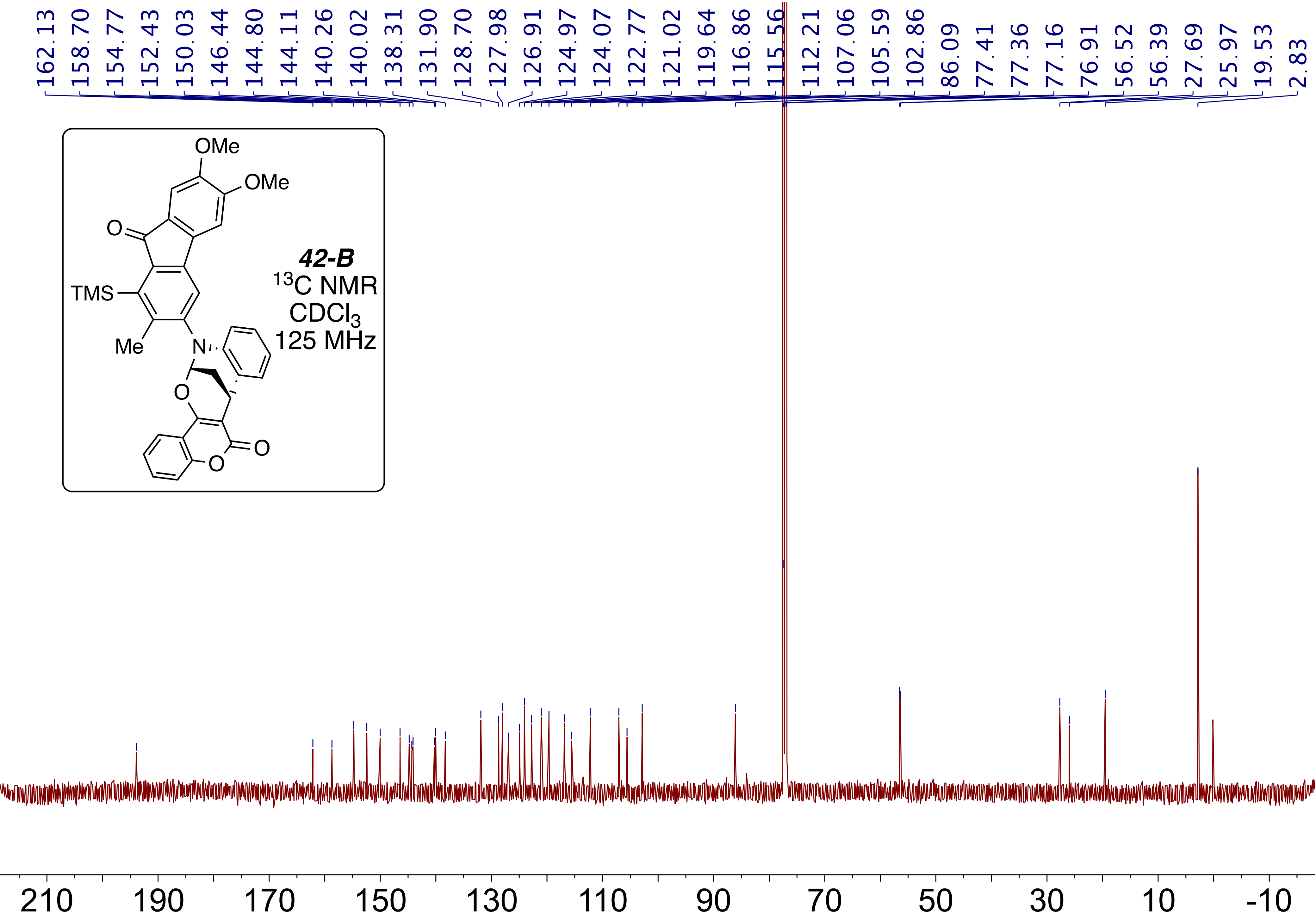
162.13
158.70
154.77
152.43
150.03
146.44
144.80
144.11
140.26
140.02
138.31
131.90
128.70
127.98
126.91
124.97
124.07
122.77
121.02
119.64
116.86
115.56
112.21
107.06
105.59
102.86
86.09
77.41
77.36
77.16
76.91
56.52
56.39
27.69
25.97
19.53
2.83
OMe
OMe
O
TMS
Me
N
O
O
O
42-B
13C NMR
CDCl3
125 MHz
210
190
170
150
130
110
90
70
50
30
10
-10

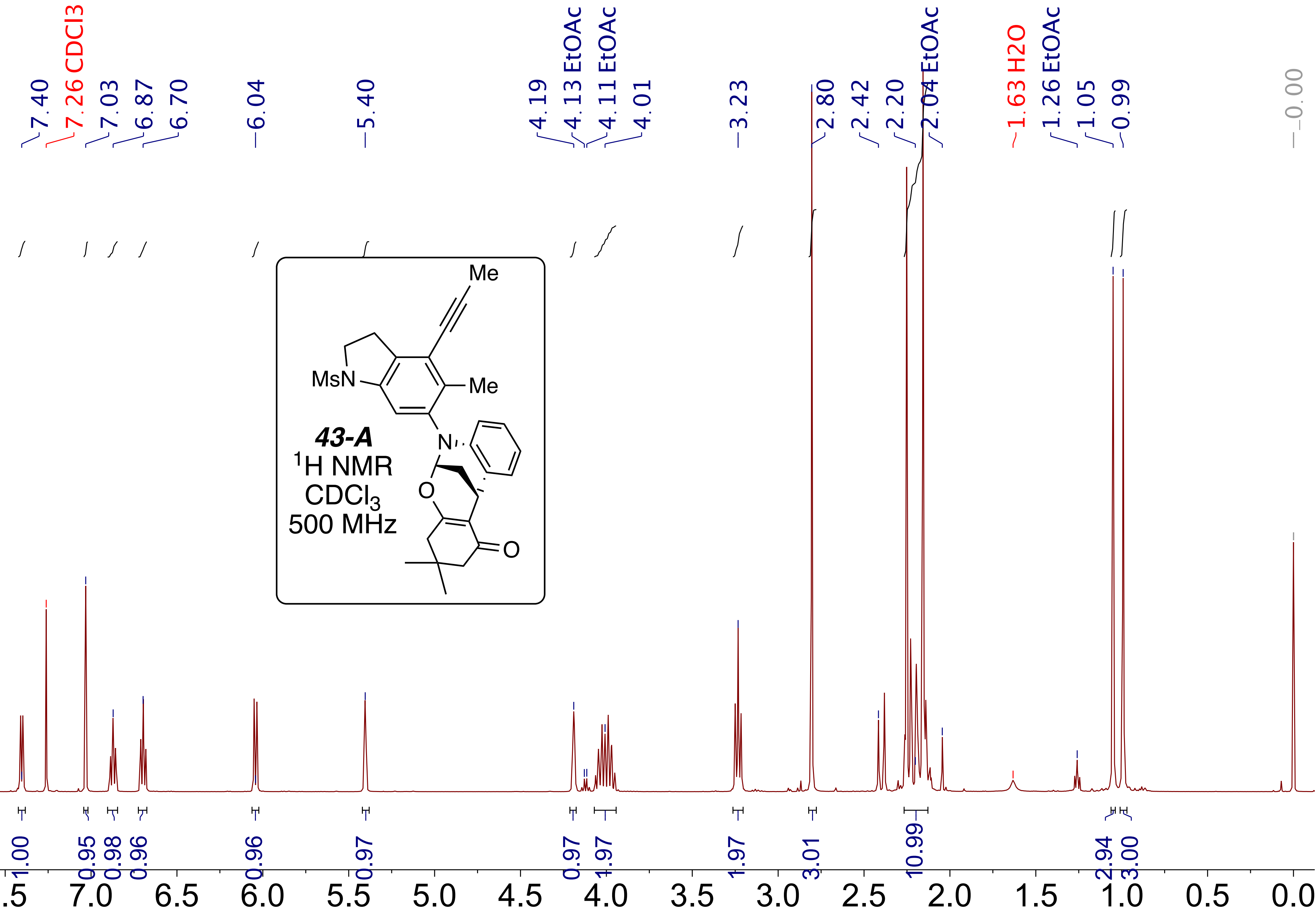
7.40
7.26 CDCl3
7.03
6.87
6.70
6.04
5.40
4.19
4.13 EtOAc
4.11 EtOAc
4.01
3.23
2.80
2.42
2.20
2.04 EtOAc
1.63 H2O
1.26 EtOAc
1.05
0.99
0.00
Me
MsN
Me
N
O
O
43-A
1H NMR
CDCl3
500 MHz
1.00
0.95
0.98
0.96
0.96
0.97
0.97
1.97
1.97
3.01
10.99
2.94
3.00
.5
7.0
6.5
6.0
5.5
5.0
4.5
4.0
3.5
3.0
2.5
2.0
1.5
1.0
0.5
0.0

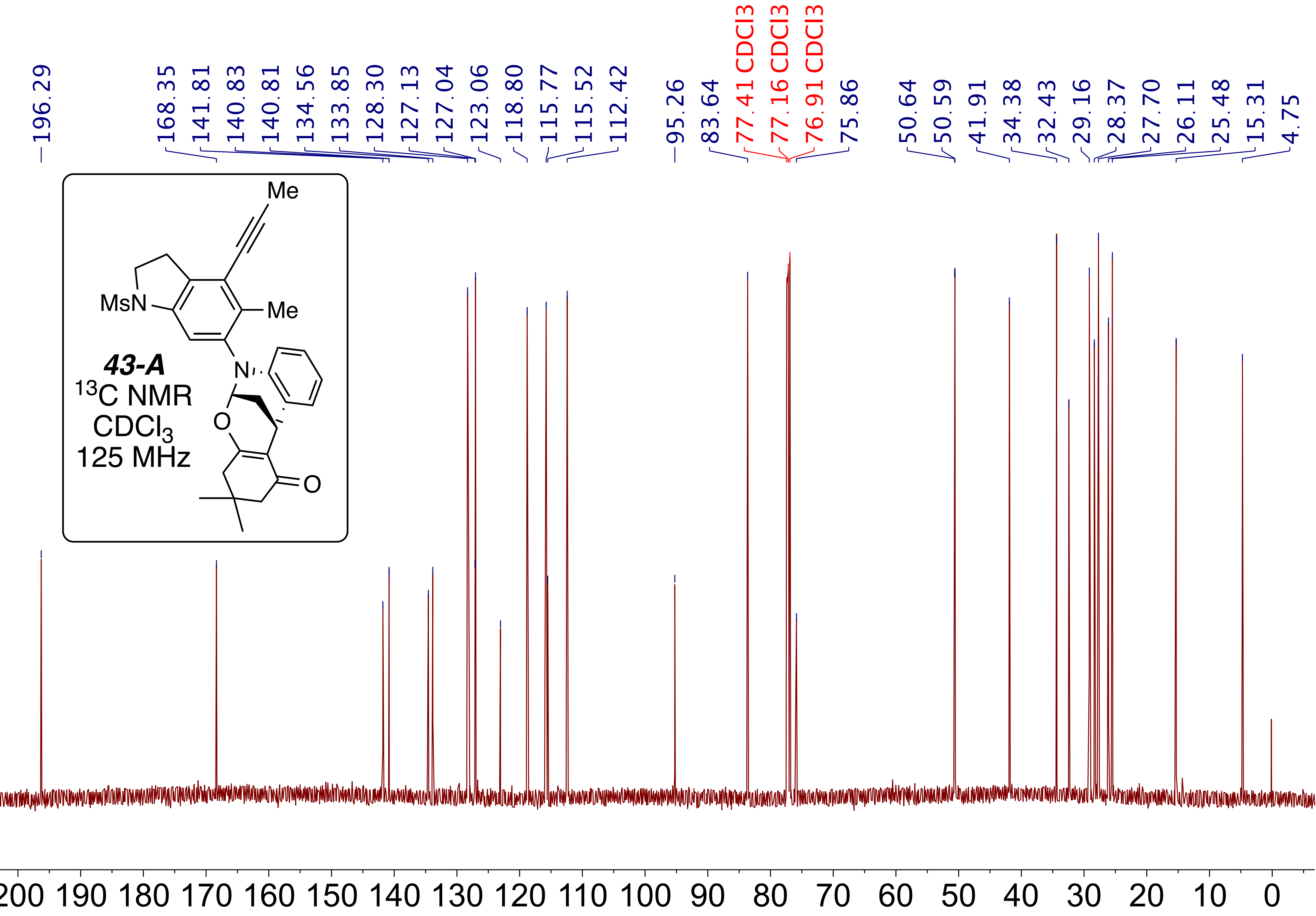

196.29
168.35
141.81
140.83
140.81
134.56
133.85
128.30
127.13
127.04
123.06
118.80
115.77
115.52
112.42
95.26
83.64
77.41 CDCl3
77.16 CDCl3
76.91 CDCl3
75.86
50.64
50.59
41.91
34.38
32.43
29.16
28.37
27.70
26.11
25.48
15.31
4.75
Me
MsN
Me
N
O
O
Me
43-A
13C NMR
CDCl3
125 MHz
200 190 180 170 160 150 140 130 120 110 100 90 80 70 60 50 40 30 20 10 0

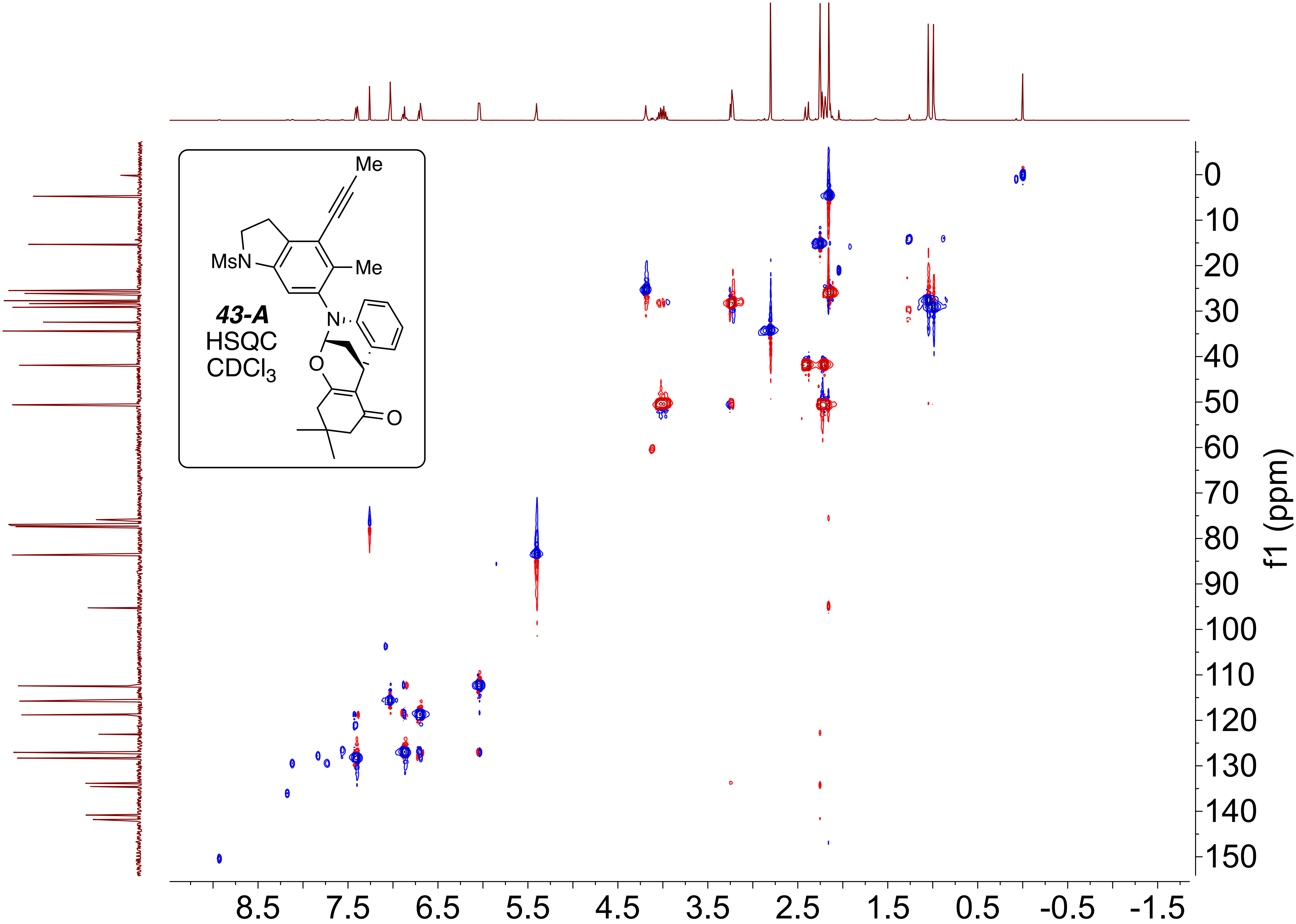

43-A
HSQC
CDCl3
MsN
Me
Me
N
O
O
f1 (ppm)

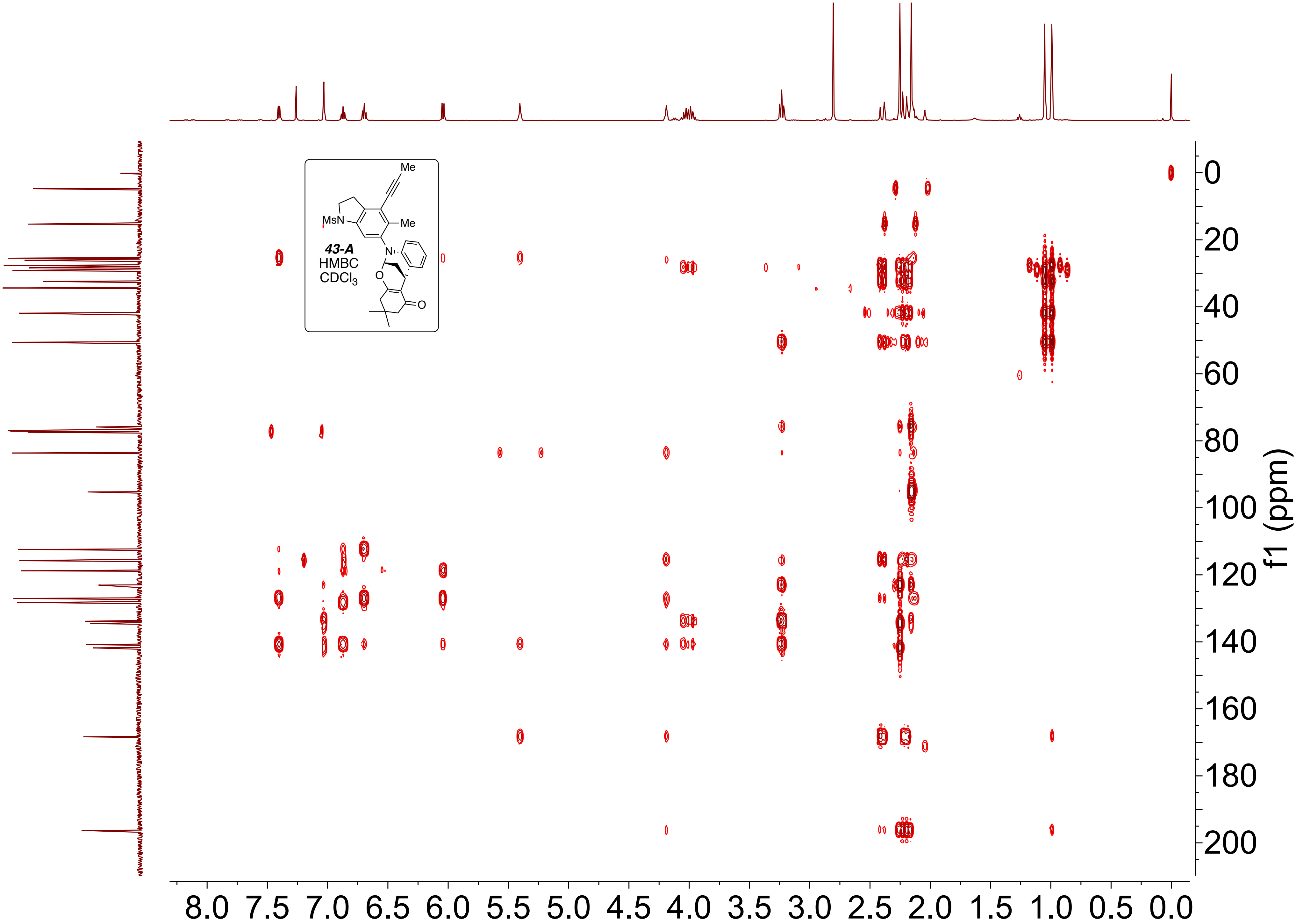
Me
MsN
Me
N
O
O
43-A
HMBC
CDCl3
f1 (ppm)
0 20 40 60 80 100 120 140 160 180 200
8.0 7.5 7.0 6.5 6.0 5.5 5.0 4.5 4.0 3.5 3.0 2.5 2.0 1.5 1.0 0.5 0.0

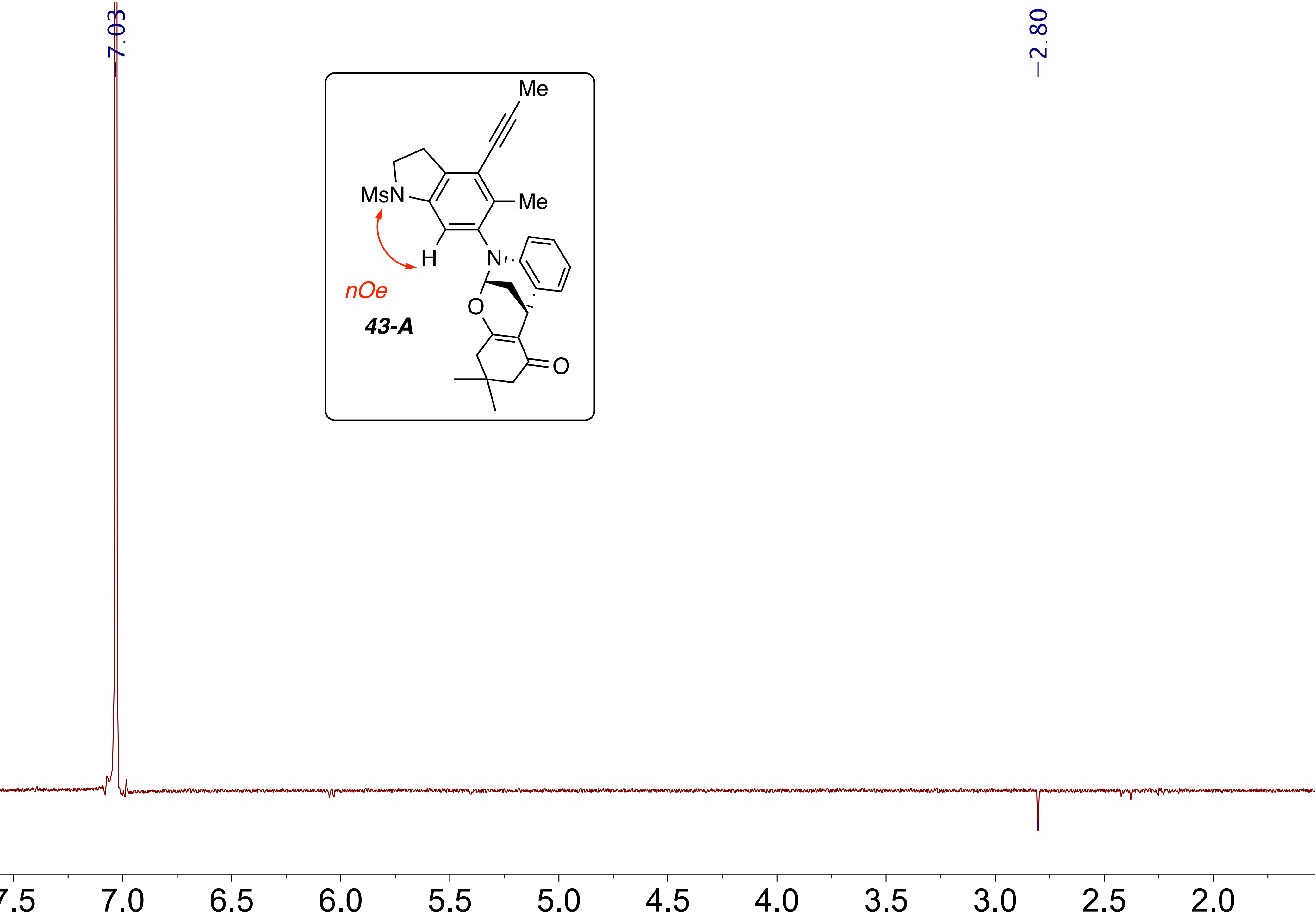
7.03
2.80
Me
MsN
Me
H
N
O
O
nOe
43-A
7.5
7.0
6.5
6.0
5.5
5.0
4.5
4.0
3.5
3.0
2.5
2.0

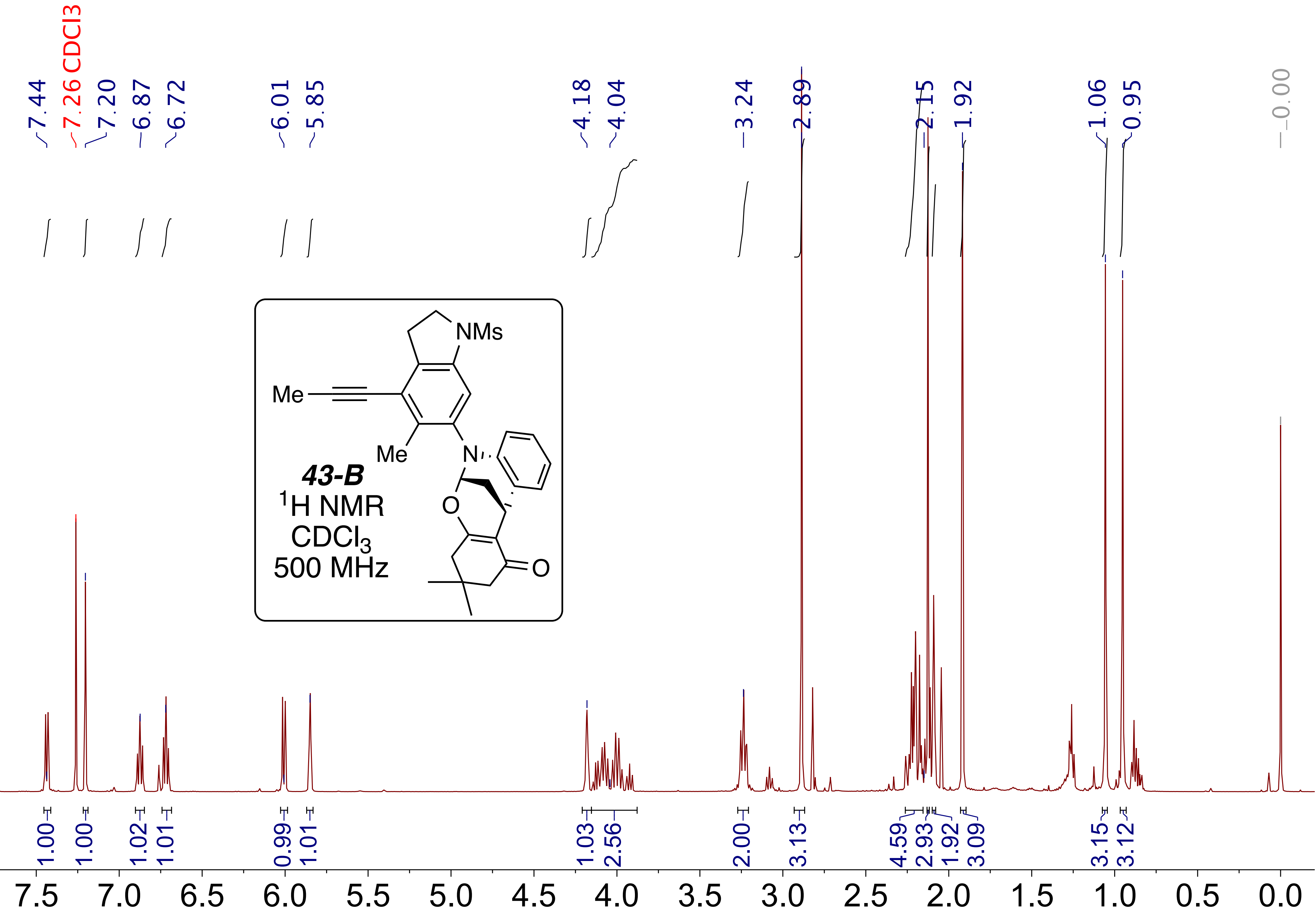
7.44
7.26 CDCl3
7.20
6.87
6.72
6.01
5.85
4.18
4.04
3.24
2.89
2.15
1.92
1.06
0.95
0.00
NMs
Me
Me
N
O
O
43-B
1H NMR
CDCl3
500 MHz
1.00
1.00
1.02
1.01
0.99
1.01
1.03
2.56
2.00
3.13
4.59
2.93
1.92
3.09
3.15
3.12
7.5
7.0
6.5
6.0
5.5
5.0
4.5
4.0
3.5
3.0
2.5
2.0
1.5
1.0
0.5
0.0

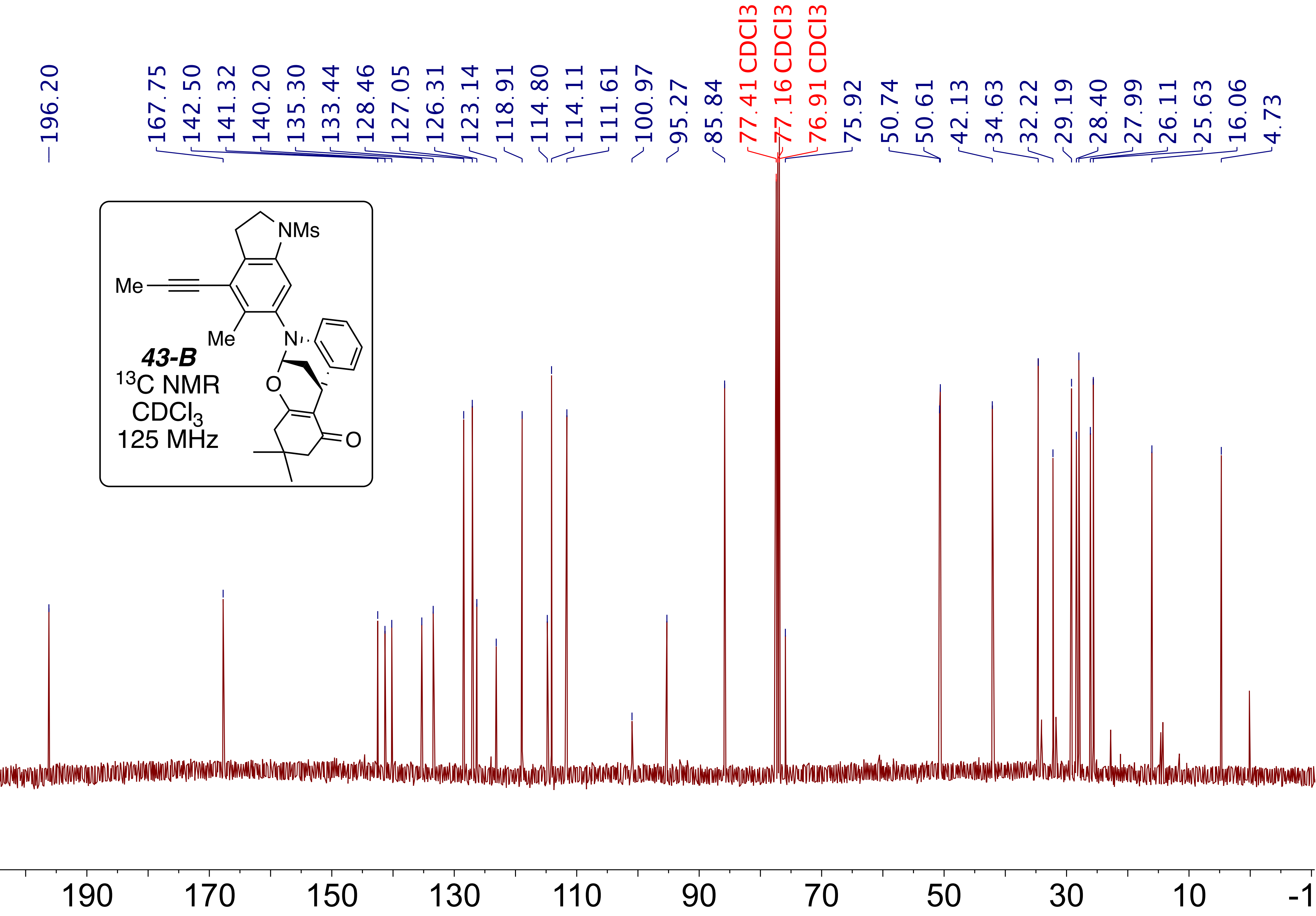
196.20
167.75
142.50
141.32
140.20
135.30
133.44
128.46
127.05
126.31
123.14
118.91
114.80
114.11
111.61
100.97
95.27
85.84
77.41 CDCl3
77.16 CDCl3
76.91 CDCl3
75.92
50.74
50.61
42.13
34.63
32.22
29.19
28.40
27.99
26.11
25.63
16.06
4.73
NMs
Me
Me
N
O
O
43-B
13C NMR
CDCl3
125 MHz
190
170
150
130
110
90
70
50
30
10
-1

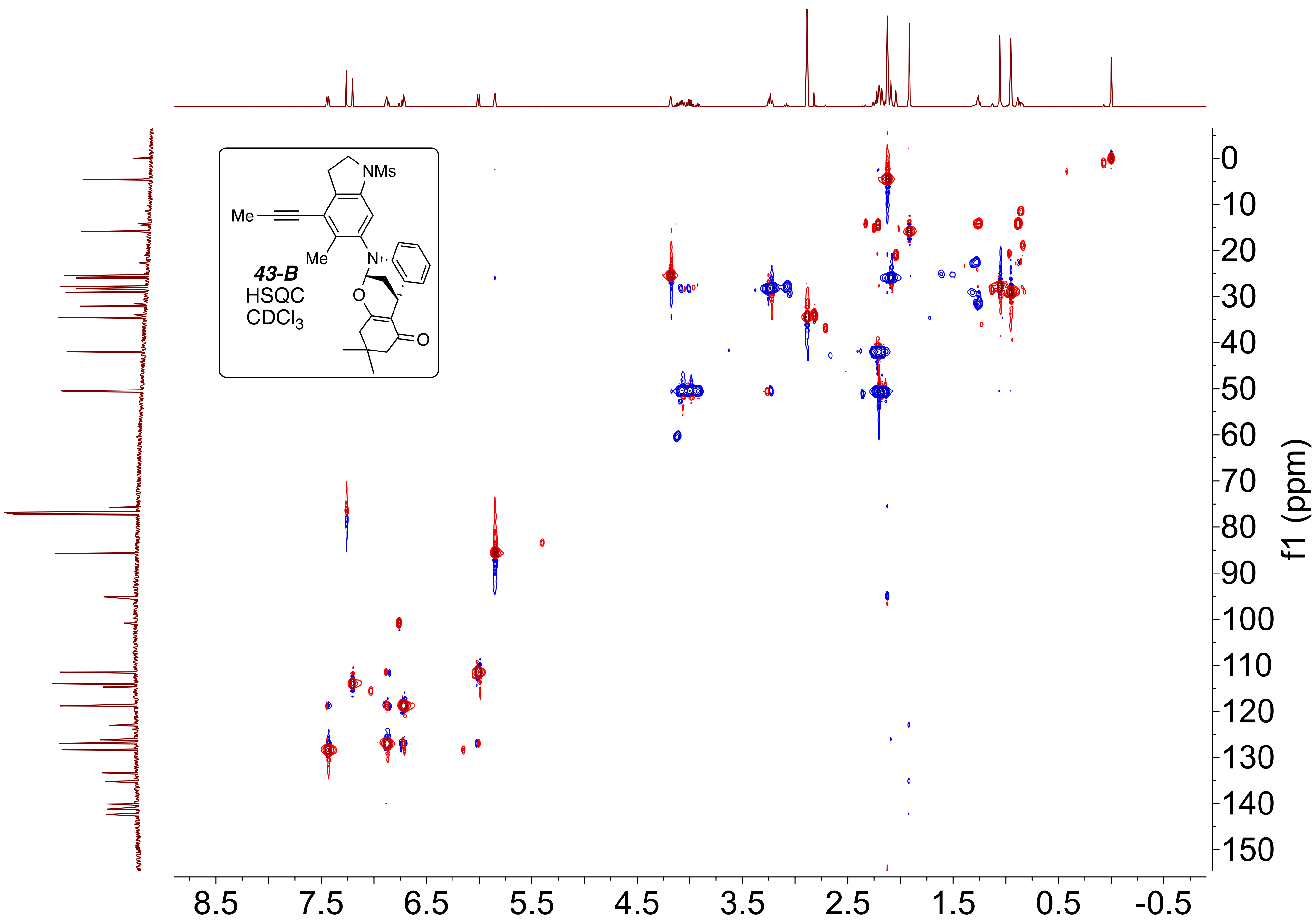
NMs
Me
Me
N
O
O
43-B
HSQC
CDCl3
f1 (ppm)
8.5
7.5
6.5
5.5
4.5
3.5
2.5
1.5
0.5
-0.5
0
10
20
30
40
50
60
70
80
90
100
110
120
130
140
150

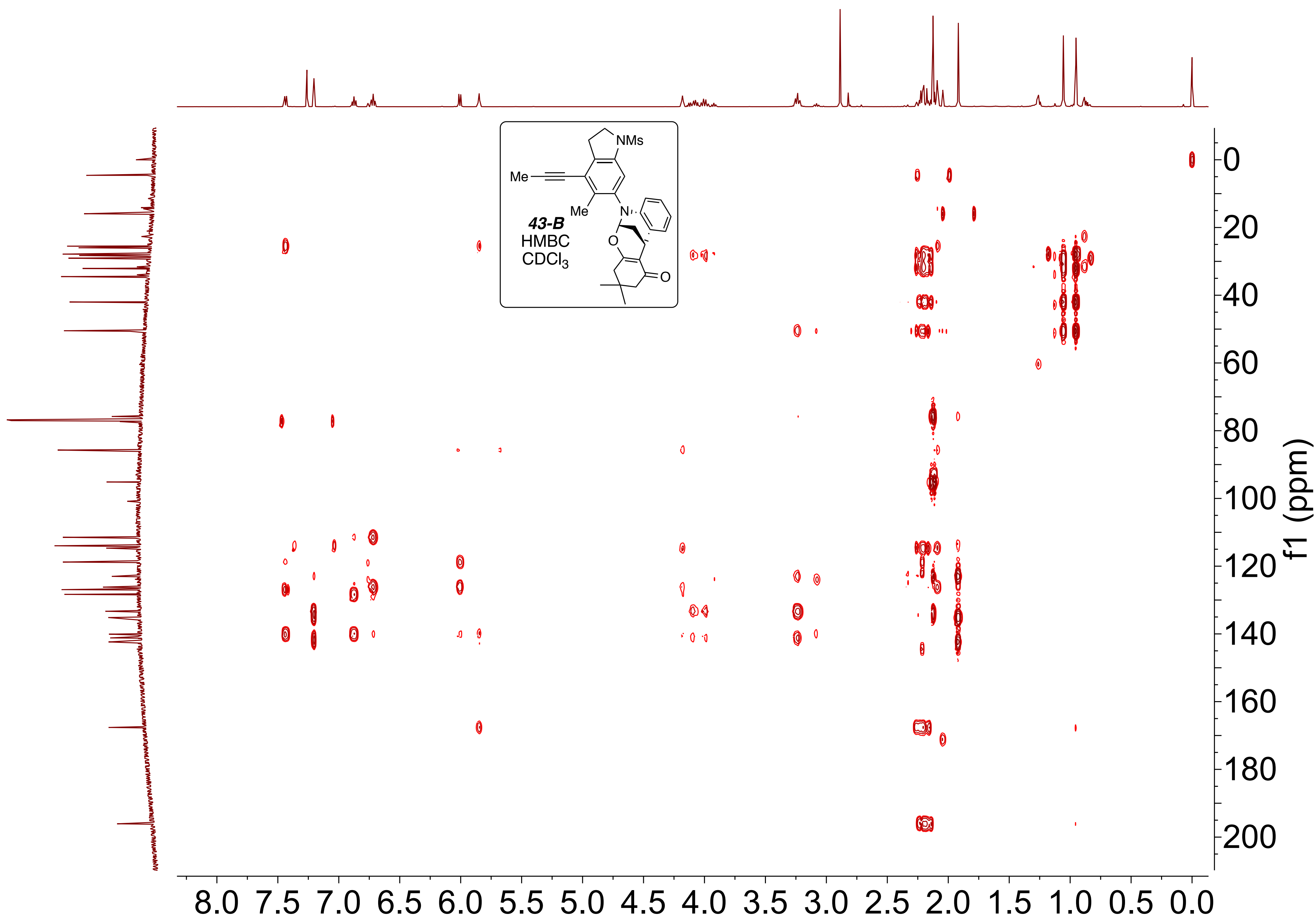
NMs
Me
Me
N
O
O
43-B
HMBC
CDCl3
f1 (ppm)
0
20
40
60
80
100
120
140
160
180
200
8.0 7.5 7.0 6.5 6.0 5.5 5.0 4.5 4.0 3.5 3.0 2.5 2.0 1.5 1.0 0.5 0.0

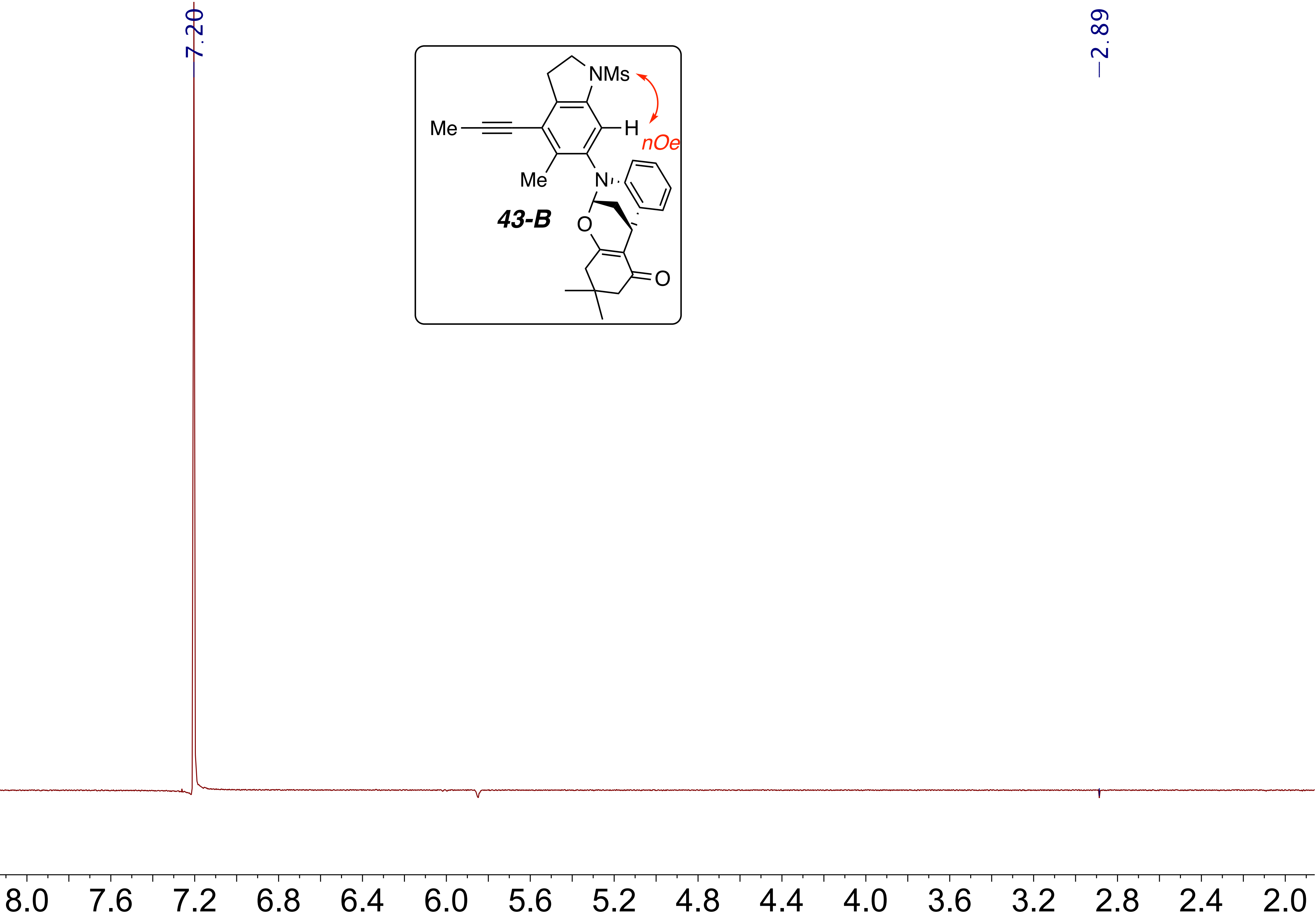

7.20
2.89
Me
NMs
H
nOe
Me
N
O
O
43-B
8.0
7.6
7.2
6.8
6.4
6.0
5.6
5.2
4.8
4.4
4.0
3.6
3.2
2.8
2.4
2.0

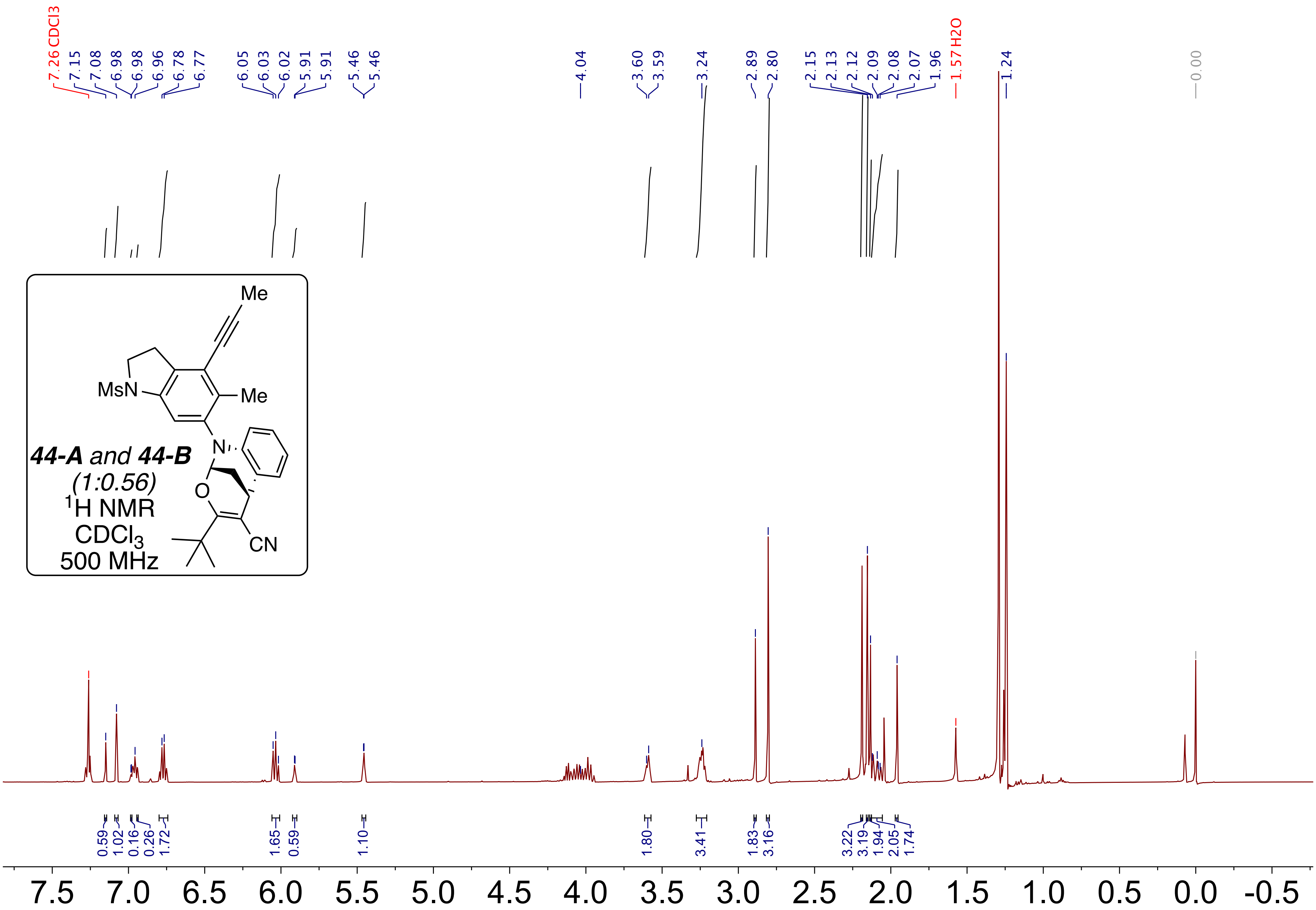
7.26 CDCl3
7.15
7.08
6.98
6.98
6.96
6.78
6.77
6.05
6.03
6.02
5.91
5.91
5.46
5.46
4.04
3.60
3.59
3.24
2.89
2.80
2.15
2.13
2.12
2.09
2.08
2.07
1.96
1.57 H2O
1.24
0.00
Me
MsN
Me
N
O
CN
44-A and 44-B
(1:0.56)
1H NMR
CDCl3
500 MHz
0.59
1.02
0.16
0.26
1.72
1.65
0.59
1.10
1.80
3.41
1.83
3.16
3.22
3.19
1.94
2.05
1.74
7.5
7.0
6.5
6.0
5.5
5.0
4.5
4.0
3.5
3.0
2.5
2.0
1.5
1.0
0.5
0.0
-0.5

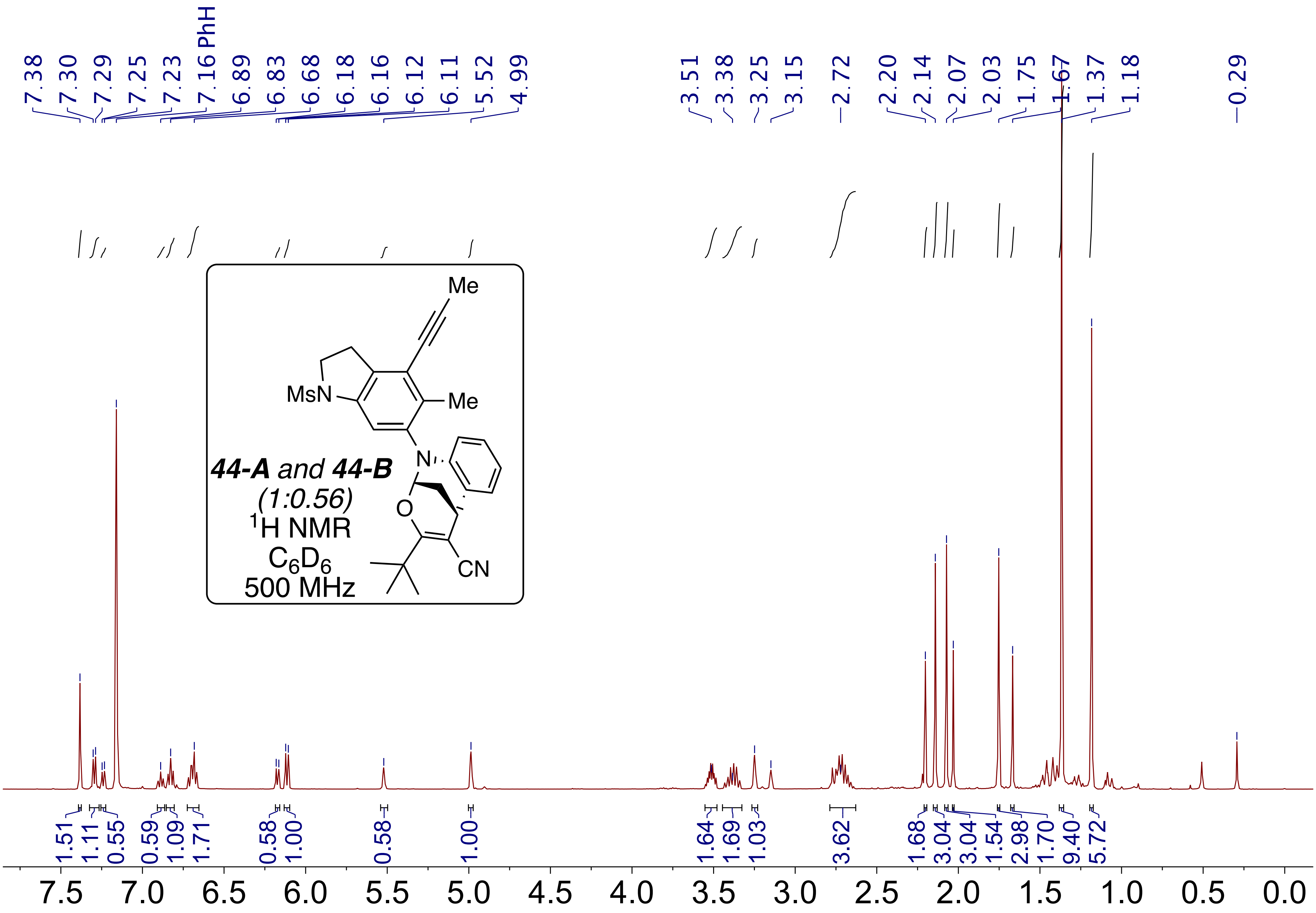

7.38
7.30
7.29
7.25
7.23
7.16 PhH
6.89
6.83
6.68
6.18
6.16
6.12
6.11
5.52
4.99
3.51
3.38
3.25
3.15
2.72
2.20
2.14
2.07
2.03
1.75
1.67
1.37
1.18
0.29
Me
MsN
Me
N
O
CN
44-A and 44-B
(1:0.56)
^{1}H NMR
C_6D_6
500 MHz
1.51
1.11
0.55
0.59
1.09
1.71
0.58
1.00
0.58
1.00
1.64
1.69
1.03
3.62
1.68
3.04
3.04
1.54
2.98
1.70
9.40
5.72
7.5
7.0
6.5
6.0
5.5
5.0
4.5
4.0
3.5
3.0
2.5
2.0
1.5
1.0
0.5
0.0

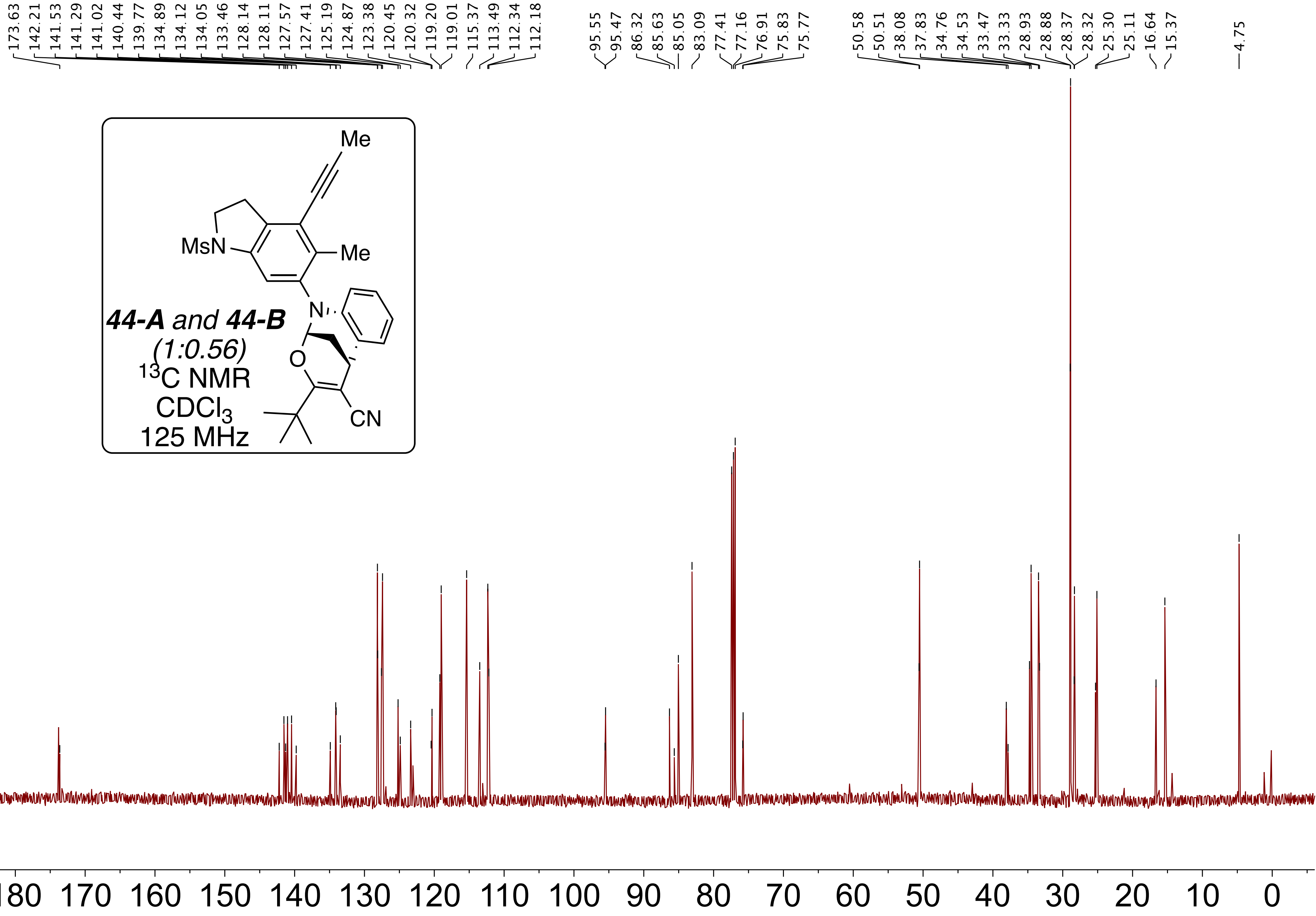
173.63
142.21
141.53
141.29
141.02
140.44
139.77
134.89
134.12
134.05
133.46
128.14
128.11
127.57
127.41
125.19
124.87
123.38
120.45
120.32
119.20
119.01
115.37
113.49
112.34
112.18
95.55
95.47
86.32
85.63
85.05
83.09
77.41
77.16
76.91
75.83
75.77
50.58
50.51
38.08
37.83
34.76
34.53
33.47
33.33
28.93
28.88
28.37
28.32
25.30
25.11
16.64
15.37
4.75
Me
MsN
Me
N
O
CN
44-A and 44-B
(1:0.56)
13C NMR
CDCl3
125 MHz
180 170 160 150 140 130 120 110 100 90 80 70 60 50 40 30 20 10 0

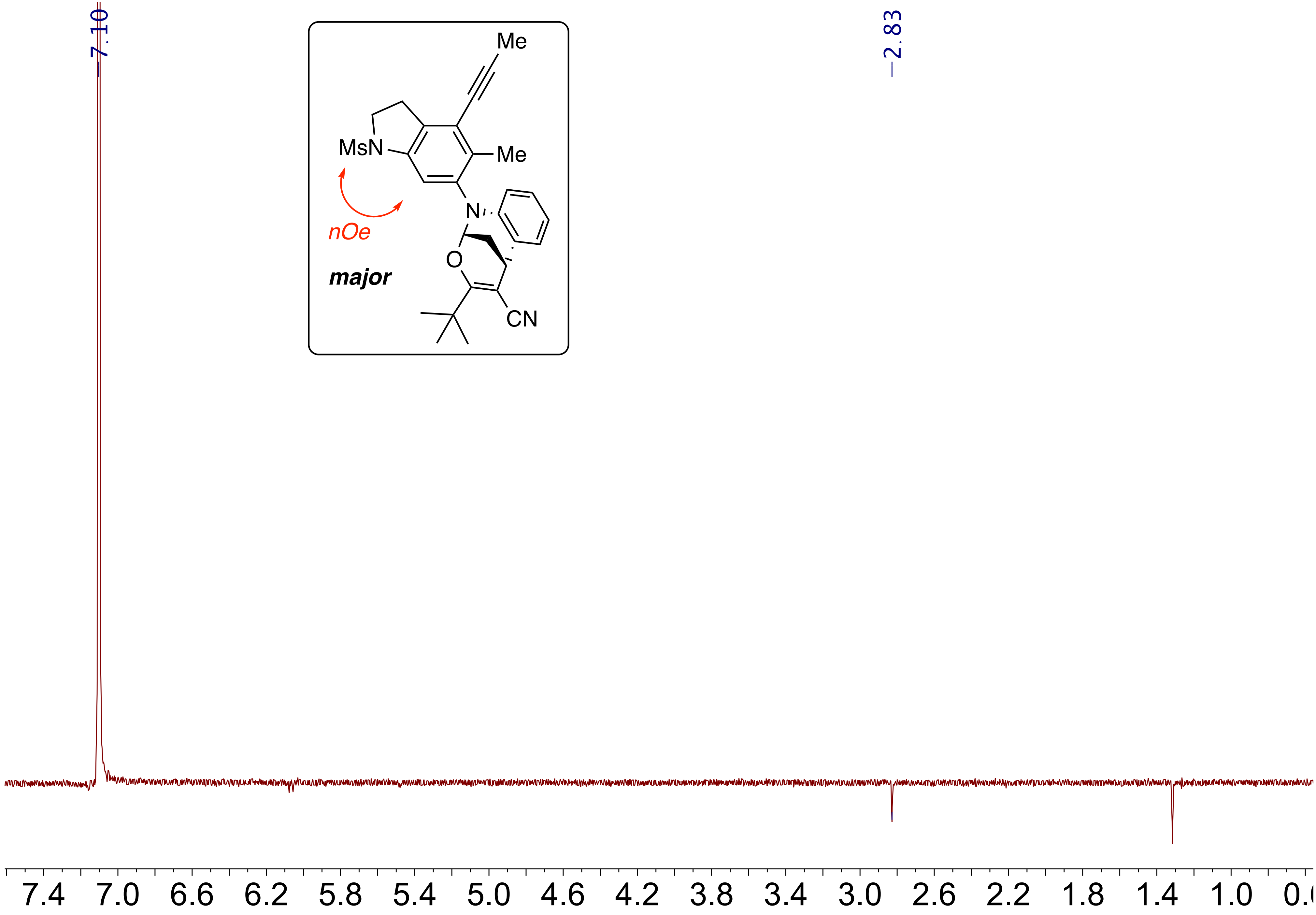
7.10
2.83
Me
MsN
Me
nOe
N
O
major
CN
7.4
7.0
6.6
6.2
5.8
5.4
5.0
4.6
4.2
3.8
3.4
3.0
2.6
2.2
1.8
1.4
1.0

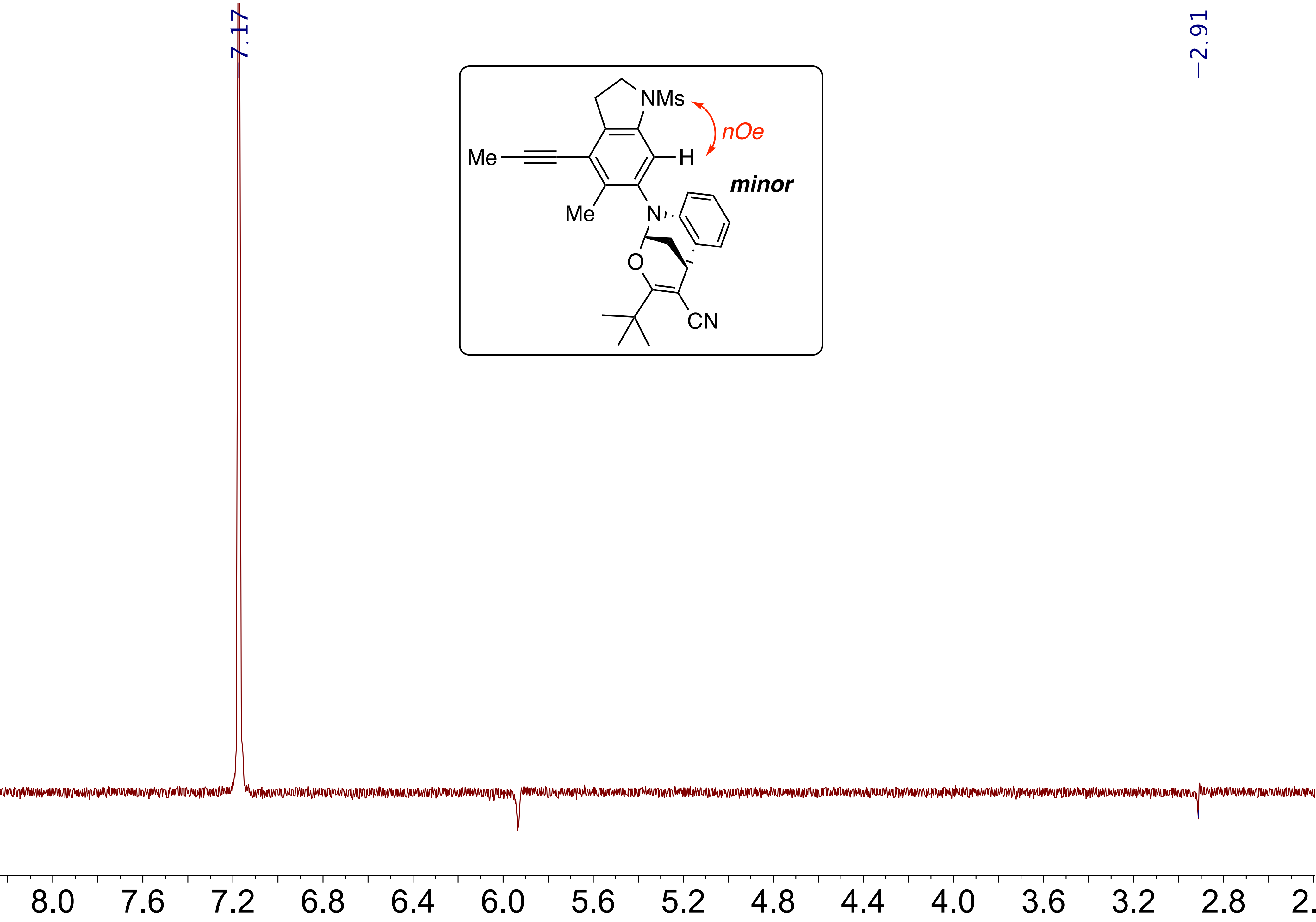
7.17
2.91
NMs
nOe
Me
H
minor
Me
N
O
CN
8.0
7.6
7.2
6.8
6.4
6.0
5.6
5.2
4.8
4.4
4.0
3.6
3.2
2.8

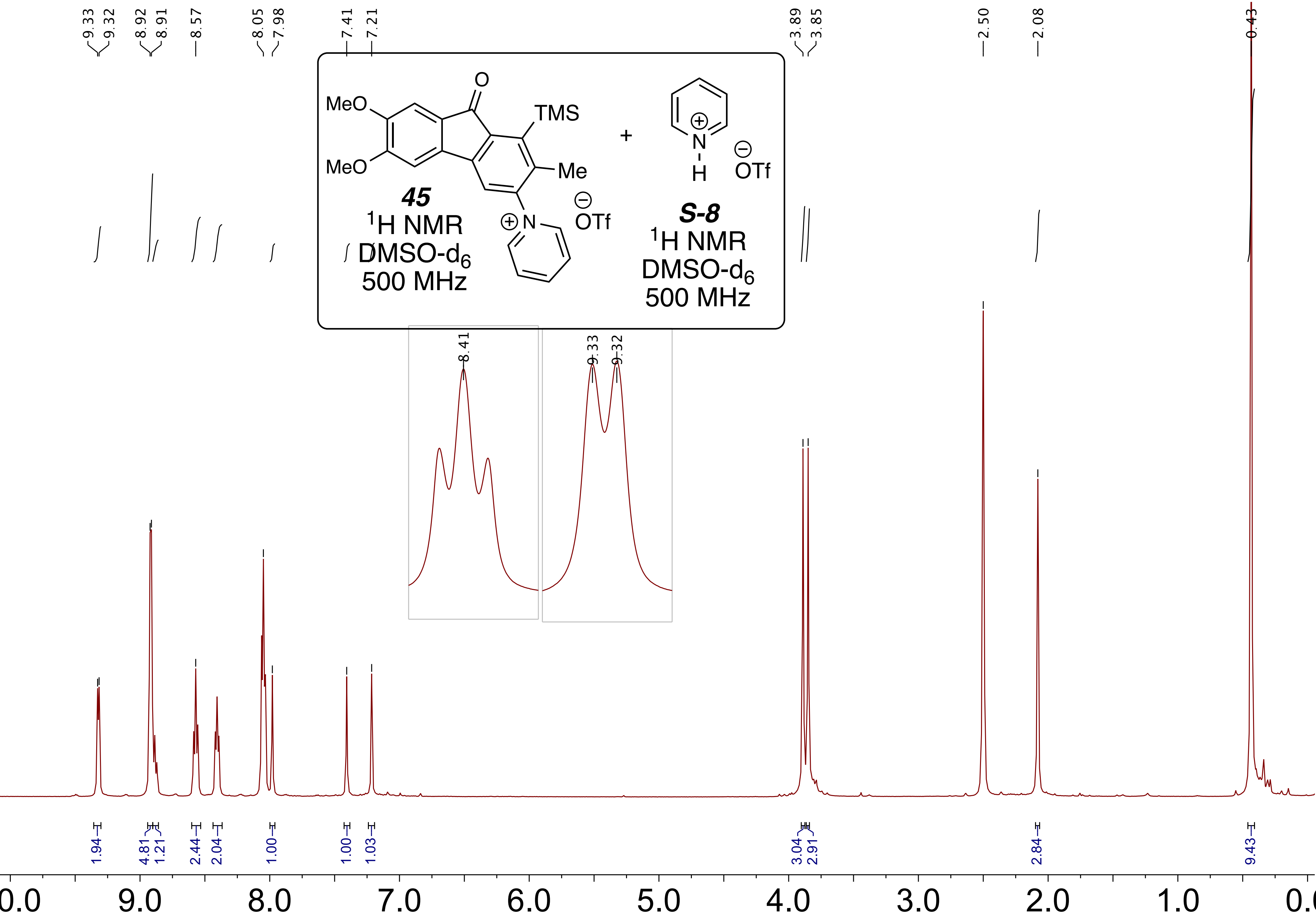
MeO
MeO
O
TMS
Me
OTf
N
45
¹H NMR
DMSO-d6
500 MHz
+
N
H
OTf
S-8
¹H NMR
DMSO-d6
500 MHz
9.33
9.32
8.92
8.91
8.57
8.05
7.98
7.41
7.21
3.89
3.85
2.50
2.08
0.43
8.41
1.94
4.81
1.21
2.44
2.04
1.00
1.00
1.03
3.04
2.91
2.84
9.43
0.0
9.0
8.0
7.0
6.0
5.0
4.0
3.0
2.0
1.0

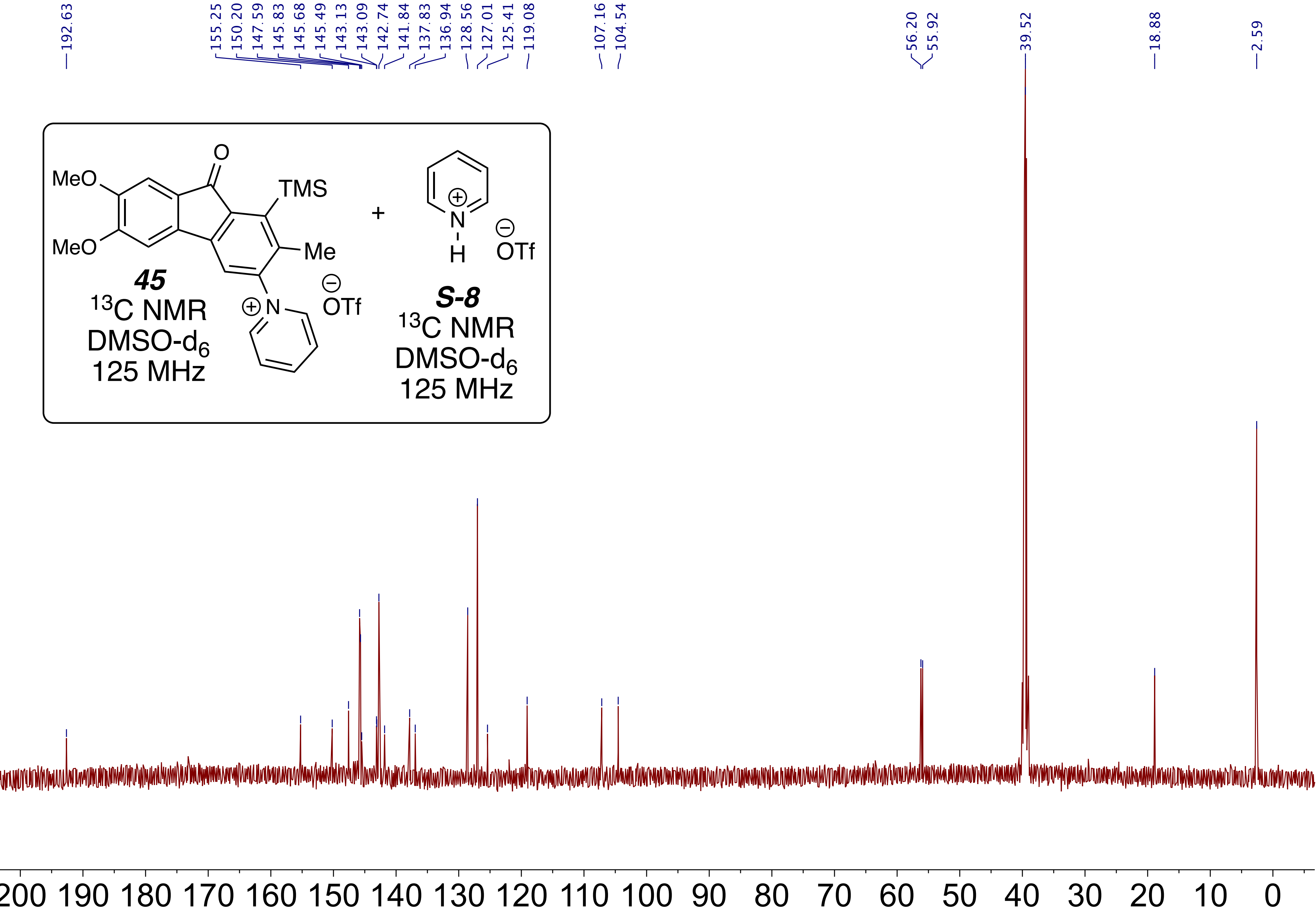
192.63
155.25
150.20
147.59
145.83
145.68
145.49
143.13
143.09
142.74
141.84
137.83
136.94
128.56
127.01
125.41
119.08
107.16
104.54
56.20
55.92
39.52
18.88
2.59
MeO
MeO
O
TMS
Me
N
OTf
+
N
H
OTf
45
^{13}C NMR
DMSO-d_6
125 MHz
S-8
^{13}C NMR
DMSO-d_6
125 MHz
200 190 180 170 160 150 140 130 120 110 100 90 80 70 60 50 40 30 20 10 0

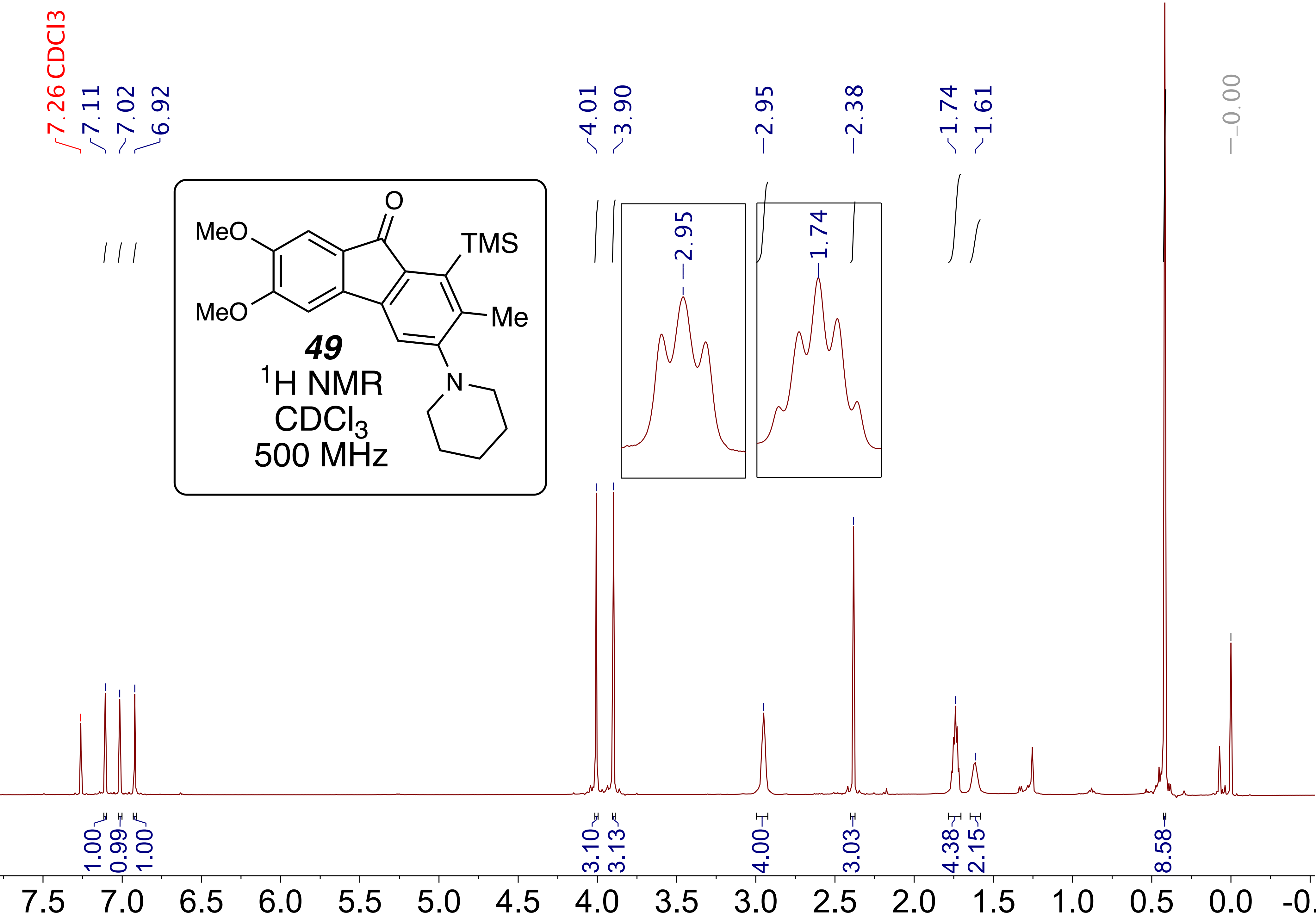
7.26 CDCl3
7.11
7.02
6.92
4.01
3.90
2.95
2.38
1.74
1.61
0.00
MeO
MeO
O
TMS
Me
N
49
1H NMR
CDCl3
500 MHz
2.95
1.74
1.00
0.99
1.00
3.10
3.13
4.00
3.03
4.38
2.15
8.58
7.5
7.0
6.5
6.0
5.5
5.0
4.5
4.0
3.5
3.0
2.5
2.0
1.5
1.0
0.5
0.0

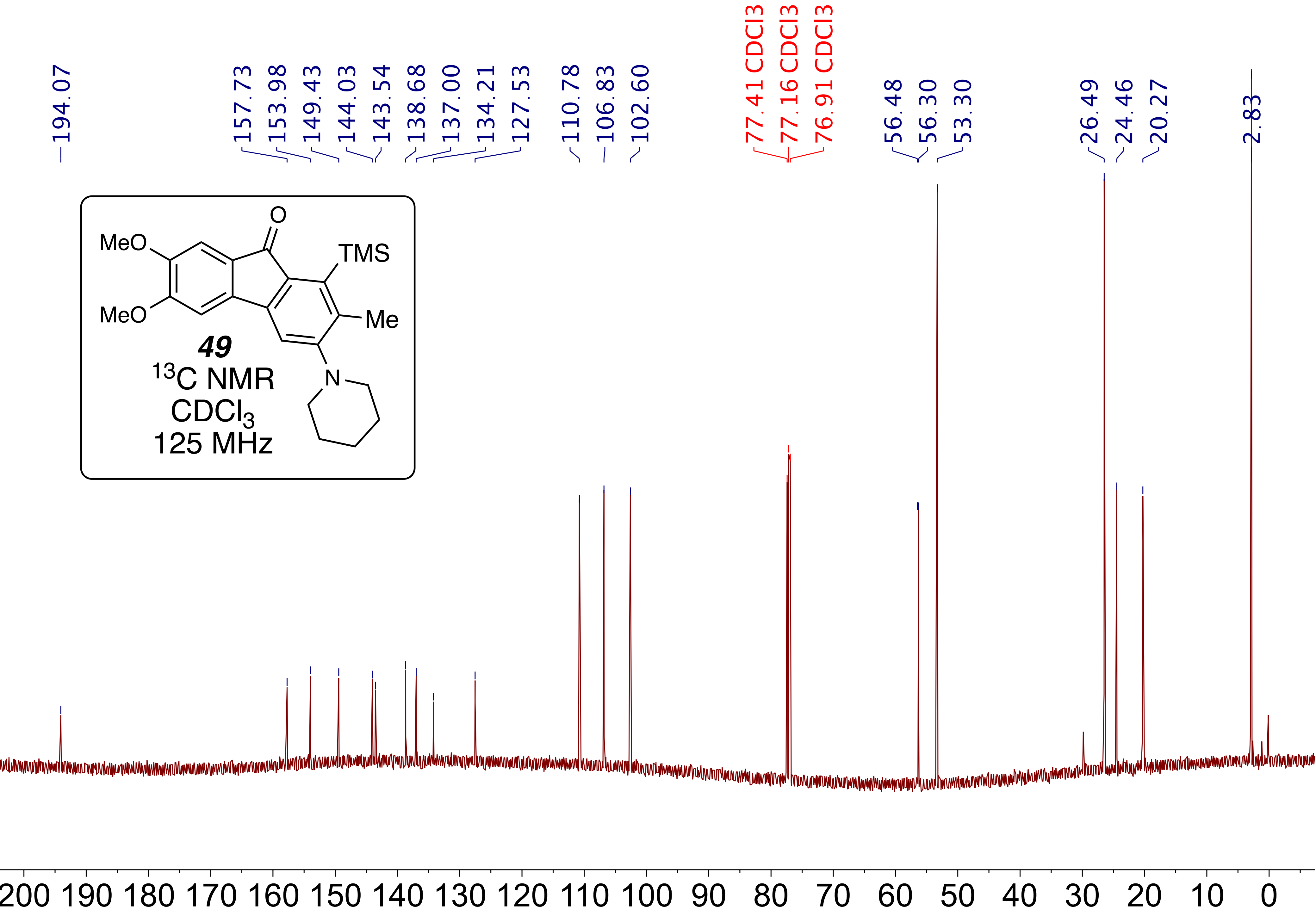

194.07
157.73
153.98
149.43
144.03
143.54
138.68
137.00
134.21
127.53
110.78
106.83
102.60
77.41 CDCl3
77.16 CDCl3
76.91 CDCl3
56.48
56.30
53.30
26.49
24.46
20.27
2.83
O
MeO
MeO
TMS
Me
N
49
13C NMR
CDCl3
125 MHz
200 190 180 170 160 150 140 130 120 110 100 90 80 70 60 50 40 30 20 10 0

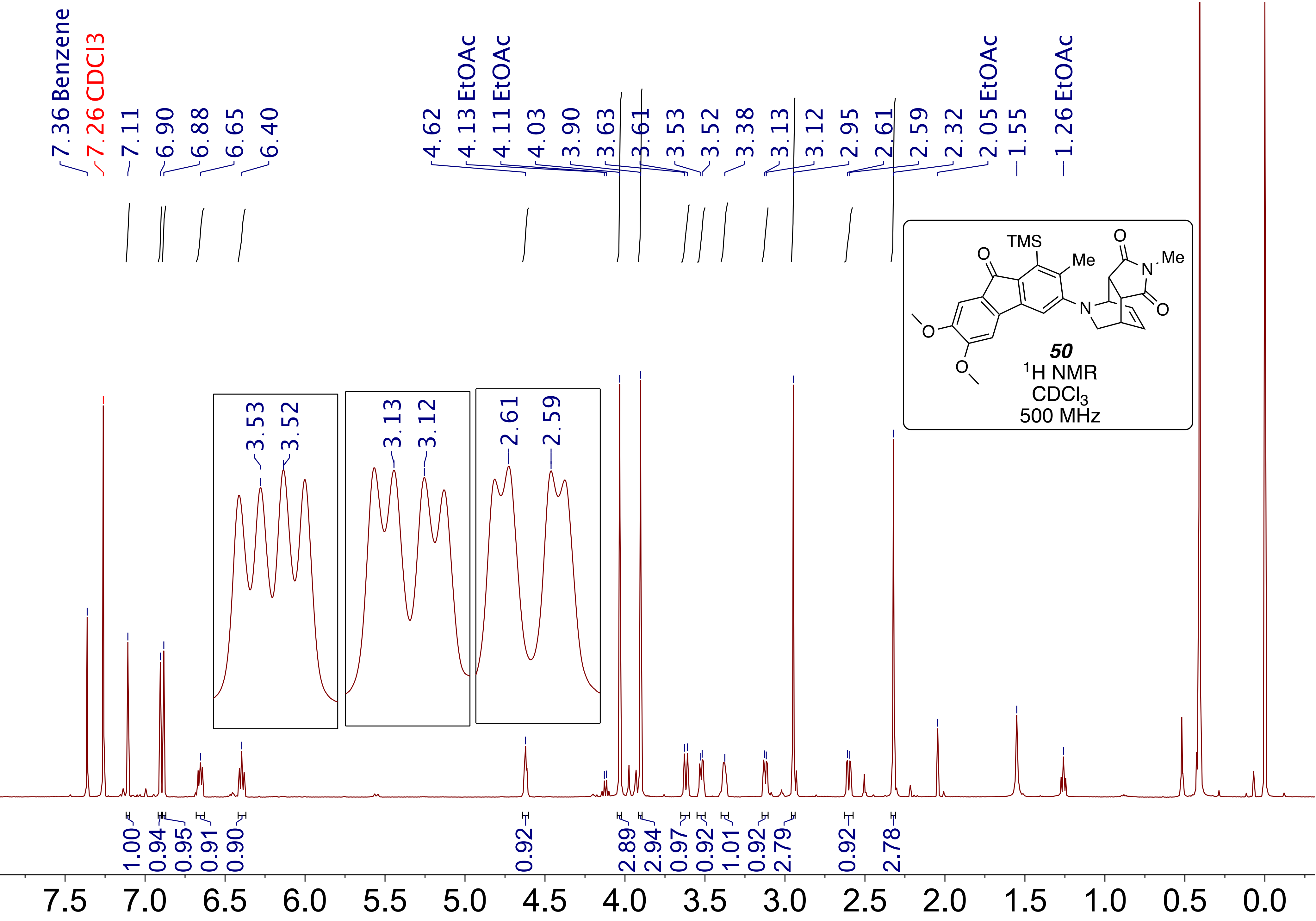
7.36 Benzene
7.26 CDCl3
7.11
6.90
6.88
6.65
6.40
4.62
4.13 EtOAc
4.11 EtOAc
4.03
3.90
3.63
3.61
3.53
3.52
3.38
3.13
3.12
2.95
2.61
2.59
2.32
2.05 EtOAc
1.55
1.26 EtOAc
TMS
Me
O
N
Me
O
O
O
O
50
1H NMR
CDCl3
500 MHz
1.00
0.94
0.95
0.91
0.90
0.92
2.89
2.94
0.97
0.92
1.01
0.92
2.79
0.92
2.78
7.5 7.0 6.5 6.0 5.5 5.0 4.5 4.0 3.5 3.0 2.5 2.0 1.5 1.0 0.5 0.0

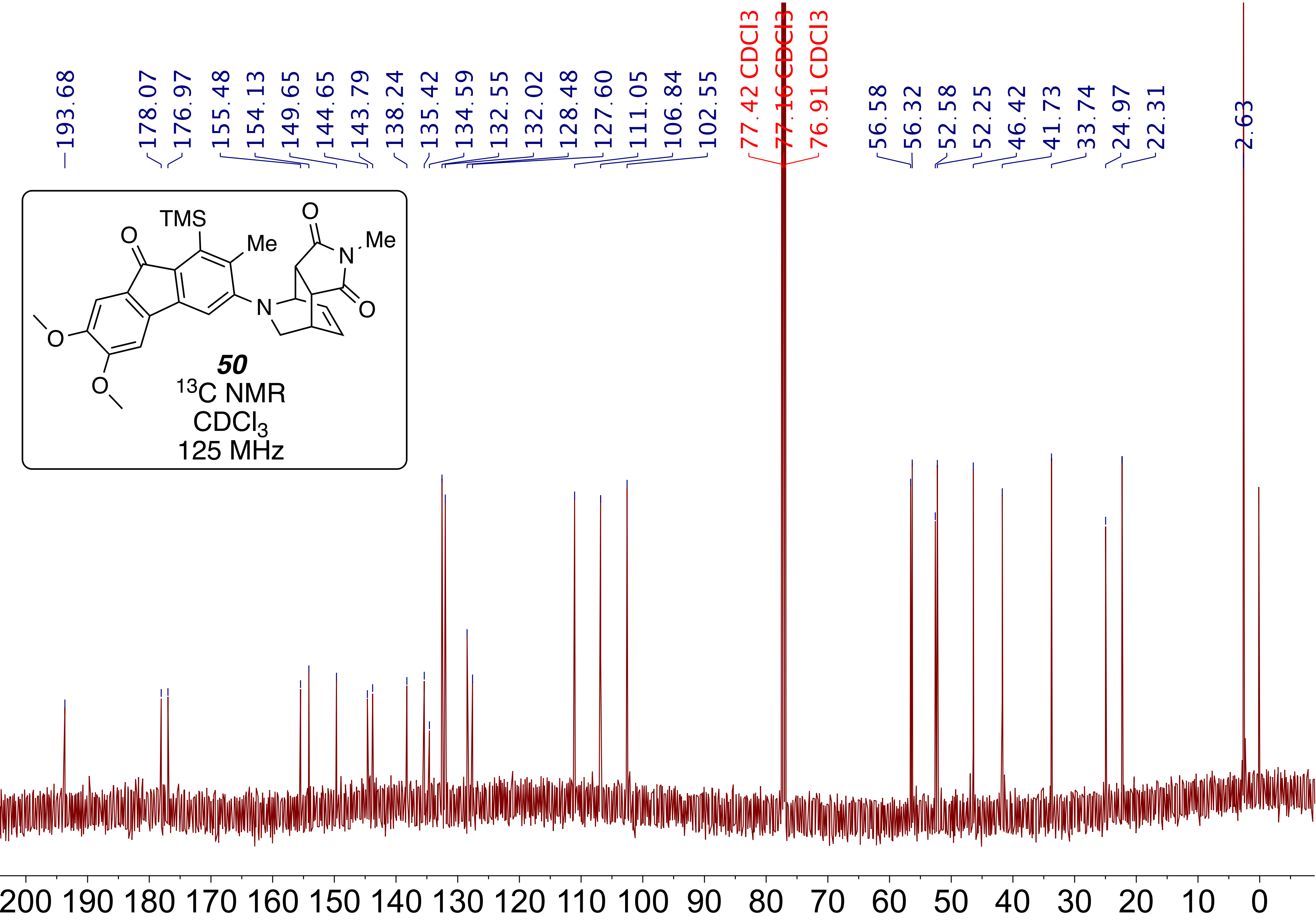
193.68
178.07
176.97
155.48
154.13
149.65
144.65
143.79
138.24
135.42
134.59
132.55
132.02
128.48
127.60
111.05
106.84
102.55
77.42 CDCl3
77.16 CDCl3
76.91 CDCl3
56.58
56.32
52.58
52.25
46.42
41.73
33.74
24.97
22.31
2.63
TMS
Me
O
N
Me
50
13C NMR
CDCl3
125 MHz
200 190 180 170 160 150 140 130 120 110 100 90 80 70 60 50 40 30 20 10 0

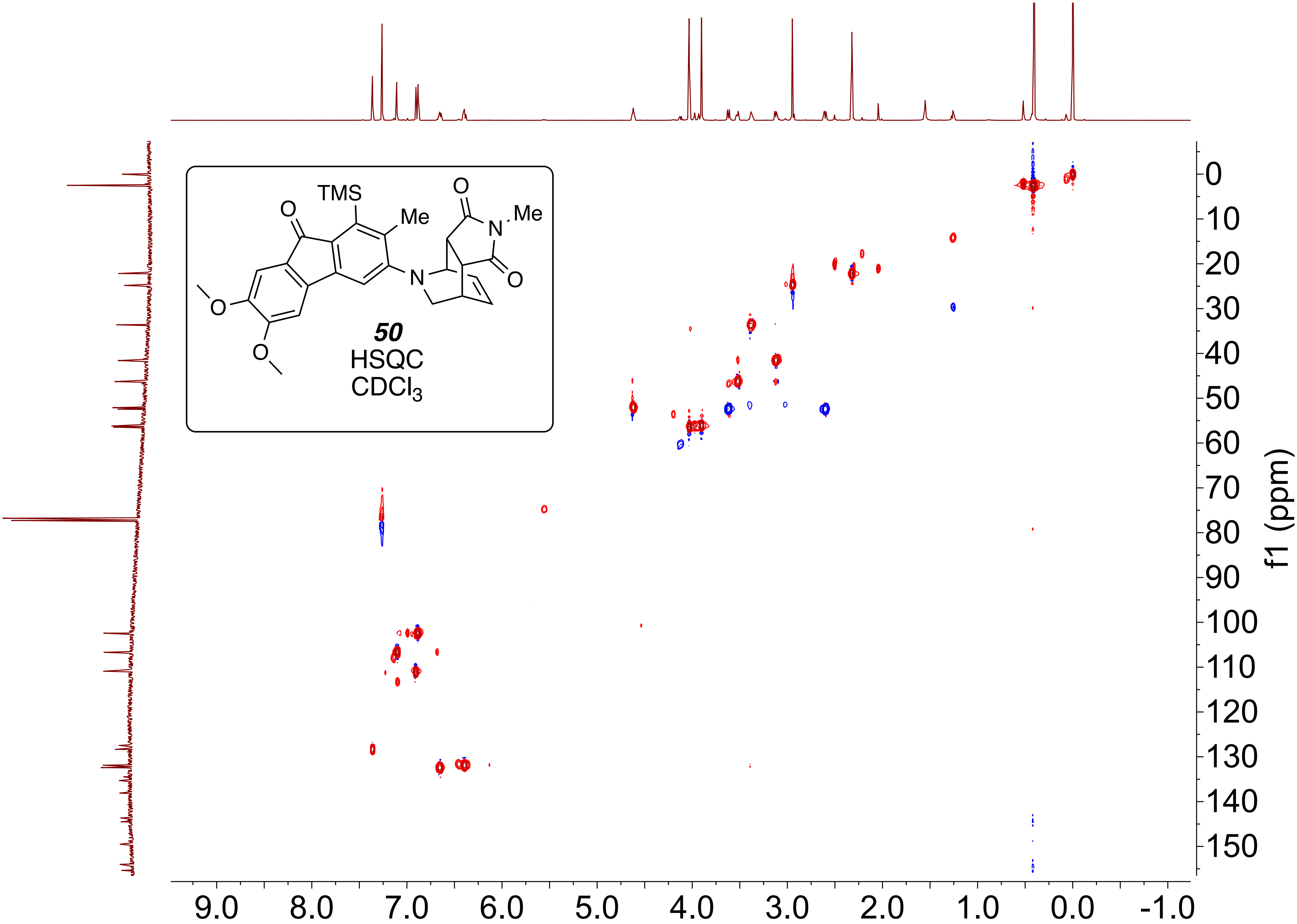
TMS
O
Me
O
N
Me
O
N
O
O
50
HSQC
CDCl3
f1 (ppm)
9.0 8.0 7.0 6.0 5.0 4.0 3.0 2.0 1.0 0.0 -1.0
0 10 20 30 40 50 60 70 80 90 100 110 120 130 140 150

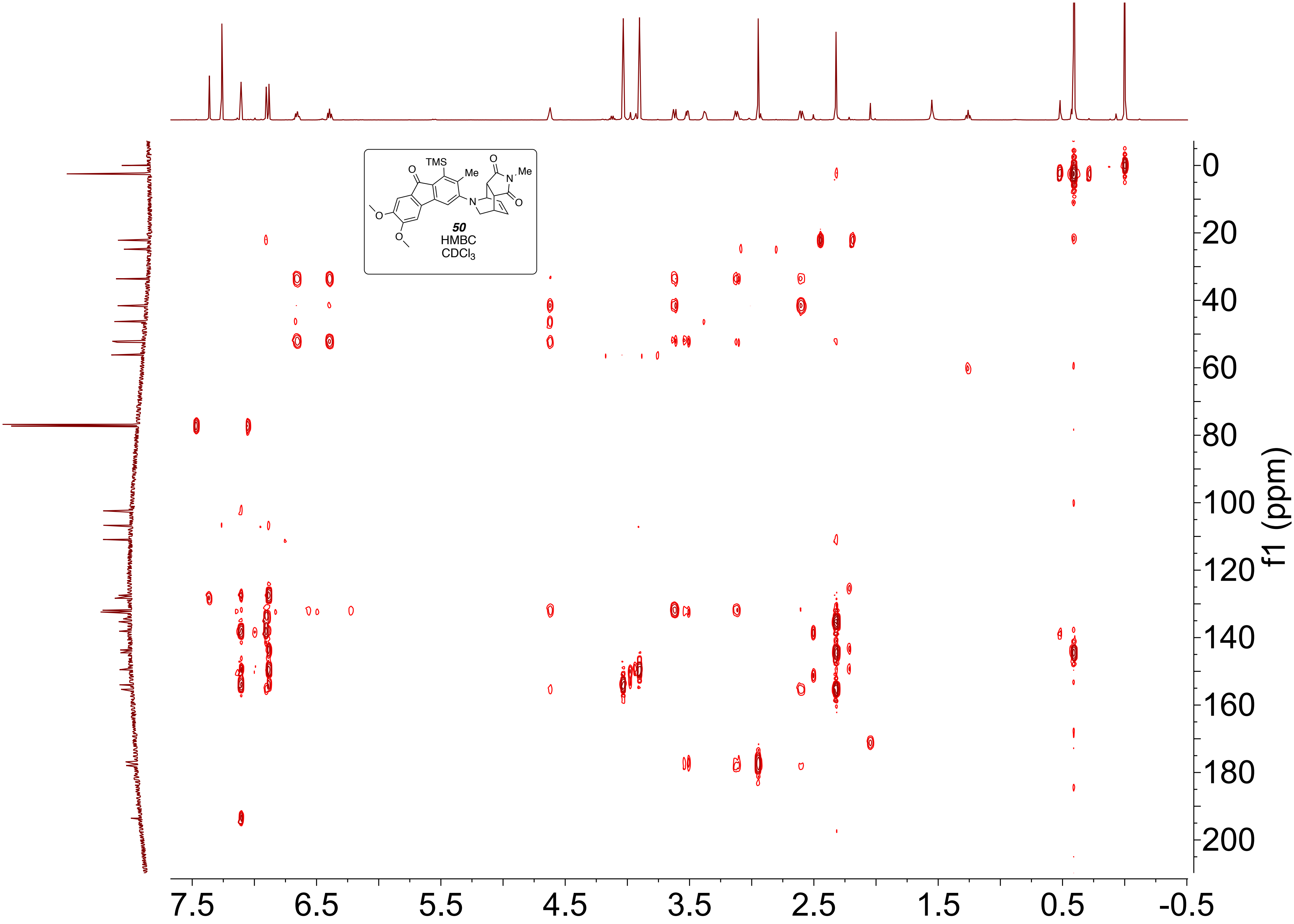
TMS
Me
N
Me
50
HMBC
CDCl3
f1 (ppm)
0
20
40
60
80
100
120
140
160
180
200
7.5
6.5
5.5
4.5
3.5
2.5
1.5
0.5
-0.5

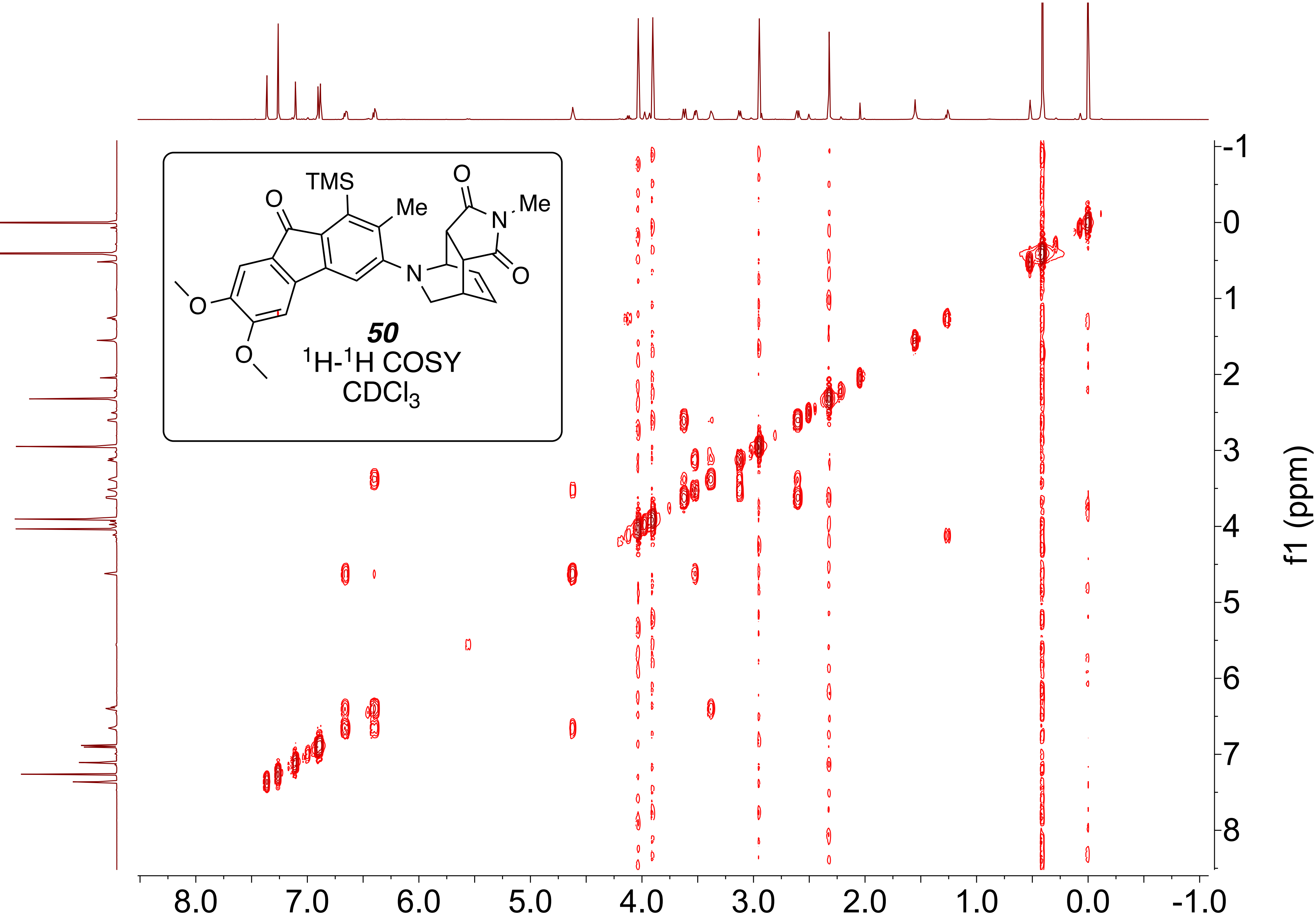
TMS
Me
O
N
Me
O
O
O
O
N
50
^{1}H-^{1}H COSY
$CDCl_3$
f1 (ppm)
-1
0
1
2
3
4
5
6
7
8
8.0
7.0
6.0
5.0
4.0
3.0
2.0
1.0
0.0
-1.0

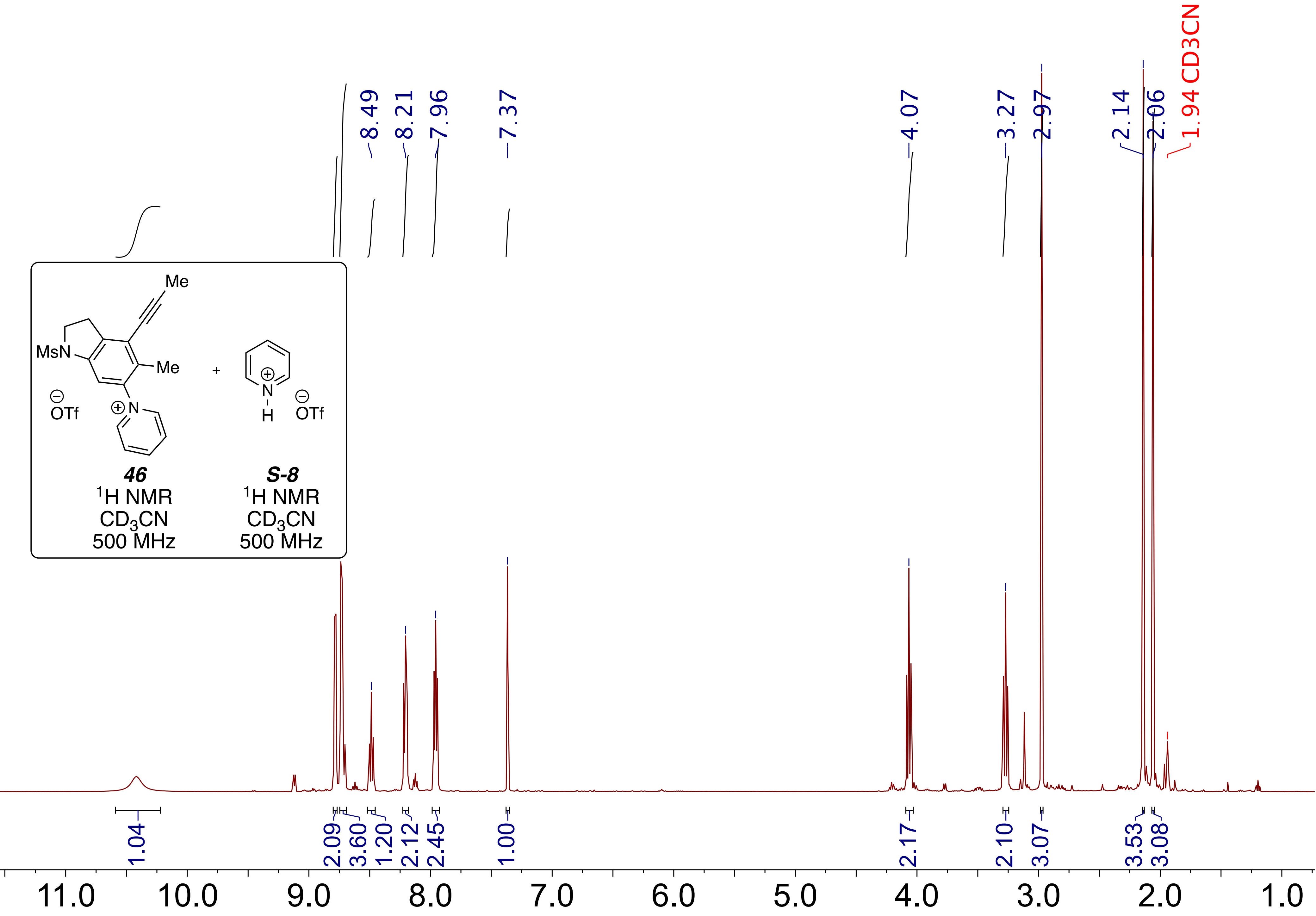
46
¹H NMR
CD3CN
500 MHz
S-8
¹H NMR
CD3CN
500 MHz
8.49
8.21
7.96
7.37
4.07
3.27
2.97
2.14
2.06
1.94 CD3CN
1.04
2.09
3.60
1.20
2.12
2.45
1.00
2.17
2.10
3.07
3.53
3.08
11.0
10.0
9.0
8.0
7.0
6.0
5.0
4.0
3.0
2.0
1.0

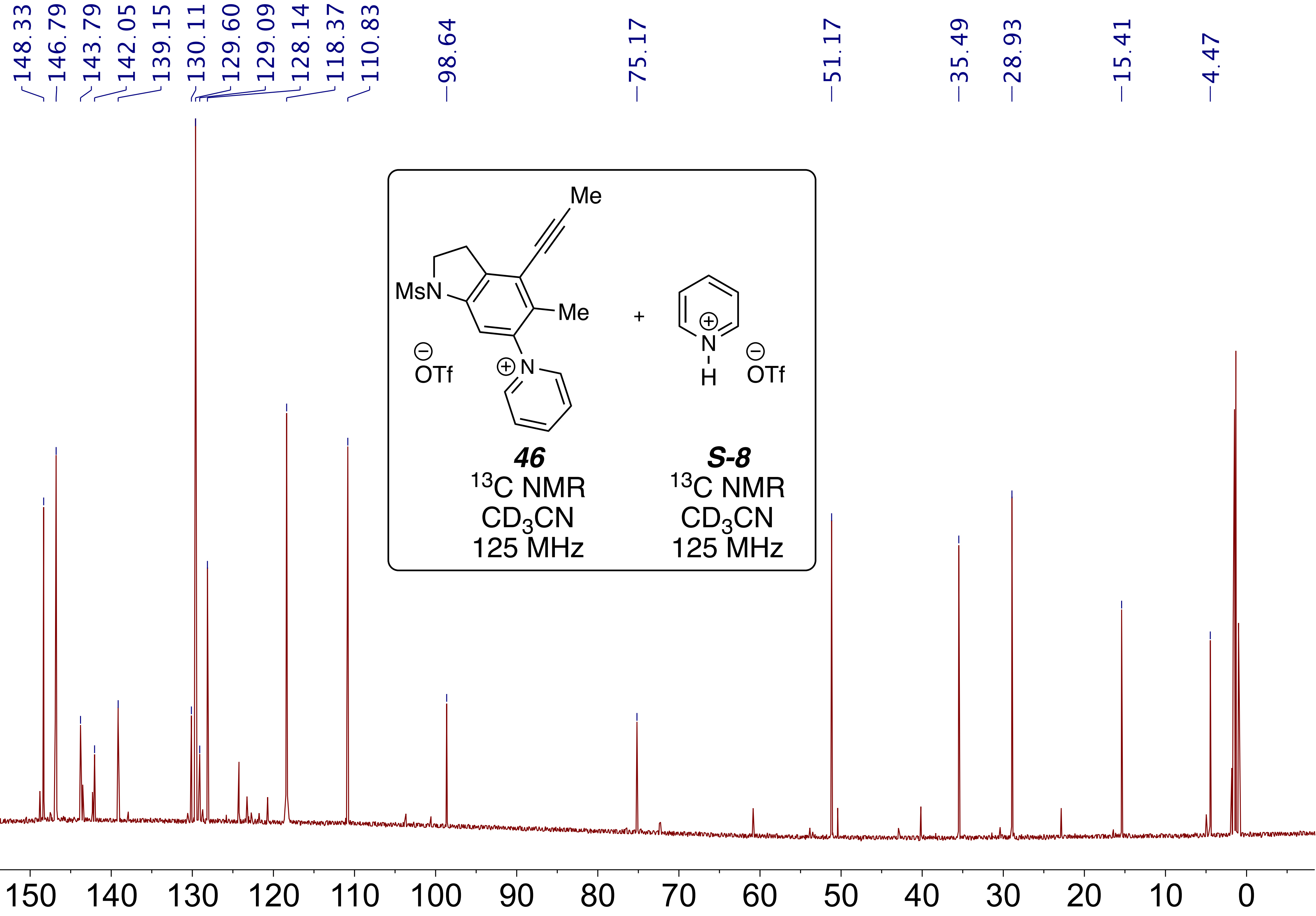
148.33
146.79
143.79
142.05
139.15
130.11
129.60
129.09
128.14
118.37
110.83
98.64
75.17
51.17
35.49
28.93
15.41
4.47
Me
MsN
Me
OTf
N
+
N
H
OTf
46
^{13}C NMR
CD_3CN
125 MHz
S-8
^{13}C NMR
CD_3CN
125 MHz
150 140 130 120 110 100 90 80 70 60 50 40 30 20 10 0

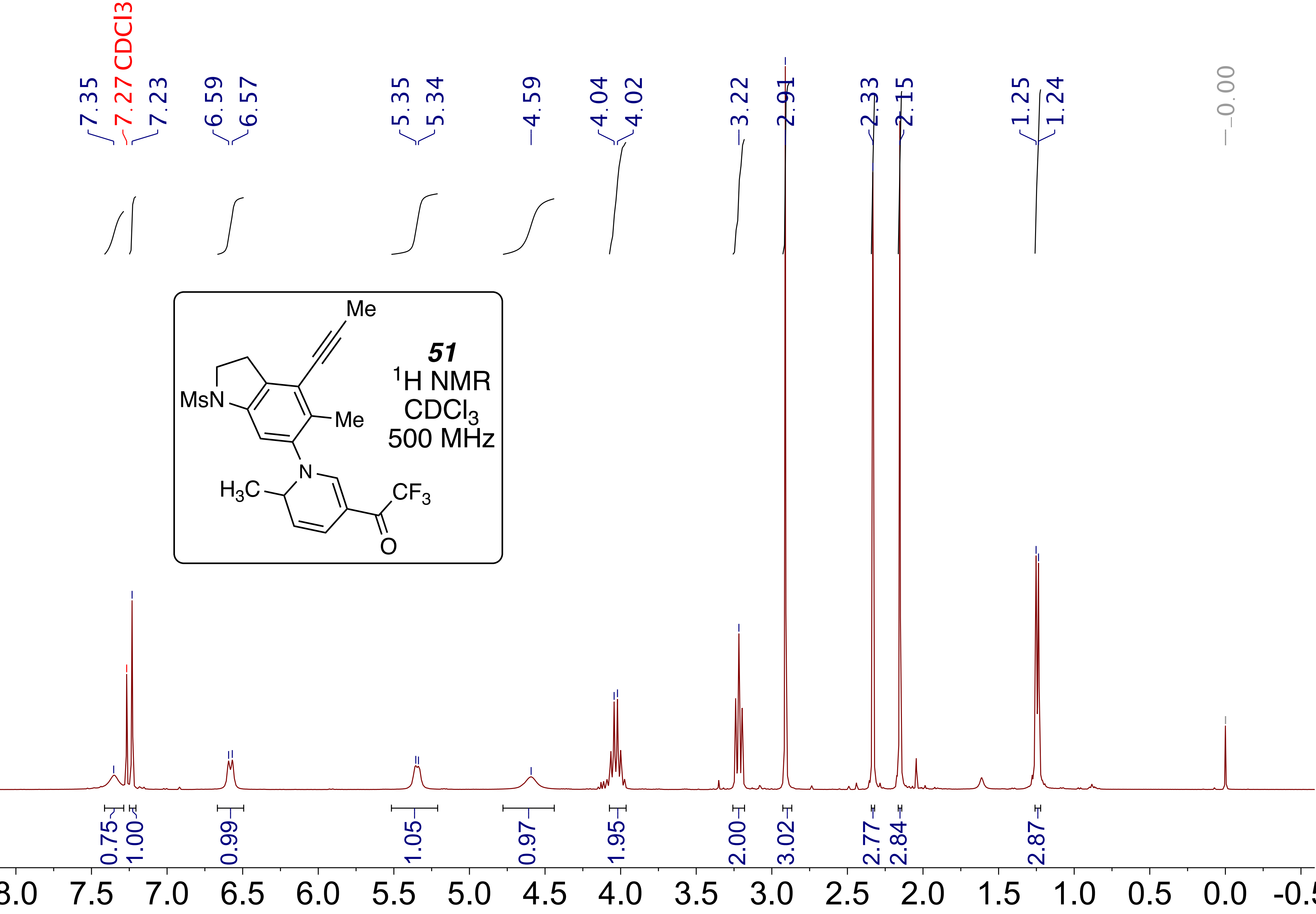
7.35
7.27 CDCl3
7.23
6.59
6.57
5.35
5.34
4.59
4.04
4.02
3.22
2.91
2.33
2.15
1.25
1.24
0.00
Me
MsN
Me
N
H_3C
CF_3
O
51
^{1}H NMR
$CDCl_3$
500 MHz
0.75
1.00
0.99
1.05
0.97
1.95
2.00
3.02
2.77
2.84
2.87
8.0
7.5
7.0
6.5
6.0
5.5
5.0
4.5
4.0
3.5
3.0
2.5
2.0
1.5
1.0
0.5
0.0
-0.5

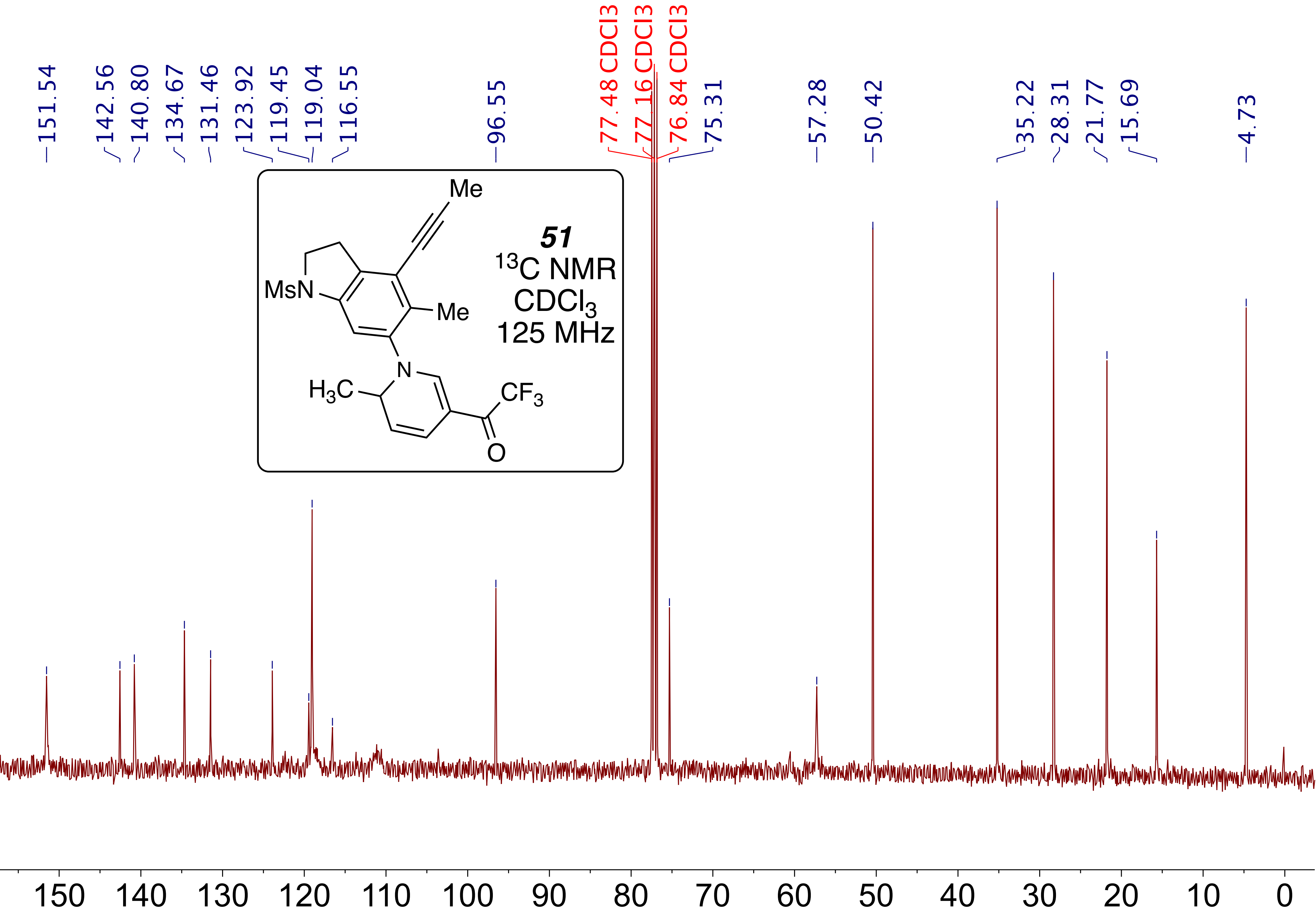
151.54
142.56
140.80
134.67
131.46
123.92
119.45
119.04
116.55
96.55
77.48 CDCl3
77.16 CDCl3
76.84 CDCl3
75.31
57.28
50.42
35.22
28.31
21.77
15.69
4.73
Me
MsN
Me
N
H3C
CF3
O
51
13C NMR
CDCl3
125 MHz
150 140 130 120 110 100 90 80 70 60 50 40 30 20 10 0

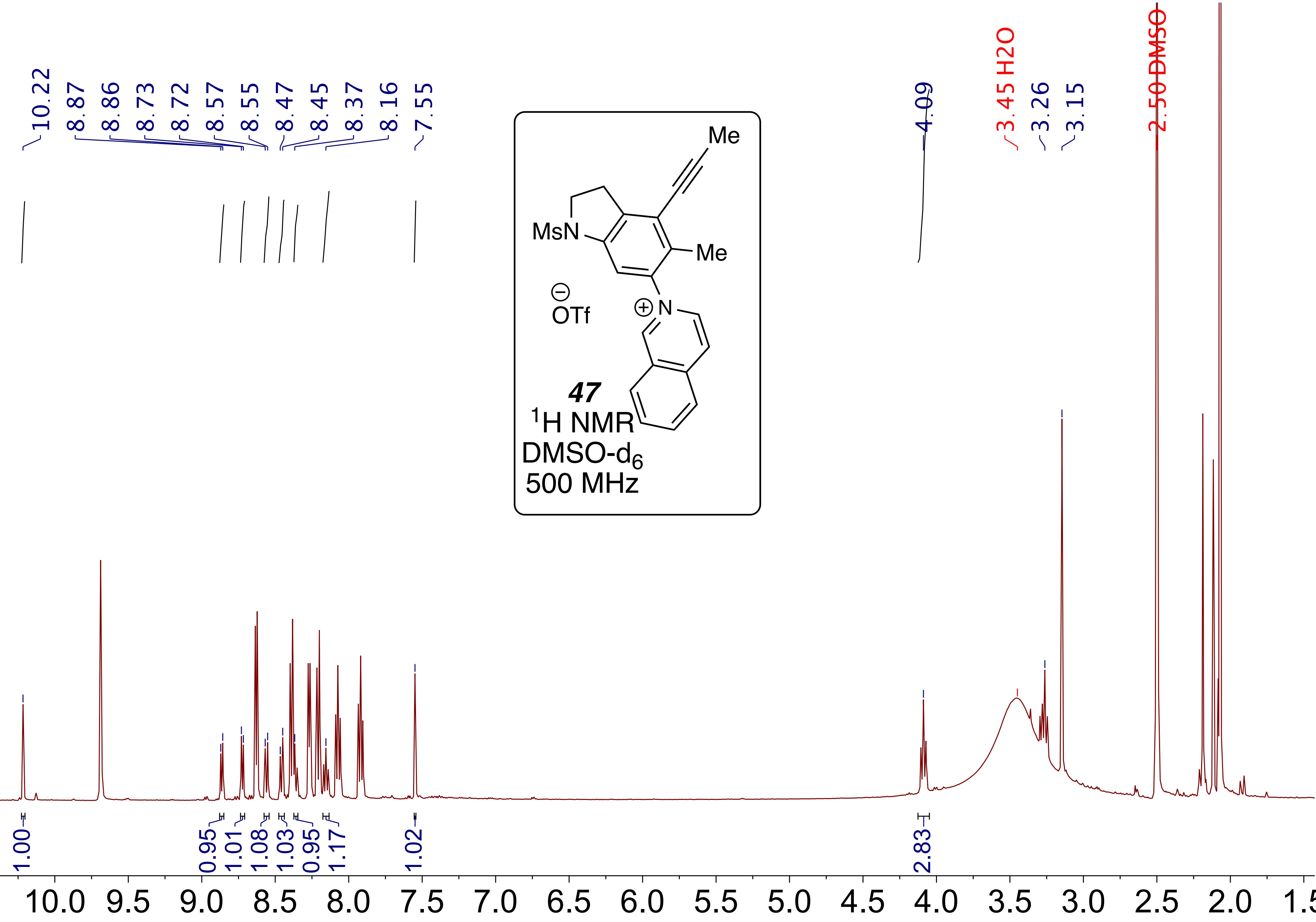

10.22
8.87
8.86
8.73
8.72
8.57
8.55
8.47
8.45
8.37
8.16
7.55
4.09
3.45 H2O
3.26
3.15
2.50 DMSO
Me
MsN
Me
OTf
N
47
1H NMR
DMSO-d6
500 MHz
1.00
0.95
1.01
1.08
1.03
0.95
1.17
1.02
2.83
10.0
9.5
9.0
8.5
8.0
7.5
7.0
6.5
6.0
5.5
5.0
4.5
4.0
3.5
3.0
2.5
2.0

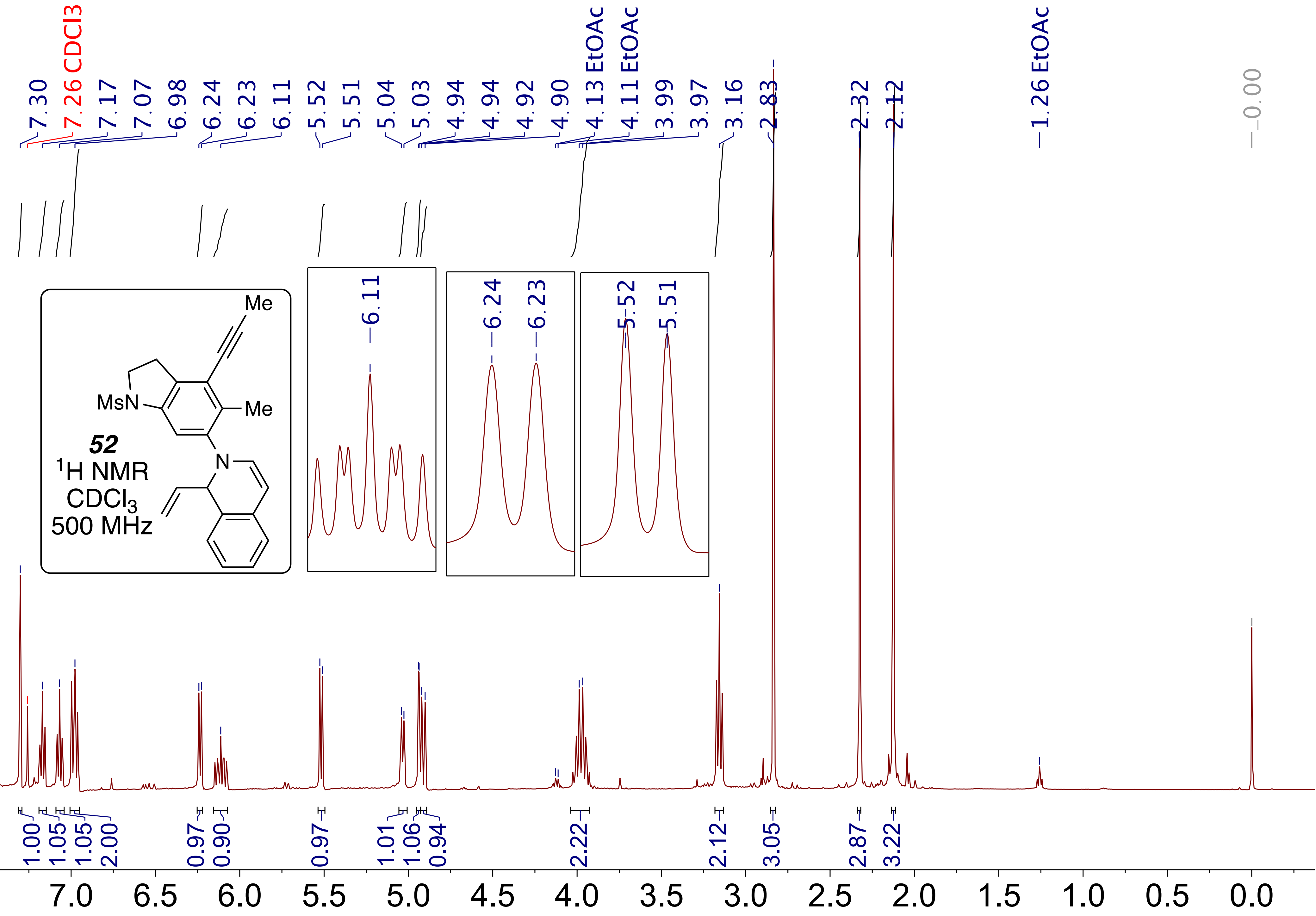
7.30
7.26 CDCl3
7.17
7.07
6.98
6.24
6.23
6.11
5.52
5.51
5.04
5.03
4.94
4.94
4.92
4.90
4.13 EtOAc
4.11 EtOAc
3.99
3.97
3.16
2.83
2.32
2.12
1.26 EtOAc
0.00
Me
MsN
Me
N
52
1H NMR
CDCl3
500 MHz
1.00
1.05
1.05
2.00
0.97
0.90
0.97
1.01
1.06
0.94
2.22
2.12
3.05
2.87
3.22
7.0
6.5
6.0
5.5
5.0
4.5
4.0
3.5
3.0
2.5
2.0
1.5
1.0
0.5
0.0

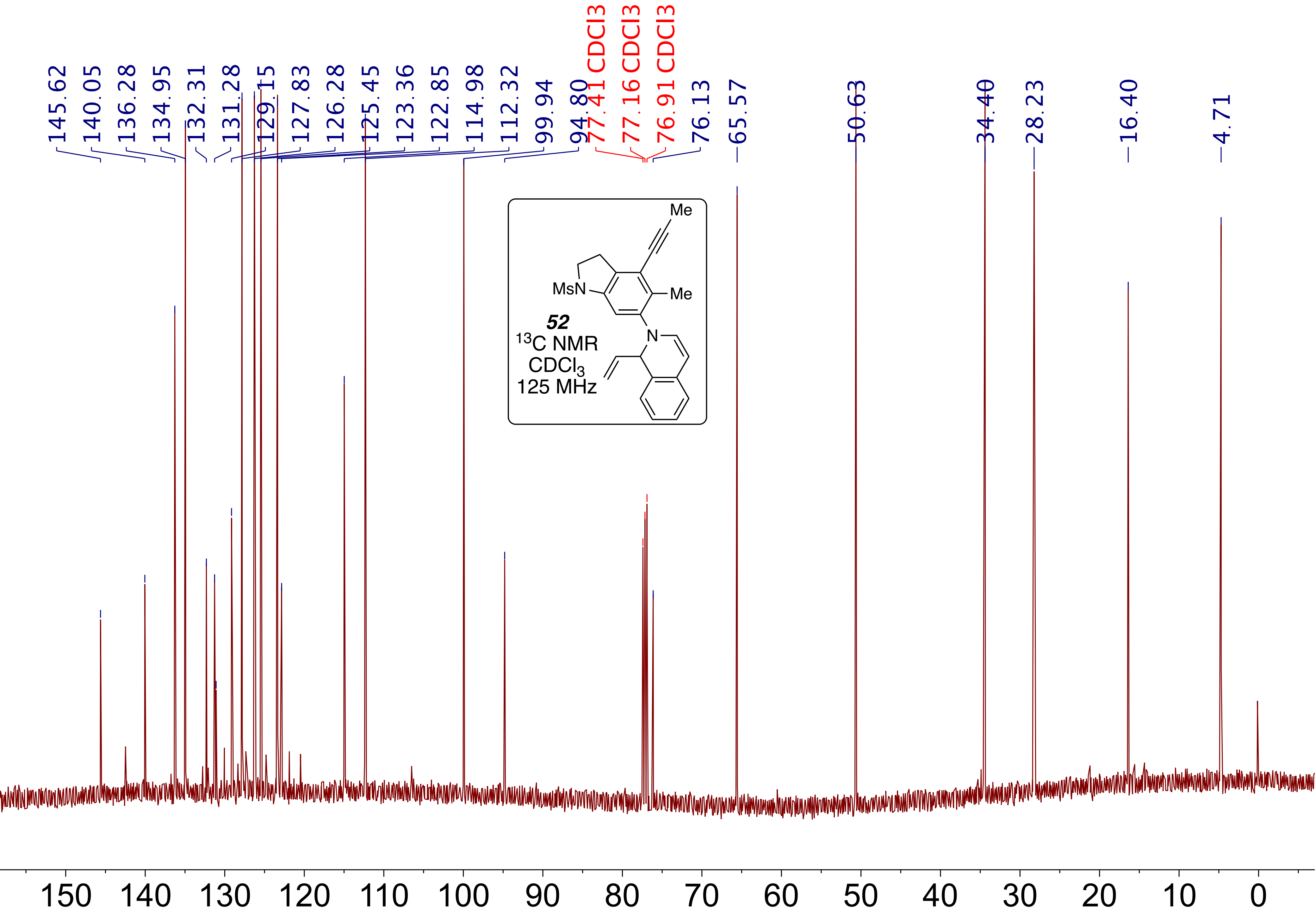
145.62
140.05
136.28
134.95
132.31
131.28
129.15
127.83
126.28
125.45
123.36
122.85
114.98
112.32
99.94
94.80
77.41 CDCl3
77.16 CDCl3
76.91 CDCl3
76.13
65.57
50.63
34.40
28.23
16.40
4.71
Me
MsN
Me
N
52
13C NMR
CDCl3
125 MHz
150
140
130
120
110
100
90
80
70
60
50
40
30
20
10
0

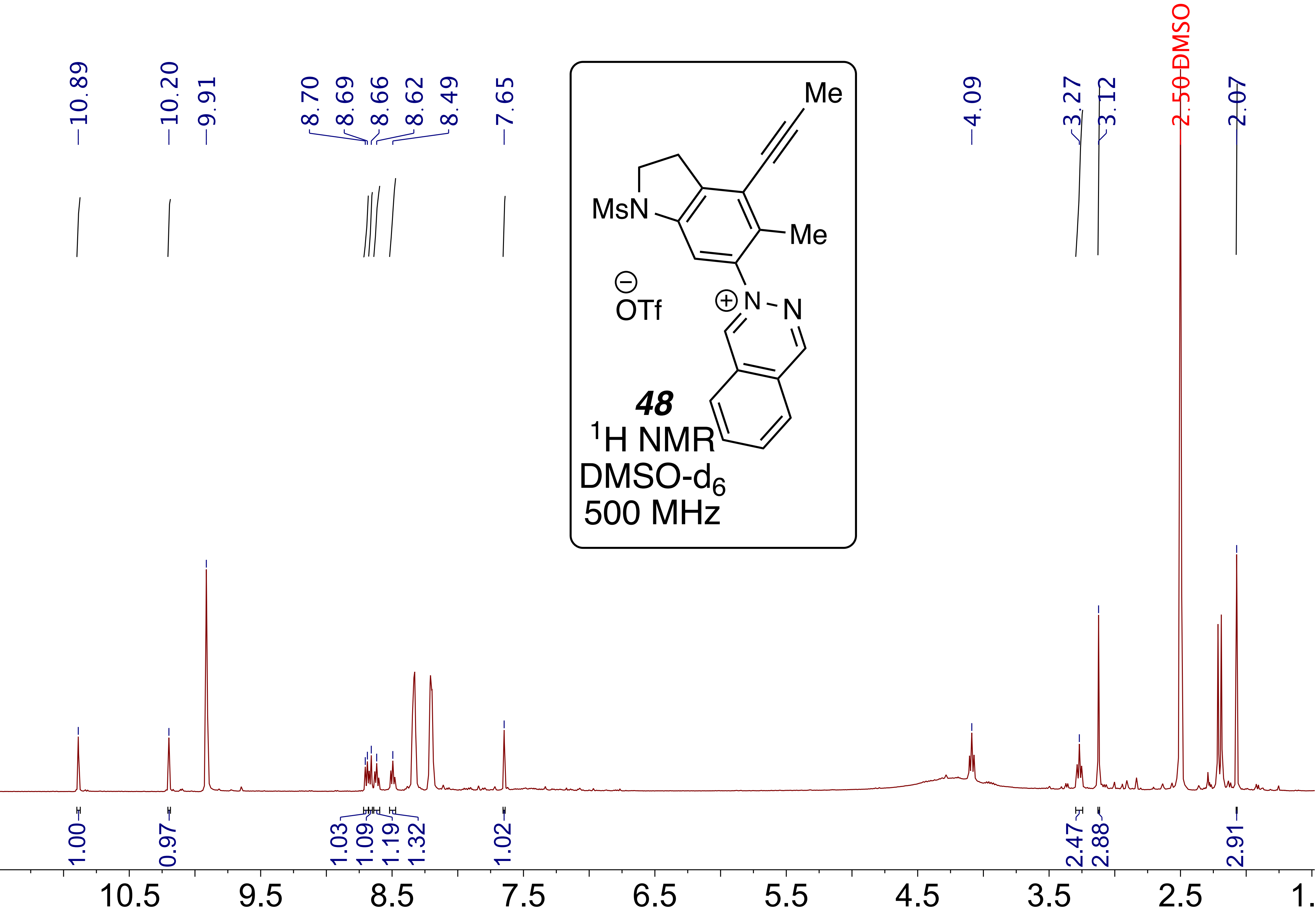
48
1H NMR
DMSO-d6
500 MHz
MsN
Me
Me
OTf
N
N
2.50 DMSO

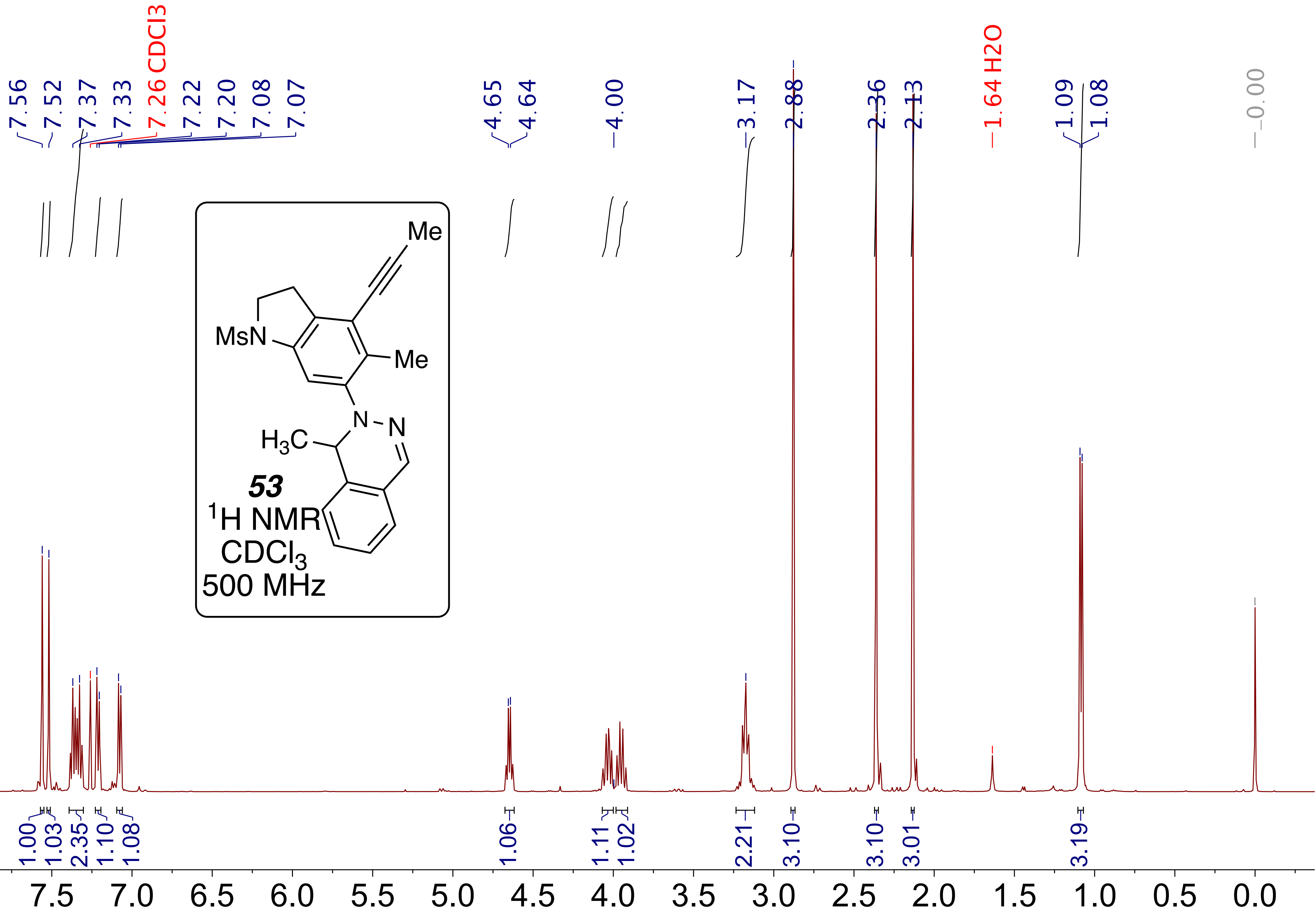
53
^{1}H NMR
$CDCl_3$
500 MHz
MsN
Me
Me
H_3C
N
N
7.56
7.52
7.37
7.33
7.26 CDCl3
7.22
7.20
7.08
7.07
4.65
4.64
4.00
3.17
2.88
2.36
2.13
1.64 H2O
1.09
1.08
0.00
1.00
1.03
2.35
1.10
1.08
1.06
1.11
1.02
2.21
3.10
3.10
3.01
3.19
7.5
7.0
6.5
6.0
5.5
5.0
4.5
4.0
3.5
3.0
2.5
2.0
1.5
1.0
0.5
0.0

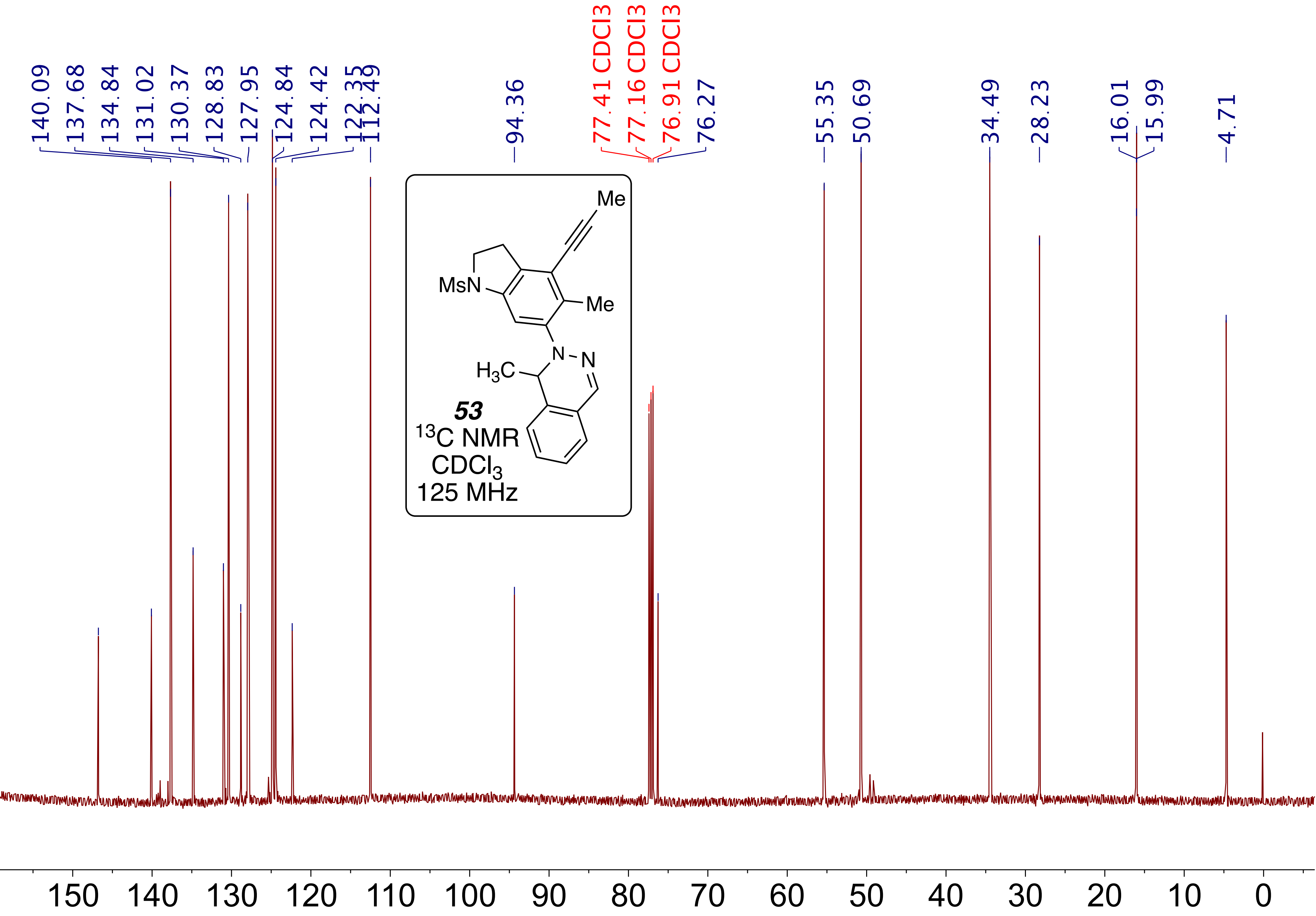
140.09
137.68
134.84
131.02
130.37
128.83
127.95
124.84
124.42
122.35
112.49
94.36
77.41 CDCl3
77.16 CDCl3
76.91 CDCl3
76.27
55.35
50.69
34.49
28.23
16.01
15.99
4.71
Me
MsN
Me
N
N
H_3C
53
^{13}C NMR
$CDCl_3$
125 MHz
150
140
130
120
110
100
90
80
70
60
50
40
30
20
10
0

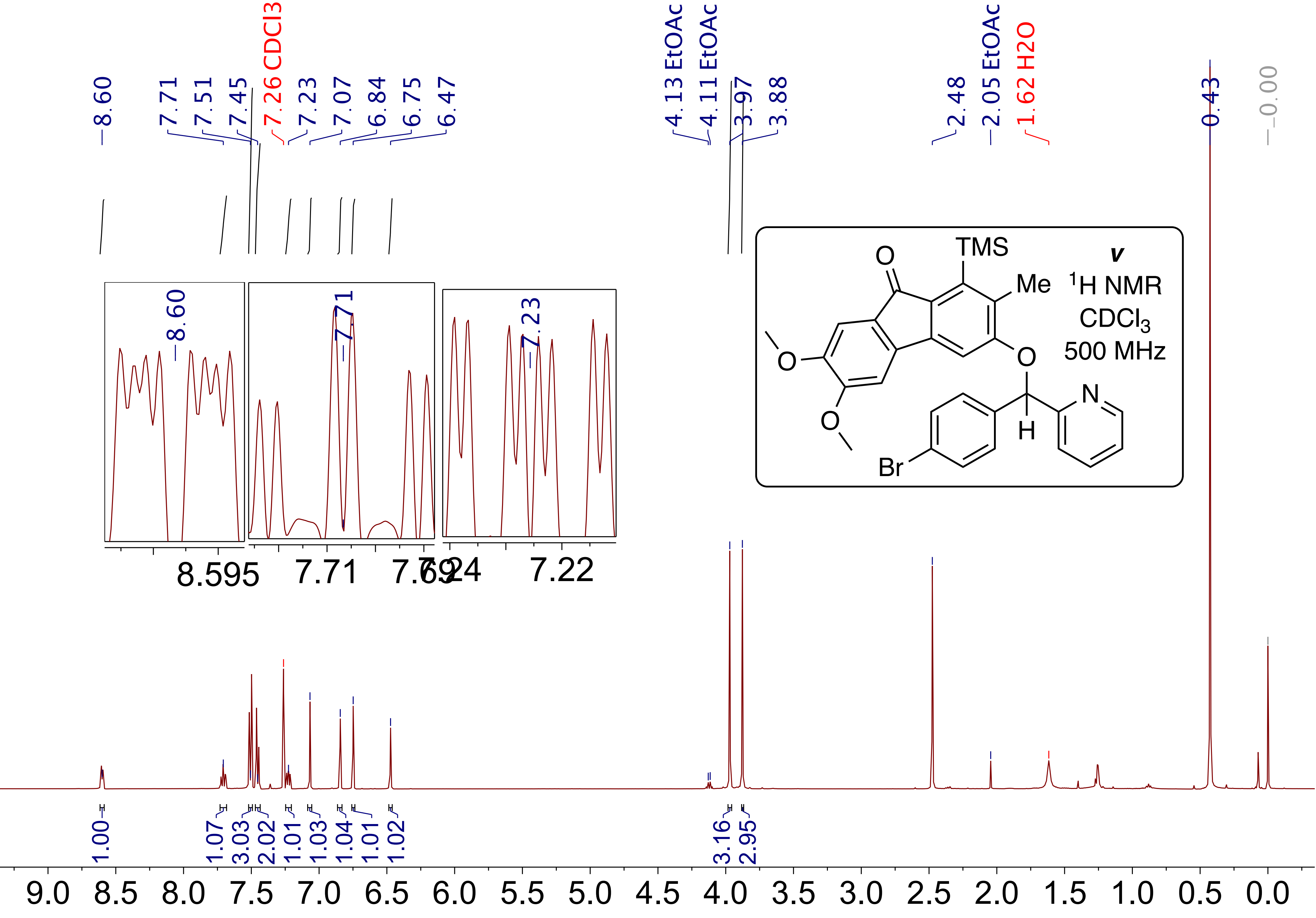
v
^{1}H NMR
CDCl3
500 MHz
TMS
Me
O
N
H
Br
8.60
7.71
7.51
7.45
7.26 CDCl3
7.23
7.07
6.84
6.75
6.47
4.13 EtOAc
4.11 EtOAc
3.97
3.88
2.48
2.05 EtOAc
1.62 H2O
0.43
0.00
1.00
1.07
3.03
2.02
1.01
1.03
1.04
1.01
1.02
3.16
2.95
8.595
7.71
7.69
7.24
7.22
9.0 8.5 8.0 7.5 7.0 6.5 6.0 5.5 5.0 4.5 4.0 3.5 3.0 2.5 2.0 1.5 1.0 0.5 0.0

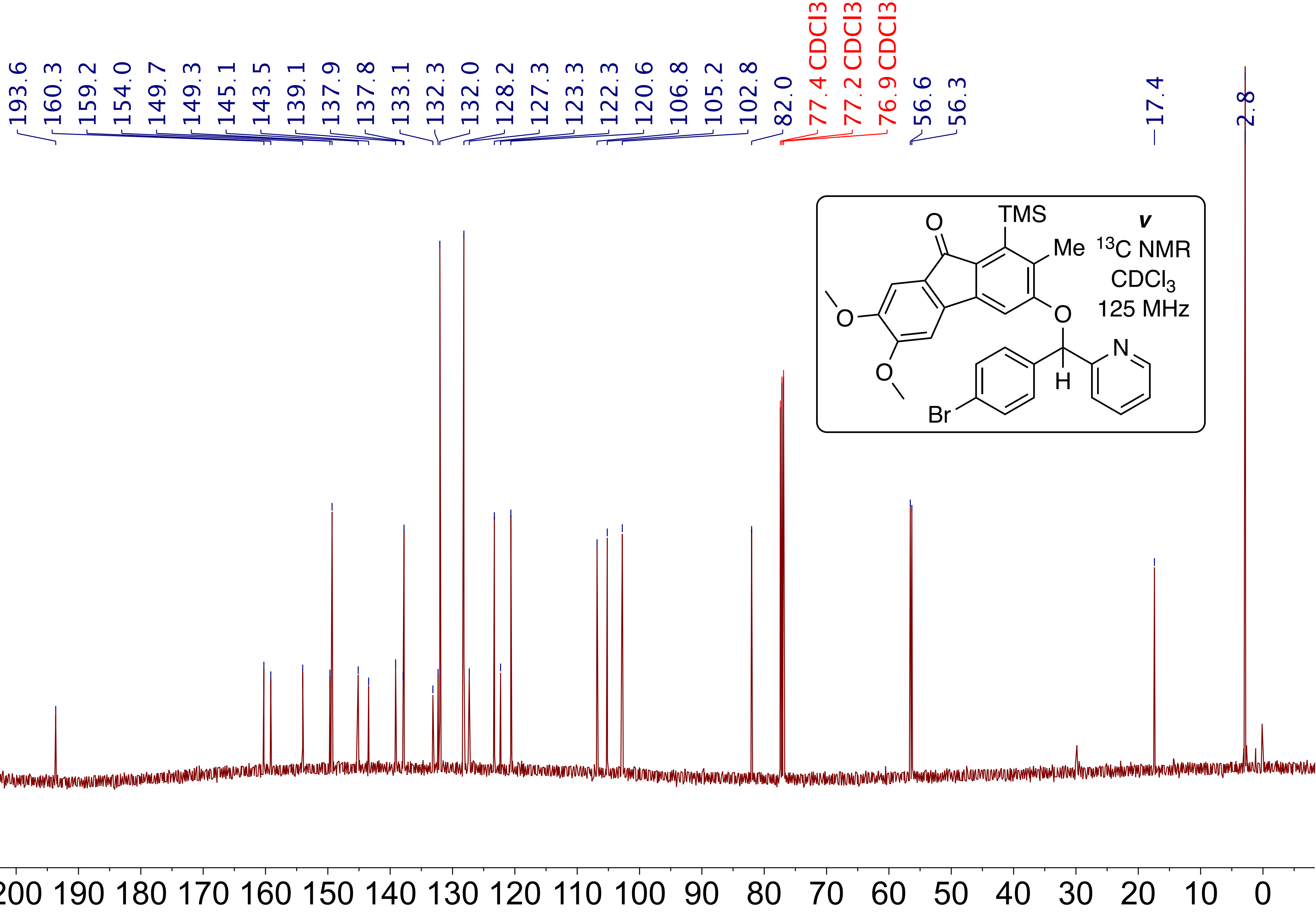
193.6
160.3
159.2
154.0
149.7
149.3
145.1
143.5
139.1
137.9
137.8
133.1
132.3
132.0
128.2
127.3
123.3
122.3
120.6
106.8
105.2
102.8
82.0
77.4 CDCl3
77.2 CDCl3
76.9 CDCl3
56.6
56.3
17.4
2.8
O
TMS
Me
O
O
O
H
N
Br
v
13C NMR
CDCl3
125 MHz
200 190 180 170 160 150 140 130 120 110 100 90 80 70 60 50 40 30 20 10 0
